# Utility of Large Language Models for Health Care Professionals and Patients in Navigating Hematopoietic Stem Cell Transplantation: Comparison of the Performance of ChatGPT-3.5, ChatGPT-4, and Bard

**DOI:** 10.2196/54758

**Published:** 2024-05-17

**Authors:** Elisabetta Xue, Dara Bracken-Clarke, Giovanni Maria Iannantuono, Hyoyoung Choo-Wosoba, James L Gulley, Charalampos S Floudas

**Affiliations:** 1 Center for Immuno-Oncology Center for Cancer Research, National Cancer Institute National Institutes of Health Bethesda, MD United States; 2 Genitourinary Malignancies Branch Center for Cancer Research, National Cancer Institute National Institutes of Health Bethesda, MD United States; 3 Biostatistics and Data Management Section Center for Cancer Research, National Cancer Institute National Institutes of Health Bethesda, MD United States

**Keywords:** hematopoietic stem cell transplant, large language models, chatbot, chatbots, stem cell, large language model, artificial intelligence, AI, medical information, hematopoietic, HSCT, ChatGPT

## Abstract

**Background:**

Artificial intelligence is increasingly being applied to many workflows. Large language models (LLMs) are publicly accessible platforms trained to understand, interact with, and produce human-readable text; their ability to deliver relevant and reliable information is also of particular interest for the health care providers and the patients. Hematopoietic stem cell transplantation (HSCT) is a complex medical field requiring extensive knowledge, background, and training to practice successfully and can be challenging for the nonspecialist audience to comprehend.

**Objective:**

We aimed to test the applicability of 3 prominent LLMs, namely ChatGPT-3.5 (OpenAI), ChatGPT-4 (OpenAI), and Bard (Google AI), in guiding nonspecialist health care professionals and advising patients seeking information regarding HSCT.

**Methods:**

We submitted 72 open-ended HSCT–related questions of variable difficulty to the LLMs and rated their responses based on consistency—defined as replicability of the response—response veracity, language comprehensibility, specificity to the topic, and the presence of hallucinations. We then rechallenged the 2 best performing chatbots by resubmitting the most difficult questions and prompting to respond as if communicating with either a health care professional or a patient and to provide verifiable sources of information. Responses were then rerated with the additional criterion of language appropriateness, defined as language adaptation for the intended audience.

**Results:**

ChatGPT-4 outperformed both ChatGPT-3.5 and Bard in terms of response consistency (66/72, 92%; 54/72, 75%; and 63/69, 91%, respectively; *P*=.007), response veracity (58/66, 88%; 40/54, 74%; and 16/63, 25%, respectively; *P*<.001), and specificity to the topic (60/66, 91%; 43/54, 80%; and 27/63, 43%, respectively; *P*<.001). Both ChatGPT-4 and ChatGPT-3.5 outperformed Bard in terms of language comprehensibility (64/66, 97%; 53/54, 98%; and 52/63, 83%, respectively; *P*=.002). All displayed episodes of hallucinations. ChatGPT-3.5 and ChatGPT-4 were then rechallenged with a prompt to adapt their language to the audience and to provide source of information, and responses were rated. ChatGPT-3.5 showed better ability to adapt its language to nonmedical audience than ChatGPT-4 (17/21, 81% and 10/22, 46%, respectively; *P*=.03); however, both failed to consistently provide correct and up-to-date information resources, reporting either out-of-date materials, incorrect URLs, or unfocused references, making their output not verifiable by the reader.

**Conclusions:**

In conclusion, despite LLMs’ potential capability in confronting challenging medical topics such as HSCT, the presence of mistakes and lack of clear references make them not yet appropriate for routine, unsupervised clinical use, or patient counseling. Implementation of LLMs’ ability to access and to reference current and updated websites and research papers, as well as development of LLMs trained in specialized domain knowledge data sets, may offer potential solutions for their future clinical application.

## Introduction

The applications of large language model (LLM)–based chatbots, artificial intelligence tools trained to understand, interact with, and produce human-readable text, are garnering increasing interest in many fields. In medicine, LLMs are successfully passing board examinations [1-3] and show potential in information retrieval and finer conceptual application [4]. LLMs are accessible to health care professionals and patients; therefore, their ability to deliver complex medical information is of particular interest; so far, several applications have been explored, including patients’ education [5], patient-trial matching [6], administrative tasks, and training purposes [7].

Hematopoietic stem cell transplantation (HSCT) is a complex medical field requiring extensive knowledge, background, and training to practice successfully and can be challenging for the nonspecialist audience to comprehend. Here, we evaluated the performance of different chatbots in answering HSCT-related questions and assessed their reliability and verifiability, with the aim to determine which LLM can best assist the nonspecialists, including nonhematology medical professionals but also patients and caregivers, in navigating this field.

## Methods

**Figure 1:**
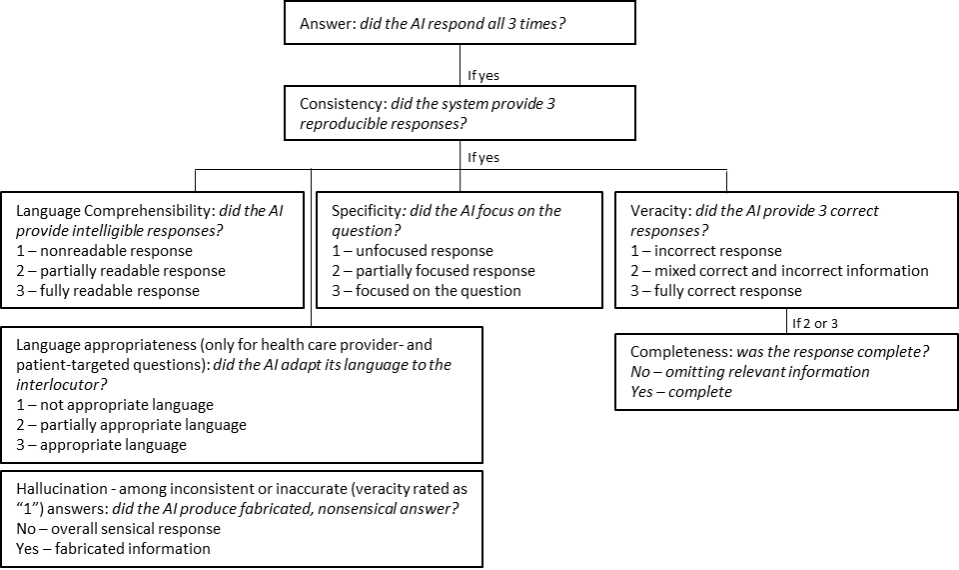
Rating system. Each question was submitted 3 times and rated according to the rating system reported. AI: artificial intelligence.

We compared the applicability to HSCT of 3 LLM chatbots, ChatGPT-3.5 (OpenAI), ChatGPT-4 (OpenAI), and Bard (Google AI) that were prominent and widely available at the time of the study design (July 2023), by assessing their responses to HSCT-related questions. ChatGPT-3.5 and ChatGPT-4 share a similar architecture, but the former is a free, easily accessible platform, whereas the second is subscription only, was released more recently, and advertised for having better performance; therefore, we included both to examine the difference in their performance. We selected four HSCT-related topics: (1) drugs (mechanisms of action and toxicities), (2) transplant indications and conditioning platforms, (3) infectious, and (4) noninfectious complications. For each topic, we generated 18 open-ended questions, with 3 levels of difficulty ranging from “easy,” testing superficial factual knowledge (eg, drug toxicities), to “difficult,” testing complex clinical scenarios (eg, inferring causative drug from a toxicity and guiding subsequent patient-tailored management; see Materials and Methods section in Multimedia Appendix 1 for the complete list of submitted questions). All questions were submitted between July 14 and 18, 2023, responses were referenced from the 7th edition European Bone Marrow Transplantation Handbook [8], Lexicomp [9], and BeTheMatch [10] reviewed on July 13, 2023. Each question was submitted 3 times consecutively, without providing feedback to the chatbot: if the chatbot declined to answer any of the 3 submissions, no further evaluation was conducted for that question. If it responded all 3 times, we evaluated the responses for consistency, defined as the ability to convey the same information at each submission. If the 3 responses were consistent, we scored them together for (1) veracity, defined as correctness of the information, (2) language comprehensibility, defined as clarity of the output, and (3) specificity, defined as focus on the question: each variable was rated from “1” (low performance) to “3” (best performance); analyses were then conducted comparing ratings “1 and 2” versus “3.” Inconsistent answers were not analyzed further. In addition, answers that scored “2” or “3” in veracity were evaluated for completeness to assess for lack of relevant information. Finally, we assessed for *hallucinations*, defined as nonsensical, fabricated information [11], among incorrect (rated as “1” in veracity) and inconsistent answers (Figure 1). For each step, 2 physicians (EX and DBC) independently graded the answers and reached consensus for any discrepancy; interrater reliability was evaluated through Cohen κ statistic. Average answer word count was also calculated.

Subsequently, after identifying the 2 overall best performing LLMs, we aimed to determine their utility as an information resource for nonspecialist audiences: we rechallenged them by resubmitting the “difficult” questions, prompting the chatbots to respond as if communicating with either a health care professional or a patient and to provide clear reference sources and concise responses. Answers were then regraded, as described above, and additionally assessed for language appropriateness, defined as language adaptation for the intended audience, to evaluate the LLMs’ ability to convey the same information using either simple or more technical terminology.

Fisher exact, Wilcoxon rank sum, and Kruskal-Wallis tests were used for categorical and continuous variables, respectively. All the statistical analyses were exploratory and performed using R (version 4.3.1; R Foundation Statistical Computing).

This study was exempt from ethical review since no human subjects were involved and 45 CFR part 46 did not apply.

## Results

**Figure 2:**
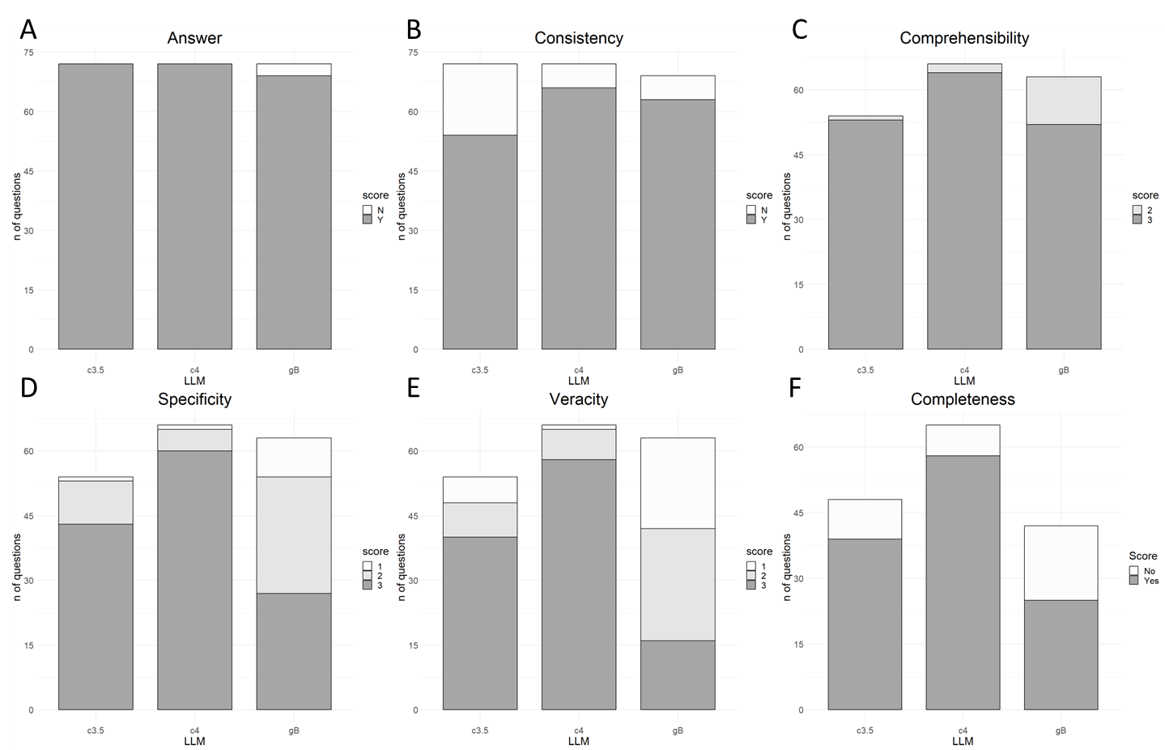
ChatGPT-3.5's, ChatGPT-4's, and Bard's performance. Seventy-two questions were submitted to each LLM. (A) Number of questions answered 3 times, (B) number of answers consistent with each other, (C) comprehensibility, (D) specificity, and (E) veracity. Veracity, language comprehensibility, and specificity were evaluated among consistent responses, and were rated as “1” (poor performance), “2” (mediocre performance), or “3” (best performance), and (F) completeness was evaluated among responses with veracity rated as “2” or “3.” LLM: large language model.

Detailed questions and responses are included in Multimedia Appendix 1. Cohen κ ranged between moderate to near perfect agreement (Table S1 in Multimedia Appendix 1). Figure 2 and Table S2 in Multimedia Appendix 1 display LLMs’ performances; ChatGPT-3.5 and ChatGPT-4 responded to all questions, whereas Bard did not answer 3 of 72 (4%; *P*=.12, reporting “I'm not able to help with that, as I'm only a language model”) questions. ChatGPT-4 had the highest rate of consistent responses (66/72, 92%) followed by Bard (63/69, 91%) and ChatGPT-3.5 (54/72, 75%; *P*=.007).

Consistent responses were evaluated for veracity, language comprehensibility, and specificity. ChatGPT-4 performed best in terms of veracity, with 58 of 66 (88%) of answers considered correct (rated as “3”), followed by ChatGPT-3.5 (40/54, 74%) and Bard (16/63, 25%; *P*<.001). For instance, when asked why a female patient who received an allogeneic HSCT from a male donor develops leukemia with 46XY karyotype, only ChatGPT-4 and Bard recognized this as donor-derived leukemia, whereas ChatGPT-3.5 wrongly suggested relapse of patient’s original disease. Bard had the highest rate of incorrect responses (rated as “1,” 21/63, 33%), especially among “moderate” and “difficult” questions. The LLMs also proved ineffective at calculating well-established risk scores (eg, Hematopoietic Cell Transplantation–specific Comorbidity Index). With respect to completeness, ChatGPT-3.5 and ChatGPT-4 answers were deemed complete in >80% of evaluable cases compared to approximately 60% for Bard answers (*P*<.001).

For language comprehensibility, ChatGPT-3.5 and ChatGPT-4 performance was equivalent, with only <4% of the answers rated less than “3” (ie, less than fully comprehensible) compared to 18% (11/63) of Bard answers (*P*=.002), especially among “moderate” and “difficult” questions (data not shown). Specifically, Bard exhibited a trend of repetitive language, yielding a less clear output.

Regarding specificity, ChatGPT-4 responses were rated as “3” (ie, very focused on the topic) in >90% of cases, followed by ChatGPT-3.5 (43/54, 80%) and Bard (27/63, 43%). For instance, when asked which drugs should be administered before antithymocyte globulin, ChatGPT-3.5 and ChatGPT-4 correctly listed the premedication and its purpose, whereas Bard listed all the premedication’s side effects. Bard provided more specific answers to the “easy” questions, whereas ChatGPT-3.5 and ChatGPT-4 performed similarly across the difficulty levels (data not shown).

All exhibited episodes of hallucinations, with ChatGPT-3.5, ChatGPT-4, and Bard showing at least 1 hallucinated answer in 7 of 24 (29%), 3 of 7 (43%), and 14 of 27 (52%) of evaluable cases, respectively. ChatGPT-4 provided shorter answers, with a median of 213 (IQR 191-261) words per answer, followed by ChatGPT-3.5 with 247 (IQR 212-307) words, and Bard with 303 (IQR 260-384) words (*P*<.001).

Due to their overall better performance, ChatGPT-4 and ChatGPT-3.5 were selected for the assessment of audience-tailored information delivery. In the “health care professional-targeted” version, with respect to specificity, ChatGPT-3.5 more frequently yielded unfocused (ie, rated as “1” or “2”) answers compared to ChatGPT-4 (6/18, 33% and 0/21, 0% respectively); no noticeable differences were seen in other parameters, including language appropriateness (Table S3 in Multimedia Appendix 1). ChatGPT-3.5 did not provide verifiable information sources despite the prompt requiring them to do so, reporting “The information I provide is based on the knowledge I was trained on until September 2021”; ChatGPT-4 referenced scientific literature in 10 of 21 (48%) evaluable cases, frequently with relevant but out-of-date papers, or with inaccurate authorship, title, or Digital Object Identifier.

In the “patient-targeted” version (Table S4 in Multimedia Appendix 1), ChatGPT-4 yielded a higher rate of correct responses compared to ChatGPT-3.5, with 19 of 22 (86%) and 10 of 21 (48%) cases rated as “3” in veracity, respectively; however, ChatGPT-4 showed excessively technical language, with only 10 of 22 (46%) rated as “3” in language appropriateness compared to 17 of 21 (81%) for ChatGPT-3.5. No differences were seen in the other parameters. Both failed to return information sources but provided website resources targeted for patients in 62% (13/21) and 95% (21/22) of the cases, respectively. Notably, ChatGPT-3.5 returned several broken URLs, likely corresponding to no longer existing pages, while ChatGPT-4 tended to provide overly general websites for very specific queries (eg, American Cancer Society web page [12] for information on sinusoidal obstruction syndrome). Both consistently acknowledged the potential for case-to-case variability and recommended referring to the medical team for any case-specific questions.

## Discussion

The emergence of LLMs has expanded the accessibility of medical information to the general public [5,13]; however, their reliability remains of concern [14]. In our study, all 3 LLMs correctly answered most of the “easy” questions, but only ChatGPT-3.5 and ChatGPT-4 successfully addressed more complex scenarios, and both outperformed Bard in producing comprehensible and specific responses. However, all exhibited episodes of hallucinations; thus, the potential for mistakes in diagnosis and recommendations remains a major obstacle to their routine unsupervised use.

When testing LLMs as support learning tools for laypeople, ChatGPT-3.5 adopted a friendly tone and, interestingly, exhibited some degree of emotional support (eg, “I understand your concern” and “take care”), showing a greater ability in adjusting its language to the audience. Adapting language to the general community and avoiding technical jargon would be optimal tools for making complex information accessible to patients and caregivers. In our opinion, LLMs cannot replace effective patient-doctor communication but rather may potentially supplement it, eventually reducing the risk of misinformation from nonscientific websites and sources. However, in our experience, current LLMs failed to consistently provide specific and updated web-based references, likely due to ChatGPT’s then lack of real-time access to the internet, thus making their output frequently unverifiable by the reader.

From a physician’s perspective, LLMs cannot replace conferences or scientific literature but may effectively support personal learning. Unfortunately, limited access to current web-based data, errors in reporting peer-reviewed material, and failure to provide valid references severely compromise this application [15].

Our study has limitations, including the submission of each question 3 consecutive times, without opening a new chat session each time, potentially urging the chatbot to provide a different answer at each submission. Furthermore, we subjectively selected 3 among the most popular available LLMs, 2 of which are developed by the same company; as more are becoming available, our observation might not apply to other LLMs. Finally, this is a rapidly changing field: since the completion of our analysis, ChatGPT has gained access to real time internet data, and Bard was updated into Google Gemini, and thus might yield a different output if tested today.

In conclusion, our evaluation suggests that, given the higher rate of correct and focused responses provided, at the time of this analysis, ChatGPT-3.5 and ChatGPT-4 are not yet appropriate for routine, unsupervised clinical use for both the general population and health care providers, or patient counseling. Their use at present should only be considered under expert supervision or for research purposes. Nevertheless, because of the rapid progression and the clear potential of LLMs to revolutionize workflows in medicine, including specialized fields, we need to engage proactively with this technology. Implementation of LLMs’ ability to access and to reference current and updated websites and research papers, as well as the development of LLMs trained in specialized domain knowledge data sets, may offer potential solutions for their future clinical application.

## Appendix

Multimedia Appendix 1 Supplementary materials.

## Abbreviations

HSCT: hematopoietic stem cell transplantation
LLM: large language model
