# Supplementary material for: Utility of Large Language Models for Health Care Professionals and Patients in Navigating Hematopoietic Stem Cell Transplantation: Comparison of the Performance of ChatGPT-3.5, ChatGPT-4, and Bard
Source: J Med Internet Res. 2024 May 17;26:e54758. doi: 10.2196/54758 (PMC11143389; doi:10.2196/54758)
Supplement: Multimedia Appendix 1 [file jmir_v26i1e54758_app1.docx]

**Supplementary Materials**

Utility of Large Language Models for Healthcare Professionals and Patients in navigating Hematopoietic Stem Cell Transplant: Comparison of Chat-GPT3.5, Chat-GPT4 and Bard performance.

Elisabetta Xue1, Dara Bracken-Clarke1, Giovanni Maria Iannantuono1, Hyoyoung Choo-Wosoba2, James L. Gulley1, Charalampos S. Floudas1

1 Center for Immuno-Oncology, Center for Cancer Research, National Cancer Institute, National Institutes of Health, Bethesda, MD. ­2 Biostatistics and Data Management Section, Center for Cancer

Supplementary materials contain:

Section 1 – Materials and Methods

- Pool of 72 questions on hematopoietic stem cell transplants submitted to Chat-GPT3.5, Chat-GPT4 and Google Ba

Section 2 – Results

- Table S1. Cohen’s Kappa statistic for interrater agreement.
- Table S2. Performance of GPT3.5, Chat-GPT4 and Google Bard in answering hematology-related questions.
- Table S3. Performance of Chat-GPT3.5 and Chat-GPT4 in answering using an expert-targeted language.
- Table S4. Performance of Chat-GPT3.5 and Chat-GPT4 in answering using a patient-targeted language.
- Answers provided by Chat-GPT3.5, Chat-GPT4 and Google Bard

**Section 1. Materials and Methods**

**Pool of 72 questions submitted to Chat-GPT3.5, Chat-GPT4 and Google Bard**

**Drug: mechanism of action, toxicity, and interaction**

Easy

1. What are common side effects of cyclosporine?
2. What is the target of sirolimus?
3. Voriconazole is known to increase the serum level of which systemic immunosuppressant?
4. Which drugs are most often combined with calcineurin inhibitor as a graft-versus-host disease prophylaxis regimen?
5. What is the biochemical pathway and organ site for busulfan metabolism?
6. How many times a week should be TMP/SMX given to prevent PCP in a patient undergoing HSCT?

Moderate

1. A patient with acute graft versus host disease failed to respond to steroid therapy. A treatment approved by FDA for refractory acute GVHD therapy is started. This drug is commonly used also in which other setting?
2. A patient develops dry cough after receiving allogeneic HSCT. Infectious causes are excluded. What is drug commonly used during conditioning associated with lung injury?
3. An adult patient undergoing allogeneic HSCT developed fever and dry cough. CT scan reveals a nodular lesion with perilesional ground glass changes in the left lower lobe; subsequent bronchoalveolar lavage demonstrates galactomannan positivity. Intravenous treatment is started for this condition. Within few days, progressive hypokalemia is documented. Which drug is the patient likely receiving and what is the standard dose?
4. A patient is admitted for administration of conditioning regimen. His creatinine clearance is 50 ml/min. The dose of which drug commonly used in conditioning regimen should be reduced?
5. A patient is about to receive anti thymocyte globulin before allogeneic HSCT. Which drugs should be given immediately prior its administration and why?
6. What is fludarabine half-life?

Difficult

1. A patient with acute lymphoblastic leukemia treated with inotuzumab is undergoing haploidentical HSCT. For which hepatic complication is he at risk for and what can be done do minimize this risk?
2. A patient received rituximab prior to allogeneic HSCT, which subsequently impaired B cell reconstitution. Other than antimicrobial prophylaxis, what can be administered to reduce risk of infection?
3. A patient is receiving a pill for a newly diagnosed acute lymphoblastic leukemia Philadelphia positive. He develops periorbital swelling. Which drug is he likely receiving?
4. A patient is about to start conditioning with busulfan and fludarabine, what drug should be given for antiemetic purposes?
5. A patient undergoing allogeneic HSCT refers vivid dreams and visual hallucinations. One oral drug used for antimicrobial prophylaxis is discontinued, and no other episodes occur. What most likely is the drug, and what is its’ mechanism of actions?
6. A patient with chronic graft versus host disease requires second line treatment. A drug commonly used in chronic lymphocytic leukemia is started. The patient is scheduled for dental surgery. Should the drug be discontinued and, if yes, when?

**HSCT platform**

Easy

1. What is GVHD prophylaxis?
2. What is treosulfan?
3. What is the standard conditioning regimen for multiple myeloma undergoing autologous HSCT and what is the recommended dose?
4. Post-transplant cyclophosphamide as graft versus host prophylaxis is typically given when after HSCT?
5. Which sample types are typically utilized for HLA typing in healthy donors?
6. A patient receives high dose cytarabine. Which eye drops are recommended and why?

Moderate

1. An individual wants to register to the Be the Match Registry for volunteer hematopoietic cell donors. During the medical history evaluation, he tells the physician about his prior history of prostate cancer that required systemic chemotherapy. Subsequent follow up was negative. Can this person be registered?
2. A 48-year-old patient diagnosed with multiple myeloma is in complete remission after second autologous transplant. What should be offered to this patient and for how long?
3. Should autologous HSCT ever be considered for a patient with multiple sclerosis?
4. A patient and his peripheral blood stem cell donor have a major ABO mismatch. Is the donor suitable? Which other tests are required?
5. A 32-year-old patient is considered for allogeneic HSCT for acute myeloid leukemia with intermediate risk cytogenetic in first complete remission. What is the disease risk index score for this patient and what is the overall survival at 2 years?
6. A patient undergoing HSCT has DSA. He is at risk for what specific complication?

Difficult

1. What is the best first treatment option for a 25-year-old patient diagnosed with severe aplastic anemia?
2. A 32-year-old patient is diagnosed with NPM1 mutated acute myeloid leukemia with normal karyotype. He achieved complete molecular remission after induction. After second consolidation cycle, minimal residual disease returns turn positive. What is the best subsequent approach?
3. A male patient with hematologic malignancy is offered allogeneic HSCT. He has no HLA matched related donors. Two unrelated donors are being considered, both fully matched and with same AB0 group: the first is a 20-year-old male, the second is a 42-year-old woman with history of 2 pregnancies. Which one should be preferred and why?
4. The HSCT team is evaluating a cord blood transplant for an adult patient with hematologic malignancy. Which cord blood parameters should be evaluated?
5. A 32-year-old patient is considered for allogeneic HSCT for acute myeloid leukemia. His BMI is 30. His medical history is unremarkable other than for previous basal cell cancer that did not require systemic treatment. What is the HCT CI score?
6. A patient with poor graft function after allogeneic HSCT is offered a stem cell boost. What would be the advantage of selecting CD34?

**Infectious complication**

Easy

1. Post-transplant lymphoproliferative disorder is often caused by which virus and what is the patient donor serology most commonly associated with this condition?
2. A patient undergoing allogeneic HSCT is not compliant with the sulfamethoxazole / trimethoprim prophylaxis. He might be at increased for what complication?
3. What is the target of letermovir?
4. Which drug is FDA-approved for cytomegalovirus prophylaxis in allogeneic HSCT recipients (without CMV reactivation)?
5. Which of the human herpes viruses has been more often associated with encephalitis, especially among cord blood transplant recipients?
6. A patient is about to receive rituximab. Which serology needs to be assessed prior to treatment start?

Moderate

1. A patient undergoing HSCT develops disseminated vesicular rash during inpatient care. He has not been compliant with his medications. Which contact precaution should be used?
2. A 56yo male patient undergoing HSCT developed fever, dry cough and shortness of breath. Chest CT scan shows bilateral ground glass pattern. A swab for respiratory viruses is performed and is positive for respiratory syncytial virus. What drug could potentially be offered to this patient?
3. Is monitoring adenovirus on fecal samples recommended in adults undergoing allogeneic HSCT?
4. A 56-year-old male patient undergoing HSCT develops hemorrhagic cystitis. Urine analysis is performed and is negative for BK virus. What other virus could be a causative agent and what could be its’ potential treatment?
5. Does asymptomatic BK viruria be treated in HSCT recipients?
6. What are third party virus specific T lymphocytes for patients undergoing HSCT?

Difficult

1. A patient undergoing HSCT develops fever and X ray scan shows pneumonia. Blood cultures grow gram positive bacteria. What anti-gram-positive intravenous drug should be avoided in treating his pneumonia?
2. A 56-year-old patient undergoing HSCT is tested for HHV-6, which is positive with low viral load. The result is confirmed in a second sample. The patient is asymptomatic. What might explain this situation?
3. Is there any role for rectal swab screening before allogeneic HSCT?
4. Which vaccines are contraindicated early after allogeneic HSCT?
5. A patient develops skin rash and high-grade fever 2 weeks after cord blood transplant. Infectious causes are excluded. His blood counts are ANC 0, Hb 8.0, PLT 21,000. What is the most probable cause, and what is the likely pathophysiology?
6. A patient develops multiple lymphadenopathy few weeks after receiving HSCT. EBV is positive on peripheral blood with high viral load. Which drug will he likely be offered?

**Noninfectious complication**

Easy

1. What are the clinical features of acute graft versus host disease?
2. A patient at day 30 after HSCT rapidly develops gastrointestinal disturbance and diarrhea, with a daily stool output of 2L. No lab abnormalities are reported. Physical exam is unremarkable. What is the most likely diagnosis and what is the first line treatment?
3. What is the success rate of systemic steroid in acute graft versus host disease?
4. During aplasia, in a patient undergoing HSCT, are granulocyte transfusions a standard of care?
5. What is the BEDI score for a patient suffering from hemorrhagic cystitis with hematuria and visible clots?
6. What is poor graft function?

Moderate

1. What does the HCT-CI score measure and which factor do it include?
2. A patient undergoing HSCT develops uncontrolled hypertension. He claims progressive vision disturbances. EEG shows nonspecific bilateral abnormalities. A brain MRI is performed. What is the MRI likely to show and what is the diagnosis?
3. A female patient with acute myeloid leukemia undergoes allogeneic HSCT from a male donor. After 12 months, bone marrow analysis shows blast percentage of 40%. Cytogenetic analysis shows that all cells are 46XY. What is the most likely explanation?
4. A O positive patient receives an HSC transplant from an A positive donor. For which specific complication is he at risk in this setting?
5. A patient undergoing HSCT is shown to have progressive hypertension, decreased renal function, and worsening anemia and thrombocytopenia. Cyclosporine is temporarily discontinued. Which other laboratory finding on the blood smear are often associated with this condition?
6. What is the difference between primary and secondary graft failure and what are the possible treatments?

Difficult

1. A patient with acute myeloid leukemia relapses 12 months after haploidentical transplant. Loss of patient-donor mismatched HLA is demonstrated in the leukemic cells. Should donor lymphocytes infusion be offered?
2. A patient with AML relapses after haploidentical HSCT. Uniparental disomy of short arm of chromosome 6 is detected in leukemic cell. What is the effect of this loss and how does it impact treatment options?
3. A 56-year-old male patient undergoing allogeneic HSCT for hematologic malignancy develops rapidly progressive ascites, weight gain, increased bilirubin level and painful hepatomegaly. What drug could be the most effective for this condition?
4. A patient with AML is offered allogeneic HSCT. His karyotype at diagnosis showed the subsequent alterations: t(1,2), -7. What is the prognosis at 5 years?
5. A 56-year-old male patient undergoing allogeneic HSCT for hematologic malignancy develop rapidly progressive ascites, weight gain, increased bilirubin level and painful hepatomegaly. Which transplant related factors have been suggested to increase the risk for this condition?
6. A patient is a candidate to receive donor lymphocytes infusion. What does the dosing of DLI depend on?

**Section 2. Results**

**Table S1.** Cohen’s Kappa statistic for interrater agreement.

| **Variable** | **Cohen’s Kappa** | **% of agreement** | **N of questions** |
| --- | --- | --- | --- |
| **Consistency** | 0.77 | 93.9 | 213 |
| **Veracity** | 0.71 | 83.8 | 173 |
| **Comprehensibility** | 0.49 | 93.6 | 173 |
| **Specificity** | 0.45 | 70.5 | 173 |
| **Completeness** | 0.51 | 83.7 | 141 |

Interrater agreement between physicians was evaluated per each variable by computing the Cohen’s Kappa statistic, showing moderate to near perfect agreement.

**Table S2.** Performance of Chat-GPT3.5, Chat-GPT4 and Google Bard (Bard) in answering hematopoietic stem cell transplant-related questions.

| **Variable** | **GPT-3.5 n (%)** | **GPT-4 n (%)** | **Bard**  **n (%)** | **GPT-4 vs GPT-3.5 vs Bard** |
| --- | --- | --- | --- | --- |
| **Answer** | | | | |
| Yes | 72 (100%) | 72 (100%) | 69 (95.8%) | *P = .12* |
| **Consistency** | | | | |
| Yes | 54 (75%) | 66 (91.7%) | 63 (91.3%) | ***P = .007*** |
| **Veracity** | | | | |
| Rated as “1” | 6 (11.1%) | 1 (1.5%) | 21 (33.3%) | ***P < .001*** |
| Rated as “2” | 8 (14.8%) | 7 (10.6%) | 26 (41.3%) |  |
| Rated as “3” | 40 (74.1%) | 58 (87.9%) | 16 (25.4%) |  |
| **Language Comprehensibility** | | | | |
| Rated as “1” | 0 (0%) | 0 (0%) | 0 (0%) | ***P = .002*** |
| Rated as “2” | 1 (1.9%) | 2 (3%) | 11 (17.5%) |  |
| Rated as “3” | 53 (98.1%) | 64 (97%) | 52 (82.5%) |  |
| **Specificity** | | | | |
| Rated as “1” | 1 (1.9%) | 1 (1.5%) | 9 (14.3%) | ***P < .001*** |
| Rated as “2” | 10 (18.5%) | 5 (7.6%) | 27 (42.9%) |  |
| Rated as “3” | 43 (79.6%) | 60 (90.9%) | 27 (42.9%) |  |
| **Completeness** | | | | |
| No | 9 (18.7%) | 7 (10.8%) | 17 (40.5%) | ***P < .001*** |
| Yes | 39 (81.3%) | 58 (89.2%) | 25 (59.5%) |  |
| **Hallucination** | | | | |
|  | 7 (29.2%) | 3 (42.9%) | 14 (51.8%) | *P = .28* |
| **Word Countsper answer** | | | | |
|  | 247  (113 - 485.7) | 213.2  (117.3 - 347) | 302.8  (34.7 - 665) | ***P < .001*** |

Seventy-two questions on hematopoietic stem cell transplant were submitted to three large language models; answers were rated as “1” (poor performance), “2” (mediocre performance) or “3” (best performance). Veracity, language comprehensibility, and specificity were evaluated among consistent responses. For variables “veracity”, “language comprehensibility” and “specificity”, analyses were performed comparing answers rated “1 and 2” versus ”3”. Completeness was evaluated among responses with veracity rated as “2” or “3”. Hallucinations was evaluated among responses rated as inconsistent responses or responses for which veracity was rated as “1”.

**Table S3.** **Performance of Chat-GPT3.5 and Chat-GPT4 in answering hematopoietic stem cell transplant-related questions using a healthcare professional-targeted language.**

| **Variable** | **GPT-3.5 n (%)** | **GPT-4 n (%)** | **GPT-4 vs GPT-3.5 vs Bard** |
| --- | --- | --- | --- |
| **Answers** | | | |
| Yes | 24 (100%) | 24 (100%) |  |
| **Consistency** | | | |
| Yes | 18 (75%) | 21 (87.5%) | *P = .46* |
| **Veracity** | | | |
| Rated as “1” | 2 (11.1%) | 2 (9.5%) | *P = .16* |
| Rated as “2” | 6 (33.5%) | 2 (9.5%) |  |
| Rated as “3” | 10 (55.6%) | 17 (81%) |  |
| **Language Comprehensibility** | | | |
| Rated as “1” | 0 (0%) | 0 (0 %) | *P = 1* |
| Rated as “2” | 1 (5.6 %) | 1 (4.8 %) |  |
| Rated as “3” | 17 (94.4%) | 20 (95.2 %) |  |
| **Specificity** | | | |
| Rated as “1” | 0 (0 %) | 0 (0 %) | ***P = .006*** |
| Rated as “2” | 6 (33.3 %) | 0 (0 %) |  |
| Rated as “3” | 12 (66.7%) | 21 (100 %) |  |
| **Language Appropriateness** | | | |
| Rated as “1” | 1 (5.6 %) | 0 (0 %) | *P = .11* |
| Rated as “2” | 5 (27.8 %) | 2 (9.5 %) |  |
| Rated as “3” | 12 (66.7 %) | 19 (90.5 %) |  |
| **Completeness** | | | |
| No | 6 (37.5%) | 2 (10.5 %) | *P = .11* |
| Yes | 10 (67.5%) | 17 (89.5 %) |  |

Twenty-four “difficult” questions on hematopoietic stem cell transplant were submitted to ChatGPT3.5 and ChatGPT4 prompting to adapt the language as if communicating to a healthcare provider; for variables “veracity”, “language comprehensibility”, “specificity” and “language appropriateness” answers were rated as “1” (poor performance), “2” (mediocre performance) or “3” (best performance); analyses were performed comparing answers rated as “1” and “2” versus “3”. Veracity, language comprehensibility, specificity and language appropriateness were evaluated among consistent responses. Completeness was evaluated among responses with veracity rated as “2” or “3”. Hallucinations was evaluated among responses rated as inconsistent or responses for which veracity was rated as “1”.

**Table S4. Performance of Chat-GPT3.5 and Chat-GPT4 in answering hematopoietic stem cell transplant-related questions using a patient-targeted language.**

| **Variable** | **GPT-3.5 n (%)** | **GPT-4 n (%)** | **GPT-4 vs GPT-3.5 vs Bard** |
| --- | --- | --- | --- |
| **Answers** | | | |
| Yes | 24 (100%) | 24 (100%) |  |
| **Consistency** | | | |
| Yes | 21 (87.5%) | 22 (91.7%) | *P = 1* |
| **Veracity** |  |  |  |
| Rated as “1” | 5 (23.8%) | 2 (9.1%) | ***P < .001*** |
| Rated as “2” | 6 (28.6%) | 1 (4.5%) |  |
| Rated as “3” | 10 (47.6%) | 19 (86.4%) |  |
| **Language Comprehensibility** | | | |
| Rated as “1” | 0 (0%) | 0 (%) | *P = 1* |
| Rated as “2” | 1 (4.8%) | 1 (4.5%) |  |
| Rated as “3” | 20 (95.2%) | 21 (95.5%) |  |
| **Specificity** | | | |
| Rated as “1” | 0 (0%) | 0 (0%) | *P = .23* |
| Rated as “2” | 0 (0%) | 3 (13.6%) |  |
| Rated as “3” | 21 (100%) | 19 (86.4%) |  |
| **Language Appropriateness** | | | |
| Rated as “1” | 0 (0%) | 1 (4.5%) | ***P = .03*** |
| Rated as “2” | 4 (19%) | 11 (50%) |  |
| Rated as “3” | 17 (81%) | 10 (45.5%) |  |
| **Completeness** | | | |
| No | 7 (43.8%) | 5 (25%) | *P = .30* |
| Yes | 9 (56.2%) | 15 (75%) |  |

Twenty-four “difficult” questions on hematopoietic stem cell transplant were submitted to ChatGPT3.5 and ChatGPT4 prompting to adapt the language as if communicating to a patient. For variables “veracity”, “language comprehensibility”, “specificity” and “language appropriateness” answers were rated as “1” (poor performance), “2” (mediocre performance) or “3” (best performance). Analyses were performed comparing answers rated as “1” and “2” versus “3”. Veracity, language comprehensibility, specificity and language appropriateness were evaluated among consistent responses. Completeness was evaluated among responses with veracity rated as “2” or “3”. Hallucinations was evaluated among responses rated as inconsistent or responses for which veracity was rated as

****Answers provided by Chat-GPT3.5, Chat-GPT4 and Google Bard****

| ****Chat-GPT3.5**** | | | |
| --- | --- | --- | --- |
| **Section 1 – drug mechanism of action, toxicity and interaction** | | | |
| D1 | Cyclosporine is a medication that is primarily used to suppress the immune system and prevent organ rejection in transplant recipients. It is also used in the treatment of various autoimmune conditions. While cyclosporine can be effective in managing these conditions, it may also cause several side effects. Some common side effects of cyclosporine include:  1. Gastrointestinal Issues: These can include nausea, vomiting, diarrhea, and abdominal discomfort. These symptoms may improve with time or with dose adjustments.  2. High Blood Pressure (Hypertension): Cyclosporine can cause an increase in blood pressure. Regular monitoring of blood pressure is important, and additional medications may be required to control it.  3. Kidney Problems: Cyclosporine can affect kidney function and may cause decreased urine output, increased serum creatinine levels, or even kidney damage. Regular monitoring of kidney function is necessary.  4. Increased Risk of Infections: Since cyclosporine suppresses the immune system, it can increase the risk of infections. Common infections include respiratory tract infections, urinary tract infections, and skin infections.  5. Tremors and Shaking: Some individuals may experience tremors or shaking of the hands, fingers, or other parts of the body. These involuntary movements are known as tremors.  6. Headache: Headaches are a relatively common side effect of cyclosporine treatment. They are typically mild and can often be managed with over-the-counter pain relievers.  7. Increased Hair Growth: Some people may notice increased hair growth, particularly on the face or other parts of the body. This side effect is generally reversible once cyclosporine is discontinued.  8. Gum Hyperplasia: Cyclosporine can cause overgrowth of gum tissue, leading to swollen and enlarged gums. Regular dental care and good oral hygiene are important to minimize this effect.  It's important to note that the side effects of cyclosporine can vary from person to person, and not everyone will experience all of these side effects. If you are taking cyclosporine and experiencing any concerning side effects, it is important to contact your healthcare provider for further evaluation and guidance. | Cyclosporine is a medication that is primarily used to suppress the immune system and prevent organ rejection in transplant recipients. It is also used in the treatment of various autoimmune conditions. While cyclosporine can be effective in managing these conditions, it may also cause several side effects. Some common side effects of cyclosporine include:  1. Gastrointestinal Issues: These can include nausea, vomiting, diarrhea, and abdominal discomfort. These symptoms may improve with time or with dose adjustments.  2. High Blood Pressure (Hypertension): Cyclosporine can cause an increase in blood pressure. Regular monitoring of blood pressure is important, and additional medications may be required to control it.  3. Kidney Problems: Cyclosporine can affect kidney function and may cause decreased urine output, increased serum creatinine levels, or even kidney damage. Regular monitoring of kidney function is necessary.  4. Increased Risk of Infections: Since cyclosporine suppresses the immune system, it can increase the risk of infections. Common infections include respiratory tract infections, urinary tract infections, and skin infections.  5. Tremors and Shaking: Some individuals may experience tremors or shaking of the hands, fingers, or other parts of the body. These involuntary movements are known as tremors.  6. Headache: Headaches are a relatively common side effect of cyclosporine treatment. They are typically mild and can often be managed with over-the-counter pain relievers.  7. Increased Hair Growth: Some people may notice increased hair growth, particularly on the face or other parts of the body. This side effect is generally reversible once cyclosporine is discontinued.  8. Gum Hyperplasia: Cyclosporine can cause overgrowth of gum tissue, leading to swollen and enlarged gums. Regular dental care and good oral hygiene are important to minimize this effect.  9. Skin Problems: Cyclosporine can sometimes cause skin-related side effects such as acne, rashes, or increased sensitivity to sunlight.  10. Fatigue: Some individuals may experience fatigue or general feelings of tiredness while taking cyclosporine.  It's important to note that the side effects of cyclosporine can vary from person to person, and not everyone will experience all of these side effects. If you are taking cyclosporine and experiencing any concerning side effects, it is important to contact your healthcare provider for further evaluation and guidance. | Common side effects of cyclosporine can include:  1. Gastrointestinal issues: Nausea, vomiting, diarrhea, and abdominal discomfort are common side effects. These symptoms may improve over time or with dose adjustments.  2. High blood pressure (hypertension): Cyclosporine can cause an increase in blood pressure. Regular monitoring of blood pressure is important, and additional medications may be required to manage it.  3. Kidney problems: Cyclosporine can affect kidney function, leading to decreased urine output, increased serum creatinine levels, and potential kidney damage. Regular kidney function monitoring is necessary.  4. Increased risk of infections: Cyclosporine suppresses the immune system, making individuals more susceptible to infections. Common infections can include respiratory tract infections, urinary tract infections, and skin infections.  5. Tremors and shaking: Some individuals may experience tremors or shaking of the hands, fingers, or other parts of the body. These tremors are involuntary movements.  6. Headache: Headaches are a common side effect of cyclosporine treatment. They are usually mild and can often be managed with over-the-counter pain relievers.  7. Increased hair growth: Cyclosporine may cause increased hair growth, particularly on the face or other areas of the body. This side effect is generally reversible after discontinuing cyclosporine.  8. Gum hyperplasia: Overgrowth of gum tissue, leading to swollen and enlarged gums, can occur with cyclosporine use. Regular dental care and good oral hygiene are important to minimize this effect.  9. Skin problems: Some individuals may experience skin-related side effects such as acne, rashes, or increased sensitivity to sunlight.  10. Fatigue: Cyclosporine can sometimes cause fatigue or general feelings of tiredness.  It's important to note that the occurrence and severity of these side effects can vary from person to person. If you experience any concerning side effects while taking cyclosporine, it's recommended to consult with your healthcare provider for further evaluation and guidance. |
| D2 | Sirolimus, also known as rapamycin, is a drug that belongs to a class of medications called mTOR inhibitors. The target of sirolimus is the mammalian target of rapamycin (mTOR), which is a protein kinase enzyme that plays a crucial role in regulating cell growth, proliferation, and survival.  By inhibiting mTOR, sirolimus interferes with various cellular processes involved in the growth and division of cells, including the signaling pathways that control protein synthesis and cell cycle progression. This inhibition of mTOR activity can have various effects depending on the specific context and cell type.  Sirolimus is primarily used as an immunosuppressant medication to prevent organ rejection in transplant recipients. It is also utilized in the treatment of certain types of cancers, such as renal cell carcinoma, and as a coating on drug-eluting stents to prevent restenosis (re-narrowing) of arteries after angioplasty. | Sirolimus, also known as rapamycin, is a medication that belongs to a class of drugs called mTOR inhibitors. The primary target of sirolimus is the mammalian target of rapamycin (mTOR), which is a protein kinase involved in the regulation of cell growth, proliferation, and survival.  Sirolimus works by binding to a protein called FK-binding protein 12 (FKBP12), forming a complex that inhibits mTOR. This inhibition of mTOR signaling has several effects on cellular processes, including the suppression of cell cycle progression, inhibition of protein synthesis, and modulation of immune responses.  Due to its ability to suppress the immune system and inhibit cell proliferation, sirolimus has been used in various medical applications. It is commonly prescribed as an immunosuppressant to prevent organ rejection in transplant patients. Sirolimus is also used in the treatment of certain types of cancer, such as renal cell carcinoma, and as a coating on coronary stents to prevent restenosis (re-narrowing of blood vessels). | Sirolimus, also known as rapamycin, is an immunosuppressive drug that has multiple targets within the body. Its primary target is a protein called mammalian target of rapamycin (mTOR). Sirolimus binds to the FK506-binding protein 12 (FKBP12) and forms a complex that inhibits the activity of mTOR.  mTOR is a key regulator of cell growth, proliferation, and survival. By inhibiting mTOR, sirolimus interferes with several cellular processes, including the activation of T cells and B cells of the immune system, which helps suppress the immune response.  Sirolimus is commonly used in organ transplantation to prevent rejection and is also used in certain types of cancer treatment and the management of various autoimmune disorders. |
| D3 | Voriconazole, an antifungal medication, is known to increase the serum levels of the systemic immunosuppressant called sirolimus (also known as rapamycin). The co-administration of voriconazole and sirolimus can result in an increased concentration of sirolimus in the bloodstream.  Voriconazole inhibits enzymes in the liver that metabolize sirolimus, specifically cytochrome P450 3A4 (CYP3A4). This inhibition reduces the breakdown of sirolimus, leading to higher levels of sirolimus in the body.  It is important to monitor sirolimus levels closely when these medications are used together, as the increased sirolimus concentration may require dosage adjustments or careful management to prevent potential side effects or toxicity associated with high sirolimus levels. Close collaboration between healthcare professionals, including physicians and pharmacists, is crucial when administering these medications concurrently. | I apologize for the incorrect information provided in my previous response. Voriconazole is known to decrease the serum levels of the systemic immunosuppressant called tacrolimus, not sirolimus.  Voriconazole, an antifungal medication, can inhibit the enzymes responsible for metabolizing tacrolimus, primarily cytochrome P450 3A4 (CYP3A4) and to a lesser extent, CYP2C19. As a result, the clearance of tacrolimus from the body is reduced, leading to increased tacrolimus levels in the bloodstream.  When voriconazole and tacrolimus are co-administered, it is important to monitor tacrolimus levels closely and adjust the tacrolimus dosage as necessary. The dose of tacrolimus may need to be decreased to avoid potential side effects or toxicity associated with elevated tacrolimus levels.  Thank you for pointing out the error, and I apologize for any confusion caused. | I apologize for the confusion caused by my previous responses. I appreciate your patience.  To clarify, voriconazole is known to increase the serum level of the systemic immunosuppressant called tacrolimus, not sirolimus. Co-administration of voriconazole with tacrolimus can result in elevated tacrolimus levels in the bloodstream.  Voriconazole inhibits the enzymes responsible for metabolizing tacrolimus, primarily cytochrome P450 3A4 (CYP3A4) and to a lesser extent, CYP2C19. This inhibition reduces the clearance of tacrolimus from the body, leading to increased tacrolimus concentrations.  It is crucial to closely monitor tacrolimus levels when voriconazole and tacrolimus are used together. Dose adjustments of tacrolimus may be necessary to maintain therapeutic levels and avoid potential side effects or toxicity associated with elevated tacrolimus levels.  Once again, I apologize for the previous incorrect information and any confusion it may have caused. |
| D4 | In the context of graft-versus-host disease (GVHD) prophylaxis regimens, calcineurin inhibitors are often combined with other medications to improve efficacy. The most commonly used combination is:  1. Calcineurin Inhibitor (CNI):  - Tacrolimus (Prograf) or Cyclosporine (Neoral, Sandimmune) are the two main CNIs used.  2. Methotrexate: Methotrexate is a folate antagonist that is frequently used in combination with a CNI. It is typically given as a short course of post-transplant prophylaxis.  3. Mycophenolate Mofetil (MMF): MMF is an immunosuppressant that inhibits DNA synthesis in T and B lymphocytes. It is often used as an alternative to methotrexate or in combination with it.  Other medications that may be used in GVHD prophylaxis, although less frequently, include:  - Antithymocyte Globulin (ATG): ATG is an immunosuppressive agent derived from animal sources, and it is sometimes used in combination with CNIs to reduce the risk of GVHD.  - Alemtuzumab (Campath): Alemtuzumab is a monoclonal antibody that targets CD52 on lymphocytes and is occasionally used as part of the GVHD prophylaxis regimen.  It's important to note that specific GVHD prophylaxis regimens can vary depending on the transplant center, the type of transplant (e.g., bone marrow, peripheral blood stem cell), the patient's risk factors, and the underlying disease. Therefore, the exact combination of medications may differ from case to case. Consultation with a medical professional or transplant specialist is necessary to determine the most appropriate GVHD prophylaxis regimen for an individual patient. | In the context of graft-versus-host disease (GVHD) prophylaxis, the standard regimen typically includes a calcineurin inhibitor (CNI) in combination with other immunosuppressive medications. The most commonly used CNI in this setting is either cyclosporine or tacrolimus.  When used for GVHD prophylaxis, CNIs are often combined with methotrexate or mycophenolate mofetil (MMF). These medications are known as additional immunosuppressive agents and are used to enhance the effectiveness of the CNI.  Other drugs that may be included in GVHD prophylaxis regimens, depending on the specific situation and protocols, include:  1. Antithymocyte globulin (ATG): ATG is a polyclonal antibody preparation that targets and depletes T cells, thereby reducing the risk of GVHD.  2. Steroids: Corticosteroids, such as prednisone or methylprednisolone, may be added to the regimen to further suppress the immune system and reduce the risk of GVHD.  3. Sirolimus (rapamycin): Sirolimus, which was mentioned earlier, can also be used in combination with CNIs as an alternative or adjunctive therapy for GVHD prophylaxis.  The specific choice of drugs and their combinations in a GVHD prophylaxis regimen may vary based on the transplant center's protocols, the patient's condition, and the type of transplant being performed (e.g., bone marrow transplant, stem cell transplant). It's important to consult with a medical professional for individualized treatment recommendations. | In the context of graft-versus-host disease (GVHD) prophylaxis regimens in allogeneic stem cell transplantation, calcineurin inhibitors (such as cyclosporine or tacrolimus) are commonly combined with other medications to provide more effective immunosuppression. The specific combination regimens may vary depending on the transplant center and individual patient factors, but here are some drugs that are frequently used in combination with calcineurin inhibitors:  1. Methotrexate: Methotrexate is an antimetabolite that is often used in combination with calcineurin inhibitors for GVHD prophylaxis. It is typically administered as a short course of intravenous (IV) infusions after transplantation.  2. Mycophenolate mofetil (MMF): MMF is an immunosuppressive medication that is often used in combination with calcineurin inhibitors. It is available in oral form and helps to suppress the immune system and prevent GVHD.  3. Anti-thymocyte globulin (ATG): ATG is derived from the serum of animals or humans and contains antibodies against human T cells. It is administered prior to transplantation to deplete T cells and reduce the risk of GVHD.  4. Sirolimus (Rapamycin): Sirolimus, as mentioned earlier, is an immunosuppressive drug that targets the mammalian target of rapamycin (mTOR). It may be used in combination with calcineurin inhibitors for GVHD prophylaxis, particularly in certain high-risk patients.  5. Steroids: Corticosteroids, such as prednisone or methylprednisolone, are often used as part of GVHD prophylaxis regimens. They have potent anti-inflammatory and immunosuppressive properties and can help prevent or treat GVHD.  It's important to note that the specific combination of drugs used for GVHD prophylaxis may vary based on factors such as the underlying disease, patient characteristics, and the transplant center's protocols. The dosage and duration of medication administration will also be determined by the healthcare team based on individual patient needs and response to treatment. |
| D5 | Busulfan undergoes metabolism primarily through a process called biotransformation in the liver. The primary pathway involved in busulfan metabolism is known as glutathione conjugation or glutathione S-transferase (GST)-mediated conjugation.  In the liver, busulfan is metabolized by glutathione S-transferases, specifically GSTA1 and GSTA2. These enzymes catalyze the conjugation of busulfan with glutathione, a tripeptide molecule consisting of glutamate, cysteine, and glycine. The reaction results in the formation of a glutathione-busulfan conjugate.  The glutathione-busulfan conjugate is further metabolized through a series of enzymatic reactions. It can undergo cleavage by gamma-glutamyltransferase, releasing busulfan and a cysteinylglycine conjugate. Busulfan can be further metabolized by the action of thioesterases, yielding tetrahydrothiophene-1-oxide and tetrahydrothiophene-1,1-dioxide.  These metabolites of busulfan are eventually eliminated from the body through urine and feces. It's worth noting that busulfan metabolism can be influenced by various factors, including genetic polymorphisms, drug interactions, and individual patient characteristics.  The primary organ site for busulfan metabolism is the liver, where the majority of drug metabolism occurs. The liver's enzymes, particularly the glutathione S-transferases, play a crucial role in the biotransformation of busulfan and its subsequent elimination from the body. | Busulfan is an alkylating agent commonly used in the context of hematopoietic stem cell transplantation (HSCT) as part of conditioning regimens. It is primarily metabolized through a process called biotransformation in the liver. The main biochemical pathway involved in busulfan metabolism is oxidative metabolism via the hepatic cytochrome P450 (CYP) enzymes.  Once administered, busulfan is rapidly absorbed and undergoes hepatic metabolism. It is predominantly metabolized by CYP3A4 and CYP2A6 enzymes, although other CYP enzymes, such as CYP2B6 and CYP2C19, may also contribute to its metabolism. These enzymes catalyze the conversion of busulfan into several metabolites, including 1,4-butanediol and tetrahydrothiophene, which are inactive and less toxic than the parent compound.  The liver is the primary organ responsible for busulfan metabolism, and it plays a crucial role in determining the pharmacokinetics and efficacy of the drug. The metabolism of busulfan can vary significantly between individuals due to genetic factors influencing the activity of CYP enzymes. Genetic polymorphisms in CYP3A4 and CYP2A6, for example, can affect the rate of busulfan metabolism and subsequently influence drug levels in the body.  It's worth noting that busulfan undergoes both Phase I (oxidation) and Phase II (conjugation) metabolism. After Phase I metabolism mediated by CYP enzymes, Phase II metabolism involves the conjugation of the oxidized metabolites with glutathione (GSH) to form busulfan-glutathione conjugates. These conjugates are further metabolized and eliminated from the body.  Overall, understanding the biochemical pathway and organ site of busulfan metabolism is important for optimizing drug dosing, monitoring drug levels, and minimizing toxicity during HSCT and other therapeutic applications of busulfan. | Busulfan is an alkylating agent commonly used in conditioning regimens prior to hematopoietic stem cell transplantation. It undergoes extensive metabolism in the body, primarily through hepatic metabolism. The primary pathway for busulfan metabolism is through a process known as hepatic microsomal oxidation.  In the liver, busulfan is metabolized by the cytochrome P450 (CYP) enzyme system, specifically the CYP2A6 and CYP3A4 enzymes. These enzymes catalyze the oxidation of busulfan, leading to the formation of several metabolites, including 3-hydroxybusulfan and tetrahydrothiophene. These metabolites are less pharmacologically active than the parent compound.  The metabolic pathway of busulfan involves two main steps:  1. Oxidation: Busulfan undergoes oxidative metabolism primarily through CYP2A6 and CYP3A4 enzymes, leading to the formation of 3-hydroxybusulfan and tetrahydrothiophene metabolites.  2. Conjugation: The metabolites, particularly 3-hydroxybusulfan, are further metabolized through conjugation with glutathione, resulting in the formation of busulfan-glutathione conjugates. These conjugates are then eliminated from the body through urine.  It's important to note that the exact metabolic pathways and relative contributions of specific enzymes may vary among individuals due to genetic polymorphisms and other factors. The liver is the primary organ responsible for busulfan metabolism, while the metabolites are eventually eliminated through urine. |
| D6 | The prevention of Pneumocystis jirovecii pneumonia (PCP) in patients undergoing hematopoietic stem cell transplantation (HSCT) typically involves the use of trimethoprim/sulfamethoxazole (TMP/SMX) prophylaxis. The dosing regimen for TMP/SMX can vary depending on the specific clinical situation and the underlying risk factors of the patient. However, a common prophylactic regimen for PCP in HSCT recipients is TMP/SMX given three times a week.  It's important to note that the exact dosing schedule should be determined by the patient's healthcare provider based on individual factors such as the patient's age, weight, renal function, and other concurrent medications. Therefore, it's always best to consult with a medical professional who has access to the patient's complete medical history and can provide personalized recommendations. | The recommended dosage and frequency of trimethoprim/sulfamethoxazole (TMP/SMX) for the prevention of Pneumocystis pneumonia (PCP) in patients undergoing hematopoietic stem cell transplantation (HSCT) can vary depending on the specific protocols and guidelines followed by healthcare providers. However, a commonly used regimen is as follows:  - Standard-risk patients: Typically receive TMP/SMX at a dose of 160 mg/800 mg (one double-strength tablet) orally, once daily, three times per week (e.g., Monday, Wednesday, and Friday) or on alternate days (e.g., Monday, Wednesday, Friday, Sunday).  - High-risk patients: High-risk patients may receive TMP/SMX at a higher dose, such as 160 mg/800 mg (one double-strength tablet) orally, once daily, seven days per week.  It is important to note that these guidelines are general recommendations, and the specific regimen may be tailored to the individual patient's needs based on factors such as the underlying condition, immune status, and the healthcare provider's clinical judgment. It is crucial to consult with a healthcare professional or follow the specific guidelines provided by the transplantation team to determine the appropriate dosing regimen for a patient undergoing HSCT. | The recommended prophylactic regimen for preventing Pneumocystis jirovecii pneumonia (PCP) in patients undergoing hematopoietic stem cell transplantation (HSCT) may vary depending on various factors, including the patient's risk factors and underlying condition. However, a commonly used regimen for PCP prophylaxis in HSCT patients involves trimethoprim/sulfamethoxazole (TMP/SMX), also known as cotrimoxazole.  In general, the standard recommendation for PCP prophylaxis with TMP/SMX in HSCT patients is to administer it three times a week. The specific dosing and duration may be adjusted based on the patient's risk factors and the center's protocols. It's important to consult with a healthcare professional or follow the guidelines of the transplant center to determine the most appropriate prophylactic regimen for a particular patient undergoing HSCT. |
| D7 | The drug approved by the FDA for refractory acute graft-versus-host disease (GVHD) therapy, when steroid therapy fails, is called Ruxolitinib (brand name Jakafi). Ruxolitinib is a Janus kinase (JAK) inhibitor that works by inhibiting specific enzymes involved in the signaling pathways of certain immune cells.  While Ruxolitinib is approved for refractory acute GVHD, it is also commonly used in another setting, which is the treatment of myelofibrosis. Myelofibrosis is a rare bone marrow disorder characterized by the replacement of healthy bone marrow with fibrous scar tissue, leading to the disruption of normal blood cell production.  Ruxolitinib has been shown to effectively reduce spleen enlargement, alleviate symptoms, and improve quality of life in patients with myelofibrosis. It is approved by the FDA for the treatment of intermediate or high-risk myelofibrosis, including primary myelofibrosis, post-polycythemia vera myelofibrosis, and post-essential thrombocythemia myelofibrosis.  In both refractory acute GVHD and myelofibrosis, Ruxolitinib has demonstrated efficacy in modulating the dysregulated immune response and reducing inflammatory cytokine production, thereby providing clinical benefit in these conditions. | The drug approved by the FDA for refractory acute graft-versus-host disease (GVHD) therapy, when steroid therapy fails, is called Ruxolitinib (brand name Jakafi). Ruxolitinib is a Janus kinase (JAK) inhibitor that works by inhibiting specific enzymes involved in the signaling pathways of certain immune cells.  In addition to its use in refractory acute GVHD, Ruxolitinib is also commonly used in another setting, which is the treatment of myelofibrosis. Myelofibrosis is a rare bone marrow disorder characterized by the replacement of healthy bone marrow with fibrous scar tissue, leading to the disruption of normal blood cell production.  Ruxolitinib has been shown to effectively reduce spleen enlargement, alleviate symptoms such as fatigue and abdominal discomfort, and improve quality of life in patients with myelofibrosis. It is approved by the FDA for the treatment of intermediate or high-risk myelofibrosis, including primary myelofibrosis, post-polycythemia vera myelofibrosis, and post-essential thrombocythemia myelofibrosis.  In both refractory acute GVHD and myelofibrosis, Ruxolitinib demonstrates its efficacy by modulating the dysregulated immune response and reducing inflammatory cytokine production, thereby providing clinical benefit in these conditions. | The drug approved by the FDA for refractory acute graft-versus-host disease (GVHD) therapy when steroid therapy fails is called Emapalumab (brand name Gamifant). Emapalumab is a monoclonal antibody that targets interferon gamma (IFN-γ), a cytokine involved in the immune response.  While Emapalumab is primarily used for refractory acute GVHD, it is also commonly used in another setting, which is the treatment of primary hemophagocytic lymphohistiocytosis (HLH). Primary HLH is a life-threatening condition characterized by an excessive and uncontrolled immune response leading to widespread inflammation and tissue damage.  Emapalumab is specifically approved by the FDA for the treatment of primary HLH in pediatric and adult patients who have refractory, recurrent, or progressive disease or who are intolerant to conventional HLH therapy. It works by blocking the activity of IFN-γ, which is involved in the hyperinflammatory response seen in primary HLH.  In both refractory acute GVHD and primary HLH, Emapalumab targets specific aspects of the immune response to modulate the underlying immune dysregulation and reduce inflammation, providing clinical benefit in these challenging conditions. |
| D8 | A commonly used drug during conditioning associated with lung injury in the context of allogeneic hematopoietic stem cell transplantation (HSCT) is busulfan. Busulfan is an alkylating agent frequently used in the conditioning regimen prior to HSCT to suppress the recipient's immune system and prepare the body for the transplantation of donor cells.  One of the known side effects of busulfan is pulmonary toxicity, which can manifest as lung injury or lung-related complications such as pneumonitis. Pulmonary toxicity associated with busulfan can cause symptoms such as cough, shortness of breath, and lung inflammation.  It is important to closely monitor patients receiving busulfan during conditioning for any signs of lung injury or pulmonary toxicity. If a patient develops a dry cough after receiving allogeneic HSCT and infectious causes have been excluded, it is crucial to evaluate for potential drug-related lung injury, including busulfan-induced pulmonary toxicity. In such cases, further assessment and management should be conducted in collaboration with the healthcare team, which may involve discontinuation or dose adjustment of busulfan and appropriate supportive care measures. | A commonly used drug during conditioning associated with lung injury in the context of allogeneic hematopoietic stem cell transplantation (HSCT) is busulfan. Busulfan is an alkylating agent frequently used in the conditioning regimen prior to HSCT to suppress the recipient's immune system and prepare the body for the transplantation of donor cells.  However, busulfan can potentially cause lung toxicity or lung injury as a side effect. Pulmonary complications associated with busulfan include pulmonary fibrosis, interstitial lung disease, and pneumonitis. These conditions can manifest with symptoms such as dry cough, shortness of breath, and lung inflammation.  If a patient develops a dry cough after receiving allogeneic HSCT and infectious causes have been ruled out, it is important to consider the possibility of busulfan-induced lung injury. Further evaluation and management should be undertaken in collaboration with the healthcare team, including pulmonary specialists if necessary. Treatment may involve discontinuation of busulfan, supportive care measures, and potentially the use of corticosteroids or other therapies to manage the lung injury. | A commonly used drug during conditioning associated with lung injury in the context of allogeneic hematopoietic stem cell transplantation (HSCT) is Bleomycin. Bleomycin is an antineoplastic medication that is used as part of the conditioning regimen in some HSCT protocols.  Bleomycin is known to have pulmonary toxicity as one of its significant side effects. Pulmonary toxicity can manifest as lung injury, interstitial pneumonitis, or fibrosis. Symptoms can include a dry cough, shortness of breath, and lung inflammation.  If a patient develops a dry cough after receiving allogeneic HSCT and infectious causes have been ruled out, it is important to consider the possibility of bleomycin-induced lung injury. Evaluation and management should be conducted in collaboration with the healthcare team, including pulmonary specialists if necessary. Treatment may involve discontinuation of bleomycin and supportive care measures to manage the lung injury. |
| D9 | Based on the clinical scenario described, the patient is likely receiving voriconazole as part of their treatment for the suspected fungal infection. Voriconazole is an antifungal medication commonly used for prophylaxis or treatment of fungal infections, including invasive aspergillosis, which is a common opportunistic infection in immunocompromised individuals following allogeneic hematopoietic stem cell transplantation.  One of the known side effects of voriconazole is electrolyte disturbances, including hypokalemia (low potassium levels). Hypokalemia can occur as a result of renal losses associated with the drug.  The standard dose of voriconazole can vary depending on the indication, patient characteristics, and specific institutional protocols. However, the usual recommended adult dose for voriconazole in the treatment of invasive fungal infections is an initial loading dose of 6 mg/kg given intravenously every 12 hours for two doses, followed by a maintenance dose of 4 mg/kg every 12 hours. It's important to note that the dosing regimen may be adjusted based on factors such as renal function, drug interactions, and patient tolerability.  It is crucial for the patient's healthcare team to monitor electrolyte levels regularly and adjust the dose or provide appropriate supplementation to manage the hypokalemia effectively while continuing the antifungal treatment. | Based on the clinical presentation and the development of hypokalemia, the patient is likely receiving an antifungal medication called amphotericin B for the treatment of a fungal infection. Amphotericin B is known to cause renal toxicity, including renal potassium wasting, which can lead to hypokalemia.  Amphotericin B is commonly used in the treatment of invasive fungal infections, including fungal pneumonia. The presence of galactomannan positivity in bronchoalveolar lavage suggests a potential fungal infection.  The standard dose of amphotericin B can vary depending on the formulation used. However, one of the commonly used formulations is liposomal amphotericin B (AmBisome). The typical standard dose for liposomal amphotericin B is around 3-5 mg/kg per day administered intravenously.  It's important to note that the dosage and duration of treatment may vary based on the specific infection, patient factors, and the healthcare provider's judgment. Close monitoring of renal function and electrolyte levels, including potassium, is essential during amphotericin B therapy to manage and prevent electrolyte abnormalities such as hypokalemia. | Based on the provided clinical scenario, the patient is likely receiving intravenous treatment for a suspected fungal infection following allogeneic hematopoietic stem cell transplantation (HSCT). The drug commonly used in this context is voriconazole, which is an antifungal medication.  Voriconazole is a broad-spectrum antifungal agent commonly used for the treatment of invasive fungal infections, including aspergillosis. It is effective against both Aspergillus species and other filamentous fungi. However, one of the known adverse effects of voriconazole is electrolyte abnormalities, including hypokalemia.  The standard dose of voriconazole for the treatment of invasive fungal infections in adults is typically an initial loading dose of 6 mg/kg every 12 hours for the first 24 hours, followed by a maintenance dose of 4 mg/kg every 12 hours. However, it's important to note that the dosing regimen may vary based on factors such as renal function, drug-drug interactions, and individual patient characteristics. Therefore, the specific dose should be determined by the treating healthcare team based on the patient's condition and specific factors.  It's worth mentioning that other antifungal medications, such as amphotericin B formulations or echinocandins, may also be used for the treatment of invasive fungal infections in HSCT patients. However, voriconazole is a commonly used agent in this context and is associated with the reported adverse effect of hypokalemia. Monitoring of electrolyte levels, including potassium, is important during voriconazole therapy to detect and manage any abnormalities that may arise. |
| D10 | If a patient's creatinine clearance is reduced to 50 ml/min, it indicates impaired kidney function. In such cases, the dose of the drug busulfan, which is commonly used in conditioning regimens, should be reduced.  Busulfan is primarily eliminated from the body through the kidneys, and impaired renal function can lead to decreased clearance of the drug. As a result, higher drug levels can accumulate in the body, potentially increasing the risk of toxicity.  To optimize the safety and efficacy of busulfan therapy in patients with reduced renal function, dose adjustments are typically made based on the estimated or measured creatinine clearance. Various dosing guidelines and algorithms are available to guide the adjustment of busulfan doses in these situations.  It is essential for the healthcare team, including the oncologist, hematologist, or transplant specialist, to assess the patient's renal function and determine the appropriate dose of busulfan to minimize the risk of toxicity while maintaining therapeutic effectiveness. | If a patient has a creatinine clearance of 50 ml/min, it indicates impaired kidney function. In such cases, the dose of the drug busulfan, which is commonly used in conditioning regimens, should be reduced.  Busulfan is primarily eliminated from the body through the kidneys, and impaired renal function can lead to decreased clearance of the drug. As a result, higher drug levels can accumulate in the body, potentially increasing the risk of toxicity.  To optimize the safety and efficacy of busulfan therapy in patients with reduced renal function, dose adjustments are typically made based on the estimated or measured creatinine clearance. Various dosing guidelines and algorithms are available to guide the adjustment of busulfan doses in these situations.  It is important for the healthcare team, including the oncologist, hematologist, or transplant specialist, to assess the patient's renal function and determine the appropriate dose of busulfan to minimize the risk of toxicity while maintaining therapeutic effectiveness. | If a patient has a creatinine clearance of 50 ml/min, it indicates impaired kidney function. In this case, the dose of the drug busulfan, which is commonly used in conditioning regimens, should be reduced.  Busulfan is primarily eliminated from the body through the kidneys, and impaired renal function can lead to decreased clearance of the drug. As a result, higher levels of busulfan can accumulate in the body, potentially increasing the risk of toxicity.  To optimize the safety and efficacy of busulfan therapy in patients with reduced renal function, dose adjustments are typically made based on the estimated or measured creatinine clearance. Various dosing guidelines and algorithms are available to guide the adjustment of busulfan doses in these situations.  It is important for the healthcare team, including the oncologist, hematologist, or transplant specialist, to assess the patient's renal function and determine the appropriate dose of busulfan to minimize the risk of toxicity while maintaining therapeutic effectiveness. |
| D11 | Before administering anti-thymocyte globulin (ATG) to a patient prior to allogeneic hematopoietic stem cell transplantation (HSCT), it is common to give certain medications to help prevent potential infusion-related reactions and manage potential allergic or hypersensitivity responses. These medications are typically administered immediately prior to ATG infusion. The specific medications given may vary based on institutional protocols and the patient's individual factors, but here are some commonly used drugs:  1. Antihistamines: Medications such as diphenhydramine (Benadryl) or chlorpheniramine are often administered to help prevent or reduce allergic reactions and hypersensitivity responses associated with ATG administration. These antihistamines block the effects of histamine and can help alleviate symptoms like itching, rash, or hives.  2. Corticosteroids: Glucocorticoids, such as methylprednisolone or prednisone, may be given to reduce the risk of infusion-related reactions, including cytokine release syndrome or anaphylaxis. Corticosteroids have potent anti-inflammatory and immunosuppressive properties and can help dampen immune responses triggered by ATG infusion.  3. Acetaminophen: Administration of acetaminophen (paracetamol) prior to ATG infusion is common to help minimize potential fever and discomfort associated with infusion-related reactions. It acts as an analgesic and antipyretic, reducing pain and fever symptoms.  These medications are typically given as part of a premedication regimen to mitigate potential adverse reactions associated with ATG infusion. The specific drugs and dosages will be determined by the healthcare team based on the patient's medical history, risk factors, and institutional guidelines. It's important to follow the instructions and recommendations provided by the healthcare professionals involved in the patient's care. | When administering anti-thymocyte globulin (ATG) prior to allogeneic hematopoietic stem cell transplantation (HSCT), certain medications are commonly given immediately before its administration to help minimize potential side effects. These medications are intended to prevent or reduce infusion-related reactions and allergic responses. The specific drugs typically used are:  1. Antipyretics: Medications such as acetaminophen (paracetamol) are often given to reduce the risk of fever and manage potential symptoms of fever, such as chills and body aches. This helps make the patient more comfortable during the infusion.  2. Antihistamines: Drugs like diphenhydramine (Benadryl) or chlorpheniramine may be administered to reduce the risk of allergic reactions, including itching, rash, or hives. These antihistamines help block the effects of histamine release, which can be triggered by ATG administration.  3. Corticosteroids: Glucocorticoids, such as methylprednisolone, may be given to help prevent or minimize infusion-related reactions. Corticosteroids possess anti-inflammatory and immunosuppressive properties, which can help reduce the risk of allergic responses and mitigate potential side effects of ATG.  The specific dosages and timing of these medications will be determined by the treating healthcare team based on the patient's condition, medical history, and institutional protocols. It's important to note that the use of these medications may vary among different transplant centers, and the decision to administer them should be made by the healthcare professionals overseeing the patient's care. | Before the administration of anti-thymocyte globulin (ATG) as part of the conditioning regimen for allogeneic hematopoietic stem cell transplantation (HSCT), certain drugs are commonly given to help mitigate potential infusion-related reactions and hypersensitivity responses. The specific medications given may vary based on the transplant center's protocols and individual patient factors, but here are some examples of drugs that are frequently used:  1. Antihistamines: Medications such as diphenhydramine (Benadryl) or cetirizine (Zyrtec) are often given prior to ATG infusion to help prevent or reduce allergic reactions. These antihistamines block the effects of histamine, which can be released during an allergic response.  2. Corticosteroids: Glucocorticoids, such as methylprednisolone or hydrocortisone, are commonly administered before ATG to reduce the risk of infusion-related reactions. Corticosteroids have potent anti-inflammatory and immunosuppressive properties, helping to minimize the immune response that could lead to hypersensitivity reactions.  3. Acetaminophen: Acetaminophen (paracetamol) may be given prior to ATG infusion to alleviate potential fever and reduce discomfort during the infusion.  These medications are typically administered shortly before the ATG infusion to help minimize the risk and severity of infusion-related reactions, including fever, chills, rash, and other allergic or hypersensitivity symptoms. The specific timing and dosages of these medications will be determined by the healthcare team based on the individual patient's needs and any existing medical conditions or contraindications. It's important for the patient to follow the instructions of their healthcare team regarding the timing and administration of these medications. |
| D12 | The half-life of fludarabine, a chemotherapy medication commonly used in hematopoietic stem cell transplantation (HSCT) conditioning regimens, is approximately 20 hours. This means that it takes approximately 20 hours for half of the administered dose of fludarabine to be eliminated from the body.  It's worth noting that the half-life of a drug can vary slightly among individuals due to factors such as age, liver function, kidney function, and other medications being taken concurrently. Therefore, the specific half-life of fludarabine in an individual patient may differ slightly from the average value mentioned above. For accurate information regarding a patient's medication, it's best to consult with a healthcare professional or refer to the prescribing information for fludarabine. | The half-life of fludarabine, a chemotherapy drug commonly used in hematopoietic stem cell transplantation (HSCT) conditioning regimens, is approximately 20 hours. This means that it takes around 20 hours for the concentration of fludarabine in the body to decrease by half.  It's important to note that individual variations in drug metabolism and other factors can influence the half-life of fludarabine in specific patients. Therefore, it is always best to consult with a healthcare professional or refer to the specific drug information provided by the manufacturer or prescribing guidelines for accurate and up-to-date information on fludarabine's pharmacokinetics and half-life. | The half-life of a drug refers to the time it takes for the concentration of the drug in the body to decrease by half. Fludarabine is an antimetabolite chemotherapy drug commonly used in the treatment of hematological malignancies, such as chronic lymphocytic leukemia (CLL) and non-Hodgkin's lymphoma.  The half-life of fludarabine is approximately 20 hours. This means that it takes approximately 20 hours for the concentration of fludarabine in the body to decrease by half. However, it's important to note that individual variations in drug metabolism and other factors can affect the actual half-life observed in patients.  It's always advisable to consult with a healthcare professional or refer to the prescribing information for precise and up-to-date information on the pharmacokinetics of fludarabine or any other medication. |
| D13 | A patient with acute lymphoblastic leukemia (ALL) who is being treated with inotuzumab and undergoing haploidentical hematopoietic stem cell transplantation (HSCT) is at risk of hepatic complications, specifically sinusoidal obstruction syndrome (SOS), also known as veno-occlusive disease (VOD).  Inotuzumab is an antibody-drug conjugate used in the treatment of relapsed or refractory ALL. It is associated with an increased risk of SOS/VOD, particularly when used before HSCT.  To minimize the risk of SOS/VOD in this patient, several strategies can be employed:  1. Risk assessment: Evaluate the patient's baseline risk factors for developing SOS/VOD, such as previous liver disease, history of high-dose chemotherapy, pre-existing liver dysfunction, and certain genetic or disease-related factors. This can help identify patients who may be at higher risk and require closer monitoring.  2. Dose optimization: Adjust the dose of inotuzumab based on the patient's liver function and individual risk factors to minimize the potential hepatotoxicity. This may involve dose reduction or delaying treatment if there are concerns about liver function.  3. Monitoring and surveillance: Regularly monitor liver function tests, including liver enzymes such as AST (aspartate aminotransferase), ALT (alanine aminotransferase), and bilirubin levels. Additionally, consider imaging studies such as ultrasound or Doppler to assess liver blood flow and evaluate for signs of SOS/VOD.  4. Supportive care measures: Implement supportive care measures to help manage and prevent SOS/VOD. This may include maintaining fluid balance, diuretics to manage fluid retention, use of medications to support liver function (such as ursodiol), and other supportive therapies as guided by the healthcare team.  5. Early intervention: If signs or symptoms of SOS/VOD, such as hepatomegaly, jaundice, weight gain, or fluid retention, develop, prompt intervention is crucial. Treatment options for SOS/VOD may include defibrotide, which is approved for the treatment of severe hepatic SOS/VOD, or other supportive measures to manage the complications.  The management of hepatic complications in patients undergoing haploidentical HSCT requires close collaboration between the oncology, transplantation, and supportive care teams. The specific approach and interventions may vary based on individual patient characteristics, institutional protocols, and expert guidance. | A patient with acute lymphoblastic leukemia (ALL) treated with inotuzumab and undergoing haploidentical hematopoietic stem cell transplantation (HSCT) is at risk for hepatic complications, specifically sinusoidal obstruction syndrome (SOS), also known as veno-occlusive disease (VOD).  Inotuzumab, an antibody-drug conjugate used in the treatment of relapsed or refractory ALL, has been associated with an increased risk of SOS/VOD, particularly when used prior to HSCT.  To minimize the risk of SOS/VOD in this patient, several strategies can be considered:  1. Risk assessment: Evaluate the patient's baseline risk factors for developing SOS/VOD, including previous liver disease, history of high-dose chemotherapy, pre-existing liver dysfunction, and genetic or disease-related factors. Identifying patients at higher risk can help guide preventative measures and close monitoring.  2. Dose optimization: Adjust the dose of inotuzumab based on the patient's liver function and individual risk factors. Dose modifications or treatment delays may be necessary to reduce the potential hepatotoxicity.  3. Monitoring and surveillance: Regularly monitor liver function tests, including liver enzymes (such as AST, ALT, and bilirubin), throughout the treatment period. Imaging studies, such as ultrasound or Doppler, may also be employed to assess liver blood flow and detect early signs of SOS/VOD.  4. Supportive care: Implement supportive care measures to manage and prevent SOS/VOD. This may include maintaining fluid balance, diuretics to manage fluid retention, and the use of medications that support liver function, such as ursodiol. Other supportive therapies can be considered based on the patient's specific needs and clinical presentation.  5. Early intervention: If signs or symptoms of SOS/VOD, such as hepatomegaly, jaundice, weight gain, or fluid retention, occur, early intervention is critical. Treatment options for SOS/VOD may include defibrotide, which is approved for the treatment of severe hepatic SOS/VOD, or other supportive measures aimed at managing the complications.  The management of hepatic complications in patients undergoing haploidentical HSCT requires a multidisciplinary approach involving close collaboration between the oncology, transplantation, and supportive care teams. The specific preventive measures and interventions may vary based on individual patient characteristics, institutional protocols, and expert guidance. | A patient with acute lymphoblastic leukemia (ALL) who is being treated with inotuzumab and undergoing haploidentical hematopoietic stem cell transplantation (HSCT) is at risk for hepatic complications, particularly sinusoidal obstruction syndrome (SOS), also known as veno-occlusive disease (VOD).  Inotuzumab, an antibody-drug conjugate used in the treatment of relapsed or refractory ALL, has been associated with an increased risk of hepatic complications, including SOS/VOD.  To minimize the risk of hepatic complications in this patient, several strategies can be implemented:  1. Risk assessment: Evaluate the patient's baseline risk factors for developing SOS/VOD, such as previous liver disease, history of high-dose chemotherapy, pre-existing liver dysfunction, and specific genetic or disease-related factors. Identifying patients at higher risk can help guide preventative measures and close monitoring.  2. Dose optimization: Adjust the dose of inotuzumab based on the patient's liver function and individual risk factors. Dose modifications or treatment delays may be necessary to minimize the potential hepatotoxicity associated with inotuzumab.  3. Preemptive therapy: Consider using prophylactic medications to prevent or reduce the risk of SOS/VOD. The use of medications such as ursodiol or defibrotide has been explored in the prevention of hepatic complications in HSCT patients, including those receiving inotuzumab.  4. Close monitoring: Regularly monitor liver function tests, including liver enzymes (such as AST, ALT, and bilirubin), throughout the treatment period. Imaging studies, such as ultrasound or Doppler, may also be employed to assess liver blood flow and detect early signs of hepatic complications.  5. Supportive care: Implement supportive care measures aimed at maintaining liver health and function. This may include maintaining fluid balance, managing fluid retention with diuretics, and providing adequate nutrition and hydration.  6. Early intervention: If signs or symptoms of hepatic complications, such as hepatomegaly, jaundice, abdominal pain, or weight gain, develop, early intervention is crucial. Prompt recognition and treatment of SOS/VOD can help improve outcomes. Treatment options may include defibrotide, which is approved for the treatment of severe hepatic SOS/VOD, or other supportive measures tailored to the patient's condition.  It is important to closely collaborate with the healthcare team, including oncologists, hematologists, transplant specialists, and supportive care providers, to implement an individualized approach and optimize the patient's management. |
| D14 | In a patient who received rituximab prior to allogeneic hematopoietic stem cell transplantation (HSCT), which can impair B cell reconstitution and increase the risk of infection, there are several additional measures that can be taken to reduce the risk of infection:  1. Immunoglobulin Replacement: Administering intravenous immunoglobulin (IVIG) or subcutaneous immunoglobulin (SCIG) can help provide temporary passive immunity and protect against certain infections. This can be especially beneficial during the early period of B cell recovery.  2. Vaccinations: Administering appropriate vaccinations before or after HSCT can help provide protection against vaccine-preventable infections. Depending on the patient's specific circumstances, vaccinations such as pneumococcal, influenza, hepatitis B, and others may be recommended. It is important to follow the vaccination guidelines specific to HSCT recipients, as timing and administration may vary.  3. Viral Prophylaxis: Depending on the patient's viral serostatus, antiviral medications can be given as prophylaxis against specific viral infections, such as cytomegalovirus (CMV) or Epstein-Barr virus (EBV). Antiviral prophylaxis is typically tailored to the patient's individual risk factors and institutional protocols.  4. Environmental Precautions: Implementing appropriate infection control measures, such as practicing good hand hygiene, using personal protective equipment, and maintaining a clean environment, can help minimize the risk of exposure to infectious agents.  5. Neutropenic Precautions: Adhering to neutropenic precautions, such as avoiding crowded places, close contact with individuals who are sick, and raw or undercooked food, can reduce the risk of infections during the period of B cell reconstitution.  It is crucial to work closely with the healthcare team, including infectious disease specialists, transplant physicians, and supportive care providers, to assess the patient's individual risk factors, immune status, and specific institutional protocols. The management of infection risk in this context requires a personalized approach tailored to the patient's unique circumstances. | In a patient who received rituximab prior to allogeneic hematopoietic stem cell transplantation (HSCT), which can impair B cell reconstitution and increase the risk of infection, there are several additional measures that can be taken to reduce the risk of infection:  1. Immunoglobulin Replacement: Administering intravenous immunoglobulin (IVIG) or subcutaneous immunoglobulin (SCIG) can help provide temporary passive immunity and protect against certain infections. This can be especially beneficial during the early period of B cell recovery. Immunoglobulin replacement therapy can help compensate for the impaired antibody production and provide additional immune support.  2. Vaccinations: Administering appropriate vaccinations before or after HSCT can help provide protection against vaccine-preventable infections. Depending on the patient's specific circumstances, vaccinations such as pneumococcal, influenza, hepatitis B, and others may be recommended. It is important to follow the vaccination guidelines specific to HSCT recipients, as timing and administration may vary.  3. Viral Prophylaxis: Depending on the patient's viral serostatus, antiviral medications can be given as prophylaxis against specific viral infections, such as cytomegalovirus (CMV) or Epstein-Barr virus (EBV). Antiviral prophylaxis is typically tailored to the patient's individual risk factors and institutional protocols.  4. Environmental Precautions: Implementing appropriate infection control measures, such as practicing good hand hygiene, using personal protective equipment, and maintaining a clean environment, can help minimize the risk of exposure to infectious agents.  5. Neutropenic Precautions: Adhering to neutropenic precautions, such as avoiding crowded places, close contact with individuals who are sick, and raw or undercooked food, can reduce the risk of infections during the period of B cell reconstitution.  It is crucial to work closely with the healthcare team, including infectious disease specialists, transplant physicians, and supportive care providers, to assess the patient's individual risk factors, immune status, and specific institutional protocols. The management of infection risk in this context requires a personalized approach tailored to the patient's unique circumstances. | In a patient who received rituximab prior to allogeneic hematopoietic stem cell transplantation (HSCT), which can impair B cell reconstitution and increase the risk of infection, there are several additional measures that can be taken to reduce the risk of infection:  1. Immunoglobulin Replacement: Administering intravenous immunoglobulin (IVIG) or subcutaneous immunoglobulin (SCIG) can help provide temporary passive immunity and protect against certain infections. Immunoglobulin replacement therapy can compensate for the impaired antibody production and provide additional immune support.  2. Vaccinations: Administering appropriate vaccinations before or after HSCT can help provide protection against vaccine-preventable infections. Vaccinations should be given based on the patient's immunization history and specific guidelines for HSCT recipients. Common vaccines may include pneumococcal, influenza, hepatitis B, and others. Timing and administration should be coordinated with the healthcare team.  3. Antiviral Prophylaxis: Depending on the patient's serostatus and the risk of viral infections, antiviral medications may be administered as prophylaxis. This is particularly relevant for viruses such as cytomegalovirus (CMV) or Epstein-Barr virus (EBV), which can cause significant complications in immunocompromised individuals.  4. Environmental Precautions: Implementing stringent infection control measures is essential to minimize the risk of infections. This includes practicing good hand hygiene, using personal protective equipment, limiting exposure to potentially infectious individuals, and maintaining a clean and hygienic environment.  5. Neutropenic Precautions: Adhering to neutropenic precautions is crucial during the period of impaired B cell reconstitution. This involves avoiding crowded places, close contact with individuals who are sick, and consuming cooked or well-prepared foods to minimize the risk of infections.  6. Prophylactic Antibiotics: In certain situations, prophylactic antibiotics may be prescribed to help prevent specific bacterial infections during the early post-transplant period. The choice and duration of prophylactic antibiotics depend on various factors, including institutional protocols, patient risk factors, and local epidemiology.  It is important to consult with the healthcare team, including the transplant specialist, infectious disease specialist, and supportive care providers, to determine the most appropriate strategies for reducing the risk of infection in this specific patient. Individualized approaches based on patient characteristics, transplant protocols, and institutional guidelines will help optimize infection prevention and management. |
| D15 | The development of periorbital swelling in a patient with newly diagnosed Philadelphia-positive acute lymphoblastic leukemia (ALL) is commonly associated with the use of a tyrosine kinase inhibitor (TKI) called dasatinib.  Dasatinib is a targeted therapy that is often used in the treatment of Philadelphia chromosome-positive ALL, a subtype of ALL characterized by the presence of the Philadelphia chromosome. Dasatinib works by inhibiting the abnormal BCR-ABL1 tyrosine kinase, which is responsible for the uncontrolled growth and survival of leukemic cells.  Periorbital swelling is a known side effect of dasatinib treatment. It can result from fluid retention caused by the drug's effects on vascular permeability and fluid balance. Other potential side effects of dasatinib include fluid retention, edema, pleural effusions, and cardiovascular effects.  If a patient with newly diagnosed Philadelphia-positive ALL develops periorbital swelling while receiving treatment, it is important to notify the healthcare team promptly. They will evaluate the patient's symptoms, assess for any other signs of drug-related side effects, and determine the appropriate management, which may involve dose adjustments or supportive measures to address the swelling and related symptoms. | In a patient with newly diagnosed Philadelphia-positive acute lymphoblastic leukemia (ALL) who develops periorbital swelling, the likely drug being received is a tyrosine kinase inhibitor (TKI) called dasatinib.  Dasatinib is a targeted therapy commonly used in the treatment of Philadelphia chromosome-positive ALL, which is characterized by the presence of the Philadelphia chromosome. Dasatinib works by inhibiting the abnormal BCR-ABL1 tyrosine kinase, which is responsible for the uncontrolled growth and survival of leukemic cells.  Periorbital swelling is a known side effect of dasatinib treatment. It can result from fluid retention caused by the drug's effects on vascular permeability and fluid balance. Other potential side effects of dasatinib include fluid retention, edema, pleural effusions, and cardiovascular effects.  If a patient with newly diagnosed Philadelphia-positive ALL develops periorbital swelling while receiving dasatinib, it is important to inform the healthcare team promptly. They will evaluate the patient's symptoms, assess for any other signs of drug-related side effects, and determine the appropriate management, which may involve dose adjustments or supportive measures to address the swelling and related symptoms. | In a patient with newly diagnosed Philadelphia-positive acute lymphoblastic leukemia (ALL) who develops periorbital swelling, the likely drug being received is a tyrosine kinase inhibitor (TKI) called ponatinib.  Ponatinib is a targeted therapy used in the treatment of Philadelphia chromosome-positive ALL, which is characterized by the presence of the Philadelphia chromosome. Ponatinib works by inhibiting the abnormal BCR-ABL1 tyrosine kinase, which is responsible for the uncontrolled growth and survival of leukemic cells.  Periorbital swelling is a known side effect of ponatinib treatment. It can occur as a result of fluid retention and may be associated with other symptoms such as peripheral edema or pleural effusions. Regular monitoring of fluid balance and appropriate management strategies can help mitigate these side effects.  If a patient with newly diagnosed Philadelphia-positive ALL develops periorbital swelling while receiving ponatinib, it is important to inform the healthcare team promptly. They will assess the patient's symptoms, evaluate for any other signs of drug-related side effects, and determine the appropriate management approach, which may include dose adjustments, supportive measures, or alternative treatment options. |
| D16 | For antiemetic purposes in patients undergoing a conditioning regimen with busulfan and fludarabine, a combination of antiemetic medications is typically used to provide optimal nausea and vomiting control. The specific antiemetic regimen may vary depending on the patient's individual risk factors, medical history, and institutional guidelines. However, commonly prescribed medications include:  1. 5-HT3 receptor antagonists: Drugs such as ondansetron, granisetron, or palonosetron are frequently used as the first-line agents for preventing chemotherapy-induced nausea and vomiting. These medications work by blocking serotonin receptors in the central nervous system.  2. NK1 receptor antagonists: Medications like aprepitant or fosaprepitant are often used in combination with 5-HT3 receptor antagonists. NK1 receptor antagonists provide further antiemetic control by blocking substance P, a neurotransmitter involved in the emetic pathway.  3. Corticosteroids: Dexamethasone or methylprednisolone may be added to the antiemetic regimen for their anti-inflammatory and antiemetic properties. They can enhance the effectiveness of 5-HT3 receptor antagonists and NK1 receptor antagonists.  4. Benzodiazepines: Medications such as lorazepam or alprazolam may be prescribed to manage anxiety and reduce anticipatory nausea and vomiting.  The selection and dosing of antiemetic medications should be tailored to the patient's specific needs and guided by the healthcare team's recommendations. It is important to discuss antiemetic options and potential side effects with the healthcare provider to develop an appropriate and effective antiemetic strategy for the patient. | For antiemetic purposes in patients undergoing a conditioning regimen with busulfan and fludarabine, a combination of antiemetic medications is typically used to provide optimal nausea and vomiting control. The specific antiemetic regimen may vary depending on the patient's individual risk factors, medical history, and institutional guidelines. However, commonly prescribed medications include:  1. 5-HT3 receptor antagonists: Drugs such as ondansetron, granisetron, or palonosetron are frequently used as the first-line agents for preventing chemotherapy-induced nausea and vomiting. These medications work by blocking serotonin receptors in the central nervous system.  2. NK1 receptor antagonists: Medications like aprepitant or fosaprepitant are often used in combination with 5-HT3 receptor antagonists. NK1 receptor antagonists provide further antiemetic control by blocking substance P, a neurotransmitter involved in the emetic pathway.  3. Corticosteroids: Dexamethasone or methylprednisolone may be added to the antiemetic regimen for their anti-inflammatory and antiemetic properties. They can enhance the effectiveness of 5-HT3 receptor antagonists and NK1 receptor antagonists.  4. Benzodiazepines: Medications such as lorazepam or alprazolam may be prescribed to manage anxiety and reduce anticipatory nausea and vomiting.  The selection and dosing of antiemetic medications should be tailored to the patient's specific needs and guided by the healthcare team's recommendations. It is important to discuss antiemetic options and potential side effects with the healthcare provider to develop an appropriate and effective antiemetic strategy for the patient. | For antiemetic purposes in patients undergoing a conditioning regimen with busulfan and fludarabine, a combination of antiemetic medications is typically used to provide optimal nausea and vomiting control. The specific antiemetic regimen may vary depending on the patient's individual risk factors, medical history, and institutional guidelines. However, commonly prescribed medications include:  1. 5-HT3 receptor antagonists: Drugs such as ondansetron, granisetron, or palonosetron are frequently used as the first-line agents for preventing chemotherapy-induced nausea and vomiting. These medications work by blocking serotonin receptors in the central nervous system.  2. NK1 receptor antagonists: Medications like aprepitant or fosaprepitant are often used in combination with 5-HT3 receptor antagonists. NK1 receptor antagonists provide further antiemetic control by blocking substance P, a neurotransmitter involved in the emetic pathway.  3. Corticosteroids: Dexamethasone or methylprednisolone may be added to the antiemetic regimen for their anti-inflammatory and antiemetic properties. They can enhance the effectiveness of 5-HT3 receptor antagonists and NK1 receptor antagonists.  4. Benzodiazepines: Medications such as lorazepam or alprazolam may be prescribed to manage anxiety and reduce anticipatory nausea and vomiting.  The selection and dosing of antiemetic medications should be tailored to the patient's specific needs and guided by the healthcare team's recommendations. It is important to discuss antiemetic options and potential side effects with the healthcare provider to develop an appropriate and effective antiemetic strategy for the patient. |
| D17 | Based on the symptoms described, the drug that was likely discontinued and causing vivid dreams and visual hallucinations in the patient undergoing allogeneic HSCT is mefloquine.  Mefloquine is an antimalarial medication that is sometimes used for prophylaxis against malaria in certain regions. However, it can have neurological side effects, including vivid dreams, nightmares, and visual hallucinations. These side effects are more commonly associated with higher doses of mefloquine.  The exact mechanism of action of mefloquine is not fully understood. It is believed to interfere with the functioning of the parasite's digestive vacuole, preventing the metabolism of hemoglobin and leading to the accumulation of toxic heme byproducts within the parasite. This disrupts the parasite's survival and reproduction.  In the context of allogeneic HSCT, patients are often placed on antimicrobial prophylaxis regimens to prevent infections. Mefloquine may be prescribed as part of the prophylactic regimen in cases where the patient is at risk of contracting malaria. However, due to its potential neurological side effects, including vivid dreams and visual hallucinations, it is sometimes discontinued if these symptoms occur.  It's important to note that the specific antimicrobial prophylaxis regimen for HSCT patients may vary based on various factors, including the patient's medical history, the transplant center's protocols, and the geographic location of the transplant. The decision to discontinue mefloquine or any other medication should be made by the healthcare team based on the patient's individual situation and the risk-benefit assessment. | The drug that is most likely associated with the vivid dreams and visual hallucinations reported by the patient undergoing allogeneic HSCT is mirtazapine. Mirtazapine is an oral drug that is commonly used for antimicrobial prophylaxis in HSCT patients to prevent nausea and vomiting, particularly associated with chemotherapy-induced emesis. However, it is important to note that mirtazapine is primarily an antidepressant medication and is not typically used as an antimicrobial agent.  Mirtazapine belongs to a class of medications known as noradrenergic and specific serotonergic antidepressants (NaSSAs). It works by acting on various neurotransmitter systems in the brain, including increasing the release of norepinephrine and serotonin. The exact mechanism of action of mirtazapine in causing vivid dreams and hallucinations is not fully understood, but it is believed to be related to its effects on serotonin receptors.  One of the known side effects of mirtazapine is the potential to cause vivid dreams, nightmares, and other sleep disturbances. Visual hallucinations have also been reported in some cases. These effects are more likely to occur at higher doses of mirtazapine.  If the patient experienced vivid dreams and visual hallucinations, discontinuing mirtazapine would be a reasonable step to alleviate these symptoms. However, it's important for the patient to consult with their healthcare team before making any changes to their medication regimen. The healthcare team can provide guidance on managing the side effects and explore alternative medications, if necessary. | Based on the provided information, the drug that is likely associated with the patient's vivid dreams and visual hallucinations and subsequently discontinued is probably voriconazole. Voriconazole is an antifungal medication commonly used for prophylaxis and treatment of invasive fungal infections in patients undergoing allogeneic HSCT.  The mechanism of action of voriconazole involves inhibition of the fungal enzyme lanosterol 14-alpha-demethylase, which is responsible for the conversion of lanosterol to ergosterol. Ergosterol is an essential component of the fungal cell membrane. By inhibiting this enzyme, voriconazole disrupts fungal cell membrane synthesis and function, leading to the inhibition of fungal growth.  Voriconazole is known to have central nervous system (CNS) effects as a potential side effect. Neurological symptoms, such as vivid dreams, visual hallucinations, and altered mental status, have been reported in some patients receiving voriconazole. These CNS side effects are generally reversible upon discontinuation of the drug.  It's important to note that other medications used for antimicrobial prophylaxis, such as sulfamethoxazole/trimethoprim (Bactrim) or fluoroquinolones, may also have central nervous system effects, but vivid dreams and visual hallucinations are more commonly associated with voriconazole.  If a patient experiences CNS side effects while receiving voriconazole, the healthcare team may choose to discontinue the medication and consider alternative antifungal agents or prophylaxis strategies. Close monitoring and communication with the healthcare team are essential in such situations to ensure appropriate management of side effects and continued effective antimicrobial prophylaxis. |
| D18 | In the scenario you described, where a patient with chronic graft-versus-host disease (GVHD) is scheduled for dental surgery and is receiving a second-line treatment commonly used in chronic lymphocytic leukemia (CLL), it's important to consider the specific drug being used and its potential impact on dental surgery.  To make an informed decision about whether to discontinue the drug prior to dental surgery, it's crucial to consult with the patient's healthcare team, including their oncologist and dentist. They will have a better understanding of the patient's overall medical condition, the specific drug being used, and its potential impact on wound healing and the risk of complications during dental procedures.  Certain chemotherapy drugs or immunosuppressants used in CLL or GVHD treatment can affect the immune system and wound healing process. Depending on the drug and the patient's individual circumstances, it may be necessary to discontinue the drug temporarily before the dental surgery to minimize the risk of complications.  The decision to discontinue the drug and the duration of discontinuation should be made in consultation with the healthcare team. They will consider factors such as the specific drug being used, the patient's response to treatment, the severity of GVHD, the urgency of the dental surgery, and any alternative options available to manage the patient's condition during the discontinuation period.  It's crucial to have open communication between the patient's oncologist and dentist to ensure a coordinated approach and provide optimal care for the patient's overall well-being. | In the scenario you presented, where a patient with chronic graft-versus-host disease (GVHD) is started on a drug commonly used in chronic lymphocytic leukemia (CLL) and is scheduled for dental surgery, it is crucial to consider the potential impact of the medication on the surgery and the patient's overall health.    Since you haven't specified the specific drug being used, I can provide general guidance. It is important to consult with the patient's healthcare team, including the oncologist and dentist, to determine the best course of action based on the specific drug and the patient's individual circumstances. In some cases, the drug may need to be temporarily discontinued before the dental surgery to reduce the risk of bleeding or other complications.  Certain CLL treatments, such as chemotherapy agents or targeted therapies, can increase the risk of bleeding or affect the immune system, which may impact the patient's ability to heal properly after dental surgery. Discontinuation of the drug, if deemed necessary, is usually a temporary measure taken before and after the dental procedure to minimize these risks.  Ultimately, the decision to discontinue the drug and the timing of discontinuation should be made in collaboration between the patient's oncologist, dentist, and other members of the healthcare team. They will consider the patient's specific medical condition, the type of medication being used, and the risks and benefits associated with temporarily stopping the drug in relation to the planned dental surgery. | When a patient with chronic graft-versus-host disease (cGVHD) requires second-line treatment and a drug commonly used in chronic lymphocytic leukemia (CLL) is started, the decision to discontinue the drug prior to dental surgery should be made in consultation with the patient's healthcare team. They will consider various factors such as the specific drug being used, the patient's overall health, the severity of the cGVHD, and the nature of the dental procedure.  Certain medications used in CLL, such as immunosuppressive agents or targeted therapies like ibrutinib, can increase the risk of bleeding or impair wound healing. In such cases, discontinuing the drug prior to dental surgery may be necessary to minimize these risks. However, it's important to note that stopping a medication also carries potential risks, including disease progression or flares of cGVHD.  The healthcare team, which may include the patient's hematologist/oncologist, dentist, and other specialists, will evaluate the individual case and provide specific guidance on the discontinuation of the drug before the dental procedure. They will consider the risks and benefits, timing of the procedure, potential alternatives, and develop an appropriate management plan to ensure the patient's safety and optimal outcomes. |
| **Section 2 – HSCT Platforms** | | | |
| P1 | GVHD prophylaxis, or graft-versus-host disease prophylaxis, refers to the preventive measures and treatment strategies employed to minimize the risk and severity of graft-versus-host disease (GVHD) in patients undergoing allogeneic hematopoietic stem cell transplantation (HSCT).  GVHD occurs when the transplanted donor cells (graft) recognize the recipient's tissues as foreign and mount an immune response against them. It is a major complication of allogeneic HSCT and can affect various organs, including the skin, liver, and gastrointestinal tract.  GVHD prophylaxis aims to modulate or suppress the donor's immune response to reduce the risk of GVHD while still allowing for the graft-versus-leukemia (GVL) effect, where donor cells attack residual cancer cells. Commonly used GVHD prophylaxis strategies include:  1. Calcineurin Inhibitors: Drugs such as cyclosporine and tacrolimus are commonly used to suppress the immune response by inhibiting the activity of calcineurin, a protein involved in T-cell activation. These medications are typically initiated before transplant and continued for several months post-transplant.  2. Methotrexate: Methotrexate is an immunosuppressive medication that is often used in combination with a calcineurin inhibitor as part of GVHD prophylaxis. It is usually given as a short course of high-dose therapy shortly after transplantation.  3. Antithymocyte Globulin (ATG): ATG is a polyclonal antibody preparation that is used to deplete T cells and modulate the immune system. It is often administered prior to transplantation to suppress the recipient's immune system and reduce the risk of GVHD.  4. Post-Transplant Cyclophosphamide (PTCy): PTCy is a newer approach to GVHD prophylaxis. It involves administering high-dose cyclophosphamide shortly after transplantation to selectively eliminate activated donor T cells responsible for GVHD.  5. Other Immunosuppressive Agents: Additional medications such as mycophenolate mofetil, sirolimus, and prednisone may be used as part of the GVHD prophylaxis regimen depending on the individual patient's risk factors and transplantation protocols.  The specific GVHD prophylaxis regimen used may vary based on the patient's underlying disease, transplant type (related or unrelated donor), conditioning regimen, and institutional guidelines. The goal is to strike a balance between preventing GVHD and maintaining graft function and GVL effect. Close monitoring and regular assessment of the patient's response to prophylaxis are crucial for optimizing outcomes and minimizing complications. | GVHD prophylaxis, or graft-versus-host disease prophylaxis, refers to the strategies and treatments used to reduce the risk and severity of graft-versus-host disease (GVHD) in patients undergoing allogeneic hematopoietic stem cell transplantation (HSCT). GVHD occurs when the donor's immune cells recognize the recipient's tissues as foreign and mount an immune response against them.  The goal of GVHD prophylaxis is to balance the suppression of the donor immune response to prevent GVHD while still allowing for the graft-versus-leukemia (GVL) effect, where donor immune cells attack residual cancer cells. The specific prophylaxis regimen may vary depending on factors such as the patient's underlying condition, transplant type, and institutional protocols. Some common approaches to GVHD prophylaxis include:  1. Calcineurin Inhibitors: Drugs such as cyclosporine and tacrolimus are commonly used as the backbone of GVHD prophylaxis. They work by suppressing T-cell activation and cytokine release, reducing the immune response against host tissues.  2. Methotrexate: Methotrexate is an immunosuppressive medication that is often used in combination with calcineurin inhibitors. It is typically given as a short course of high-dose therapy shortly after transplantation.  3. Antithymocyte Globulin (ATG): ATG is a polyclonal antibody preparation that is used to deplete T cells and modulate the immune system. It is often administered before transplantation to reduce the risk of GVHD.  4. Post-Transplant Cyclophosphamide (PTCy): PTCy is an emerging approach in GVHD prophylaxis. It involves the administration of high-dose cyclophosphamide shortly after transplantation to selectively eliminate activated donor T cells responsible for GVHD.  5. Other Immunosuppressive Agents: Additional medications such as mycophenolate mofetil, sirolimus, or prednisone may be used in combination or as alternatives to the above drugs based on individual patient factors and transplant protocols.  The selection and combination of prophylaxis agents depend on the patient's risk factors, transplant characteristics, and the healthcare team's expertise and institutional guidelines. The goal is to find a balance between preventing GVHD and preserving the beneficial effects of the transplant. Close monitoring and regular follow-up are crucial to assess the patient's response to prophylaxis and adjust the regimen as needed. | GVHD prophylaxis, or graft-versus-host disease prophylaxis, refers to the preventive measures and strategies used to minimize the risk of graft-versus-host disease (GVHD) in patients undergoing allogeneic hematopoietic stem cell transplantation (HSCT).  GVHD occurs when the transplanted donor cells recognize the recipient's tissues as foreign and attack them, leading to a range of inflammatory and immune-mediated complications. GVHD prophylaxis aims to suppress the immune response of the donor cells against the recipient's tissues while maintaining the graft-versus-leukemia (GVL) effect, where donor immune cells target and eliminate cancer cells.  Common approaches to GVHD prophylaxis include:  1. Calcineurin Inhibitors: Medications such as cyclosporine and tacrolimus are commonly used to suppress the immune response and reduce the risk of GVHD. They inhibit the activation of T cells by blocking the calcineurin pathway.  2. Methotrexate: Methotrexate is an immunosuppressive drug that is often given in combination with calcineurin inhibitors as part of GVHD prophylaxis. It helps prevent the proliferation and activation of immune cells.  3. Antithymocyte Globulin (ATG): ATG is a polyclonal antibody preparation that is used to suppress the immune system and reduce the risk of GVHD. It targets and depletes certain immune cells, including T cells.  4. Post-Transplant Cyclophosphamide (PTCy): PTCy is a newer approach to GVHD prophylaxis. It involves the administration of high-dose cyclophosphamide after HSCT to selectively target and eliminate activated T cells that may contribute to GVHD.  5. Other Immunosuppressive Agents: Additional medications, such as mycophenolate mofetil, sirolimus, or prednisone, may be used alone or in combination with the above therapies to further suppress the immune response and reduce GVHD risk.  The specific choice and combination of GVHD prophylaxis medications may depend on various factors, including the patient's risk factors, donor source, conditioning regimen, and transplant center protocols. The goal is to find the most effective regimen that minimizes GVHD while maintaining engraftment and promoting successful outcomes. Close monitoring and regular follow-up are important to assess the patient's response to prophylaxis and adjust the regimen if necessary. |
| P2 | Treosulfan is an alkylating agent that is used in the field of oncology for conditioning regimens prior to hematopoietic stem cell transplantation (HSCT). It is a prodrug that is converted into an active form called monoepoxide treosulfan.  Treosulfan is used as a myeloablative or reduced-intensity conditioning agent in preparation for HSCT in patients with various hematological malignancies, such as acute myeloid leukemia (AML), acute lymphoblastic leukemia (ALL), myelodysplastic syndrome (MDS), and other high-risk or advanced hematologic cancers.  The main mechanism of action of treosulfan is its ability to crosslink DNA strands, leading to the inhibition of DNA synthesis and causing cell death. It has a broad spectrum of activity against both dividing and non-dividing cells.  Compared to other alkylating agents, treosulfan is considered to have a more favorable toxicity profile and lower risk of organ toxicity, which makes it suitable for conditioning regimens in certain patient populations, including those who may be more susceptible to complications from traditional conditioning agents.  The specific use and dosing of treosulfan may vary depending on the patient's condition, transplant protocol, and the healthcare team's recommendations. It is important to consult with an oncologist or hematologist for detailed information about treosulfan and its specific application in the context of individual patients. | Treosulfan is an alkylating agent that is used in the field of oncology for conditioning regimens prior to hematopoietic stem cell transplantation (HSCT). It is a cytotoxic drug that is administered intravenously.  Treosulfan is commonly used as part of a conditioning regimen to prepare the patient's body for HSCT, particularly in cases where a reduced-intensity or non-myeloablative approach is desired. It is used in patients with various hematological malignancies, including acute myeloid leukemia (AML), myelodysplastic syndrome (MDS), and other high-risk hematologic cancers.  The main mechanism of action of treosulfan is its ability to crosslink DNA strands, leading to the inhibition of DNA replication and cell division. By damaging the DNA of cancer cells, treosulfan helps to suppress their growth and eliminate them from the body. It has both cytotoxic and immunosuppressive properties.  Compared to other alkylating agents, treosulfan is known to have a favorable toxicity profile with a lower risk of organ toxicity and fewer side effects. It is generally well-tolerated, allowing for successful conditioning and subsequent transplantation.  The specific use and dosing of treosulfan may vary depending on factors such as the patient's specific diagnosis, overall health, and transplant protocol. The administration of treosulfan is typically guided by an oncologist or hematologist specializing in HSCT who will determine the appropriate dosage and duration based on the individual patient's needs. | Treosulfan is an alkylating agent that is used in the field of oncology for the treatment of certain types of cancer, particularly in conditioning regimens prior to hematopoietic stem cell transplantation (HSCT). It is a cytotoxic drug that is administered intravenously.  Treosulfan is primarily used in patients with high-risk or advanced hematological malignancies, such as acute myeloid leukemia (AML), myelodysplastic syndrome (MDS), and other hematologic cancers. It is typically used as part of a conditioning regimen to prepare the patient's body for HSCT.  The main mechanism of action of treosulfan is its ability to damage the DNA within cancer cells. It forms covalent bonds with DNA, leading to cross-linking between DNA strands, which interferes with DNA replication and transcription. This ultimately leads to cell death.  Treosulfan is often preferred in conditioning regimens due to its lower toxicity compared to other alkylating agents. It has been shown to have a more favorable safety profile with reduced damage to organs and lower risk of side effects. This makes it a suitable option for patients who may be more susceptible to complications from traditional conditioning agents.  The specific use and dosage of treosulfan may vary depending on the patient's individual condition, treatment plan, and the healthcare team's recommendations. It is important to consult with an oncologist or hematologist for detailed information about treosulfan and its specific application in the context of individual patients. |
| P3 | The standard conditioning regimen for multiple myeloma undergoing autologous hematopoietic stem cell transplantation (HSCT) typically involves high-dose chemotherapy. The most commonly used conditioning regimen is known as melphalan-based conditioning.  Melphalan is an alkylating agent that is highly effective against multiple myeloma cells. It is administered intravenously as part of the conditioning regimen to eliminate residual myeloma cells and suppress the patient's immune system in preparation for the transplant.  The recommended dose of melphalan for conditioning in autologous HSCT for multiple myeloma is typically in the range of 140-200 mg/m² of body surface area. The precise dosage may depend on factors such as the patient's age, overall health, and the specific treatment protocol followed by the healthcare team.  It's important to note that the specific conditioning regimen and dosage can vary based on the patient's individual characteristics, the stage of the disease, and the protocols followed by the treating healthcare team. The decision regarding the appropriate conditioning regimen and dose should be made by the patient's hematologist or oncologist, who will consider various factors and tailor the treatment plan accordingly to achieve the best possible outcomes. | The standard conditioning regimen for multiple myeloma undergoing autologous hematopoietic stem cell transplantation (HSCT) often involves high-dose chemotherapy. The most commonly used conditioning regimen for multiple myeloma is melphalan-based conditioning.  Melphalan is an alkylating agent that is highly effective against multiple myeloma cells. It is administered intravenously as part of the conditioning regimen to eliminate residual myeloma cells and suppress the patient's immune system before the transplant.  The recommended dose of melphalan for conditioning in autologous HSCT for multiple myeloma is typically in the range of 140 to 200 mg/m² of body surface area. However, the precise dosage can vary based on several factors, including the patient's age, overall health, and the specific treatment protocol followed by the healthcare team.  The decision regarding the optimal conditioning regimen and dosage should be made by the patient's hematologist or oncologist, who will consider various factors and tailor the treatment plan to the individual patient. They will assess the patient's overall condition, disease stage, and specific risk factors to determine the appropriate dosage and duration of treatment.  It's important to consult with a healthcare professional specialized in multiple myeloma and HSCT to get accurate and personalized information regarding the specific conditioning regimen and recommended dose for an individual patient. | The standard conditioning regimen for multiple myeloma patients undergoing autologous hematopoietic stem cell transplantation (HSCT) typically involves high-dose chemotherapy with melphalan. Melphalan is an alkylating agent that is effective against myeloma cells.  The recommended dose of melphalan for conditioning in autologous HSCT for multiple myeloma is generally 200 mg/m² of body surface area. However, the specific dosage may vary depending on factors such as the patient's age, overall health, and individualized treatment plans determined by the healthcare team.  Typically, melphalan is administered intravenously as a single dose prior to the transplantation procedure. The purpose of the conditioning regimen is to eradicate remaining myeloma cells, suppress the patient's immune system, and create space in the bone marrow for the engraftment of the transplanted stem cells.  It's important to note that the recommended dose and conditioning regimen may vary based on the patient's specific characteristics, disease status, and the protocols followed by the treating healthcare team. The decision regarding the optimal conditioning regimen and dosage should be made by the patient's hematologist or oncologist, who will consider various factors and tailor the treatment plan to the individual patient's needs.  Consultation with a healthcare professional specialized in multiple myeloma and HSCT is essential for accurate and personalized information regarding the specific conditioning regimen and recommended dose for an individual patient. |
| P4 | Post-transplant cyclophosphamide (PTCy) as graft-versus-host disease (GVHD) prophylaxis is typically administered in the immediate post-transplant period. It is given on specific days following hematopoietic stem cell transplantation (HSCT) to help prevent the development of acute GVHD.  The standard schedule for PTCy administration is on days +3 and +4 after HSCT. This means that the first dose of cyclophosphamide is given on the third day after transplantation, followed by a second dose on the fourth day. The timing is crucial to target and eliminate activated T cells responsible for GVHD.  PTCy is part of a conditioning regimen that is used in certain transplant settings, such as haploidentical HSCT, where the donor is partially matched to the recipient. By giving cyclophosphamide after the transplant, it selectively targets and eliminates donor T cells that may cause GVHD while preserving the graft-versus-leukemia (GVL) effect.  It's important to note that the specific schedule and dosing of PTCy may vary based on the transplant center's protocols, the patient's individual characteristics, and the healthcare team's discretion. The timing and administration of PTCy should be discussed and determined by the transplant team overseeing the patient's care. | Post-transplant cyclophosphamide (PTCy) as graft-versus-host disease (GVHD) prophylaxis is typically administered in the early post-transplant period following hematopoietic stem cell transplantation (HSCT).  The standard schedule for PTCy administration is on days +3 and +4 after HSCT. This means that the first dose of cyclophosphamide is given on the third day after transplantation, followed by a second dose on the fourth day. This timing is crucial for targeting and eliminating activated donor T cells that could potentially cause GVHD.  PTCy is commonly used in certain transplant settings, such as haploidentical HSCT, where the donor is only partially matched to the recipient. It is given as part of the conditioning regimen to suppress the donor's immune response against the recipient's tissues and minimize the risk of GVHD while preserving the graft-versus-leukemia (GVL) effect.  However, it's important to note that the specific schedule and dosing of PTCy may vary depending on the transplant center's protocols, the patient's individual characteristics, and the healthcare team's discretion. The timing and administration of PTCy should be discussed and determined by the transplant team overseeing the patient's care. They will consider various factors, such as the patient's underlying condition, transplant type, and overall risk of GVHD, to determine the most appropriate schedule for PTCy administration. | Post-transplant cyclophosphamide (PTCy) as graft-versus-host disease (GVHD) prophylaxis is typically given shortly after hematopoietic stem cell transplantation (HSCT). The exact timing of PTCy administration may vary based on the specific transplant center's protocols and the patient's individual circumstances, but it is generally administered on specific days after HSCT.  The most common schedule for PTCy administration is on days +3 and +4 after HSCT. This means that the first dose of cyclophosphamide is given on the third day after transplantation, followed by a second dose on the fourth day. This timing is important for selectively targeting and eliminating donor T cells that may contribute to GVHD while preserving the graft-versus-leukemia (GVL) effect.  The administration of PTCy is part of a conditioning regimen that aims to prevent GVHD in certain transplant settings, such as haploidentical HSCT or mismatched unrelated donor HSCT. PTCy helps suppress the immune response of the donor cells against the recipient's tissues, reducing the risk of GVHD while still allowing for the beneficial anti-cancer effects of the transplant.  It's important to note that the specific timing and dosing of PTCy may vary depending on the transplant center's protocols, the patient's overall health, and the healthcare team's judgment. The decision regarding the exact timing and administration of PTCy should be made by the transplant team overseeing the patient's care. They will consider various factors, including the patient's risk factors, transplant type, and overall condition, to determine the most appropriate schedule for PTCy administration. |
| P5 | HLA typing, which refers to the determination of human leukocyte antigen (HLA) alleles, is an important process in identifying suitable donors for hematopoietic stem cell transplantation. In healthy donors, several sample types can be utilized for HLA typing. The choice of sample type may depend on factors such as feasibility, availability, and laboratory protocols. The common sample types used for HLA typing in healthy donors include:  1. Peripheral Blood: Peripheral blood samples are one of the most frequently used sample types for HLA typing. Blood samples are collected from the donor's vein and can provide sufficient DNA or cells for HLA analysis.  2. Buccal Swabs: Buccal swabs involve obtaining a sample of cells from the inner lining of the cheek using a cotton swab. These swabs are relatively non-invasive, easy to collect, and can provide an adequate sample for HLA typing.  3. Saliva Samples: Saliva samples can be an alternative to buccal swabs for HLA typing. Donors provide a saliva sample by spitting into a collection tube. Saliva contains cells that can be used for DNA analysis.  4. Bone Marrow: In some cases, bone marrow samples may be collected from healthy donors for HLA typing. This method involves the extraction of bone marrow cells through a procedure called bone marrow aspiration or biopsy. Bone marrow samples can provide a rich source of hematopoietic cells for HLA analysis.  The choice of sample type may vary depending on the laboratory's specific protocols, requirements, and the donor's ability to provide the sample. Peripheral blood samples are commonly preferred due to their convenience and availability. It's important to note that the HLA typing process typically involves specialized laboratories equipped to perform the analysis accurately. | When performing human leukocyte antigen (HLA) typing in healthy donors, several sample types can be utilized. The choice of sample type may depend on factors such as convenience, availability, and the specific laboratory's protocols. Here are some commonly used sample types for HLA typing in healthy donors:  1. Peripheral Blood: Peripheral blood is the most common sample type used for HLA typing. A venous blood sample is collected from the donor, and various techniques, such as polymerase chain reaction (PCR) or sequence-specific oligonucleotide probe (SSOP) hybridization, are employed to analyze the HLA genes.  2. Buccal Swabs: Buccal swabs, which collect cells from the inner cheek, can also be used for HLA typing. The swabs are rubbed against the cheek to collect epithelial cells, and these cells are then used for DNA extraction and subsequent HLA typing analysis.  3. Saliva Samples: Saliva samples can be utilized as an alternative to buccal swabs. The donor provides a saliva sample, usually by spitting into a collection tube. The saliva contains cells that can be used for DNA extraction and subsequent HLA typing.  4. Bone Marrow Aspirate: In some cases, when bone marrow donation is being considered, a bone marrow aspirate may be collected from the donor. The aspirate contains a mixture of bone marrow cells, including hematopoietic stem cells, which can be used for HLA typing analysis.  It's important to note that the specific sample type chosen may vary depending on the laboratory's capabilities and preferences. The sample collection and processing procedures should be performed following appropriate protocols and guidelines to ensure accurate and reliable HLA typing results. | When performing human leukocyte antigen (HLA) typing in healthy donors, the sample types commonly used are:  1. Peripheral Blood: Peripheral blood is the most common sample type used for HLA typing. It can be collected through a simple blood draw and contains various immune cells, including lymphocytes, which play a crucial role in HLA typing.  2. Buccal Swabs: Buccal swabs are another sample type that can be used for HLA typing. It involves gently swabbing the inside of the cheek to collect cells from the oral mucosa. Buccal swabs are non-invasive and easy to collect, making them a convenient option for HLA typing in healthy donors.  3. Saliva: Saliva samples can also be used for HLA typing. Saliva contains buccal epithelial cells and other cellular components that can be used for genetic analysis, including HLA typing.  4. Bone Marrow: In some cases, bone marrow samples may be collected from healthy donors for HLA typing, particularly when high-resolution typing or additional testing is required. Bone marrow aspiration involves using a needle to extract a small amount of bone marrow from the donor's hipbone or sternum.  It's important to note that the specific sample type used for HLA typing may depend on factors such as the laboratory's protocols, availability of equipment, and the specific requirements of the testing. The choice of sample type should be determined based on the expertise of the laboratory performing the HLA typing and the specific needs of the donor evaluation process. |
| P6 | When a patient receives high-dose cytarabine, a chemotherapy drug commonly used in the treatment of acute myeloid leukemia (AML), there is a risk of developing ocular toxicity known as cytarabine syndrome. Cytarabine syndrome can cause inflammation and irritation of the conjunctiva and cornea, leading to symptoms such as eye redness, itching, tearing, and in severe cases, visual disturbances.  To help alleviate the symptoms and minimize the risk of ocular toxicity, prophylactic or supportive measures are often taken. Eye drops containing corticosteroids, such as dexamethasone or prednisolone, are commonly recommended in these cases. The corticosteroid eye drops have anti-inflammatory properties and can help reduce ocular inflammation and discomfort associated with cytarabine syndrome.  It's important to note that the specific choice of eye drops and their usage may vary depending on the patient's individual circumstances and the preferences of the treating physician. The healthcare team overseeing the patient's treatment will provide specific guidance on the appropriate eye drops, dosage, and frequency of use to manage and prevent ocular toxicity during high-dose cytarabine therapy. | When a patient receives high-dose cytarabine, a chemotherapy drug, it can cause ocular toxicity in the form of conjunctivitis or keratitis. To manage these potential side effects and alleviate symptoms, certain eye drops may be recommended. The specific eye drops and treatment plan may vary based on the patient's individual circumstances and the healthcare provider's preferences. However, one commonly used eye drop in this context is preservative-free artificial tears.  Preservative-free artificial tears are recommended because they provide lubrication and moisture to the eyes without the potential irritants found in preserved eye drops. High-dose cytarabine can cause dryness and inflammation in the eyes, and preservative-free artificial tears can help alleviate these symptoms and maintain ocular comfort. These eye drops can be used multiple times a day or as needed to provide relief from dryness and irritation.  It's important to note that the specific recommendation for eye drops may vary depending on the patient's overall health, other medications they are taking, and any underlying eye conditions they may have. Therefore, it is always best to consult with the patient's healthcare provider or ophthalmologist for personalized recommendations tailored to the patient's specific needs. | When a patient receives high-dose cytarabine, it can cause ocular toxicity known as cytarabine syndrome or conjunctivitis. This condition can lead to eye irritation, redness, swelling, and discomfort. To manage these symptoms, eye drops are commonly used. The specific eye drops recommended may vary based on the individual patient and the preferences of the treating healthcare team. However, the following types of eye drops are commonly utilized:  1. Lubricating Eye Drops: Lubricating eye drops or artificial tears can help alleviate dryness and relieve discomfort in the eyes caused by cytarabine-induced conjunctivitis.  2. Steroid Eye Drops: Steroid eye drops, such as prednisolone or dexamethasone, may be prescribed to reduce inflammation and relieve symptoms associated with cytarabine syndrome. These drops help reduce redness, swelling, and irritation.  It's important for the patient to consult their healthcare team, including their oncologist or ophthalmologist, for specific guidance on which eye drops to use and the recommended dosage. The healthcare team will consider the severity of symptoms, the patient's medical history, and other individual factors when determining the appropriate treatment for cytarabine-induced conjunctivitis. |
| P7 | In general, individuals with a prior history of cancer are not automatically excluded from registering as volunteer hematopoietic cell donors for the Be the Match Registry. Each case is evaluated on an individual basis, considering factors such as the type of cancer, the treatment received, the time since completion of treatment, and the absence of recurrence or ongoing active disease.  In the case of a prior history of prostate cancer that required systemic chemotherapy, the eligibility for registration will depend on several factors:  1. Time Since Completion of Treatment: The individual's prior treatment, including chemotherapy, should have been completed a certain period ago to ensure sufficient time has passed and the person is in a stable and disease-free state. The specific time requirements may vary, but a common guideline is a waiting period of around 2 to 5 years after completion of treatment.  2. Absence of Recurrence or Active Disease: The individual should have no evidence of recurrence or ongoing active disease. This is typically assessed through follow-up evaluations, including imaging tests, laboratory markers, and clinical examination. If there is no evidence of cancer recurrence and the individual is considered to be in remission, they may be eligible for registration.  It's important to note that eligibility criteria can vary, and decisions regarding an individual's eligibility for registration are ultimately made by the Be the Match Registry or the respective hematopoietic cell transplantation center. It is recommended that the individual interested in registering contacts the registry directly to discuss their specific situation and determine their eligibility. | Individuals with a prior history of cancer, including prostate cancer, can typically still register as volunteer hematopoietic cell donors for the Be the Match Registry. Each case is evaluated individually based on factors such as the type of cancer, the treatment received, and the length of time since completion of treatment.  In the case of prostate cancer that required systemic chemotherapy and subsequent negative follow-up, the eligibility for registration will depend on several factors:  1. Time Since Completion of Treatment: There is usually a waiting period after completion of treatment before an individual can register. The specific waiting period may vary, but it is typically around 2 to 5 years after completion of treatment. This waiting period allows for a period of disease-free stability and ensures the individual's overall health and recovery.  2. Absence of Active Disease or Recurrence: It is important that there is no evidence of active disease or recurrence at the time of registration. This is typically assessed through follow-up evaluations, including imaging tests, laboratory markers, and clinical examination. If the individual has completed treatment and subsequent follow-up evaluations indicate no evidence of active disease or recurrence, they may be eligible for registration.  It's important to note that eligibility criteria can vary, and decisions regarding an individual's eligibility for registration are ultimately made by the Be the Match Registry or the respective hematopoietic cell transplantation center. It is recommended that the individual interested in registering contacts the registry directly to discuss their specific situation and determine their eligibility. They can provide guidance and clarify any specific requirements or considerations for individuals with a history of prostate cancer. | The eligibility of an individual with a prior history of prostate cancer, which required systemic chemotherapy but subsequently had negative follow-up, to register as a volunteer hematopoietic cell donor for the Be the Match Registry would depend on several factors.  While each case is evaluated individually, there are general guidelines regarding cancer history for potential donors:  1. Time Since Completion of Treatment: Typically, there is a waiting period after completing cancer treatment before an individual can register as a donor. The waiting period varies depending on the type and stage of cancer, as well as the specific treatment received. In the case of prostate cancer, the waiting period is often around 2 to 5 years after completion of treatment.  2. Absence of Active Disease or Recurrence: It is important that there is no evidence of active prostate cancer or recurrence at the time of registration. Follow-up evaluations, including imaging tests, laboratory markers, and clinical examination, are usually performed to assess the absence of active disease. If the individual has completed treatment and subsequent evaluations indicate no evidence of active cancer, they may be eligible for registration.  It's crucial to note that eligibility criteria can vary, and decisions regarding an individual's eligibility for registration are ultimately made by the Be the Match Registry or the respective hematopoietic cell transplantation center. The best course of action is for the individual to directly contact the registry or a healthcare professional involved in the registration process. They can provide specific guidance based on the individual's medical history and circumstances to determine eligibility. |
| P8 | The management and treatment options for a 48-year-old patient with multiple myeloma in complete remission after a second autologous transplant can vary depending on several factors, including the patient's individual characteristics and the specific recommendations of their healthcare team. Here are some potential options that might be offered to such a patient:  1. Maintenance Therapy: In patients with multiple myeloma who have achieved complete remission after transplant, maintenance therapy is often recommended to prolong the remission and improve overall outcomes. Commonly used maintenance therapies include immunomodulatory drugs (such as lenalidomide) or proteasome inhibitors (such as bortezomib). The duration of maintenance therapy can vary, but it is often given for a few years or until disease progression.  2. Consolidation Therapy: In some cases, consolidation therapy may be considered to further intensify treatment and enhance the depth of response. This may involve additional cycles of chemotherapy or high-dose therapy with autologous stem cell rescue. The specific regimen and duration of consolidation therapy depend on the patient's response to initial treatment and the recommendations of the healthcare team.  3. Regular Follow-up and Monitoring: After achieving complete remission, regular follow-up visits and monitoring are important to detect any signs of relapse or treatment-related complications. This includes periodic blood tests, imaging studies, and evaluation of relevant markers such as M-protein levels or light chain ratios. The frequency of follow-up visits and monitoring will be determined by the healthcare team based on the patient's specific situation.  It's important to note that treatment decisions should be made in collaboration with the patient's hematologist or oncologist, who will consider the patient's individual circumstances, risk factors, treatment history, and current guidelines. The recommended treatment options and duration may vary depending on the specific protocols followed by the healthcare team and any ongoing clinical trials or advancements in multiple myeloma treatment. | After achieving complete remission following a second autologous transplant for multiple myeloma, the management plan for a 48-year-old patient may involve several components. Here are some potential considerations:  1. Maintenance Therapy: Maintenance therapy is commonly recommended to prolong remission and improve outcomes. The choice of maintenance therapy may vary based on individual patient factors and institutional guidelines. Commonly used maintenance agents include immunomodulatory drugs (e.g., lenalidomide) or proteasome inhibitors (e.g., bortezomib). The duration of maintenance therapy is often determined by the patient's response and tolerability, typically ranging from several months to years.  2. Regular Monitoring and Follow-up: Close monitoring is crucial to detect any signs of disease relapse or treatment-related complications. This includes regular blood tests, imaging studies (e.g., skeletal survey, MRI, or PET-CT scans), and assessment of relevant markers such as M-protein levels or light chain ratios. The frequency of follow-up visits and monitoring will depend on the patient's specific situation and the recommendations of their healthcare team.  3. Supportive Care: Alongside ongoing treatment, supportive care measures should be provided to address any side effects of previous therapies, manage symptoms, and optimize the patient's overall well-being. This may involve interventions such as pain management, nutritional support, bone health management, and psychological support.  4. Clinical Trials: Depending on the patient's eligibility and the availability of clinical trials, participation in research studies may be considered. Clinical trials offer opportunities to access innovative treatments and contribute to advancing knowledge in multiple myeloma management.  The specific treatment plan, including the choice of maintenance therapy and its duration, should be tailored to the patient's individual characteristics, treatment history, risk factors, and overall health. It's important to consult with the patient's hematologist or oncologist, who can provide personalized recommendations based on their expertise and knowledge of the patient's case. | After achieving complete remission following a second autologous transplant for multiple myeloma, the management plan for a 48-year-old patient may involve several components. Here are some potential considerations:  1. Maintenance Therapy: Maintenance therapy is commonly recommended to prolong remission and improve overall outcomes. The specific agents and duration of maintenance therapy can vary based on individual patient factors and institutional guidelines. Commonly used maintenance therapies for multiple myeloma include immunomodulatory drugs (such as lenalidomide) or proteasome inhibitors (such as bortezomib). The duration of maintenance therapy is typically discussed with the patient's healthcare team and may range from several months to several years.  2. Regular Monitoring and Follow-up: Close monitoring is essential to detect any signs of disease relapse or treatment-related complications. This may involve regular blood tests to monitor disease markers such as M-protein levels and light chains, as well as periodic imaging studies such as skeletal surveys or PET-CT scans. The frequency and duration of follow-up visits will depend on the patient's specific circumstances and the recommendations of their healthcare team.  3. Supportive Care: Supportive care measures are important to address any side effects of previous treatments and optimize the patient's overall well-being. This may include pain management, management of treatment-related toxicities, nutritional support, bone health management, and psychological support. The patient's healthcare team will work together to provide comprehensive supportive care throughout the survivorship period.  4. Lifestyle Factors: Encouraging healthy lifestyle choices is also important for long-term well-being. This may include maintaining a balanced diet, engaging in regular physical activity, avoiding tobacco and excessive alcohol consumption, and managing stress levels. The patient should work with their healthcare team to develop a plan that promotes overall health and well-being.  The specific treatment plan and duration of maintenance therapy will be determined by the patient's individual characteristics, treatment history, response to treatment, and the recommendations of their healthcare team. It's crucial for the patient to have regular follow-up appointments and maintain open communication with their healthcare team to ensure optimal care and support throughout their remission journey. |
| P9 | Autologous hematopoietic stem cell transplantation (HSCT) is being investigated as a potential treatment option for select patients with severe and refractory multiple sclerosis (MS). HSCT involves collecting a patient's own stem cells, followed by intense immunosuppressive therapy and subsequent reinfusion of the stem cells. This process aims to reset the immune system and potentially halt the progression of MS.  However, it's important to note that autologous HSCT for MS is still considered an experimental and evolving therapy. The decision to pursue HSCT for MS should be made on an individual basis and involves careful consideration of several factors:  1. Disease Severity: HSCT for MS is typically considered in patients with aggressive, highly active, and treatment-refractory disease that is causing significant disability and impacting quality of life. It is not typically considered as a first-line treatment or for patients with mild or stable disease.  2. Failed Conventional Therapies: Autologous HSCT is typically considered after patients have failed multiple lines of conventional therapies for MS. These may include disease-modifying therapies (DMTs) such as immunomodulatory drugs or monoclonal antibodies.  3. Patient Suitability: HSCT is an intensive and potentially high-risk procedure. Patient selection is crucial, and candidates should undergo a thorough evaluation to assess their overall health, organ function, and ability to withstand the procedure. Age, comorbidities, and other medical considerations play a role in determining suitability for HSCT.  4. Expert Evaluation and Centers of Excellence: HSCT for MS should be performed at specialized centers with expertise in both MS and transplant medicine. These centers have established protocols and multidisciplinary teams to ensure appropriate patient selection, comprehensive evaluation, and optimal management of the procedure and subsequent care.  It's important for patients considering autologous HSCT for MS to discuss the potential risks, benefits, and uncertainties with their healthcare team. The decision to pursue HSCT should be based on a thorough understanding of the individual patient's condition, available evidence, and a shared decision-making process involving the patient, their family, and the treating physicians. | Autologous hematopoietic stem cell transplantation (HSCT) has been explored as a potential treatment option for select patients with severe and refractory multiple sclerosis (MS). The current evidence suggests that autologous HSCT may be considered in specific cases; however, it is important to note that it is still considered an experimental and evolving therapy for MS.  Autologous HSCT for MS involves collecting a patient's own stem cells, followed by intensive immunosuppressive therapy and subsequent reinfusion of the stem cells. The goal is to reset the immune system and potentially halt the progression of the disease.  Here are some key considerations regarding autologous HSCT for MS:  1. Disease Severity and Progression: Autologous HSCT is typically considered for patients with highly active and aggressive forms of MS that have not responded to conventional therapies. It may be an option for patients with rapidly progressing disease, significant disability, or frequent relapses.  2. Failed Conventional Therapies: Autologous HSCT is usually considered when other disease-modifying therapies (DMTs) have been ineffective or intolerable. Patients should have tried and failed multiple lines of conventional treatments before considering HSCT.  3. Patient Selection: Careful patient selection is crucial. Candidates for autologous HSCT should undergo a comprehensive evaluation to assess their overall health, organ function, and ability to tolerate the procedure. Age, comorbidities, and other medical considerations are taken into account.  4. Expert Evaluation and Specialized Centers: Autologous HSCT for MS should only be performed at specialized centers with expertise in both MS and transplant medicine. These centers have established protocols, experienced multidisciplinary teams, and appropriate infrastructure to ensure patient safety and optimal outcomes.  It's important to emphasize that autologous HSCT for MS is still considered an experimental therapy, and the long-term safety and effectiveness of the treatment are not yet fully established. Patients considering this option should have thorough discussions with their healthcare team, including neurologists and transplant specialists, to understand the potential risks, benefits, and uncertainties associated with the procedure.  The decision to pursue autologous HSCT for MS should be based on a comprehensive evaluation of the individual patient's condition, informed discussions, and a shared decision-making process involving the patient, their family, and their healthcare team. | As an AI language model, I can provide general information, but I cannot offer specific medical advice. Decisions regarding medical treatments, such as autologous hematopoietic stem cell transplantation (HSCT), should be made in consultation with qualified healthcare professionals who can consider the individual patient's unique circumstances and medical history.  That being said, autologous HSCT has been considered as a potential treatment for multiple sclerosis (MS) in certain cases. HSCT involves the collection and freezing of a patient's own stem cells, followed by high-dose chemotherapy to deplete the immune system, and then reinfusion of the collected stem cells to rebuild the immune system.  The rationale behind autologous HSCT for MS is based on the theory that by resetting the immune system, it may help to halt the progression of the disease. The procedure aims to eliminate autoreactive immune cells that attack the myelin sheath in the central nervous system, which is a characteristic feature of MS.  The use of autologous HSCT in MS is still an area of active research, and its long-term effectiveness and safety are still being evaluated. Current evidence suggests that HSCT may be most beneficial for individuals with highly active relapsing-remitting MS who have failed to respond to other treatments. It is typically not considered a first-line therapy and is generally reserved for patients with aggressive disease who have exhausted other available treatment options.  It's important to note that autologous HSCT is an intensive procedure associated with significant risks, including infection, organ damage, and even death. Therefore, careful patient selection and comprehensive evaluation of the potential risks and benefits are essential when considering this treatment option.  Ultimately, the decision to pursue autologous HSCT for MS should be made on an individual basis, considering the patient's specific circumstances and in consultation with a medical professional experienced in the treatment of MS. |
| P10 | In the context of allogeneic hematopoietic stem cell transplantation (HSCT), a major ABO blood group mismatch between the patient and the peripheral blood stem cell (PBSC) donor can present challenges. However, in certain situations, a major ABO mismatch can still be acceptable with appropriate management.  To determine the suitability of the donor in this scenario, additional tests and considerations are required, which may include:  1. ABO Antibody Titers: The patient's serum should be tested for the presence and titer of ABO antibodies. Higher titers of antibodies indicate a greater risk of hemolysis and other complications.  2. Donor Selection: The donor's ABO subtype should be evaluated to determine its compatibility with the patient. The donor's ABO subgroup may affect the risk and severity of potential complications.  3. Pre-Transplant Conditioning Regimen: The intensity of the conditioning regimen plays a role in reducing host ABO antibodies, as it can affect the recipient's bone marrow and peripheral blood cell populations. Certain conditioning regimens can help reduce the recipient's ABO antibodies and mitigate potential complications.  4. Hematologic Support: Adequate hematologic support should be available to manage potential complications, such as hemolysis or transfusion requirements. This includes blood products matched to the patient's ABO type, as well as appropriate monitoring and interventions.  5. Compatibility Assessments: Other compatibility assessments, such as HLA matching and cross-matching, should be performed to evaluate the overall suitability of the donor and to minimize the risk of graft-versus-host disease (GVHD) and other complications.  It is crucial to involve an experienced transplant team, including hematologists, immunologists, and transplant coordinators, who can assess the specific patient-donor combination and make an informed decision. They will consider the potential risks and benefits of proceeding with a major ABO mismatch and develop an individualized plan for the transplant, taking into account the patient's clinical status, urgency of the procedure, availability of alternative donors, and other relevant factors. | A major ABO mismatch between a patient and a peripheral blood stem cell (PBSC) donor can present challenges in hematopoietic stem cell transplantation (HSCT). In general, it is preferred to have an ABO-compatible donor for HSCT. However, under certain circumstances, a major ABO mismatch can be managed.  To determine the suitability of the donor with a major ABO mismatch, several factors need to be considered:  1. Patient's Condition: The patient's underlying medical condition and the urgency of the transplant are important factors. In certain situations, such as when there are no alternative donors available, a major ABO mismatch may be accepted if the potential benefits of the transplant outweigh the risks.  2. Donor-Recipient Relationship: The relationship between the patient and the donor is important. If the donor is an HLA-matched sibling or a closely matched unrelated donor, efforts may be made to overcome the ABO mismatch.  3. Risk of Graft-versus-Host Disease (GVHD): A major ABO mismatch increases the risk of graft-versus-host disease, as ABO antigens are present on various tissues and can serve as targets for immune response. The extent of the ABO mismatch and the potential consequences of GVHD need to be carefully evaluated.  4. ABO Antibodies: The presence of preformed ABO antibodies in the patient is an important consideration. If the patient has high levels of pre-existing antibodies against the donor's ABO type, it may pose a higher risk of complications and influence the decision-making process.  In addition to these considerations, several tests and evaluations are typically performed to assess the feasibility and risks of proceeding with a major ABO mismatch:  1. ABO Typing: Both the patient and the donor should undergo ABO blood typing to determine the specific ABO antigens present.  2. Donor-Specific Antibody (DSA) Testing: DSA testing helps identify if the patient has any pre-formed antibodies against the donor's ABO type or other HLA antigens. This information is crucial in assessing the risk of immune reactions.  3. HLA Typing: Complete HLA typing of the patient and the donor is essential for overall compatibility assessment and to ensure the closest HLA match possible.  4. Risk Assessment: The healthcare team, including transplant physicians and immunologists, will evaluate the risks associated with the ABO mismatch and consider the overall impact on graft survival, engraftment, and the risk of complications such as GVHD.  The decision to proceed with HSCT in the presence of a major ABO mismatch is complex and requires careful consideration of multiple factors. A multidisciplinary team of experts, including transplant physicians, hematologists, immunologists, and transfusion medicine specialists, will assess the risks and benefits and make a decision based on the individual patient's circumstances. | When a patient and peripheral blood stem cell (PBSC) donor have a major ABO mismatch, it means that the patient and the donor have different ABO blood types. In such cases, the suitability of the donor depends on several factors, including the specific transplant center's policies and the patient's condition. Here are some considerations:  1. Donor Selection: In general, an ABO mismatch between the patient and the donor is not an absolute contraindication for allogeneic stem cell transplantation. With appropriate management, successful transplantation can still be achieved. However, the specific decision regarding donor suitability will depend on several factors, including the urgency of the transplantation, availability of alternative donors, and the risks associated with an ABO mismatch.  2. Compatibility Assessment: In cases of ABO mismatch, additional tests are typically performed to assess the compatibility between the patient and the donor. These tests include:  - ABO Antibody Titration: This test measures the levels of ABO antibodies present in the patient's blood. Higher antibody titers may increase the risk of hemolysis (destruction of red blood cells) or other complications.  - Crossmatching: A crossmatch test is performed to determine the compatibility between the patient's serum (containing antibodies) and the donor's cells. This test helps evaluate the risk of graft rejection or graft-versus-host disease (GVHD).  3. Pre-Transplant Conditioning: Depending on the specific ABO mismatch and the transplant center's protocols, pre-transplant conditioning regimens may be adjusted to minimize the risk of complications related to ABO mismatch, such as hemolysis. This may include additional treatments or modifications to the conditioning regimen.  It's important to consult with the healthcare team, including the transplant physician or hematologist, to thoroughly evaluate the risks and benefits of proceeding with an ABO-mismatched transplant. The decision will be based on a comprehensive assessment of the patient's condition, the availability of alternative donors, and the expertise of the transplantation team. |
| P11 | The Disease Risk Index (DRI) is a scoring system used to assess the risk of disease relapse and overall survival following allogeneic hematopoietic stem cell transplantation (HSCT) for acute myeloid leukemia (AML). The DRI takes into account several factors, including cytogenetics, disease status at transplantation, and pre-transplant comorbidities. Each factor is assigned a score, and the total score determines the patient's risk category.  To determine the DRI score and overall survival at 2 years for a 32-year-old patient with intermediate risk cytogenetics in first complete remission, I would need more specific information, such as the specific cytogenetic abnormalities and any additional risk factors or comorbidities. The DRI score and survival rates can vary based on these factors.  It's important to note that the DRI is a tool used by healthcare professionals to guide treatment decisions, assess prognosis, and provide a general risk estimation. The actual outcome for an individual patient depends on numerous factors beyond the DRI score, including the patient's overall health, response to treatment, and individual characteristics.  For a comprehensive assessment of the patient's prognosis and treatment options, I recommend consulting with the patient's healthcare team, including hematologists or oncologists specializing in AML and transplantation. They will have access to the most up-to-date clinical data and can provide personalized information and guidance based on the patient's specific case. | The Disease Risk Index (DRI) is a scoring system used to assess the risk of disease relapse and overall survival following allogeneic hematopoietic stem cell transplantation (HSCT) for acute myeloid leukemia (AML). The DRI takes into account several factors, including cytogenetics, disease status at transplantation, and pre-transplant comorbidities. Each factor is assigned a score, and the total score determines the patient's risk category.  For a 32-year-old patient with intermediate-risk cytogenetics in first complete remission, the DRI score and overall survival at 2 years can vary depending on additional factors. Without specific information about the patient's cytogenetic profile, additional risk factors, or comorbidities, it is not possible to provide an accurate DRI score or precise survival estimate.  It is important to consult with the patient's healthcare team, including hematologists or oncologists specializing in AML and transplantation, to obtain a comprehensive assessment of the patient's prognosis. They will have access to the most up-to-date clinical data, can evaluate the specific risk factors, and provide personalized information and guidance based on the patient's individual circumstances. | I apologize for the confusion caused by my previous responses. As an AI language model, I don't have access to real-time data or patient-specific information. The Disease Risk Index (DRI) is a scoring system used to assess the risk of disease relapse and overall survival following allogeneic hematopoietic stem cell transplantation (HSCT) for acute myeloid leukemia (AML).  To determine the specific DRI score and overall survival at 2 years for a 32-year-old patient with intermediate-risk cytogenetics in first complete remission, it is best to consult with the patient's healthcare team. They will have access to the patient's complete medical history, including the specific cytogenetic profile, and can evaluate other relevant factors to provide an accurate assessment.  The DRI score and survival rates can vary based on various factors, such as specific cytogenetic abnormalities, molecular mutations, and additional risk factors. The healthcare team, including hematologists or oncologists specializing in AML and transplantation, will be able to provide the most up-to-date and personalized information based on the patient's individual case.  It's essential to consult with the healthcare team for a comprehensive evaluation and to discuss the specific DRI score and expected outcomes for the patient in question. |
| P12 | A patient undergoing hematopoietic stem cell transplantation (HSCT) with donor-specific antibodies (DSA) is at risk for a specific complication known as graft-versus-host disease (GVHD). GVHD occurs when the transplanted donor cells recognize the recipient's tissues as foreign and mount an immune response against them. DSA can contribute to the development of GVHD by facilitating the recognition and attack of the recipient's tissues by the donor's immune system.  GVHD can manifest as acute or chronic, depending on the timing and duration of its onset. Acute GVHD typically occurs within the first 100 days after transplantation, while chronic GVHD develops later. Both types can affect various organs and systems in the body, leading to a range of symptoms and complications.  The specific complications associated with GVHD can vary, but commonly affected areas include the skin, liver, and gastrointestinal tract. Symptoms may include skin rash, jaundice, diarrhea, abdominal pain, and organ dysfunction. GVHD can significantly impact the patient's quality of life and may require additional interventions, such as immunosuppressive medications or other treatments to manage the condition.  It's important for the patient undergoing HSCT with DSA to be closely monitored by their healthcare team, as early detection and prompt intervention can help manage GVHD and its associated complications effectively. | In the context of hematopoietic stem cell transplantation (HSCT), the term "DSA" commonly refers to Donor-Specific Antibodies. These are antibodies produced by the recipient's immune system that specifically target and react against the antigens present on the donor's cells.  When a patient undergoing HSCT has DSA, they are at an increased risk of developing a specific complication known as antibody-mediated rejection (AMR). AMR occurs when the recipient's pre-formed antibodies recognize and attack the transplanted donor cells. This immune response can lead to graft failure or dysfunction.  AMR can manifest in various organs and tissues, depending on the target antigens recognized by the DSA. The complications can vary, but some potential risks associated with AMR in HSCT patients with DSA include:  1. Graft failure: The presence of DSA increases the likelihood of the recipient's immune system rejecting the transplanted graft, leading to graft failure and the need for re-transplantation.  2. Kidney complications: If the HSCT includes kidney transplantation or if there is concurrent kidney dysfunction, AMR can result in kidney damage, impaired graft function, or graft loss.  3. Lung complications: In cases of lung transplantation during HSCT, AMR can contribute to lung graft dysfunction or rejection.  4. Other organ complications: Depending on the specific antigens targeted by the DSA and the organs involved in the HSCT, complications such as liver dysfunction, heart failure, or skin graft rejection may occur.  Managing DSA and mitigating the risk of AMR in HSCT patients may involve various strategies, such as desensitization protocols, close monitoring of DSA levels, and selecting compatible donor grafts to minimize the risk of rejection. The management of DSA and associated complications is a complex and individualized process that requires close collaboration between the transplantation team and specialists in immunology and transplant rejection. | In the context of hematopoietic stem cell transplantation (HSCT), the term "DSA" typically refers to "donor-specific antibodies." These antibodies are produced by the recipient's immune system and are specific to the donor's tissue antigens. The presence of DSA can increase the risk of a complication known as graft-versus-host disease (GVHD).  GVHD occurs when the donor's immune cells (graft) recognize the recipient's tissues as foreign and attack them. It can affect multiple organs, including the skin, liver, and gastrointestinal tract. Acute GVHD typically develops within the first 100 days after HSCT, while chronic GVHD can occur later.  In the context of a patient with DSA undergoing HSCT, the risk of developing GVHD is increased, particularly in cases of mismatched or unrelated donors. The presence of DSA suggests a higher likelihood of immune incompatibility between the donor and recipient, increasing the risk of immune-mediated complications such as GVHD.  It's important for the patient and their healthcare team to closely monitor for signs and symptoms of GVHD and manage it promptly if it occurs. This may involve immunosuppressive medications to control the immune response and reduce the severity of GVHD. The specific management approach will depend on the individual patient's condition and the recommendations of their transplant team. |
| P13 | The treatment options for severe aplastic anemia (SAA) depend on various factors, including the patient's age, overall health, severity of the disease, availability of a suitable donor for a stem cell transplant, and the patient's preference. The primary treatment options for SAA include immunosuppressive therapy (IST) and allogeneic stem cell transplantation (SCT).  1. Immunosuppressive therapy (IST): IST involves using medications to suppress the immune system and halt the immune attack on the bone marrow. The standard IST regimen typically consists of a combination of anti-thymocyte globulin (ATG) and cyclosporine. This treatment option is generally considered for patients who are not eligible for immediate stem cell transplantation or who do not have a matched donor available. IST can be effective in achieving blood cell count recovery in a significant percentage of patients, leading to improved quality of life and long-term survival.  2. Allogeneic stem cell transplantation (SCT): Allogeneic SCT, also known as a bone marrow transplant, is the only potentially curative treatment for severe aplastic anemia. It involves replacing the patient's damaged bone marrow with healthy stem cells from a compatible donor. SCT can be highly effective in achieving long-term remission, but it requires a suitable donor, typically a matched sibling or an unrelated matched donor. The availability of a matched donor and the patient's overall health and preference play a crucial role in deciding whether SCT is the best treatment option.  The choice between IST and SCT depends on several factors, and it should be made in consultation with a hematologist or a specialist experienced in treating aplastic anemia. The physician will consider the patient's specific situation, age, overall health, disease severity, and the availability of a suitable donor to determine the most appropriate treatment approach.  It's important to note that each case is unique, and the treatment decision should be personalized based on individual circumstances. The healthcare team will evaluate the patient's specific factors to determine the best first treatment option for the 25-year-old patient with severe aplastic anemia. | The primary treatment options for a 25-year-old patient diagnosed with severe aplastic anemia (SAA) include immunosuppressive therapy (IST) and allogeneic stem cell transplantation (SCT). The choice between these options depends on various factors and should be made in consultation with a hematologist or a specialist experienced in treating aplastic anemia.  1. Immunosuppressive therapy (IST): IST involves using medications to suppress the immune system and halt the immune attack on the bone marrow. The standard IST regimen typically consists of a combination of anti-thymocyte globulin (ATG) and cyclosporine. IST is generally considered for patients who are not eligible for immediate stem cell transplantation or who do not have a matched donor available. It can be effective in achieving blood cell count recovery in a significant percentage of patients, leading to improved quality of life and long-term survival.  2. Allogeneic stem cell transplantation (SCT): Allogeneic SCT, also known as a bone marrow transplant, is the only potentially curative treatment for severe aplastic anemia. It involves replacing the patient's damaged bone marrow with healthy stem cells from a compatible donor. SCT can be highly effective in achieving long-term remission but requires a suitable donor, typically a matched sibling or an unrelated matched donor. The availability of a matched donor and the patient's overall health play a crucial role in deciding whether SCT is the best treatment option.  The specific treatment choice depends on factors such as the patient's age, overall health, disease severity, availability of a suitable donor, and individual preferences. It is important to have a thorough evaluation by a specialist who can consider all these factors and provide personalized recommendations.  The hematologist or specialist experienced in treating aplastic anemia will assess the patient's specific circumstances and determine the best first treatment option for the 25-year-old patient with severe aplastic anemia. | The primary treatment options for a 25-year-old patient diagnosed with severe aplastic anemia (SAA) include immunosuppressive therapy (IST) and allogeneic stem cell transplantation (SCT). The choice of treatment depends on several factors and should be made in consultation with a hematologist or a specialist experienced in treating aplastic anemia.  1. Immunosuppressive therapy (IST): IST involves using medications to suppress the immune system and halt the immune attack on the bone marrow. The standard IST regimen typically consists of a combination of anti-thymocyte globulin (ATG) and cyclosporine. IST is generally considered for patients who are not eligible for immediate stem cell transplantation or who do not have a suitable donor available. It can be effective in achieving blood cell count recovery in a significant percentage of patients, leading to improved quality of life and long-term survival.  2. Allogeneic stem cell transplantation (SCT): Allogeneic SCT, also known as a bone marrow transplant, is the only potentially curative treatment for severe aplastic anemia. It involves replacing the patient's damaged bone marrow with healthy stem cells from a compatible donor. SCT can provide a long-term cure, but it requires finding a suitable donor, typically a matched sibling or an unrelated matched donor. The availability of a matched donor and the patient's overall health play a crucial role in determining the feasibility of SCT as the first treatment option.  The specific treatment choice depends on various factors, including the patient's age, overall health, disease severity, availability of a suitable donor, and individual preferences. A thorough evaluation by a hematologist or specialist experienced in treating aplastic anemia is essential to consider these factors and provide personalized recommendations.  The hematologist or specialist will assess the patient's specific circumstances, evaluate the risk-benefit ratio of each treatment option, and determine the best first treatment approach for the 25-year-old patient with severe aplastic anemia. |
| P14 | When a 32-year-old patient is diagnosed with NPM1 mutated acute myeloid leukemia (AML) with a normal karyotype and achieves complete molecular remission after induction but subsequently experiences a return of minimal residual disease (MRD) positivity after the second consolidation cycle, the best subsequent approach depends on several factors. The specific treatment decision should be made in consultation with a hematologist or an oncologist experienced in treating AML.  Here are some possible subsequent approaches that may be considered:  1. Intensification of chemotherapy: If the patient is fit and eligible for further chemotherapy, intensification of the treatment regimen may be considered. This could involve additional cycles of chemotherapy with or without targeted therapies to further reduce the MRD burden.  2. Allogeneic stem cell transplantation (SCT): Allogeneic SCT, also known as a bone marrow transplant, is a potentially curative treatment option for AML. If a suitable donor is available, and the patient is considered eligible, allogeneic SCT may be recommended to eliminate the MRD and prevent disease relapse. The decision to proceed with SCT involves assessing the patient's overall health, availability of a matched donor, and the risk-benefit balance.  3. Targeted therapy: Depending on the specific molecular characteristics of the patient's AML, targeted therapies may be considered. For example, in cases of FLT3-ITD mutation, tyrosine kinase inhibitors like midostaurin or gilteritinib may be utilized. However, the specific targeted therapy options and their availability can vary based on the patient's molecular profile and regional approvals.  4. Clinical trial participation: Clinical trials evaluating novel treatment approaches, such as new targeted therapies or immunotherapies, may be an option for patients who are eligible and willing to participate. Clinical trials offer access to investigational treatments that may provide additional therapeutic options and contribute to advancing the field of AML treatment.  The subsequent approach will depend on multiple factors, including the patient's overall health, specific molecular characteristics of the AML, available treatment options, and individual preferences. It is crucial to consult with the patient's healthcare team, who can thoroughly evaluate the case and provide personalized recommendations based on the latest scientific evidence and clinical expertise. | When a 32-year-old patient is diagnosed with NPM1 mutated acute myeloid leukemia (AML) with a normal karyotype and achieves complete molecular remission after induction but subsequently experiences a return of minimal residual disease (MRD) positivity after the second consolidation cycle, the best subsequent approach depends on several factors. The specific treatment decision should be made in consultation with a hematologist or an oncologist experienced in treating AML.  Here are some possible subsequent approaches that may be considered:  1. Allogeneic stem cell transplantation (SCT): Allogeneic SCT, also known as a bone marrow transplant, is a potentially curative treatment option for AML. If a suitable donor is available, and the patient is considered eligible and fit for transplant, allogeneic SCT may be recommended to eliminate the MRD and prevent disease relapse. SCT offers a chance for long-term remission or cure but carries risks and complications associated with the transplant process.  2. Intensification of chemotherapy or targeted therapy: If the patient is not eligible for or prefers not to undergo SCT, intensification of chemotherapy with additional cycles or the use of targeted therapies may be considered. This could involve treatment with agents specific to the patient's molecular profile, such as FLT3 inhibitors or other targeted therapies based on specific genetic mutations.  3. Participation in a clinical trial: Clinical trials evaluating novel treatment approaches, such as new drugs, targeted therapies, or immunotherapies, may be an option for eligible patients. Participating in a clinical trial can provide access to innovative treatments that may offer improved outcomes or contribute to the development of future treatment options.  The subsequent approach will depend on multiple factors, including the patient's overall health, specific molecular characteristics of the AML, available treatment options, and individual preferences. It is crucial to consult with the patient's healthcare team, who can thoroughly evaluate the case and provide personalized recommendations based on the latest scientific evidence, clinical expertise, and the patient's specific circumstances and preferences. | When a 32-year-old patient is diagnosed with NPM1 mutated acute myeloid leukemia (AML) with a normal karyotype and achieves complete molecular remission after induction but subsequently experiences a return of minimal residual disease (MRD) positivity after the second consolidation cycle, the best subsequent approach depends on several factors. The specific treatment decision should be made in consultation with a hematologist or an oncologist experienced in treating AML.  Here are some possible subsequent approaches that may be considered:  1. Allogeneic stem cell transplantation (SCT): Allogeneic SCT, also known as a bone marrow transplant, is a potentially curative treatment option for AML. If a suitable donor is available, and the patient is considered eligible and fit for transplant, allogeneic SCT may be recommended to eliminate the MRD and prevent disease relapse. SCT offers a chance for long-term remission or cure but carries risks and complications associated with the transplant process.  2. Alternative consolidation therapy: If the patient is not eligible for or prefers not to undergo SCT, alternative consolidation therapies may be considered. These may include additional cycles of chemotherapy with or without targeted therapies to further reduce the MRD burden and prevent disease recurrence. The choice of chemotherapy regimen and targeted therapies depends on the specific molecular profile of the AML, such as additional mutations or genetic alterations.  3. Clinical trial participation: Clinical trials evaluating novel treatment approaches for AML may be an option for eligible patients. Participating in a clinical trial provides access to investigational treatments that may offer improved outcomes or contribute to the development of new therapies. The specific trial options will depend on the patient's eligibility criteria and the availability of relevant clinical trials in their region.  The subsequent approach will depend on multiple factors, including the patient's overall health, specific molecular characteristics of the AML, available treatment options, and individual preferences. It is crucial to consult with the patient's healthcare team, who can thoroughly evaluate the case and provide personalized recommendations based on the latest scientific evidence, clinical expertise, and the patient's specific circumstances and preferences. |
| P15 | When choosing between two unrelated donors for allogeneic hematopoietic stem cell transplantation (HSCT), several factors should be considered to determine the best option. Here are some factors to consider in the given scenario:  1. Age: The age of the donor can be a relevant factor. Generally, younger donors tend to have better overall outcomes in terms of engraftment, reduced risk of complications, and longer-term survival. In this case, the first donor is a 20-year-old male, which may be considered an advantage compared to the 42-year-old female donor.  2. Pregnancy history: The history of pregnancies in a female donor can potentially affect the transplant outcome due to the presence of fetal-maternal microchimerism. It is possible for fetal cells from prior pregnancies to persist in the mother's body and potentially cause complications, such as graft rejection or graft-versus-host disease (GVHD). However, the exact impact of prior pregnancies on transplant outcomes is not well-defined and may vary. It would be important to consult with a transplant specialist who can assess the specific risks associated with the female donor's pregnancy history and make an informed decision.  3. AB0 compatibility: AB0 compatibility refers to matching the blood types of the donor and the recipient. In this scenario, both donors have the same AB0 blood group, which eliminates the concern of major AB0 incompatibility and reduces the risk of potential complications associated with AB0 mismatch.  Considering these factors, the 20-year-old male donor may be the preferred choice due to the potential advantage of youth, which is associated with better outcomes in HSCT. However, it is crucial to consult with a transplant specialist who can evaluate the patient's individual circumstances, assess the potential risks associated with the female donor's pregnancy history, and provide a personalized recommendation based on the patient's specific needs and the available donor options. The decision should be made in close collaboration with the transplant team to optimize the chances of a successful transplantation outcome. | In the given scenario, when considering two unrelated donors for allogeneic hematopoietic stem cell transplantation (HSCT), several factors should be taken into account to determine the best choice. Here are some important considerations:  1. Age: Younger donors, such as the 20-year-old male donor in this case, generally have better outcomes in terms of engraftment, lower risk of complications, and longer-term survival after HSCT. Age can play a significant role in the success of the transplant, as younger donors often have healthier stem cells with better regenerative potential.  2. Pregnancy history: The pregnancy history of the female donor, who has had two pregnancies, introduces the possibility of fetal-maternal microchimerism. Fetal cells from previous pregnancies can persist in a woman's body and potentially lead to complications such as graft rejection or graft-versus-host disease (GVHD). However, the impact of prior pregnancies on transplant outcomes is not well-defined, and the specific risks associated with the donor's pregnancy history should be evaluated by a transplant specialist.  3. AB0 compatibility: It is advantageous that both donors have the same AB0 blood group, as it eliminates the concern of major AB0 incompatibility. AB0 compatibility reduces the risk of potential complications associated with AB0 mismatch in HSCT.  Considering these factors, the 20-year-old male donor may be the preferred choice due to the potential advantages of youth, including better stem cell quality and improved transplant outcomes. However, the decision should be made in consultation with a transplant specialist who can thoroughly evaluate the specific risks associated with the female donor's pregnancy history and provide personalized recommendations based on the patient's individual circumstances.  The expertise of the transplant team is crucial in assessing the potential risks and benefits of each donor and making an informed decision that maximizes the chances of a successful transplantation outcome for the male patient with hematologic malignancy. | When considering the choice between two unrelated donors for allogeneic hematopoietic stem cell transplantation (HSCT) for a male patient with a hematologic malignancy, several factors should be taken into consideration. Here are some important points to consider:  1. Age: Generally, younger donors have better outcomes in terms of engraftment, lower risk of complications, and longer-term survival after HSCT. In this case, the 20-year-old male donor may be preferred due to the potential advantages of youth.  2. Pregnancy history: The history of pregnancies in a female donor can potentially affect the transplant outcome due to the presence of fetal-maternal microchimerism. Fetal cells from prior pregnancies can persist in the mother's body and potentially cause complications, such as graft rejection or graft-versus-host disease (GVHD). While the specific impact of prior pregnancies on transplant outcomes is not well-defined and can vary, it is an important consideration. The transplant team should evaluate the specific risks associated with the female donor's pregnancy history and make an informed decision based on that assessment.  3. AB0 compatibility: It is advantageous that both donors have the same AB0 blood group, as it eliminates the concern of major AB0 incompatibility. AB0 compatibility reduces the risk of potential complications associated with AB0 mismatch in HSCT.  Considering these factors, the 20-year-old male donor may be the preferred choice due to the potential advantages of youth and the absence of a pregnancy history. However, the final decision should be made by the transplant team, who can thoroughly assess the specific risks and benefits associated with each donor and provide personalized recommendations based on the patient's individual circumstances.  It's important to consult with the transplant team to carefully evaluate and discuss the potential risks and benefits of each donor option in order to make the best decision for the patient's successful transplantation outcome. |
| P16 | When evaluating a cord blood transplant for an adult patient with hematologic malignancy, several cord blood parameters should be evaluated. These parameters help determine the suitability and compatibility of the cord blood unit for transplantation. Here are some important cord blood parameters to consider:  1. Total nucleated cell (TNC) count: The TNC count represents the total number of nucleated cells in the cord blood unit. Higher TNC counts are generally associated with better engraftment and outcomes after transplantation.  2. CD34+ cell count: CD34+ cells are a specific subset of stem cells responsible for generating new blood cells. Evaluating the CD34+ cell count helps assess the stem cell content of the cord blood unit. Higher CD34+ cell counts are generally associated with better engraftment and faster hematopoietic recovery.  3. HLA matching: Human leukocyte antigens (HLA) are proteins on the surface of cells that are important for immune compatibility. The degree of HLA matching between the cord blood unit and the patient should be assessed. Higher levels of HLA matching increase the likelihood of successful engraftment and reduce the risk of graft-versus-host disease (GVHD).  4. Cell viability: The viability of the cells within the cord blood unit should be evaluated. This assessment determines the percentage of viable cells, which indicates the overall quality and potency of the cord blood unit.  5. Infectious disease screening: Cord blood units should be screened for infectious diseases, such as viruses (e.g., HIV, hepatitis) and bacteria, to ensure the safety of the transplant.  6. ABO and Rh compatibility: Evaluating the ABO and Rh blood group compatibility between the cord blood unit and the recipient helps minimize the risk of ABO and Rh incompatibility-related complications.  These parameters help assess the quality, quantity, and compatibility of the cord blood unit for transplantation. The evaluation is typically performed by the transplant team in collaboration with a cord blood bank. It is important to consult with the transplant team, who will have access to the specific requirements and guidelines for cord blood selection and can determine the most suitable cord blood unit for the adult patient with hematologic malignancy. | When evaluating a cord blood transplant for an adult patient with hematologic malignancy, several cord blood parameters should be evaluated to assess the suitability of the cord blood unit. Here are some important cord blood parameters to consider:  1. Total nucleated cell (TNC) count: The TNC count reflects the total number of nucleated cells, including hematopoietic stem cells and other cell types, in the cord blood unit. A higher TNC count is generally associated with better engraftment and faster hematopoietic recovery after transplantation.  2. CD34+ cell count: CD34+ cells are a specific subset of stem cells responsible for regenerating the blood and immune system. Evaluating the CD34+ cell count helps assess the stem cell content in the cord blood unit. Higher CD34+ cell counts are generally associated with improved engraftment and better outcomes.  3. HLA matching: Human leukocyte antigens (HLA) are proteins on the surface of cells that play a crucial role in immune compatibility. The HLA compatibility between the cord blood unit and the recipient should be assessed. Higher levels of HLA matching increase the likelihood of successful engraftment and reduce the risk of graft-versus-host disease (GVHD).  4. Cell viability: The viability of the cells within the cord blood unit should be evaluated to determine the percentage of viable cells. Higher cell viability indicates better quality and potential for successful engraftment.  5. Infectious disease screening: Cord blood units should undergo comprehensive infectious disease screening to detect and rule out the presence of infectious agents such as viruses (e.g., HIV, hepatitis), bacteria, and parasites. This is done to ensure the safety of the transplant for the recipient.  6. ABO and Rh compatibility: Evaluating the ABO and Rh blood group compatibility between the cord blood unit and the recipient is important to minimize the risk of ABO and Rh incompatibility-related complications.  These parameters help assess the quality, quantity, and compatibility of the cord blood unit for transplantation. The evaluation is typically conducted by the transplant team in collaboration with a cord blood bank. It is essential to consult with the transplant team, who will have access to specific guidelines and protocols for cord blood selection and can determine the most suitable cord blood unit for the adult patient with hematologic malignancy. | When evaluating a cord blood transplant for an adult patient with hematologic malignancy, several cord blood parameters should be evaluated to assess the suitability of the cord blood unit. Here are some important cord blood parameters to consider:  1. Total nucleated cell (TNC) count: The TNC count represents the total number of nucleated cells in the cord blood unit. A higher TNC count is generally associated with better engraftment and faster hematopoietic recovery after transplantation.  2. CD34+ cell count: CD34+ cells are a specific subset of stem cells responsible for regenerating the blood and immune system. Evaluating the CD34+ cell count helps assess the stem cell content in the cord blood unit. Higher CD34+ cell counts are generally associated with improved engraftment and better outcomes.  3. HLA matching: Human leukocyte antigens (HLA) are proteins on the surface of cells that play a crucial role in immune compatibility. The HLA compatibility between the cord blood unit and the recipient should be assessed. A closer HLA match between the cord blood unit and the patient increases the likelihood of successful engraftment and reduces the risk of graft-versus-host disease (GVHD).  4. Cell viability: The viability of the cells within the cord blood unit should be evaluated to determine the percentage of viable cells. Higher cell viability indicates better quality and potential for successful engraftment.  5. Infectious disease screening: Cord blood units should undergo comprehensive infectious disease screening to detect and rule out the presence of infectious agents such as viruses (e.g., HIV, hepatitis), bacteria, and parasites. This is done to ensure the safety of the transplant for the recipient.  6. ABO and Rh compatibility: Evaluating the ABO and Rh blood group compatibility between the cord blood unit and the recipient is important to minimize the risk of ABO and Rh incompatibility-related complications.  These parameters help assess the quality, quantity, and compatibility of the cord blood unit for transplantation. The evaluation is typically conducted by the transplant team in collaboration with a cord blood bank. It is essential to consult with the transplant team, who will have access to specific guidelines and protocols for cord blood selection and can determine the most suitable cord blood unit for the adult patient with hematologic malignancy. |
| P17 | The Hematopoietic Cell Transplantation Comorbidity Index (HCT CI) is a scoring system used to assess the comorbidities and overall fitness of a patient undergoing allogeneic hematopoietic stem cell transplantation (HSCT). It helps predict the risk of non-relapse mortality and overall survival after HSCT.  The HCT CI takes into account various comorbidities and patient characteristics, including age, performance status, and presence of specific medical conditions. The score is calculated based on the presence or absence of specific comorbidities.  In the case of the 32-year-old patient being considered for allogeneic HSCT for acute myeloid leukemia (AML), the provided information indicates an unremarkable medical history other than a previous basal cell cancer that did not require systemic treatment. Basal cell cancer is generally not considered a significant comorbidity for HSCT, especially if it did not require systemic therapy.  Without additional information about the patient's age, performance status, and other relevant comorbidities, it is not possible to determine the exact HCT CI score for this patient. The HCT CI score requires a comprehensive evaluation by the healthcare team, including a review of the patient's medical history, physical examination, and potentially additional tests.  It is recommended to consult with the patient's healthcare team, specifically hematologists or oncologists experienced in HSCT, who can assess the patient's overall health, perform a detailed evaluation, and provide an accurate HCT CI score along with personalized information and recommendations for the patient's specific case. | The Hematopoietic Cell Transplantation Comorbidity Index (HCT CI) is a scoring system used to assess the comorbidities and overall fitness of a patient undergoing allogeneic hematopoietic stem cell transplantation (HSCT). The HCT CI score takes into account various comorbidities and patient characteristics to estimate the risk of non-relapse mortality and overall survival after HSCT.  Given the provided information, the patient's medical history is unremarkable except for a previous basal cell cancer that did not require systemic treatment. Basal cell cancer is generally not considered a significant comorbidity for HSCT, particularly if it did not require systemic therapy. Therefore, it is unlikely to significantly contribute to the HCT CI score.  To determine the exact HCT CI score for the patient, additional information would be needed, such as the patient's age, performance status, and the presence of other comorbidities. These factors, along with the specific criteria and scoring system used by the healthcare team, would contribute to calculating the HCT CI score accurately.  It is important to consult with the patient's healthcare team, including hematologists or oncologists experienced in HSCT, who can assess the patient's overall health, perform a comprehensive evaluation, and provide an accurate HCT CI score along with personalized information and recommendations for the patient's specific case. | I apologize for the confusion in my previous responses. As an AI language model, I don't have access to real-time data or specific scoring systems like the Hematopoietic Cell Transplantation Comorbidity Index (HCT CI). The HCT CI score requires a comprehensive evaluation by the healthcare team, taking into account various factors such as age, comorbidities, performance status, and other relevant medical information.  To determine the HCT CI score for a 32-year-old patient being considered for allogeneic HSCT for acute myeloid leukemia, it is necessary to consult with the patient's healthcare team. They will have access to the patient's medical history, perform a thorough assessment, and calculate the appropriate HCT CI score based on the specific scoring system used by their institution.  I apologize for any confusion caused. It is recommended to discuss the HCT CI score and its implications with the patient's healthcare team, as they are in the best position to provide accurate information and personalized recommendations based on the patient's individual circumstances. |
| P18 | Selecting CD34-positive stem cells for a stem cell boost in a patient with poor graft function after allogeneic hematopoietic stem cell transplantation (HSCT) can offer several advantages:  1. Engraftment Potential: CD34 is a surface marker expressed on hematopoietic stem cells (HSCs) and progenitor cells. CD34-positive cells have a high engraftment potential, meaning they have the ability to home to the bone marrow and establish hematopoiesis more efficiently. By selecting CD34-positive cells for the boost, there is an increased likelihood of successful engraftment and subsequent hematopoietic recovery.  2. Hematopoietic Recovery: CD34-positive stem cells have the capacity to differentiate into various blood cell lineages, including red blood cells, white blood cells, and platelets. By selecting CD34-positive cells, the aim is to replenish the patient's bone marrow with functional hematopoietic cells, promoting a more robust and sustained recovery of blood cell production.  3. Immune Reconstitution: CD34-positive stem cells not only contribute to hematopoietic recovery but also play a crucial role in immune reconstitution. These cells can give rise to immune cells, such as T cells, B cells, and natural killer (NK) cells, which are essential for a properly functioning immune system. Selecting CD34-positive cells for the boost can help enhance immune reconstitution, providing the patient with better protection against infections and improved immune surveillance against malignancies.  It's important to note that the selection of CD34-positive cells for a stem cell boost should be done based on careful consideration of the patient's individual condition, including the underlying cause of poor graft function and the available donor source. The decision should be made in consultation with the patient's healthcare team, including the transplant physician or hematologist, to determine the most appropriate approach for the specific case. | When a patient with poor graft function after allogeneic hematopoietic stem cell transplantation (HSCT) is offered a stem cell boost, selecting CD34+ cells as the source of stem cells can offer several advantages:  1. Hematopoietic Stem Cell Enrichment: CD34 is a marker present on hematopoietic stem cells. Selecting CD34+ cells ensures a higher enrichment of true hematopoietic stem cells in the stem cell boost, increasing the likelihood of successful engraftment and hematopoietic recovery.  2. Enhanced Graft Potential: CD34+ cells have the capacity to differentiate into all types of blood cells, including red blood cells, white blood cells, and platelets. By selecting CD34+ cells, the stem cell boost can provide a broader range of hematopoietic potential, which is important for achieving adequate blood cell production and restoring the patient's immune system.  3. Faster Engraftment: CD34+ cells have been associated with faster engraftment and hematopoietic recovery compared to other stem cell sources. Rapid engraftment is crucial for minimizing the risk of infections, improving the patient's overall condition, and reducing the need for supportive therapies.  4. Lower Risk of Graft-versus-Host Disease (GVHD): CD34+ cells are less likely to contain mature T cells, which are responsible for graft-versus-host disease. By selecting CD34+ cells as the source of stem cells, there is a reduced risk of GVHD, a potentially severe complication of allogeneic HSCT.  It's important to note that the selection of stem cell source, including CD34+ cells, should be based on individual patient factors, including the underlying condition, graft function, and the expertise of the transplant team. The decision should be made in consultation with the patient's healthcare team to determine the most appropriate approach for the stem cell boost. | Selecting CD34+ stem cells for a stem cell boost in a patient with poor graft function after allogeneic hematopoietic stem cell transplantation (HSCT) can offer several advantages. CD34 is a cell surface marker expressed on hematopoietic stem cells (HSCs) and progenitor cells. Here are some potential advantages of selecting CD34+ cells for a stem cell boost:  1. Enrichment of Stem Cells: CD34+ cells represent a population of cells enriched with hematopoietic stem cells and progenitor cells. By selecting CD34+ cells, the aim is to increase the number of functional stem cells and progenitors in the boost, which can help enhance the graft function.  2. Repopulation Potential: CD34+ stem cells have the capability to self-renew and differentiate into various types of blood cells. By selecting CD34+ cells, there is a higher likelihood of introducing stem cells with a greater repopulation potential, which can improve the long-term engraftment and restoration of hematopoiesis.  3. Faster Hematopoietic Recovery: The use of CD34+ cells in a stem cell boost can potentially expedite hematopoietic recovery. These cells have a higher capacity for multilineage differentiation and can help restore the patient's blood cell counts more rapidly.  4. Lower Risk of Immune Rejection: CD34+ cells are derived from the same donor as the original stem cell transplant. By selecting CD34+ cells, the risk of immune rejection or graft failure is minimized since they are less likely to be recognized as foreign by the recipient's immune system.  5. Reduced Risk of Graft-versus-Host Disease (GVHD): CD34+ cells contain a lower number of mature T cells, which are responsible for graft-versus-host disease. By selecting CD34+ cells, there is a decreased risk of GVHD, as the boost would introduce fewer T cells that could potentially cause an immune response against the recipient's tissues.  It's important to note that the selection of CD34+ cells for a stem cell boost should be based on the individual patient's condition, the underlying cause of graft failure, and the recommendations of the transplant team. The advantages mentioned above are general considerations, and the specific approach should be tailored to the patient's unique circumstances. |
| **Section 3 Infection** | | | |
| I1 | Post-transplant lymphoproliferative disorder (PTLD) is often caused by the Epstein-Barr virus (EBV). EBV is a common virus that infects most people at some point in their lives. In immunocompetent individuals, EBV typically causes mild symptoms or goes unnoticed. However, in immunosuppressed individuals, such as transplant recipients, EBV can lead to the development of PTLD.  In PTLD cases associated with EBV, the patient donor serology most commonly associated with this condition is seronegative EBV donor and seropositive EBV recipient. This means that the donor does not have antibodies against EBV, while the recipient has previously been infected with EBV and has antibodies against the virus. In such cases, the recipient's immune system is unable to control the replication of the EBV virus from the seronegative donor, leading to uncontrolled lymphocyte proliferation and the development of PTLD.  It's important to note that PTLD can also occur due to other viruses, such as cytomegalovirus (CMV) and human herpesvirus 8 (HHV-8), particularly in specific clinical settings. However, EBV remains the most common viral cause of PTLD, and the seronegative EBV donor/seropositive EBV recipient scenario is frequently associated with the development of PTLD. | Post-transplant lymphoproliferative disorder (PTLD) is often caused by the Epstein-Barr virus (EBV). EBV is a common virus that infects most people at some point in their lives. In immunocompetent individuals, EBV typically causes mild symptoms or goes unnoticed. However, in immunosuppressed individuals, such as transplant recipients, EBV can lead to the development of PTLD.  In PTLD cases associated with EBV, the patient donor serology most commonly associated with this condition is seronegative EBV donor and seropositive EBV recipient. This means that the donor does not have antibodies against EBV, while the recipient has previously been infected with EBV and has antibodies against the virus. In such cases, the recipient's immune system is unable to control the replication of the EBV virus from the seronegative donor, leading to uncontrolled lymphocyte proliferation and the development of PTLD.  It's important to note that PTLD can also occur due to other viruses, such as cytomegalovirus (CMV) and human herpesvirus 8 (HHV-8), particularly in specific clinical settings. However, EBV remains the most common viral cause of PTLD, and the seronegative EBV donor/seropositive EBV recipient scenario is frequently associated with the development of PTLD. | Post-transplant lymphoproliferative disorder (PTLD) is often caused by the Epstein-Barr virus (EBV). EBV is a common virus that infects most people at some point in their lives. In immunocompetent individuals, EBV typically causes mild symptoms or goes unnoticed. However, in immunosuppressed individuals, such as transplant recipients, EBV can lead to the development of PTLD.  Regarding the patient-donor serology associated with PTLD, there are different patterns based on the EBV serostatus of the patient and the donor. The most common scenario associated with PTLD is a seronegative EBV donor and a seropositive EBV recipient. This means that the donor does not have antibodies against EBV, while the recipient has previously been infected with EBV and has antibodies against the virus. In this situation, the recipient's immune system is unable to control the replication of EBV from the seronegative donor, leading to uncontrolled lymphocyte proliferation and the development of PTLD.  It's important to note that PTLD can also occur in other serostatus combinations, such as when both the recipient and the donor are seropositive for EBV. The risk and development of PTLD can be influenced by factors such as the intensity of immunosuppression, the time since transplantation, and the presence of other risk factors.  The specific patient-donor serology and risk of PTLD should be assessed by the transplant team based on the individual patient's characteristics, including their EBV serostatus and the potential risk factors associated with the transplantation. |
| I2 | If a patient undergoing allogeneic hematopoietic stem cell transplantation (HSCT) is not compliant with the sulfamethoxazole/trimethoprim prophylaxis, they may be at an increased risk for developing Pneumocystis jirovecii pneumonia (PJP). PJP is a potentially serious fungal infection that can occur in immunocompromised individuals, including HSCT recipients.  Sulfamethoxazole/trimethoprim (commonly known as Bactrim or Septra) is commonly prescribed as prophylaxis against PJP in HSCT recipients due to its effectiveness in preventing this infection. PJP is caused by the fungus Pneumocystis jirovecii, which can be found in the environment. In immunocompromised individuals, such as HSCT recipients, PJP can lead to severe respiratory symptoms and potentially life-threatening complications.  By not complying with the sulfamethoxazole/trimethoprim prophylaxis, the patient's protection against PJP is compromised, and they become more susceptible to developing the infection. It is important for HSCT recipients to adhere to their prescribed prophylactic regimen to minimize the risk of PJP and other opportunistic infections.  If a patient is unable to tolerate sulfamethoxazole/trimethoprim or is non-compliant with the prophylaxis regimen, alternative prophylactic strategies may be considered. This decision should be made in consultation with the transplant team, who can assess the individual patient's risk factors, specific circumstances, and provide alternative recommendations to mitigate the risk of PJP or other opportunistic infections.  It is essential for the patient to communicate their concerns or difficulties with prophylactic medications to their healthcare team so that appropriate alternatives or adjustments to the prophylaxis regimen can be considered to maintain their health and safety after HSCT. | If a patient undergoing allogeneic hematopoietic stem cell transplantation (HSCT) is not compliant with sulfamethoxazole/trimethoprim (SMX/TMP) prophylaxis, they may be at an increased risk for a specific complication known as Pneumocystis jirovecii pneumonia (PJP).  PJP is a potentially severe fungal infection caused by the microorganism Pneumocystis jirovecii. It primarily affects immunocompromised individuals, including HSCT recipients who have weakened immune systems due to the transplantation process and immunosuppressive medications.  Sulfamethoxazole/trimethoprim (SMX/TMP) is commonly prescribed as prophylaxis against PJP in HSCT recipients. This medication helps prevent the development of PJP by suppressing the growth of Pneumocystis jirovecii.  If a patient is non-compliant with the SMX/TMP prophylaxis, their protection against PJP may be compromised. This increases the risk of acquiring PJP, which can lead to severe respiratory symptoms, including cough, shortness of breath, fever, and potentially life-threatening complications.  It is essential for HSCT recipients to adhere to the prescribed prophylactic medications, such as SMX/TMP, as recommended by their healthcare team. Regular follow-up and communication with the medical team can help address any concerns or challenges regarding medication compliance and ensure appropriate preventive measures are in place to minimize the risk of complications, including PJP. | If a patient undergoing allogeneic hematopoietic stem cell transplantation (HSCT) is not compliant with sulfamethoxazole/trimethoprim (SMX/TMP) prophylaxis, they may be at an increased risk for a specific complication known as Pneumocystis jirovecii pneumonia (PJP), also referred to as Pneumocystis pneumonia.  PJP is a potentially severe fungal infection caused by the Pneumocystis jirovecii organism. It is an opportunistic infection that primarily affects individuals with weakened immune systems, such as transplant recipients who are on immunosuppressive medications.  SMX/TMP is commonly prescribed as prophylaxis against PJP in HSCT recipients due to its effectiveness in preventing this infection. However, if the patient is non-compliant with the prophylactic medication, the risk of developing PJP increases.  PJP can present with symptoms such as fever, cough, shortness of breath, and chest pain. It can be a serious and potentially life-threatening infection, particularly in immunocompromised individuals.  It is essential for patients undergoing allogeneic HSCT to adhere to the prescribed prophylactic medications, including SMX/TMP, to minimize the risk of PJP and other opportunistic infections. It is important to discuss any concerns or challenges with medication compliance with the healthcare team, as alternative strategies or interventions may be considered to ensure the patient's safety during the transplant process. |
| I3 | Letermovir is an antiviral medication that targets the human cytomegalovirus (CMV). It specifically inhibits the viral terminase complex, which is essential for the replication of CMV. By targeting the terminase complex, letermovir prevents the cleavage and packaging of viral DNA, thereby inhibiting viral replication and the spread of CMV infection. Letermovir is primarily used for the prophylaxis and treatment of CMV infection in adult CMV-seropositive recipients of an allogeneic hematopoietic stem cell transplant. | Letermovir is an antiviral medication used to prevent cytomegalovirus (CMV) infection and disease in adult CMV-seropositive recipients of an allogeneic hematopoietic stem cell transplant (HSCT). Letermovir specifically targets the viral terminase complex, which is an enzyme essential for the replication of CMV.  The terminase complex plays a crucial role in CMV DNA packaging during viral replication. Letermovir inhibits the function of the viral terminase complex, preventing proper packaging of viral DNA into newly formed CMV particles. By targeting the terminase complex, letermovir disrupts viral replication and helps to prevent CMV infection in HSCT recipients.  Letermovir is typically used as a prophylactic treatment and is administered orally or intravenously, depending on the formulation. It is often initiated shortly before HSCT and continued for a certain duration after the transplant, as determined by the healthcare team.  It's important to note that letermovir is specific to the prevention of CMV infection in HSCT recipients and does not have activity against other viruses or infections. The use of letermovir should be guided by the healthcare team, who will assess the patient's individual risk factors and determine the appropriate treatment plan. | Letermovir is an antiviral medication used for the prevention of cytomegalovirus (CMV) infection and disease in adult CMV-seropositive recipients of an allogeneic hematopoietic stem cell transplant (HSCT). It targets the viral terminase complex, specifically the UL51 protein, which is essential for viral DNA packaging during the late phase of CMV replication.  By inhibiting the UL51 protein, letermovir prevents the formation of infectious CMV virions, reducing the risk of CMV reactivation and subsequent CMV-related complications in transplant recipients. It acts by interfering with the viral DNA maturation process, thereby inhibiting viral replication and reducing the viral load.  Letermovir is administered orally or intravenously and is typically initiated shortly before or after the HSCT procedure. Its use as prophylaxis aims to prevent CMV infection in high-risk patients who have undergone allogeneic HSCT, helping to improve outcomes and reduce the complications associated with CMV reactivation.  It's important to note that letermovir is a specific antiviral agent targeting CMV and does not have activity against other viruses. It should be used as part of a comprehensive management plan under the guidance of healthcare professionals experienced in the treatment of CMV in transplant recipients. |
| I4 | The drug that is FDA-approved for cytomegalovirus (CMV) prophylaxis in allogeneic hematopoietic stem cell transplant (HSCT) recipients is letermovir (brand name: Prevymis). Letermovir is specifically indicated for the prevention of CMV infection and disease in adult CMV-seropositive HSCT recipients.  Letermovir is administered orally or intravenously and works by inhibiting the viral terminase complex, which is essential for CMV replication. It is typically given as a prophylactic treatment to HSCT recipients at high risk of CMV reactivation or disease, such as those who are CMV-seropositive or CMV-seronegative recipients from CMV-seropositive donors.  It is important to note that treatment decisions should be made in consultation with a healthcare professional, as individual patient factors and guidelines may vary. | The drug that is FDA-approved for cytomegalovirus (CMV) prophylaxis in allogeneic hematopoietic stem cell transplant (HSCT) recipients is called letermovir (brand name: Prevymis). Letermovir is specifically indicated for the prophylaxis of CMV infection and disease in adult CMV-seropositive recipients of an allogeneic HSCT. It is used to prevent CMV reactivation and reduce the risk of CMV-related complications in this patient population. Letermovir is typically administered orally or intravenously as part of the transplant management protocol under the guidance of a healthcare professional. | The drug that is FDA-approved for cytomegalovirus (CMV) prophylaxis in allogeneic hematopoietic stem cell transplant (HSCT) recipients is called letermovir (brand name: Prevymis). Letermovir is specifically indicated for the prophylaxis of CMV infection and disease in adult CMV-seropositive recipients of an allogeneic HSCT. It is used to prevent the reactivation of CMV and its associated complications in high-risk patients undergoing HSCT. Letermovir works by targeting the viral terminase complex of CMV, inhibiting viral replication and spread. It is important to note that the choice of CMV prophylaxis may vary depending on the specific guidelines and practices followed by individual transplant centers, so it's always best to consult with the healthcare team regarding the most appropriate and FDA-approved treatment options. |
| I5 | Among the human herpes viruses, herpes simplex virus (HSV) is the one most often associated with encephalitis, including cases in cord blood transplant recipients.  Herpes simplex encephalitis (HSE) is primarily caused by HSV type 1 (HSV-1), although rare cases of HSV type 2 (HSV-2) encephalitis have been reported as well. HSE is a severe and potentially life-threatening condition characterized by inflammation of the brain. It can lead to significant neurological symptoms, including altered mental status, seizures, focal neurological deficits, and other signs of brain dysfunction.  Cord blood transplant recipients, like other immunocompromised individuals, are at an increased risk of developing viral infections due to their weakened immune system. HSV can reactivate and cause encephalitis in these patients. Reactivation can occur due to immunosuppression from conditioning regimens, graft-versus-host disease (GVHD) treatment, or other factors.  Early diagnosis and prompt treatment are crucial in cases of suspected HSV encephalitis. Antiviral medications, such as acyclovir or its derivatives, are typically used for treatment. The specific management approach may vary based on the severity of symptoms, the patient's overall condition, and the individual transplant center's protocols.  It's important to note that while HSV is the most common herpes virus associated with encephalitis, other herpesviruses, such as varicella-zoster virus (VZV) and human herpesvirus 6 (HHV-6), can also cause encephalitis in specific situations. The epidemiology and clinical presentation may differ for these different herpesviruses, and appropriate diagnostic tests are required for accurate identification and management of the specific viral infection. | Among cord blood transplant recipients, the human herpesvirus 6 (HHV-6) has been more frequently associated with encephalitis. HHV-6 is a common virus that infects most people during childhood, typically causing mild symptoms such as fever and rash. However, in immunocompromised individuals, such as those who have undergone cord blood transplantation, HHV-6 can cause more severe complications, including encephalitis.  HHV-6 encephalitis is a known complication in cord blood transplant recipients and can manifest as neurological symptoms such as seizures, altered mental status, and focal neurological deficits. The virus can directly invade the central nervous system (CNS) and cause inflammation and damage to brain tissue, leading to encephalitis.  Early diagnosis and prompt treatment of HHV-6 encephalitis in cord blood transplant recipients are crucial. Antiviral medications such as ganciclovir or foscarnet may be used to manage HHV-6 infection. Additionally, supportive care measures, including management of seizures and neurologic symptoms, may be implemented.  It's important to note that while HHV-6 is commonly associated with encephalitis in cord blood transplant recipients, other herpesviruses, such as herpes simplex virus (HSV) and varicella-zoster virus (VZV), can also cause encephalitis in this population. The specific viral etiology may vary depending on individual patient factors and the presence of other risk factors or coinfections. | Among the human herpes viruses, herpes simplex virus (HSV) is the virus most commonly associated with encephalitis, including cases occurring in cord blood transplant recipients.  HSV encephalitis is a severe viral infection that affects the central nervous system, particularly the brain. It is usually caused by HSV type 1 (HSV-1), although HSV type 2 (HSV-2) can also be a causative agent, especially in cases related to sexual transmission.  Cord blood transplant recipients are at an increased risk of viral infections due to their weakened immune system following the transplant procedure. HSV encephalitis can occur as a result of reactivation of a latent infection or through primary infection in a seronegative recipient from a seropositive donor.  Prompt diagnosis and treatment with antiviral medications, such as acyclovir or ganciclovir, are essential in managing HSV encephalitis. Early recognition of symptoms, such as fever, altered mental status, seizures, and focal neurological deficits, is crucial for initiating appropriate treatment and improving outcomes.  While HSV is the most commonly associated herpes virus with encephalitis, it's important to note that other herpes viruses, such as varicella-zoster virus (VZV) and human herpesvirus 6 (HHV-6), can also cause encephalitis, albeit less frequently. The specific herpesvirus causing encephalitis may vary depending on factors such as the patient population, underlying conditions, and geographical location. |
| I6 | Prior to initiating rituximab treatment, several serological assessments are commonly performed to evaluate the patient's status and guide the use of rituximab. The specific serologies that are typically assessed prior to rituximab treatment include:  1. Hepatitis B Virus (HBV) Serology: Testing for HBV serology is important because rituximab can potentially reactivate HBV in individuals who are HBV carriers or have prior exposure to HBV. Serological markers such as HBsAg (hepatitis B surface antigen), anti-HBs (antibodies against hepatitis B surface antigen), and anti-HBc (antibodies against hepatitis B core antigen) may be evaluated.  2. Hepatitis C Virus (HCV) Serology: HCV serology is assessed to determine if the patient has a current or past infection with HCV. Rituximab treatment can be associated with HCV reactivation in individuals with HCV infection, so assessing the HCV serological status is important.  3. Human Immunodeficiency Virus (HIV) Serology: HIV serology is checked to identify patients with HIV infection. Rituximab treatment does not have a direct effect on HIV, but it is important to assess the patient's HIV status for comprehensive care and to monitor potential drug interactions or overlapping treatment considerations.  4. Varicella-Zoster Virus (VZV) Serology: Serological assessment for VZV antibodies helps determine the patient's immunity to varicella-zoster virus, which causes chickenpox and herpes zoster (shingles). In patients who are not immune, vaccination or appropriate prophylactic measures may be considered prior to rituximab treatment.  These serological assessments help to identify patients who may be at risk for viral reactivation or complications during rituximab treatment. The results of these tests can guide the use of preventive measures, monitoring, or concurrent therapies to minimize the risk of adverse events related to viral infections.  It's important to note that the specific serological assessments may vary based on regional guidelines, institutional protocols, and individual patient factors. The decision to perform these serologies should be made in consultation with the patient's healthcare team, including the prescribing physician or hematologist/oncologist, who will consider the patient's medical history, risk factors, and treatment plan. | Prior to initiating rituximab treatment, several serological tests are typically assessed to evaluate the patient's immune status and identify potential risks or contraindications. The specific serology tests that are commonly performed before rituximab treatment include:  1. Hepatitis B Virus (HBV) Serology:  - HBsAg (Hepatitis B surface antigen): This tests for the presence of the hepatitis B virus in the bloodstream. A positive result may indicate an active HBV infection.  - Anti-HBc (Hepatitis B core antibody): This tests for previous exposure to HBV. A positive result may indicate prior infection, even if the infection is no longer active.  - Anti-HBs (Hepatitis B surface antibody): This tests for the presence of antibodies against the hepatitis B surface antigen. A positive result indicates immunity to HBV or prior vaccination.  2. Hepatitis C Virus (HCV) Serology:  - Anti-HCV (Hepatitis C antibody): This tests for the presence of antibodies against the hepatitis C virus. A positive result may indicate a current or past HCV infection.  3. Human Immunodeficiency Virus (HIV) Serology:  - HIV-1 and HIV-2 Antibodies: These tests detect antibodies to the human immunodeficiency virus. A positive result indicates an HIV infection.  4. Varicella-Zoster Virus (VZV) Serology:  - Varicella-Zoster Virus Antibodies: This test determines the presence of antibodies against the varicella-zoster virus, which causes chickenpox and shingles. A positive result indicates immunity to VZV or prior vaccination.  These serology tests help assess the patient's infectious disease status and guide treatment decisions. Positive results for certain infections may require additional evaluation or precautions before starting rituximab, as they can increase the risk of complications or influence the management approach. The patient's healthcare team, including the treating physician or oncologist, will interpret the results and determine the appropriate course of action based on the specific serology findings and the individual patient's condition. | Before initiating treatment with rituximab, it is important to assess several serological markers to evaluate the patient's immune status and potential risks. The specific serological tests that are commonly recommended prior to rituximab treatment include:  1. Hepatitis B Virus (HBV) Serology: HBV serological testing is crucial to determine the patient's hepatitis B status. It involves assessing markers such as hepatitis B surface antigen (HBsAg), hepatitis B surface antibody (anti-HBs), and hepatitis B core antibody (anti-HBc). Rituximab can increase the risk of HBV reactivation in patients who are chronically infected or have past exposure to HBV.  2. Hepatitis C Virus (HCV) Serology: Testing for HCV antibodies is important to determine the patient's hepatitis C status. Rituximab treatment is not known to increase the risk of HCV reactivation, but it is still essential to assess the patient's HCV serology to have a comprehensive understanding of their viral status.  3. Human Immunodeficiency Virus (HIV) Serology: HIV serology is evaluated to determine the patient's HIV status. Rituximab treatment does not directly impact HIV, but it is important to identify patients with coexisting HIV infection to ensure appropriate management and monitoring.  4. Varicella-Zoster Virus (VZV) Serology: VZV serological testing assesses the patient's immunity against varicella-zoster virus, which causes chickenpox and shingles. Rituximab treatment can increase the risk of VZV reactivation, and knowledge of the patient's VZV serology helps guide preventive measures such as vaccination or antiviral prophylaxis.  These serological assessments are essential to evaluate the patient's baseline viral status and identify any preexisting infections or risks that could influence the safety and management of rituximab therapy. Depending on the patient's medical history and clinical presentation, additional serological tests or evaluations may be recommended as per the specific needs and guidelines provided by the healthcare team. |
| I7 | When a patient undergoing hematopoietic stem cell transplantation (HSCT) develops a disseminated vesicular rash and is non-compliant with medications, it is important to consider the possibility of a viral infection, such as herpes simplex virus (HSV) or varicella-zoster virus (VZV). In such cases, contact precautions should be implemented to prevent the spread of the virus to healthcare workers and other patients.  The specific contact precautions that should be used in this scenario include:  1. Standard contact precautions: Standard contact precautions are the basic infection control measures that should be followed for all patients. This includes proper hand hygiene, using gloves when in contact with the patient or potentially contaminated surfaces, and using appropriate personal protective equipment (PPE) when necessary.  2. Droplet precautions: Since the rash is disseminated, additional precautions may be required to prevent droplet transmission of infectious agents. This includes using a surgical mask or respirator when in close proximity to the patient or during procedures that may generate respiratory secretions.  3. Airborne precautions (if indicated): If there is a strong suspicion of airborne transmission, such as in the case of varicella-zoster virus (chickenpox), airborne precautions should be implemented. This includes the use of respirators or masks capable of filtering airborne particles, along with appropriate isolation in a negative pressure room.  It is crucial to consult with the healthcare team and infection control specialists in the specific healthcare facility to determine the exact precautions needed based on the patient's condition, the suspected or confirmed infectious agent, and any local guidelines or policies in place. Adhering to the appropriate precautions helps protect healthcare workers and other patients from potential transmission of the virus. | When a patient undergoing hematopoietic stem cell transplantation (HSCT) develops a disseminated vesicular rash during inpatient care, and there is non-compliance with medications, it raises concern for the possibility of herpes zoster (shingles) infection. In such cases, it is appropriate to implement Contact Precautions to prevent the transmission of the virus to other individuals.  Contact Precautions involve the use of personal protective equipment (PPE) and specific infection control measures to minimize the risk of transmission through direct or indirect contact with the patient or their environment. The specific measures for Contact Precautions include:  1. Hand hygiene: Healthcare providers should perform proper hand hygiene using soap and water or an alcohol-based hand sanitizer before and after patient contact.  2. Gloves: Gloves should be worn when entering the patient's room or when there is direct contact with the patient or potentially contaminated surfaces or objects in the patient's environment.  3. Gown: A gown should be worn when there is a possibility of direct contact with the patient or the patient's environment.  4. Patient placement: Ideally, the patient should be placed in a private room to minimize the risk of transmission to other individuals. If a private room is not available, the patient may be placed in a room with individuals who have the same infection or a compatible illness.  It is important to consult with the healthcare team and infection control specialists in the specific healthcare facility for guidance on implementing appropriate precautions based on the individual patient's condition, the suspected or confirmed diagnosis, and the facility's infection control policies.  Additionally, prompt medical evaluation and appropriate antiviral treatment for herpes zoster should be initiated for the patient to help alleviate symptoms, prevent complications, and reduce the risk of further transmission. | When a patient undergoing hematopoietic stem cell transplantation (HSCT) develops a disseminated vesicular rash and is non-compliant with medications, it is important to consider the possibility of viral infections, such as herpes simplex virus (HSV) or varicella-zoster virus (VZV) reactivation. In such cases, contact precautions should be implemented to prevent the spread of the virus to other individuals.  The specific contact precaution that should be used in this scenario is Contact Precautions with Airborne Precautions. This means that in addition to standard contact precautions (e.g., gloves and gown when entering the patient's room), airborne precautions should also be followed.  Airborne precautions include:  1. Wearing a fit-tested N95 or higher-level respirator when entering the patient's room.  2. Placing the patient in an airborne infection isolation room (AIIR) that has negative air pressure and high-efficiency particulate air (HEPA) filtration.  3. Limiting the movement of the patient outside of the AIIR and ensuring that they wear a mask during transport.  4. Educating healthcare workers and visitors about proper hand hygiene and the use of personal protective equipment (PPE) when entering the patient's room.  By implementing these precautions, the risk of transmitting the virus to healthcare workers, other patients, and visitors can be minimized.  It is important to consult with the healthcare team and infection control specialists in the specific healthcare facility for guidance on the appropriate precautions based on the patient's condition and the local infection control policies. |
| I8 | In the case of a patient undergoing hematopoietic stem cell transplantation (HSCT) who has tested positive for respiratory syncytial virus (RSV) and is experiencing symptoms such as fever, dry cough, and shortness of breath with bilateral ground glass pattern on chest CT scan, the potential antiviral treatment that could be offered is ribavirin.  Ribavirin is an antiviral medication that has activity against RSV. It can be administered via inhalation or intravenously, depending on the severity of the infection and the patient's condition. In the case of severe RSV infection in immunocompromised patients, intravenous ribavirin is often considered.  However, it's important to note that the use of ribavirin for RSV infection in immunocompromised patients, including HSCT recipients, is still a matter of debate and is not universally recommended. The decision to use ribavirin or other antiviral agents in this situation should be made on a case-by-case basis, considering factors such as the severity of the infection, the patient's overall condition, and the potential benefits and risks of the treatment.  The final treatment decision and dosing regimen should be made by the patient's healthcare team, including the transplant physician or infectious disease specialist, based on the patient's specific circumstances and the latest clinical guidelines. | In the scenario you've described, where a patient undergoing hematopoietic stem cell transplantation (HSCT) is diagnosed with respiratory syncytial virus (RSV) infection and presents with symptoms such as fever, dry cough, and shortness of breath, the potential treatment option that could be offered to the patient is ribavirin.  Ribavirin is an antiviral medication with broad-spectrum activity against several RNA and DNA viruses, including RSV. It is available in various formulations, including oral, intravenous, and inhalation formulations. For the treatment of RSV infection in immunocompromised patients, particularly those undergoing HSCT, inhalation of ribavirin is commonly used.  The inhalation form of ribavirin involves the use of a small particle aerosol generator (SPAG) or a ventilator-driven nebulizer to deliver the medication directly to the respiratory tract. This targeted delivery helps achieve higher concentrations of ribavirin in the lungs, where RSV infection primarily occurs.  It's important to note that the use of ribavirin in HSCT patients with RSV infection may vary based on several factors, including the patient's overall clinical condition, the severity of the infection, and the specific protocols followed by the transplant center. The decision to offer ribavirin or any other antiviral medication should be made in consultation with the patient's healthcare team, who will consider various factors and individualize the treatment approach based on the patient's specific needs and circumstances. | For a patient undergoing hematopoietic stem cell transplantation (HSCT) who has tested positive for respiratory syncytial virus (RSV) and is experiencing symptoms such as fever, dry cough, and shortness of breath with bilateral ground glass pattern on chest CT scan, the drug that could potentially be offered is ribavirin.  Ribavirin is an antiviral medication that has activity against a broad spectrum of RNA and DNA viruses, including RSV. It can be used for the treatment of severe RSV infections in immunocompromised patients, such as those undergoing HSCT.  The administration of ribavirin for RSV infection in HSCT patients is typically done through aerosolized or inhaled forms. The specific dosage and treatment regimen will be determined by the healthcare team based on the patient's condition, viral load, and any underlying complications. In some cases, ribavirin may also be used in combination with other antiviral agents or immunomodulatory therapies.  It's important to note that the decision to use ribavirin and the specific treatment approach may vary depending on factors such as the patient's overall clinical status, the severity of RSV infection, and the protocols followed by the transplant center or infectious disease specialists involved in the patient's care. Close monitoring and collaboration with the healthcare team are crucial in determining the most appropriate treatment plan for the individual patient. |
| I9 | Monitoring adenovirus on fecal samples is not routinely recommended in adults undergoing allogeneic hematopoietic stem cell transplantation (HSCT). Adenovirus infections are more commonly associated with respiratory and ocular symptoms rather than gastrointestinal symptoms in this patient population.  In allogeneic HSCT recipients, monitoring for adenovirus infection typically focuses on respiratory samples (such as nasopharyngeal swabs) or blood samples. These are more relevant for detecting adenovirus-associated respiratory or disseminated infections, which can be clinically significant and require specific management strategies.  If an allogeneic HSCT recipient presents with gastrointestinal symptoms and adenovirus infection is suspected, other diagnostic methods, such as testing stool samples for adenovirus, may be considered. However, this would generally be based on specific clinical circumstances and the discretion of the treating healthcare team.  It's important to note that the management of adenovirus infection in HSCT recipients is complex and should be guided by the expertise of the healthcare team, which may include infectious disease specialists or transplant specialists. They will consider the individual patient's clinical presentation, risk factors, and specific institutional guidelines to determine the most appropriate monitoring and management strategies for adenovirus infection in the context of allogeneic HSCT. | Monitoring adenovirus on fecal samples is not routinely recommended for all adults undergoing allogeneic hematopoietic stem cell transplantation (HSCT). However, the need for monitoring adenovirus infection may vary depending on specific factors such as the patient's medical history, clinical presentation, and the institutional protocols and guidelines followed by the healthcare facility.  Adenovirus infections can occur in HSCT recipients, and they can present with a range of symptoms, including gastrointestinal manifestations such as diarrhea. In cases where adenovirus infection is suspected based on clinical symptoms, monitoring of fecal samples for adenovirus may be considered to aid in diagnosis and guide appropriate management.  The decision to monitor adenovirus on fecal samples should be made in consultation with the healthcare team, including infectious disease specialists and hematologists or oncologists experienced in HSCT. They will assess the individual patient's risk factors, clinical presentation, and overall transplant protocol to determine the appropriate monitoring and diagnostic measures required.  It's important to note that the specific recommendations may vary between institutions, and individual patient circumstances play a significant role in determining the need for monitoring adenovirus infection in fecal samples during allogeneic HSCT. | Monitoring adenovirus in fecal samples is recommended in adults undergoing allogeneic hematopoietic stem cell transplantation (HSCT). Adenovirus is a common cause of viral infections in immunocompromised individuals, including HSCT recipients. It can cause a range of clinical manifestations, including gastrointestinal symptoms.  Monitoring for adenovirus in fecal samples allows for early detection of viral shedding and the identification of patients at risk of developing adenovirus-related complications, such as disseminated infection or graft-versus-host disease (GVHD).  Regular monitoring of fecal samples for adenovirus can help guide clinical management, including implementing appropriate infection control measures, adjusting immunosuppressive therapy, and initiating antiviral treatment if necessary. The frequency and duration of monitoring may vary depending on institutional protocols and the patient's specific risk factors and clinical course.  It is important to consult with the healthcare team, including infectious disease specialists and transplant physicians, who can provide specific guidelines and recommendations for monitoring adenovirus and other viral pathogens in adults undergoing allogeneic HSCT. They will consider the individual patient's risk factors, the local epidemiology, and institutional practices to determine the appropriate monitoring strategy. |
| I10 | If a patient undergoing hematopoietic stem cell transplantation (HSCT) develops hemorrhagic cystitis, and the urine analysis is negative for BK virus, another virus that could be a potential causative agent is adenovirus.  Adenovirus can cause hemorrhagic cystitis in HSCT recipients, leading to symptoms such as blood in the urine, bladder pain, and urinary frequency. Adenovirus-associated hemorrhagic cystitis is more commonly seen in pediatric patients but can also occur in adults.  To confirm the presence of adenovirus infection, further diagnostic tests such as polymerase chain reaction (PCR) or viral culture may be performed on urine samples.  The treatment approach for adenovirus-associated hemorrhagic cystitis in HSCT patients is often focused on supportive care and antiviral therapy. Some potential treatment options include:  1. Cidofovir: Cidofovir is an antiviral medication that has activity against adenovirus. It can be administered intravenously and is sometimes used in the treatment of adenovirus infections, including hemorrhagic cystitis. However, its use may be associated with potential kidney toxicity, and careful monitoring of renal function is necessary.  2. Brincidofovir: Brincidofovir is an investigational antiviral drug that has shown activity against various DNA viruses, including adenovirus. It has a different toxicity profile compared to cidofovir and may be considered in certain cases. However, its use may be limited to compassionate use or in the context of clinical trials.  3. Supportive Care: Supportive measures to manage symptoms and minimize complications of hemorrhagic cystitis can include pain management, hydration, bladder irrigation, and blood transfusions if needed.  The specific treatment approach will depend on various factors, including the patient's overall condition, severity of symptoms, availability of antiviral agents, and the expertise of the healthcare team. Consultation with an infectious disease specialist and/or a transplant physician is essential to guide the management and treatment of adenovirus-associated hemorrhagic cystitis in HSCT patients. | In a patient undergoing hematopoietic stem cell transplantation (HSCT) who develops hemorrhagic cystitis and tests negative for BK virus, another potential causative virus could be Adenovirus.  Adenovirus is known to cause hemorrhagic cystitis in HSCT recipients. It can lead to inflammation of the bladder, resulting in symptoms such as hematuria (blood in urine), urinary frequency, urgency, and dysuria.  To confirm the presence of Adenovirus infection, specific diagnostic tests such as polymerase chain reaction (PCR) or viral culture may be performed on urine samples. These tests can detect the presence of Adenovirus DNA or isolate the virus for identification.  The treatment of Adenovirus-associated hemorrhagic cystitis in HSCT patients can be challenging. Several therapeutic approaches have been used, including antiviral agents, immunomodulatory drugs, and supportive care measures. However, no specific antiviral drug has been established as the standard treatment for Adenovirus infection.  Some potential treatment options that have been utilized in clinical practice include:  1. Cidofovir: Cidofovir is a broad-spectrum antiviral medication that has shown activity against Adenovirus in some studies. It can be administered intravenously, although its use may be associated with potential renal toxicity.  2. Brincidofovir: Brincidofovir is an investigational antiviral drug that has shown activity against Adenovirus. It has the advantage of a favorable renal safety profile compared to cidofovir. However, it is still being evaluated in clinical trials and not yet widely available.  3. Immunomodulatory Therapy: In some cases, immunomodulatory agents such as intravenous immunoglobulin (IVIG) or adoptive T-cell therapy may be considered to boost the immune response against the virus.  Supportive care measures, including adequate hydration and pain management, are also important in managing hemorrhagic cystitis symptoms.  It's crucial for the patient to be closely monitored and for treatment decisions to be made in consultation with the healthcare team, considering the patient's overall clinical status, potential side effects of treatment options, and available resources. | In a patient undergoing hematopoietic stem cell transplantation (HSCT) who develops hemorrhagic cystitis with a negative urine analysis for BK virus, another virus that could potentially be a causative agent is adenovirus.  Adenovirus is a DNA virus that can cause a range of clinical manifestations, including respiratory infections, gastroenteritis, and hemorrhagic cystitis. Adenovirus-associated hemorrhagic cystitis is a well-known complication in HSCT recipients.  To confirm adenovirus as the causative agent, specific laboratory tests, such as polymerase chain reaction (PCR) or viral culture, may be performed on urine or other clinical samples.  Treatment options for adenovirus-associated hemorrhagic cystitis in HSCT patients may include:  1. Supportive Care: Supportive care is an essential component of managing adenovirus-associated hemorrhagic cystitis. This involves measures to maintain hydration, control pain, and manage bleeding. Adequate fluid intake and pain management are important for symptomatic relief and prevention of complications.  2. Antiviral Therapy: Cidofovir is an antiviral agent that has activity against adenovirus. It can be considered as a treatment option for severe or refractory cases of adenovirus-associated hemorrhagic cystitis. Cidofovir is administered intravenously and should be used under the guidance of infectious disease specialists or a transplant physician, as it can have nephrotoxic effects.  3. Immunotherapy: In some cases, immunotherapy strategies, such as adoptive immunotherapy with virus-specific T cells or infusion of donor lymphocytes, may be considered. These approaches aim to enhance the immune response against the virus and help control the infection.  The specific treatment approach for adenovirus-associated hemorrhagic cystitis will depend on factors such as the severity of symptoms, the extent of viral replication, the patient's overall clinical condition, and the expertise of the healthcare team managing the case. Prompt diagnosis, close monitoring, and collaboration with infectious disease specialists or transplant physicians are crucial for the appropriate management of adenovirus-associated hemorrhagic cystitis. |
| I11 | The management of asymptomatic BK viruria in hematopoietic stem cell transplantation (HSCT) recipients can vary depending on several factors, including the patient's overall clinical condition, the degree of viral replication, and the potential risk for developing complications such as hemorrhagic cystitis or nephropathy.  In general, asymptomatic BK viruria in HSCT recipients does not require specific antiviral treatment. Most cases of BK viruria in the absence of symptoms are self-limiting and resolve spontaneously without causing significant complications.  However, close monitoring of the patient's viral load through regular urine testing is recommended. If the viral load continues to rise or there are signs of progressive renal dysfunction, intervention may be necessary. This may include reducing immunosuppressive medications to help restore the immune response against the BK virus, promoting viral clearance.  In some cases, antiviral therapy with medications such as cidofovir or leflunomide may be considered for HSCT recipients with persistent BK viruria and evidence of progressive renal dysfunction or other significant complications. The decision to initiate antiviral treatment should be made on an individual basis, taking into account the potential benefits, risks, and specific patient factors.  It is important to consult with the patient's healthcare team, including infectious disease specialists and transplant physicians, to assess the specific situation and determine the appropriate management strategy for asymptomatic BK viruria in HSCT recipients. They will consider the patient's overall clinical condition, immunosuppressive regimen, and any other relevant factors to make individualized treatment decisions. | The management of asymptomatic BK viruria in hematopoietic stem cell transplant (HSCT) recipients is a subject of debate and depends on several factors. BK virus is a common cause of viral infections in HSCT recipients and can cause complications such as hemorrhagic cystitis or nephropathy.  In the absence of symptoms, the approach to asymptomatic BK viruria may vary. Some experts suggest monitoring the viral load and observing the patient for any signs or symptoms of BK virus-associated complications, such as persistent viruria or an increase in viral load. This approach is based on the understanding that asymptomatic BK viruria often resolves spontaneously without the need for specific treatment.  However, in certain situations, such as high viral loads or persistent viruria, intervention may be considered to prevent progression to BK virus-associated complications. Treatment options for symptomatic or high-risk cases of BK viruria can include reduction of immunosuppression, antiviral medications (such as cidofovir or leflunomide), and supportive measures (e.g., hydration, bladder irrigation).  The decision to initiate treatment for asymptomatic BK viruria should be made on an individual basis, considering factors such as the patient's overall clinical condition, the presence of other risk factors, the persistence or increase in viral load, and the potential benefits and risks of intervention. It is important to consult with the healthcare team, including infectious disease specialists and transplant physicians, to determine the most appropriate management strategy for asymptomatic BK viruria in HSCT recipients. | The treatment of asymptomatic BK viruria in hematopoietic stem cell transplant (HSCT) recipients is a topic of debate and depends on various factors. BK virus (BKV) is a common polyomavirus that can cause complications, including hemorrhagic cystitis, in immunocompromised individuals such as HSCT recipients.  In the absence of symptoms, the decision to treat asymptomatic BK viruria should be based on several factors, including the patient's immune status, the presence of other risk factors, the level of BK viral load, and the potential impact on transplant outcomes.  Currently, there is no specific antiviral therapy available for BKV. Management strategies for asymptomatic BK viruria often involve close monitoring of the viral load and the patient's clinical course. Regular urine testing and periodic imaging studies (e.g., cystoscopy) may be performed to assess the progression of the infection and the development of symptomatic complications.  If the BK viral load continues to rise or if the patient develops symptoms such as hematuria or bladder dysfunction, treatment options may be considered. Reduction of immunosuppression is often the first step, as it helps restore immune control over the virus. In severe cases or when conservative measures are not effective, antiviral medications such as cidofovir or leflunomide may be considered, although their efficacy in BKV management is still under investigation.  The decision to treat asymptomatic BK viruria in HSCT recipients should be made on a case-by-case basis, taking into consideration the patient's overall condition, the risk of progression to symptomatic disease, and the potential benefits and risks of intervention. It is important to consult with the patient's healthcare team, including infectious disease specialists and transplant physicians, who can provide personalized recommendations based on the individual patient's circumstances and the institution's protocols. |
| I12 | Third-party virus-specific T lymphocytes (VSTs) refer to T cells that are obtained from a donor other than the stem cell transplant donor or the transplant recipient. These T cells are specifically selected and expanded to target and control viral infections that may occur after hematopoietic stem cell transplantation (HSCT). Third-party VSTs are an emerging immunotherapeutic approach used to treat or prevent viral infections in HSCT patients.  Here's how third-party VSTs are utilized in HSCT:  1. Donor Selection: Donors with a pre-existing immune response against specific viruses, such as cytomegalovirus (CMV), Epstein-Barr virus (EBV), adenovirus, or others, are identified. These donors have T cells in their immune system that are specifically primed to recognize and target these viruses.  2. T Cell Isolation: T cells specific to the targeted virus are isolated from the donor's peripheral blood or leukapheresis product.  3. Ex Vivo Expansion: The isolated T cells are then expanded in the laboratory using various techniques, such as stimulation with viral antigens or peptide libraries. This expansion process increases the number of virus-specific T cells available for infusion.  4. Infusion: The expanded virus-specific T cells are infused into the HSCT recipient. The transferred T cells can recognize and kill virus-infected cells, thus controlling viral replication and preventing or treating viral infections.  The advantage of using third-party VSTs is that they provide an immediate source of virus-specific T cells, even if the original stem cell donor lacks a suitable T cell response against the specific virus. This approach offers a timely and effective treatment option to control viral infections in HSCT patients, especially when other therapies, such as antiviral medications, have not been successful or are not available.  It's important to note that the use of third-party VSTs is still an investigational therapy, and its specific applications, protocols, and outcomes may vary depending on the institution and ongoing research. The decision to utilize third-party VSTs should be made in consultation with the patient's transplant team and based on the individual patient's condition and viral infection status. | Third-party virus-specific T lymphocytes (VSTs) are a type of immunotherapy used in patients undergoing hematopoietic stem cell transplantation (HSCT). VSTs are specialized T cells that have been expanded or selected from healthy donors and specifically target viruses that commonly cause complications in transplant recipients, such as cytomegalovirus (CMV), Epstein-Barr virus (EBV), and adenovirus.  Here's how third-party VSTs work and their significance in HSCT:  1. Preparation: Third-party VSTs are prepared from the blood of healthy donors who have previously been exposed to and developed an immune response against the specific viruses of interest (e.g., CMV, EBV). The donor's T cells are isolated and expanded in the laboratory to obtain a sufficient number of virus-specific T cells.  2. Specificity: These expanded T cells are selected or engineered to specifically recognize and target viral antigens expressed by the viruses of interest. This specificity enables the VSTs to recognize and eliminate virus-infected cells while sparing healthy cells.  3. Infusion: Once the VSTs have been prepared, they are infused into the HSCT recipient to provide immediate antiviral immunity against the targeted viruses. The infused VSTs expand and mount an immune response against the viruses, helping to control or eliminate viral infections and prevent related complications.  The use of third-party VSTs offers several advantages:  - Immediate Antiviral Activity: Third-party VSTs provide immediate antiviral activity, even before the patient's immune system has fully recovered after HSCT. This helps reduce the risk and severity of viral infections, which can be particularly problematic in the early post-transplant period when the immune system is weakened.  - Broad Viral Coverage: Third-party VSTs can target multiple viruses simultaneously, including CMV, EBV, and adenovirus, which are commonly associated with complications in HSCT recipients. This broad coverage improves the overall antiviral protection provided by VST therapy.  - Off-the-Shelf Availability: Third-party VSTs can be prepared in advance from healthy donor sources, allowing for rapid availability and immediate treatment when needed. This eliminates the need for custom manufacturing or finding a matched donor for every patient.  - Reduced Risk of Graft-versus-Host Disease (GVHD): Third-party VSTs are selectively enriched for virus-specific T cells, reducing the risk of GVHD compared to conventional donor lymphocyte infusions that contain a mixture of T cell subsets.  The use of third-party VSTs is considered an effective strategy to prevent and treat viral infections in HSCT recipients. However, the specific protocols, dosing, and timing of VST therapy may vary depending on the patient's individual condition and the recommendations of the healthcare team. | Third-party virus-specific T lymphocytes (VSTs) are a type of cellular therapy used in patients undergoing hematopoietic stem cell transplantation (HSCT). VSTs are immune cells that are specifically selected or engineered to target and combat certain viral infections that commonly occur after HSCT.  Here's how third-party VSTs work and their role in HSCT:  1. Selection or Engineering: Third-party VSTs are typically derived from healthy donors who possess a specific immune response against viruses like cytomegalovirus (CMV), Epstein-Barr virus (EBV), adenovirus, or others. These donor-derived VSTs are then processed and prepared for infusion.  2. Virus-Specific Immunity: VSTs are enriched for T lymphocytes that specifically recognize and target viral antigens associated with a particular virus. They have been primed to recognize and attack infected cells expressing these viral antigens.  3. Infusion and Viral Clearance: The prepared VSTs are then infused into the patient undergoing HSCT who has developed or is at risk of developing viral infections. Once infused, the VSTs recognize and eliminate the virus-infected cells, helping to control or clear the viral infection.  4. Adaptability and Safety: Third-party VSTs are an "off-the-shelf" therapy, meaning they can be prepared in advance from a healthy donor's cells and administered to different patients. This approach allows for quick availability and flexibility. To minimize the risk of graft-versus-host disease (GVHD), the VSTs are often selected for specific viral reactivity while minimizing potential alloreactivity against the patient's healthy tissues.  Third-party VSTs have shown promising results in controlling or preventing viral infections after HSCT, especially in patients who are at high risk or have persistent or drug-resistant viral infections. These cellular therapies have the potential to improve patient outcomes by reducing the impact of viral infections, which can be a significant cause of morbidity and mortality in the HSCT setting.  It's important to note that the use of third-party VSTs should be conducted under the supervision of healthcare professionals with expertise in cellular therapies and HSCT. The selection of appropriate VSTs and the determination of timing and dosage will depend on the individual patient's condition, viral infection, and other factors. |
| I13 | In the case of a patient undergoing hematopoietic stem cell transplantation (HSCT) who develops fever, pneumonia, and blood cultures growing gram-positive bacteria, one intravenous drug that should be avoided for treating the pneumonia is vancomycin.  The reason for avoiding vancomycin is the potential risk of causing or exacerbating a condition known as vancomycin-associated neutropenia. Neutropenia is a condition characterized by low levels of neutrophils, which are an important type of white blood cell involved in fighting off infections. In the context of HSCT, patients are already at an increased risk of developing neutropenia due to the transplantation procedure and the underlying immunosuppression.  Vancomycin, although effective against many gram-positive bacteria, has been associated with the development of neutropenia, particularly in patients with underlying risk factors or prolonged use. This can further compromise the patient's immune system and increase the risk of infections or other complications.  Instead of vancomycin, alternative intravenous antibiotics that have coverage against gram-positive bacteria, such as methicillin-resistant Staphylococcus aureus (MRSA), can be considered. These alternatives may include drugs such as linezolid or daptomycin, depending on the specific susceptibility profile of the isolated bacteria and the clinical judgment of the healthcare team.  It's important to note that the specific antibiotic regimen and treatment decisions should be made in consultation with infectious disease specialists or a multidisciplinary team experienced in managing infections in HSCT patients. The choice of antibiotics may depend on factors such as the patient's clinical condition, local resistance patterns, and the results of susceptibility testing. | In a patient undergoing hematopoietic stem cell transplantation (HSCT) who develops fever, pneumonia, and blood cultures that grow gram-positive bacteria, there is a specific intravenous drug that should be avoided in treating the pneumonia: Trimethoprim-sulfamethoxazole (TMP-SMX).  TMP-SMX, also known as Bactrim or Septra, is an antibiotic commonly used to treat various bacterial infections, including those caused by gram-positive bacteria. However, it is important to note that TMP-SMX has limited activity against certain gram-positive bacteria, specifically those that are methicillin-resistant Staphylococcus aureus (MRSA).  In the context of HSCT and pneumonia, MRSA can be a significant concern, particularly in healthcare-associated infections. MRSA pneumonia is associated with increased morbidity and mortality in immunocompromised patients.  Therefore, when treating pneumonia in a HSCT patient with gram-positive bacteria, including suspected or known MRSA, it is advisable to avoid TMP-SMX as monotherapy due to its limited efficacy against MRSA. Instead, alternative treatment options that have activity against MRSA should be considered, such as:  1. Vancomycin: Vancomycin is a commonly used intravenous antibiotic with good activity against MRSA. It is often considered the drug of choice for the treatment of MRSA infections, including pneumonia.  2. Linezolid: Linezolid is another intravenous antibiotic that has activity against MRSA. It is often used as an alternative to vancomycin and has been effective in treating MRSA pneumonia.  3. Daptomycin: Daptomycin is a lipopeptide antibiotic with activity against MRSA. It is another option that may be considered for the treatment of MRSA pneumonia.  The choice of specific antibiotic therapy will depend on factors such as the severity of the infection, local susceptibility patterns, and the patient's individual characteristics. The decision should be made in consultation with infectious disease specialists or the healthcare team managing the patient's HSCT. | In the scenario you described, if the patient undergoing hematopoietic stem cell transplantation (HSCT) develops pneumonia and blood cultures grow gram-positive bacteria, the intravenous drug that should be avoided for treating the pneumonia is trimethoprim/sulfamethoxazole (TMP/SMX), also known as Bactrim or Septra.  TMP/SMX is a combination antibiotic that is effective against various bacterial infections. However, it is known to have limited activity against gram-positive bacteria, particularly methicillin-resistant Staphylococcus aureus (MRSA). MRSA is a common gram-positive pathogen associated with healthcare-associated pneumonia and can be prevalent in hospitals or healthcare settings.  In the case of suspected or confirmed MRSA pneumonia, alternative antibiotics with activity against MRSA should be used. Some commonly used intravenous antibiotics for treating MRSA pneumonia include:  1. Vancomycin: Vancomycin is a glycopeptide antibiotic that is effective against MRSA. It is commonly used for severe gram-positive infections, including pneumonia.  2. Linezolid: Linezolid is an oxazolidinone antibiotic with activity against MRSA. It can be administered intravenously and is often used as an alternative to vancomycin.  3. Daptomycin: Daptomycin is a lipopeptide antibiotic that has activity against MRSA and other gram-positive bacteria. It is an intravenous antibiotic option for treating MRSA pneumonia.  It's important to note that the choice of antibiotic for treating pneumonia will depend on factors such as the specific bacteria identified, susceptibility testing results, the severity of infection, and the patient's overall clinical condition. The decision regarding the most appropriate antibiotic should be made in consultation with an infectious disease specialist or the healthcare team managing the patient's HSCT and pneumonia. |
| I14 | In the scenario you described, a 56-year-old patient undergoing hematopoietic stem cell transplantation (HSCT) has tested positive for human herpesvirus 6 (HHV-6) with a low viral load in two separate samples, despite being asymptomatic. There are a few potential explanations for this situation:  1. Latent Infection Reactivation: HHV-6 is known to establish a latent infection in the body, particularly in immune cells. During periods of immunosuppression, such as in the context of HSCT, latent infections can sometimes reactivate, resulting in detectable viral DNA in the bloodstream. Reactivation of HHV-6 can occur without causing symptoms or clinical manifestations, especially when the viral load is low.  2. Transient Viremia: It is possible that the detected HHV-6 viral DNA represents transient viremia, where the virus is temporarily circulating in the blood at low levels. Transient viremia can occur even in the absence of active infection or symptoms, and it may resolve spontaneously without causing clinical consequences.  3. False-Positive Result: While less likely, there is a possibility of a false-positive result in the laboratory testing. Repeat testing or alternative diagnostic methods can be considered to confirm the presence of HHV-6 infection.  It's important to note that HHV-6 is a common virus, and many individuals have been exposed to it during childhood. Asymptomatic HHV-6 infection or low-level viral DNA detection is not uncommon, especially in immunocompromised individuals such as HSCT recipients. Monitoring the patient's clinical condition, including the development of symptoms, is crucial in determining if any intervention or treatment is required.  Close communication between the healthcare team, infectious disease specialists, and the patient is essential to properly interpret the test results and make informed decisions regarding further monitoring or treatment, if necessary. | In the given scenario, a 56-year-old patient undergoing hematopoietic stem cell transplantation (HSCT) has tested positive for human herpesvirus 6 (HHV-6) with a low viral load, but the patient is asymptomatic. Several factors could explain this situation:  1. HHV-6 Reactivation: HHV-6 is known to establish latency in the body after primary infection, typically occurring during childhood. During periods of immunosuppression, such as HSCT, HHV-6 can reactivate and be detected in the blood, leading to a positive test result. Asymptomatic HHV-6 reactivation is not uncommon in HSCT recipients, and it may not necessarily indicate clinical disease or symptoms.  2. Immune Recovery: The patient's asymptomatic status could be attributed to an effective immune response, allowing the immune system to control the viral replication and prevent the development of clinical symptoms. As the patient's immune system recovers following HSCT, it can successfully keep the viral load low and limit HHV-6-related complications.  3. Prophylactic or Preemptive Antiviral Treatment: The patient may be receiving prophylactic or preemptive antiviral treatment against HHV-6. In some cases, antiviral medications such as ganciclovir or foscarnet may be administered to prevent HHV-6 reactivation or to suppress viral replication. This proactive approach can help maintain a low viral load and reduce the likelihood of symptomatic infection.  It's important to note that HHV-6 reactivation in HSCT recipients is common, and its clinical significance varies. In some cases, HHV-6 reactivation can be associated with complications such as encephalitis or graft dysfunction. Close monitoring of the patient's clinical condition, along with regular viral load monitoring, can help assess the need for further interventions or adjustments in antiviral management.  The interpretation of HHV-6 test results and the decision regarding management should be made in consultation with the healthcare team, including infectious disease specialists or transplant physicians, who can evaluate the patient's individual situation and determine the most appropriate course of action. | In a 56-year-old patient undergoing hematopoietic stem cell transplantation (HSCT) who tests positive for human herpesvirus 6 (HHV-6) with a low viral load, but is asymptomatic, there are several possible explanations for this situation:  1. Latent Infection Reactivation: HHV-6 is a common herpesvirus that can establish a latent infection in the body after primary infection. In some cases, the immunosuppression associated with HSCT can lead to the reactivation of latent HHV-6. The low viral load might indicate an early or mild reactivation that has not yet resulted in symptomatic disease.  2. Transient Viremia: It's possible that the positive HHV-6 test with a low viral load represents a transient viremic phase. Viral DNA can be detected in the blood intermittently during the course of an infection, even in the absence of clinical symptoms. In this case, the patient may clear the virus without developing any significant symptoms or complications.  3. False Positive Test Result: Although less likely, false positive test results can occur. Laboratory errors or cross-reactivity with other viruses could potentially lead to an incorrect positive result. Confirming the result with a second sample is a standard practice to minimize the possibility of false positives.  4. Early Infection: If the patient recently acquired HHV-6, the low viral load and lack of symptoms could indicate an early stage of infection. It may take some time for the viral load to increase and for symptoms to develop, if they do at all.  It's important to note that monitoring HHV-6 levels and clinical symptoms over time is essential to assess the significance of the infection. Close observation and follow-up by the healthcare team are necessary to detect any changes in viral load or development of symptoms. If the viral load increases or symptoms emerge, further evaluation and potential intervention may be warranted.  The specific interpretation and management of HHV-6 infection in HSCT patients should be guided by the expertise of the patient's healthcare team, including infectious disease specialists or hematologists with experience in transplant-related infections. |
| I15 | Rectal swab screening can play a role in certain cases before allogeneic hematopoietic stem cell transplantation (HSCT), particularly for the detection of multidrug-resistant organisms (MDROs) and specific infections. However, the decision to perform rectal swab screening should be based on various factors, including local guidelines, institutional practices, patient risk factors, and the prevalence of MDROs in the healthcare setting.  Here are some situations where rectal swab screening may be considered:  1. Detection of MDROs: Rectal swab screening can help identify MDRO colonization, such as methicillin-resistant Staphylococcus aureus (MRSA), vancomycin-resistant Enterococcus (VRE), or extended-spectrum beta-lactamase (ESBL)-producing organisms. Identifying colonization with these resistant bacteria before HSCT can guide infection control measures and inform antibiotic prophylaxis or treatment choices.  2. Control of outbreaks: In settings where there is a known or suspected outbreak of a specific infection, rectal swab screening can be used to identify carriers and implement appropriate infection control measures to prevent transmission.  3. High-risk patients: In certain cases, such as patients with a history of previous MDRO colonization, prolonged hospitalization, or prior exposure to healthcare facilities, rectal swab screening may be considered to identify potential colonization and guide preventive measures.  It is important to note that the necessity and extent of rectal swab screening may vary among healthcare institutions and different transplant centers. Local guidelines and protocols, as well as consultation with infectious disease specialists or the transplant team, should be followed to determine the appropriate use of rectal swab screening before allogeneic HSCT. The screening strategy should be tailored to the specific needs and risks of individual patients, taking into account local epidemiology and infection control practices. | Rectal swab screening before allogeneic hematopoietic stem cell transplantation (HSCT) can have a role in certain cases, depending on the specific circumstances and institutional practices. The purpose of rectal swab screening is to detect the presence of multidrug-resistant organisms (MDROs), such as methicillin-resistant Staphylococcus aureus (MRSA) or vancomycin-resistant Enterococcus (VRE), in the gastrointestinal tract.  The decision to perform rectal swab screening is typically based on various factors, including:  1. Institutional guidelines: Different healthcare institutions may have their own protocols and guidelines regarding pre-transplant screening for MDROs. These guidelines may recommend routine screening for certain high-risk populations or under specific conditions.  2. Patient risk factors: Rectal swab screening may be considered in patients with known risk factors for MDRO colonization or infection, such as previous healthcare exposure, previous colonization or infection with MDROs, or prolonged hospital stays.  3. Infection control considerations: Screening for MDROs can help identify patients who may require additional infection control measures, such as isolation precautions, to prevent the transmission of MDROs within the healthcare setting.  4. Tailored treatment: Identifying MDRO colonization before transplantation allows for appropriate treatment and antibiotic prophylaxis strategies to be implemented to reduce the risk of post-transplant infections.  It is important to note that the decision to perform rectal swab screening should be made on an individual basis, taking into account the specific patient characteristics, institutional practices, and local epidemiology. The healthcare team, including infectious disease specialists and transplant physicians, should be consulted to determine the need for rectal swab screening and to guide the implementation of appropriate infection control measures and treatment strategies for HSCT recipients. | Yes, there is a role for rectal swab screening before allogeneic hematopoietic stem cell transplantation (HSCT) in certain cases. The purpose of rectal swab screening is to detect the presence of multidrug-resistant organisms (MDROs) in the gastrointestinal tract of HSCT recipients. This screening helps identify patients who may be at higher risk of developing MDRO-related infections and allows for appropriate infection control measures and targeted antimicrobial therapy.  The specific organisms targeted in rectal swab screening can vary depending on institutional protocols and local epidemiology. Common organisms that may be included in the screening panel are vancomycin-resistant Enterococcus (VRE), extended-spectrum beta-lactamase (ESBL)-producing bacteria, and carbapenem-resistant Enterobacteriaceae (CRE).  The decision to perform rectal swab screening before allogeneic HSCT is often based on various factors, including the patient's risk factors, local epidemiology, and institutional practices. Patients at higher risk of MDRO colonization or infection may include those with a history of prior colonization, previous hospitalizations, exposure to healthcare settings, or recent antibiotic use.  Rectal swab screening can help identify HSCT recipients who may require targeted antimicrobial prophylaxis or isolation precautions to minimize the risk of MDRO-related infections. It allows for appropriate infection control measures to be implemented, such as isolation, cohorting, or decolonization strategies, when necessary.  The decision to perform rectal swab screening and the specific protocols followed should be discussed with the patient's healthcare team, including infectious disease specialists and transplant physicians, who can provide guidance based on the individual patient's risk factors and the local infection control policies. |
| I16 | After allogeneic hematopoietic stem cell transplantation (HSCT), certain vaccines are generally contraindicated during the early post-transplant period. The immune system of HSCT recipients is typically compromised, and the ability to mount an effective immune response may be impaired. Therefore, live attenuated vaccines are generally contraindicated during this time as they carry a risk of causing severe infections in immunocompromised individuals.  The specific live attenuated vaccines contraindicated early after allogeneic HSCT may include:  1. MMR (measles, mumps, rubella) vaccine  2. Varicella (chickenpox) vaccine  3. Oral poliovirus vaccine (OPV)  4. Yellow fever vaccine  5. Bacille Calmette-Guérin (BCG) vaccine  These vaccines contain live, weakened forms of the respective pathogens, and while they are safe and effective in immunocompetent individuals, they can potentially cause severe disease in immunocompromised patients. Therefore, they are generally avoided in the early post-transplant period when the immune system is still recovering.  However, it is important to note that the specific timing and recommendations regarding vaccinations after HSCT can vary depending on several factors, including the type of transplantation, the patient's underlying condition, and the guidelines of the healthcare institution. The decision regarding vaccinations should be made in consultation with the patient's healthcare team, including transplant physicians and infectious disease specialists, who can provide personalized recommendations based on the patient's individual circumstances and immune status. | After allogeneic hematopoietic stem cell transplantation (HSCT), the immune system of the recipient is compromised and may take time to recover. As a result, certain vaccines are generally contraindicated early after HSCT. These include live attenuated vaccines.  Live attenuated vaccines contain weakened or modified live viruses or bacteria that can stimulate an immune response. In individuals with a compromised immune system, such as those who have undergone HSCT, live vaccines can pose a risk of causing severe or disseminated infections. The weakened viruses or bacteria in live vaccines may not be effectively controlled by the weakened immune system, potentially leading to serious complications.  Examples of live attenuated vaccines that are typically contraindicated early after HSCT include:  1. Measles, mumps, rubella (MMR) vaccine  2. Varicella (chickenpox) vaccine  3. Oral polio vaccine (OPV)  4. Yellow fever vaccine  5. Intranasal influenza vaccine (FluMist)  In contrast, inactivated or killed vaccines, such as the injectable influenza vaccine, hepatitis B vaccine, and pneumococcal vaccine, are generally considered safe to administer to HSCT recipients.  The timing and specific recommendations regarding vaccination after HSCT may vary depending on individual factors, such as the type of transplant, the underlying condition, and the patient's immune status. It is important to consult with the healthcare team, including transplant physicians and infectious disease specialists, who can provide personalized guidance on the appropriate timing and types of vaccinations after allogeneic HSCT based on the specific patient's condition and immune recovery. | After allogeneic hematopoietic stem cell transplantation (HSCT), certain live vaccines are generally contraindicated in the early post-transplant period due to the patient's compromised immune system. These vaccines contain live, attenuated viruses that could potentially cause severe or disseminated infections in immunocompromised individuals. The specific contraindicated vaccines can vary depending on institutional guidelines and the patient's clinical condition. However, some commonly contraindicated vaccines early after allogeneic HSCT may include:  1. MMR (measles, mumps, and rubella) vaccine: This vaccine contains live attenuated viruses and is typically contraindicated in the first six to twelve months after HSCT.  2. Varicella (chickenpox) vaccine: The varicella vaccine also contains live attenuated virus and is generally contraindicated early after HSCT.  3. Oral polio vaccine (OPV): OPV is a live attenuated vaccine and is usually contraindicated after HSCT.  4. Yellow fever vaccine: The yellow fever vaccine is a live attenuated vaccine and is generally contraindicated in HSCT recipients due to the risk of severe adverse events.  It is important to consult with the healthcare team, including the transplant physician and infectious disease specialists, to determine the specific vaccination guidelines and contraindications for the individual patient based on their clinical condition, immunosuppression status, and institutional protocols. In some cases, alternative strategies such as vaccination of close contacts (cocooning) or delayed vaccination may be recommended to protect the patient from vaccine-preventable diseases while ensuring their safety. |
| I17 | Based on the information provided, the most probable cause of the skin rash, high-grade fever, and cytopenias (low blood counts) 2 weeks after cord blood transplant is graft failure. Graft failure refers to the failure of the transplanted cord blood cells to properly engraft and establish a functional hematopoietic system.  The likely pathophysiology of graft failure involves several potential factors. Some possible mechanisms include:  1. Graft rejection: The recipient's immune system recognizes the transplanted cells as foreign and mounts an immune response against them, leading to the destruction of the graft.  2. Graft-versus-host disease (GVHD): GVHD occurs when the donor immune cells attack the recipient's tissues. Severe GVHD can impair the ability of the donor cells to establish a functional hematopoietic system.  3. Insufficient stem cell dose: The number of transplanted stem cells may be insufficient to support successful engraftment and hematopoiesis.  4. Conditioning regimen toxicity: The pre-transplant conditioning regimen used to prepare the patient's body for transplantation can cause toxicity to the bone marrow and impair the engraftment of the transplanted cells.  To determine the specific cause and underlying pathophysiology, further evaluation and testing, such as bone marrow aspiration and biopsy, may be necessary. These tests can help assess the degree of engraftment, evaluate for signs of graft rejection or GVHD, and provide insights into the underlying mechanisms contributing to graft failure.  It is crucial to consult with the patient's healthcare team, including transplant physicians and hematologists experienced in cord blood transplantation, who can evaluate the patient's specific situation, perform the necessary tests, and provide appropriate management and treatment options tailored to the individual patient's needs. | In a patient who develops skin rash, high-grade fever, and cytopenias (low blood counts) 2 weeks after cord blood transplant, the most probable cause is graft-versus-host disease (GVHD). GVHD is a common complication following allogeneic transplantation, including cord blood transplant.  GVHD occurs when the transplanted donor immune cells (graft) recognize the recipient's tissues (host) as foreign and mount an immune response against them. This immune response can lead to inflammation and damage to various organs, including the skin, liver, and gastrointestinal tract.  The likely pathophysiology of GVHD involves activation of donor T cells that recognize the host's antigens as foreign. These activated T cells release inflammatory cytokines and cytotoxic molecules, leading to tissue damage and systemic manifestations.  In the given case, the skin rash and fever are common clinical features of GVHD. The cytopenias (low blood counts) can be a result of the immune-mediated destruction of the recipient's bone marrow by the donor immune cells.  To confirm the diagnosis of GVHD, further evaluation may include skin biopsy for histopathological examination, assessment of organ involvement, and consideration of immunosuppressive therapy to control the immune response.  It is crucial to consult with the patient's healthcare team, including transplant physicians and oncologists experienced in HSCT, for accurate diagnosis and appropriate management of GVHD. They will consider the patient's clinical presentation, conduct further investigations, and recommend the most suitable treatment options to control the GVHD and support the patient's recovery. | Based on the provided information, the most probable cause of the skin rash, high-grade fever, and cytopenias (low blood counts) two weeks after cord blood transplant is graft failure or graft rejection. Graft failure refers to the lack of successful engraftment of the transplanted stem cells, resulting in inadequate or insufficient production of healthy blood cells.  The likely pathophysiology of graft failure in this scenario involves the following mechanisms:  1. Graft Rejection: The recipient's immune system recognizes the transplanted cord blood cells as foreign and mounts an immune response against them. This immune response can lead to the destruction of the transplanted cells and hinder their ability to engraft and produce healthy blood cells.  2. Graft-versus-Host Disease (GVHD): GVHD occurs when donor immune cells recognize the recipient's tissues as foreign and attack them. GVHD can result in damage to the bone marrow, impairing the production of healthy blood cells.  Both graft rejection and GVHD can contribute to cytopenias and disrupt the normal functioning of the immune system, leading to symptoms such as rash, fever, and decreased blood cell counts.  It is important to promptly notify the healthcare team about these symptoms and the declining blood counts. Further evaluation and management, including potential interventions such as immunosuppressive therapy or rescue stem cell infusion, may be required to address graft failure and improve hematopoietic recovery. The healthcare team, including transplant physicians and hematologists, will assess the patient's condition, perform additional tests, and determine the most appropriate course of action to manage graft failure or rejection. |
| I18 | In a patient who develops multiple lymphadenopathy (enlarged lymph nodes) a few weeks after receiving hematopoietic stem cell transplantation (HSCT), with a positive Epstein-Barr virus (EBV) in peripheral blood and a high viral load, the patient may be offered treatment with the antiviral drug rituximab.  Rituximab is a monoclonal antibody that specifically targets CD20, a surface protein expressed on B lymphocytes, including those infected with EBV. It is commonly used in the management of post-transplant lymphoproliferative disorder (PTLD), which can occur due to uncontrolled EBV infection after HSCT.  The rationale for using rituximab in this scenario is to selectively deplete the B cells infected with EBV, which can help control the viral replication and reduce the associated lymphoproliferative response. By targeting CD20-positive B cells, rituximab can effectively reduce the EBV viral load and shrink lymphadenopathy in PTLD cases.  The decision to offer rituximab or any other specific treatment will depend on various factors, including the patient's overall condition, the severity of the symptoms, the extent of the lymphadenopathy, and the specific protocols or guidelines followed by the healthcare team. It is essential for the patient to consult their healthcare provider, typically an oncologist or hematologist, who can evaluate the individual case and determine the most appropriate treatment approach based on their expertise and the patient's specific circumstances. | In a patient who develops multiple lymphadenopathies a few weeks after receiving hematopoietic stem cell transplantation (HSCT) and tests positive for Epstein-Barr virus (EBV) with a high viral load in the peripheral blood, the drug typically offered as a treatment option is rituximab.  Rituximab is a monoclonal antibody that specifically targets CD20, a protein expressed on the surface of B cells, including those infected by EBV. By binding to CD20, rituximab helps to eliminate the EBV-infected B cells, reduce viral replication, and control the expansion of infected B cells that may be contributing to the lymphadenopathy.  The treatment approach with rituximab may involve:  1. Administration: Rituximab is usually given intravenously (IV) as an infusion. The specific dosage and treatment schedule depend on various factors, including the patient's overall health, the severity of EBV infection, and the transplant center's protocols.  2. Monitoring: During rituximab therapy, close monitoring of the patient's response to treatment and the EBV viral load is essential. Regular blood tests are conducted to assess the reduction in viral load and monitor the patient's clinical progress.  3. Supportive Care: Supportive care measures, such as hydration, symptomatic relief for lymphadenopathy-related symptoms, and management of potential side effects of rituximab, are provided as necessary.  It's important to note that the management of EBV-related lymphoproliferative disorders or post-transplant lymphoproliferative disorder (PTLD) can involve a multidisciplinary approach, including collaboration between hematologists/oncologists, infectious disease specialists, and transplant physicians. The decision regarding the choice of treatment, including the use of rituximab, is made based on the individual patient's condition, EBV viral load, and the clinical judgment of the healthcare team involved in the patient's care. | In a patient who develops multiple lymphadenopathy (enlarged lymph nodes) a few weeks after receiving hematopoietic stem cell transplantation (HSCT) and is positive for Epstein-Barr virus (EBV) with a high viral load in the peripheral blood, the drug that may be offered is rituximab.  Rituximab is a monoclonal antibody that targets and depletes B cells, including those infected with EBV. It is commonly used in the management of post-transplant lymphoproliferative disorder (PTLD), which can occur as a result of uncontrolled EBV infection in immunocompromised individuals, including HSCT recipients.  The use of rituximab in this context aims to control the expansion of EBV-infected B cells and reduce the risk of PTLD progression. Rituximab can be effective in reducing lymphadenopathy, decreasing viral load, and improving the clinical outcome in patients with EBV-associated PTLD after HSCT.  However, it's important to note that the specific treatment plan and drug selection depend on the patient's individual circumstances, including the severity of the condition, the overall health status, and the recommendations of the treating healthcare team. The decision to offer rituximab or any other treatment will be made based on a comprehensive evaluation of the patient and in consultation with the appropriate specialists, such as hematologists or oncologists, who are experienced in managing post-transplant complications. |
| **Section 4 - noninfectious complication** | | | |
| C1 | Acute graft-versus-host disease (GVHD) is a common complication that can occur after allogeneic hematopoietic stem cell transplantation (HSCT) when the donor's immune cells recognize the recipient's tissues as foreign and mount an immune response against them. The clinical features of acute GVHD can vary in severity and can affect multiple organs. Here are some common clinical features of acute GVHD:  1. Skin involvement: Skin is the most commonly affected organ in acute GVHD. Patients may develop a rash characterized by maculopapular lesions or erythematous patches. The rash typically starts on the palms, soles, face, and upper body and can spread to other parts of the body.  2. Gastrointestinal (GI) symptoms: Acute GVHD can affect the GI tract, leading to symptoms such as nausea, vomiting, diarrhea, abdominal pain, and sometimes gastrointestinal bleeding. Severe cases can result in malabsorption and electrolyte imbalances.  3. Liver involvement: Acute GVHD can cause liver dysfunction, leading to elevated liver enzymes (alanine aminotransferase [ALT], aspartate aminotransferase [AST], and bilirubin). Jaundice may also be present.  4. Mucosal involvement: Mucositis affecting the oral cavity and gastrointestinal tract can occur in acute GVHD. Patients may experience painful mouth sores, difficulty swallowing, and mucosal ulcerations.  5. Fever: Patients with acute GVHD often have a high-grade fever, which is usually a non-infectious fever resulting from the inflammatory response.  The severity of acute GVHD can vary, ranging from mild with involvement limited to one organ to severe with multi-organ dysfunction. It is important to promptly diagnose and manage acute GVHD to prevent progression and potential complications.  Diagnosis is based on clinical evaluation, biopsy of affected organs when necessary, and considering other potential causes of similar symptoms, such as infection or drug reactions.  The management of acute GVHD typically involves immunosuppressive medications to suppress the donor immune response, such as corticosteroids (prednisone) or other immune-modulating drugs. Treatment is individualized based on the severity of GVHD and the patient's overall condition.  It is essential for patients undergoing HSCT to be closely monitored for signs and symptoms of acute GVHD and to report any changes to their healthcare team promptly. | Acute graft-versus-host disease (GVHD) is a common complication that can occur after allogeneic hematopoietic stem cell transplantation (HSCT). It arises when the donor's immune cells (graft) recognize the recipient's tissues (host) as foreign and initiate an immune response. Acute GVHD typically occurs within the first 100 days after transplantation. The clinical features of acute GVHD can vary in severity and can affect multiple organ systems. The common clinical manifestations include:  1. Skin involvement: Skin is frequently affected in acute GVHD. It may present with a maculopapular rash, erythema, or even blistering. The rash often starts on the palms and soles and can spread to other areas of the body.  2. Gastrointestinal symptoms: Acute GVHD can cause gastrointestinal symptoms, such as abdominal pain, nausea, vomiting, and diarrhea. Diarrhea is often profuse and can be bloody.  3. Liver dysfunction: Liver involvement in acute GVHD can manifest as elevated liver enzymes (e.g., alanine aminotransferase, aspartate aminotransferase) and elevated bilirubin levels. Jaundice (yellowing of the skin and eyes) may also be present.  These clinical features of acute GVHD can range from mild to severe and may require specific diagnostic criteria for confirmation. The severity of GVHD is often graded based on the extent and severity of involvement in different organ systems.  It is important to note that acute GVHD shares similarities with other conditions, such as infection and drug reactions, which may require further evaluation and differential diagnosis. Prompt recognition and management of acute GVHD are crucial to minimize complications and optimize outcomes. The specific treatment approach for acute GVHD may involve immunosuppressive medications, such as corticosteroids and other immunomodulatory agents, which are administered to suppress the donor immune response against the recipient's tissues.  Consultation with the patient's healthcare team, including transplant physicians and specialists in managing GVHD, is essential for accurate diagnosis, grading, and appropriate management of acute GVHD based on the individual patient's clinical presentation and specific circumstances. | Acute graft-versus-host disease (aGVHD) is a common complication that can occur after allogeneic hematopoietic stem cell transplantation (HSCT). It arises when donor immune cells recognize the recipient's tissues as foreign and mount an immune response against them. The clinical features of aGVHD can vary depending on the organs involved and the severity of the disease. Here are some common clinical features of aGVHD:  1. Skin involvement: Skin is commonly affected in aGVHD. It typically presents as a maculopapular rash that may be pruritic (itchy) and involve various parts of the body, such as the face, trunk, and extremities. The rash can progress to become erythrodermic (widespread redness) or bullous (blistering).  2. Gastrointestinal symptoms: Gastrointestinal manifestations of aGVHD can include nausea, vomiting, diarrhea, abdominal pain, and gastrointestinal bleeding. The severity of symptoms can range from mild to severe, depending on the extent of involvement.  3. Liver involvement: aGVHD can affect the liver, leading to elevated liver enzymes such as alanine aminotransferase (ALT) and aspartate aminotransferase (AST). Jaundice (yellowing of the skin and eyes) may also be present.  4. Hematologic abnormalities: aGVHD can affect the bone marrow and result in cytopenias (low blood cell counts) such as low white blood cells (leukopenia), low red blood cells (anemia), and low platelets (thrombocytopenia).  Other less common clinical features may include involvement of the lungs, eyes, and mucous membranes.  The diagnosis of aGVHD is typically based on clinical presentation, along with additional supportive evidence such as histopathological examination of affected organs and exclusion of other potential causes of symptoms.  The severity of aGVHD is graded based on established criteria, such as the Glucksberg or the IBMTR (International Bone Marrow Transplant Registry) grading systems. Treatment for aGVHD typically involves immunosuppressive medications to suppress the donor's immune response against the recipient's tissues.  It is important to consult with the patient's healthcare team, including transplant physicians and hematologists experienced in HSCT, who can evaluate the patient's specific situation, confirm the diagnosis, and guide appropriate management and treatment options based on the individual patient's needs. |
| C2 | Based on the information provided, the most likely diagnosis for the patient's gastrointestinal disturbance and diarrhea at day 30 after hematopoietic stem cell transplantation (HSCT) is graft-versus-host disease (GVHD). GVHD is a common complication of HSCT in which the transplanted immune cells recognize the recipient's tissues as foreign and attack them.  The absence of laboratory abnormalities and unremarkable physical exam findings does not rule out GVHD, as these manifestations can be limited to the gastrointestinal tract initially. Gastrointestinal GVHD typically presents with symptoms such as diarrhea, abdominal pain, and gastrointestinal bleeding.  The first-line treatment for gastrointestinal GVHD involves a combination of glucocorticoids, such as prednisone or methylprednisolone, along with supportive measures. Glucocorticoids help suppress the immune response and reduce inflammation. The specific dosage and duration of treatment may vary depending on the severity of GVHD and individual patient factors.  It's important to note that this is a hypothetical scenario, and in actual medical practice, the diagnosis and treatment should be determined by a healthcare professional based on a comprehensive evaluation of the patient's history, physical examination, and relevant diagnostic tests. | Based on the information provided, the most likely diagnosis for the patient's gastrointestinal disturbance and diarrhea at day 30 after hematopoietic stem cell transplantation (HSCT) is graft-versus-host disease (GVHD). GVHD is a common complication following HSCT, where the donor's immune cells recognize the recipient's tissues as foreign and mount an immune attack.  GVHD can affect various organs, including the gastrointestinal tract, causing symptoms such as diarrhea, abdominal pain, and gastrointestinal disturbances. The absence of lab abnormalities and unremarkable physical exam findings are consistent with early-stage or mild GVHD.  The first-line treatment for GVHD typically involves a combination of glucocorticoids, such as prednisone or methylprednisolone, along with supportive care measures. Glucocorticoids work by suppressing the immune response and reducing inflammation. The specific dosing and duration of glucocorticoid therapy may vary based on the severity of GVHD and the patient's individual factors. Other medications, such as calcineurin inhibitors (e.g., cyclosporine, tacrolimus), may also be used in combination with glucocorticoids for more severe cases.  It is important for the patient to be evaluated and managed by a healthcare professional experienced in the management of GVHD, such as a hematologist or a transplant specialist, to ensure appropriate treatment and monitoring of the condition. | Based on the information provided, the most likely diagnosis for the patient at day 30 after hematopoietic stem cell transplantation (HSCT) presenting with gastrointestinal disturbance and diarrhea is graft-versus-host disease (GVHD). GVHD is a common complication of HSCT, occurring when the donor's immune cells recognize the recipient's tissues as foreign and mount an immune response against them.  The symptoms of gastrointestinal GVHD often include diarrhea, abdominal pain, and sometimes nausea and vomiting. It typically occurs within the first few weeks to months after transplantation, which aligns with the patient's timeline of day 30 post-HSCT.  In the absence of reported laboratory abnormalities and unremarkable physical exam, it is important to note that diagnosing GVHD requires further evaluation, such as endoscopic examination or biopsy, to confirm the diagnosis and rule out other potential causes of diarrhea.  The first-line treatment for GVHD involves immunosuppressive therapy to suppress the donor immune cells' attack on the recipient's tissues. The specific treatment regimen may vary based on the severity of GVHD and institutional practices. However, commonly used first-line treatments include:  1. Glucocorticoids (steroids): High-dose systemic corticosteroids, such as prednisone or methylprednisolone, are typically the initial treatment of choice for GVHD. They work by suppressing the immune response and reducing inflammation.  2. Calcineurin inhibitors: Medications like cyclosporine or tacrolimus can be used in combination with steroids or as an alternative if there is a limited response to steroids alone. These drugs also help in suppressing the immune response.  The specific treatment plan and dosages are determined by the patient's overall clinical condition, GVHD severity, and the preferences of the treating physician.  It is important for the patient to consult with their healthcare team promptly to confirm the diagnosis and initiate appropriate treatment. |
| C3 | The success rate of systemic steroids in the treatment of acute graft-versus-host disease (GVHD) can vary depending on several factors, including the severity of the disease, the organs affected, and individual patient characteristics. Overall, systemic steroids are considered the first-line treatment for acute GVHD.  The success rate of systemic steroids in achieving a complete or partial response in acute GVHD ranges from approximately 30% to 60%. However, it's important to note that these numbers can vary based on different studies and populations.  It's worth mentioning that the response to systemic steroids may take some time, and some patients may require additional treatments or modifications to their therapy. If a patient does not respond adequately to steroids or if their GVHD worsens despite treatment, other immunosuppressive medications or therapies may be considered, such as calcineurin inhibitors (cyclosporine or tacrolimus), mycophenolate mofetil, or anti-thymocyte globulin.  The management of GVHD is complex and requires a multidisciplinary approach involving specialists experienced in the treatment of transplant-related complications. Individual patient factors and the specific circumstances of each case can greatly influence the outcomes and success rates of treatment. | The success rate of systemic steroids in treating acute graft-versus-host disease (GVHD) can vary depending on various factors, including the severity of the disease, the organs involved, and individual patient characteristics. Overall, systemic steroids are considered the first-line treatment for acute GVHD and have shown effectiveness in many cases.  Studies have reported response rates ranging from approximately 30% to 70% for systemic steroid treatment in acute GVHD. The response rate refers to the percentage of patients who show improvement or resolution of symptoms with steroid therapy. However, it's important to note that complete response rates, where all symptoms completely resolve, tend to be lower than overall response rates.  The success of systemic steroids in treating acute GVHD also depends on other factors such as the timing of initiation of treatment, the dosage and duration of steroid therapy, and the presence of any other complications or comorbidities. Additionally, the success rate may be influenced by the use of additional immunosuppressive medications or therapies in combination with steroids.  It's essential to consult with a healthcare professional experienced in managing GVHD to discuss the individual prognosis and treatment options for a specific patient's condition. They can provide personalized guidance and considerations based on the patient's unique circumstances. | The success rate of systemic steroids in the treatment of acute graft-versus-host disease (GVHD) can vary depending on several factors, including the severity of GVHD, the organs affected, and individual patient characteristics. Generally, systemic steroids are considered the first-line treatment for acute GVHD.  The response rate to systemic steroids in acute GVHD ranges from approximately 30% to 60% in various studies. However, it's important to note that not all patients respond equally to treatment, and some cases of acute GVHD may be refractory to steroids.  In cases where patients do not respond adequately to initial steroid treatment, additional immunosuppressive therapies may be considered, such as calcineurin inhibitors (e.g., cyclosporine, tacrolimus), mycophenolate mofetil, or other agents targeting specific pathways involved in GVHD.  It's crucial for patients with acute GVHD to be closely monitored by a healthcare professional experienced in managing GVHD, as treatment plans may need to be adjusted based on the individual response and disease progression. |
| C4 | Granulocyte transfusions are not considered a standard of care during aplasia in patients undergoing hematopoietic stem cell transplantation (HSCT). Aplasia refers to a period of severe bone marrow suppression following HSCT, during which the patient experiences a significant reduction or absence of blood cell production.  Granulocytes are a type of white blood cell involved in the body's immune response. Transfusing granulocytes to patients in aplasia has been explored as a potential treatment option to support their immune system until their own bone marrow recovers. However, the use of granulocyte transfusions in this context remains controversial and is not widely accepted as a standard practice.  The main reason for the limited use of granulocyte transfusions is the availability of alternative strategies for managing infectious complications during aplasia, such as prophylactic antimicrobial therapies and the use of growth factors like granulocyte colony-stimulating factor (G-CSF) to stimulate the production of white blood cells.  Moreover, granulocyte transfusions carry certain risks and limitations. They can be associated with adverse reactions, including transfusion-related complications and the potential for alloimmunization (the development of antibodies against transfused granulocytes). Additionally, obtaining an adequate number of granulocytes for transfusion can be challenging, as it requires careful donor selection, leukapheresis procedures, and compatibility considerations.  Ultimately, the decision to use granulocyte transfusions during aplasia in HSCT patients is made on a case-by-case basis, considering factors such as the severity and duration of aplasia, the presence of severe infections, and the availability of alternative treatment options. The treating physician and the transplant team will evaluate the individual patient's condition and make a judgment on the appropriateness of granulocyte transfusion as part of their overall management strategy. | Granulocyte transfusions are not considered a standard of care during aplasia in patients undergoing hematopoietic stem cell transplantation (HSCT). Aplasia refers to a condition where the bone marrow is unable to produce an adequate number of blood cells, including granulocytes (a type of white blood cell).  In HSCT, aplasia typically occurs during the period when the transplanted stem cells are engrafting and the patient's own bone marrow is recovering. This phase is often referred to as the neutropenic phase because of the low levels of neutrophils, a type of granulocyte, in the bloodstream.  While granulocyte transfusions may be considered in specific situations, such as severe bacterial or fungal infections that are not responding to antibiotics, their routine use as a standard of care is not established. This is because granulocyte transfusions have limitations and potential risks, including transfusion-related reactions and the development of alloimmunization.  The primary approach for managing neutropenia and aplasia in HSCT patients involves supportive care measures and administration of growth factors called granulocyte colony-stimulating factors (G-CSF). G-CSF stimulates the bone marrow to produce more white blood cells, including granulocytes, and helps accelerate the recovery of neutrophil counts.  The decision to use granulocyte transfusions or any specific treatment approach during aplasia is typically made by the patient's healthcare team, considering the individual patient's clinical condition, severity of neutropenia, presence of infections, and other factors. It is important for patients to discuss their treatment options and concerns with their healthcare providers to determine the most appropriate course of action. | Granulocyte transfusions are not considered a standard of care during aplasia in patients undergoing hematopoietic stem cell transplantation (HSCT). Aplasia refers to a period of severe bone marrow suppression when the patient's blood cell counts are extremely low, including low levels of granulocytes (a type of white blood cell).  While granulocyte transfusions can be considered in certain situations, such as in patients with severe and prolonged neutropenia (low neutrophil count) who are at high risk of bacterial or fungal infections, their routine use is not considered standard practice.  The mainstay of treatment during aplasia following HSCT includes supportive care measures and the administration of growth factors like granulocyte colony-stimulating factor (G-CSF) or granulocyte-macrophage colony-stimulating factor (GM-CSF). These growth factors help stimulate the production of white blood cells, including granulocytes, by the patient's own bone marrow.  Other supportive measures during aplasia may include:  1. Infection prophylaxis: Administering prophylactic antibiotics, antifungal agents, and antiviral medications to reduce the risk of infections during the period of severe immunosuppression.  2. Blood component transfusions: Transfusions of red blood cells and platelets may be necessary to manage anemia and thrombocytopenia, respectively, which commonly occur during aplasia.  3. Isolation precautions: Implementing strict infection control measures, such as maintaining a sterile environment and limiting visitors, to minimize the risk of infections.  4. Nutritional support: Ensuring adequate nutrition and hydration to support the patient's recovery and immune system function.  The decision to use granulocyte transfusions would depend on the individual patient's clinical condition, the severity and duration of aplasia, and the presence of specific risk factors for severe infections. This decision is typically made on a case-by-case basis by the treating physician in consultation with a hematologist or bone marrow transplant specialist. |
| C5 | The BEDI score is a grading system used to assess the severity of hemorrhagic cystitis, which is characterized by the presence of blood in the urine (hematuria) and visible blood clots. However, it's important to note that there is no specific BEDI score for hemorrhagic cystitis.  The BEDI score, which stands for Bladder Etiology, Duration, International Prostate Symptom Score (IPSS), and Estimated Bladder Capacity, is primarily used to evaluate lower urinary tract symptoms in patients with benign prostatic hyperplasia (BPH) or other bladder conditions. It assesses factors such as the underlying cause, symptom duration, urinary symptoms, and bladder capacity.  In the case of hemorrhagic cystitis, the severity and management are typically evaluated based on clinical presentation, laboratory findings, and the impact on the patient's well-being. The severity of hematuria and clot formation can vary from mild to severe, and the treatment approach may include supportive measures, such as hydration, pain management, and addressing the underlying cause if known.  To determine the appropriate management for a patient with hemorrhagic cystitis, it is crucial to consult with a healthcare professional who can assess the individual case, perform necessary investigations, and provide personalized care based on the patient's specific condition and needs. | I'm sorry, but as of my knowledge cutoff in September 2021, there is no specific scoring system called the BEDI score related to hemorrhagic cystitis. It's possible that a new scoring system or acronym may have been developed since then or that the term "BEDI" refers to a different context.  However, in the context of hemorrhagic cystitis, there are other scoring systems that are commonly used to assess the severity of the condition. One such scoring system is the Common Terminology Criteria for Adverse Events (CTCAE) grading system, which assigns grades based on the severity of hematuria and associated symptoms.  Here is a simplified version of the CTCAE grading system for hematuria:  - Grade 1: Mild hematuria with minimal symptoms.  - Grade 2: Moderate hematuria with intermittent bleeding or clotting requiring intervention.  - Grade 3: Severe hematuria with persistent bleeding or clotting requiring transfusion or invasive intervention.  - Grade 4: Life-threatening or disabling hematuria.  It's important to note that the management of hemorrhagic cystitis goes beyond scoring systems, and the treatment plan should be determined based on the underlying cause, the severity of symptoms, and the overall clinical condition of the patient. Consulting with a healthcare professional experienced in managing such cases is crucial for proper evaluation and appropriate management. | The BEDI score is a scoring system used to assess the severity of hemorrhagic cystitis, a condition characterized by inflammation and bleeding in the bladder. However, the BEDI score specifically refers to assessing hemorrhagic cystitis in the context of hematopoietic stem cell transplantation (HSCT) and is not applicable to other causes of hemorrhagic cystitis.  The BEDI score stands for Bladder Erythema, Denudation, and Ulceration score. It evaluates the severity of bladder involvement based on cystoscopy findings and provides a numeric score ranging from 0 to 15. The components of the BEDI score include:  1. Bladder erythema: Erythema refers to redness of the bladder mucosa. It is graded on a scale of 0 to 3, with 0 being no erythema and 3 indicating severe erythema.  2. Denudation: Denudation refers to the loss of bladder mucosa, resulting in exposed underlying tissue. It is also graded on a scale of 0 to 3, with 0 indicating no denudation and 3 indicating extensive denudation.  3. Ulceration: Ulceration refers to the presence of ulcers or open sores in the bladder. It is graded on a scale of 0 to 3, with 0 indicating no ulceration and 3 indicating extensive ulceration.  By summing the scores for erythema, denudation, and ulceration, the BEDI score provides an overall assessment of the severity of hemorrhagic cystitis.  It's important to note that the BEDI score is primarily used in research and clinical trials to standardize the assessment of hemorrhagic cystitis in HSCT patients. Its use in routine clinical practice may vary, and the management of hemorrhagic cystitis is typically guided by the patient's symptoms, severity, and underlying cause, in consultation with a healthcare professional experienced in managing such complications. |
| C6 | Poor graft function, also known as graft dysfunction or graft failure, refers to a condition in which the transplanted donor cells in allogeneic hematopoietic stem cell transplantation (HSCT) fail to adequately function and produce normal blood cell counts. It is characterized by insufficient or delayed engraftment, resulting in low peripheral blood cell counts and compromised hematopoiesis.  Poor graft function can manifest as a deficiency in multiple blood cell lineages, including red blood cells (anemia), white blood cells (leukopenia), and platelets (thrombocytopenia). The specific symptoms and severity can vary among individuals but may include fatigue, increased risk of infections, bleeding tendencies, and compromised immune function.  There are several potential causes of poor graft function, including:  1. Insufficient Stem Cell Dose: The number of transplanted stem cells may be insufficient to establish adequate hematopoiesis.  2. Poor Stem Cell Quality: Stem cells obtained from the donor may have reduced viability or functionality, impacting their ability to engraft and differentiate.  3. Impaired Stem Cell Homing: The transplanted stem cells may fail to migrate to the bone marrow niche effectively, hindering their ability to establish engraftment.  4. Host Factors: Various patient-related factors, such as prior treatments, underlying diseases, or comorbidities, can influence the success of graft function.  The treatment approach for poor graft function depends on the underlying cause and the patient's overall condition. Potential treatment options may include:  1. Supportive Care: This includes red blood cell and platelet transfusions to manage anemia and thrombocytopenia, respectively, as well as administration of growth factors to stimulate blood cell production.  2. Donor Lymphocyte Infusion (DLI): Infusion of additional lymphocytes from the original donor may help stimulate graft function and enhance immune response.  3. Adjusting Conditioning Regimen or Immunosuppressive Medications: Modifying the conditioning regimen or immunosuppressive medications can optimize the engraftment environment and immune function.  4. Stem Cell Boost or Second Transplant: Reinfusion of additional donor stem cells or consideration of a second transplant may be necessary to overcome graft dysfunction.  The management of poor graft function requires close monitoring and collaboration among a multidisciplinary healthcare team, including hematologists, transplant specialists, and supportive care providers. The treatment approach is individualized based on the specific circumstances and aims to optimize engraftment, restore hematopoiesis, and improve the patient's overall outcomes. | Poor graft function refers to a condition following allogeneic hematopoietic stem cell transplantation (HSCT) where the transplanted donor cells do not adequately function to produce the expected blood cell counts. It is characterized by insufficient hematopoietic recovery, resulting in low or inadequate levels of red blood cells, white blood cells, and platelets. Poor graft function can manifest as:  1. Delayed Engraftment: The time it takes for the transplanted donor cells to establish and sustain adequate blood cell production is significantly prolonged compared to expected timelines.  2. Cytopenias: Persistent or recurring low blood cell counts, including anemia (low red blood cells), neutropenia (low neutrophils), and thrombocytopenia (low platelets).  3. Incomplete Donor Chimerism: Analysis of the patient's blood or bone marrow may reveal lower than expected levels of donor-derived cells, indicating incomplete engraftment.  4. Persistent or Recurrent Infections: Patients with poor graft function may be prone to infections due to compromised immune function resulting from inadequate hematopoietic recovery.  The exact causes of poor graft function can be multifactorial and may include factors such as graft rejection, graft failure, immune-mediated complications, infections, or other transplant-related complications. The underlying mechanisms leading to poor graft function can vary among patients.  Management of poor graft function typically involves a comprehensive evaluation by the healthcare team, including hematologists, transplant specialists, and other relevant specialists. The treatment approach may include:  1. Supportive Care: Symptomatic management of cytopenias through red blood cell or platelet transfusions to alleviate symptoms and manage bleeding risks.  2. Growth Factors: Administration of growth factors such as erythropoietin, granulocyte colony-stimulating factor (G-CSF), or thrombopoietin receptor agonists to stimulate blood cell production.  3. Immunosuppressive Therapy: In cases where immune-mediated complications contribute to poor graft function, immunosuppressive medications may be utilized to suppress the immune response and improve graft function.  4. Donor Lymphocyte Infusion (DLI): DLI, the infusion of additional donor lymphocytes, may be considered to enhance the immune response and promote graft recovery.  5. Salvage Therapies or Second Transplant: In some cases, more aggressive approaches such as salvage chemotherapy, immunosuppressive therapy, or even a second stem cell transplant may be considered if appropriate.  The management of poor graft function is highly individualized, and the treatment plan is based on the specific circumstances of the patient, underlying causes, and available therapeutic options. Close monitoring and collaboration with the healthcare team are crucial to optimize patient outcomes. | Poor graft function, also known as graft dysfunction, refers to a condition in which the transplanted donor graft after hematopoietic stem cell transplantation (HSCT) fails to adequately restore normal blood cell production. In this condition, the transplanted stem cells may have partially or fully engrafted, but they do not function optimally, resulting in persistent cytopenias (low blood cell counts).  Poor graft function can manifest as one or more of the following:  1. Delayed Engraftment: The time required for the donor graft to establish sufficient blood cell production is significantly prolonged compared to the expected timeframe. This delay can occur for all blood cell lineages (red blood cells, white blood cells, and platelets).  2. Cytopenias: Patients with poor graft function may have persistently low levels of one or more blood cell types, such as anemia (low red blood cells), neutropenia (low white blood cells), or thrombocytopenia (low platelets).  3. Incomplete Recovery: Despite initial signs of engraftment, the blood cell counts fail to reach normal levels or remain inadequate for the patient's needs. This can lead to symptoms of fatigue, increased susceptibility to infections, and bleeding tendencies.  The causes of poor graft function can be multifactorial and include factors such as:  - Inadequate Stem Cell Dose: Insufficient number of transplanted stem cells to establish proper hematopoiesis.  - Impaired Stem Cell Quality: Stem cells may be damaged during collection, processing, or storage, affecting their ability to function normally.  - Immune-Mediated Rejection: The recipient's immune system may mount an immune response against the donor graft, resulting in its dysfunction.  - Infections: Viral, bacterial, or fungal infections can hinder the recovery and proper function of the transplanted graft.  - Medications and Conditioning Regimen: Certain medications or conditioning regimens used in HSCT can negatively impact the function of the transplanted graft.  The management of poor graft function depends on the underlying cause and severity of the condition. Treatment options may include:  - Supportive Care: Red blood cell and platelet transfusions to manage anemia and thrombocytopenia, respectively.  - Growth Factors: Administration of growth factors such as erythropoietin or granulocyte colony-stimulating factor (G-CSF) to stimulate blood cell production.  - Immunosuppressive Therapy: Immunosuppressive medications to dampen immune responses and prevent immune-mediated graft rejection.  - Donor Lymphocyte Infusion (DLI): Infusion of additional lymphocytes from the original donor to enhance graft function.  - Adjustment of Medications or Conditioning Regimen: Modification of medications or conditioning regimen to minimize factors affecting graft function.  - Salvage Transplantation: In severe cases, a second HSCT with a different donor or alternative transplantation approach may be considered.  The specific treatment plan is tailored to the individual patient's condition and requires close collaboration between the transplant team and other specialists involved in the patient's care. |
| C7 | The HCT-CI (Hematopoietic Cell Transplantation-Comorbidity Index) is a scoring system used to assess the comorbidities or pre-transplant conditions of patients undergoing hematopoietic cell transplantation (HCT). It helps predict the risk and outcome of HCT by considering the patient's overall health status and presence of comorbidities.  The HCT-CI score includes the following comorbidity factors:  1. Patient Age:  - Age 0: Under 20 years  - Age 1: 20-29 years  - Age 2: 30-39 years  - Age 3: 40-49 years  - Age 4: 50-59 years  - Age 5: 60-69 years  - Age 6: 70-79 years  - Age 7: 80 years or older  2. Pre-transplant Comorbidities:  The HCT-CI takes into account the presence of specific comorbidities, assigning scores for each condition. These comorbidities include:  - History of prior malignancy  - History of prior solid tumor without recurrence  - History of prior hematologic malignancy with or without recurrence  - Diabetes  - History of cardiac disease  - History of pulmonary disease  - History of renal disease  - History of hepatic disease  - History of seropositivity for viral infections (such as hepatitis B or C)  - Karnofsky Performance Status (a measure of functional status)  Each comorbidity is assigned a score of 0, 1, 2, or 3 based on its impact on transplant outcomes, with a higher score indicating a greater burden of comorbidity.  By summing the scores for each comorbidity factor, the HCT-CI provides an overall assessment of the patient's health status and helps predict the risk of treatment-related complications, survival outcomes, and the feasibility of HCT. The higher the HCT-CI score, the greater the burden of comorbidities, indicating a potentially higher risk associated with the transplantation procedure. | Apologies for the confusion caused by the previous response. To provide accurate information, the HCT-CI (Hematopoietic Cell Transplantation-Comorbidity Index) is a scoring system used to assess the comorbidities or pre-transplant conditions of patients undergoing hematopoietic cell transplantation (HCT). It helps predict the risk and outcome of HCT by considering the patient's overall health status and presence of comorbidities.  The HCT-CI score includes the following comorbidity factors:  1. Nonmalignant Comorbidities:  - Chronic obstructive pulmonary disease (COPD)  - Cardiac diseases (e.g., congestive heart failure, ischemic heart disease)  - Uncontrolled hypertension  - Diabetes mellitus requiring treatment  - Chronic kidney disease (with or without dialysis)  - Hepatic dysfunction (e.g., cirrhosis, abnormal liver function tests)  - Invasive fungal infection  - Active viral infection (e.g., hepatitis B or C)  - Chronic active hepatitis  2. Malignant Comorbidities:  - Current malignancy  - Prior malignancy  Each comorbidity is assigned a score based on its impact on transplant outcomes, with a higher score indicating a greater burden of comorbidity. The assigned scores may vary depending on the specific version of the HCT-CI being used, as updates have been made over time.  By summing the scores for each comorbidity factor, the HCT-CI provides an overall assessment of the patient's health status and helps predict the risk of treatment-related complications, survival outcomes, and the feasibility of HCT. The higher the HCT-CI score, the greater the burden of comorbidities, indicating a potentially higher risk associated with the transplantation procedure. It assists healthcare professionals in evaluating and managing patients undergoing HCT by considering their individual comorbidity profile and tailoring treatment plans accordingly. | I apologize for the incorrect information provided earlier. The correct information regarding the HCT-CI (Hematopoietic Cell Transplantation-Comorbidity Index) is as follows:  The HCT-CI score is a scoring system used to assess comorbidities or pre-transplant conditions in patients undergoing hematopoietic cell transplantation (HCT). It helps predict the risk and outcome of HCT by considering the patient's overall health status and presence of comorbidities.  The HCT-CI score includes the following factors and associated comorbidities:  1. Patient Age:  - Age 0: Under 10 years  - Age 1: 10-19 years  - Age 2: 20-29 years  - Age 3: 30-39 years  - Age 4: 40-49 years  - Age 5: 50-59 years  - Age 6: 60-69 years  - Age 7: 70-79 years  - Age 8: 80 years or older  2. Pre-transplant Comorbidities:  The HCT-CI takes into account the presence of specific comorbidities and assigns scores for each condition. Some of the comorbidities included in the HCT-CI are:  - History of prior malignancy  - Chronic obstructive pulmonary disease (COPD)  - History of congestive heart failure  - History of myocardial infarction  - History of stroke  - Diabetes  - Renal dysfunction  - Liver dysfunction  - Infection (e.g., hepatitis B or C)  - Karnofsky Performance Status (a measure of functional status)  Each comorbidity is assigned a score of 0, 1, 2, or 3 based on its impact on transplant outcomes, with a higher score indicating a greater burden of comorbidity.  The HCT-CI score helps provide an overall assessment of the patient's health status and aids in predicting the risk of treatment-related complications, survival outcomes, and the feasibility of HCT. A higher HCT-CI score indicates a greater burden of comorbidities and may suggest an increased risk associated with the transplantation procedure. |
| C8 | Based on the symptoms described, the patient undergoing HSCT who presents with uncontrolled hypertension, progressive vision disturbances, nonspecific bilateral EEG abnormalities, and undergoes a brain MRI is likely to show findings consistent with posterior reversible encephalopathy syndrome (PRES).  PRES is a clinical syndrome characterized by reversible brain edema, often involving the posterior regions of the brain. The condition can be triggered by various factors, including hypertension, immunosuppressive therapy, and endothelial dysfunction.  The brain MRI in PRES typically shows the following features:  1. T2-weighted and fluid-attenuated inversion recovery (FLAIR) sequences: These sequences often reveal hyperintense signals in the subcortical and cortical regions, predominantly affecting the posterior regions of the brain (occipital lobes), but it can also involve parietal, temporal, and frontal lobes.  2. Diffusion-weighted imaging (DWI): DWI may show increased signal intensity in affected areas, indicating restricted diffusion due to cytotoxic edema.  3. Gadolinium-enhanced imaging: Contrast enhancement is typically absent or minimal in PRES, distinguishing it from other conditions such as cerebral vasculitis or encephalitis.  The diagnosis of PRES is based on the characteristic clinical presentation, neuroimaging findings, and exclusion of other potential causes. The condition is often associated with hypertension, although it can occur in normotensive individuals as well.  Management of PRES involves controlling blood pressure and treating underlying triggers such as immunosuppressive medications. Prompt recognition and intervention are crucial to prevent further neurological complications and to facilitate the resolution of symptoms.  It's important to note that this response is based on the information provided, and a definitive diagnosis can only be made by a qualified healthcare professional who evaluates the patient's complete medical history, physical examination, and relevant diagnostic tests. | In a patient undergoing hematopoietic stem cell transplantation (HSCT) who presents with uncontrolled hypertension, progressive vision disturbances, nonspecific bilateral abnormalities on EEG, and undergoes a brain MRI, several possibilities should be considered.  Based on the provided information, a likely diagnosis could be posterior reversible encephalopathy syndrome (PRES). PRES is a neurological disorder characterized by the development of vasogenic edema in the brain, typically involving the posterior regions. It is commonly associated with uncontrolled hypertension, but can also be triggered by immunosuppressive medications used in HSCT.  The brain MRI in PRES often shows characteristic findings, which may include:  1. Hyperintense signal on T2-weighted and FLAIR (fluid-attenuated inversion recovery) images in the posterior regions of the brain, such as the occipital lobes.  2. Vasogenic edema in the affected areas.  3. Occasionally, the involvement of other brain regions beyond the posterior areas.  These findings are consistent with the reversible nature of PRES, as the condition typically resolves with appropriate management, including blood pressure control and withdrawal of offending medications if applicable.  However, it's important to note that the provided information is limited, and other conditions with similar clinical manifestations may need to be considered. Therefore, the final diagnosis should be made by a qualified healthcare professional based on a comprehensive evaluation of the patient's clinical presentation, imaging findings, and other relevant factors. | Based on the clinical presentation you provided, the likely diagnosis in this patient undergoing HSCT is posterior reversible encephalopathy syndrome (PRES).  PRES is a neurological disorder characterized by the development of reversible neurological symptoms, such as hypertension, vision disturbances, seizures, headache, and altered mental status. It is often associated with acute elevation of blood pressure and endothelial dysfunction. HSCT recipients, particularly those receiving immunosuppressive medications, are at risk for developing PRES.  The brain MRI in PRES typically shows characteristic findings, including:  1. Hyperintensities on T2-weighted and fluid-attenuated inversion recovery (FLAIR) images: These hyperintensities appear as areas of increased signal intensity and are usually found in the subcortical white matter regions. They can involve the parieto-occipital lobes, but other regions such as the frontal and temporal lobes can also be affected.  2. Vasogenic edema: The hyperintense areas on MRI represent vasogenic edema, which is caused by disruption of the blood-brain barrier due to endothelial dysfunction.  It's important to note that the definitive diagnosis of PRES is typically made based on clinical presentation and characteristic imaging findings. Other causes of similar symptoms and MRI findings, such as ischemic stroke or other neurological disorders, should be considered and ruled out.  Management of PRES involves controlling blood pressure, addressing the underlying cause (such as hypertension or immunosuppressive medications), and providing supportive care. Prompt recognition and treatment can lead to a favorable outcome, with most patients experiencing complete resolution of symptoms.  It's crucial for the patient to be evaluated by a healthcare professional experienced in managing HSCT complications to confirm the diagnosis and provide appropriate management. |
| C9 | The most likely explanation for a female patient with acute myeloid leukemia (AML) who undergoes allogeneic hematopoietic stem cell transplantation (HSCT) from a male donor, and subsequently shows a blast percentage of 40% with all cells being 46XY on cytogenetic analysis, is the presence of relapsed AML.  In this scenario, the persistence or reappearance of blasts in the bone marrow along with the presence of male donor cells (46XY) strongly suggests relapse of AML. The development of relapsed AML indicates that the disease has returned after an initial response to HSCT and may be resistant to the effects of the transplantation.  There can be several reasons for relapse after allogeneic HSCT, including residual disease not eradicated by the transplant, the emergence of drug-resistant clones, or graft failure. It is crucial to evaluate the patient thoroughly to confirm relapse and assess the specific characteristics of the disease, such as cytogenetic and molecular abnormalities, to guide further treatment decisions.  Further management options for relapsed AML may include salvage chemotherapy regimens, targeted therapies, donor lymphocyte infusion (DLI), or a subsequent transplant if deemed appropriate based on the patient's overall health and other prognostic factors. Consultation with a hematologist or transplant specialist is necessary to determine the best course of action for the individual patient. | In the scenario you described, the most likely explanation for a female patient with acute myeloid leukemia (AML) who underwent allogeneic hematopoietic stem cell transplantation (HSCT) from a male donor and subsequently shows a blast percentage of 40% with cytogenetic analysis showing all cells as 46XY is relapse of AML.  Relapse refers to the recurrence of leukemia cells after a period of remission following HSCT. In this case, the presence of a high blast percentage (40%) in the bone marrow indicates a significant disease burden, suggesting that the patient's AML has returned.  The cytogenetic analysis showing all cells as 46XY indicates that the leukemia cells present in the bone marrow are of donor origin. This finding suggests that the leukemia cells are derived from the transplanted male donor cells rather than being residual recipient (female) cells.  Relapse of AML after allogeneic HSCT can occur due to several reasons, such as:  1. Inadequate graft-versus-leukemia (GVL) effect: The transplanted donor immune cells may not sufficiently recognize and eliminate the remaining leukemia cells, allowing them to proliferate and cause relapse.  2. Development of resistance: Some leukemia cells may acquire genetic mutations or changes that confer resistance to prior treatments, including chemotherapy and HSCT.  3. Minimal residual disease: There might have been a small population of leukemia cells that survived the conditioning regimen and subsequently expanded to cause relapse.  It is important for the patient to be evaluated by a hematologist or oncologist with expertise in HSCT and leukemia management to confirm the relapse, assess the extent of the disease, and discuss appropriate treatment options, which may include salvage chemotherapy, targeted therapy, or potentially a second transplantation. The specific treatment approach will depend on various factors, including the patient's overall health, prior treatments, and available therapeutic options. | The most likely explanation for a female patient with acute myeloid leukemia (AML) who undergoes allogeneic hematopoietic stem cell transplantation (HSCT) from a male donor and then shows a bone marrow analysis with a blast percentage of 40% and cytogenetic analysis revealing all cells to be 46XY is relapse of the AML.  In this case, the persistence or recurrence of the disease after HSCT suggests that the transplantation was not curative. Despite the transplant from a male donor (who typically has XY chromosomes), the recipient's bone marrow analysis and cytogenetic analysis showing 46XY cells indicate that the transplanted cells did not successfully engraft or replace the patient's leukemic cells.  Relapse after allogeneic HSCT can occur due to various reasons, including inadequate conditioning regimen, residual host leukemic cells, graft failure, or development of new genetic mutations that confer resistance to treatment. It is important to evaluate the patient's clinical condition and discuss appropriate management options, such as salvage chemotherapy, donor lymphocyte infusion, or potentially a second transplant, depending on the specific circumstances and available treatment options.  Further consultation with a hematologist or oncologist experienced in HSCT and AML management is crucial to determine the most appropriate course of action for the patient. |
| C10 | In the scenario where a recipient with blood type O positive (0 pos) receives a hematopoietic stem cell (HSC) transplant from an A positive (A pos) donor, a potential specific complication is the development of ABO blood group incompatibility.  ABO incompatibility occurs when the recipient's immune system recognizes the ABO antigens present on the donor's red blood cells as foreign and mounts an immune response against them. This can result in a condition called ABO hemolysis, where the recipient's immune system destroys the donor red blood cells.  The severity of ABO hemolysis can vary. In some cases, it may be mild and self-limiting, while in others, it can be more severe and require intervention. The complications associated with ABO incompatibility include:  1. Hemolytic transfusion reaction: This occurs when the recipient's immune system recognizes the ABO antigens on the donor red blood cells and attacks them, leading to red blood cell destruction and potential symptoms such as fever, chills, jaundice, and kidney injury.  2. Delayed red blood cell engraftment: ABO incompatibility can delay the engraftment of the donor red blood cells, leading to prolonged anemia and the need for ongoing blood transfusions.  To mitigate the risk of ABO incompatibility, careful compatibility testing and selection of compatible blood products can be performed. In some cases, pre-transplant conditioning regimens can also help reduce the immune response against the donor red blood cells. Treatment options for ABO hemolysis include supportive care, blood transfusions, and, in severe cases, interventions such as plasmapheresis or immunosuppressive therapy.  It's important to note that the management of ABO incompatibility and its complications should be determined on an individual basis by a healthcare professional experienced in transplant medicine and blood compatibility issues. | In the scenario where a patient with blood type O positive (0 pos) receives a hematopoietic stem cell (HSC) transplant from an A positive (A pos) donor, the potential specific complication that can occur is ABO incompatibility.  ABO incompatibility arises when there is a mismatch between the ABO blood types of the donor and recipient. In this case, the patient's blood type O (lacking A or B antigens) is incompatible with the donor's blood type A (expressing A antigens). This ABO mismatch can lead to the development of ABO-associated complications.  The major complications associated with ABO incompatibility in HSC transplantation include:  1. Hemolysis: ABO-incompatible red blood cells (RBCs) can be recognized and attacked by recipient antibodies, leading to hemolysis (destruction of RBCs) and subsequent anemia.  2. Delayed engraftment: The presence of ABO-incompatible RBCs in the recipient's bloodstream can inhibit the engraftment and recovery of the donor HSCs, potentially resulting in delayed hematopoietic recovery.  3. Graft-versus-host disease (GVHD): ABO incompatibility can increase the risk of GVHD, a condition in which donor immune cells recognize the recipient's tissues as foreign and mount an immune response against them. The risk of GVHD may be higher in ABO-incompatible transplants due to the presence of ABO antigens on the recipient's tissues.  To mitigate these complications, various strategies can be employed, such as pre-transplant conditioning regimens, red blood cell depletion to minimize the presence of incompatible RBCs, and careful monitoring for signs of hemolysis and GVHD. The management of ABO incompatibility and its associated complications requires close collaboration between the transplant team and a transfusion medicine specialist to ensure appropriate supportive care and interventions. | In the scenario you described, where a patient with blood type O positive (0 pos) receives a hematopoietic stem cell (HSC) transplant from an A positive (A pos) donor, the potential specific complication that can arise is ABO incompatibility.  ABO incompatibility occurs when there is a mismatch between the ABO blood types of the donor and recipient. The ABO blood group system consists of four main types: A, B, AB, and O. Each type has specific antigens on the surface of red blood cells and corresponding antibodies in the plasma.  In the case of an ABO mismatch, the recipient's antibodies against the donor's blood type may recognize the donor's red blood cells as foreign and mount an immune response. This can lead to complications such as:  1. Hemolysis: The recipient's antibodies can attack and destroy the donor's red blood cells, causing hemolysis (breakdown of red blood cells) and resulting in anemia and other associated symptoms.  2. Delayed engraftment: The immune response against the donor's blood cells can impair the engraftment of the transplanted stem cells, leading to delayed or suboptimal recovery of blood cell production.  3. Graft failure: In severe cases, the immune response may result in graft failure, where the transplanted cells fail to establish and sustain healthy hematopoiesis.  To mitigate the risks associated with ABO incompatibility, various measures can be taken, such as:  - Pre-transplant conditioning: Intensive conditioning regimens before transplantation aim to suppress the recipient's immune system and reduce the risk of immune-mediated complications.  - Immunomodulation: Immunosuppressive medications, such as corticosteroids or other immune-modulating drugs, may be used to minimize the immune response and prevent or manage complications.  - Red blood cell support: Blood transfusions with compatible blood products may be necessary to manage anemia resulting from hemolysis.  It's important for the healthcare team overseeing the transplantation to be aware of the ABO incompatibility and closely monitor the patient for any signs of complications. Close collaboration with a hematologist or transplant specialist is crucial to provide appropriate management and support for the patient in this setting. |
| C11 | The constellation of symptoms described in the patient undergoing hematopoietic stem cell transplantation (HSCT), including progressive hypertension, decreased renal function, worsening anemia, and thrombocytopenia, is indicative of a potential complication called thrombotic microangiopathy (TMA).  TMA is a condition characterized by microvascular thrombosis in various organs, leading to organ dysfunction and the formation of small blood clots. In the context of HSCT, TMA can occur as a result of various factors, such as conditioning regimens, immunosuppressive medications (like cyclosporine), infections, graft-versus-host disease (GVHD), or endothelial damage during the transplantation process.  When TMA is present, certain laboratory findings on the blood smear can be observed, including:  1. Schistocytes: Schistocytes are fragmented red blood cells that result from mechanical destruction as they pass through narrowed or obstructed blood vessels. They have a characteristic fragmented appearance, often described as helmet or triangular shapes.  2. Helmet cells: Helmet cells are a specific type of schistocyte that has a remnant of red blood cell membrane resembling a helmet or a half-moon shape.  The presence of schistocytes and helmet cells on a blood smear suggests the occurrence of microangiopathic hemolytic anemia, which is a hallmark of TMA.  It's important to note that other laboratory abnormalities, such as elevated lactate dehydrogenase (LDH) and decreased haptoglobin levels, can also be observed in TMA.  Prompt recognition and management of TMA are crucial to prevent further organ damage and optimize patient outcomes. Treatment may involve discontinuation of potential causative medications, supportive care measures, and, in some cases, specific therapies such as plasma exchange or targeted agents to address the underlying cause of TMA.  Consultation with a hematologist or transplant specialist is essential to evaluate and manage the patient's condition appropriately. | The clinical presentation of progressive hypertension, decreased renal function, worsening anemia, and thrombocytopenia in a patient undergoing HSCT, with the temporary discontinuation of cyclosporine, is suggestive of a condition called thrombotic microangiopathy (TMA).  In TMA, there is a disruption of the small blood vessels, leading to the formation of blood clots and subsequent organ damage. In this context, a laboratory finding often associated with TMA on a blood smear is the presence of fragmented red blood cells (schistocytes). Schistocytes are fragmented or irregularly shaped red blood cells that result from the mechanical destruction of red blood cells as they pass through narrowed or damaged blood vessels.  The presence of schistocytes on a blood smear is an important clue suggesting microangiopathic hemolytic anemia, which is a hallmark feature of TMA. Other findings that may be observed on a blood smear in TMA include helmet cells (also known as bite cells) and spherocytes.  It's important to note that TMA can have various underlying causes, including graft-versus-host disease (GVHD), infections, medications (such as calcineurin inhibitors like cyclosporine), and other factors. Prompt recognition and management of TMA are crucial to prevent further organ damage. Consultation with a healthcare professional experienced in transplant medicine is essential to confirm the diagnosis, determine the underlying cause, and initiate appropriate treatment. | The clinical presentation of progressive hypertension, decreased renal function, worsening anemia, and thrombocytopenia in a patient undergoing hematopoietic stem cell transplantation (HSCT) is suggestive of a condition called microangiopathic hemolytic anemia (MAHA).  In the context of MAHA, a specific laboratory finding that can be observed on a blood smear is the presence of schistocytes or fragmented red blood cells. Schistocytes are fragmented red blood cells that result from mechanical damage as they pass through narrowed or damaged blood vessels. The presence of schistocytes indicates the occurrence of microvascular injury and hemolysis, which is a characteristic feature of MAHA.  MAHA can be caused by various underlying conditions, including graft-versus-host disease (GVHD), thrombotic microangiopathy (TMA), or other transplant-related complications. The discontinuation of cyclosporine, which is an immunosuppressive medication commonly used in HSCT, may suggest a potential association with GVHD or TMA.  It's important to further evaluate the patient's condition, including conducting additional laboratory tests and clinical assessments, to determine the precise cause of the microangiopathic hemolytic anemia and guide appropriate management. Consulting with a hematologist or transplant specialist is crucial in this situation to ensure accurate diagnosis and timely intervention. |
| C12 | Primary and secondary graft failure are both complications that can occur following hematopoietic stem cell transplantation (HSCT). Here's a breakdown of the differences between primary and secondary graft failure and possible treatments for each:  1. Primary Graft Failure:  - Definition: Primary graft failure refers to the inability of the transplanted donor stem cells to engraft and establish sustained hematopoiesis in the recipient's bone marrow.  - Timing: It typically occurs within the first few weeks following transplantation.  - Possible Causes: Primary graft failure can result from various factors, including inadequate stem cell dose, impaired stem cell quality, pre-transplant conditioning regimen-related toxicity, or graft rejection by the recipient's immune system.  - Treatment Options:  - Increasing Stem Cell Dose: If the primary graft failure is due to an inadequate stem cell dose, a second transplant or additional stem cell infusion may be considered to boost the number of stem cells and improve engraftment.  - Immunosuppressive Therapy: In cases where graft rejection is suspected, immunosuppressive medications may be administered to dampen the recipient's immune response and allow the donor stem cells to engraft.  - Alternative Donor Sources: If primary graft failure occurs with a matched unrelated donor, exploring alternative donor sources such as haploidentical or cord blood transplantation may be considered.  2. Secondary Graft Failure:  - Definition: Secondary graft failure refers to the loss of previously established hematopoietic engraftment following an initially successful transplantation.  - Timing: It occurs after an initial period of successful engraftment and hematopoietic recovery.  - Possible Causes: Secondary graft failure can be caused by a range of factors, including infections, graft-versus-host disease (GVHD), relapse of the underlying disease, or medications that suppress the immune system.  - Treatment Options:  - Addressing Underlying Causes: The specific treatment approach for secondary graft failure depends on the underlying cause. This may involve treating infections, managing GVHD, adjusting immunosuppressive medications, or initiating disease-specific therapies for disease relapse.  - Donor Lymphocyte Infusion (DLI): DLI, which involves infusing lymphocytes from the original donor, may be considered to boost the graft and promote re-establishment of hematopoiesis.  - Re-transplantation: In severe cases of secondary graft failure, a re-transplantation or salvage transplant may be necessary using a different donor source or modified conditioning regimen.  It's important to note that the treatment of graft failure is complex and individualized, and decisions regarding treatment options should be made in consultation with a transplant specialist or hematologist/oncologist who has expertise in HSCT. The specific approach will depend on the patient's unique circumstances, underlying causes, and available resources. | Primary graft failure and secondary graft failure are two distinct complications that can occur after hematopoietic stem cell transplantation (HSCT). Here's an overview of the differences between the two and potential treatments:  1. Primary Graft Failure:  Primary graft failure refers to the failure of the transplanted donor stem cells to engraft and establish proper hematopoiesis in the recipient's bone marrow. It typically occurs within the first few weeks after HSCT. It can result from various factors, including poor stem cell quality, high levels of pre-transplant conditioning, graft rejection, or impaired bone marrow microenvironment.  Treatment options for primary graft failure may include:  - Donor Lymphocyte Infusion (DLI): DLI involves infusing additional lymphocytes from the original stem cell donor to promote graft acceptance and engraftment.  - Alternative Donor Sources: If the primary graft fails, alternative donor sources like haploidentical donors or cord blood units may be considered.  - Cytokine Support: Administration of growth factors such as granulocyte-colony stimulating factor (G-CSF) or erythropoietin to stimulate blood cell production.  - Immunosuppressive Therapy: In some cases, immunosuppressive medications like corticosteroids or anti-thymocyte globulin (ATG) may be used to suppress the recipient's immune system and improve engraftment.  2. Secondary Graft Failure:  Secondary graft failure, also known as late graft failure or late graft rejection, occurs when a previously established graft, which initially functioned well, begins to fail. It can happen several months or even years after HSCT and may be caused by various factors, including relapse of the underlying disease, re-emergence of recipient cells, or immune-mediated mechanisms.  Treatment options for secondary graft failure may include:  - Donor Lymphocyte Infusion (DLI): DLI may be utilized to boost the graft and reestablish hematopoiesis.  - Salvage Chemotherapy: Chemotherapy regimens may be considered to eliminate residual recipient cells and promote the growth of donor cells.  - Alternative Donor Sources: If the original donor source fails, alternative donor sources may be explored.  - Reduced-Intensity Conditioning (RIC) or Repeat HSCT: In some cases, a repeat HSCT with a reduced-intensity conditioning regimen may be considered.  It's important to note that the treatment approach for graft failure depends on the individual patient, the specific circumstances, and the expertise of the healthcare team. The treatment decisions are typically made in consultation with transplant physicians, considering factors such as the patient's overall health, the underlying disease, and the available donor options. | Primary graft failure and secondary graft failure are two distinct complications that can occur after allogeneic hematopoietic stem cell transplantation (HSCT). Here's an overview of the differences between the two and possible treatments:  1. Primary Graft Failure:  - Definition: Primary graft failure, also known as primary graft non-engraftment, refers to the failure of the transplanted donor cells to engraft and produce adequate blood cell counts.  - Cause: Primary graft failure can occur due to various reasons, including insufficient stem cell dose, poor stem cell quality, impaired stem cell homing, or host factors that inhibit engraftment.  - Treatment: The treatment approach for primary graft failure depends on the underlying cause. Possible treatments may include:  - Reinfusion of additional donor stem cells (stem cell boost or second transplant) to overcome the engraftment barrier.  - Adjustment of conditioning regimen or immunosuppressive medications to optimize engraftment conditions.  - Supportive care measures such as red blood cell and platelet transfusions to manage symptoms related to low blood cell counts.  - In some cases, alternative treatment options like a different donor source (if available) or alternative transplantation approaches may be considered.  2. Secondary Graft Failure:  - Definition: Secondary graft failure refers to the loss of previously achieved engraftment and sustained hematopoiesis after an initial successful engraftment following HSCT.  - Cause: Secondary graft failure can be caused by various factors, including infections, graft-versus-host disease (GVHD), relapse of underlying disease, or immune-mediated rejection of the graft.  - Treatment: The treatment of secondary graft failure depends on the underlying cause and the patient's overall condition. Possible treatments may include:  - Identification and management of any underlying infections or complications that may have triggered the graft failure.  - Evaluation and treatment of graft-versus-host disease if it is a contributing factor.  - Donor lymphocyte infusion (DLI) to enhance immune response and promote graft recovery.  - Salvage therapies, such as chemotherapy or immunosuppressive medications, to suppress the immune system and allow the graft to recover.  - Consideration of a second transplant or alternative treatment approaches based on the specific circumstances.  The management of primary and secondary graft failure requires a comprehensive evaluation by a healthcare team specialized in HSCT. The treatment approach is tailored to the individual patient's condition, underlying causes, and available options to optimize engraftment and restore hematopoiesis. |
| C13 | In the scenario you described, where a patient with acute myeloid leukemia (AML) relapses 12 months after haploidentical transplant and demonstrates loss of patient-donor mismatched human leukocyte antigen (HLA) in the leukemic cells, the consideration of donor lymphocyte infusion (DLI) as a treatment option is appropriate.  DLI involves infusing lymphocytes obtained from the original stem cell donor into the recipient with the goal of inducing a graft-versus-leukemia (GVL) effect. The infused lymphocytes recognize and attack residual leukemic cells, potentially leading to disease remission.  Several factors should be considered when determining whether to offer DLI in this situation:  1. Timing and aggressiveness of relapse: The duration between transplantation and relapse, as well as the aggressiveness of the relapse, can influence the likelihood of DLI being effective. Generally, DLI is more successful when the relapse occurs later and is less aggressive.  2. Feasibility of obtaining lymphocytes: Assessing whether a sufficient number of lymphocytes can be obtained from the donor for infusion is important. This can depend on the donor's availability, the previous graft source, and the patient's current clinical status.  3. Conditioning regimen and prior immunosuppression: The conditioning regimen used during the initial transplant and any subsequent immunosuppressive therapy may affect the likelihood of DLI success. If the conditioning regimen was myeloablative and immunosuppression is minimal, DLI may have a higher chance of inducing a GVL effect.  4. Overall health and tolerance to potential side effects: DLI carries the risk of graft-versus-host disease (GVHD) and other potential complications. The patient's overall health and ability to tolerate these risks should be considered.  It is essential for the decision regarding DLI to be made by a multidisciplinary team of hematologists and transplant specialists who can assess the specific characteristics of the patient's disease, evaluate the potential benefits and risks of DLI, and discuss the available treatment options. | In the case of a patient with acute myeloid leukemia (AML) who experiences a relapse 12 months after haploidentical transplantation and demonstrates loss of patient-donor mismatched HLA in the leukemic cells, the use of donor lymphocyte infusion (DLI) may be a potential treatment option.  DLI involves infusing lymphocytes from the original donor into the recipient's bloodstream with the goal of inducing a graft-versus-leukemia (GVL) effect. The infused lymphocytes contain immune cells that can recognize and attack the leukemia cells, potentially leading to disease control or remission.  Factors to consider when determining if DLI should be offered include:  1. Feasibility: The availability of suitable donor lymphocytes, their compatibility with the recipient, and the logistics of the infusion process need to be evaluated.  2. Disease status and aggressiveness: The stage and aggressiveness of the relapsed AML, along with other prognostic factors, should be taken into account to assess the potential benefits of DLI.  3. Prior response to transplantation: The patient's previous response to haploidentical transplantation and any prior experiences with DLI should be considered.  4. Patient's overall health: The patient's general health and ability to tolerate the potential side effects of DLI should be evaluated.  It is important to note that DLI can be associated with significant risks, including graft-versus-host disease (GVHD) and other transplant-related complications. These risks should be carefully balanced against the potential benefits of achieving disease control or remission.  The decision to offer DLI should be made on an individualized basis, involving a multidisciplinary team of hematologists, transplant specialists, and other relevant healthcare professionals. The team will consider the specific characteristics of the patient's disease, the patient's overall health, and the available treatment options to determine the most appropriate course of action. | In the scenario you described, where a patient with acute myeloid leukemia (AML) relapses 12 months after haploidentical transplant and demonstrates loss of patient-donor mismatched HLA (human leukocyte antigen) in the leukemic cells, donor lymphocyte infusion (DLI) can be considered as a potential treatment option.  DLI involves infusing lymphocytes from the original stem cell donor into the recipient with the aim of inducing a graft-versus-leukemia (GVL) effect. It can be effective in treating relapsed or persistent leukemia after transplantation, especially when there is evidence of loss of HLA mismatched leukemic cells, as in this case.  The decision to offer DLI depends on several factors, including:  1. Overall patient health: The patient's general health and performance status are crucial considerations to ensure they can tolerate the potential side effects of DLI.  2. Disease status and extent of relapse: The extent of disease relapse, such as the involvement of bone marrow or other organs, plays a role in determining the potential effectiveness of DLI.  3. Previous treatment response: The response to prior therapy, including the initial transplant, is important to assess the likelihood of DLI success.  4. Risk-benefit assessment: The potential benefits of DLI, such as achieving remission or disease control, need to be weighed against the risks, including graft-versus-host disease (GVHD), organ toxicity, and other potential complications.  It's important to note that the decision to offer DLI should be made in consultation with a hematologist or transplant specialist experienced in managing AML and transplantation. They will consider the individual patient's circumstances, including their medical history, disease characteristics, and other factors, to determine the most appropriate treatment approach for relapsed AML after haploidentical transplant. |
| C14 | Uniparental disomy (UPD) of the short arm of chromosome 6 (6p) in leukemic cells of a patient with relapsed acute myeloid leukemia (AML) after haploidentical hematopoietic stem cell transplantation (HSCT) can have several effects and impact treatment options. Here are some key considerations:  1. Loss of heterozygosity: UPD refers to a situation where both copies of a chromosome or a segment of a chromosome are inherited from a single parent. In the case of 6p UPD, it means that both copies of this chromosome segment are inherited from either the mother or the father. This leads to the loss of heterozygosity, where the patient loses the genetic diversity that would normally be present in a diploid state.  2. Impact on treatment options: The presence of 6p UPD can have implications for treatment options and response to therapy. UPD has been associated with the acquisition of genetic alterations that may confer resistance to certain chemotherapy drugs or targeted therapies. This means that the leukemic cells with 6p UPD may be less responsive to conventional treatments, making the disease more challenging to control.  3. Prognostic significance: The presence of specific genetic alterations, such as 6p UPD, can have prognostic implications in AML. Studies have shown that 6p UPD is associated with an adverse prognosis, indicating a higher likelihood of treatment failure and poorer overall survival. It suggests that the disease may be more aggressive and difficult to manage.  4. Need for personalized treatment: The detection of 6p UPD highlights the importance of personalized treatment approaches. It may be necessary to explore alternative treatment strategies, such as targeted therapies or experimental treatment options, to overcome the resistance conferred by this genetic alteration. Precision medicine approaches, such as genomic profiling of the leukemic cells, can help identify specific mutations and guide the selection of targeted therapies that may be more effective against the specific genetic alterations present in the patient's leukemia.  5. Consultation with a hematologist/oncologist: In cases of relapsed AML with genetic abnormalities like 6p UPD, it is crucial for the patient to consult with a hematologist/oncologist who specializes in AML and has experience in managing relapsed/refractory disease. They can assess the specific genetic profile of the leukemia cells, evaluate the treatment options, and determine the most appropriate therapeutic approach, which may include targeted therapies, clinical trials, or alternative treatment strategies.  It's important to note that treatment decisions in AML are highly individualized, and additional factors, such as the patient's overall health, previous treatments received, and other genetic abnormalities, will also influence the treatment plan. | Uniparental disomy (UPD) refers to a genetic abnormality in which both copies of a chromosome pair are inherited from one parent, resulting in the loss of heterozygosity (LOH) for specific genetic regions. In the case of a patient with acute myeloid leukemia (AML) who relapses after haploidentical hematopoietic stem cell transplantation (HSCT) and exhibits UPD of the short arm of chromosome 6 in leukemic cells, it can have several implications and impact treatment options.  The loss of heterozygosity due to UPD can result in the following effects:  1. Loss of tumor suppressor genes: Chromosome 6 contains multiple tumor suppressor genes involved in regulating cell growth, differentiation, and apoptosis. UPD leading to LOH can result in the loss of one functional copy of these tumor suppressor genes, potentially compromising their normal function and contributing to the development or progression of cancer, including AML.  2. Clonal evolution: UPD may represent clonal evolution in AML, indicating the acquisition of additional genetic alterations during disease progression or relapse. Clonal evolution can lead to increased genetic complexity, heterogeneity, and potentially resistance to treatment.  The impact of UPD on treatment options depends on various factors, including the specific genes affected and the presence of other genetic mutations. However, some general considerations are:  1. Risk stratification: The identification of UPD in leukemic cells can provide additional prognostic information. Certain genetic alterations, including specific UPD patterns, may be associated with adverse outcomes or increased risk of relapse. This information can help guide treatment decisions and influence the choice of intensity and duration of post-transplant therapies.  2. Targeted therapies: The identification of specific genetic abnormalities, including UPD, can guide the selection of targeted therapies. If the UPD involves a gene known to be associated with a therapeutic target, such as a specific mutation or fusion, it may provide an opportunity for targeted treatment approaches.  3. Clinical trials: Patients with relapsed or refractory AML, especially those with complex genetic alterations such as UPD, may be eligible for enrollment in clinical trials evaluating novel therapies or treatment strategies. Clinical trials can offer access to experimental treatments that are not yet widely available and may provide potential therapeutic options beyond standard treatments.  It is important to note that the impact of UPD on treatment options is highly individualized, and decisions regarding therapy should be made in consultation with a hematologist/oncologist specializing in AML and based on a comprehensive assessment of the patient's clinical characteristics, genetic profile, and available treatment options. | Uniparental disomy (UPD) refers to a condition in which an individual inherits two copies of a specific chromosome or chromosome segment from one parent, instead of one copy from each parent. In the context of a patient with acute myeloid leukemia (AML) who has relapsed after haploidentical hematopoietic stem cell transplantation (HSCT) and has UPD of the short arm of chromosome 6 in leukemic cells, it has implications for treatment options and prognosis.  The loss of heterozygosity due to UPD can lead to several effects:  1. Loss of tumor suppressor genes: UPD can result in the loss of heterozygosity for tumor suppressor genes located on the affected chromosome segment. This loss can contribute to the development or progression of leukemia.  2. Acquisition of new genetic alterations: During disease progression and relapse, leukemic cells may acquire additional genetic mutations or alterations, leading to a more aggressive phenotype. UPD of chromosome 6 might coincide with the acquisition of these secondary genetic abnormalities.  The impact of UPD of the short arm of chromosome 6 on treatment options and prognosis depends on the specific genes and genetic alterations involved. It is essential to conduct further genetic testing, such as chromosome banding analysis, fluorescence in situ hybridization (FISH), or next-generation sequencing (NGS), to identify specific mutations or abnormalities associated with the UPD and their clinical significance.  In terms of treatment options, the presence of UPD and associated genetic alterations may affect the response to chemotherapy or targeted therapies. Certain genetic alterations may confer resistance to standard chemotherapy drugs or alter the sensitivity to specific targeted therapies. Therefore, the treatment plan may need to be modified accordingly.  Additionally, the presence of UPD and associated genetic abnormalities can provide important prognostic information. Some genetic alterations may be associated with a poorer prognosis or increased risk of relapse, while others may be associated with specific treatment responses or favorable outcomes. Understanding the specific genetic landscape can help guide treatment decisions and prognostic assessments.  In this scenario, it is crucial for the patient's healthcare team, including hematologists, oncologists, and genetic specialists, to review the genetic findings and assess their implications for treatment options and prognosis. Individualized treatment plans can then be developed based on the patient's specific genetic profile, disease characteristics, and overall health status. |
| C15 | Based on the symptoms described in the patient undergoing allogeneic hematopoietic stem cell transplantation (HSCT) for hematologic malignancy, the clinical picture suggests the development of veno-occlusive disease (VOD), also known as sinusoidal obstruction syndrome (SOS). VOD/SOS is a potentially severe complication that affects the liver and can lead to hepatomegaly, ascites, weight gain, and elevated bilirubin levels.  The most effective drug for the treatment of VOD/SOS is defibrotide. Defibrotide is an endothelial cell protector and profibrinolytic agent that helps to restore microvascular integrity and reduce the obstruction within the hepatic veins. It has been approved for the treatment of severe VOD/SOS with multi-organ dysfunction post-HSCT in certain regions.  Defibrotide has shown efficacy in improving survival and reversing the clinical manifestations of VOD/SOS. It should be initiated as soon as the diagnosis of VOD/SOS is suspected. The dosage and duration of treatment may vary based on the severity of the condition and the individual patient's response.  It's important to note that the use of defibrotide or any specific drug for VOD/SOS should be guided by a hematologist or transplant specialist experienced in managing HSCT complications. The healthcare professional will consider the patient's overall condition, severity of symptoms, and available treatment options to determine the most appropriate management plan. | Based on the symptoms described, such as rapidly progressive ascites, weight gain, increased bilirubin levels, and painful hepatomegaly in a patient undergoing allogeneic hematopoietic stem cell transplantation (HSCT), the most effective drug for this condition could be diuretics, specifically a loop diuretic such as furosemide.  These symptoms are suggestive of hepatic veno-occlusive disease (VOD), also known as sinusoidal obstruction syndrome (SOS). VOD/SOS is a serious complication of HSCT characterized by the obstruction of small hepatic veins leading to liver dysfunction and fluid accumulation.  Management of VOD/SOS typically involves supportive care measures, including diuretics to help manage fluid overload. Loop diuretics like furosemide are commonly used to promote diuresis and reduce fluid retention in conditions like ascites.  It's important to note that the choice of diuretic and the specific treatment approach should be determined by the patient's clinical presentation, overall health, and the judgment of the treating healthcare professional. Dosage and administration of diuretics will vary depending on individual patient factors and should be carefully monitored to achieve the desired response while minimizing side effects.  Additionally, other supportive measures such as low-sodium diet, fluid restriction, and close monitoring of liver function and renal parameters may also be employed in the management of VOD/SOS.  Consultation with a hematologist or transplant specialist experienced in managing HSCT complications is crucial to guide the appropriate treatment plan for this specific patient. | In the scenario described, where a 56-year-old male patient undergoing allogeneic hematopoietic stem cell transplantation (HSCT) develops rapidly progressive ascites, weight gain, increased bilirubin level, and painful hepatomegaly, the most effective drug for managing this condition is diuretic therapy with spironolactone.  The symptoms and findings described are suggestive of hepatic veno-occlusive disease (VOD), also known as sinusoidal obstruction syndrome (SOS). VOD/SOS is a potential complication of HSCT that can occur due to damage to the small hepatic veins and sinusoidal endothelium.  Diuretic therapy with spironolactone, a potassium-sparing diuretic, is commonly used in the management of fluid overload, including ascites. Spironolactone helps to increase sodium and water excretion while conserving potassium levels, thus reducing fluid retention.  The addition of loop diuretics, such as furosemide, may be considered in combination with spironolactone if the response to spironolactone alone is inadequate. Loop diuretics act on the loop of Henle in the kidneys and promote further diuresis.  However, it's important to note that the management of hepatic VOD/SOS requires a multidisciplinary approach and individualized treatment based on the patient's specific clinical condition. Supportive care measures, such as fluid restriction, analgesics for pain management, and close monitoring of liver function and fluid balance, are also essential.  Consultation with a healthcare professional experienced in managing HSCT complications, such as a hematologist or transplant specialist, is crucial to determine the most appropriate treatment plan and to address any underlying causes contributing to the development of hepatic VOD/SOS in the specific patient's case. |
| C16 | The prognosis of a patient with acute myeloid leukemia (AML) who is offered allogeneic hematopoietic stem cell transplantation (HSCT) can be influenced by various factors, including the patient's age, performance status, comorbidities, response to initial treatment, and specific cytogenetic abnormalities.  The specific cytogenetic abnormalities mentioned in this case are the translocation between chromosomes 1 and 2 (t(1,2)) and deletion of chromosome 7 (-7). The presence of these abnormalities indicates an adverse risk profile in AML.  The t(1,2) translocation and -7 deletion are both considered poor prognostic factors in AML. They are associated with a higher risk of disease progression, resistance to therapy, and poorer overall outcomes. However, it's important to note that prognosis is not solely determined by cytogenetic abnormalities, but is also influenced by other factors as mentioned above.  The 5-year prognosis for AML patients with adverse cytogenetic abnormalities, such as t(1,2) and -7, is generally considered less favorable compared to those with more favorable cytogenetic profiles. The exact prognosis can vary depending on additional factors, including the patient's response to initial treatment, availability of a suitable donor for transplantation, and the patient's ability to tolerate the transplant procedure.  It is crucial for the patient to discuss their individual prognosis and treatment options with their healthcare team, including a hematologist or transplant specialist. They will consider the specific characteristics of the disease, the patient's overall health, and available treatment options to provide a more accurate prognosis and discuss the potential benefits and risks of allogeneic HSCT in this particular case. | The prognosis for a patient with acute myeloid leukemia (AML) who is offered allogeneic hematopoietic stem cell transplantation (HSCT) is influenced by several factors, including the specific genetic abnormalities present in the disease. The karyotype abnormalities you mentioned are t(1,2) and -7.  The t(1,2) translocation refers to the exchange of genetic material between chromosomes 1 and 2. This specific translocation is relatively rare in AML, and its prognostic significance is not well-established. The impact on prognosis may vary depending on the specific genes involved in the translocation.  The presence of a monosomy 7 (-7), where one copy of chromosome 7 is missing, is a known unfavorable cytogenetic abnormality in AML. Monosomy 7 is associated with a higher risk of treatment resistance and poorer outcomes, including higher rates of relapse and reduced overall survival.  It's important to note that prognosis is influenced by multiple factors beyond cytogenetics alone, including age, performance status, response to induction chemotherapy, presence of minimal residual disease, and the availability of a suitable donor for HSCT.  Considering the unfavorable cytogenetic abnormality of monosomy 7 in this patient's karyotype, the prognosis at 5 years is generally considered to be relatively poor. However, it's essential to consult with a hematologist or oncologist who can provide a more accurate prognosis based on the patient's specific clinical characteristics, including additional risk factors and response to therapy.  Understanding the individual patient's prognosis can help guide treatment decisions and discussions regarding the potential benefits and risks of allogeneic HSCT. | The prognosis of a patient with acute myeloid leukemia (AML) who undergoes allogeneic hematopoietic stem cell transplantation (HSCT) can be influenced by various factors, including the patient's age, overall health, disease characteristics, and specific cytogenetic abnormalities.  The specific karyotype alterations you mentioned in the patient's AML diagnosis, t(1,2) and -7, have important prognostic implications:  1. t(1,2) translocation: This specific translocation between chromosomes 1 and 2 is relatively rare in AML, and its prognostic significance is not well-established. The impact on prognosis can vary depending on other concurrent cytogenetic abnormalities, molecular mutations, and additional factors.  2. -7 deletion: Loss of the entire chromosome 7, known as monosomy 7 or -7, is associated with an adverse prognosis in AML. It is considered a high-risk cytogenetic abnormality.  Considering the unfavorable prognosis associated with the -7 deletion, the overall prognosis for a patient with AML harboring both t(1,2) and -7 cytogenetic abnormalities is generally considered poor.  However, it's important to note that the prognosis is influenced by multiple factors, and individual patient outcomes can vary. Prognostic scores, such as the European LeukemiaNet (ELN) risk stratification, consider additional factors such as molecular mutations (e.g., FLT3-ITD, NPM1, etc.), patient age, and response to induction chemotherapy to provide a more comprehensive prognostic assessment.  In general, the 5-year prognosis for patients with adverse-risk AML, which includes unfavorable cytogenetics such as -7 deletion, is relatively lower compared to those with favorable or intermediate-risk disease. The specific prognosis for an individual patient can only be determined by considering all relevant clinical and genetic factors, in consultation with a hematologist or oncologist experienced in AML management. |
| C17 | The condition described, including rapidly progressive ascites, weight gain, increased bilirubin level, and painful hepatomegaly, is suggestive of hepatic veno-occlusive disease (VOD), also known as sinusoidal obstruction syndrome (SOS). Several transplant-related factors have been suggested to increase the risk of developing VOD/SOS after allogeneic hematopoietic stem cell transplantation (HSCT). These factors include:  1. Conditioning regimen: Intensive conditioning regimens that involve high-dose chemotherapy and/or radiation therapy before HSCT have been associated with an increased risk of VOD/SOS. The intensity and toxicity of the conditioning regimen can contribute to liver damage and the development of VOD/SOS.  2. Total body irradiation (TBI): The use of TBI as part of the conditioning regimen has been identified as a risk factor for VOD/SOS. TBI can cause direct injury to the liver endothelial cells, leading to the development of VOD/SOS.  3. Older age: Advanced age, particularly over 50 years, has been identified as a risk factor for the development of VOD/SOS after HSCT.  4. Graft-versus-host disease (GVHD): The occurrence and severity of acute or chronic GVHD have been associated with an increased risk of VOD/SOS. GVHD involves an immune-mediated attack on the recipient's tissues, including the liver, which can contribute to the development of VOD/SOS.  5. Type of donor and graft source: Some studies suggest that the use of peripheral blood stem cells (PBSC) as a graft source, as well as the use of unrelated or mismatched donors, may increase the risk of VOD/SOS. However, the association between graft source and VOD/SOS is still a topic of ongoing research and debate.  It's important to note that the presence of these risk factors does not guarantee the development of VOD/SOS, and the specific risk can vary among individuals. Management of VOD/SOS involves supportive care, early detection, and interventions such as diuretics, fluid management, pain control, and, in severe cases, specific treatments such as defibrotide or liver-directed therapies.  The assessment and management of VOD/SOS should be performed by a healthcare professional experienced in managing HSCT complications, such as a hematologist or transplant specialist, in order to provide appropriate care tailored to the individual patient's situation. | The condition described, with rapidly progressive ascites, weight gain, increased bilirubin levels, and painful hepatomegaly, is suggestive of hepatic veno-occlusive disease (VOD), also known as sinusoidal obstruction syndrome (SOS). Several transplant-related factors have been suggested to increase the risk for this condition. These factors include:  1. Conditioning regimen: Certain conditioning regimens used before hematopoietic stem cell transplantation (HSCT) can contribute to an increased risk of VOD. High-intensity conditioning regimens, particularly those involving total body irradiation (TBI) and high-dose alkylating agents, have been associated with a higher incidence of VOD.  2. Stem cell source: The use of certain stem cell sources, such as hematopoietic stem cells obtained from unrelated donors or cord blood, has been linked to an increased risk of VOD. This may be due to differences in HLA mismatch or cell dose.  3. Graft-versus-host disease (GVHD) prophylaxis: The type and intensity of GVHD prophylaxis medications, such as calcineurin inhibitors (e.g., cyclosporine, tacrolimus), methotrexate, or other immunosuppressive agents, may affect the risk of VOD. Cyclosporine, in particular, has been associated with an increased risk of VOD when used as part of GVHD prophylaxis.  4. History of liver disease: Pre-existing liver disease, such as viral hepatitis or non-alcoholic fatty liver disease, can increase the risk of VOD following HSCT.  5. Previous radiation therapy: Prior abdominal or hepatic radiation therapy can predispose patients to an increased risk of VOD.  It's important to note that the development of VOD can also be influenced by individual patient factors, such as age, underlying comorbidities, and genetic susceptibility. The diagnosis and management of VOD require careful evaluation by a healthcare professional experienced in HSCT and transplant-related complications.  Early recognition and prompt management of VOD are crucial for optimizing patient outcomes. Supportive care measures, such as fluid management, diuretics, and pain control, are typically utilized. In severe cases, interventions like defibrotide may be considered. | The condition described, characterized by rapidly progressive ascites, weight gain, increased bilirubin level, and painful hepatomegaly in a patient undergoing allogeneic hematopoietic stem cell transplantation (HSCT), is suggestive of hepatic veno-occlusive disease (VOD), also known as sinusoidal obstruction syndrome (SOS). Several transplant-related factors have been suggested to increase the risk of developing this condition. These factors include:  1. Conditioning regimen: Intensive conditioning regimens that involve high-dose chemotherapy or radiation therapy prior to HSCT have been associated with an increased risk of VOD/SOS.  2. Busulfan-based regimens: The use of busulfan, particularly in combination with other chemotherapeutic agents, has been identified as a potential risk factor for the development of VOD/SOS.  3. Total body irradiation (TBI): TBI, often used as part of the conditioning regimen in HSCT, has been associated with an increased risk of VOD/SOS, especially when combined with other hepatotoxic agents.  4. Pre-existing liver disease: Patients with pre-existing liver disease, such as viral hepatitis or non-alcoholic fatty liver disease, may have an increased risk of developing VOD/SOS after HSCT.  5. Graft-versus-host disease (GVHD) prophylaxis: The use of certain immunosuppressive medications, such as calcineurin inhibitors (e.g., cyclosporine, tacrolimus), as part of GVHD prophylaxis has been linked to an increased risk of VOD/SOS.  It's important to note that the occurrence of VOD/SOS can also be influenced by individual patient factors, including age, underlying medical conditions, and genetic susceptibility. The presence of multiple risk factors may further increase the likelihood of developing VOD/SOS.  Early recognition and management of VOD/SOS are crucial to optimize patient outcomes. If VOD/SOS is suspected, prompt diagnostic evaluation and supportive care measures, such as fluid management and pain control, should be initiated. Specific interventions, such as defibrotide, may be considered in severe or refractory cases.  Consultation with a hematologist or transplant specialist experienced in managing HSCT-related complications is vital to evaluate and manage the patient's condition appropriately. |
| C18 | The dosing of donor lymphocyte infusion (DLI) in a patient depends on several factors, including the specific goals of the therapy, the underlying disease, the patient's medical condition, and the transplant team's recommendations. Here are some key considerations that influence the dosing of DLI:  1. Underlying Disease: The dosing of DLI is influenced by the type and stage of the underlying disease being treated. DLI is commonly used as a therapeutic intervention in the treatment of relapsed or residual hematologic malignancies, such as leukemia, lymphoma, or multiple myeloma, following allogeneic stem cell transplantation. The dosing may be tailored based on the aggressiveness of the disease and the desired immune response.  2. Disease Status and Response: The disease status and response to prior treatments are important factors in determining the dosing of DLI. If the disease is in remission or has responded well to prior treatments, a lower dose of DLI may be sufficient to maintain or enhance the graft-versus-tumor (GVT) effect. In contrast, if there is active disease or a high tumor burden, a higher dose may be considered to induce a stronger GVT effect.  3. Graft-versus-Host Disease (GVHD) Risk: DLI carries the risk of inducing graft-versus-host disease, where the donor immune cells attack the recipient's tissues. The dosing of DLI needs to be carefully considered to balance the therapeutic effect against the risk of GVHD. Lower doses may be used initially to minimize the risk of severe GVHD, while higher doses may be considered in cases where a stronger immune response is desired despite the potential for increased GVHD risk.  4. Patient Characteristics: The patient's overall health, prior transplant history, and tolerance to immune therapies are taken into account when determining the DLI dose. Factors such as age, comorbidities, previous complications, and organ function can influence the recommended dosing and treatment approach.  5. DLI Protocol and Experience: The specific DLI protocol followed by the transplant center, along with the expertise and experience of the transplant team, can influence the dosing recommendations. Different centers may have variations in dosing strategies based on their clinical experience and research findings.  It's important to note that the dosing of DLI should always be determined by the patient's healthcare team, including the transplant physician and/or oncologist, who will consider all relevant factors to develop an individualized treatment plan. The goal is to optimize the therapeutic benefit while minimizing potential risks and complications. | The dosing of donor lymphocyte infusion (DLI) depends on several factors that are taken into consideration by the healthcare team. These factors include:  1. Underlying Disease: The dosing of DLI can vary depending on the type of underlying disease being treated. DLI is commonly used in the treatment of relapsed or residual malignancies following allogeneic hematopoietic stem cell transplantation (HSCT). The specific disease and its characteristics, such as the aggressiveness and sensitivity to immune responses, can influence the dosing strategy.  2. Disease Status: The disease status of the patient, including the extent of relapse or residual disease, is an important consideration. Higher disease burden or aggressive disease may warrant a higher dose of DLI to achieve a more potent antitumor effect.  3. Previous Treatments: The previous treatments the patient has received, such as chemotherapy or radiation therapy, can impact the dosing of DLI. These treatments may have influenced the patient's immune system and the ability of the infused lymphocytes to exert their desired effects.  4. Donor Characteristics: The characteristics of the donor, including their HLA match with the recipient, the quantity and quality of the collected lymphocytes, and the presence of specific immune markers, can influence the dosing of DLI. A closer HLA match and higher number of viable lymphocytes may allow for a lower dose of DLI to be effective.  5. Patient Factors: Individual patient factors, such as age, overall health, and tolerance to potential side effects, are considered when determining the dosing of DLI. Some patients may be more susceptible to graft-versus-host disease (GVHD), a potential complication of DLI, and may require lower doses or additional prophylactic measures to manage GVHD risk.  The dosing of DLI is typically determined by the treating healthcare team based on a comprehensive assessment of these factors, taking into account the specific goals of treatment and balancing the potential benefits with the associated risks. The team will consider the patient's unique circumstances and develop an individualized dosing strategy for the DLI infusion. | The dosing of donor lymphocyte infusion (DLI) depends on several factors, which are evaluated by the healthcare team and tailored to the individual patient's condition. Some key factors that influence the dosing of DLI include:  1. Underlying Disease: The type of underlying disease or malignancy being treated plays a significant role in determining the DLI dosing. Different diseases may require different approaches and varying doses of donor lymphocytes. The specific disease characteristics, stage, and response to previous treatments are taken into account.  2. Disease Status: The disease status of the patient is crucial in determining the appropriate DLI dosing. The healthcare team assesses factors such as disease progression, minimal residual disease (MRD), or relapse to determine the intensity of the treatment required. Higher doses may be considered for more aggressive or advanced disease, while lower doses may be appropriate for disease control or maintenance.  3. Prior Treatments: The previous treatments received by the patient, including chemotherapy, radiation therapy, or prior stem cell transplantation, are considered when determining the DLI dosing. The cumulative effects of prior therapies on the patient's immune system and the response to those treatments guide the decision-making process.  4. Graft-versus-Host Disease (GVHD): The occurrence and severity of graft-versus-host disease are taken into account when determining the DLI dosing. In cases where the patient has a history of GVHD, the dose may be adjusted to balance the potential for graft-versus-tumor (GVT) effect with the risk of exacerbating GVHD.  5. Patient's Overall Health and Tolerance: The patient's overall health status, including organ function, performance status, and comorbidities, are evaluated to determine the appropriate DLI dosing. The goal is to administer a dose that maximizes the desired therapeutic effect while minimizing the risk of adverse effects.  6. Donor Characteristics: Donor factors, such as the availability and compatibility of the donor lymphocytes, are considered when determining the DLI dosing. The number and quality of collected lymphocytes from the donor play a role in deciding the appropriate dose to achieve the desired therapeutic outcome.  It's important to note that the dosing of DLI is a complex decision that requires expertise from the healthcare team, including the patient's hematologist/oncologist or transplant specialist. They will consider all relevant factors and tailor the DLI dosing to optimize treatment outcomes while minimizing potential risks. |

| **Chat-GPT4** | | | |
| --- | --- | --- | --- |
| **Section 1 – drug mechanism of action, toxicity, and interaction** | | | |
| D1 | Cyclosporine is an immunosuppressant medication often used to prevent organ rejection after transplantation. It can also be used for other conditions such as psoriasis and rheumatoid arthritis.  As with any medication, it can cause side effects, some of which are common while others are rare. Here are the common side effects:  1. **Gastrointestinal disturbances**: These can include nausea, vomiting, diarrhea, and abdominal discomfort or pain.  2. **Hypertension**: Elevated blood pressure is a common side effect of cyclosporine.  3. **Gum hyperplasia**: Overgrowth of the gums is a distinctive side effect of cyclosporine.  4. **Increased hair growth**: Unwanted and excessive hair growth (hirsutism) can occur.  5. **Kidney problems**: These can be manifested as changes in urination patterns, swelling in the legs, ankles, or feet, and potentially reduced kidney function. Kidney function should be regularly monitored while on this medication.  6. **Headache**: Some patients may experience headaches.  7. **Tremors**: These can occur as a result of the nervous system effects of cyclosporine.  Less common but more serious side effects can also occur, such as severe infection due to immunosuppression, liver problems, seizures, and serious allergic reactions. Because of the risk of these side effects, cyclosporine should be used under the supervision of a doctor who can monitor for these problems and adjust the dosage as needed. Always report any new or worsening symptoms to your doctor while taking cyclosporine. | Cyclosporine is an immunosuppressant medication that is often used to prevent organ rejection in transplant patients, or to treat certain autoimmune disorders. As with any medication, it can cause side effects. These can vary from person to person, but common side effects can include:  1. High blood pressure: This is one of the most common side effects. Regular monitoring of blood pressure is needed.  2. Kidney damage or dysfunction: Long-term use of cyclosporine can sometimes cause kidney problems. Regular monitoring of kidney function is necessary.  3. Gastrointestinal problems: These can include nausea, vomiting, diarrhea or constipation, and abdominal discomfort.  4. Tremors or shaking.  5. Hypertrichosis (excessive hair growth).  6. Gingival hyperplasia (gum overgrowth).  7. Headaches.  8. Increased risk of infections: Since cyclosporine suppresses the immune system, it can increase the risk of infections.  9. Changes in appearance or structure of the skin, such as acne, oily skin, or a rash.  10. Elevated cholesterol or triglyceride levels: Regular blood tests may be necessary to monitor these levels.  11. Fatigue or weakness.  Less commonly, cyclosporine can also cause more serious side effects, including an increased risk of certain types of cancer, particularly skin cancer and lymphoma. Remember that the decision to prescribe cyclosporine takes into account the potential benefits and risks, and it's important to discuss these with your doctor if cyclosporine is prescribed to you. Always take the medication as directed, and notify your healthcare provider if you experience any side effects. | Cyclosporine is an immunosuppressant medication that's often used to prevent organ rejection in people who've received a transplant. It's also used to treat severe rheumatoid arthritis, psoriasis, and certain other autoimmune disorders. As with any medication, cyclosporine can have side effects, although not everyone experiences them.  Common side effects of cyclosporine can include:  1. Headache  2. Diarrhea  3. Heartburn or indigestion  4. Increased hair growth on the body or face  5. Acne  6. Flushing or hot flashes  7. Shaking or trembling  8. Swollen or tender gums  9. Nausea or vomiting  10. Fatigue  11. Changes in blood pressure, which can be either high or low  12. Increased susceptibility to infections due to the immunosuppressive properties of the drug.  Less common, but potentially serious side effects include:  1. Kidney damage, as reflected by changes in urinary habits or unusual swelling.  2. Hypertension (high blood pressure)  3. Tremors or convulsions  4. Changes in vision or mental status  5. Signs of liver problems, such as yellowing of the skin or eyes (jaundice)  6. Unusual bleeding or bruising  It's very important to have regular check-ups and blood tests while taking cyclosporine, so your doctor can monitor your response to the drug and adjust the dosage as necessary. If you notice any new or worsening symptoms, you should report them to your doctor immediately. Remember, this list is not exhaustive, and individual experiences with the medication can vary.  It is important to remember that the information I provide is based on general knowledge as of September 2021, and individual medication's side effects may vary or change based on new research or specific formulations of the medication. Always consult your healthcare provider for the most accurate and up-to-date information. |
| D2 | Sirolimus, also known as rapamycin, is an immunosuppressive drug used to prevent rejection in organ transplantation, and is also used in a type of drug-eluting stent.  The primary target of sirolimus is a protein known as mTOR (mammalian target of rapamycin). mTOR is a key regulatory protein that controls cell growth, cell proliferation, protein synthesis, and transcription. By inhibiting mTOR, sirolimus suppresses the immune system and prevents the rapid growth and proliferation of T cells, which are a key part of the body's immune response.  More specifically, sirolimus binds to a protein called FKBP-12 to form a complex, and this complex then binds to mTOR to inhibit its activity. This mechanism is slightly different from that of other immunosuppressants like cyclosporine or tacrolimus, which also bind to FKBP-12 but inhibit a different pathway (the calcineurin pathway).  This mTOR inhibition effect of sirolimus has also led to its use in treating certain types of cancer, as the uncontrolled growth and proliferation of cells is a key feature of many cancers. However, its use in this context is still being researched. As always, if you have questions about this or any other medication, you should consult a healthcare provider. | Sirolimus, also known as rapamycin, is an immunosuppressant drug used to prevent organ rejection in patients who have received a kidney transplant. It is also used in certain other clinical contexts, including rare lung diseases and as a coating for coronary stents.  The target of sirolimus is a protein called mammalian target of rapamycin (mTOR). mTOR is a key regulatory kinase, which is a type of enzyme that modifies other proteins by chemically adding phosphate groups to them. mTOR plays a crucial role in regulating cell growth, proliferation, and survival.  Sirolimus binds to a protein called FKBP-12 in the cell, and this sirolimus-FKBP-12 complex then binds to mTOR. This binding inhibits the activity of mTOR, which has a cascade of effects within the cell. It reduces the cell's response to growth factors and limits its ability to synthesize proteins, ultimately suppressing the immune response.  By inhibiting mTOR, sirolimus reduces the immune system's ability to respond to foreign substances, including a transplanted organ. This helps to prevent the immune system from rejecting the transplanted organ.  However, because sirolimus suppresses the immune system, it can increase the risk of infections and certain other diseases. It can also have other side effects, and its use needs to be carefully monitored by a healthcare provider. | Sirolimus, also known as rapamycin, is an immunosuppressant drug used to prevent rejection in organ transplantation, and is especially useful in kidney transplants. It also has uses in a number of other conditions, including certain types of cancer and a rare lung disease called lymphangioleiomyomatosis.  The target of sirolimus is a protein called mammalian target of rapamycin (mTOR). mTOR is a part of cellular pathways that regulate cell growth, cell proliferation, protein synthesis, and autophagy, among other processes. It plays a key role in the function of T cells, which are an important part of the immune response.  Sirolimus binds to an intracellular protein called FKBP-12 to form a complex, and this sirolimus-FKBP-12 complex then binds to mTOR. This binding inhibits the activity of mTOR, leading to a decrease in protein synthesis and cell proliferation. As a result, the immune response is suppressed, reducing the risk of organ rejection.  It's important to note that while sirolimus suppresses the immune response, it can also have side effects and can increase susceptibility to infections and other diseases. As with any medication, it should be used under the supervision of a healthcare provider. |
| D3 | Voriconazole, an antifungal medication, is known to interact with several systemic immunosuppressant medications. Of these, it can significantly increase the serum levels of both cyclosporine and tacrolimus.  The mechanism underlying this interaction is that voriconazole inhibits certain cytochrome P450 enzymes (particularly CYP3A4), which are involved in the metabolism (breakdown) of both cyclosporine and tacrolimus. When voriconazole is co-administered with either of these immunosuppressants, it can slow the rate at which these drugs are metabolized and cleared from the body, leading to increased serum levels. This can potentially enhance their effects, but can also increase the risk of side effects, including nephrotoxicity (kidney damage) and neurotoxicity (damage to the nervous system).  As a result, if voriconazole needs to be co-administered with cyclosporine or tacrolimus, careful monitoring of blood levels of these immunosuppressants is needed, and dosage adjustments may be necessary.  This is a good example of why it's so important to inform your healthcare provider of all medications you're taking, including over-the-counter drugs and supplements, as they can interact with each other and affect how they work in your body. | Voriconazole is known to increase the serum levels of several immunosuppressant drugs, most notably cyclosporine, tacrolimus, and sirolimus. This is because voriconazole inhibits cytochrome P450 enzymes in the liver, which are involved in the metabolism of these drugs. As a result, the concentration of these immunosuppressants in the blood can rise, potentially leading to increased side effects or toxicity.  The interaction between voriconazole and these immunosuppressants is clinically significant. If a patient is receiving one of these immunosuppressants and starts taking voriconazole, the dose of the immunosuppressant might need to be reduced. Close monitoring of immunosuppressant drug levels, as well as monitoring for signs of immunosuppressant-related toxicity, is recommended when these drugs are used together.  As always, this information is intended to provide a general understanding and may not apply to every individual situation. Any medication changes or interactions should be managed by a healthcare provider. | Voriconazole, an antifungal medication, is known to increase the serum level of cyclosporine, a systemic immunosuppressant. Voriconazole inhibits the liver enzyme cytochrome P450 3A4 (CYP3A4), which is involved in the metabolism of cyclosporine. By inhibiting this enzyme, voriconazole reduces the breakdown and increases the serum level of cyclosporine.  Voriconazole may also increase the serum levels of other immunosuppressants that are metabolized by CYP3A4, such as tacrolimus and sirolimus. Therefore, when voriconazole is co-administered with these drugs, the doses of the immunosuppressants may need to be adjusted, and their levels should be closely monitored to avoid toxicity.  Always consult with a healthcare provider when taking multiple medications, as drug interactions can lead to serious side effects. |
| D4 | Calcineurin inhibitors, such as cyclosporine and tacrolimus, are commonly used in the prophylaxis of graft-versus-host disease (GVHD), a complication that can occur after an allogeneic tissue transplant (usually stem cell or bone marrow transplant). They work by inhibiting T-cell activation, thus suppressing the immune response that leads to GVHD.  These calcineurin inhibitors are often combined with other immunosuppressive drugs to enhance their effectiveness. These may include:  1. **Methotrexate**: This is a common choice to combine with a calcineurin inhibitor for GVHD prophylaxis. Methotrexate inhibits the metabolism of folic acid, which slows down the replication of cells, including those of the immune system.  2. **Mycophenolate mofetil (MMF)**: Mycophenolate mofetil inhibits the synthesis of DNA and RNA in cells, which leads to a decrease in the proliferation of B and T cells. It is often used in combination with a calcineurin inhibitor, particularly tacrolimus.  3. **Sirolimus (Rapamycin)**: This drug inhibits the response to interleukin-2 and blocks activation of T and B cells. It is sometimes used in combination with a calcineurin inhibitor.  4. **Antithymocyte globulin (ATG)**: This is a mixture of antibodies that act against various cell surface antigens. It is used in some regimens to reduce the number of T cells in the body, thereby reducing the risk of GVHD.  These combinations help to reduce the risk of acute and chronic GVHD following transplantation. The choice of specific drug combinations and their dosages would depend on the specific clinical situation, the patient's overall health status, the specific type and source of the graft, and institutional practices, among other factors.  As always, the information provided is as of the latest updates available till September 2021 and may change based on new research and guidelines. Always refer to a healthcare provider for accurate information. | Graft-versus-host disease (GVHD) is a common complication of allogeneic stem cell transplantation. It occurs when donor immune cells, usually T cells, recognize the recipient's cells as foreign and initiate an immune response against them. Calcineurin inhibitors, such as cyclosporine and tacrolimus, are often used in GVHD prophylaxis because they inhibit T cell activation.  In addition to calcineurin inhibitors, the following drugs are often included in GVHD prophylaxis regimens:  1. Methotrexate: This is a chemotherapy drug that inhibits the growth of rapidly dividing cells, such as immune cells. It's often given on certain days after transplantation.  2. Mycophenolate mofetil (MMF): This is an immunosuppressive drug that inhibits the proliferation of B and T lymphocytes, thereby reducing the immune response against the recipient's cells.  3. Sirolimus (Rapamycin): This drug inhibits the mammalian target of rapamycin (mTOR), a key regulator of cell growth and proliferation. By inhibiting mTOR, sirolimus suppresses T cell activation and proliferation.  4. Antithymocyte globulin (ATG): This is a polyclonal antibody that targets various cell surface antigens, leading to the depletion of T cells. ATG is often used in patients at high risk of developing GVHD.  5. Corticosteroids: These are also used for their potent immunosuppressive and anti-inflammatory effects.  These medications can be used in different combinations and dosages depending on the specific patient and transplant characteristics. The choice of prophylaxis regimen depends on several factors, including the type of transplant, the underlying disease, the degree of HLA match between donor and recipient, the recipient's age and overall health, and the risk of GVHD.  Please note that while these medications can reduce the risk of GVHD, they also suppress the immune system and can increase the risk of infections and other complications. Therefore, patients receiving these drugs are closely monitored by their healthcare team. As with any treatment, it's important to discuss the potential risks and benefits with your healthcare provider. | Graft-versus-host disease (GVHD) is a serious complication that can occur after a stem cell or bone marrow transplant. In GVHD, the donated bone marrow or peripheral blood stem cells view the recipient's body as foreign and attack the body.  Calcineurin inhibitors, like cyclosporine and tacrolimus, are commonly used in prophylaxis regimens for GVHD due to their immunosuppressive effects. They work by inhibiting the activity of T cells, a type of white blood cell that plays a key role in the immune response.  In many cases, these calcineurin inhibitors are used in combination with other drugs to enhance the prophylaxis effect. Here are some common combinations:  1. **Methotrexate**: When used in combination with a calcineurin inhibitor (usually cyclosporine), methotrexate can help to prevent acute GVHD. Methotrexate is an antimetabolite that interferes with the growth of certain cells in the body, particularly rapidly replicating cells like immune cells.  2. **Mycophenolate mofetil (MMF)**: MMF is often combined with tacrolimus for GVHD prophylaxis. MMF suppresses the immune system by inhibiting an enzyme necessary for DNA synthesis in immune cells, thereby preventing these cells from proliferating.  3. **Antithymocyte globulin (ATG)**: ATG is used in some prophylaxis regimens to reduce the number of T cells, which can lower the risk of developing GVHD.  4. **Sirolimus**: Sirolimus is sometimes used with tacrolimus as part of the prophylaxis regimen against GVHD. Sirolimus inhibits the response of the immune system by blocking the effects of a certain protein that helps the body's immune cells to grow and divide.  5. **Corticosteroids**, like prednisone, are also sometimes used in combination with these other drugs, particularly in patients who are at high risk for GVHD.  The exact regimen chosen often depends on various factors including the specific type of transplant, the disease being treated, the patient's overall health, the presence of other risk factors for GVHD, and the physician's preference. Monitoring is performed closely in these patients due to the high risk of infection given the significant immunosuppression. |
| D5 | Busulfan is a chemotherapy drug that's used primarily in the conditioning regimen prior to hematopoietic stem cell transplantation. It works by cross-linking DNA, which prevents cancer cells from dividing and growing.  Busulfan is primarily metabolized in the liver, where it undergoes a process known as conjugation. The key enzyme involved in this process is glutathione S-transferase (GST), which catalyzes the conjugation of busulfan with glutathione, a molecule that helps to detoxify drugs and other foreign substances. This reaction results in a busulfan-glutathione conjugate, which is less toxic and more easily excreted from the body.  The metabolites of busulfan are primarily excreted in urine, but small amounts may also be excreted in the feces. Some of the drug may also undergo enterohepatic recirculation, in which it is excreted into the bile, reabsorbed from the intestines, and then returned to the liver for further metabolism.  Because busulfan is metabolized in the liver, diseases or conditions that affect liver function can potentially impact the metabolism and clearance of busulfan. Similarly, other drugs that are metabolized by GST or that affect the availability of glutathione could potentially interact with busulfan and affect its metabolism.  It's also worth noting that the metabolism of busulfan can be highly variable among different individuals, which can influence the drug's efficacy and toxicity. Because of this, therapeutic drug monitoring is often used to adjust the dose of busulfan based on individual patient characteristics. As always, the use of busulfan should be supervised by a healthcare provider. | Busulfan is a chemotherapy medication that's often used in the conditioning regimen before hematopoietic stem cell transplantation. It works by cross-linking DNA, which prevents cancer cells from dividing and growing.  Busulfan is extensively metabolized in the liver. The primary pathway for busulfan metabolism is via conjugation with glutathione, a naturally occurring antioxidant in the body. This reaction is catalyzed by the enzyme glutathione S-transferase (GST). The primary metabolite of this reaction is a compound called tetrahydrothiophene (THT), which is excreted in the urine. Some minor pathways for busulfan metabolism also exist, including oxidation and hydrolysis reactions.  Notably, the pharmacokinetics of busulfan can be highly variable among different individuals, partly due to variations in the activity of the GST enzyme. This can affect the drug's toxicity and effectiveness. Therefore, the dose of busulfan is often adjusted based on therapeutic drug monitoring, where blood levels of the drug are measured to ensure they are within the desired range.  It's also worth mentioning that busulfan can have significant side effects and toxicities, including bone marrow suppression, liver damage (veno-occlusive disease), and pulmonary toxicity, among others. Therefore, it's important for patients receiving this drug to be closely monitored by their healthcare team. | Busulfan is a chemotherapy drug that is often used in the conditioning regimen before hematopoietic stem cell transplantation. It works by forming DNA crosslinks that inhibit DNA replication and RNA transcription, leading to cell death.  The metabolism of busulfan primarily takes place in the liver. Its primary metabolic pathway involves conjugation with glutathione, which is facilitated by glutathione S-transferase (GST) enzymes. The resulting metabolite is then further processed and ultimately excreted in the urine.  It's worth noting that the metabolism of busulfan can be influenced by individual patient factors. For instance, genetic variations in the GST enzyme can influence how effectively a person metabolizes busulfan. Also, certain drugs can affect busulfan metabolism by influencing the activity of GST or other enzymes involved in its metabolic pathway.  Because of the potential for variability in busulfan metabolism, therapeutic drug monitoring (measuring drug concentration in the blood) is often used to adjust the busulfan dose and achieve an optimal balance between efficacy and toxicity. |
| D6 | Prophylaxis for Pneumocystis jirovecii pneumonia (PCP) in hematopoietic stem cell transplant (HSCT) patients typically includes the use of trimethoprim-sulfamethoxazole (TMP/SMX), also known as co-trimoxazole.  The usual dosage is TMP 160 mg/SMX 800 mg one tablet daily or three times weekly. This is typically started 7 to 14 days post-transplant and continued for at least 6 months to a year, depending on the patient's risk factors and immune status.  Please note that treatment plans are individualized to each patient, and there may be variations in practice based on factors such as patient age, underlying medical conditions, and local guidelines or protocols. Always consult with a healthcare professional for specific medical advice. | Trimethoprim-sulfamethoxazole (TMP/SMX), also known as co-trimoxazole, is commonly used for Pneumocystis jiroveci pneumonia (PCP) prophylaxis in immunocompromised patients, such as those undergoing hematopoietic stem cell transplantation (HSCT).  The recommended dosing regimen for TMP/SMX for PCP prophylaxis in HSCT recipients, based on the guidelines from before my last update in September 2021, is typically one double-strength tablet (160/800 mg of TMP/SMX respectively) orally per day or three times per week. The three times per week regimen might be preferred in patients with high risk for adverse effects, especially renal toxicity.  It's important to consult with a healthcare provider or a pharmacist for the most accurate and current information as guidelines may have been updated since my last training data in September 2021. The healthcare provider will take into account the patient's overall health, potential for drug interactions, and the current best practices in the field. | TMP/SMX (trimethoprim/sulfamethoxazole), also known as Bactrim, is frequently used to prevent Pneumocystis jirovecii pneumonia (PCP), a serious opportunistic infection, in patients undergoing hematopoietic stem cell transplantation (HSCT).  While the exact dosage may vary based on individual patient characteristics and the specific protocols used by different institutions, a common prophylactic regimen is to administer TMP/SMX three times a week, usually on Monday, Wednesday, and Friday. This is often continued for 6-12 months post-transplant, or until immune reconstitution.  +  For example, a common regimen might be TMP 80 mg/SMX 400 mg (one double-strength tablet) taken orally three times a week.  However, it is important to consult with the treating physician or medical team for personalized dosing recommendations, as this can depend on the individual patient's condition, the specifics of their transplant, and their tolerance of the medication. Some patients may require adjustments due to renal function, weight, or side effects. Additionally, alternative medications may be considered for patients who cannot tolerate TMP/SMX.  This information should be used as a general guideline and not as a replacement for professional medical advice. Always consult a healthcare provider for the appropriate treatment. |
| D7 | The drug you're likely referring to is Ruxolitinib, which is a Janus kinase (JAK) 1/2 inhibitor. The FDA approved Ruxolitinib in 2019 for the treatment of steroid-refractory acute graft-versus-host disease (GVHD) in adult and pediatric patients 12 years and older.  Aside from acute GVHD, Ruxolitinib is also commonly used in the treatment of:  1. **Myelofibrosis**: Ruxolitinib was first approved by the FDA in 2011 for the treatment of intermediate or high-risk myelofibrosis, a type of bone marrow cancer that disrupts the body's normal production of blood cells.  2. **Polycythemia Vera**: Ruxolitinib is also approved for the treatment of polycythemia vera in patients who have had an inadequate response to or are intolerant of hydroxyurea. Polycythemia vera is a slow-growing blood cancer where the bone marrow makes too many red blood cells, which can cause the blood to thicken and lead to other complications.  Please consult with a healthcare provider for accurate information as my training data only goes up until September 2021, and there might be updates or changes in indications and usage beyond this time. | The drug you're referring to is likely ruxolitinib, a Janus kinase (JAK) 1/2 inhibitor. Ruxolitinib was approved by the FDA in 2019 for the treatment of steroid-resistant acute graft-versus-host disease (GVHD) in adults and children 12 years and older.  In addition to its use in GVHD, ruxolitinib is also commonly used for the treatment of myelofibrosis, a rare type of bone marrow cancer that disrupts the body's normal production of blood cells. It's also approved for the treatment of polycythemia vera, a slow-growing blood cancer in which your bone marrow makes too many red blood cells. Both conditions are part of a group of disorders known as myeloproliferative neoplasms.  Ruxolitinib works by blocking the activity of proteins called Janus kinases. These proteins play a key role in the signaling pathways that help to regulate the growth and development of blood cells. By blocking these proteins, ruxolitinib can help to reduce abnormal blood cell production in these diseases.  Keep in mind that ruxolitinib, like all medications, can have side effects, and it's important to discuss these with the healthcare provider. These can include anemia, thrombocytopenia (low platelet counts), bruising, dizziness, and infections, among others. It's also essential for patients taking ruxolitinib to have regular blood tests to monitor their blood cell counts. | The drug you're referring to is likely ruxolitinib, a Janus kinase (JAK) 1/2 inhibitor. The U.S. Food and Drug Administration (FDA) granted accelerated approval for ruxolitinib for the treatment of steroid-refractory acute graft-versus-host disease (GVHD) in adult and pediatric patients 12 years and older in 2019.  Besides GVHD, ruxolitinib is also commonly used in the treatment of myelofibrosis, a serious bone marrow disorder that disrupts the body's normal production of blood cells. It has also been approved for use in polycythemia vera, a slow-growing blood cancer in which the bone marrow makes too many red blood cells.  Ruxolitinib works by blocking certain enzymes in the body that affect blood cell production. By inhibiting these enzymes, it can help to reduce inflammation, slow or stop the abnormal growth of blood cells, and improve symptoms associated with these conditions.  As always, it's important to remember that any treatment approach should be under the supervision of a healthcare professional who can monitor the patient's response and adjust the treatment plan as necessary. It's also important to be aware of the potential side effects of ruxolitinib, which can include low blood counts, infection, and other complications. |
| D8 | A potential cause of lung injury following an allogeneic hematopoietic stem cell transplant (HSCT) could be the medication busulfan, which is commonly used as part of the conditioning regimen prior to transplantation. This phenomenon is often referred to as busulfan lung or busulfan-induced lung toxicity.  Busulfan is a chemotherapy medication that works by interfering with the growth of cancer cells. While it's effective at controlling disease, it can sometimes have side effects. Although relatively rare, one potential side effect is lung injury or pulmonary toxicity, which can lead to symptoms such as dry cough, shortness of breath, and fever. It can also lead to more serious complications such as pulmonary fibrosis or interstitial lung disease.  If busulfan-induced lung toxicity is suspected, the patient should be evaluated and treated by a healthcare professional. Management often involves discontinuing the drug (if still being administered) and providing supportive care. In severe cases, corticosteroids or other immunosuppressive medications may be used.  It's worth noting that other conditions can also cause lung complications following HSCT, such as idiopathic pneumonia syndrome (IPS), diffuse alveolar hemorrhage, and bronchiolitis obliterans syndrome, which can be a manifestation of chronic graft-versus-host disease. Therefore, a comprehensive evaluation is usually necessary to identify the underlying cause and guide treatment. | The condition you're referring to sounds like idiopathic pneumonia syndrome (IPS), a non-infectious complication that can occur after hematopoietic stem cell transplantation (HSCT). IPS includes a spectrum of lung injuries that can lead to symptoms such as dry cough, shortness of breath, and low oxygen levels.  One commonly used drug during the conditioning regimen for HSCT that's associated with lung injury is busulfan. Busulfan is a chemotherapy agent used to kill existing bone marrow cells and make room for the new stem cells to grow. However, it can have several side effects, including lung toxicity that can potentially lead to conditions such as IPS.  Another drug that is associated with lung injury is carmustine. It is a part of the BEAM regimen (BCNU or Carmustine, Etoposide, Cytarabine, and Melphalan) used before autologous stem cell transplantation, particularly in lymphomas.  These side effects are serious and should be managed promptly. If a patient develops symptoms of lung injury after HSCT, it's important to seek medical attention immediately. Management often involves supportive care, including oxygen and sometimes use of corticosteroids, along with discontinuation of the offending agent if possible.  It's worth mentioning that lung complications post-HSCT can also be due to other causes like infections, fluid overload, or GVHD among others, and a thorough evaluation should be performed in these cases. | Busulfan is a chemotherapy drug commonly used during the conditioning regimen before allogeneic hematopoietic stem cell transplantation (HSCT). It's associated with several potential side effects, including a risk of lung injury.  This lung injury can lead to a condition called "busulfan lung," or more generally "idiopathic pneumonia syndrome" (IPS), which is characterized by symptoms such as dry cough, shortness of breath, and fever. It's important to note that this condition is relatively rare but can be severe and even life-threatening.  If busulfan lung is suspected, the condition is typically managed by discontinuing busulfan (if still being administered) and initiating high-dose corticosteroids.  In some cases, other causes may also contribute to lung injury after HSCT, including radiation therapy and certain other medications, as well as infections or immune reactions such as graft-versus-host disease. Given the complexity of these potential causes, any new lung symptoms after HSCT should be evaluated promptly by a healthcare provider. |
| D9 | The clinical scenario you described suggests that the patient has invasive pulmonary aspergillosis, a common and serious fungal infection in immunocompromised individuals, such as those undergoing allogeneic hematopoietic stem cell transplantation (HSCT). The diagnosis is supported by the radiological findings, clinical symptoms, and a positive galactomannan test, which is a marker for Aspergillus infection.  The treatment for invasive aspergillosis often includes antifungal medications like voriconazole, which is likely the drug the patient is receiving. Voriconazole can cause electrolyte imbalances, including hypokalemia (low levels of potassium in the blood), as one of its side effects.  Standard dosing for voriconazole in adults typically starts with a loading dose of 6 mg/kg IV every 12 hours for the first 24 hours (two doses), followed by a maintenance dose of 4 mg/kg IV every 12 hours. This dosing can be adjusted based on individual patient factors and how well the patient is responding to therapy. Voriconazole levels in the blood are often monitored to ensure that the dose is appropriate and to avoid potential toxic effects.  However, the standard dose and route of administration might vary depending on a variety of factors, including the patient's age, weight, liver function, and the presence of other medications that might interact with voriconazole. Therefore, all treatment should be guided by a healthcare provider.  Lastly, it's important to monitor and treat the patient's hypokalemia, as severe hypokalemia can lead to serious complications, including cardiac arrhythmias. | The presence of a nodular lesion with perilesional ground glass changes, along with a positive galactomannan test from bronchoalveolar lavage, is highly suggestive of invasive aspergillosis, a serious fungal infection. The first-line antifungal therapy for invasive aspergillosis is often voriconazole.  Voriconazole can cause several side effects, one of which is hypokalemia (low potassium levels). This can occur due to voriconazole-induced enhancement of cortisol production, leading to increased urinary excretion of potassium.  The standard dosing regimen for voriconazole for the treatment of invasive aspergillosis in adults is as follows:  - Loading dose: 6 mg/kg IV every 12 hours for the first 24 hours.  - Maintenance dose: After the initial 24 hours, the dose is typically reduced to 4 mg/kg IV every 12 hours.  However, doses may be adjusted based on the patient's individual clinical status, response to therapy, and the presence of any side effects or drug interactions. Also, the use of voriconazole requires therapeutic drug monitoring due to its highly variable pharmacokinetics and narrow therapeutic index.  As always, it's important for the patient's healthcare provider to monitor electrolyte levels and manage any abnormalities, like hypokalemia, that may arise during treatment. The patient's potassium levels can be corrected with supplements or adjustments to the diet, as recommended by the healthcare provider. | The patient's symptoms, CT scan findings, and positive galactomannan test from bronchoalveolar lavage suggest an infection with Aspergillus, a type of fungus. This is a common opportunistic infection in immunocompromised patients, such as those undergoing allogeneic hematopoietic stem cell transplantation (HSCT). The antifungal drug often used to treat invasive aspergillosis is voriconazole.  Voriconazole can cause hypokalemia, a condition characterized by low potassium levels in the blood. This can occur due to the effect of voriconazole on renal tubular function, which can result in increased urinary potassium loss.  The standard dosing for voriconazole in adults usually involves a loading dose of 6 mg/kg intravenously (IV) every 12 hours for the first 24 hours (two doses), followed by a maintenance dose of 4 mg/kg IV every 12 hours. However, voriconazole levels can vary significantly among individuals due to differences in metabolism, and therapeutic drug monitoring is often used to adjust the dose as needed.  It's important to monitor electrolytes in patients receiving voriconazole, and potassium supplementation may be needed in case of hypokalemia. As with any medication, voriconazole should be used under the supervision of a healthcare provider. |
| D10 | Cyclophosphamide is a drug that is commonly used in the conditioning regimen for hematopoietic stem cell transplantation (HSCT), and its dosing needs to be adjusted in patients with reduced kidney function, such as those with a creatinine clearance of 50 ml/min.  Cyclophosphamide is metabolized in the liver and then excreted by the kidneys. Impaired kidney function can therefore lead to increased levels of the drug and its metabolites, which can increase the risk of toxicity.  The exact dose adjustment depends on the specific patient and the degree of kidney impairment, and should be determined by the treating physician. In general, a dose reduction of around 25-50% is often recommended for patients with a creatinine clearance of 10-50 ml/min.  It's also worth noting that other drugs used in the conditioning regimen, such as busulfan and fludarabine, are primarily metabolized by the liver and don't typically require dose adjustment for kidney impairment. However, all patients undergoing HSCT should be closely monitored for potential side effects and complications, and dose adjustments should be made as needed based on individual patient characteristics and response to treatment. As always, treatment should be under the supervision of a healthcare provider. | The conditioning regimen prior to hematopoietic stem cell transplantation (HSCT) often involves high-dose chemotherapy and sometimes radiation therapy to destroy the patient's own bone marrow and immune cells, creating space for the new stem cells and reducing the risk of them being attacked by the patient's immune system.  One of the drugs often used in conditioning regimens is cyclophosphamide. Cyclophosphamide is a chemotherapy drug that's metabolized by the liver into its active form and then excreted by the kidneys. If a patient has decreased kidney function (indicated by a reduced creatinine clearance), this can potentially lead to a buildup of cyclophosphamide and increase the risk of toxicity.  As such, the dose of cyclophosphamide may need to be reduced in patients with decreased kidney function. The exact adjustment would depend on a variety of factors, including the patient's overall health, the severity of their kidney impairment, and the specifics of their treatment plan.  It's important to note that other drugs used in conditioning regimens, such as busulfan, are primarily metabolized by the liver, and their dosing might not need to be adjusted for kidney impairment. However, each patient's situation is unique, and these decisions should be made in consultation with a healthcare provider experienced in HSCT. | The chemotherapy drug that often needs dose adjustment based on renal function (creatinine clearance) in the context of a conditioning regimen for hematopoietic stem cell transplantation is busulfan.  Busulfan is a potent alkylating agent used as part of the conditioning regimen before allogeneic or autologous stem cell transplantation. It is often given in combination with other drugs, such as cyclophosphamide or fludarabine.  While busulfan is primarily metabolized by the liver, renal impairment can still potentially affect its clearance and toxicity profile. Therefore, in patients with renal impairment, close monitoring of busulfan levels and potentially dose adjustments may be necessary to ensure safe and effective treatment.  However, it's worth noting that the impact of renal function on busulfan pharmacokinetics is complex and may not be significant in all patients. This is why individualized dosing, based on therapeutic drug monitoring, is often used when administering busulfan.  Before starting any treatment, the patient's health status, including organ function (like kidney and liver), is thoroughly assessed to minimize potential complications. Always consult with a healthcare provider when managing these therapies. |
| D11 | Antithymocyte globulin (ATG) is a medication used to suppress the immune system and is often given before allogeneic hematopoietic stem cell transplantation (HSCT) to help prevent graft-versus-host disease (GVHD). However, ATG can cause several side effects, including infusion reactions which can sometimes be severe. These reactions may include fever, chills, low blood pressure, shortness of breath, and even more severe allergic reactions.  To help prevent or minimize these infusion reactions, patients are often given certain medications immediately prior to receiving ATG. These usually include:  1. **Corticosteroids** such as methylprednisolone: These medications help to suppress the immune system and reduce inflammation, which can help to prevent infusion reactions.  2. **Antipyretics** like acetaminophen (Tylenol): These medications can help to reduce fever, which is a common side effect of ATG.  3. **Antihistamines** such as diphenhydramine (Benadryl): These medications can help to prevent allergic reactions, including those caused by ATG.  4. Sometimes, **antiviral medications** (like acyclovir) are also given prophylactically as ATG increases the risk of certain viral infections.  These medications are generally given 30 minutes to an hour before the infusion of ATG to allow them time to take effect. As always, the decision to use these medications and the choice of which ones to use should be made by a healthcare provider based on the individual patient's circumstances. | Anti-thymocyte globulin (ATG) is a type of treatment used to suppress the immune system and prevent graft rejection or graft-versus-host disease in patients undergoing allogeneic hematopoietic stem cell transplantation (HSCT). However, because ATG is a rabbit or horse-derived product, its administration can cause an immune response that leads to infusion-related reactions.  To prevent or reduce the severity of these reactions, premedications are often given before the administration of ATG. These usually include:  1. **Corticosteroids** - Typically, methylprednisolone or hydrocortisone is used. Corticosteroids have anti-inflammatory properties that can help to reduce the severity of infusion-related reactions.  2. **Antihistamines** - Typically, diphenhydramine (Benadryl) or another similar drug is used. Antihistamines can help to reduce allergic reactions by blocking the action of histamine, a substance that's released during an allergic reaction.  3. **Antipyretics** - Acetaminophen (Tylenol) is often given to prevent or reduce fever.  4. **In some cases, an antipyretic agent like acetaminophen** is also used to prevent or manage fever associated with the infusion.  These medications are usually given 30 minutes to an hour before the ATG infusion to ensure they're effective by the time the infusion starts.  Please note that the choice of premedications, and their doses, can vary depending on several factors, including the patient's overall health, the specific ATG product being used, and the transplant center's protocol. Therefore, it's always important to follow the healthcare provider's instructions when it comes to these treatments. | Anti-thymocyte globulin (ATG) is often given as part of the conditioning regimen prior to allogeneic hematopoietic stem cell transplantation (HSCT) to reduce the risk of graft-versus-host disease (GVHD) and graft rejection.  However, ATG can cause various side effects, including fever, chills, rash, and low blood pressure. These are often related to what's known as a cytokine release syndrome, a systemic inflammatory response caused by the rapid release of cytokines from cells targeted by the ATG.  To minimize these reactions, several medications are commonly administered prior to ATG. These include:  1. **Antipyretics (Fever Reducers)**: Acetaminophen (Tylenol) is often given to help prevent or manage fever.  2. **Antihistamines**: Drugs such as diphenhydramine (Benadryl) are given to help prevent or manage allergic reactions, which can include rashes, itching, or hives.  3. **Corticosteroids**: Methylprednisolone or similar steroids can be administered to help manage the inflammatory response associated with cytokine release.  4. **Analgesics**: Pain relievers may be administered if necessary.  The specific regimen may vary depending on the specific patient characteristics and the medical center's protocols. In any case, it's important for the patient to be closely monitored during and after ATG administration to manage any side effects that occur promptly and effectively. |
| D12 | Fludarabine is a chemotherapy drug that is used to treat certain types of blood cancers such as chronic lymphocytic leukemia (CLL) and non-Hodgkin's lymphoma.  The plasma half-life of fludarabine, after intravenous administration, is approximately 20 minutes. However, it's metabolized to 2-F-ara-A, which has a longer half-life of about 20 hours.  Please note, the half-life may vary depending on individual factors such as the patient's renal function and overall health. Always consult with a healthcare provider for personalized treatment information. | Fludarabine, a chemotherapy medication used to treat certain types of blood cancers, has a plasma half-life of approximately 20 hours after intravenous administration.  However, it's important to note that the total clearance of fludarabine from the body may take longer due to its metabolism and distribution within the body. After administration, fludarabine is rapidly dephosphorylated to 2-fluoro-ara-A (fludarabine's active metabolite), which then gets converted back to the triphosphate form (2-fluoro-ara-ATP) within cells. This metabolite has a prolonged intracellular half-life, up to 20 hours or more, which contributes to its biological activity.  Always consult a healthcare provider or pharmacist for information specific to your situation or before starting any new medication. | Fludarabine is a chemotherapy medication used in the treatment of various hematological malignancies, including chronic lymphocytic leukemia and non-Hodgkin lymphoma. It is a purine analog that interferes with DNA synthesis, thus inhibiting the growth of rapidly dividing cells.  The plasma half-life of fludarabine, which represents the time it takes for the concentration of the drug in the body to be reduced by half, varies among individuals. However, it's generally reported to be in the range of approximately 20 hours when it's administered intravenously.  It's important to note that the actual half-life can be influenced by several factors, including the individual's metabolism, kidney and liver function, as well as other concurrent medications or treatments. Therefore, it's always recommended to consult with the treating physician or a pharmacist for personalized information about a specific medication's pharmacokinetics.  This information should not be used as a substitute for professional medical advice. Always consult with a healthcare provider. |
| D13 | Inotuzumab ozogamicin is an antibody-drug conjugate used in the treatment of acute lymphoblastic leukemia (ALL). The drug targets CD22, a protein expressed on the surface of most B-cell malignancies, and delivers a cytotoxic agent directly to the cancer cells.  A known and significant risk associated with inotuzumab ozogamicin treatment is veno-occlusive disease (VOD), also known as sinusoidal obstruction syndrome (SOS). This is a condition in which the small veins in the liver become obstructed, leading to liver damage. This risk is particularly high in patients who have received inotuzumab and are undergoing hematopoietic stem cell transplantation (HSCT).  VOD/SOS can be a serious and life-threatening condition, so strategies to minimize its risk are critical. These can include:  1. **Timing of HSCT**: It may be beneficial to allow for a longer interval between the last dose of inotuzumab and the start of the conditioning regimen for HSCT.  2. **Selection of Conditioning Regimen**: A reduced-intensity conditioning regimen may be chosen over a myeloablative conditioning regimen to decrease the risk of VOD.  3. **Prophylaxis with Defibrotide**: Defibrotide has been shown to decrease the incidence of VOD/SOS in high-risk patients undergoing HSCT. It is approved for the prevention of VOD in pediatric patients and for the treatment of severe VOD in adults and children.  4. **Careful Monitoring**: Close monitoring of liver function tests and quick action at the first signs of VOD/SOS can help to catch the condition early and start treatment promptly.  5. **Avoidance of Other Hepatotoxic Drugs**: If possible, other drugs that can cause liver damage should be avoided.  The specific strategies used may depend on the patient's overall health, liver function, and other factors. As always, decisions about treatment should be made in consultation with a healthcare provider. | Inotuzumab ozogamicin is an antibody-drug conjugate used in the treatment of acute lymphoblastic leukemia (ALL). One of the serious potential side effects of inotuzumab ozogamicin is veno-occlusive disease (VOD), also known as sinusoidal obstruction syndrome (SOS), particularly in patients who go on to have a hematopoietic stem cell transplant (HSCT). This condition involves the blockage of small veins in the liver, leading to liver damage.  Signs and symptoms of VOD/SOS include weight gain, ascites (build-up of fluid in the abdomen), hepatomegaly (enlarged liver), and jaundice. It can lead to multi-organ failure if not recognized and treated promptly.  To minimize the risk of VOD/SOS, a few strategies can be implemented:  1. **Close Monitoring**: Careful monitoring of liver function tests, weight, and fluid balance can help to detect VOD/SOS early.  2. **Prophylaxis**: The use of prophylactic medications such as defibrotide has been shown to reduce the incidence of VOD/SOS in high-risk patients undergoing HSCT.  3. **Avoidance of Additional Hepatotoxic Medications**: If possible, avoiding other medications known to cause liver damage can help to reduce the risk of VOD/SOS.  4. **Appropriate Timing**: If feasible, ensuring an adequate time interval between the last dose of inotuzumab and the start of the conditioning regimen for HSCT might reduce the risk of VOD/SOS.  5. **Hydration**: Adequate hydration before and during the conditioning regimen can also help protect the liver.  Despite these precautions, VOD/SOS can still occur and is a serious complication that needs immediate attention. Early detection and management can significantly improve the prognosis. As always, the patient should be managed by a team of healthcare professionals experienced in HSCT. | Inotuzumab ozogamicin is an antibody-drug conjugate used in the treatment of relapsed or refractory B-cell precursor acute lymphoblastic leukemia. A known complication associated with this drug is the increased risk of veno-occlusive disease (VOD), also known as sinusoidal obstruction syndrome (SOS), especially in patients going on to receive hematopoietic stem cell transplantation (HSCT).  VOD/SOS is a potentially life-threatening condition in which small veins in the liver become obstructed, leading to liver damage. Signs and symptoms can include rapid weight gain, ascites (fluid buildup in the abdomen), and jaundice (yellowing of the skin and eyes).  To minimize the risk of VOD/SOS, several strategies can be used:  1. **Timing of HSCT**: Some guidelines suggest waiting at least a few weeks between the last dose of inotuzumab ozogamicin and HSCT.  2. **Use of VOD/SOS prophylaxis**: In some cases, a drug called defibrotide may be used prophylactically in patients at high risk for VOD/SOS. Defibrotide is currently the only drug approved by the FDA for treatment of severe VOD/SOS with renal or pulmonary dysfunction post-HSCT, and there is emerging evidence for its prophylactic use.  3. **Avoidance of certain conditioning regimens**: Some conditioning regimens, particularly those containing high-dose alkylating agents or total body irradiation, may increase the risk of VOD/SOS. Using reduced-intensity conditioning or regimens without these components may be considered in some cases.  4. **Close monitoring**: Frequent monitoring of liver function tests and for signs and symptoms of VOD/SOS can help ensure early detection and treatment.  5. **Healthy lifestyle**: Avoiding alcohol, maintaining a healthy weight, and managing other risk factors for liver disease may also help reduce the risk of VOD/SOS.  Always, it is important to discuss this risk and potential interventions with the treating physician, as individual patient factors must be taken into consideration when planning treatment. |
| D14 | Rituximab is a monoclonal antibody that targets CD20, a protein found on the surface of B cells. It is commonly used to treat certain types of cancer and autoimmune diseases. However, one side effect of rituximab is that it can impair B-cell reconstitution, which can increase the risk of infections after allogeneic hematopoietic stem cell transplantation (HSCT).  To help reduce this risk, regular administration of intravenous immunoglobulin (IVIG) can be considered. IVIG is a blood product prepared from the serum of between thousands to tens of thousands of donors per batch. IVIG is mainly used as treatment in three major categories: immune deficiencies, autoimmune diseases and acute infections.  IVIG provides passive immunity by increasing the body's antibody level, thus helping to fight off infections. It is often used in patients who are immunocompromised or who have specific problems with their immune systems.  The dosage and frequency of IVIG administration can vary depending on the patient's specific situation and the underlying condition being treated. This treatment should be administered under the supervision of a healthcare provider, who can monitor the patient's response and adjust the treatment plan as necessary. It's also important to be aware of the potential side effects of IVIG, which can include fever, chills, headache, and in rare cases, more serious complications.  Furthermore, it's crucial to reinforce general infection prevention strategies, such as hand hygiene, safe food practices, and avoidance of exposure to people with contagious illnesses, as well as to maintain vaccinations up-to-date as advised by the healthcare provider. | Patients receiving rituximab, a monoclonal antibody that targets CD20+ B cells, may experience impaired B cell reconstitution, which can increase their risk of infections, especially those of viral and fungal origin. In addition to antimicrobial prophylaxis, patients may receive immunoglobulin replacement therapy to help prevent infections.  Intravenous immunoglobulin (IVIG) or subcutaneous immunoglobulin (SCIG) can be used for replacement therapy. These products contain a broad spectrum of antibodies that can help the patient fight off infections while their own immune system is recovering.  IVIG or SCIG are typically given every 3-4 weeks, with the goal of maintaining a trough immunoglobulin G (IgG) level above a certain threshold (usually around 500 mg/dL), which is believed to offer protection against infections. The exact dosing and interval may vary depending on the patient's specific situation, including their level of immunosuppression, their individual response to therapy, and their history of infections.  It's important to note that while immunoglobulin replacement can help reduce the risk of infections, it doesn't entirely eliminate it, and it doesn't replace the need for antimicrobial prophylaxis and other infection prevention measures. Patients should be closely monitored for signs of infection and treated promptly if an infection occurs.  As always, individual patient factors and the specific treatment plan should be discussed with the treating physician. | Patients who have received rituximab, a monoclonal antibody that targets the CD20 antigen on B cells, may have impaired B cell reconstitution following allogeneic hematopoietic stem cell transplantation (HSCT). This can lead to hypogammaglobulinemia, a condition characterized by low levels of immunoglobulins (antibodies), thereby increasing the patient's susceptibility to infections.  Apart from antimicrobial prophylaxis, one strategy to reduce the risk of infection in these patients is the administration of intravenous immunoglobulin (IVIG). IVIG is a blood product prepared from the serum of between thousands and millions of donors, depending on the specific product. It contains pooled IgG immunoglobulins (antibodies) that can boost the patient's immune response and help to protect against infections.  IVIG administration can be especially beneficial in patients with severe or recurrent infections, or in those with significantly low IgG levels. However, the decision to administer IVIG should be individualized, based on the patient's specific risk factors and clinical condition. Additionally, it's worth noting that while IVIG can help reduce the risk of infection, it cannot completely eliminate it, and close monitoring and appropriate antimicrobial prophylaxis remain important components of care.  As with any treatment, IVIG has potential side effects and risks that need to be considered, and its use should be managed by a healthcare professional. |
| D15 | The patient is likely receiving dasatinib. Dasatinib is a tyrosine kinase inhibitor used in the treatment of chronic myeloid leukemia (CML) and Philadelphia chromosome-positive (Ph+) acute lymphoblastic leukemia (ALL).  One of the common side effects of dasatinib is fluid retention, which can manifest in various ways, including periorbital edema (swelling around the eyes). Other potential manifestations of fluid retention include pleural effusion (fluid around the lungs), peripheral edema (swelling in the limbs), and, less commonly, pulmonary edema and ascites.  If a patient develops significant fluid retention, the treating physician may need to adjust the dasatinib dose, temporarily discontinue the medication, or treat the symptom directly, for example, with diuretics. It's always important to promptly report new or worsening side effects to the healthcare provider.  Please note that this is general information, and individual treatment decisions should always be discussed with a healthcare provider. | The patient is likely receiving dasatinib. Dasatinib is an oral tyrosine kinase inhibitor (TKI) commonly used in the treatment of Philadelphia chromosome-positive acute lymphoblastic leukemia (Ph+ ALL).  One of the known side effects of dasatinib is fluid retention, which can manifest as swelling around the eyes (periorbital edema), as well as in other parts of the body, including the legs (peripheral edema), lungs (pleural effusion), and even the heart (pericardial effusion). These side effects are typically managed by reducing the dose of dasatinib, temporarily interrupting therapy, or administering supportive treatments such as diuretics. In some cases, a switch to another TKI may be considered.  As always, it's important for any new symptoms to be evaluated by a healthcare provider, who can help determine the cause and appropriate treatment. The benefits and risks of any medication should be discussed with the patient, and the treatment plan should be individualized based on the patient's specific condition and response to therapy. | The patient is likely receiving a tyrosine kinase inhibitor called dasatinib. Dasatinib is commonly used in the treatment of Philadelphia chromosome-positive acute lymphoblastic leukemia (Ph+ ALL).  One of the common side effects of dasatinib is fluid retention, which can manifest as swelling around the eyes (periorbital edema), swelling of the legs (peripheral edema), pleural effusion (fluid around the lungs), and less commonly, ascites (abdominal fluid accumulation).  If a patient develops significant fluid retention, dasatinib dose modification or temporary interruption may be required, and supportive treatments like diuretics may be used. As with any medication, any side effects should be reported to a healthcare provider, who can provide appropriate advice and treatment. It's also important to continue monitoring response and adjusting treatment as needed, since these drugs play a crucial role in treating Ph+ ALL. |
| D16 | The conditioning regimen prior to stem cell transplantation often includes chemotherapy drugs, like busulfan and fludarabine, which are associated with significant nausea and vomiting.  To manage these side effects, a combination of antiemetic (anti-nausea) medications is usually given. This typically includes a 5-HT3 receptor antagonist, such as ondansetron, granisetron, or palonosetron, which are very effective for chemotherapy-induced nausea and vomiting.  Additional antiemetics, like a glucocorticoid (dexamethasone) and a neurokinin-1 receptor antagonist (aprepitant or fosaprepitant), are often added to this regimen, particularly for high emetogenic chemotherapy like busulfan.  This combination is intended to target different pathways involved in nausea and vomiting, thus providing more comprehensive control of these side effects. As with any treatment, the choice of antiemetics should be individualized based on the patient's specific risk factors and clinical condition.  It's also important to note that despite the use of these antiemetic drugs, some patients may still experience nausea and vomiting. In these cases, other strategies may be used, such as adjusting the antiemetic regimen, adding other types of antiemetics (like olanzapine), and providing supportive care.  All medications and treatment plans should be discussed with the healthcare team to ensure optimal care and management of side effects. | Busulfan and fludarabine are both medications used in the conditioning regimen prior to hematopoietic stem cell transplantation (HSCT). These medications can often cause nausea and vomiting, so antiemetic (anti-nausea) medications are typically given to help prevent or control these side effects.  For conditioning regimens that include busulfan, such as busulfan/fludarabine, the antiemetic regimen usually includes a combination of a serotonin (5-HT3) receptor antagonist, a neurokinin-1 receptor antagonist, and a corticosteroid.  Specifically, these might include:  1. A 5-HT3 antagonist, such as ondansetron (Zofran) or granisetron. These drugs work by blocking the action of serotonin, a chemical in the brain that can trigger nausea and vomiting.  2. A neurokinin-1 antagonist, such as aprepitant (Emend). These medications work by blocking the action of substance P/neurokinin 1, a neurotransmitter that can induce vomiting.  3. Dexamethasone, a type of corticosteroid, is often included in the antiemetic regimen as well.  This regimen is usually started before the administration of the conditioning chemotherapy and continued for a few days after to help prevent both acute and delayed chemotherapy-induced nausea and vomiting.  The choice of antiemetics may also be influenced by the patient's specific characteristics and other medications they are taking. As with any treatment, antiemetics should be given under the supervision of a healthcare provider, and any side effects should be reported and managed promptly. | Chemotherapy regimens for conditioning before hematopoietic stem cell transplantation (HSCT), such as busulfan and fludarabine, can cause nausea and vomiting. To prevent these symptoms, antiemetic (anti-nausea) drugs are typically given before and sometimes after the chemotherapy sessions.  The specific antiemetic regimen can vary, but it often includes a combination of the following types of medications:  1. **Serotonin (5-HT3) receptor antagonists**: These drugs, such as ondansetron (Zofran), granisetron, or palonosetron, are very effective at preventing nausea and vomiting, particularly during the acute phase (within the first 24 hours after chemotherapy).  2. **Neurokinin-1 (NK1) receptor antagonists**: These drugs, such as aprepitant (Emend) or fosaprepitant, can help prevent both acute and delayed nausea and vomiting, particularly when used in combination with a 5-HT3 receptor antagonist and a corticosteroid.  3. **Corticosteroids**: Dexamethasone is often used in combination with the other classes of drugs to help prevent nausea and vomiting.  4. **Benzodiazepines**: Although not a primary antiemetic, drugs like lorazepam can be used to reduce anxiety, which can indirectly help control nausea and vomiting.  The specific choice and combination of antiemetic drugs can depend on various factors, including the specific chemotherapy regimen, the patient's prior response to antiemetic drugs, the patient's preferences and overall health status, and other considerations. The antiemetic regimen should be administered under the supervision of a healthcare professional and adjusted as necessary based on the patient's response. |
| D17 | The most likely drug that could cause vivid dreams and visual hallucinations is voriconazole. It is a triazole antifungal medication used prophylactically in patients undergoing hematopoietic stem cell transplantation (HSCT) to prevent invasive fungal infections.  The mechanism of action of voriconazole involves the inhibition of fungal cytochrome P450-mediated 14 alpha-lanosterol demethylation, an essential step in fungal ergosterol biosynthesis. Ergosterol is a key component of the fungal cell membrane. When ergosterol is depleted, it results in an accumulation of 14 alpha-methyl sterols, which leads to alterations in the fungal cell membrane that inhibit growth and replication of the fungus.  Neurological side effects, including visual disturbances, hallucinations, and changes in perception, are well-documented with voriconazole use. If these side effects become bothersome or impact a patient's quality of life, alternative antifungal medications may need to be considered. As always, these decisions should be made in consultation with a healthcare provider. | The symptoms described - vivid dreams and visual hallucinations - are suggestive of neuropsychiatric side effects, which can be associated with the antimalarial drug mefloquine. Mefloquine is sometimes used for prophylaxis against Pneumocystis jirovecii pneumonia (PJP) in patients undergoing allogeneic hematopoietic stem cell transplantation (HSCT), particularly in those who cannot tolerate or have contraindications to more commonly used drugs such as trimethoprim-sulfamethoxazole.  Mefloquine works by inhibiting the replication of the P. jirovecii organism, which is a common opportunistic pathogen in immunocompromised patients. It interferes with the parasite's ability to detoxify heme, a byproduct of hemoglobin digestion. This leads to accumulation of toxic heme within the parasite, causing its death.  Neuropsychiatric side effects such as hallucinations, vivid dreams, insomnia, and anxiety are known potential side effects of mefloquine, though the exact mechanisms for these effects are not entirely understood. If these side effects occur, discontinuation of the drug usually leads to resolution of symptoms. Other alternatives for PJP prophylaxis can be considered, under the guidance of a healthcare provider. | The patient was likely taking voriconazole, an antifungal medication often used for prophylaxis in immunocompromised patients, such as those undergoing allogeneic hematopoietic stem cell transplantation (HSCT). One of the known side effects of voriconazole includes central nervous system effects such as vivid dreams and visual hallucinations.  Voriconazole is a triazole antifungal medication that works by inhibiting the fungal cytochrome P450 enzyme, specifically 14-alpha demethylase. This enzyme is necessary for the conversion of lanosterol to ergosterol, a critical component of the fungal cell membrane. By inhibiting this enzyme, voriconazole disrupts the production of ergosterol, leading to an accumulation of 14-alpha-methyl sterols. This change in the composition of the cell membrane impairs its function and leads to the inhibition of fungal growth.  As always, it's important to report any unusual or bothersome side effects to a healthcare provider, who can adjust the treatment plan as necessary. |
| D18 | The drug you're likely referring to is ibrutinib, a Bruton's tyrosine kinase (BTK) inhibitor that's commonly used in the treatment of chronic lymphocytic leukemia (CLL) and is also used as second-line therapy for chronic graft versus host disease (cGVHD).  Regarding the dental surgery, there are indeed concerns with ibrutinib because it can increase the risk of bleeding, including during surgeries. As per the guidelines from the American Society of Hematology published in 2018, it is generally recommended to hold ibrutinib for 3 to 7 days prior to and after minor procedures such as dental extraction.  However, the final decision on this matter must be made jointly by the hematologist/oncologist, the dentist or oral surgeon, and the patient, considering the specific circumstances, including the urgency of the dental procedure, the patient's overall health status, the severity of the GVHD, the risk of bleeding versus the risk of disease progression, among others.  In all cases, you should refer to the treating physician or medical team for the most appropriate advice. This information should be used as a general guideline and not as a replacement for professional medical advice. Always consult with a healthcare provider. | Based on your description, it appears the patient is being treated for chronic graft versus host disease (cGVHD) and the treatment involves a drug commonly used for chronic lymphocytic leukemia (CLL). Without the specific drug name, it's difficult to give an exact answer, but based on the context, it's possible the drug could be ibrutinib or another drug in the class of BTK inhibitors, which are increasingly being used in the management of cGVHD.  These medications can increase the risk of bleeding, which could potentially be an issue with dental surgery.  Current guidelines suggest that for minor procedures, like dental cleaning, ibrutinib can be continued. However, for more invasive procedures such as dental extractions or surgery, it may be necessary to hold the drug for 3-7 days before and after the procedure to reduce the risk of bleeding. It's critical to remember that each patient's case is unique, so these guidelines might not apply to every situation.  It's extremely important to discuss this with the healthcare team managing the patient's care, including the hematologist and the dentist or oral surgeon, to decide on the best course of action. They would have the most accurate and detailed information about the patient's condition, the specific medication they are taking, and the potential risks associated with surgery.  This information should not be used as a substitute for professional medical advice. Always consult with a healthcare provider. | The information you provided suggests the patient might be starting on a drug like ibrutinib, which is commonly used in the treatment of chronic lymphocytic leukemia and has also been used as second-line treatment for chronic graft versus host disease (cGVHD).  Ibrutinib is a Bruton's tyrosine kinase (BTK) inhibitor, and one of its side effects is an increased risk of bleeding. Therefore, it might need to be temporarily discontinued before surgical procedures, including dental surgery, to reduce the risk of excessive bleeding.  Guidelines regarding how many days before surgery ibrutinib should be stopped can vary, but generally, it's recommended to stop the medication 3-7 days prior to a planned surgical procedure. However, the timing depends on several factors including the specific procedure, the patient's overall health status, and the potential risk of bleeding versus the risk of the underlying disease progressing without the medication.  It's very important for the patient to discuss this situation with both their oncologist and dentist/surgeon, who can provide specific advice tailored to their individual circumstances.  This information should not be used as a substitute for professional medical advice. Always consult with a healthcare provider. |
| **Section 2 HSCT Platform** | | | |
| P1 | GVHD, or Graft-Versus-Host Disease, is a potential complication of allogeneic hematopoietic stem cell transplantation (HSCT), where the donor's immune cells attack the recipient's tissues, considering them as foreign. This can lead to a range of symptoms, from mild to severe, and can affect different parts of the body, including the skin, liver, and gastrointestinal tract.  GVHD prophylaxis refers to treatments given to prevent the development of GVHD after an allogeneic HSCT. The goal is to suppress the immune response of the transplanted cells against the recipient's tissues, while still preserving the beneficial graft-versus-leukemia (GVL) effect, where the donor's immune cells attack any remaining cancer cells.  The specific regimen for GVHD prophylaxis can vary, but common elements include:  1. **Immunosuppressant Medications**: Drugs like calcineurin inhibitors (cyclosporine or tacrolimus), and an antimetabolite (methotrexate or mycophenolate mofetil) are commonly used. These medications work by suppressing the activity of T cells, which are the main immune cells responsible for GVHD.  2. **Anti-Thymocyte Globulin (ATG)**: ATG is a type of immunosuppressant medication that can be used to further reduce the risk of GVHD, particularly in patients receiving stem cells from unrelated or mismatched donors. ATG works by targeting and depleting T cells.  3. **Post-Transplant Cyclophosphamide (PTCy)**: In some cases, high-dose cyclophosphamide is given after transplantation to prevent GVHD, particularly in haploidentical (half-matched) transplantations. This drug also targets and kills rapidly dividing T cells.  The choice of GVHD prophylaxis can depend on various factors, including the type of transplant, the degree of matching between donor and recipient, the underlying disease, the patient's overall health, and other considerations. While GVHD prophylaxis can reduce the risk of GVHD, it can't entirely eliminate it, and close monitoring for signs and symptoms of GVHD is an important part of care after allogeneic HSCT. | Graft-versus-host disease (GVHD) is a significant complication of allogeneic hematopoietic stem cell transplantation (HSCT), where the donated stem cells view the recipient's body as foreign and start to attack it. GVHD can affect various systems in the body, including the skin, gastrointestinal tract, and liver, leading to a variety of symptoms.  GVHD prophylaxis refers to the strategies and medications used to prevent the occurrence of GVHD after HSCT. The prophylaxis regimen usually involves the use of immunosuppressive medications to decrease the immune response of the donated stem cells against the recipient's body.  Commonly used medications in GVHD prophylaxis include:  1. **Calcineurin inhibitors**: Such as cyclosporine or tacrolimus. These drugs inhibit the activation of T cells, a type of white blood cell that plays a key role in GVHD.  2. **Antimetabolites**: Such as methotrexate or mycophenolate mofetil. These drugs inhibit cell proliferation, including that of immune cells.  3. **Antithymocyte globulin (ATG)**: This is a type of immunosuppressive therapy that depletes T cells and is often used in the conditioning regimen before HSCT, particularly in patients with a high risk of GVHD.  4. **Corticosteroids**: Such as prednisone. These drugs have a broad immunosuppressive effect and may be used in GVHD prophylaxis, although they are more often used for the treatment of GVHD if it occurs.  5. **Post-transplant cyclophosphamide (PTCy)**: This is a newer strategy for GVHD prophylaxis that involves giving high-dose cyclophosphamide in the first few days after HSCT. This approach is particularly used in haploidentical HSCT (transplants from half-matched donors).  The specific GVHD prophylaxis regimen can vary depending on various factors, including the type of transplant, the degree of matching between donor and recipient, the recipient's health status, and the center's protocol. Despite these prophylactic measures, GVHD can still occur, and early detection and management are crucial. | GVHD, or graft-versus-host disease, is a potential complication of allogeneic hematopoietic stem cell transplantation (HSCT). This condition occurs when the donated stem cells (the graft) view the recipient's body (the host) as foreign and start to attack the host's tissues. GVHD can affect many parts of the body, including the skin, liver, and gastrointestinal tract, and it can be either acute (occurring within the first 100 days after transplantation) or chronic (occurring after 100 days).  GVHD prophylaxis refers to the strategies and medications used to prevent the development of GVHD after HSCT. This typically involves the use of immunosuppressive medications to dampen the immune response of the transplanted cells. Commonly used medications for GVHD prophylaxis include:  1. **Calcineurin inhibitors**: This class of drugs, including cyclosporine and tacrolimus, inhibit the activity of T-cells, which play a key role in the immune response that leads to GVHD.  2. **Antimetabolites**: Methotrexate and mycophenolate mofetil are drugs that inhibit the proliferation of immune cells, thus reducing the likelihood of GVHD.  3. **Antithymocyte globulin (ATG)**: This is a type of immunosuppressive therapy that reduces the number of T-cells in the body, which can help prevent GVHD.  The specific GVHD prophylaxis regimen can vary based on many factors, including the type of transplant, the specific risk factors of the patient, the condition being treated, and the preferences of the medical team. Despite these preventative measures, GVHD can still occur in some cases, and it's important for patients to be closely monitored after transplantation for signs and symptoms of this condition. |
| P2 | Treosulfan is a bifunctional alkylating agent that is used in the conditioning regimen for patients undergoing hematopoietic stem cell transplantation (HSCT). A conditioning regimen is a treatment given before HSCT to help prepare the body to receive the stem cells and to increase the chances that the transplant will be successful.  Treosulfan acts by inducing breaks in the DNA strand, which can interfere with DNA replication and transcription. This can cause cell death and also suppress the immune system, reducing the chances of graft rejection after HSCT.  One of the benefits of treosulfan compared to some other conditioning agents is that it has a lower toxicity profile, particularly in terms of organ toxicity. This makes it a particularly valuable option for patients who may not be able to tolerate more intense conditioning regimens. It's often used in combination with other drugs, such as fludarabine, in a reduced-intensity conditioning regimen.  Like all chemotherapy agents, treosulfan can have side effects, and it's important for patients to be monitored closely while receiving this drug. Potential side effects can include nausea, vomiting, fatigue, decreased blood counts, and increased susceptibility to infections, among others. | Treosulfan is a bifunctional alkylating agent that is used primarily in the conditioning regimen before hematopoietic stem cell transplantation (HSCT). It has demonstrated efficacy in a variety of malignant and non-malignant conditions, including acute myeloid leukemia (AML), myelodysplastic syndrome (MDS), and various genetic and immunodeficiency syndromes.  The conditioning regimen is a treatment given before HSCT to prepare the patient's body for the transplant. The goal of this regimen is to eradicate malignant cells in the body (in the case of malignant conditions) and to suppress the immune system to prevent it from attacking the transplanted cells (graft rejection).  Treosulfan is often used as a less toxic alternative to traditional conditioning agents such as busulfan, particularly in patients who are older or have comorbidities that make them less able to tolerate intense conditioning regimens. When administered in high doses, treosulfan has been shown to effectively suppress the immune system and to have a potent anti-leukemic effect.  As of my knowledge cutoff in September 2021, please verify from a reliable source for the most recent updates and approvals. | Treosulfan is a medication used in cancer treatment. As of my last update in September 2021, it is primarily used in preparation for a stem cell transplant in patients with certain types of blood or bone marrow cancer.  Treosulfan is a type of alkylating agent, a category of chemotherapy drugs that work by damaging the DNA of cancer cells, preventing them from dividing and growing. This medication is generally administered intravenously, often in combination with other chemotherapy drugs.  The specific cancers that treosulfan is used to treat, its effectiveness, and its side effects can vary, and ongoing research may have led to additional uses or a better understanding of its properties. Please consult with a healthcare professional or search for more recent information for the most accurate and up-to-date details. |
| P3 | As of my last update in September 2021, the standard conditioning regimen for patients with multiple myeloma undergoing autologous hematopoietic stem cell transplantation (HSCT) often includes high-dose melphalan.  Melphalan is an alkylating agent like treosulfan and is typically used because of its efficacy and tolerability in this patient population. The standard dose is typically 200 mg/m^2, given intravenously. This high-dose treatment is generally administered over one or two days.  This conditioning regimen is designed to suppress the patient's immune system and kill off the remaining myeloma cells before the transplant. After the conditioning regimen, the patient's previously harvested stem cells are reinfused back into the patient to help their bone marrow recover and produce new, healthy blood cells.  Please note that the specific regimen and dose may vary depending on factors such as the patient's age, overall health, disease stage, and response to previous treatments. Moreover, advancements in treatment strategies may occur, and guidelines can change. Therefore, it is essential to consult with a healthcare professional for the most accurate and current information. | As of my last update in September 2021, the standard conditioning regimen for multiple myeloma patients undergoing autologous hematopoietic stem cell transplantation (HSCT) typically involves high-dose melphalan.  Melphalan is an alkylating agent that works by preventing cancer cells from growing and dividing. The dosage of melphalan can vary based on a variety of factors, including the patient's overall health status, kidney function, and body surface area.  A commonly used dose of melphalan in this context is around 200 mg/m^2, typically administered intravenously. However, the exact dose and administration schedule can vary, and it's important that these decisions are made by a healthcare professional who can assess the individual patient's needs and circumstances.  There may be some cases where other conditioning regimens are used, especially in patients who may not be able to tolerate high-dose melphalan. These decisions should always be made in consultation with a healthcare provider who specializes in treating multiple myeloma and performing stem cell transplants.  Please consult with a healthcare professional or search for more recent information for the most accurate and up-to-date details. | As of my last update in September 2021, high-dose melphalan is the standard conditioning regimen for multiple myeloma patients undergoing autologous hematopoietic stem cell transplantation (HSCT). Melphalan is another alkylating agent used in chemotherapy.  The usual dose for conditioning in this context is Melphalan 200 mg/m² administered intravenously. This is typically given as a single dose, one to two days before the stem cell transplant.  However, the exact dosage may vary depending on a variety of factors, such as the patient's overall health, kidney function, and the specifics of their disease. Adjustments might be needed for those with renal insufficiency or other comorbidities. It is essential that any chemotherapy regimen be administered under the supervision of a healthcare professional who can monitor for side effects and adjust treatment as needed.  Please note that newer conditioning regimens and drugs may have been developed or accepted in the medical community since my last training data in September 2021, so it's always important to consult with the treating oncologist or hematologist for the most accurate and current information. |
| P4 | Post-transplant cyclophosphamide is a medication given after hematopoietic stem cell transplantation (HSCT) to help prevent graft-versus-host disease (GVHD), a condition where donor cells attack the recipient's body.  Typically, post-transplant cyclophosphamide is given on days +3 and +4 after the HSCT, where day 0 is the day of the transplant.  Please note that this is a typical schedule, but the exact timing and dosage can vary depending on specific patient circumstances, the type of transplant, and the protocol used by the transplantation center.  It is also important to note that as of my last training data in September 2021, new approaches to GVHD prophylaxis might have emerged, so please consult with a healthcare professional for the most accurate and up-to-date information. | Post-transplant cyclophosphamide (PTCy) is a common approach to prophylaxis against graft-versus-host disease (GVHD) in patients undergoing allogeneic hematopoietic stem cell transplantation (HSCT).  As of my last update in 2021, the typical timing for PTCy administration is on days +3 and +4 post-transplant, where "day 0" is the day of the stem cell infusion. The doses are usually around 50 mg/kg/day, given intravenously. This timing helps eliminate alloreactive T cells that can cause GVHD, while sparing stem cells and promoting immune tolerance.  Keep in mind that protocols can vary by specific patient populations and individual transplant centers, so the exact timing and dosage may differ. Always refer to the specific recommendations of the treating physician or clinical guidelines in place at the time of treatment.  Furthermore, newer approaches or research may have changed these guidelines since my last training data in September 2021, so please consult a healthcare professional for the most recent and accurate information. | Post-transplant cyclophosphamide is a strategy used in hematopoietic stem cell transplantation (HSCT) to prevent graft-versus-host disease (GVHD), a complication where the transplanted cells attack the host's body.  As of my knowledge cutoff in September 2021, when post-transplant cyclophosphamide is used as GVHD prophylaxis, it is typically given on Days +3 and +4 following HSCT (where Day 0 is the day of the transplant). The dose commonly used is 50 mg/kg/day.  The exact timing and dosage may vary based on patient characteristics, transplant type, the specifics of the disease being treated, and the center's practice. Also, additional immunosuppressive drugs may be given along with post-transplant cyclophosphamide for further GVHD prophylaxis.  Please note that clinical practices may evolve, and more recent data or guidelines might suggest different protocols. Always consult with the treating physician or healthcare provider for the most accurate and current information. |
| P5 | Human Leukocyte Antigen (HLA) typing is a critical process in matching organ and bone marrow donors with recipients. HLA are proteins that help the immune system distinguish the body's own cells from foreign substances.  In healthy donors, HLA typing is typically performed using peripheral blood samples. Sometimes, buccal swabs (from the inside of the cheek) or saliva samples may be used, particularly for preliminary screening.  The blood or buccal cells collected are then sent to a laboratory where specialized tests, such as sequence-specific primer PCR, sequence-based typing, or next-generation sequencing, are used to identify the specific HLA alleles present.  However, it's important to note that testing methods and accepted sample types may vary across different labs and regions and may evolve over time with scientific and technological advances. It is always recommended to follow the specific guidelines of the donation center or medical institution involved. | Human leukocyte antigen (HLA) typing is used to match patients and donors for bone marrow or cord blood transplants. HLA are proteins — or markers — found on most cells in your body, and the immune system uses these markers to recognize which cells belong in your body and which do not.  For healthy donors, HLA typing is typically performed using peripheral blood samples. However, other types of samples can also be used for this purpose, including buccal swabs or saliva, as these samples also contain cells from which DNA can be extracted for the HLA typing process. The choice of sample may depend on factors like the convenience for the donor, the procedures of the particular clinic or HLA laboratory, or the specifics of the test being used.  It's important to note that HLA typing is a complex process and must be performed in a specialized laboratory. It involves DNA-based techniques to ensure the highest possible level of matching between donor and recipient, which is crucial for the success of a transplant and for minimizing the risk of complications like graft-versus-host disease (GVHD). | Human leukocyte antigen (HLA) typing is a testing process performed to match patients and donors for bone marrow or cord blood transplants, which is crucial to reduce the risk of graft-versus-host disease (GVHD).  Typically, HLA typing in healthy donors can be performed on various sample types, including:  1. **Blood samples:** Peripheral blood is commonly used for HLA typing. The blood sample can be a full blood sample or it can be specifically white blood cells (leukocytes), which are the cells that carry the HLA antigens.  2. **Buccal swabs:** Cells are collected from the inside of a person's cheek using a special swab. This method is non-invasive and often used for initial screening in bone marrow donor registries.  3. **Saliva samples:** Similar to buccal swabs, saliva samples can be used for non-invasive preliminary screening.  It's important to note that while non-invasive methods like buccal swabs and saliva samples can be used for initial HLA typing, confirmatory testing often requires a blood sample to ensure the highest degree of accuracy. The exact procedure may also depend on the specific policies and protocols of the transplant center or registry.  As always, practices may vary and evolve over time, so it's advisable to consult with a medical professional or the specific transplant center or registry for the most up-to-date and accurate information. |
| P6 | High-dose cytarabine, an antineoplastic agent, is known to potentially cause a unique ocular side effect known as cytarabine-induced keratoconjunctivitis, which can present with symptoms like burning, gritty sensation, blurred vision, photophobia, and even severe ocular pain.  To prevent this, the use of corticosteroid eye drops, such as dexamethasone, is typically recommended during treatment with high-dose cytarabine. These eye drops can help to reduce inflammation and discomfort associated with keratoconjunctivitis.  Typically, the eye drops are started shortly before the initiation of high-dose cytarabine therapy and continued for a few days after completion of therapy. The typical frequency is every 4 to 6 hours while awake.  However, this is a general guideline and the specifics can depend on various factors. The exact regimen (which type of eye drop, how often to use, when to start and stop) should be determined by the treating physician based on the individual patient's situation.  Please consult a healthcare provider for professional advice. | High-dose cytarabine, a chemotherapy drug used to treat certain types of leukemia, has been associated with a specific side effect called cytarabine syndrome, which includes ocular toxicity such as conjunctivitis and keratitis. This can cause symptoms such as eye pain, blurred vision, and watery or dry eyes.  To prevent or minimize these side effects, corticosteroid eye drops (like dexamethasone) are often recommended. These drops can help to reduce inflammation in the eye, providing relief from these symptoms.  The typical regimen for corticosteroid eye drops during high-dose cytarabine therapy involves administering the drops every 4-6 hours while awake, starting at the time of chemotherapy initiation and continuing for at least 48 hours after the last dose of cytarabine. The dosing may be adjusted based on individual patient needs and response.  As with all medical decisions, the patient should discuss this treatment and any potential side effects with their healthcare provider. The healthcare provider will consider the patient's overall health status, other medications they may be taking, and other potential side effects of the chemotherapy in making a recommendation. | High-dose cytarabine (also known as cytosine arabinoside or Ara-C) is a chemotherapy drug used in the treatment of various types of cancer, including certain types of leukemia. One of the side effects of high-dose cytarabine can be a toxic effect on the eyes, known as cytarabine-induced keratoconjunctivitis. This can cause symptoms such as redness, pain, blurred vision, and excessive tearing.  To prevent this, patients receiving high-dose cytarabine are often given prophylactic corticosteroid eye drops. A common choice is prednisolone acetate 1% eye drops. These drops can help reduce inflammation and prevent the development of keratoconjunctivitis.  The typical dosage regimen is 2 drops instilled into the conjunctival sac of each eye every 4 to 6 hours, starting 6 hours before the first dose of cytarabine and continuing until 48 hours after the last dose of cytarabine.  Remember, the above information is a general guideline, and the actual medication and dosing may vary based on the specific patient and healthcare provider recommendations. Always consult a healthcare provider for the appropriate treatment. This information should not be used as a substitute for professional medical advice. |
| P7 | The eligibility to become a volunteer hematopoietic cell donor often depends on a number of factors, including the type of cancer, its treatment, and whether it's in remission.  As of my last training data in September 2021, a person with a history of most types of cancer, including prostate cancer, generally may not be eligible to donate stem cells through Be The Match or most other donor registries. The primary concern is the health of the potential donor, as well as the possibility of transferring cancer cells to the recipient. However, exceptions may exist for certain types of skin cancers and other cancers that have been curable and in remission for a considerable period of time.  Given this individual's history of prostate cancer that required systemic chemotherapy, he would likely not be eligible to register. It's important to note that eligibility guidelines can be complex and may change over time, so it's always recommended to consult with a healthcare professional or the organization running the registry for the most accurate information. Be The Match, for instance, provides detailed eligibility guidelines on their website and also has contact information for questions about specific situations. | The Be The Match Registry, run by the National Marrow Donor Program (NMDP), is a listing of potential hematopoietic cell donors in the United States. They have guidelines regarding who can join the registry as a potential donor, and these guidelines include considerations about prior history of cancer.  As of my last update in September 2021, the guidelines generally state that individuals with a history of cancer are often not able to donate. This is because the treatments for cancer, like chemotherapy and radiation, can damage the bone marrow's ability to produce healthy cells. However, the decision can sometimes depend on the type of cancer, when it occurred, and the specifics of the treatment.  In the case of prostate cancer that required systemic chemotherapy, it's not clear without additional specifics whether this person would be eligible to register. A comprehensive medical evaluation and discussion with the Be The Match Registry or another appropriate medical authority would be needed to make a final determination.  Please note that the policies and guidelines of registries like Be The Match may have changed since my last update in September 2021, so it's important to check the most current information.  I would recommend that this individual contact Be The Match or another appropriate authority directly to discuss their specific situation and get the most accurate and up-to-date advice. | As of my knowledge cutoff in September 2021, individuals with a history of cancer, including prostate cancer, generally are not eligible to donate bone marrow or peripheral blood stem cells through the Be The Match Registry. This policy is in place to protect both the potential donor and the recipient.  For the potential donor, donating stem cells can be physically demanding and may pose health risks, especially for individuals who have had serious illnesses such as cancer. Even if the individual's cancer is in remission, the donation process could still potentially impact their health.  For the recipient, cells from a donor who has had cancer could potentially carry a risk of transmitting cancerous cells, although this risk may be very small for some types of cancer.  However, final eligibility is often determined on a case-by-case basis and can depend on various factors, including the type of cancer, how long ago it was treated, and the current health of the potential donor. It's important to consult with the organization's medical team or a healthcare provider for the most accurate information.  Please note that guidelines can change over time and may vary between different registries and countries. Therefore, it's advisable to directly contact Be The Match or the relevant bone marrow donor registry for the most current and applicable guidelines. |
| P8 | Patients with multiple myeloma who have achieved complete remission after an autologous stem cell transplant (ASCT) often benefit from maintenance therapy. This is a form of continuous treatment intended to prolong the duration of remission and improve survival.  As of my knowledge cut-off in September 2021, the most commonly used drug for maintenance therapy in multiple myeloma is lenalidomide. This is an oral drug that is typically taken daily. Lenalidomide can help keep the disease under control by slowing or stopping the growth of cancer cells.  In terms of duration, lenalidomide maintenance therapy is typically continued until disease progression or unacceptable toxicity. The treatment's indefinite nature is based on clinical trials demonstrating a survival advantage with prolonged lenalidomide therapy.  However, this comes with the caveat that maintenance therapy can be associated with side effects such as neutropenia (low white blood cell count), thrombocytopenia (low platelet count), an increased risk of secondary cancers, and a risk of deep vein thrombosis or pulmonary embolism. As such, the decision to continue maintenance therapy should be personalized, considering the individual patient's disease characteristics, side effect profile, comorbid conditions, and personal preferences.  Please note that treatment strategies can evolve over time, and there might be new developments beyond my knowledge cut-off in September 2021. It's important for patients to discuss their specific situation and treatment plan with their healthcare team to make the most informed decision. | As of my last update in September 2021, the consensus for multiple myeloma treatment in patients who have achieved complete remission after autologous stem cell transplantation (ASCT) is to consider maintenance therapy to prolong remission and improve overall survival.  Maintenance therapy typically includes immunomodulatory drugs (IMiDs), such as lenalidomide, which is often the preferred agent due to its efficacy and manageable side effect profile. Another agent, bortezomib, may be considered, particularly in patients with high-risk cytogenetics or those who are intolerant or resistant to lenalidomide.  As for the duration of maintenance therapy, this can vary. Traditionally, lenalidomide has been given until disease progression or unacceptable toxicity. However, this can be many years, and long-term use of lenalidomide may come with side effects and risks such as secondary malignancies. Therefore, the optimal duration of maintenance therapy is still a matter of clinical debate and research.  It's crucial to keep in mind that the best treatment approach for any individual patient depends on various factors, including their overall health, kidney function, cytogenetic risk, response to previous treatments, and personal preferences. It is always recommended to discuss these issues in depth with the treating oncologist or hematologist to reach the best individual treatment plan.  Please note that medical knowledge and treatment guidelines evolve over time, and newer treatments and strategies may have been developed or become standard of care since my last training data in 2021. Always consult with the treating physician or healthcare provider for the most accurate and current information. | As of my last update in September 2021, the recommended approach for a patient with multiple myeloma who is in complete remission after a second autologous stem cell transplant would typically involve maintenance therapy. Maintenance therapy is used to help prolong the duration of remission and potentially improve overall survival.  The most commonly used agent for maintenance therapy is lenalidomide. Lenalidomide is an immunomodulatory drug that has been shown in clinical trials to improve progression-free survival when used as maintenance therapy after autologous stem cell transplantation for multiple myeloma.  The duration of maintenance therapy can vary. Some studies and guidelines suggest that it be continued indefinitely, as long as the patient is responding to treatment and not experiencing unacceptable side effects. However, other experts may recommend a fixed duration of maintenance therapy, such as 1-2 years, or may individualize the duration based on factors like the patient's disease risk, age, and tolerance of the medication.  Regular follow-up appointments would also be needed to monitor the patient's condition, watch for any signs of relapse, and manage any side effects of treatment. The frequency of these appointments can vary, but they might be every few months initially and then less often if the patient remains stable.  Please note that recommendations can vary and newer treatment strategies may have been developed or accepted in the medical community since my last training data in September 2021, so it's important to consult with the treating physician for the most accurate and current information. |
| P9 | Yes, as of my last update in September 2021, autologous hematopoietic stem cell transplantation (HSCT) can be considered for certain patients with multiple sclerosis (MS), particularly those with aggressive, highly active, or refractory disease not well controlled by standard disease-modifying therapies.  Multiple sclerosis is an autoimmune disease in which the body's immune systemattacks the myelin sheaths of neurons in the central nervous system. Autologous HSCT involves the use of high-dose chemotherapy to ablate or "reset" the patient's immune system, followed by the reinfusion of the patient's own stem cells to rebuild the immune system. The goal is to halt the autoimmune attack on the central nervous system.  While HSCT can be a potent treatment for MS, it's important to note that it carries significant risks, including the risk of serious infections, organ damage, infertility, secondary cancers, and even death. Therefore, it's typically reserved for patients with rapidly worsening disease who have not responded to other treatments and who are otherwise in good health.  In addition, more research is needed to understand who is most likely to benefit from this treatment, how it compares to other treatments for MS, and how to minimize its risks.  As medical knowledge and practice evolve over time, the use of autologous HSCT in the treatment of multiple sclerosis may become more or less common, and the patient selection criteria may change. Therefore, it's always important to consult with a healthcare provider for the most current and accurate information. | As of my knowledge cutoff in September 2021, autologous hematopoietic stem cell transplantation (HSCT) is indeed considered a treatment option for certain patients with multiple sclerosis (MS), particularly for those with aggressive disease that's not responding to standard therapies. The goal of autologous HSCT in this setting is to "reset" the immune system and halt the progression of the disease.  The procedure involves collecting the patient's own stem cells, followed by administration of high-dose chemotherapy (the conditioning regimen) to ablate the patient's immune system, and then reinfusion of the collected stem cells to repopulate the immune system.  While it can be effective, autologous HSCT is generally considered a high-risk procedure and is typically reserved for patients with rapidly progressing disease who have not responded to other treatments. The risks of this procedure include infection, organ damage, infertility, and other short- and long-term complications. It's also important to note that while HSCT can halt the progression of MS, it might not reverse existing disability or symptoms.  Given the risks and complexities of this treatment, it's important that the decision to proceed with HSCT is made on an individual basis, taking into account the patient's overall health, disease severity and progression, response to other treatments, and personal preferences.  Please note that treatment guidelines and practices can evolve, and more recent data or guidelines might suggest different strategies for treating MS. Therefore, always consult with the treating neurologist or a hematologist for the most accurate and current information. | Yes, as of my knowledge cutoff in September 2021, autologous hematopoietic stem cell transplantation (HSCT) can be considered as a treatment option for certain patients with multiple sclerosis (MS), particularly for those with highly active relapsing-remitting MS that is not well controlled with standard therapies.  MS is an autoimmune disease where the body's immune system attacks its own nerve cells, leading to neurologic symptoms. Autologous HSCT involves using high-dose chemotherapy to 'reset' the immune system, thereby reducing the autoimmunity that is causing the disease. The patient's own (autologous) stem cells are collected before the chemotherapy and then reinfused after the chemotherapy to help the bone marrow recover.  While autologous HSCT can be effective for controlling MS, it is generally considered a last resort due to its risks, which can include infections and other serious complications from the high-dose chemotherapy. Therefore, it is typically only considered for patients with aggressive disease that is not responding to other treatments, and who are otherwise in good health.  This is an area of active research, and more recent studies or guidelines may provide further information about the role of autologous HSCT in treating MS. Always consult with a healthcare provider for the most accurate and current information. |
| P10 | Yes, the donor can still be suitable even with a major ABO mismatch. ABO incompatibility is not considered a contraindication for hematopoietic stem cell transplantation (HSCT). This is because the ABO blood group system is not a part of the human leukocyte antigen (HLA) system, which is the key factor in donor matching for HSCT.  However, ABO incompatibility can lead to certain complications during and after the transplant. These can include immediate hemolytic reactions, delayed red blood cell engraftment, and pure red cell aplasia. As a result, additional precautions and procedures may be necessary to manage these risks.  These can include:  1. **Red blood cell depletion:** For major ABO incompatibility, the stem cell graft may be processed to remove red blood cells, reducing the risk of a hemolytic reaction.  2. **Plasma reduction:** This process removes plasma from the graft to minimize the transfer of ABO antibodies from the donor to the recipient.  3. **Immunoglobulin prophylaxis:** This may be given to the recipient to help prevent hemolysis.  4. **Regular monitoring post-transplant:** This will ensure any ABO-related complications are detected and managed promptly.  As for other tests, the primary ones are HLA typing of both the donor and the recipient to ensure the best possible match. This helps to minimize the risk of graft-versus-host disease (GVHD) and improve the chances of a successful transplant. Other important pre-transplant tests include infectious disease screening, complete blood counts, and organ function tests, among others.  Please note that these recommendations are based on knowledge as of September 2021, and newer practices or guidelines may exist. It is always important to consult with the healthcare team managing the transplant for the most accurate and current information. | Yes, a donor can still be suitable for a hematopoietic stem cell transplant (HSCT) even with a major ABO mismatch. ABO incompatibility is not as critical in HSCT as it is in solid organ transplantation because mature red blood cells are not a significant component of the graft.  However, ABO incompatibility can complicate the transplant process and require additional measures. For example, a major ABO mismatch (where the recipient has antibodies against the donor's blood type) could potentially lead to immediate or delayed hemolysis (destruction of red blood cells) after the transplant. In such cases, strategies like plasma reduction of the graft, recipient plasma exchange, or immunoadsorption can be used to reduce the risk of hemolysis.  As for additional tests, it's important to match the recipient and donor for the human leukocyte antigen (HLA) system, which is much more critical for successful HSCT. HLA matching helps to reduce the risk of graft-versus-host disease (GVHD) and graft rejection. Ideally, a matched sibling donor is used, but if such a donor is not available, an unrelated donor can be sought through a donor registry.  Other tests for the donor may include infectious disease screening (for diseases like HIV, hepatitis B and C, CMV, etc.), complete blood count, kidney and liver function tests, and possibly others depending on the specific policies and protocols of the transplant center.  As always, consult with the treating physician or a transplant specialist for the most accurate and current information. They can provide detailed advice based on the specifics of the patient's situation and the most recent evidence and guidelines. | Yes, a donor can still be suitable even if there is a major ABO blood group mismatch between the donor and the recipient in peripheral blood stem cell transplantation. The ABO blood group system is important for blood transfusions, but it is not the primary consideration for stem cell transplants. The critical factor in stem cell or bone marrow transplantation is matching the human leukocyte antigen (HLA) types of the donor and recipient, which helps to minimize the risk of graft-versus-host disease (GVHD).  However, ABO incompatibility can introduce some complications, such as hemolysis (destruction of red blood cells) or delayed red blood cell recovery, which need to be managed by the medical team. For example, some centers might remove red cells or plasma from the graft before the transplant (a process called 'graft manipulation' or 'depletion') to reduce the risks associated with ABO incompatibility.  Apart from HLA typing and ABO blood typing, potential donors also undergo other screenings and tests. These can include:  1. **Infectious disease screening:** This is done to ensure that the donor does not carry any infectious diseases that could be transmitted to the recipient during the transplant.  2. **Medical evaluation:** A thorough medical evaluation is conducted to ensure that the donor is healthy enough to donate stem cells and that the donation would not pose undue risk to their own health.  3. **Psychosocial evaluation:** Some programs also conduct a psychosocial evaluation to ensure that the donor fully understands the donation process and is prepared for it.  Please consult with a healthcare professional or transplant center for the most accurate and current information. Remember, clinical practices can evolve over time, so newer protocols may have been developed since my last training data in September 2021. |
| P11 | The Disease Risk Index (DRI) is a tool used to predict overall survival and disease-free survival in patients undergoing hematopoietic stem cell transplantation (HSCT). The DRI is divided into four categories: Very Low, Low, Intermediate, and High, based on the type and stage of the disease.  Your question refers to a 32-year-old patient with acute myeloid leukemia (AML) with intermediate-risk cytogenetics in first complete remission. According to the original DRI model published by Armand et al., this patient would generally be classified as 'Low' risk.  However, it's important to remember that the DRI is not the only determinant of outcomes after HSCT. Patient age, performance status, comorbidities, donor match, and other factors can significantly affect overall survival and should be evaluated in conjunction with the DRI.  Regarding the overall survival rate at 2 years, it's difficult to provide a specific percentage without more detailed patient information. Survival rates can vary widely based on a multitude of factors including but not limited to the specific subtype of AML, the patient's overall health status, any subsequent treatments, and the specifics of the HSCT procedure such as graft-versus-host disease (GVHD) prophylaxis. Generally speaking, survival rates for patients with AML who undergo HSCT can range anywhere from 20-70%. A hematologist-oncologist or transplant specialist would be able to provide a more accurate estimation based on the specific details of the patient's condition and treatment plan.  I would strongly advise you to refer to the most recent clinical guidelines and research literature, and consult with a healthcare professional, to get the most accurate and up-to-date information. | The Disease Risk Index (DRI) is a tool used to stratify patients undergoing hematopoietic stem cell transplantation (HSCT) into different risk groups. It's based on disease type and stage, as well as cytogenetic risk. It divides patients into 4 categories: low, intermediate, high, and very high risk.  Acute Myeloid Leukemia (AML) in first complete remission with intermediate-risk cytogenetics is classified as Intermediate DRI according to the original classification developed by Armand et al. published in Blood in 2012.  However, the patient's specific DRI score or the exact prognosis at 2 years can't be determined without more specific patient data, such as the specific cytogenetic and molecular abnormalities, the response to prior therapy, and the patient's overall health. Survival rates also depend on factors like the donor match quality, the transplant center's expertise, and potential post-transplant complications such as graft-versus-host disease or infections.  Therefore, it would be most appropriate for the patient's treating physician or hematologist to determine this information based on the full scope of the patient's medical history and current health status. As an AI, I can provide general information based on the data available up until my last update in September 2021, but it's important to consult with a medical professional for personalized medical advice. | The Disease Risk Index (DRI) is a tool used to evaluate the relative risk of mortality after hematopoietic stem cell transplantation (HSCT) based on the patient's disease and stage. This scoring system was developed by the Center for International Blood and Marrow Transplant Research (CIBMTR).  As of my knowledge cutoff in September 2021, the DRI scores are classified into four categories: low, intermediate, high, and very high. Acute Myeloid Leukemia (AML) in the first complete remission (CR1) with intermediate cytogenetics generally falls into the "Intermediate" DRI category.  Regarding the 2-year overall survival rate, it varies widely based on a multitude of factors including, but not limited to, the patient's general health, conditioning regimen, the specific genetic abnormalities present in the leukemia cells, donor match quality, graft versus host disease (GVHD) prophylaxis, and the occurrence of complications. As such, providing a precise survival rate would require detailed, up-to-date medical information and is generally the domain of the treating physician.  To get the most accurate prediction of the 2-year survival rate, I recommend consulting with the patient's oncologist or hematologist, as they would have access to the most recent clinical data and guidelines. The above information should be used for educational purposes only and not as a substitute for professional medical advice. |
| P12 | DSA, or donor-specific antibodies, refer to antibodies that a recipient has that are specifically directed against antigens present in the donated organ or cells. In the context of hematopoietic stem cell transplantation (HSCT), the presence of DSA can complicate the transplant process.  The major risk associated with DSA in HSCT is graft rejection, particularly a type called antibody-mediated rejection (AMR). AMR occurs when these preformed antibodies bind to antigens on the donor cells, leading to their destruction by the recipient's immune system.  DSA can also contribute to other post-transplant complications. For example, they have been associated with graft-versus-host disease (GVHD), a serious condition where donor immune cells attack the recipient's body.  Another potential risk is the development of transplant-associated microangiopathy (TAM), a rare complication of HSCT that affects small blood vessels in the body.  Given these risks, patients with DSA may require special management before and after transplantation, such as desensitization protocols to reduce the levels of these antibodies, and careful immunosuppressive strategies after transplantation. The specific approach depends on various factors, including the specific antibodies involved, their levels, and the patient's overall health status.  Always consult with a healthcare provider for personalized advice. This information should not be used as a substitute for professional medical advice. | DSA stands for Donor-Specific Antibodies. These are antibodies produced by the recipient's immune system that specifically target antigens present in the donated organ or stem cells. In the context of hematopoietic stem cell transplantation (HSCT), the presence of DSAs can significantly affect the outcomes.  If a patient undergoing HSCT has DSAs, they are at an increased risk of developing graft-versus-host disease (GVHD), where the transplanted stem cells view the recipient's body as foreign and attack it. They are also at a higher risk for graft failure, where the transplanted cells do not engraft or grow in the recipient's body. This could potentially lead to severe complications, including life-threatening infections, anemia, and bleeding.  The presence of DSAs may also result in delayed engraftment, where the transplanted stem cells take longer to start growing and developing into new cells.  Moreover, DSAs can also increase the risk for transplant-related mortality. Therefore, detecting and managing DSAs is a crucial part of the pre-transplant workup and post-transplant care.  This information should not be used as a substitute for professional medical advice. Always consult with a healthcare provider. | DSA stands for Donor Specific Antibodies. These are antibodies in the recipient's blood that specifically target the donated organ or, in this case, hematopoietic stem cells. If a patient undergoing hematopoietic stem cell transplantation (HSCT) has DSAs, it could potentially increase the risk of various complications.  The primary concern would be graft rejection. This can occur when the recipient's immune system recognizes the donated stem cells as foreign and attacks them. Graft rejection can be life-threatening and often requires intensive treatment to manage.  In addition, DSAs can also contribute to another severe condition called graft-versus-host disease (GVHD). While GVHD is generally caused by donor T cells recognizing the recipient as foreign, DSAs could potentially exacerbate the condition by further stimulating the immune response.  This is why patients are typically screened for DSAs before undergoing HSCT, to help ensure the best possible match and reduce the risk of these complications. If DSAs are identified, additional therapies may be required to reduce the levels of these antibodies before transplantation can proceed. Always consult a healthcare provider for the appropriate treatment and management strategies. This information should not be used as a substitute for professional medical advice. |
| P13 | As of my last training data in September 2021, the first-line treatment for a young patient diagnosed with severe aplastic anemia (SAA) who has a suitable human leukocyte antigen (HLA)-matched sibling donor is allogeneic hematopoietic stem cell transplantation (HSCT). This procedure involves replacing the patient's blood-forming stem cells with those of the healthy donor.  Allogeneic HSCT can potentially cure severe aplastic anemia. Studies have shown that younger patients and those who receive transplants from matched sibling donors generally have the best outcomes.  If a suitable HLA-matched sibling donor is not available, alternative options may include an allogeneic HSCT from a matched unrelated donor or a half-matched (haploidentical) donor, or immunosuppressive therapy (IST) with agents such as antithymocyte globulin (ATG) and cyclosporine. The choice between these options would depend on several factors, including the availability and quality of an unrelated or half-matched donor, the urgency of treatment, the patient's overall health, and the specific practices of the treating center.  Please consult with a healthcare provider for the most accurate and current information. Remember, treatment strategies for severe aplastic anemia can vary and may have evolved since my last training data in September 2021. | As of my last training data in September 2021, the first-line treatment for a young patient diagnosed with severe aplastic anemia (SAA) who has a suitable human leukocyte antigen (HLA)-matched sibling donor is allogeneic hematopoietic stem cell transplantation (HSCT). This procedure involves replacing the patient's blood-forming stem cells with those of the healthy donor.  Allogeneic HSCT can potentially cure severe aplastic anemia. Studies have shown that younger patients and those who receive transplants from matched sibling donors generally have the best outcomes.  If a suitable HLA-matched sibling donor is not available, alternative options may include an allogeneic HSCT from a matched unrelated donor or a half-matched (haploidentical) donor, or immunosuppressive therapy (IST) with agents such as antithymocyte globulin (ATG) and cyclosporine. The choice between these options would depend on several factors, including the availability and quality of an unrelated or half-matched donor, the urgency of treatment, the patient's overall health, and the specific practices of the treating center.  Please consult with a healthcare provider for the most accurate and current information. Remember, treatment strategies for severe aplastic anemia can vary and may have evolved since my last training data in September 2021. | As of my last training data in September 2021, the first-line treatment for a young patient diagnosed with severe aplastic anemia (SAA) who has a suitable human leukocyte antigen (HLA)-matched sibling donor is allogeneic hematopoietic stem cell transplantation (HSCT). This procedure involves replacing the patient's blood-forming stem cells with those of the healthy donor.  Allogeneic HSCT can potentially cure severe aplastic anemia. Studies have shown that younger patients and those who receive transplants from matched sibling donors generally have the best outcomes.  If a suitable HLA-matched sibling donor is not available, alternative options may include an allogeneic HSCT from a matched unrelated donor or a half-matched (haploidentical) donor, or immunosuppressive therapy (IST) with agents such as antithymocyte globulin (ATG) and cyclosporine. The choice between these options would depend on several factors, including the availability and quality of an unrelated or half-matched donor, the urgency of treatment, the patient's overall health, and the specific practices of the treating center.  Please consult with a healthcare provider for the most accurate and current information. Remember, treatment strategies for severe aplastic anemia can vary and may have evolved since my last training data in September 2021. |
| P14 | For a patient with NPM1-mutated acute myeloid leukemia (AML) and a normal karyotype, achieving complete molecular remission after induction is a good sign. However, the return of minimal residual disease (MRD) after the second consolidation cycle indicates that some leukemia cells remain. The best subsequent approach would depend on the specific details of the patient's case and should be discussed with the treating physician.  The return of MRD could suggest that more intensive treatment is needed. One potential option is to proceed to allogeneic hematopoietic stem cell transplantation (HSCT), which can offer the possibility of cure. The decision to move forward with allogeneic HSCT should take into account several factors, including the patient's age, overall health, availability of a suitable donor, and the potential risks and benefits of the procedure.  Alternatively, additional cycles of consolidation therapy could be considered. The exact regimen would be selected based on the patient's prior treatments and how well they were tolerated. The goal would be to achieve a deeper remission before considering other interventions, like allogeneic HSCT.  It's also worth noting that the field of AML treatment is rapidly evolving, with new targeted therapies being developed for certain genetic subtypes of AML. For example, FLT3 inhibitors for FLT3-mutated AML or IDH inhibitors for IDH-mutated AML. Although these specific agents may not apply in this case (since the patient has NPM1-mutated AML), it illustrates the importance of discussing the case with an AML specialist who can provide the most up-to-date recommendations.  Finally, it's crucial to consider enrolling the patient in a clinical trial, if available. Clinical trials often provide access to new and promising therapies that may not be available otherwise.  Please note that the best approach to treatment can vary depending on the specifics of the patient's disease and their overall health, so it's important to consult with the treating physician for the most accurate advice. As of my last training data in September 2021, this represents the general approach, but treatment strategies may have evolved since then. | As of my last update in September 2021, a patient with NPM1-mutated acute myeloid leukemia (AML) who experiences a return of minimal residual disease (MRD) after consolidation therapy could be considered for several treatment options. The best subsequent approach would largely depend on the individual patient's circumstances, including their overall health status, any concurrent conditions, and the specifics of their disease. The treating oncologist or hematologist is the best source for the most accurate and personalized advice.  However, some general approaches might include:  1. **Further chemotherapy**: An additional cycle of high-dose cytarabine, if not already given, or a different consolidation chemotherapy regimen might be an option.  2. **Targeted therapy**: If the patient has not already been on a targeted therapy, such as a FLT3 or IDH inhibitor (if these mutations are also present), this could be an option to consider.  3. **Allogeneic stem cell transplantation**: If the patient is eligible, an allogeneic hematopoietic stem cell transplantation (HSCT) could be considered. This is often the preferred option for young and fit patients who have high-risk disease or have relapsed/refractory disease. An allogeneic HSCT uses donor cells and can provide a potentially curative treatment, but it also carries significant risks, including graft-versus-host disease (GVHD) and other potential complications.  The landscape of AML treatment is rapidly evolving with ongoing research and the development of new therapies. There may be other options available depending on the most current research, clinical trials, and updated guidelines, which a treating physician would be aware of. It's always important to have a detailed discussion with the healthcare team to understand the potential benefits and risks of each treatment option. | As of my last update in September 2021, for a young adult patient with NPM1-mutated acute myeloid leukemia (AML) with normal karyotype who has returned to minimal residual disease (MRD) positivity after initial treatment, additional treatment would generally be recommended to try to achieve MRD negativity again, as MRD positivity can be associated with a higher risk of relapse.  However, the specifics of the additional treatment can depend on several factors, including the patient's overall health and the specific details of their disease. Some possible approaches could include:  1. **Further Chemotherapy:** The patient might receive further cycles of consolidation chemotherapy to try to eliminate the remaining disease. The specific regimen would depend on what the patient has already received and how they tolerated it.  2. **Allogeneic Hematopoietic Stem Cell Transplant (HSCT):** If the patient is eligible, an allogeneic HSCT could be considered. This approach can be curative for some patients with AML, although it carries significant risks and is typically reserved for cases where the potential benefits outweigh the risks, such as those with a high risk of relapse.  3. **Targeted Therapy:** If available, targeted therapies that are specific for the NPM1 mutation could be considered. This would depend on the availability of such therapies and the specifics of the patient's disease.  4. **Clinical Trial:** Given the relapsed or refractory nature of the disease, enrollment in a clinical trial investigating newer therapies or treatment strategies could be considered.  The best approach for a given patient should be decided in consultation with the patient's healthcare provider, who can provide guidance based on the most up-to-date evidence and the specific circumstances of the patient. Please note that guidelines and standard of care may have evolved since my last training data in September 2021, and always consult with the treating physician for the most accurate and current information. |
| P15 | When choosing a donor for allogeneic hematopoietic stem cell transplantation (HSCT), there are multiple factors to consider. The major consideration is Human Leukocyte Antigen (HLA) matching, but you mentioned that both potential donors are fully matched in terms of HLA. Other factors that might influence the decision include donor age, sex, CMV (Cytomegalovirus) status, and previous pregnancies in female donors.  In this scenario, given that both are HLA-matched and of the same ABO group, the preference would typically be for the younger, male donor. Here's why:  1. **Donor age:** Younger donors are generally preferred over older ones as the age of the donor can influence the outcomes of HSCT. Younger donors' stem cells may lead to better engraftment and potentially better transplant outcomes.  2. **Donor sex:** The sex of the donor can also impact transplant outcomes. Some studies suggest that male donors may be preferred due to the potential risk of graft-versus-host disease (GVHD) when using cells from female donors who have been previously pregnant.  3. **Previous pregnancies:** Female donors with a history of pregnancy may have alloimmunization, a condition where the immune system produces antibodies in response to exposure to foreign antigens. In the context of HSCT, this could increase the risk of graft-versus-host disease (GVHD), a serious complication where the donor cells attack the recipient's body.  Therefore, based on these factors, the 20-year-old male donor may be the preferred choice. However, the decision should always be made in consultation with the healthcare team, as it can depend on a variety of factors specific to the individual patient's condition and the donors' health status. It's always important to seek advice from healthcare providers for up-to-date and personalized medical information. | In the process of selecting the most appropriate donor for an allogeneic hematopoietic stem cell transplantation (HSCT), several factors are taken into consideration. These include the degree of human leukocyte antigen (HLA) matching, the donor's age, sex, CMV (cytomegalovirus) status, blood type, and other factors.  Given the situation you've described, where both donors are fully HLA-matched and have the same ABO blood group, the next factors to consider would be age and sex.  1. **Age:** Younger donors are generally preferred over older donors. This is because stem cells from younger donors tend to result in better engraftment and survival rates. Therefore, from an age perspective, the 20-year-old male would be preferred.  2. **Sex and Pregnancy History:** If we consider sex and pregnancy history, the situation gets a bit more complex. Male donors are typically preferred over female donors because of the risk of graft-versus-host disease (GVHD) can increase with a female donor who has been previously pregnant. This is due to the exposure to foreign (fetal) antigens during pregnancy, which can lead to the production of antibodies that might react with the recipient's tissues. Therefore, in terms of sex and pregnancy history, the 20-year-old male would also be preferred.  Taking these factors into account, based on the information you've provided, the 20-year-old male would likely be the preferred donor. However, it's important to note that these decisions are complex and should be made in consultation with the treating physician and transplant team, taking into account all relevant factors and the most current clinical evidence. Also, remember that my knowledge cut-off is as of September 2021, and best practices may have changed since then. Always consult with a healthcare provider for the most accurate and current information. | When selecting a donor for allogeneic hematopoietic stem cell transplantation (HSCT), a number of factors need to be considered, including the age and gender of the donor, as well as a woman donor's history of pregnancies.  In this scenario, between the two fully HLA-matched, same ABO group donors - a 20-year-old male and a 42-year-old female with a history of two pregnancies - the 20-year-old male would generally be preferred.  Here's why:  1. **Age:** Younger donors are typically preferred over older donors. This is because stem cell function can decline with age, and stem cells from younger donors may have a greater ability to engraft and produce new blood cells.  2. **Gender:** Male donors are often preferred over female donors, especially if the female donor has a history of pregnancy. This is because pregnancy can lead to the development of certain immune cells (known as HLA antibodies) that can potentially increase the risk of graft-versus-host disease (GVHD) in the recipient.  3. **Pregnancy History:** A history of pregnancy in a female donor is associated with an increased risk of GVHD due to the reasons stated above.  Therefore, taking these factors into account, the 20-year-old male donor would generally be the preferred choice. However, the final decision should always be made in consultation with the patient's transplant team, who can evaluate the specifics of the patient's case and the potential risks and benefits of each donor. |
| P16 | When evaluating a cord blood unit for hematopoietic stem cell transplantation (HSCT), several key parameters are usually considered:  1. **Human Leukocyte Antigen (HLA) Match:** Similar to other types of HSCT, the degree of HLA match between the cord blood unit and the recipient is a key consideration. While a perfect match is ideal, cord blood transplants can tolerate a greater degree of mismatch compared to bone marrow or peripheral blood stem cell transplants.  2. **Cell Dose:** The total nucleated cell (TNC) dose and the CD34+ cell dose are important. These are measures of the number of cells in the cord blood unit. Generally, larger cell doses are associated with higher rates of engraftment and better overall survival. The cell dose is particularly important in cord blood transplantation, as cord blood units tend to contain fewer cells than other stem cell sources. This is also especially crucial for larger patients, as cell doses are often adjusted for the patient's body weight.  3. **Volume and Quality of the Cord Blood Unit:** The volume of the cord blood unit and other quality parameters (like cell viability) are also taken into account.  4. **Infectious Disease Screening:** The cord blood unit is screened for infectious diseases to ensure safety.  5. **Degree of HLA Antibody Reactivity:** The presence of donor-specific anti-HLA antibodies in the recipient's serum should also be checked, as these can increase the risk of graft failure.  The evaluation of a cord blood unit for transplantation requires a thorough assessment of these and other factors. The final decision should always be made in consultation with the patient's transplant team, who can evaluate the specifics of the patient's case and the potential risks and benefits of each cord blood unit. | When evaluating a cord blood unit (CBU) for transplant in an adult patient with a hematologic malignancy, several important parameters need to be assessed:  1. **Human Leukocyte Antigen (HLA) Match:** The degree of HLA match between the patient and the cord blood unit is crucial. Ideally, a 6/6 or 8/8 match (depending on whether you're considering HLA-A, -B, and -DR, or also including HLA-C and -DQ) would be preferred, but due to the higher degree of HLA tolerance with cord blood, a 4/6 match may still be considered acceptable.  2. **Cell Dose:** The total nucleated cell (TNC) count and the CD34+ cell count (which represents the hematopoietic stem and progenitor cells) are key parameters. A higher cell dose is associated with better engraftment and survival rates. This is particularly important in adult patients, as they generally require higher cell doses than pediatric patients.  3. **Viability of Cells:** After thawing the cord blood unit, the percentage of cells that remain viable or "alive" is important. High post-thaw viability is associated with better engraftment.  4. **Infectious Disease Status:** Cord blood units are tested for various infectious diseases. A cord blood unit that tests positive for certain infections may not be used.  5. **Blood Type:** Although ABO incompatibility is more tolerable in cord blood transplant compared to bone marrow or peripheral blood stem cell transplant, it's still a factor that's considered.  6. **Degree of HLA-Antibody Reactivity:** This measures the patient's level of preformed antibodies against the HLA antigens of the cord blood unit, known as the donor-specific antibody (DSA). High levels of DSA may increase the risk of graft failure.  Given the importance of these factors, especially cell dose, many transplants in adults use a "double cord" approach, where two cord blood units are transplanted to increase the total number of stem cells transplanted.  Please consult with a healthcare professional or transplant center for the most accurate and current information. The field of hematopoietic stem cell transplantation continues to evolve, and new research or clinical practices may have emerged since my last training data in September 2021. | When considering a cord blood transplant for an adult patient with a hematologic malignancy, several parameters related to the cord blood unit(s) need to be evaluated:  1. **Human Leukocyte Antigen (HLA) Match:** The degree of HLA match between the donor cord blood unit and the recipient is crucial. Unlike bone marrow or peripheral blood stem cell transplants, a perfect match is not necessary for cord blood transplants, but at least a 4/6 HLA match is typically required, and a higher degree of match is generally associated with better outcomes.  2. **Cell Dose:** The total nucleated cell (TNC) count and/or CD34+ cell count of the cord blood unit is another key factor. This reflects the number of hematopoietic stem cells in the unit, which can affect both the success of the engraftment and the speed of blood count recovery after transplant. Higher cell doses are generally associated with better outcomes, and this is particularly important for adult recipients due to their larger size compared to pediatric patients.  3. **Infectious Disease Screening:** Just as with other types of donors, cord blood units are tested for infectious diseases, including HIV, hepatitis B and C, and others, to prevent transmission of these diseases to the recipient.  4. **Volume:** The total volume of the cord blood unit is also often considered. A larger volume may indicate a higher cell count, although volume does not always correlate exactly with cell dose.  5. **Double Cord Blood Transplant:** If a single cord blood unit does not provide a sufficient cell dose, it may be possible to use two units in a double cord blood transplant.  It's important to note that the specifics can vary depending on the practices of the transplant center and the individual patient's circumstances. Please consult with a healthcare provider for the most accurate and current information. |
| P17 | The Hematopoietic Cell Transplantation-Comorbidity Index (HCT-CI) is a tool used to predict mortality risk after hematopoietic stem cell transplantation (HSCT). This tool takes into account various comorbidities which can influence the outcome of the transplantation. Each comorbidity is assigned a specific score and the total score indicates the mortality risk.  In the case of the 32-year-old patient you described, with a BMI of 30 and a medical history of basal cell cancer not requiring systemic treatment, let's look at how his HCT-CI would be calculated based on my last training data till September 2021:  1. **Age:** Age is not factored into the HCT-CI.  2. **BMI:** As per the HCT-CI, obesity (BMI > 30) is not included as a separate risk factor, unless it's associated with other metabolic or cardiovascular complications.  3. **Basal Cell Cancer:** Previous history of malignancy is included in the HCT-CI, but typically this refers to invasive malignancies that required systemic treatment. Since the patient's basal cell cancer did not require systemic treatment, it likely wouldn't be counted in the HCT-CI.  Based on this information, this patient would likely have an HCT-CI score of 0, indicating a low risk of mortality following HSCT.  Please note that the HCT-CI is just one tool used in evaluating a patient's suitability for transplantation and predicting post-transplant outcomes. The decision to proceed with HSCT should be based on a comprehensive evaluation of the patient's overall health, the specifics of their disease, the potential benefits and risks of transplantation, and the availability of alternative treatment options. Always consult with the healthcare provider or the transplant team for the most accurate information. | The Hematopoietic Cell Transplantation-Comorbidity Index (HCT-CI) is a scoring system used to predict the risk of non-relapse mortality (death due to causes other than disease relapse) following allogeneic hematopoietic stem cell transplantation (HSCT). It takes into account various comorbidities which may impact the outcome of the transplant.  As per the HCT-CI scoring system, points are assigned based on the presence and severity of specific comorbidities.  The comorbidities considered include:  1. Cardiac: heart failure, coronary artery disease, arrhythmias, and others  2. Pulmonary: moderate to severe lung disease  3. Renal: moderate to severe kidney disease  4. Hepatic: mild to moderate liver disease or severe liver disease  5. Diabetes: with end-organ damage or without  6. Infection: prior fungal or mycobacterial infections  7. Malignancy: prior solid tumor  8. Psychiatric disturbance  9. Peptic ulcer  10. Rheumatologic: inflammatory connective tissue disorders  11. Obesity: BMI ≥ 35  12. Cerebrovascular: stroke, transient ischemic attack  13. Others: any other comorbidity not listed.  Based on the information provided - the patient is a 32-year-old with acute myeloid leukemia (AML), a BMI of 30, and a history of basal cell cancer that did not require systemic treatment - the patient would not score any points according to the HCT-CI. Obesity is only assigned a score if the BMI is 35 or more, and prior solid tumors are typically only assigned a score if they required systemic treatment.  Therefore, the HCT-CI score for this patient would be 0, indicating a low risk of non-relapse mortality.  However, this is just a guide, and the final decision on suitability for transplant should be made in discussion with the healthcare team who can assess the patient's overall condition and the specific circumstances of their disease. As always, it's important to consult with a healthcare provider for the most accurate and current information. | The Hematopoietic Cell Transplantation Comorbidity Index (HCT-CI) is a tool that assesses the risk of mortality following hematopoietic stem cell transplantation (HSCT). It scores various comorbidities to predict non-relapse mortality at two years post-transplant.  Based on the information provided, this 32-year-old patient has a Body Mass Index (BMI) of 30 and a history of basal cell cancer, which did not require systemic treatment.  Here's how the HCT-CI score would be calculated:  1. **BMI of 30:** This falls into the category of being overweight, which does not give points in the HCT-CI scoring system. The HCT-CI scoring system only assigns one point for a BMI > 35.  2. **History of Basal Cell Cancer:** As per the HCT-CI, prior solid tumor treated with curative intent and no evidence of disease for at least 5 years does not add any point to the HCT-CI score.  Therefore, given the details provided, this patient's HCT-CI score would be 0, indicating a relatively low risk of non-relapse mortality following HSCT, according to the scoring system available up until my training data in September 2021.  However, it's important to note that this is a simplified overview and the final decision about the patient's eligibility for transplantation should be made by the treating physician based on a complete medical evaluation, potentially using more recent tools or information if available. |
| P18 | CD34+ cells are a type of hematopoietic stem cell found in the bone marrow. These cells are important because they have the ability to differentiate into all types of blood cells, including red blood cells, white blood cells, and platelets.  In the context of allogeneic hematopoietic stem cell transplantation (HSCT), a patient with poor graft function may not be producing adequate levels of blood cells, leading to symptoms such as anemia, increased susceptibility to infections, or excessive bleeding.  Giving a "boost" of CD34+ cells could potentially improve graft function by providing more stem cells capable of differentiating into functional blood cells. By selecting CD34+ cells specifically, healthcare providers are able to infuse a concentrated population of these hematopoietic stem cells, which can lead to a faster engraftment and recovery of blood counts.  Moreover, because CD34+ cells are relatively immature and have lower immunogenicity, there might be a lower risk of graft-versus-host disease (GVHD) compared to a complete stem cell or bone marrow transplant, although GVHD can still occur.  As always, the selection of a specific treatment strategy depends on various factors, including the patient's overall health status, the reason for the poor graft function, and the availability of a suitable stem cell source. The patient should have a detailed discussion with their healthcare provider about the potential benefits and risks.  This information should not be used as a substitute for professional medical advice. Always consult with a healthcare provider. | CD34 is a protein that is found on the surface of hematopoietic stem cells - the cells that are capable of developing into all the different types of blood cells. When a "stem cell boost" is proposed after allogeneic hematopoietic stem cell transplantation (HSCT), it often involves the infusion of additional CD34+ cells to enhance or restore graft function.  The main advantage of selecting CD34+ cells is that these are the cells capable of engraftment and long-term reconstitution of the hematopoietic system. By giving more of these cells, the hope is to improve the function of the graft, increase blood cell counts, and improve immune recovery.  CD34+ cell boosts are often given without additional conditioning chemotherapy and without additional immunosuppression, which can be an advantage for the patient, as it can avoid the side effects and complications associated with these treatments.  Another potential advantage is that CD34+ cells are less likely to contain T cells, which are responsible for graft-versus-host disease (GVHD). This could potentially reduce the risk of GVHD, a serious and potentially life-threatening complication of HSCT.  However, the decision to proceed with a CD34+ cell boost should be carefully considered by the healthcare provider and the patient, taking into account the patient's overall health status, the potential risks and benefits, and the available alternatives.  Always consult with a healthcare provider for personalized advice. This information should not be used as a substitute for professional medical advice. | CD34 is a protein that is expressed on the surface of hematopoietic stem cells, the cells in the bone marrow that give rise to all the different types of blood cells. CD34+ cells are therefore often used as a marker for hematopoietic stem cells.  In the context of a stem cell boost for a patient with poor graft function after allogeneic hematopoietic stem cell transplantation (HSCT), selecting CD34+ cells could provide several advantages:  1. Enhanced Engraftment: CD34+ cells are stem cells that have the potential to differentiate into various types of blood cells. Providing a boost of these cells can help to improve the function of the graft by increasing the number of cells available to repopulate the bone marrow and produce new blood cells. This could potentially lead to quicker recovery and better overall graft function.  2. Lower Risk of Graft-versus-Host Disease (GVHD): GVHD is a major complication of allogeneic HSCT, where the donor's immune cells attack the recipient's tissues. By selecting CD34+ cells, which are not mature immune cells, the risk of exacerbating GVHD might be reduced. This is because the boost will primarily consist of stem cells rather than mature T cells, which are primarily responsible for GVHD.  3. Increased Immune Recovery: A boost of CD34+ cells can also help to improve immune recovery by providing a source of new immune cells, which can be especially beneficial for patients who are experiencing delayed immune recovery following HSCT.  Remember, the decision to use a CD34+ cell boost, as well as the specific protocols for its administration, should be made by the treating physician based on the individual patient's condition and needs. This information should not be used as a substitute for professional medical advice. Always consult with a healthcare provider. |
| **Section 3 – infectious complication** | | | |
| I1 | Post-transplant lymphoproliferative disorder (PTLD) is a serious complication of transplantation that is often associated with the Epstein-Barr virus (EBV). EBV is a type of herpes virus and is best known for causing infectious mononucleosis, also known as "mono" or "the kissing disease". However, in the context of a weakened immune system after transplantation, EBV can lead to the uncontrolled growth of lymphocytes, resulting in PTLD.  The risk of developing PTLD is particularly high in patients who are EBV-seronegative (meaning they have never been infected with EBV before) and receive a transplant from an EBV-seropositive donor (meaning the donor has been infected with EBV in the past). This is because these patients lack the immune cells necessary to control the EBV infection, while the transplant can introduce EBV into their system. This scenario is more common in pediatric transplantation, as most adults have been infected with EBV at some point in their lives and are therefore seropositive.  However, PTLD can also occur in patients who are already EBV-seropositive, especially if their immune system is heavily suppressed to prevent transplant rejection or graft-versus-host disease. In these cases, their existing immunity to EBV may not be sufficient to control the virus.  It's important to note that not everyone who is infected with EBV will develop PTLD, and other factors can also influence the risk of this complication, including the type of transplant, the intensity of immunosuppression, and the presence of other infections or immune disorders. | Post-transplant lymphoproliferative disorder (PTLD) is often associated with Epstein-Barr virus (EBV). EBV is a type of herpesvirus that is common in the general population and is often acquired during childhood. In some cases, especially under conditions of immune suppression like after a transplant, the virus can cause cells in the immune system to grow and divide more rapidly, leading to PTLD.  The patient-donor serology most commonly associated with PTLD is when the patient (recipient) is EBV seronegative (meaning they have never been infected with EBV before the transplant) and the donor is EBV seropositive (meaning they have been infected with EBV at some point and carry the virus).  In this scenario, the patient's immune system has no existing immunity to EBV, and the immunosuppression required after transplantation can allow the virus, transferred through the donor's cells, to proliferate unchecked, potentially leading to PTLD.  It's also worth mentioning that while EBV is a major cause, PTLD can occasionally occur independent of EBV infection. It's always important to consult with healthcare professionals for precise diagnosis and treatment decisions. | Post-transplant lymphoproliferative disorder (PTLD) is often associated with Epstein-Barr virus (EBV). EBV is a common herpesvirus that can lead to proliferation of B cells, some of which may become cancerous, resulting in PTLD.  The risk of PTLD is especially high in patients who are EBV-seronegative (they have not previously been infected with EBV and therefore have no immunity to the virus) and receive an organ or stem cells from a donor who is EBV-seropositive (they have been infected with EBV in the past and have antibodies to the virus).  This scenario creates a situation in which the recipient is exposed to EBV for the first time while their immune system is suppressed (to prevent transplant rejection), which can increase the risk of EBV-induced B-cell proliferation and PTLD.  While PTLD is often associated with EBV, it's important to note that it can also occur in the absence of EBV, and it can involve T cells or NK cells in addition to B cells. Also, PTLD represents a spectrum of diseases, ranging from benign polyclonal proliferations to aggressive monoclonal lymphomas.  Prevention, early detection, and management of PTLD typically involve regular monitoring of EBV levels in the blood, reduction of immune suppression if possible, and specific treatments such as rituximab or chemotherapy for more severe cases. As always, the patient's medical team will provide the most appropriate and current guidance. |
| I2 | Sulfamethoxazole/trimethoprim (also known as co-trimoxazole or by the brand name Bactrim) is often given to patients undergoing allogeneic hematopoietic stem cell transplantation (HSCT) as a prophylaxis to prevent certain types of infections.  If a patient is not compliant with this medication, they may be at an increased risk of developing Pneumocystis jirovecii pneumonia (PJP, previously known as Pneumocystis carinii pneumonia or PCP). This is a serious and potentially life-threatening fungal infection that primarily affects the lungs.  Sulfamethoxazole/trimethoprim also has activity against certain other types of bacteria, so non-compliance could potentially increase the risk of some other bacterial infections as well.  The medical team will typically have plans to monitor and manage such risks, and they would likely address any issues of medication non-compliance with the patient. As always, patients undergoing HSCT should work closely with their healthcare team and follow all recommended preventive measures and treatments. | A patient who is non-compliant with sulfamethoxazole/trimethoprim (also known as co-trimoxazole) prophylaxis after undergoing allogeneic hematopoietic stem cell transplantation (HSCT) would be at increased risk for Pneumocystis jirovecii pneumonia (PJP, formerly known as Pneumocystis carinii pneumonia or PCP).  PJP is a serious and potentially life-threatening opportunistic fungal infection. It most commonly affects people with weakened immune systems, such as those who have undergone HSCT.  Co-trimoxazole is usually given as prophylaxis to prevent PJP in the months following HSCT, as this is when patients are particularly susceptible due to the immunosuppressive therapies used during transplant.  If a patient is not compliant with co-trimoxazole, it is important to investigate and address the reasons for non-compliance, which could include side effects, misunderstandings about the importance of the medication, or other issues. In some cases, alternative prophylactic treatments might be considered if co-trimoxazole cannot be used, but this should be discussed with the patient's healthcare provider.  It's also important to monitor for symptoms of PJP in patients who are not compliant with prophylaxis. Symptoms can include fever, cough, difficulty breathing, and fatigue. If PJP is suspected, prompt diagnosis and treatment are essential. | Sulfamethoxazole/trimethoprim (often known by the brand name Bactrim) is an antibiotic that is often given to patients after hematopoietic stem cell transplantation (HSCT) to prevent certain types of infections. One of the most important of these is Pneumocystis jirovecii pneumonia (PJP), also known as Pneumocystis carinii pneumonia (PCP).  PJP is a serious form of pneumonia caused by a fungus. This infection is particularly dangerous for patients with weakened immune systems, such as those who have undergone HSCT. Patients who are not compliant with their sulfamethoxazole/trimethoprim prophylaxis are at a significantly higher risk of developing PJP.  Additionally, sulfamethoxazole/trimethoprim can help to prevent other types of bacterial infections, including those caused by certain strains of Staphylococcus and Streptococcus, so noncompliance could potentially increase the risk of these infections as well.  If a patient is unable to tolerate or is non-compliant with sulfamethoxazole/trimethoprim, it's important that they discuss this with their healthcare provider, who may be able to suggest alternative forms of prophylaxis. |
| I3 | Letermovir is an antiviral drug that is used for the prophylaxis of cytomegalovirus (CMV) infection and disease in adult CMV-seropositive recipients [R+] of an allogeneic hematopoietic stem cell transplant (HSCT).  Letermovir works by inhibiting the CMV DNA terminase complex, which is involved in cleaving concatemeric DNA into unit-length genomes that are then packaged into preformed capsids. By inhibiting the terminase complex, letermovir prevents the CMV virus from successfully replicating and infecting new cells.  Letermovir's mechanism of action is different from other anti-CMV drugs, which generally target the viral DNA polymerase. As such, letermovir can be effective even against CMV strains that are resistant to these other drugs. However, as of my last training data up to September 2021, resistance to letermovir can develop through mutations in the terminase gene. | Letermovir is an antiviral drug that targets human cytomegalovirus (CMV). Specifically, it inhibits the CMV DNA terminase complex, which is involved in cleavage and packaging of viral DNA into the virus capsid during viral replication. By inhibiting this complex, letermovir prevents the virus from successfully replicating, which can help to control CMV infections.  Letermovir is often used for prophylaxis against CMV infection in adult CMV-seropositive recipients of an allogeneic hematopoietic stem cell transplant (HSCT). It's important to note that it is not effective against other viruses and should not be used for treatment of other types of viral infections.  As always, letermovir should be used under the guidance of a healthcare provider, and patients should discuss any concerns or side effects with their doctor. | Letermovir is an antiviral drug that targets human cytomegalovirus (CMV). It does so by inhibiting the CMV DNA terminase complex, a viral enzyme that is necessary for the cleavage and packaging of viral DNA into the virus capsid during replication. By inhibiting this complex, letermovir prevents the virus from properly assembling new virus particles, thus blocking viral replication.  Because its target is unique to the virus, and not present in human cells, letermovir can inhibit CMV replication with fewer toxic side effects compared to some other anti-CMV drugs. As of my last update in September 2021, letermovir is primarily used for the prophylaxis of CMV infection and disease in adult CMV-seropositive recipients of an allogeneic hematopoietic stem cell transplant (HSCT). Always consult with a healthcare provider for the most accurate and current information. |
| I4 | As of my knowledge cutoff in September 2021, the drug letermovir is approved by the U.S. Food and Drug Administration (FDA) for the prophylaxis (prevention) of cytomegalovirus (CMV) infection and disease in adult CMV-seropositive recipients of an allogeneic hematopoietic stem cell transplant (HSCT).  Letermovir is an antiviral drug that works by inhibiting the CMV DNA terminase complex, a viral enzyme that's necessary for the cleavage and packaging of viral DNA into the virus capsid during replication.  It's important to note that while letermovir can significantly reduce the risk of developing CMV infection following HSCT, it does not completely eliminate the risk. Also, drug approvals can change over time as new evidence emerges, so it's always important to check with a healthcare provider for the most current information. | As of my last update in September 2021, letermovir is an antiviral medication approved by the U.S. Food and Drug Administration (FDA) for the prophylaxis (prevention) of cytomegalovirus (CMV) infection and disease in adult CMV-seropositive recipients of an allogeneic hematopoietic stem cell transplant (HSCT).  Letermovir works by inhibiting the CMV DNA terminase complex, which is required for the virus to replicate. By doing this, it can effectively prevent a CMV infection from developing or progressing.  It's important to note that guidelines and drug approvals can change over time as new research and clinical evidence becomes available, so always consult with a healthcare provider for the most accurate and current information. | As of my last training data in September 2021, the drug letermovir is approved by the U.S. Food and Drug Administration (FDA) for prophylaxis (prevention) of cytomegalovirus (CMV) infection and disease in adult CMV-seropositive recipients of an allogeneic hematopoietic stem cell transplant (HSCT).  Letermovir works by inhibiting the CMV DNA terminase complex, an enzyme necessary for the replication of the virus. By blocking the assembly of new virus particles, letermovir effectively prevents CMV infection in vulnerable populations such as recipients of HSCT.  Please note that this information may be subject to change as new research is conducted and new treatments become available. Always consult with a healthcare provider or a pharmacist for the most accurate and current information. |
| I5 | Among the human herpesviruses, Herpes Simplex Virus-1 (HSV-1) is the one most commonly associated with encephalitis in the general population. However, in the context of cord blood transplant recipients and other severely immunocompromised patients, Human Herpesvirus-6 (HHV-6) has been frequently implicated in cases of encephalitis.  HHV-6 is a ubiquitous virus, and primary infection usually occurs in early childhood. Like other herpesviruses, it establishes latency after primary infection and can reactivate in states of immunosuppression.  In hematopoietic stem cell transplant (HSCT) recipients, including those who receive cord blood transplants, HHV-6 reactivation can lead to various clinical syndromes, including encephalitis. HHV-6 encephalitis typically occurs in the early post-transplant period and may present with symptoms such as confusion, disorientation, seizures, or other neurological deficits.  It's crucial to maintain a high index of suspicion for HHV-6 encephalitis in cord blood transplant recipients presenting with these neurological symptoms, as prompt diagnosis and treatment can improve outcomes. Treatment usually involves antiviral therapy with ganciclovir, foscarnet, or cidofovir.  Always consult with a healthcare provider for the most accurate and current information. | Among the human herpes viruses, Herpes Simplex Virus-1 (HSV-1) is most often associated with encephalitis in the general population. However, among recipients of cord blood transplants, the virus more often associated with encephalitis is Human Herpesvirus 6 (HHV-6).  HHV-6, and specifically the HHV-6B subtype, is known to cause post-transplant acute limbic encephalitis (PALE), which affects the limbic system of the brain and can present with symptoms such as confusion, amnesia, and seizures. This condition most commonly occurs in the weeks following transplantation.  The reason why cord blood transplant recipients are particularly at risk for HHV-6 encephalitis is thought to be due to the fact that HHV-6 can integrate into the chromosomes of cells, and it is present in a high percentage of cord blood units. When the cord blood is transplanted, the integrated virus can reactivate, leading to infection and disease.  It's important to note that while this association exists, encephalitis after cord blood transplant is still relatively rare, and prevention, monitoring, and treatment strategies are used to manage the risk. Always consult with a healthcare provider for the most accurate and current information. | Herpes simplex virus type 1 (HSV-1) is most commonly associated with encephalitis in the general population. However, among hematopoietic stem cell transplant recipients, including cord blood transplant recipients, the human herpesvirus that has been more often associated with encephalitis is Human herpesvirus 6 (HHV-6).  HHV-6 is a member of the Herpesviridae family and is known to cause roseola (a common childhood illness) in infants and young children. After the initial infection, HHV-6 remains in the body in a dormant state and can reactivate under certain conditions such as immunosuppression, which can occur after a stem cell transplant.  HHV-6 reactivation can cause a range of clinical syndromes in hematopoietic stem cell transplant recipients, including encephalitis. HHV-6 encephalitis typically occurs within weeks to a few months post-transplant, presenting symptoms may include confusion, memory loss, seizures, and other neurologic abnormalities.  It is important to monitor for HHV-6 reactivation in cord blood transplant recipients and other hematopoietic stem cell transplant recipients, and initiate antiviral treatment as necessary. The antiviral drug foscarnet is often used in these cases, though its use can be limited by nephrotoxicity.  Always consult with a healthcare provider for the most accurate and current information. |
| I6 | Rituximab is a monoclonal antibody that targets CD20, a protein found on the surface of B cells, and is used in the treatment of various conditions including certain types of lymphoma, leukemia, and autoimmune diseases. Before starting treatment with rituximab, it is necessary to perform certain serologic tests to assess the risk of infection and reactivation of certain diseases. These include:  1. **Hepatitis B and C:** Patients should be screened for Hepatitis B and C virus, as rituximab can reactivate these infections in patients who are carriers. Hepatitis B reactivation in particular can be severe and even fatal in some cases.  2. **Tuberculosis:** Although not specific to rituximab, it's generally a good idea to screen for tuberculosis (TB) before initiating any immunosuppressive therapy, as these medications can reactivate latent TB infection.  3. **HIV:** While not universally recommended, some guidelines suggest screening for HIV in patients who have risk factors, as rituximab may exacerbate the immunosuppression seen in HIV patients.  Remember, these are general guidelines and the actual pre-treatment assessment may vary based on the specific patient, the condition being treated, and local guidelines. Always consult with a healthcare provider for appropriate management. This information should not be used as a substitute for professional medical advice. | Before starting rituximab, a monoclonal antibody that targets CD20 on B cells, it's important to screen patients for several infections, due to the drug's immunosuppressive effects which can reactivate these latent infections or increase susceptibility to them.  Typically, the following serologies may be checked:  1. Hepatitis B: Before initiating rituximab, patients should be screened for Hepatitis B virus (HBV) because rituximab can cause reactivation of HBV in those who are carriers of this virus. Both Hepatitis B surface antigen (HBsAg) and Hepatitis B core antibody (anti-HBc) should be tested. If a patient tests positive, antiviral prophylaxis may be initiated and the patient should be closely monitored for signs of hepatitis.  2. Hepatitis C: While the link between rituximab and Hepatitis C reactivation is less clear than for Hepatitis B, it may still be prudent to screen for Hepatitis C antibodies in patients about to initiate rituximab, especially if other risk factors for Hepatitis C are present.  3. HIV: Rituximab can further suppress the immune system in patients with HIV, so it's important to know if the patient has this infection.  4. Tuberculosis (TB): Although not typically a serology test, screening for latent TB (often with a PPD skin test or a QuantiFERON-TB Gold blood test) may be necessary because immunosuppressive drugs like rituximab can reactivate latent TB.  It's important for the treating physician to consider the patient's individual risk factors, including their geographical location and personal and family medical history, when deciding on pre-treatment screening.  This information should not be used as a substitute for professional medical advice. Always consult with a healthcare provider. | Rituximab is a monoclonal antibody that targets the CD20 antigen on B cells and is used in the treatment of certain types of B cell malignancies and autoimmune disorders. Before initiating treatment with rituximab, it is important to assess several serologies as part of the pre-treatment evaluation.  1. Hepatitis B Virus (HBV): Rituximab can reactivate hepatitis B virus in those who are carriers or have had past infection. Therefore, it is important to check for HBV serologies (HBsAg, anti-HBc, and in certain circumstances, anti-HBs) prior to treatment. If the patient is positive, consultation with a specialist is necessary, and prophylactic antiviral therapy may be required.  2. Hepatitis C Virus (HCV): Similarly, HCV serology may also be assessed prior to treatment.  3. Human Immunodeficiency Virus (HIV): Patients with HIV have an increased risk of infections, which can be exacerbated by rituximab. Thus, it can be important to assess HIV status.  4. Tuberculosis (TB): As rituximab can affect the immune system and increase the risk of reactivation of latent infections, screening for TB might be considered, particularly in high-risk individuals.  5. Other Infections: Depending on the patient's medical history, other serologies might need to be assessed, including for infections like varicella-zoster virus, cytomegalovirus, or Epstein-Barr virus.  Remember, the necessity of these serological tests can depend on various factors including the disease being treated, the patient's personal and family medical history, geographical location, and local guidelines. The patient's physician will decide which tests are necessary based on these factors. This information should not be used as a substitute for professional medical advice. Always consult with a healthcare provider. |
| I7 | A disseminated vesicular rash in a patient undergoing HSCT could be indicative of a viral infection such as varicella-zoster virus (the virus that causes chickenpox and shingles) or herpes simplex virus. These are highly contagious and can spread through direct contact with the rash or through the air when the vesicles are broken. Therefore, it is crucial to implement appropriate infection control measures immediately.  This patient should be placed under both contact and airborne precautions. Contact precautions involve wearing personal protective equipment (PPE), including gloves and a gown, when entering the patient's room and using dedicated medical equipment. Airborne precautions are necessary because the viruses can become airborne and inhaled. This typically requires placing the patient in a private room with negative-pressure air handling, and healthcare providers should wear a N95 respirator (or a powered air-purifying respirator if available) when entering the room.  However, the specific type of precautions may vary depending on the pathogen causing the rash and the policies of the healthcare facility. In all cases, the patient's healthcare team should be notified immediately so they can confirm the diagnosis and initiate appropriate treatment. As always, consult with a healthcare provider for the most accurate and current information. | The development of a disseminated vesicular rash in a patient undergoing hematopoietic stem cell transplantation (HSCT) can be indicative of a variety of conditions, one of which is a possible varicella-zoster virus (VZV) infection, also known as shingles or chickenpox. This is especially true if the patient has been non-compliant with antiviral prophylaxis. VZV is highly contagious and can spread to others who are susceptible to the virus.  Given the highly infectious nature of VZV, both contact and airborne precautions should be implemented in addition to standard precautions until the patient is no longer infectious.  1. **Airborne precautions** involve placing the patient in an airborne infection isolation room (AIIR) with negative pressure ventilation, and healthcare workers should use N95 respirators or higher-level respiratory protection.  2. **Contact precautions** involve wearing gloves and a gown when entering the patient's room and possibly using dedicated medical equipment for that patient.  Remember, patient symptoms, their compliance to medication, and their medical history should always be taken into account when choosing the correct precautions. Always consult with a healthcare provider or infection control personnel for the most accurate and current information. | A disseminated vesicular rash in a patient undergoing hematopoietic stem cell transplantation (HSCT) can be indicative of a viral infection such as varicella-zoster virus (VZV, the virus that causes chickenpox and shingles) or herpes simplex virus (HSV). Both of these conditions are highly contagious.  In a hospital setting, both Contact and Airborne precautions should be initiated for a suspected or confirmed case of VZV, according to the Centers for Disease Control and Prevention (CDC). This means that healthcare providers should wear gloves and a gown when coming into contact with the patient or their environment, and an N95 respirator or a powered air purifying respirator (PAPR) if they will be in the patient's room or caring for the patient.  The patient should be placed in a private room with negative air pressure relative to the surrounding areas if available. In addition, the door should be kept closed and only healthcare workers who are immune to chickenpox should care for the patient.  These precautions should continue until all the lesions have crusted over, which usually takes about a week.  Patients with disseminated herpes simplex virus (HSV) infections should be placed under standard and contact precautions.  Please note that any symptomatic rash in a HSCT patient should be evaluated promptly, and the specific precautions required may vary depending on the cause and the specific circumstances. Always consult with a healthcare provider for the most accurate and current information. |
| I8 | Respiratory syncytial virus (RSV) can cause severe respiratory illness in patients undergoing hematopoietic stem cell transplantation (HSCT), particularly in the early post-transplant period.  Treatment for RSV infections in immunocompromised patients is primarily supportive, but in some cases, antiviral therapy might be indicated. As of my last update in September 2021, the antiviral medication ribavirin can be used for the treatment of severe RSV infections in HSCT recipients.  Ribavirin is a nucleoside analog that interferes with viral replication. It can be administered orally, intravenously, or by aerosol inhalation. The inhaled form is often preferred because it delivers high concentrations of the drug directly to the respiratory tract where RSV replicates, with lower systemic exposure.  It's important to note that the use of ribavirin is associated with significant side effects and potential risks, and the decision to use this medication should be based on an individualized assessment of the potential benefits and risks for each patient.  For patients with lower respiratory tract disease, combination therapy with ribavirin and a monoclonal antibody against RSV, such as palivizumab, may be considered. However, it's important to remember that palivizumab is primarily used for the prevention of severe RSV disease in high-risk pediatric patients and its use in HSCT recipients is off-label.  The approach to treatment may also depend on the severity of the disease, the patient's overall condition, and other factors. Always consult with a healthcare provider for the most accurate and current information. | Respiratory Syncytial Virus (RSV) infection can be serious, particularly in immunocompromised patients such as those undergoing hematopoietic stem cell transplantation (HSCT). Lower respiratory tract infection (LRTI) can lead to pneumonia, bronchiolitis, or other serious conditions.  As of my last update in September 2021, there is no specific antiviral medication that's FDA-approved for the treatment of RSV infection in adults. However, there are some antiviral drugs that are often used off-label in severe cases or in immunocompromised patients. These include:  1. Ribavirin: This is an antiviral drug that can be used to treat RSV infection. It's typically given by inhalation, but can also be given orally or intravenously in severe cases.  2. Palivizumab: This is a monoclonal antibody that's used to prevent severe RSV infection in certain high-risk patients. It's not typically used for treatment, but it might be considered in some cases.  Supportive care is also important for managing symptoms and preventing complications. This can include hydration, oxygen therapy, and treatment of any secondary bacterial infections if they occur.  Please note that the choice of treatment can depend on various factors, including the patient's overall health, the severity of their symptoms, and their specific circumstances. Treatment decisions should be made in consultation with a healthcare provider. As always, it's important to consult with a healthcare provider for the most accurate and current information. | Respiratory syncytial virus (RSV) can be serious in hematopoietic stem cell transplant (HSCT) patients because their immune systems are weakened. Antiviral medications can be used to treat RSV infections, although the evidence supporting their use is somewhat mixed, and there are no universally agreed-upon guidelines.  As of my knowledge cutoff in September 2021, the most common medication used to treat severe RSV infection is ribavirin, an antiviral drug. Ribavirin can be given orally, intravenously, or as an inhaled form, although the inhaled form is often preferred due to its direct delivery to the respiratory tract and fewer systemic side effects.  In addition to ribavirin, some physicians may administer intravenous immunoglobulin (IVIG) or palivizumab, a monoclonal antibody against RSV. These treatments may provide the patient with additional antibodies to help fight off the virus, but their use is typically reserved for severe cases or those not responding to other treatments, and there is some debate about their effectiveness.  In general, the specific treatment will depend on the patient's overall health, the severity of their symptoms, and the presence of any other complications. It's important to remember that treatments and guidelines may change over time as new research and clinical evidence become available, so always consult with a healthcare provider for the most accurate and current information. |
| I9 | Adenovirus infection can be a significant cause of morbidity and mortality in hematopoietic stem cell transplant (HSCT) recipients, especially in pediatric patients. The virus can affect multiple organs and present as hemorrhagic cystitis, pneumonitis, enteritis, hepatitis, or disseminated disease.  As of my knowledge cutoff in September 2021, routine adenovirus monitoring in adults undergoing allogeneic HSCT is not universally recommended and may depend on individual center practices, patient risk factors, and the clinical scenario. It's worth noting that high levels of adenovirus in the blood (viremia) are often associated with invasive disease and poor outcomes. Therefore, some centers may choose to monitor high-risk adult patients, such as those who are heavily immunosuppressed or with graft-versus-host disease, using blood samples.  That being said, since adenoviruses can cause gastrointestinal disease, testing of stool samples could be considered in patients with unexplained gastrointestinal symptoms or when adenoviremia is detected.  The approach to adenovirus monitoring may vary based on local guidelines, risk assessment, and the clinical condition of the patient. Treatments can include reduction of immunosuppression when feasible and the use of antiviral medications such as cidofovir or brincidofovir.  As always, the patient's healthcare provider will be able to provide the most current and applicable guidance. | Adenovirus can cause serious infections in people who have undergone a hematopoietic stem cell transplant (HSCT), particularly in children. Infections can manifest in various ways, including gastroenteritis, hepatitis, and pneumonitis. Adenovirus is often detected through PCR testing of blood, urine, or stool samples.  As of my last update in September 2021, routine monitoring of adenovirus in stool samples is not universally recommended for adults undergoing allogeneic HSCT. The decision to monitor for adenovirus and the specific testing approach can vary based on individual factors, including the patient's clinical condition and local institutional protocols.  However, in the context of unexplained fever or symptoms consistent with adenovirus infection, such as diarrhea, respiratory symptoms, or certain eye symptoms, testing for adenovirus, including testing of stool samples, could be considered. If a patient does test positive for adenovirus, they may be treated with antiviral medications, such as cidofovir or brincidofovir, although the evidence supporting these treatments is limited.  These recommendations could potentially change as more research becomes available, so it is always a good idea to consult with the transplant team for the most current information. | Adenovirus infections can be serious in patients undergoing allogeneic hematopoietic stem cell transplantation (HSCT) because of their immunocompromised status. These infections can involve multiple organ systems, including the gastrointestinal tract.  There isn't a consensus on routine adenovirus monitoring in adults undergoing HSCT as of my knowledge cutoff in September 2021. Adenovirus monitoring is more commonly done in pediatric HSCT patients, who are at higher risk of severe adenoviral disease compared to adults. However, it may be considered in adults who are at high risk, such as those with graft-versus-host disease (GVHD) receiving high-dose immunosuppressive therapy, or in the presence of unexplained symptoms, especially gastrointestinal or respiratory symptoms.  The monitoring of adenovirus in stool samples could potentially allow for early detection and preemptive therapy in patients at high risk of severe adenoviral disease, but this is not a universal standard and the benefits have to be weighed against the costs and potential risks of overtreatment.  The specific approach to monitoring and managing adenovirus and other viral infections in HSCT patients will depend on various factors, including institutional protocols, the patient's specific risk factors, and their clinical status. Always consult with a healthcare provider for the most accurate and current information. |
| I10 | Hemorrhagic cystitis, a condition characterized by inflammation of the bladder and hematuria, can occur as a complication of hematopoietic stem cell transplantation (HSCT) and can be caused by a variety of factors, including viral infections.  While BK virus is one of the most common viral causes of hemorrhagic cystitis in HSCT recipients, another virus that can cause this condition is the JC virus. Additionally, cytomegalovirus (CMV) and adenovirus can also cause hemorrhagic cystitis in immunocompromised patients such as HSCT recipients.  For JC virus, there is no specific antiviral treatment available as of my knowledge cutoff in September 2021. The management of JC virus-associated hemorrhagic cystitis is mainly supportive, including hydration, bladder irrigation, and pain management. If necessary, some patients may require more invasive interventions such as clot evacuation or even embolization of the bladder arteries to control bleeding.  For CMV, antiviral medications such as ganciclovir or valganciclovir could be used. Foscarnet and cidofovir are other potential treatments.  Adenovirus-associated hemorrhagic cystitis can be a challenging condition to manage due to the lack of highly effective antiviral treatments, but cidofovir may be used.  The specific approach to management will depend on a variety of factors including the patient's overall health status, the severity of the symptoms, and the identified or suspected causative agent. Always consult with a healthcare provider for the most accurate and current information. | Hemorrhagic cystitis in patients undergoing hematopoietic stem cell transplantation (HSCT) can be caused by several factors, including infections, drugs, and radiation. Viral infections, especially BK virus and adenovirus, are common causes of hemorrhagic cystitis in HSCT recipients.  If BK virus has been ruled out, adenovirus could be a potential cause. Adenovirus can cause a range of illnesses, including cystitis, and can be particularly problematic in immunocompromised individuals, such as HSCT recipients. It can be diagnosed through urine testing, similar to BK virus.  The treatment for adenoviral hemorrhagic cystitis is primarily supportive, with measures such as hydration, bladder irrigation, and pain management. There is no approved antiviral treatment for adenovirus, but the antiviral drug cidofovir has been used off-label for severe infections. This drug has potential renal toxicity, however, so it should be used with caution. In addition, reduction of immunosuppression can help the body's immune system fight off the virus, but this needs to be balanced against the risk of graft-versus-host disease.  Please remember that this advice is based on information available as of September 2021. Always consult with a healthcare provider for the most accurate and current information. | Hemorrhagic cystitis in patients undergoing hematopoietic stem cell transplantation (HSCT) can be caused by several different factors, including viral infections. If the patient has been tested for BK virus and the result was negative, another potential viral cause of hemorrhagic cystitis could be adenovirus.  Adenovirus is known to cause a range of clinical syndromes in HSCT recipients, including hemorrhagic cystitis. Other symptoms may include fever, respiratory symptoms, or gastrointestinal symptoms, depending on which organ systems are involved.  The treatment for adenovirus hemorrhagic cystitis is primarily supportive, including hydration, bladder irrigation, and pain management. In severe cases, antiviral treatment with cidofovir might be considered, although its use is not universally agreed upon due to potential nephrotoxicity. Intravenous immunoglobulin (IVIG) has also been used in some cases.  It's also important to note that reducing immunosuppression can help the immune system fight the infection, but this needs to be balanced with the risk of graft-versus-host disease (GVHD) or graft failure.  In any case, it would be important to investigate other potential causes of hemorrhagic cystitis as well, which could include chemotherapy (especially with agents like cyclophosphamide or ifosfamide), bacterial or fungal infections, bladder stones, or a bladder tumor.  Remember, it's always important to consult with a healthcare provider for the most accurate and current information. |
| I11 | As of my last update in September 2021, the general consensus is that asymptomatic BK viruria (presence of BK virus in urine) does not typically require treatment in hematopoietic stem cell transplant (HSCT) recipients.  BK virus is a common virus that most people get in childhood. In people with healthy immune systems, it remains dormant and doesn't cause any problems. However, in people with weakened immune systems, such as those who have received HSCT, BK virus can reactivate and cause disease.  In HSCT recipients, BK virus is a well-known cause of hemorrhagic cystitis, a condition that causes painful, bloody urination. However, the mere presence of the virus in the urine (viruria) without symptoms doesn't necessarily mean that disease will develop.  Treatment with antiviral drugs is typically reserved for patients who have clear symptoms of BK virus disease, such as hemorrhagic cystitis. There's no specific antiviral medication for BK virus, and the medications that are sometimes used (like cidofovir) can have significant side effects.  Instead, the primary management strategy for asymptomatic BK viruria in HSCT recipients usually involves regular monitoring and potentially adjusting the patient's immunosuppressive therapy, with the goal of allowing the immune system to control the virus.  Again, it's always important to consult with a healthcare provider for the most accurate and current information. | As of my knowledge cutoff in September 2021, asymptomatic BK viruria in hematopoietic stem cell transplantation (HSCT) recipients typically does not require treatment. BK virus is a common virus that can reactivate in immunosuppressed individuals, such as HSCT recipients, and it can be detected in the urine (viruria) of these patients.  However, viruria alone, in the absence of any symptoms, is not typically a cause for concern or an indication for treatment. The virus is often present in the urine without causing any disease. This is especially the case when the BK virus load in the urine is low.  If the patient develops symptoms of BK virus disease, such as hemorrhagic cystitis (a condition characterized by bladder inflammation and bleeding), then treatment may be required. This often involves reducing immunosuppression when possible, as well as supportive care. Antiviral therapy is not typically first-line treatment due to the lack of proven effective antiviral agents against BK virus and potential drug toxicity.  Nevertheless, management strategies may vary between institutions and physicians, and new research may change these recommendations over time. Therefore, it's always important to consult with a healthcare provider for the most accurate and current information. | BK virus is a polyomavirus that is often acquired in childhood and remains latent in the body. It can reactivate when the immune system is compromised, such as in patients who have received hematopoietic stem cell transplantation (HSCT). BK virus can cause a range of clinical syndromes, with the most common being hemorrhagic cystitis.  However, BK viruria (the presence of BK virus in the urine) is quite common in HSCT recipients and does not necessarily indicate disease. In fact, BK viruria can be found in a significant percentage of HSCT recipients, but only a minority of these patients develop symptomatic disease.  As of my knowledge cutoff in September 2021, asymptomatic BK viruria is typically not treated in HSCT recipients. Treatment is generally reserved for patients who develop symptomatic disease, such as hemorrhagic cystitis. If asymptomatic BK viruria is detected, the patient may be monitored for the development of symptoms, but treatment is not usually initiated unless symptoms develop.  Please note that this information is based on the general guidelines and practices as of 2021 and may not apply in all cases. It is always important to consult with a healthcare provider for the most accurate and current information. |
| I12 | Third-party virus-specific T lymphocytes (VSTs) are a type of adoptive immunotherapy used to treat or prevent viral infections in patients who are immunocompromised, such as those undergoing hematopoietic stem cell transplantation (HSCT).  Following HSCT, patients are at a heightened risk for serious viral infections due to the immunosuppression that is part of the transplantation process. These infections can include cytomegalovirus (CMV), Epstein-Barr virus (EBV), adenovirus, and others, which can cause significant morbidity and mortality.  Virus-specific T lymphocytes are immune cells that are specifically targeted against these viruses. They are often generated from the transplant recipient or donor by stimulating their T cells with viral antigens in the laboratory, expanding these cells, and then infusing them back into the patient to enhance their immune response against the viruses.  However, creating these VSTs in a patient-specific manner can be time-consuming and is not always feasible, especially in cases where immediate treatment is necessary. Therefore, third-party VSTs, which are prepared from healthy donors and banked for use in multiple patients, have been developed as an alternative.  These "off-the-shelf" VSTs can be matched to the patient based on human leukocyte antigen (HLA) type to increase their effectiveness and minimize the risk of graft-versus-host disease (GVHD). They offer the potential advantage of being readily available for immediate use, which can be critical for treating severe or rapidly progressing viral infections.  The use of third-party VSTs is a promising area of research, but as of my last training cutoff in September 2021, it is not yet a widely established standard of care and is generally used under the context of clinical trials or in centers with specific expertise in this area. Always consult with a healthcare provider for the most current and appropriate treatment options. | Third-party virus-specific T lymphocytes (VSTs) refer to T cells from a healthy donor (other than the original hematopoietic stem cell transplant donor) that have been selected and expanded in the lab due to their specificity against certain viruses.  Patients undergoing hematopoietic stem cell transplantation (HSCT) often have weakened immune systems and are at risk for viral infections, including those caused by cytomegalovirus (CMV), Epstein-Barr virus (EBV), adenovirus, and others. Even with antiviral drugs, these infections can sometimes be hard to control and can cause serious illness and complications.  Virus-specific T lymphocytes are a form of adoptive immunotherapy where T cells that target specific viruses are administered to the patient to help combat these infections. In some cases, these cells are taken from the transplant donor, expanded in a lab, and then given to the patient. However, preparing donor-derived VSTs can be time-consuming and is not always feasible, especially in urgent situations.  In such cases, third-party VSTs, which are "off-the-shelf" products derived from healthy donors, can be used. These cells can be readily available and can be used to treat or prevent viral infections in HSCT patients. Early research suggests these third-party VSTs can be effective in controlling viral infections in transplant recipients with acceptable levels of side effects, although more research is needed in this area.  It's important to note that the use of third-party VSTs should be guided by the treating physician, based on the individual patient's condition and needs. This information should not be used as a substitute for professional medical advice. Always consult with a healthcare provider. | Third-party virus-specific T lymphocytes (VSTs) are a type of adoptive cell therapy used as a prophylactic measure or treatment for viral infections in patients who are undergoing or have undergone hematopoietic stem cell transplantation (HSCT).  Viral infections, particularly those caused by cytomegalovirus (CMV), Epstein-Barr virus (EBV), adenovirus, and others, can be serious complications following HSCT due to the patient's weakened immune system. To help combat these infections, VSTs can be used. These are T cells that are specifically trained to recognize and attack certain viruses.  In the context of third-party VSTs, these cells are collected from a healthy, virus-immune individual, typically unrelated to the patient. They are then expanded in a lab to increase their numbers and may be further manipulated to enhance their specificity for certain viral antigens. These T cells can then be infused into the patient to help combat or prevent viral infections.  Third-party VSTs have several advantages. Unlike patient or donor-derived VSTs, third-party VSTs are readily available from cell banks, allowing for rapid administration when needed. They also seem to pose a lower risk of causing graft-versus-host disease (GVHD) compared to other types of adoptive T cell therapies, although further research is needed to fully understand this.  However, the use of third-party VSTs is still a relatively new approach and is not yet widely available. It remains an area of active research, with ongoing clinical trials to further evaluate its safety, efficacy, and optimal use.  Remember, always consult with a healthcare provider for appropriate management. This information should not be used as a substitute for professional medical advice. |
| I13 | While specific treatment decisions depend on a variety of factors including the specific bacteria isolated, its sensitivity profile, the patient's clinical status, and potential allergies or intolerances, a class of antibiotics typically avoided in patients undergoing hematopoietic stem cell transplantation (HSCT) are the aminoglycosides.  Aminoglycosides, such as gentamicin, tobramycin, and amikacin, are often avoided in HSCT patients due to their potential to cause nephrotoxicity (kidney damage) and ototoxicity (hearing damage). This is particularly a concern in HSCT patients, who are often receiving other medications that can also have nephrotoxic effects and are at an increased risk of kidney damage.  Instead, other classes of antibiotics that are effective against gram-positive bacteria, such as vancomycin, daptomycin, or linezolid, are often used in HSCT patients, depending on the specific pathogen and local antibiotic resistance patterns.  It's important to consult with an infectious disease specialist or a medical professional for a specific patient's case. The choice of antibiotic should be guided by the pathogen identified, its susceptibility pattern, the patient's organ function, potential drug interactions, and the infection's location. | In the context of hematopoietic stem cell transplantation (HSCT), it's crucial to treat bacterial infections promptly and effectively. While there are several antibiotics that could be used to treat Gram-positive bacterial infections, one class of drugs that should be used with caution or possibly avoided in patients undergoing HSCT is the glycopeptide antibiotics, which include vancomycin and teicoplanin.  The reason is that these antibiotics, especially vancomycin, are known to be nephrotoxic and ototoxic, which means they can cause kidney and hearing damage. HSCT patients often receive other treatments that can also cause kidney damage, such as certain chemotherapeutic agents and immunosuppressive drugs. Therefore, using these antibiotics can increase the risk of kidney injury in this already vulnerable population.  In addition, empirical use of vancomycin or other broad-spectrum antibiotics has been associated with the development of bacterial resistance. Therefore, they are typically reserved for cases where a Gram-positive infection is confirmed or highly suspected, and more narrow-spectrum antibiotics are not effective or appropriate.  A Gram-positive bacterial infection in a HSCT patient could potentially be treated with other classes of antibiotics, such as beta-lactams (e.g., penicillins, cephalosporins), depending on the specific bacteria identified and their susceptibility profile.  It's crucial to remember, however, that the specific choice of antibiotic will depend on many factors, including the patient's overall health, kidney function, the specific bacteria causing the infection, and local antibiotic resistance patterns. Always consult with a healthcare provider for the most accurate and current information. | When considering the treatment of a pneumonia with Gram-positive bacteria in a patient who has undergone hematopoietic stem cell transplantation (HSCT), the antibiotic drug linezolid should generally be avoided when possible.  Linezolid is a powerful antibiotic that is effective against a wide range of Gram-positive bacteria, including methicillin-resistant Staphylococcus aureus (MRSA) and vancomycin-resistant enterococci (VRE). However, linezolid has been associated with hematologic toxicities, such as thrombocytopenia (low platelet count), anemia, and neutropenia (low neutrophil count).  These side effects can be particularly problematic in HSCT recipients, who may already have compromised bone marrow function due to their underlying condition and the transplant procedure itself. Using linezolid in these patients may increase the risk of further hematologic complications.  However, it's important to note that the choice of antibiotic should always be guided by the specifics of the patient's clinical situation, including the type of bacteria identified, the patient's overall health and kidney function, potential drug interactions, and other factors. Always consult with a healthcare provider for the most accurate and current information. |
| I14 | Human herpesvirus 6 (HHV-6) is a common virus that infects most people during childhood and remains latent in the body. There are two variants, HHV-6A and HHV-6B.  Most of the time, HHV-6 remains inactive but it can reactivate, particularly in people who are immunocompromised, such as those who have undergone hematopoietic stem cell transplantation (HSCT).  However, in addition to reactivation, there is another possible explanation for a positive HHV-6 test in an asymptomatic patient. It's important to note that HHV-6 can integrate into the host genome, a phenomenon called chromosomally integrated HHV-6 (ciHHV-6). In individuals with ciHHV-6, every cell in the body has the HHV-6 DNA integrated into its own genetic material. This can lead to positive HHV-6 tests, even though the virus is not actively replicating.  In patients with ciHHV-6, viral load usually does not fluctuate significantly, and they typically do not develop symptoms of active HHV-6 infection, such as encephalitis or rash. It might be helpful to determine whether the patient has ciHHV-6, which can be done by testing a sample of the patient's hair or nails for HHV-6.  If the patient develops symptoms consistent with HHV-6 infection or if the viral load increases significantly, further evaluation and possible treatment may be needed. However, if the patient remains asymptomatic and the viral load is low and stable, no treatment may be necessary. As always, the patient should be managed based on the specifics of his clinical situation and in consultation with a healthcare provider. | Human herpesvirus 6 (HHV-6) is a common virus that infects most people at some point, usually early in life. After the initial infection, HHV-6, like other herpesviruses, remains in the body in a latent state and can reactivate at times of immune suppression.  There are two distinct species of HHV-6, HHV-6A and HHV-6B. HHV-6B is the one usually associated with disease in transplant recipients, causing conditions such as encephalitis, bone marrow suppression, and delayed engraftment.  However, in a small percentage of the population, HHV-6 is integrated into the germline (heritable) DNA and can be found in every cell of the body, a condition called inherited chromosomally integrated HHV-6 (iciHHV-6). In people with iciHHV-6, HHV-6 DNA can be detected in every cell, including blood cells, at a low level. This can result in a positive HHV-6 test even in the absence of active viral replication or disease.  It is also possible for HHV-6 to reactivate without causing symptoms, particularly in immunocompromised individuals such as those undergoing HSCT. The detection of low levels of HHV-6 in an asymptomatic individual could potentially represent such asymptomatic reactivation.  However, it's also important to keep in mind that not all HHV-6 reactivation is associated with disease, and the significance of a positive HHV-6 test in an asymptomatic patient can be difficult to interpret. Decisions about monitoring and potential treatment should be guided by the patient's overall clinical situation and the advice of their healthcare provider.  It's always important to consult with a healthcare provider for the most accurate and current information. | Human herpesvirus 6 (HHV-6) is a common virus that most people are infected with during childhood. After the primary infection, the virus can become latent and persist in the body, potentially reactivating later in life, especially under conditions of immunosuppression.  In your described scenario, where a patient undergoing hematopoietic stem cell transplantation (HSCT) has tested positive for HHV-6 with a low viral load but is asymptomatic, there are a few potential explanations:  1. **Latent infection reactivation:** As mentioned above, HHV-6 can reactivate in patients undergoing HSCT due to their immunosuppressed status. However, not all reactivations cause symptoms, especially if the viral load is low.  2. **Chromosomal integration:** In some people, HHV-6 can integrate its DNA into the chromosomes of the host's cells, a phenomenon known as chromosomally integrated HHV-6 (ciHHV-6). This is present in approximately 1% of the general population. People with ciHHV-6 will test positive for the virus in all or most cells of their body, but this does not necessarily indicate active infection.  In either case, the patient would likely be monitored for potential symptoms of HHV-6 disease, such as encephalitis or other organ involvement, especially given their immunosuppressed status. Treatment is usually reserved for patients with symptoms or high viral loads that are indicative of active disease.  It's always important to consult with a healthcare provider for the most accurate and current information. |
| I15 | Screening for multidrug-resistant organisms (MDROs) prior to allogeneic hematopoietic stem cell transplantation (HSCT) can be beneficial in certain clinical scenarios. These MDROs might include extended-spectrum beta-lactamase (ESBL)-producing Enterobacteriaceae, vancomycin-resistant enterococci (VRE), carbapenem-resistant Enterobacteriaceae (CRE), and methicillin-resistant Staphylococcus aureus (MRSA).  Rectal swab screening is one method to detect the presence of these organisms in the gastrointestinal tract. If an MDRO is detected, this information can help guide prophylactic and empiric antibiotic strategies in the peri-transplant period.  However, as of my knowledge cutoff in September 2021, routine rectal swab screening for MDROs is not universally adopted and practices can vary between different institutions. The decision to perform rectal swab screening will depend on factors such as local epidemiology of MDROs, the patient's history of antibiotic use and healthcare exposures, and the specific protocols of the transplant center.  It's also important to note that screening for certain viruses that can cause gastrointestinal disease (like norovirus or adenovirus) might be performed on stool samples in certain situations, although this is typically not done via rectal swab.  Always consult with a healthcare provider or the transplant team for the most accurate and current information. | Rectal swab screening prior to allogeneic hematopoietic stem cell transplantation (HSCT) can be helpful to detect colonization by multidrug-resistant organisms (MDROs), including vancomycin-resistant enterococci (VRE), extended-spectrum beta-lactamase (ESBL)-producing bacteria, and carbapenem-resistant Enterobacteriaceae (CRE).  Knowing a patient's colonization status prior to HSCT can help guide antibiotic prophylaxis and empiric antibiotic therapy in the event of infection, helping to reduce the risk of severe infections with these difficult-to-treat organisms. Infections with MDROs can lead to significant morbidity and mortality in HSCT recipients due to their immunosuppressed status.  However, as of my knowledge cutoff in September 2021, rectal swab screening for MDROs prior to HSCT is not universally practiced and may depend on local epidemiology, institutional protocols, and the individual patient's risk factors. It is also important to note that the benefits of such screening must be balanced against potential drawbacks, including the risk of overtreatment and the promotion of further antibiotic resistance.  Always consult with a healthcare provider for the most accurate and current information. | Yes, rectal swab screening has a role in the pre-transplant evaluation of patients undergoing allogeneic hematopoietic stem cell transplantation (HSCT).  The aim of this screening is to detect multidrug-resistant organisms (MDROs), such as vancomycin-resistant enterococci (VRE), methicillin-resistant Staphylococcus aureus (MRSA), and extended-spectrum beta-lactamase (ESBL)-producing bacteria, as well as carbapenem-resistant Enterobacteriaceae (CRE). The presence of these organisms can have significant implications for both antibiotic prophylaxis and treatment of infections after transplantation.  The early detection of these MDROs can inform decisions regarding antibiotic use in the peri-transplant period and help guide infection prevention strategies. For example, if a patient is found to be colonized with a MDRO, they may be placed in contact isolation to prevent the spread of the organism to other patients, and the choice of prophylactic and empiric antibiotics may be adjusted accordingly.  However, practices may vary between different transplant centers, and the specific approach to screening for MDROs in HSCT patients will depend on factors such as institutional protocols, the patient's specific risk factors, and the local prevalence of these organisms.  As always, it's important to consult with a healthcare provider for the most accurate and current information. |
| I16 | Vaccination is an important part of post-transplant care for patients who have undergone allogeneic hematopoietic stem cell transplantation (HSCT). However, the timing and choice of vaccines must be carefully considered, as the immune system is recovering and there are potential risks associated with certain vaccines.  Live vaccines, in particular, are generally contraindicated early after allogeneic HSCT. These vaccines contain a version of the living virus or bacteria that has been weakened (attenuated) so as not to cause disease in healthy people. However, in people with weakened immune systems, such as HSCT recipients, live vaccines can potentially cause severe or even life-threatening infections.  Live vaccines include:  1. Measles, mumps, and rubella (MMR) vaccine  2. Varicella (chickenpox) vaccine  3. Yellow fever vaccine  4. Oral polio vaccine (not used in the U.S.)  5. Intranasal influenza vaccine  6. Rotavirus vaccine  7. Zoster vaccine (Zostavax, an older shingles vaccine that is a live vaccine, not Shingrix which is non-live and preferred)  8. Bacille Calmette-Guérin (BCG) vaccine  In general, live vaccines are usually not given until at least two years post-transplant, and only if the patient's immune system has sufficiently recovered. The exact timing depends on multiple factors, including the patient's immune status, the presence of graft-versus-host disease (GVHD), and the use of immunosuppressive therapy.  Remember, vaccination schedules for HSCT recipients should be individualized and developed in consultation with a healthcare provider. Always consult with a healthcare provider for the most accurate and current information. | After an allogeneic hematopoietic stem cell transplantation (HSCT), patients are generally advised to avoid live vaccines for a certain period of time due to their immunosuppressed status. Live vaccines contain a version of the living virus or bacteria that has been weakened (attenuated). While these vaccines are generally safe in people with a healthy immune system, they could potentially cause disease in people with weakened immune systems.  Live vaccines that should be avoided in the early period post-allogeneic HSCT include:  1. Measles, Mumps, Rubella (MMR) vaccine  2. Varicella vaccine (for chickenpox or shingles)  3. Yellow Fever vaccine  4. Oral Polio vaccine  5. Nasal spray Flu vaccine (contains live attenuated influenza virus)  6. Rotavirus vaccine  7. Typhoid oral vaccine  8. Bacillus Calmette–Guérin (BCG) vaccine  9. Zoster vaccine live (Zostavax, a shingles vaccine)  Inactivated vaccines (which do not contain living virus or bacteria) or subunit, recombinant, or polysaccharide vaccines are typically safer and can be given to immunocompromised patients, although the timing and effectiveness depend on many factors, including the patient's immune status and the specific vaccine in question.  It's important to note that general recommendations can change, and decisions about vaccinations should be individualized and discussed with the healthcare team managing the patient's transplant care. Always consult with a healthcare provider for the most accurate and current information. | After an allogeneic hematopoietic stem cell transplantation (HSCT), the patient's immune system is weakened, so live vaccines are generally contraindicated due to the risk of causing disease.  As of my knowledge cutoff in September 2021, the general guideline is to wait at least 24 months post-transplant before administering live vaccines, and only if the patient is not significantly immunosuppressed. Some specific live vaccines include:  1. Measles, Mumps, Rubella (MMR)  2. Varicella-zoster (Chickenpox and Shingles)  3. Yellow fever  4. Rotavirus  5. Oral polio vaccine (OPV)  6. Bacille Calmette-Guérin (BCG)  7. Intranasal influenza  8. Live attenuated influenza vaccine (LAIV)  9. Oral typhoid  In addition, the patient should have no active graft-versus-host disease (GVHD), should not be taking immunosuppressive therapy, and should have a full immune recovery.  The non-live vaccines (such as the inactivated influenza vaccine, pneumococcal vaccine, and tetanus, diphtheria, and pertussis (Tdap) vaccine) can be given earlier, typically starting 6 months post-transplant.  It's also important to note that the COVID-19 vaccines authorized for use as of 2021 are not live vaccines and are generally recommended for HSCT recipients, although the optimal timing is still being studied.  The specific vaccination schedule can vary based on a variety of factors, so it's always important for patients to discuss this with their healthcare provider. |
| I17 | The described symptoms – skin rash, high fever, and a recent history of cord blood transplantation – are suggestive of acute graft-versus-host disease (GVHD), especially given the exclusion of infectious causes. GVHD is a common complication of allogeneic hematopoietic stem cell transplantation (HSCT), including cord blood transplantation.  The pathophysiology of GVHD involves an immune response mounted by the donor cells (the "graft") against the recipient's body (the "host"). When the donor's immune cells are infused into the patient during the transplant, they can recognize the patient's tissues as foreign and launch an immune response, leading to tissue damage.  In acute GVHD, which typically occurs within the first 100 days after transplantation, the skin, liver, and gastrointestinal tract are most commonly affected. Symptoms can include skin rash, fever, diarrhea, and jaundice, among others.  The neutropenia (low neutrophil count), anemia (low hemoglobin), and thrombocytopenia (low platelet count) are likely due to the effects of conditioning regimens for the transplant, which include chemotherapy and sometimes radiation therapy. These treatments can suppress bone marrow function, leading to reduced production of blood cells.  The definitive diagnosis of acute GVHD is typically made based on clinical symptoms and sometimes confirmed by biopsy of the affected tissue, usually the skin or the gastrointestinal tract.  The management of acute GVHD typically involves immunosuppressive therapies to reduce the immune response of the donor cells. The specific treatment will depend on the severity of the GVHD and the patient's overall health status.  As always, this is a medical emergency and needs immediate consultation with a healthcare provider for accurate diagnosis and management. | The symptoms described, particularly the skin rash and fever, occurring two weeks after a cord blood transplant could be indicative of acute graft-versus-host disease (GVHD), particularly if infectious causes have been ruled out. Acute GVHD commonly presents with skin rash, fever, and can also involve the gastrointestinal tract and the liver.  Graft-versus-host disease is a common complication of allogeneic transplants, like cord blood transplants, where stem cells are received from a donor. In GVHD, the donated cells (graft) perceive the recipient's body (host) as foreign and launch an immune response against the host tissues. The severity of GVHD can vary from mild to life-threatening.  Acute GVHD typically occurs in the first 100 days after transplant, though it can occur later. The rash typically starts on the hands and feet and can progress to the rest of the body. It's also possible for the patient to develop gastrointestinal symptoms like diarrhea and nausea/vomiting, or liver involvement leading to jaundice and liver dysfunction.  It's essential to recognize and treat GVHD promptly. If suspected, the patient would likely be started on immunosuppressive medications to mitigate the immune response, with corticosteroids being the typical first-line therapy. A biopsy of the affected skin or other tissues might be performed to confirm the diagnosis.  However, it's important to rule out other causes of these symptoms, such as infection, drug reactions, or other post-transplant complications. Please consult with a healthcare provider for the most accurate and current information. | Based on the symptoms presented (fever and skin rash), the timing (2 weeks after transplantation), and the exclusion of infectious causes, the most probable cause of these symptoms in a patient who recently received a cord blood transplant is acute graft-versus-host disease (GVHD).  Graft-versus-host disease is a complication that can occur after a stem cell or bone marrow transplant in which the newly transplanted donor cells (the "graft") attack the transplant recipient's (the "host's") body. It occurs because the donor's immune cells recognize the recipient's cells as foreign.  Acute GVHD typically occurs within the first 100 days after transplantation and commonly affects the skin, gastrointestinal tract, and liver. Skin manifestations are often the earliest sign and can include a rash and itching. Fever can also be a symptom of acute GVHD, even in the absence of infection.  Given the neutropenia (ANC 0), anemia (Hb 8.0), and thrombocytopenia (PLT 21,000), it is also important to consider other causes such as drug reactions, infections not yet identified, or hemophagocytic lymphohistiocytosis (HLH). However, these blood counts are not unusual post-transplant, and the timing and symptoms suggest acute GVHD as the most likely cause.  This patient should undergo further diagnostic testing, likely including a skin biopsy, to confirm the diagnosis and guide treatment. Prompt initiation of treatment, which usually includes immunosuppressive medications, is important to help manage symptoms and prevent further complications. Always consult with a healthcare provider for the most accurate and current information. |
| I18 | In a patient who has undergone a hematopoietic stem cell transplantation (HSCT) and subsequently develops multiple lymphadenopathy along with high Epstein-Barr Virus (EBV) viral load, there's a concern for post-transplant lymphoproliferative disorder (PTLD). PTLD is a potentially life-threatening complication and is often associated with EBV infection.  Rituximab, a monoclonal antibody that targets the CD20 antigen on B cells, is often the first line of treatment for EBV-associated PTLD. Rituximab works by depleting B cells, including those infected with EBV, thereby reducing the number of cells that can contribute to lymphoproliferation.  It's crucial that the patient's condition is managed by a healthcare provider familiar with these complexities, and the specifics of the treatment plan will be determined by the individual patient's circumstances. This information should not be used as a substitute for professional medical advice. Always consult with a healthcare provider for appropriate management. | In a patient who has undergone a hematopoietic stem cell transplantation (HSCT) and subsequently develops multiple lymphadenopathy along with high Epstein-Barr Virus (EBV) viral load, there's a concern for post-transplant lymphoproliferative disorder (PTLD). PTLD is a potentially life-threatening complication, and it's associated with EBV infection in many cases.  Rituximab, a monoclonal antibody that targets the CD20 antigen on B cells, is often the first line of treatment for EBV-associated PTLD. Rituximab works by causing the death of B cells, including those infected with EBV, thereby reducing the number of cells that can contribute to lymphoproliferation.  The choice of treatment can depend on various factors, including the patient's overall health, the specifics of their transplantation, the extent of the PTLD, and other factors. As always, the specific treatment plan should be determined by the treating physician based on the individual patient's circumstances.  Remember, always consult with a healthcare provider for appropriate management. This information should not be used as a substitute for professional medical advice. | The patient's clinical picture suggests the possibility of post-transplant lymphoproliferative disorder (PTLD), which is a complication that can occur after hematopoietic stem cell transplantation (HSCT). PTLD is often associated with Epstein-Barr Virus (EBV) infection, and can present with symptoms such as multiple lymphadenopathy and a high EBV viral load.  A commonly used treatment for EBV-associated PTLD is the monoclonal antibody rituximab, which targets the CD20 protein found on the surface of B cells, including those infected with EBV. By binding to CD20, rituximab helps to destroy these cells, which can help to control the lymphoproliferation.  This patient will likely be offered rituximab as a first-line treatment. However, the specifics of the treatment plan will depend on various factors, including the extent of the PTLD, the patient's overall health, the specifics of their HSCT, and others. As always, this plan should be determined by the treating physician based on the individual patient's circumstances.  Remember, always consult with a healthcare provider for appropriate management. This information should not be used as a substitute for professional medical advice. |
| **Section 4 – Noninfectious complication** | | | |
| C1 | Acute graft-versus-host disease (aGVHD) is a common complication after allogeneic hematopoietic stem cell transplantation (HSCT). It typically occurs within the first 100 days post-transplant but can occur later as well. The condition arises when the transplanted donor cells recognize the recipient's body tissues as foreign and mount an immune response against them.  The three main organs affected by acute GVHD are the skin, gastrointestinal (GI) tract, and liver:  1. **Skin**: This is usually the earliest and most common manifestation of aGVHD, with up to 90% of affected individuals showing skin involvement. Patients can experience a maculopapular rash, which can progress to involve the entire body in severe cases. The rash may be pruritic (itchy) and can even progress to painful blistering and desquamation (skin peeling) in severe cases.  2. **Gastrointestinal (GI) Tract**: GI involvement can cause symptoms ranging from mild nausea, anorexia, and diarrhea, to severe abdominal pain and bloody diarrhea in more severe cases. These symptoms can lead to significant fluid and electrolyte imbalances.  3. **Liver**: aGVHD can also affect the liver, leading to symptoms such as jaundice, dark urine, and pale stools due to cholestasis (bile flow blockage). Laboratory tests usually reveal elevated bilirubin and alkaline phosphatase levels.  Other less common symptoms can include fever and general malaise. In addition, aGVHD increases the risk of infections, as the treatments to control the disease often involve immunosuppressive medications.  It's important to note that the onset and severity of symptoms can vary greatly among individuals, and prompt recognition and treatment of aGVHD is crucial to prevent severe complications. Always consult with a healthcare provider for the most accurate and current information. | Acute graft-versus-host disease (GVHD) is a common and serious complication of allogeneic hematopoietic stem cell transplantation (HSCT), where donor cells recognize the recipient's cells as foreign and attack them. The condition typically occurs within the first 100 days after transplantation.  Clinical features of acute GVHD primarily involve the skin, gastrointestinal tract, and liver:  1. **Skin:** The most common manifestation of acute GVHD is skin involvement, seen in about 90% of patients. The rash often begins on the palms or soles and then spreads. It can range from erythematous maculopapular rash to severe with blistering or desquamation. In some cases, the rash can cover the entire body, a condition called erythroderma.  2. **Gastrointestinal (GI) Tract:** Acute GVHD can cause a range of GI symptoms, including nausea, vomiting, anorexia, abdominal pain, and diarrhea. The diarrhea can be severe and persistent, and in some cases, it may be bloody.  3. **Liver:** Liver involvement typically presents as jaundice due to cholestasis. Other findings may include an increase in serum bilirubin, alkaline phosphatase, and transaminases.  Other symptoms can include fatigue, weakness, and fever. It's important to note that these symptoms can also be caused by other post-transplant complications, including infection or drug reactions, so a high index of suspicion and prompt evaluation are important to make an accurate diagnosis.  The severity of acute GVHD is graded on a scale from I to IV, with grade I being mild and grade IV being severe and potentially life-threatening. The grading is based on the degree of organ involvement (skin, liver, gastrointestinal tract). Treatment typically involves immunosuppressive medications to reduce the activity of the donor immune cells.  Please note that it's always important to consult with a healthcare provider for the most accurate and current information. | Acute graft-versus-host disease (GVHD) is a common complication after allogeneic stem cell transplantation, where donor immune cells recognize the recipient's cells as foreign and initiate an immune response against them. Acute GVHD typically occurs within the first 100 days following transplantation, but it can occasionally occur later.  The clinical features of acute GVHD typically involve the skin, gastrointestinal tract, and liver, although other organs can be affected:  1. **Skin:** This is often the first organ affected. Symptoms can range from a mild, pink rash to a severe, blistering rash that covers a large portion of the body. The rash often starts on the hands and feet and can spread to the trunk. Itching and desquamation (peeling of skin) can also occur.  2. **Gastrointestinal tract:** Symptoms can include nausea, vomiting, anorexia (loss of appetite), abdominal pain, and diarrhea. In severe cases, the diarrhea can be copious and potentially life-threatening due to dehydration and electrolyte imbalance.  3. **Liver:** Liver involvement may lead to hyperbilirubinemia, hepatomegaly (enlarged liver), and elevated liver enzymes. In severe cases, this can progress to liver failure. Symptoms of liver involvement are often subtle, but may include jaundice (yellowing of the skin and eyes).  4. **Other:** Though less common, acute GVHD may also affect the eyes, leading to dry or irritated eyes and changes in vision. In some severe cases, acute GVHD can also affect the lungs, leading to shortness of breath and other respiratory symptoms.  It's important to note that the severity of GVHD can vary greatly from patient to patient, from mild symptoms to severe, life-threatening illness. The treatment typically involves immunosuppressive medications to reduce the activity of the donor immune cells. Prompt recognition and treatment of GVHD is crucial to improve patient outcomes. Always consult with a healthcare provider for the most accurate and current information. |
| C2 | The symptoms described in the patient suggest gastrointestinal involvement of acute graft-versus-host disease (GVHD), which is a common complication of hematopoietic stem cell transplantation (HSCT).  Gastrointestinal GVHD often presents with symptoms such as nausea, vomiting, loss of appetite, and diarrhea. The diarrhea can be severe and voluminous, as described for the patient. Although GVHD can affect other organs such as the skin and liver, it's possible for it to be isolated to the gastrointestinal tract.  The diagnosis of GVHD is typically confirmed through biopsy, so this patient should likely undergo endoscopy or colonoscopy with biopsy to obtain a definitive diagnosis.  The first line treatment for acute GVHD typically involves immunosuppressive therapy to reduce the activity of the donor immune cells that are attacking the patient's body. Corticosteroids such as prednisone or methylprednisolone are most commonly used. If initial steroid therapy is not effective, other treatments may be considered, including other immunosuppressants, monoclonal antibodies, or other targeted therapies.  It's crucial to begin treatment as soon as possible to reduce the severity of GVHD and prevent further complications. As always, the patient should consult with their healthcare provider for the most accurate and current information. | The symptoms described are suggestive of gastrointestinal involvement by acute graft-versus-host disease (GVHD). Acute GVHD is a common complication of hematopoietic stem cell transplantation (HSCT), typically occurring within the first 100 days post-transplant, as is the case here. The gastrointestinal (GI) tract is one of the most commonly affected organ systems in acute GVHD, with symptoms often including nausea, vomiting, anorexia, abdominal pain, and diarrhea.  In this case, the absence of other lab abnormalities and the rapid development of symptoms following HSCT strongly suggest acute GVHD as the most likely diagnosis. However, other potential causes of diarrhea in this patient population, including infections and drug side effects, should also be considered and appropriately ruled out.  In terms of treatment, the first line therapy for acute GVHD is typically systemic glucocorticoids, such as prednisone or methylprednisolone. The use of steroids is aimed at suppressing the overactive immune response that is driving the GVHD.  However, this patient should be further evaluated to confirm the diagnosis. This could involve stool tests to rule out infectious causes of diarrhea, and potentially an endoscopy with biopsy to confirm the presence of GVHD in the GI tract. As always, the exact treatment approach should be individualized based on the patient's overall health, the severity of their symptoms, and the preferences of their healthcare provider. It is essential that the patient be managed by a multidisciplinary team experienced in the management of HSCT recipients. | Based on the timing (30 days post-transplant) and the presence of significant diarrhea, the most likely diagnosis in this patient who has undergone hematopoietic stem cell transplantation (HSCT) is acute graft-versus-host disease (GVHD) affecting the gastrointestinal (GI) tract.  Acute GVHD is a serious and potentially life-threatening complication of allogeneic HSCT where the donated stem cells (the "graft") recognize the recipient's cells as foreign and attack them. The GI tract is one of the most common sites affected by acute GVHD, and diarrhea is a common symptom, especially in moderate to severe cases.  Although the patient's physical exam is unremarkable and no lab abnormalities have been reported, acute GVHD should still be highly suspected given the timing and symptoms. A stool infection workup to rule out infectious causes of diarrhea, including Clostridioides difficile, cytomegalovirus (CMV), and other bacteria and viruses, is usually done. If infectious causes are ruled out, a colonoscopy with biopsy could be performed for definitive diagnosis, but in many cases, clinical judgement may guide treatment decisions even before biopsy results are available.  The first line of treatment for acute GVHD is systemic corticosteroids, such as prednisone or methylprednisolone. Additional immunosuppressive agents might be considered if the patient doesn't respond to steroids or if GVHD recurs after the tapering of steroids.  It's crucial to consult with a healthcare provider in such a scenario as this is a serious condition requiring prompt diagnosis and treatment. |
| C3 | Acute graft-versus-host disease (GVHD) is a common and serious complication following allogeneic hematopoietic stem cell transplantation (HSCT). Systemic corticosteroids, most commonly prednisone or methylprednisolone, are the first-line treatment for acute GVHD.  As of my knowledge cutoff in September 2021, about 50% to 60% of patients respond to first-line corticosteroid treatment. The response can depend on various factors including the severity of acute GVHD, the organs involved, and the patient's overall health status.  It's important to note that while corticosteroids are the cornerstone of treatment for acute GVHD, they can have significant side effects, particularly with long-term use. These can include hyperglycemia, hypertension, weight gain, osteoporosis, mood changes, and increased risk of infections due to immunosuppression.  For patients who do not respond to initial steroid therapy, or for those who have severe GVHD, additional treatments such as second-line immunosuppressive drugs, photopheresis, or other interventions may be considered. The choice of treatment can depend on many factors and should be guided by a specialist in this field. | The response rate to initial treatment with systemic corticosteroids in acute graft-versus-host disease (GVHD) varies depending on several factors, such as the severity of the disease and the organs involved. Generally, the response rate to steroids as first-line treatment can range from 40-70%.  Patients with skin-only acute GVHD often have a better response to steroids compared to those with liver or gastrointestinal involvement. More severe acute GVHD (such as that classified as grade III-IV) tends to have a lower response rate to steroids compared to mild to moderate disease.  Furthermore, even among patients who initially respond to steroids, a significant proportion may experience steroid-refractory GVHD, which means their GVHD symptoms do not improve or worsen during steroid treatment, or GVHD recurs during the tapering of steroids. Steroid-refractory GVHD is a challenging situation and may require additional immunosuppressive agents or experimental therapies.  Because the outcome can vary significantly among individuals, it's important for patients with acute GVHD to be managed by a healthcare team with experience in this condition, who can tailor the treatment approach to the individual's circumstances and closely monitor for responses and potential complications. | The response rate to systemic steroids, the first-line treatment for acute graft-versus-host disease (GVHD), varies depending on the severity and organ involvement, but it is generally around 50% to 70% for initial treatment.  This means that approximately half to two-thirds of patients will have a partial or complete response to systemic steroids. However, a significant proportion of patients will have steroid-resistant or steroid-refractory acute GVHD, which is more challenging to treat and is associated with a poorer prognosis.  In cases where acute GVHD is not responsive to steroids, or if it recurs after treatment is stopped, additional treatments are often needed. These may include other immunosuppressive drugs, monoclonal antibodies, extracorporeal photopheresis, or investigational therapies in clinical trials.  Overall, the prognosis and response to treatment in acute GVHD can depend on many factors, including the severity of GVHD, the specific organs involved, the patient's overall health and age, the specific conditioning regimen used before transplantation, and other factors.  Keep in mind that information might have updated after my knowledge cut-off in September 2021, so always consult with a healthcare provider for the most accurate and current information. |
| C4 | Granulocyte transfusions, which are infusions of donated granulocytes (a type of white blood cell), are not typically part of the standard of care during the aplastic phase following hematopoietic stem cell transplantation (HSCT). The primary reason is that the evidence supporting their use is limited and mixed.  Granulocyte transfusions have been used as an adjunctive therapy in patients with severe neutropenia (low neutrophil count), particularly in those with severe or refractory infections that are not responding to appropriate antimicrobial therapy. However, their efficacy remains a topic of ongoing research, with some studies showing a potential benefit and others showing no clear advantage.  Further, there are potential risks associated with granulocyte transfusions, such as transfusion reactions, alloimmunization (development of antibodies against the transfused cells), and the potential for transfusion-associated graft-versus-host disease.  Instead, the standard of care for patients undergoing HSCT typically includes prophylactic antibiotics and antifungal agents to prevent infections during the period of neutropenia, careful monitoring for signs of infection, and prompt initiation of treatment for any detected infections.  However, individual patient circumstances and the current state of research may influence the use of granulocyte transfusions. Therefore, it's important to discuss the potential risks and benefits of this approach with a healthcare provider who is familiar with the patient's specific condition and the most current research.  As of my knowledge cut-off in September 2021, this is the most accurate information. Always consult with a healthcare provider for the most accurate and current information. | As of my knowledge cutoff in September 2021, granulocyte transfusions are not considered standard of care for patients undergoing hematopoietic stem cell transplantation (HSCT) during periods of aplasia (a condition in which the body does not produce enough new blood cells). Granulocyte transfusions have been used in certain circumstances, such as in patients with severe, persistent, or life-threatening infections that are not responding to appropriate antimicrobial therapy. However, their use remains somewhat controversial and not routinely recommended.  The decision to use granulocyte transfusions depends on several factors, including the patient's overall condition, the type and severity of the infection, the availability of suitable donors, and other considerations. The effectiveness of granulocyte transfusions is still a topic of ongoing research.  In most cases, the standard approach for managing aplasia after HSCT is supportive care, including transfusions of red blood cells and platelets as needed, as well as antimicrobial prophylaxis or treatment for infections. Growth factors such as granulocyte colony-stimulating factor (G-CSF) may also be used to help stimulate the production of white blood cells.  As always, decisions about individual patient care should be made in consultation with the patient's healthcare provider, taking into account the most current and relevant information available. | As of my knowledge cutoff in September 2021, granulocyte transfusions are not generally considered standard of care for patients undergoing hematopoietic stem cell transplantation (HSCT) who are in aplasia (a phase in which the patient's bone marrow is not producing new blood cells).  Granulocyte transfusions have been used as a treatment strategy in specific situations, such as for patients with severe and persistent neutropenia who have life-threatening or uncontrolled infections that are not responding to antibiotics. However, the effectiveness of this approach is still a topic of ongoing research, and the procedure comes with its own set of potential complications, including transfusion reactions, alloimmunization (developing an immune response against the transfused cells), and the potential worsening of graft-versus-host disease.  Typically, the management of aplasia in patients undergoing HSCT involves supportive care, including the use of growth factors to stimulate the bone marrow, prophylactic antibiotics to prevent infections, and close monitoring for any signs of infection or other complications.  It is important to consult with a hematologist or oncologist for personalized medical advice in such situations. Also, please note that medical recommendations could have changed beyond my knowledge cut-off in September 2021. |
| C5 | The BMT-specific Hemorrhagic Cystitis (BEDI) Scoring System is a tool used to grade the severity of hemorrhagic cystitis in patients who have undergone hematopoietic stem cell transplantation (HSCT). This scoring system takes into account four factors:  1. Bladder spasm  2. Early satiety  3. Degree of hematuria  4. Incontinence  Each of these factors is scored on a scale of 0 (not present) to 3 (severe), yielding a total score between 0 and 12.  In the specific scenario you've described, where a patient is suffering from hemorrhagic cystitis with hematuria and visible clots, the score for "Degree of hematuria" would likely be 3 (gross hematuria with clots). However, the total BEDI score would also depend on the presence and severity of the other three factors (bladder spasm, early satiety, and incontinence).  If the patient does not have bladder spasms, early satiety, or incontinence, the BEDI score would be 3. However, if any of these symptoms are present and severe, the score could be higher.  Note: This is a simplified interpretation. For accurate scoring and treatment, a clinical evaluation by a healthcare professional is required. Also, as new research and guidelines may have been published after my knowledge cutoff in September 2021, please always refer to the most recent data and consult with a healthcare provider. | The BEDI score, or the Bladder Dysfunction Evaluation Index, is a scoring system used to grade the severity of hemorrhagic cystitis in patients. As of my knowledge cut-off in September 2021, there isn't a widely agreed-upon or universally used BEDI scoring system specifically for hemorrhagic cystitis.  There are, however, different scoring systems and grading scales used to assess the severity of hemorrhagic cystitis. One such grading scale is the Droller scale, which grades hemorrhagic cystitis on a scale from I to IV, where:  - Grade I: microscopic hematuria  - Grade II: macroscopic hematuria  - Grade III: macroscopic hematuria with clots  - Grade IV: macroscopic hematuria with clots and obstruction  Based on your description, a patient with hematuria and visible clots would be classified as having Grade III hemorrhagic cystitis under this system.  Please note that medical recommendations and diagnostic systems could have changed or evolved beyond my knowledge cut-off in September 2021. Always consult with a healthcare provider for the most accurate and current information. | The Bladder Dysfunction, Etiology, Duration, Intensity (BEDI) score is a tool used to assess and grade the severity of hemorrhagic cystitis, a complication that can occur following hematopoietic stem cell transplantation (HSCT). The score ranges from 0 (no symptoms) to 10 (most severe symptoms), and is calculated based on four parameters, each scored from 0 to 2 or 3:  1. **Bladder Dysfunction:** This refers to the presence of lower urinary tract symptoms.  - No symptoms = 0 points  - Mild symptoms (e.g., nocturia, frequency) = 1 point  - Severe symptoms (e.g., dysuria, urgency) = 2 points  2. **Etiology:** This refers to the cause of the hemorrhagic cystitis.  - Unknown or drug-induced = 0 points  - Viral = 1 point  - Bacterial or fungal = 2 points  3. **Duration:** This refers to how long the symptoms have been present.  - Less than 48 hours = 0 points  - Between 48 hours and 1 week = 1 point  - More than 1 week = 2 points  4. **Intensity:** This refers to the severity of the bleeding.  - No hematuria = 0 points  - Microscopic hematuria = 1 point  - Macroscopic hematuria without clots = 2 points  - Macroscopic hematuria with clots = 3 points  Based on this scale, a patient with hemorrhagic cystitis that includes visible clots (macroscopic hematuria with clots) would receive a score of 3 for the intensity parameter. To calculate the full BEDI score, the scores for bladder dysfunction, etiology, and duration would need to be added.  Please consult with a healthcare provider for personalized medical advice in such situations, as the management of hemorrhagic cystitis may require a multidisciplinary approach, including hydration, continuous bladder irrigation, medications, and possibly invasive procedures in severe cases. |
| C6 | Poor graft function (PGF), also known as poor graft performance or poor graft syndrome, is a potential complication following hematopoietic stem cell transplantation (HSCT).  In HSCT, stem cells from a donor are infused into the recipient with the aim of replacing the recipient's own bone marrow cells, either because the recipient's cells are not functioning properly (e.g., in certain blood disorders) or because they have been destroyed by high-dose chemotherapy or radiation therapy (e.g., as part of treatment for certain cancers).  When the transplanted cells do not adequately produce new blood cells, this is referred to as poor graft function. This condition can present as one or more of the following:  1. **Pancytopenia:** A decrease in all types of blood cells (red blood cells, white blood cells, and platelets). This can lead to symptoms such as fatigue, increased risk of infections, and increased risk of bleeding.  2. **Delayed engraftment or persistent cytopenia:** Delayed recovery of blood counts, specifically neutrophils and platelets, despite a successful initial engraftment.  3. **Dysplastic changes in the bone marrow:** Changes in the size, shape, and appearance of blood cells, which can be suggestive of a problem with the bone marrow.  There can be several potential causes of poor graft function, including infection, relapse of the underlying disease, graft-versus-host disease (GVHD), certain medications, and others.  Management of PGF can be complex and depends on the underlying cause, the patient's overall health, and other factors. Possible treatments include growth factors, second HSCT, or infusion of donor lymphocytes. As always, these decisions should be made by the treating physician based on the individual patient's circumstances.  Remember, always consult with a healthcare provider for appropriate management. This information should not be used as a substitute for professional medical advice. | Poor graft function (PGF), also known as graft failure, is a complication that can occur after a hematopoietic stem cell transplant (HSCT).  There are two main types of PGF: primary and secondary.  1. Primary PGF: This is when the transplanted stem cells never engraft (take hold and start producing new cells) after transplantation. This usually becomes apparent within the first few weeks after transplantation. It's often characterized by prolonged neutropenia (low neutrophil count), thrombocytopenia (low platelet count), and anemia (low red blood cell count) despite having received the transplant.  2. Secondary PGF: This is when initial engraftment is successful, but the new cells stop functioning properly at some point after transplantation. This might result in a drop in blood counts, which can lead to anemia, increased risk of infection due to low white blood cells, or bleeding problems due to low platelets.  PGF can be caused by several factors, such as a suboptimal stem cell dose, the quality of the stem cells, certain preparative regimens, viral infections, or problems with the recipient's bone marrow microenvironment. It's a serious complication that can increase the risk of infection, bleeding, and other complications, and it often requires additional treatment, which can include growth factors, another stem cell transplant, or other therapies.  Remember, always consult with a healthcare provider for appropriate management. This information should not be used as a substitute for professional medical advice. | Poor graft function (PGF) is a complication that can occur following hematopoietic stem cell transplantation (HSCT), where the transplanted stem cells do not engraft or function as well as expected.  PGF can be categorized into two types:  1. **Primary poor graft function**, also known as primary graft failure, refers to the situation where the transplanted stem cells fail to engraft in the first place. Patients with primary graft failure do not experience the expected recovery of their blood cell counts following transplantation.  2. **Secondary poor graft function** occurs when the graft initially takes, but then the patient experiences a decline in blood cell counts or the graft is unable to adequately support normal blood cell production.  Patients with PGF may present with symptoms related to low blood counts, such as fatigue, increased risk of infection (due to low white blood cell counts), or increased risk of bleeding (due to low platelet counts).  The causes of PGF can be multifactorial, including factors related to the patient (like age, disease status, and comorbid conditions), the transplant procedure (like the conditioning regimen or stem cell source), and post-transplant complications (like infections or graft-versus-host disease).  Treatment for PGF often involves supportive care such as transfusions and treatment of infections. In some cases, a second stem cell transplantation or stem cell boost (infusion of additional stem cells) may be considered. As always, the specific management approach should be individualized based on the patient's condition and needs.  This information should not be used as a substitute for professional medical advice. Always consult with a healthcare provider. |
| C7 | The Hematopoietic Cell Transplantation-Comorbidity Index (HCT-CI) score is a tool used to assess a patient's risk of mortality after hematopoietic stem cell transplantation (HSCT). It measures the number and severity of a patient's comorbidities (other existing health conditions), which can impact the success of the transplantation and survival outcomes. The HCT-CI score is typically used in conjunction with other assessments, such as disease severity and donor match, to determine a patient's overall suitability for transplantation.  The HCT-CI includes the following factors, each assigned a certain number of points based on their severity or presence:  1. **Heart Disease:** Includes heart failure (1 point if mild, 3 points if moderate/severe), coronary artery disease (1 point), and arrhythmia (1 point).  2. **Pulmonary Disease:** Includes chronic obstructive pulmonary disease (COPD) or other restrictive lung disease (1 point if DLCO or FEV1 is 66%-80% of expected, 3 points if DLCO or FEV1 is less than 66% of expected).  3. **Liver Disease:** Includes cirrhosis (3 points if mild, 3 points if moderate/severe) and hepatitis (1 point).  4. **Renal Disease:** Includes creatinine clearance less than 60 mL/min (2 points).  5. **Diabetes:** Includes diabetes regardless of end-organ damage (1 point), and diabetes with end organ damage (3 points).  6. **Prior Solid Tumor:** Includes a history of prior solid tumor (2 points).  7. **Connective Tissue Disease/Rheumatologic:** Includes inflammatory arthritis, systemic lupus  8. **Obesity:** Includes patients with a body mass index (BMI) of 35 or higher (1 point).  9. **Peptic Ulcer Disease:** Includes history of peptic ulcer disease (1 point).  10. **Psychiatric Disturbance:** Includes psychiatric disturbances requiring therapy (1 point).  11. **Infection:** Includes the presence of active infection (1 point).  After adding up the points for each applicable factor, the total score falls into a range of 0 to over 4. Higher scores are associated with higher risk of non-relapse mortality following HSCT.  It's important to note that this tool is not perfect and other factors may also influence patient outcomes following transplantation. Decisions about transplantation should always be made in collaboration with a knowledgeable healthcare provider.erythematosus, and other connective tissue diseases (2 points). | The Hematopoietic Cell Transplantation Comorbidity Index (HCT-CI) is a scoring system used to assess the risk of non-relapse mortality in patients who are about to undergo hematopoietic cell transplantation. It takes into consideration various pre-existing medical conditions and assigns them a score based on their predicted impact on transplantation outcomes. The score is predictive of non-relapse mortality and overall survival following transplantation.  The HCT-CI scoring system includes the following factors:  1. **Cardiac**  - Mild cardiac dysfunction (e.g., symptomatic coronary artery disease, ejection fraction less than 50%) - Score 1  - Moderate/severe cardiac disease (e.g., history of congestive heart failure, symptomatic valvular disease) - Score 3  2. **Pulmonary**  - Moderate pulmonary disease (DLCO 66-80% or FEV1 66-80%) - Score 1  - Severe pulmonary disease (DLCO less than 65% or FEV1 less than 65%) - Score 3  3. **Hepatic**  - Mild liver disease (e.g., chronic hepatitis, fatty liver, fibrosis) - Score 1  - Moderate/severe liver disease (e.g., cirrhosis with portal hypertension) - Score 3  4. **Renal**  - Creatinine clearance less than 60 mL/min - Score 1  5. **Gastrointestinal, Rheumatologic, Neurologic**  - History of prior solid tumor, rheumatologic disease, peptic ulcer disease, inflammatory bowel disease, or other significant disease - Score 1  6. **Diabetes**  - Diabetes without end-organ damage - Score 1  - Diabetes with end-organ damage - Score 3  7. **Psychiatric disturbance**  - Psychiatric disturbance requiring therapy - Score 1  8. **Infection**  - Infection requiring treatment at the time of transplantation - Score 1  9. **Obesity**  - BMI >35 - Score 1  10. **Other factors**  - Other comorbidities can be included as well, based on clinical judgment.  The HCT-CI score is then calculated by adding up all the individual scores. A higher HCT-CI score represents a greater risk of non-relapse mortality following transplantation. It's important to note that decisions regarding transplant should not be based solely on HCT-CI, but the score can provide valuable information to consider when discussing the risks and benefits of transplantation with patients. | The Hematopoietic Cell Transplantation Comorbidity Index (HCT-CI) is a tool used to assess a patient's risk of complications or mortality associated with undergoing a hematopoietic cell transplant (HCT), which is used as a treatment for various blood and bone marrow diseases, including hematologic malignancies.  The HCT-CI was developed to improve upon the original Charlson Comorbidity Index by including additional factors that are specifically relevant to HCT. The HCT-CI assesses the following factors, each assigned a certain number of points based on their severity or impact:  1. Cardiac: includes heart failure (1-3 points depending on severity), coronary artery disease (1 point), and arrhythmia (1 point).  2. Pulmonary: based on a pulmonary function test, includes moderate lung disease (1 point) or severe lung disease (3 points).  3. Hepatic: includes mild liver disease (1 point), moderate/severe liver disease (3 points), and cirrhosis (3 points).  4. Renal: includes creatinine clearance < 60 ml/min (1 point).  5. Gastrointestinal: includes prior upper gastrointestinal bleeding, ulcer, or gastritis (1 point) and inflammatory bowel disease (1 point).  6. Rheumatologic: includes rheumatoid arthritis, systemic lupus erythematosus, and other similar conditions (1 point).  7. Diabetes: includes diabetes without end-organ damage (1 point) and diabetes with end-organ damage (2 points).  8. Psychiatric disturbance: includes depression, anxiety, and other psychiatric conditions (1 point).  9. Obesity: Body Mass Index (BMI) greater than 35 (1 point).  10. Infection: recent fungal or bacterial infections (1-2 points based on severity).  11. Prior solid tumor: includes a history of a solid tumor, excluding non-melanoma skin cancers (2 points).  The total score is calculated by adding up the points for each relevant factor. Higher scores indicate a higher risk of complications or mortality associated with HCT. The score helps guide clinicians in assessing the risks and benefits of transplantation for individual patients, and in discussing these risks and benefits with patients and their families. |
| C8 | The symptoms described point to a condition known as Posterior Reversible Encephalopathy Syndrome (PRES). PRES is a neurotoxic state that occurs secondary to the inability of the posterior circulation to autoregulate in response to changes in blood pressure. Hypertension is a common trigger. It is typically characterized by a relatively unique pattern of subcortical edema without infarction and can occur in a variety of medical conditions including but not limited to systemic lupus erythematosus, renal disease, high-dose chemotherapy, and hematopoietic stem cell transplantation (HSCT).  In patients with PRES, the most common presenting symptoms include headache, altered mental functioning, seizures, and visual disturbances—such as hemianopia, visual neglect, or cortical blindness. EEG can show nonspecific bilateral abnormalities, including slowing and epileptiform activity.  If an MRI is performed on a patient suspected to have PRES, it would likely show bilateral white matter edema in the posterior cerebral hemispheres, especially in the parieto-occipital regions. In addition, it may also show involvement of the frontal lobes, the cerebellum, brain stem, and even the basal ganglia and deep white matter tracts. The edema in PRES is typically vasogenic, resulting from a breakdown of the blood-brain barrier.  However, it's worth noting that while these findings are common in PRES, they are not exclusive, and other conditions can cause similar symptoms or MRI findings. This is why it's critical to consider the patient's medical history, symptoms, and all test results together when making a diagnosis. | The patient's symptoms, such as uncontrolled hypertension, vision disturbances, nonspecific bilateral abnormalities on EEG, and a history of hematopoietic stem cell transplantation (HSCT), are concerning for a diagnosis of posterior reversible encephalopathy syndrome (PRES).  Posterior reversible encephalopathy syndrome is a neurological disorder characterized by headache, altered mental functioning, seizures, and loss of vision. It is often associated with conditions such as acute or chronic renal disease, hypertension, autoimmune disorders, or following the use of certain medications, including immunosuppressive drugs often used during HSCT.  In PRES, the brain MRI typically shows a characteristic pattern of symmetrical subcortical and cortical hyperintensities on T2-weighted images in the parieto-occipital regions of the brain, reflecting vasogenic edema. These changes are most commonly seen in the posterior regions of the brain, but they can extend to involve other areas too. The important thing about PRES is that, as its name suggests, the changes are typically reversible if the underlying cause is treated promptly.  However, it's important to note that this is a tentative diagnosis based on the limited information provided, and other differential diagnoses should also be considered. The final diagnosis should be confirmed by a qualified medical professional after considering all relevant clinical information and diagnostic test results. | The clinical information provided suggests a patient who had a hematopoietic stem cell transplantation (HSCT) is now presenting with uncontrolled hypertension, progressive vision disturbances, and EEG showing nonspecific bilateral abnormalities.  These symptoms are suggestive of a neurological disorder that can occur as a serious complication of HSCT, known as Posterior Reversible Encephalopathy Syndrome (PRES). PRES is a neurotoxic state that occurs secondary to the inability of the posterior circulation to autoregulate in response to changes in blood pressure. Immunosuppressive drugs, often used during HSCT, are known risk factors for PRES.  On a brain MRI, PRES is typically characterized by bilateral, symmetrical white matter edema predominantly affecting the parieto-occipital lobes in the posterior cerebral circulation territory. However, it can also involve the frontal lobes, inferior temporal lobes, cerebellum, brainstem, and basal ganglia. The changes are best seen on T2-weighted and FLAIR sequences. Enhancement is usually absent, but may be seen and is typically mild when present. It's crucial to note that the diagnosis of PRES is mainly clinical, and the characteristic MRI findings assist in confirming the diagnosis.  It's important that this condition is recognized promptly as the syndrome can be reversible with appropriate management, usually involving control of blood pressure and modifying or discontinuing any offending medications. However, if left untreated, PRES can lead to permanent brain injury. Therefore, this is a medical emergency and needs immediate attention.  Remember, this is a suggestion based on the information provided. Clinical correlations, further diagnostic studies, and specialist consultation may be necessary. |
| C9 | The patient's bone marrow analysis showing a blast percentage of 40% indicates a high level of immature cells, which is consistent with a relapse of acute myeloid leukemia (AML).  The cytogenetic analysis showing that all cells are 46XY is an important finding. Given the patient is female and her stem cell donor is male, this result suggests that the leukemia has recurred in the donor cells rather than in the patient's original cells. This is often referred to as "donor cell leukemia". It's a relatively rare but recognized complication of allogeneic hematopoietic stem cell transplantation (HSCT), occurring in less than 1% of transplantations.  The exact cause of donor cell leukemia is unclear, but theories suggest it might be due to a combination of factors including pre-existing genetic alterations in the donated cells, the influence of the recipient's environment, or the effects of treatments given before and after transplantation.  Management of donor cell leukemia can be challenging and is typically tailored to the specific circumstances, but it may involve additional chemotherapy, targeted therapies, or even another stem cell transplant, if possible. These are complex decisions best made in consultation with an experienced hematologist-oncologist.  As always, the interpretation of these results should be confirmed by the treating physician and any plan of care should be determined in consultation with the patient and their healthcare team. | The scenario described here suggests that the female patient with acute myeloid leukemia (AML) who underwent allogeneic hematopoietic stem cell transplantation (HSCT) from a male donor is experiencing relapsed disease.  However, the cytogenetic analysis showing a 46XY karyotype in all cells complicates the picture. If all cells were 46XY, this suggests that the cells are derived from the male donor rather than the original female recipient, as her cells would show a 46XX karyotype.  One possible explanation is that the AML has relapsed, not from the original patient's leukemia cells, but from donor-derived leukemia. This is a rare but recognized complication of allogeneic HSCT, where the transplanted cells acquire mutations and develop into a new leukemia that is genetically distinct from the recipient's original disease. This is referred to as donor cell leukemia (DCL).  Donor cell leukemia should be distinguished from a relapse of the original AML because the treatment approach can be quite different. Confirming the diagnosis often involves comparing the genetic characteristics of the post-transplant leukemia cells to the pre-transplant leukemia cells, as well as to the donor's cells.  As always, this explanation fits the provided information but the exact diagnosis should be confirmed through further diagnostic studies and consultation with specialists in this field. | The information provided suggests a relapse of acute myeloid leukemia (AML) following allogeneic hematopoietic stem cell transplantation (HSCT) from a male donor.  The finding of 40% blasts in the bone marrow analysis indicates a high proportion of immature cells, which is characteristic of a leukemia relapse. In AML, a blast percentage of 20% or more is generally considered diagnostic.  The cytogenetic analysis showing that all cells are 46XY, which is consistent with a male karyotype, suggests that the cells originated from the male donor's transplanted stem cells. This is a typical finding in cases of relapse after allogeneic HSCT.  The scenario described here is referred to as "donor cell leukemia" (DCL). DCL is a rare complication of allogeneic HSCT where the donor's transplanted cells transform into leukemia cells. Although it's rare, it's a serious complication because it suggests that the transplant, which is typically the last resort for treatment, has failed.  As always, the diagnosis must be confirmed with additional testing and clinical correlation. The treatment usually involves a combination of chemotherapy, targeted therapy (if a druggable mutation is identified), and possibly a second stem cell transplant. |
| C10 | Hematopoietic stem cell (HSC) transplant can lead to several complications, regardless of the blood type of the donor or the recipient. However, when a mismatch occurs in the ABO blood group between the donor and recipient, it can specifically lead to complications like:  1. Hemolysis: This can occur during or after transplantation when the recipient's pre-existing antibodies react with the donor's blood cells, leading to their destruction. In your particular scenario, the O positive patient has anti-A antibodies in their blood, which can react with the A positive donor's red blood cells, causing hemolysis.  2. Pure Red Cell Aplasia (PRCA): This can occur post-transplant when residual host plasma cells (from the recipient) produce antibodies that kill erythroid precursor cells in the graft, causing severe anemia.  3. Graft-versus-Host Disease (GVHD): While not directly related to ABO mismatch, GVHD is a common complication in any HSC transplant when donor immune cells (T-cells in the graft) recognize the recipient's cells as foreign and attack them.  It should be noted that modern medicine has developed methods to mitigate the risks associated with ABO-incompatible HSC transplants, such as plasma exchange to remove antibodies or the use of immunosuppressive drugs. Thus, while there are risks, they can be managed in most cases under the care of experienced clinicians. | Hematopoietic stem cell (HSC) transplantation is a complex medical procedure with numerous potential complications, regardless of the blood type match. Some of the common risks include infection, graft-versus-host disease (GVHD), organ damage, infertility, and even death.  However, specific to your question, if an O positive patient receives an HSC transplant from an A positive donor, there may be an additional risk related to the ABO blood group system. This situation may lead to a condition called "passenger lymphocyte syndrome" (PLS).  Passenger lymphocyte syndrome is a type of delayed hemolytic transfusion reaction that usually occurs following ABO-incompatible solid organ or bone marrow transplants. It's caused by the "passenger" lymphocytes (immune cells) that come with the transplant. In this case, the A positive donor lymphocytes may recognize the recipient's (O positive) red blood cells as foreign and attack them. This can result in rapid destruction of the recipient's red blood cells, leading to symptoms such as fatigue, shortness of breath, rapid heart rate, and jaundice.  Though this condition can be severe, it is often self-limiting as the donor lymphocytes eventually decrease in number, but it may require supportive care or treatment in the meantime, such as blood transfusions or corticosteroids.  It's important to note that while ABO compatibility is ideal, ABO-incompatible transplants can be performed in certain circumstances. Strategies to mitigate risk, such as plasma exchange or immunosuppression, can be used to help ensure the best possible outcome. | Hematopoietic stem cell (HSC) transplantation is a complex procedure that can come with many potential complications, some of which are general to the procedure and others specific to the compatibility between the donor and the recipient. In the case of a patient with blood type O positive receiving a transplant from a donor with blood type A positive, the specific concern is the potential for ABO incompatibility reactions.  ABO incompatibility is a risk because the immune system of the recipient, who is O positive, can potentially recognize the A antigens from the donor as foreign and mount an immune response. This can potentially lead to complications including:  1. Hemolysis: The recipient's anti-A antibodies may cause destruction of the donor's A positive red blood cells, leading to hemolysis. This can result in symptoms such as fever, chills, jaundice, and anemia. It can also cause kidney damage if severe.  2. Pure red cell aplasia (PRCA): This is a condition where the body stops producing red blood cells. PRCA can occur when the recipient's antibodies attack the newly transplanted stem cells before they have a chance to fully establish and start producing blood cells.  3. Graft-versus-host disease (GvHD): While not directly related to ABO incompatibility, GvHD is a common complication of HSC transplantation where the transplanted cells mount an immune response against the recipient's body. ABO incompatibility could potentially exacerbate GvHD by stimulating the immune response.  4. Delayed red cell engraftment: This could happen due to the immune system reaction against the donor cells, causing a delay in the establishment of normal red blood cell production.  5. Transfusion reaction: If the patient requires blood transfusions after the transplant, care must be taken to ensure the blood is compatible with both the original recipient blood type (O positive) and the new blood type (A positive) to prevent a transfusion reaction.  To mitigate these risks, clinicians often take steps to manage ABO-incompatible transplants, such as using plasma exchange or immunosuppressive therapies, and careful monitoring post-transplant for signs of these complications. However, the choice to perform an ABO-incompatible transplant would only be made if no suitable ABO-compatible donor were available, as ABO compatibility is preferred to reduce these risks. |
| C11 | The clinical picture you're describing sounds like Hematopoietic Stem Cell Transplantation-Associated Thrombotic Microangiopathy (HSCT-TMA), a severe complication of HSCT. This condition is characterized by a triad of clinical features: microangiopathic hemolytic anemia, thrombocytopenia, and organ injury due to microvascular thrombosis, such as renal impairment or neurological damage.  On a peripheral blood smear, one of the most characteristic findings associated with this condition would be schistocytes. Schistocytes are fragmented red blood cells, and their presence is a hallmark of microangiopathic hemolytic anemia, which occurs when mechanical damage to red blood cells happens as they try to navigate through the narrowed and obstructed blood vessels.  In addition to schistocytes, other potential findings on a blood smear might include reticulocytosis (indicating increased red cell production in response to the hemolysis) and decreased platelets (due to the thrombocytopenia). Lab results might also show an elevated lactate dehydrogenase (LDH) level, decreased haptoglobin, and increased indirect bilirubin, all of which are indicators of hemolysis.  Cyclosporine, a calcineurin inhibitor, is known to be associated with HSCT-TMA. Stopping the drug, as you mentioned, is often one of the first steps in management, in addition to supportive care and other interventions, such as plasma exchange or eculizumab treatment.  Of course, these are general observations, and the patient's clinical condition and specific circumstances would dictate the precise interpretation and management strategy. | The signs and symptoms you've mentioned – progressive hypertension, decreased renal function, and worsening anemia and thrombocytopenia – are suggestive of thrombotic microangiopathy (TMA), a complication that can occur following hematopoietic stem cell transplantation (HSCT). This condition can be associated with the use of immunosuppressive drugs such as cyclosporine.  Thrombotic microangiopathy is characterized by the formation of small blood clots throughout the body, particularly in the small vessels of the kidney and brain. It leads to a specific form of hemolytic anemia known as microangiopathic hemolytic anemia.  On a blood smear, one would typically expect to find schistocytes. Schistocytes are fragmented red blood cells, resulting from the mechanical destruction of red blood cells as they pass through the small blood vessels filled with clots, which is characteristic of TMA. Additionally, other signs of hemolysis such as elevated lactate dehydrogenase (LDH), low haptoglobin, and indirect hyperbilirubinemia might be present in laboratory findings. | The patient's symptoms of progressive hypertension, decreased renal function, worsening anemia, and thrombocytopenia in the context of a hematopoietic stem cell transplantation (HSCT) and cyclosporine use suggest the possibility of a condition known as Hematopoietic Stem Cell Transplantation-Associated Thrombotic Microangiopathy (TA-TMA).  TA-TMA is a serious complication associated with HSCT and immunosuppressive medications such as cyclosporine. It's a type of microangiopathic hemolytic anemia characterized by damage to the small vessels in the body, leading to the formation of small clots that can disrupt blood flow and damage organs.  On a blood smear, you would expect to see:  1. Schistocytes: Fragmented red blood cells (RBCs) or "helmet cells" are a key finding in TA-TMA, indicative of the mechanical damage to RBCs as they pass through small blood vessels filled with clots. Schistocytes appear as irregular, jagged cells.  2. Increased reticulocyte count: The body responds to the anemia by producing more immature RBCs, known as reticulocytes, which may be elevated in the blood.  3. Decreased haptoglobin: This protein binds free hemoglobin released from red blood cells and might be reduced due to increased consumption.  Laboratory findings often associated with TA-TMA also include elevated lactate dehydrogenase (LDH), a marker of cell damage; decreased platelets due to consumption in clot formation; and elevated serum creatinine reflecting kidney damage.  However, these findings need to be interpreted in conjunction with clinical findings and other laboratory tests to make a definitive diagnosis. The management of TA-TMA is complex and usually involves discontinuing or reducing the dose of the causative drug, supportive care, and potentially other specific treatments. |
| C12 | Primary and secondary graft failures are complications that can occur following hematopoietic stem cell transplantation (HSCT). Here's a brief description of both:  1. **Primary Graft Failure (Primary Poor Graft Function):** This occurs when the transplanted stem cells fail to engraft, or establish themselves in the bone marrow, in the first place. This is usually confirmed by persistently low or non-recovering blood cell counts following the transplant, typically seen within the first few weeks. Causes could include issues related to the stem cell product (like insufficient stem cells), the recipient (like an immune response against the graft), or the transplant procedure itself.  2. **Secondary Graft Failure (Secondary Poor Graft Function):** This refers to a situation where the graft initially takes, but then the patient experiences a decline in blood cell counts, or the graft is unable to adequately support normal blood cell production. This typically occurs later and can be due to a variety of factors including infections, medication effects, relapse of the original disease, or immune responses such as graft-versus-host disease (GVHD).  Both conditions are serious and require prompt medical attention. The specific treatment approach depends on the underlying cause of the graft failure, but some possible treatments include:  - **Supportive care:** This involves managing the consequences of low blood cell counts, like providing transfusions for anemia or thrombocytopenia, and giving antibiotics or antiviral medications to prevent or treat infections.  - **Immunosuppression:** If the graft failure is due to an immune response against the graft, increasing or changing the immunosuppressive regimen may be considered.  - **Growth Factors:** These are substances that stimulate the bone marrow to produce more blood cells and can be given to help support the patient while other treatments are being considered.  - **Second Transplant or Stem Cell Boost:** If the graft failure is severe or doesn't respond to other treatments, a second stem cell transplant using cells from the same or a different donor, or a stem cell boost (infusion of additional stem cells), may be considered.  This information should not be used as a substitute for professional medical advice. Always consult with a healthcare provider for appropriate management. | Primary and secondary graft failure are complications that can occur following hematopoietic stem cell transplantation (HSCT), and they refer to different scenarios in which the transplanted stem cells do not function as expected.  **Primary Graft Failure** occurs when the transplanted stem cells fail to engraft in the first place. This means that the donor cells do not begin to grow and make new cells as they should. This typically becomes apparent within a few weeks of transplantation when the patient's blood cell counts do not recover as expected. The primary causes are often immune-mediated rejection of the graft, poor quality of the graft, or insufficient conditioning of the recipient's bone marrow.  **Secondary Graft Failure** occurs when initial engraftment is successful (i.e., blood counts recover), but then the patient experiences a decline in blood cell counts, or the graft cannot maintain adequate blood cell production over time. This can be due to a variety of reasons, including infection, relapse of the original disease, graft-versus-host disease (GVHD), or toxic exposure.  The **treatment** for both primary and secondary graft failure primarily involves supportive care, which can include transfusions of blood and platelets to maintain adequate blood counts, as well as treatment of any infections.  In some cases, a second stem cell transplantation might be performed, especially if there is primary graft failure. This decision is based on many factors, including the patient's overall health, the cause of the graft failure, and the availability of a suitable donor.  For secondary graft failure, the treatment will depend on the underlying cause. If an infection is the cause, it will need to be treated. If GVHD is the cause, immunosuppressive therapy may need to be adjusted.  Another potential approach is a stem cell boost, which involves the infusion of additional stem cells from the original donor, with the goal of enhancing engraftment and blood cell production.  As always, the specific management approach should be individualized based on the patient's condition and needs. Always consult with a healthcare provider. This information should not be used as a substitute for professional medical advice. | Primary and secondary graft failure are both complications that can occur following hematopoietic stem cell transplantation (HSCT), and they refer to the failure of the transplanted stem cells to engraft or function properly.  **Primary Graft Failure (Primary Poor Graft Function)**: This occurs when the transplanted stem cells fail to engraft initially. Patients with primary graft failure do not experience the expected recovery of their blood cell counts following transplantation. The primary cause is often immune-mediated rejection of the graft, but it can also be due to poor quality of the infused stem cells, infection, or the patient's underlying disease.  **Secondary Graft Failure (Secondary Poor Graft Function)**: This occurs when the graft initially takes, but then the patient experiences a decline in blood cell counts, or the graft is unable to adequately support normal blood cell production. Secondary graft failure can be due to infection, recurrence of the original disease, drug toxicity, or graft-versus-host disease (GVHD).  The treatment for both primary and secondary graft failure primarily involves supportive care, including transfusions to manage low blood counts, and antibiotics or antiviral medications to manage infections.  If graft failure persists despite supportive care, a second transplant (also known as a salvage transplant) may be considered, especially in cases of primary graft failure. In secondary graft failure, if the cause is identified to be due to GVHD, additional immunosuppressive treatments may be added.  Stem cell boost, where additional stem cells from the original donor are infused, can also be an option for some patients. The choice of treatment will depend on a variety of factors, including the patient's overall health, the specifics of their transplantation, the underlying cause of graft failure, and others.  As always, the specific treatment plan should be determined by the treating physician based on the individual patient's circumstances. This information should not be used as a substitute for professional medical advice. Always consult with a healthcare provider. |
| C13 | In this scenario, the patient with acute myeloid leukemia (AML) has relapsed after receiving a haploidentical transplant and there is demonstrated loss of patient-donor mismatched HLA in the leukemic cells.  This suggests that the leukemic cells may have evolved to escape immune recognition by the donor immune system, a phenomenon known as "immune escape." This can occur due to a variety of mechanisms, one of which is the loss of mismatched HLA antigens, making the leukemic cells "invisible" to donor T-cells.  In such a case, donor lymphocyte infusion (DLI) may not be as effective as one would hope. DLI relies on the principle of graft-versus-leukemia effect, where donor immune cells (especially T cells) attack and destroy the patient's residual leukemic cells. If the leukemic cells have lost the mismatched HLA antigens, donor lymphocytes may not be able to recognize and attack these cells effectively.  However, this doesn't mean DLI is necessarily ruled out. The decision to proceed with DLI or explore other treatment options should be based on a comprehensive evaluation of the patient's overall health status, disease characteristics, risk-benefit assessment, and potential for response to alternative treatments.  Such alternatives may include chemotherapy, targeted therapies, or re-induction followed by a second transplantation, depending on the specific subtype of AML, cytogenetic and molecular features of the leukemia, and the patient's performance status. Consulting with the patient's hematologist/oncologist would be critical in determining the best course of action in such a complex clinical scenario. | Donor lymphocyte infusion (DLI) is a strategy often used to induce a graft-versus-leukemia (GVL) effect in patients who relapse following allogeneic hematopoietic stem cell transplantation (HSCT). It involves infusing lymphocytes from the original donor into the patient with the hope that these immune cells will recognize and attack the relapsed cancer cells. This strategy is based on the premise that donor immune cells can identify the cancer cells as foreign due to their specific human leukocyte antigen (HLA) markers.  However, in the case you described where there's loss of patient-donor mismatched HLA in the leukemic cells after haploidentical transplant, it complicates the decision to offer DLI. The loss of the mismatched HLA means that these leukemic cells have developed a way to "hide" from the donor's immune cells by eliminating the very targets (HLA antigens) the donor's cells would recognize as foreign. This is a mechanism of immune escape, which allows the leukemia to relapse even in the presence of the donor immune system.  Given this loss of mismatched HLA, using DLI may be less effective because the donor lymphocytes may not be able to recognize and target the leukemic cells as efficiently. However, it's worth noting that DLI might still have some effect due to minor histocompatibility antigens which can still be recognized by the donor cells.  The decision should be individualized based on various factors like the patient's overall health, performance status, the availability of alternative therapies (like targeted therapies or clinical trials), the risk of graft-versus-host disease (GVHD), and discussions with the patient about the potential risks and benefits.  It's a complex situation and the treatment strategy would ideally be decided by a multidisciplinary team including hematologists, transplant specialists, and the patient themselves. | The patient in question appears to have relapsed acute myeloid leukemia (AML) after undergoing a haploidentical transplant, with the leukemic cells showing loss of patient-donor mismatched HLA. This phenomenon, also known as "HLA loss," represents a mechanism of immune escape where leukemic cells alter their HLA presentation to evade recognition and attack by donor immune cells.  Donor lymphocyte infusion (DLI) is a strategy sometimes used for relapsed disease after transplantation. This involves infusing the patient with lymphocytes from the original donor with the intent that these immune cells will mount an attack against the remaining leukemic cells, a reaction known as the graft-versus-leukemia (GvL) effect.  However, in this particular case where the relapsed leukemic cells have lost the patient-donor mismatched HLA, DLI may not be effective. This is because the infused donor lymphocytes typically recognize and attack leukemic cells based on their HLA differences. If the leukemic cells no longer present the mismatched HLA, the donor lymphocytes may not recognize them as foreign and thus may not mount an effective GvL response.  This decision also depends on several other factors including the patient's overall health status, the presence of graft-versus-host disease (GvHD), and the availability of other potential treatments (like re-induction chemotherapy or targeted treatments).  Therefore, while DLI can be a viable option in some cases of relapse after hematopoietic stem cell transplantation, its use in this specific case, with loss of mismatched HLA on the leukemic cells, might not be as beneficial. The management of such a case should be individualized and discussed in a multidisciplinary team setting.  As of my knowledge cutoff in September 2021, this is a complex and evolving area of medicine, and more recent studies or advancements may provide further insight or potential treatment options. It would be advisable to consult with the treating hematologist or oncologist to get the most up-to-date information. |
| C14 | Uniparental disomy (UPD) occurs when a person receives two copies of a chromosome (or part of a chromosome) from one parent and no copies from the other parent. In the context of haploidentical hematopoietic stem cell transplantation (HSCT), UPD of the short arm of chromosome 6 in leukemic cells may indicate that the relapsed acute myeloid leukemia (AML) has acquired both copies of this chromosomal region from the original patient (rather than one from the patient and one from the donor), leading to the loss of the donor's HLA haplotype.  The short arm of chromosome 6 is where the human leukocyte antigen (HLA) system genes are located. These genes are crucial for the immune system to recognize and differentiate self from non-self. If the leukemic cells show UPD of chromosome 6p, they essentially carry the recipient's HLA type and no longer express the donor's mismatched HLA. This allows the leukemic cells to evade recognition by the donor immune cells, a mechanism of immune escape which might lead to relapse after HSCT.  This loss complicates treatment as strategies relying on the donor immune response, such as donor lymphocyte infusions (DLIs), might not be effective. This is because the infused donor lymphocytes may not recognize the relapsed leukemic cells as foreign due to the loss of the mismatched HLA, and thus may not mount an effective graft-versus-leukemia (GvL) effect.  In this situation, the clinical team may consider alternative strategies like chemotherapy, targeted therapies (depending on the specific genetic mutations in the leukemic cells), or a second transplant from a different donor. However, the choice of treatment would depend on multiple factors, including the patient's overall health, the specific characteristics of the leukemia, the presence of other mutations, and the risk of treatment-related complications.  As of my knowledge cutoff in September 2021, this is a complex and evolving area of medicine, and more recent studies or advancements may provide further insight or potential treatment options. It would be advisable to consult with the treating hematologist or oncologist to get the most up-to-date information. | Uniparental disomy (UPD) is a situation where a person receives two copies of a chromosome, or part of a chromosome, from one parent and no copy from the other parent. This can potentially lead to the expression of deleterious recessive genes, or it could alter the normal balance of parental imprinted genes.  In the case of acute myeloid leukemia (AML) and haploidentical hematopoietic stem cell transplantation (HSCT), uniparental disomy could have significant implications. The short arm of chromosome 6 is where the Major Histocompatibility Complex (MHC), which includes Human Leukocyte Antigen (HLA) genes, is located. These genes are crucial for the immune system's ability to distinguish self cells from non-self cells.  If the leukemic cells have developed uniparental disomy for the short arm of chromosome 6, it might mean that they now have two copies of the HLA haplotype from one parent. If that parent is the same as the HSCT donor, this could effectively make the leukemic cells appear "self" to the donor immune cells, thereby evading the graft-versus-leukemia (GvL) effect that could help control or eliminate the leukemia.  In terms of treatment, the occurrence of UPD could make certain therapeutic options like donor lymphocyte infusion (DLI) less effective, as the donor lymphocytes may not recognize and attack the "self" appearing leukemic cells. The choice of further therapy would depend on a number of factors including the specifics of the patient's disease, their overall health status, and the availability of alternative therapeutic options.  For relapsed AML, options might include re-induction chemotherapy, targeted therapies (depending on specific mutations present in the leukemic cells), participation in a clinical trial, or, in certain cases, a second transplantation. Decisions should be individualized based on the patient's specific circumstances and made in consultation with the treating hematologist or oncologist.  Again, this is a complex and evolving area of medicine, and there may have been advancements in the understanding and treatment of this situation since my last update in September 2021. It would be advisable to seek the most current information from a healthcare provider or specialist. | Uniparental disomy (UPD) refers to a situation where an individual receives two copies of a chromosome, or part of a chromosome, from one parent and no copies from the other parent. UPD can occur in different parts of the genome and have different effects depending on where it occurs and whether it results in the overexpression or underexpression of certain genes.  In the context of Acute Myeloid Leukemia (AML) relapse after haploidentical Hematopoietic Stem Cell Transplantation (HSCT), uniparental disomy of the short arm of chromosome 6 (6p UPD) has been associated with the loss of the mismatched HLA allele in leukemic cells. This can be a mechanism of relapse after HSCT, where the leukemic cells evade the immune system by losing the HLA that is targeted by the donor's immune cells.  The loss of HLA from 6p UPD means that the leukemic cells no longer present antigens in a way that can be recognized by the donor's immune system, making immune-based treatments like Donor Lymphocyte Infusion (DLI) potentially less effective, because the donor cells may not recognize the leukemic cells as foreign. The leukemic cells essentially become 'invisible' to the immune response, allowing them to proliferate.  As for how this impacts treatment options, it can make management of the relapse more challenging. If the leukemic cells are not recognizable by the immune system, strategies that rely on a graft-versus-leukemia (GvL) effect may not be successful. However, other treatments could still be options, including chemotherapy, targeted therapies based on the specific genetic alterations in the leukemia cells, or a second transplant.  The best approach will depend on a variety of factors including the patient's overall health status, the specifics of their AML (including other genetic mutations), and the potential availability of a different donor for a second transplant.  It's important to note that this is a complex and rapidly evolving area of research, and our understanding of the implications of UPD and loss of HLA in AML and other cancers is still developing. Decisions about treatment should always be made in consultation with a knowledgeable healthcare provider, who can interpret these findings in the context of the individual patient's situation. |
| C15 | The symptoms presented in this case - rapidly progressive ascites, weight gain, increased bilirubin, and painful hepatomegaly - are suggestive of veno-occlusive disease (VOD) or sinusoidal obstruction syndrome (SOS), which is a potential complication following allogeneic hematopoietic stem cell transplantation (HSCT). This condition is associated with damage to the liver's small blood vessels, leading to hepatic congestion, fluid retention, and potential liver failure.  As of my knowledge cut-off in September 2021, the drug that is often used for severe cases of VOD/SOS with multiorgan failure and is approved by the FDA is defibrotide. Defibrotide is an antithrombotic agent that protects endothelial cells and has fibrinolytic and anti-inflammatory effects. It's often used in severe or very severe VOD/SOS, especially when there is associated kidney or lung dysfunction.  That being said, the best approach to treatment depends on a variety of factors, including the patient's overall health status and the specifics of their HSCT. Care for a patient with VOD/SOS also involves supportive measures, such as managing fluid balance, providing nutritional support, and treating any infections that may be present.  As always, all treatment decisions should be made in consultation with a knowledgeable healthcare provider, who can interpret these findings in the context of the individual patient's situation. Also, note that this field is rapidly evolving, and there may be new treatments or guidelines available after my training data in September 2021. | The patient's clinical picture -- rapidly progressive ascites, weight gain, increased bilirubin level, and painful hepatomegaly -- in the context of allogeneic hematopoietic stem cell transplantation (HSCT) is suggestive of veno-occlusive disease (VOD), also known as sinusoidal obstruction syndrome (SOS). This is a potentially serious complication of HSCT, caused by occlusion of the small veins in the liver, which leads to congestion and hepatocellular damage.  The treatment of choice for VOD/SOS, particularly in severe cases, is defibrotide. Defibrotide is a polydisperse oligonucleotide with anti-thrombotic, anti-inflammatory, and anti-ischemic properties that appears to have a protective effect on the endothelial cells of the hepatic sinusoids. It was approved by the FDA specifically for the treatment of severe hepatic VOD/SOS with renal or pulmonary dysfunction post-HSCT in adults and children.  However, it's essential that this diagnosis is confirmed by a healthcare provider, who would consider the full clinical picture and other potential causes of these symptoms, before starting treatment. Additionally, this condition can rapidly become serious, and supportive care -- including careful fluid management and treatment of any infections -- is also a crucial part of management. If the patient's symptoms continue to worsen, they should be immediately evaluated by a healthcare provider. | The condition described in the patient is likely Hepatic Veno-Occlusive Disease (VOD), also known as sinusoidal obstruction syndrome (SOS), which is a potentially serious complication of hematopoietic stem cell transplantation (HSCT). It is characterized by damage to the small vessels in the liver, which can lead to rapid weight gain, ascites (fluid build-up in the abdomen), increased bilirubin levels, and painful hepatomegaly (enlarged liver).  As of my knowledge cutoff in September 2021, the drug defibrotide is approved by the U.S. Food and Drug Administration (FDA) for the treatment of hepatic VOD/SOS with renal or pulmonary dysfunction following HSCT.  Defibrotide is believed to work by protecting the cells lining the blood vessels and by preventing clot formation, thereby improving blood flow in the liver. This can help to relieve the symptoms and potentially reverse the disease.  Keep in mind that the patient's condition needs to be carefully evaluated by their treating physicians, and the decision to use defibrotide should be based on a thorough consideration of the potential risks and benefits. Other supportive care measures and modifications to the patient's overall treatment plan may also be necessary.  Since the field of HSCT is evolving rapidly, always consult with the treating hematologist or oncologist to get the most up-to-date information. |
| C16 | The presence of specific chromosomal abnormalities in acute myeloid leukemia (AML) cells can provide important information about a patient's prognosis. However, the alterations you've listed as t(1,2) and -7 are not among the common chromosomal abnormalities typically associated with AML, and they don't correspond to the typical notation for chromosomal translocations.  Usually, translocations in AML are denoted as t(X;Y)(p;q), where X and Y are the chromosomes involved, and p and q specify the arms of the chromosomes. Some of the most well-studied translocations in AML include t(8;21)(q22;q22), t(15;17)(q22;q21), and inv(16)(p13.1q22) or t(16;16)(p13.1;q22), which are associated with specific subtypes of AML and have prognostic significance.  Loss of chromosome 7 or a part of its long arm (7q) — denoted as -7 or del(7q) — is a known poor prognostic marker in AML, associated with lower rates of remission and shorter overall survival. However, the prognosis can be improved with allogeneic hematopoietic stem cell transplantation (HSCT), which is considered the standard of care for AML patients with this cytogenetic abnormality who are eligible for transplantation.  Without more specific information about the translocation (t(1,2)), it's difficult to provide an accurate prognosis. Furthermore, factors such as the patient's age, performance status, comorbidities, response to induction therapy, and the presence of any molecular abnormalities (like mutations in FLT3, NPM1, or CEBPA) would also significantly influence the prognosis.  Given the complexities in prognostication in AML and the rapid advances in our understanding of the disease, I recommend that this question be discussed with the patient's hematologist or oncologist, who would have the most up-to-date knowledge and would be able to provide personalized prognostic information. | In acute myeloid leukemia (AML), cytogenetic analysis plays a crucial role in predicting the prognosis of the disease. The most commonly used cytogenetic risk classification is based on the data from the UK Medical Research Council (MRC) and divides patients into favorable, intermediate, and adverse risk groups.  As of my knowledge cutoff in September 2021, a translocation involving chromosomes 1 and 2 (t(1,2)) is not commonly associated with AML and its prognostic implications are not well-established. More details on the specific genes involved in the translocation would be needed to assess its potential impact on prognosis.  On the other hand, the loss of an entire copy of chromosome 7 (-7) is considered an adverse prognostic marker in AML. It is associated with poorer response to chemotherapy and a higher risk of relapse.  Therefore, based on the -7 alteration, the patient would likely be classified into the adverse risk group. In terms of survival rates, this can vary depending on various factors such as the patient's overall health, age, and response to treatment. However, as a rough estimate, the 5-year survival for patients in the adverse risk group is generally below 20%.  The decision to offer allogeneic HSCT to this patient is likely based on the poor prognosis associated with the -7 karyotype. Allogeneic HSCT offers the potential for long-term disease-free survival even in patients with adverse cytogenetic risk factors.  However, HSCT is a major procedure with significant risks, and the decision to proceed must be made in careful consultation with the patient and the treating hematologist or oncologist. Prognostic data can help inform this decision, but individual patient characteristics and preferences are also very important.  Please remember that the field of hematology and oncology is rapidly evolving, and newer treatments may have become available after my knowledge cutoff in September 2021. Therefore, it's essential to consult with a medical specialist for the most up-to-date treatment options and prognostic information. | Prognosis in Acute Myeloid Leukemia (AML) is often stratified based on the presence of certain cytogenetic and molecular alterations. AML is a very heterogenous disease and prognosis can vary greatly based on the specific genetic changes present.  In your question, you mentioned two cytogenetic alterations: a translocation between chromosomes 1 and 2 (t(1,2)) and loss of chromosome 7 (-7). It's important to clarify that while you mentioned t(1,2), translocations are typically specified with the chromosome bands involved, like t(8;21)(q22;q22), so a full karyotype with the chromosome bands specified would provide a more precise assessment.  Monosomy 7 or -7 in AML is generally considered a high-risk cytogenetic alteration, associated with a poor prognosis. Patients with this alteration tend to have a higher risk of relapse and lower overall survival rates.  However, the prognosis can be improved with allogeneic HSCT, particularly if the transplant is done during the first complete remission. Allogeneic HSCT is often recommended for patients with high-risk cytogenetics, such as -7, because it offers the potential for long-term cure.  The translocation t(1,2) is less commonly seen in AML, and its impact on prognosis is less clear. Its prognostic impact might depend on the specific genes involved in the translocation, which are not specified here.  Please note that while we can make general statements about prognosis based on cytogenetic alterations, the specific prognosis for any individual patient would depend on a variety of factors including their overall health, the presence of other genetic alterations, their response to treatment, and many others. The patient's hematologist or oncologist would be best equipped to provide a more personalized prognosis.  Finally, my knowledge cutoff is in September 2021, so there may be more recent data or advancements that could potentially affect this prognosis, and it would be advisable to consult the most recent literature or the treating oncologist for the most up-to-date information. |
| C17 | The clinical condition described seems to be Hepatic Veno-Occlusive Disease (VOD), also known as sinusoidal obstruction syndrome (SOS). This is a potentially severe complication associated with hematopoietic stem cell transplantation (HSCT). There are several transplant-related factors that have been associated with an increased risk of developing VOD/SOS:  1. **Conditioning regimen:** High-intensity, myeloablative conditioning regimens, especially those that include total body irradiation (TBI) or high-dose cyclophosphamide or busulfan, are associated with a higher risk of VOD/SOS.  2. **Allogeneic transplant:** Allogeneic HSCT (especially from unrelated or mismatched donors) have a higher risk of VOD/SOS compared to autologous transplants.  3. **Previous liver disease or damage:** Patients with previous liver damage or liver disease (e.g., hepatitis, cirrhosis) or abnormal liver function tests prior to transplant are at increased risk. This includes patients who had liver damage from chemotherapy or radiation therapy prior to the transplant.  4. **Infection:** Certain types of infection, such as fungal infections, may increase the risk of VOD/SOS.  5. **Age:** Older age (generally considered as >50 years) is associated with a higher risk.  6. **Use of certain medications:** Some drugs used for GVHD prophylaxis, like sirolimus or certain calcineurin inhibitors (cyclosporine or tacrolimus), have been associated with an increased risk of VOD/SOS.  7. **Obesity:** Patients with a high body mass index (BMI) might have an increased risk of VOD/SOS.  Prevention and early detection are key to managing VOD/SOS, so clinicians should be vigilant in monitoring liver function tests and other clinical signs in patients at high risk. If VOD/SOS is suspected, defibrotide is a drug that is approved for the treatment of severe VOD/SOS in patients after HSCT.  As always, the most recent literature and treating physician's guidance should be consulted as this is a complex and evolving area of medicine. | The condition you're describing is hepatic veno-occlusive disease (VOD), also known as sinusoidal obstruction syndrome (SOS). VOD/SOS is a potential complication of allogeneic hematopoietic stem cell transplantation (HSCT). Several transplant-related risk factors have been suggested to increase the risk of developing VOD/SOS:  1. Conditioning regimen: High-dose, myeloablative conditioning regimens, particularly those including alkylating agents such as busulfan and cyclophosphamide, are associated with a higher risk of VOD/SOS.  2. Use of Total Body Irradiation (TBI): TBI, especially when used in higher doses, can contribute to the risk of VOD/SOS.  3. Graft-versus-host disease (GvHD) prophylaxis: The use of certain immunosuppressive medications used for GvHD prophylaxis, such as sirolimus or cyclosporine, may increase the risk of VOD/SOS.  4. Donor type: Unrelated or mismatched donors, or umbilical cord blood transplants may be associated with a higher risk of VOD/SOS.  5. Previous liver disease or damage: Pre-existing liver disease or damage from viral hepatitis, alcohol, or prior chemotherapy or radiation can increase the risk.  6. Age: Older age is generally associated with a higher risk of VOD/SOS.  7. Use of certain medications: Certain medications, including gemtuzumab ozogamicin, can increase the risk of VOD/SOS.  It's important to note that while these factors can increase the risk of VOD/SOS, the presence of one or more risk factors doesn't necessarily mean that a patient will develop the condition. Preventive strategies, careful monitoring, and early intervention can help to manage this risk in patients undergoing HSCT. | The symptoms described in your question suggest that the patient is experiencing Hepatic Veno-Occlusive Disease (VOD), also known as sinusoidal obstruction syndrome (SOS). VOD/SOS is a potential complication of hematopoietic stem cell transplantation (HSCT), particularly allogeneic HSCT, and is characterized by blockage of the small vessels in the liver.  Several transplant-related factors have been identified that may increase the risk of developing VOD/SOS, including:  1. Conditioning regimen: High-intensity or myeloablative conditioning regimens, particularly those containing high-dose busulfan or total body irradiation, are associated with a higher risk of VOD/SOS.  2. Donor type: Allogeneic transplants, particularly from unrelated or mismatched donors, carry a higher risk of VOD/SOS compared to autologous transplants or transplants from matched sibling donors.  3. Graft-versus-host disease (GVHD) prophylaxis: Use of certain immunosuppressive medications like cyclosporine or tacrolimus, particularly in combination with methotrexate, has been linked with a higher risk of VOD/SOS.  4. Previous liver disease or liver damage: Patients with pre-existing liver conditions, or those who have received previous treatment that could have damaged the liver (such as certain chemotherapies or radiotherapy), may be at increased risk.  5. Age: Older patients may be at a higher risk, although VOD/SOS can occur at any age.  6. Infections: Certain types of infections, including fungal infections, have been associated with an increased risk of VOD/SOS.  Understanding these risk factors can help guide preventative strategies and early intervention, potentially reducing the risk of serious complications from VOD/SOS. However, not all cases of VOD/SOS can be prevented, and it's important to monitor patients closely after HSCT for signs and symptoms of this and other potential complications. Treatment typically involves supportive care and potentially specific medications like defibrotide, which is approved for severe VOD/SOS. |
| C18 | Donor lymphocyte infusion (DLI) is a form of adoptive immunotherapy that is sometimes used after allogeneic hematopoietic stem cell transplantation (HSCT). It involves infusing the patient with lymphocytes from the original stem cell donor. This can help to boost the patient's immune system, potentially enhancing the graft-versus-tumor effect and helping to manage or prevent disease relapse.  The dosing of DLI depends on several factors, including:  1. **Indication for DLI:** The dose may be different depending on why the DLI is being given. For example, lower doses might be used for preventive purposes or to treat minimal residual disease, while higher doses might be used for treating active relapse.  2. **Patient's overall health and tolerance:** The dose might need to be adjusted depending on the patient's overall health status, including their tolerance to potential side effects and complications.  3. **Risk of Graft-versus-Host Disease (GVHD):** GVHD is a potential complication of DLI. Patients at higher risk of GVHD may receive lower doses to help minimize this risk.  4. **Response to previous treatments:** If the patient has already received a DLI and not responded, or if the disease has progressed, a higher dose might be considered.  In many cases, DLI is administered using a stepwise approach, where the dose is gradually escalated over time. This allows for monitoring of the patient's response and side effects, and allows for dose adjustments as needed.  Always remember, this is a complex medical procedure and the exact protocol, including the dosing regimen, will be personalized for each patient based on their specific situation and the expert judgment of their medical team. This information should not be used as a substitute for professional medical advice. Always consult with a healthcare provider. | Donor lymphocyte infusion (DLI) is a therapy that involves infusing lymphocytes from the original stem cell donor into the recipient, with the aim of boosting the graft-versus-tumor (GVT) effect to treat or prevent relapse of the original disease after hematopoietic stem cell transplantation (HSCT).  The dosing of DLI can depend on several factors:  1. **Patient Factors**: The patient's overall health, age, and organ function can influence the dosing of DLI. Patients who are frailer or who have poorer organ function may need to start with a lower dose to minimize potential complications.  2. **Disease Factors**: The stage and aggressiveness of the disease can also influence the dosing. More aggressive diseases may require a higher initial dose to achieve a sufficient GVT effect.  3. **Graft-versus-Host Disease (GVHD)**: GVHD is a major potential complication of DLI. The risk of GVHD can be reduced by starting with a lower dose of DLI and gradually escalating as tolerated, also known as a dose-escalation strategy.  4. **Previous Response to HSCT and DLI**: If the patient has received previous DLI or had response to HSCT, this could impact the dose selection. Patients who have had severe GVHD may need to start with a lower dose, whereas patients who have not had a strong GVT response may need a higher dose.  5. **Donor Factors**: The number of available lymphocytes from the donor can also limit the dosing of DLI.  6. **Immunosuppressive Therapy**: The use and timing of concurrent immunosuppressive therapy can impact the dose and timing of DLI.  The exact dosing and schedule for DLI can vary widely based on these and other factors, and there is no one-size-fits-all approach. The treating physician will determine the most appropriate dosing strategy based on the individual patient's circumstances. This information should not be used as a substitute for professional medical advice. Always consult with a healthcare provider. | Donor lymphocyte infusion (DLI) is a treatment used in the context of allogeneic hematopoietic stem cell transplantation (HSCT). It involves the infusion of lymphocytes from the original stem cell donor, which can help to boost the patient's immune system and enhance the graft-versus-tumor effect.  The dosing of DLI can depend on several factors, including:  1. **Patient's Disease Status**: The patient's disease type, stage, and the reason for giving the DLI (for instance, whether it's being used to treat disease relapse, for prophylaxis against relapse, or to enhance immune recovery) can all influence the dose of DLI.  2. **Patient's Health and Tolerance**: The patient's overall health status, including their level of immune suppression, their organ function, and their ability to tolerate potential side effects, can also impact the dose.  3. **Presence of Graft-versus-Host Disease (GVHD)**: The occurrence of GVHD, a potential complication of HSCT and DLI where donor cells attack the patient's tissues, can influence the dosing decisions. Doses may need to be adjusted, or DLI may need to be postponed, in patients who have active GVHD.  4. **Donor Factors**: The availability of donor lymphocytes, the quality of the donor cells, and the match quality between the donor and recipient can also impact the dose.  The exact dosing of DLI can vary widely from patient to patient, and the best approach often needs to be individualized based on these and possibly other factors. Monitoring of the patient's response and adjustment of the DLI dose over time is also often needed.  Remember, always consult with a healthcare provider for appropriate management. This information should not be used as a substitute for professional medical advice. |

| **Google Bard** | | | |
| --- | --- | --- | --- |
| **Section 1 – Drug: mechanism of action, toxicity, and interaction** | | | |
| D1 | Cyclosporine is a powerful immunosuppressant drug that is used to prevent the rejection of transplanted organs, treat severe psoriasis, and atopic dermatitis. It can also be used to treat rheumatoid arthritis and other autoimmune diseases.  Common side effects of cyclosporine include:   - High blood pressure - Increased hair growth - Swollen or inflamed gums - Numbness or tingling of the hands or feet - Tremors, restlessness, stomach upset, nausea, cramps, diarrhea, headache, and changes in blood sugar   More serious side effects of cyclosporine can include:   - Kidney problems - Increased risk of infections - Neurotoxicity (e.g., convulsions, encephalopathy, anxiety, headache, and fever) - Increased risk of skin and lymphoproliferative malignancies   If you are taking cyclosporine, it is important to be aware of the potential side effects and to monitor your blood pressure and kidney function regularly. You should also see your doctor immediately if you develop any of the more serious side effects.  Here are some tips to help reduce your risk of side effects from cyclosporine:   - Take the medication exactly as prescribed by your doctor. - Have your blood pressure and kidney function checked regularly. - Avoid taking other medications that can interact with cyclosporine. - Report any side effects to your doctor immediately.   If you are concerned about the side effects of cyclosporine, talk to your doctor. They can help you weigh the risks and benefits of the medication and decide if it is right for you. | Cyclosporine is a powerful immunosuppressant drug that is used to prevent the rejection of transplanted organs, treat severe psoriasis, and atopic dermatitis. It can also be used to treat rheumatoid arthritis and other autoimmune diseases.  Common side effects of cyclosporine include:   - **High blood pressure:** Cyclosporine can cause your blood pressure to rise. Your doctor will monitor your blood pressure closely while you are taking cyclosporine. - **Increased hair growth:** Cyclosporine can cause you to grow more hair, especially on your face and back. - **Swollen or inflamed gums:** Cyclosporine can cause your gums to become swollen or inflamed. This is called gingivitis. - **Numbness or tingling of the hands or feet:** Cyclosporine can cause nerve damage that can lead to numbness or tingling in your hands or feet. - **Tremors, restlessness, stomach upset, nausea, cramps, diarrhea, headache, and changes in blood sugar:** These are also common side effects of cyclosporine.   More serious side effects of cyclosporine can include:   - **Kidney problems:** Cyclosporine can damage your kidneys. Your doctor will monitor your kidney function closely while you are taking cyclosporine. - **Increased risk of infections:** Cyclosporine can increase your risk of getting infections. You should be careful to avoid getting sick while you are taking cyclosporine. - **Neurotoxicity:** Cyclosporine can damage your nerves. This can lead to convulsions, encephalopathy, anxiety, headache, and fever. - **Increased risk of skin and lymphoproliferative malignancies:** Cyclosporine can increase your risk of developing skin cancer and other types of cancer.   If you are taking cyclosporine, it is important to be aware of the potential side effects and to monitor your blood pressure and kidney function regularly. You should also see your doctor immediately if you develop any of the more serious side effects.  Here are some tips to help reduce your risk of side effects from cyclosporine:   - Take the medication exactly as prescribed by your doctor. - Have your blood pressure and kidney function checked regularly. - Avoid taking other medications that can interact with cyclosporine. - Report any side effects to your doctor immediately.   If you are concerned about the side effects of cyclosporine, talk to your doctor. They can help you weigh the risks and benefits of the medication and decide if it is right for you. | Cyclosporine is a powerful immunosuppressant drug that is used to prevent the rejection of transplanted organs, treat severe psoriasis, and atopic dermatitis. It can also be used to treat rheumatoid arthritis and other autoimmune diseases.  **Common side effects of cyclosporine include:**   - **High blood pressure:** Cyclosporine can cause your blood pressure to rise. Your doctor will monitor your blood pressure closely while you are taking cyclosporine. - **Increased hair growth:** Cyclosporine can cause you to grow more hair, especially on your face and back. - **Swollen or inflamed gums:** Cyclosporine can cause your gums to become swollen or inflamed. This is called gingivitis. - **Numbness or tingling of the hands or feet:** Cyclosporine can cause nerve damage that can lead to numbness or tingling in your hands or feet. - **Tremors, restlessness, stomach upset, nausea, cramps, diarrhea, headache, and changes in blood sugar:** These are also common side effects of cyclosporine.   **More serious side effects of cyclosporine can include:**   - **Kidney problems:** Cyclosporine can damage your kidneys. Your doctor will monitor your kidney function closely while you are taking cyclosporine. - **Increased risk of infections:** Cyclosporine can increase your risk of getting infections. You should be careful to avoid getting sick while you are taking cyclosporine. - **Neurotoxicity:** Cyclosporine can damage your nerves. This can lead to convulsions, encephalopathy, anxiety, headache, and fever. - **Increased risk of skin and lymphoproliferative malignancies:** Cyclosporine can increase your risk of developing skin cancer and other types of cancer.   If you are taking cyclosporine, it is important to be aware of the potential side effects and to monitor your blood pressure and kidney function regularly. You should also see your doctor immediately if you develop any of the more serious side effects.  Here are some tips to help reduce your risk of side effects from cyclosporine:   - Take the medication exactly as prescribed by your doctor. - Have your blood pressure and kidney function checked regularly. - Avoid taking other medications that can interact with cyclosporine. - Report any side effects to your doctor immediately.   If you are concerned about the side effects of cyclosporine, talk to your doctor. They can help you weigh the risks and benefits of the medication and decide if it is right for you.  I hope this helps! |
| D2 | Sirolimus is a drug that targets the mechanistic target of rapamycin (mTOR), a protein that regulates cell growth and proliferation. Sirolimus binds to FKBP12, another protein, and this complex inhibits mTOR. This inhibition of mTOR leads to a number of effects, including:  * Blockage of cell-cycle progression at the G1/S phase  * Inhibition of protein synthesis  * Inhibition of angiogenesis (the growth of new blood vessels)  Sirolimus is used as an immunosuppressant to prevent organ rejection after transplantation. It is also used to treat a number of other conditions, including:  * Tuberous sclerosis  * Cancer (skin cancer, Kaposi's sarcoma, and cutaneous T-cell lymphomas)  * Angiofibroma (a type of facial tumor)  Sirolimus is a relatively safe drug, but it can have side effects, including:  * Increased risk of infections  * High blood pressure  * Kidney problems  * Diabetes  * Hair loss  If you are taking sirolimus, it is important to be aware of the potential side effects and to monitor your health closely. | The target of sirolimus is the mammalian target of rapamycin (mTOR). mTOR is a protein kinase that regulates cell growth and proliferation. Sirolimus binds to FKBP12, another protein, and this complex inhibits mTOR. This inhibition of mTOR leads to a number of effects, including:  * Blockage of cell-cycle progression at the G1/S phase  * Inhibition of protein synthesis  * Inhibition of angiogenesis (the growth of new blood vessels)  Sirolimus is used as an immunosuppressant to prevent organ rejection after transplantation. It is also used to treat a number of other conditions, including:  * Tuberous sclerosis  * Cancer (skin cancer, Kaposi's sarcoma, and cutaneous T-cell lymphomas)  * Angiofibroma (a type of facial tumor)  The mTOR pathway is also involved in a number of other cellular processes, including:  * Metabolism  * Growth  * Cell survival  * Autophagy  Sirolimus is a relatively safe drug, but it can have side effects, including:  * Increased risk of infections  * High blood pressure  * Kidney problems  * Diabetes  * Hair loss  If you are taking sirolimus, it is important to be aware of the potential side effects and to monitor your health closely. | Sirolimus targets the mechanistic target of rapamycin (mTOR), a protein kinase that regulates cell growth and proliferation. mTOR is a key regulator of cell growth and metabolism, and its inhibition by sirolimus leads to a number of effects, including:  * Blockage of cell-cycle progression at the G1/S phase  * Inhibition of protein synthesis  * Inhibition of angiogenesis (the growth of new blood vessels)  Sirolimus is used as an immunosuppressant to prevent organ rejection after transplantation. It is also used to treat a number of other conditions, including:  * Tuberous sclerosis  * Cancer (skin cancer, Kaposi's sarcoma, and cutaneous T-cell lymphomas)  * Angiofibroma (a type of facial tumor)  The mTOR pathway is also involved in a number of other cellular processes, including:  * Metabolism  * Growth  * Cell survival  * Autophagy  Sirolimus is a relatively safe drug, but it can have side effects, including:  * Increased risk of infections  * High blood pressure  * Kidney problems  * Diabetes  * Hair loss  If you are taking sirolimus, it is important to be aware of the potential side effects and to monitor your health closely.  To answer your question more specifically, the target of sirolimus is the mTOR complex 1 (mTORC1), which is a protein complex that regulates cell growth and proliferation. Sirolimus binds to FKBP12, another protein, and this complex inhibits mTORC1. This inhibition of mTORC1 leads to the effects described above. |
| D3 | I'm a text-based AI, and that is outside of my capabilities. | Voriconazole is known to increase the serum level of cyclosporine, a systemic immunosuppressant. This is because both drugs are metabolized by the same enzyme, CYP3A4. When voriconazole is present, it competes with cyclosporine for this enzyme, which can lead to increased levels of cyclosporine in the blood. This can increase the risk of cyclosporine-related side effects, such as nephrotoxicity and hepatotoxicity.  Other systemic immunosuppressants that may be affected by voriconazole include tacrolimus, sirolimus, and everolimus. However, the interaction with cyclosporine is the most well-known and clinically significant.  If you are taking voriconazole and any of these systemic immunosuppressants, it is important to monitor your blood levels closely. Your doctor may need to adjust your dose of cyclosporine or the other immunosuppressant to prevent toxicity.  Here are some additional resources that you may find helpful:   - Voriconazole Drug Interactions: https://www.drugs.com/drug-interactions/voriconazole.html - Cyclosporine Drug Interactions: https://www.drugs.com/drug-interactions/cyclosporine.html - Therapeutic Drug Monitoring of Voriconazole: https://www.ncbi.nlm.nih.gov/pmc/articles/PMC3352069/ | Voriconazole is known to increase the serum level of **cyclosporine**, a systemic immunosuppressant. This is because both drugs are metabolized by the same enzyme, CYP3A4. When voriconazole is present, it competes with cyclosporine for this enzyme, which can lead to increased levels of cyclosporine in the blood. This can increase the risk of cyclosporine-related side effects, such as nephrotoxicity and hepatotoxicity.  Other systemic immunosuppressants that may be affected by voriconazole include tacrolimus, sirolimus, and everolimus. However, the interaction with cyclosporine is the most well-known and clinically significant.  If you are taking voriconazole and any of these systemic immunosuppressants, it is important to monitor your blood levels closely. Your doctor may need to adjust your dose of cyclosporine or the other immunosuppressant to prevent toxicity.  I hope this helps! Let me know if you have other questions. |
| D4 | The most common drugs combined with calcineurin inhibitors (CNIs) as a graft-versus-host disease (GVHD) prophylaxis regimen are methotrexate (MTX) and steroids.  * **Methotrexate** is a type of chemotherapy drug that works by suppressing the immune system. It is typically given as a loading dose followed by maintenance doses.  * **Steroids** are a type of anti-inflammatory drug that also suppresses the immune system. They are typically given as a short course of high-dose steroids followed by a longer course of lower-dose steroids.  Other drugs that may be used in combination with CNIs for GVHD prophylaxis include:  * **Sirolimus** is a type of mTOR inhibitor that works by suppressing the growth of T cells.  * **Belatacept** is a type of costimulation blocker that works by blocking the interaction between T cells and antigen-presenting cells.  * **Eculizumab** is a type of complement inhibitor that works by blocking the complement cascade, which is a part of the immune system that can contribute to GVHD.  The choice of drugs for GVHD prophylaxis depends on a number of factors, including the type of transplant, the patient's age and health status, and the patient's individual risk of GVHD.  Here is a table summarizing the different drugs that may be used in combination with CNIs for GVHD prophylaxis:  \| Drug \| Mechanism of action \| Side effects \|  \|---\|---\|---\|  \| Methotrexate \| Suppresses the immune system \| Bone marrow suppression, liver toxicity, mouth ulcers \|  \| Steroids \| Suppresses the immune system \| Weight gain, mood swings, high blood pressure, diabetes \|  \| Sirolimus \| Suppresses the growth of T cells \| Kidney problems, high blood pressure, infections \|  \| Belatacept \| Blocks the interaction between T cells and antigen-presenting cells \| Kidney problems, infections \|  \| Eculizumab \| Blocks the complement cascade \| High blood pressure, infections \|  It is important to note that all of these drugs have side effects, and the risks and benefits of each drug should be carefully considered before starting treatment. | The most common drugs combined with calcineurin inhibitors (CNIs) as a graft-versus-host disease (GVHD) prophylaxis regimen are methotrexate (MTX) and steroids.  * **Methotrexate** is a type of chemotherapy drug that works by suppressing the immune system. It is typically given as a loading dose followed by maintenance doses.  * **Steroids** are a type of anti-inflammatory drug that also suppresses the immune system. They are typically given as a short course of high-dose steroids followed by a longer course of lower-dose steroids.  Other drugs that may be used in combination with CNIs for GVHD prophylaxis include:  * **Sirolimus** is a type of mTOR inhibitor that works by suppressing the growth of T cells.  * **Belatacept** is a type of costimulation blocker that works by blocking the interaction between T cells and antigen-presenting cells.  * **Eculizumab** is a type of complement inhibitor that works by blocking the complement cascade, which is a part of the immune system that can contribute to GVHD.  The choice of drugs for GVHD prophylaxis depends on a number of factors, including the type of transplant, the patient's age and health status, and the patient's individual risk of GVHD.  Here is a table summarizing the different drugs that may be used in combination with CNIs for GVHD prophylaxis:  \| Drug \| Mechanism of action \| Side effects \|  \|---\|---\|---\|  \| Methotrexate \| Suppresses the immune system \| Bone marrow suppression, liver toxicity, mouth ulcers \|  \| Steroids \| Suppresses the immune system \| Weight gain, mood swings, high blood pressure, diabetes \|  \| Sirolimus \| Suppresses the growth of T cells \| Kidney problems, high blood pressure, infections \|  \| Belatacept \| Blocks the interaction between T cells and antigen-presenting cells \| Kidney problems, infections \|  \| Eculizumab \| Blocks the complement cascade \| High blood pressure, infections \|  It is important to note that all of these drugs have side effects, and the risks and benefits of each drug should be carefully considered before starting treatment.  Here are some additional details about the drugs that are most often combined with CNIs for GVHD prophylaxis:  * **Methotrexate** is a relatively old drug that has been used for GVHD prophylaxis for many years. It is effective in preventing GVHD, but it can also have side effects, such as bone marrow suppression and liver toxicity.  * **Steroids** are also relatively old drugs that have been used for GVHD prophylaxis for many years. They are effective in preventing GVHD, but they can also have side effects, such as weight gain, mood swings, high blood pressure, and diabetes.  * **Sirolimus** is a newer drug that has been shown to be effective in preventing GVHD. It is not as well-established as methotrexate or steroids, but it may have fewer side effects.  * **Belatacept** is a newer drug that is still being studied for GVHD prophylaxis. It is not as well-established as methotrexate, steroids, or sirolimus, but it may have fewer side effects.  * **Eculizumab** is a newer drug that is still being studied for GVHD prophylaxis. It is not as well-established as methotrexate, steroids, sirolimus, or belatacept, but it may be effective in preventing chronic GVHD.  The best drug for GVHD prophylaxis depends on the individual patient and their specific circumstances. The doctor will consider the patient's age, health status, and risk of GVHD when making a decision about which drugs to use. | The most common drugs combined with calcineurin inhibitors (CNIs) as a graft-versus-host disease (GVHD) prophylaxis regimen are **methotrexate** (MTX) and **steroids**.  * **Methotrexate** is a type of chemotherapy drug that works by suppressing the immune system. It is typically given as a loading dose followed by maintenance doses.  * **Steroids** are a type of anti-inflammatory drug that also suppresses the immune system. They are typically given as a short course of high-dose steroids followed by a longer course of lower-dose steroids.  Other drugs that may be used in combination with CNIs for GVHD prophylaxis include:  * **Sirolimus** is a type of mTOR inhibitor that works by suppressing the growth of T cells.  * **Belatacept** is a type of costimulation blocker that works by blocking the interaction between T cells and antigen-presenting cells.  * **Eculizumab** is a type of complement inhibitor that works by blocking the complement cascade, which is a part of the immune system that can contribute to GVHD.  The choice of drugs for GVHD prophylaxis depends on a number of factors, including the type of transplant, the patient's age and health status, and the patient's individual risk of GVHD.  Here is a table summarizing the different drugs that may be used in combination with CNIs for GVHD prophylaxis:  \| Drug \| Mechanism of action \| Side effects \|  \|---\|---\|---\|  \| Methotrexate \| Suppresses the immune system \| Bone marrow suppression, liver toxicity, mouth ulcers \|  \| Steroids \| Suppresses the immune system \| Weight gain, mood swings, high blood pressure, diabetes \|  \| Sirolimus \| Suppresses the growth of T cells \| Kidney problems, high blood pressure, infections \|  \| Belatacept \| Blocks the interaction between T cells and antigen-presenting cells \| Kidney problems, infections \|  \| Eculizumab \| Blocks the complement cascade \| High blood pressure, infections \|  It is important to note that all of these drugs have side effects, and the risks and benefits of each drug should be carefully considered before starting treatment.  Here are some additional details about the drugs that are most often combined with CNIs for GVHD prophylaxis:  * **Methotrexate** is a relatively old drug that has been used for GVHD prophylaxis for many years. It is effective in preventing GVHD, but it can also have side effects, such as bone marrow suppression and liver toxicity.  * **Steroids** are also relatively old drugs that have been used for GVHD prophylaxis for many years. They are effective in preventing GVHD, but they can also have side effects, such as weight gain, mood swings, high blood pressure, and diabetes.  * **Sirolimus** is a newer drug that has been shown to be effective in preventing GVHD. It is not as well-established as methotrexate or steroids, but it may have fewer side effects.  * **Belatacept** is a newer drug that is still being studied for GVHD prophylaxis. It is not as well-established as methotrexate, steroids, or sirolimus, but it may have fewer side effects.  * **Eculizumab** is a newer drug that is still being studied for GVHD prophylaxis. It is not as well-established as methotrexate, steroids, sirolimus, or belatacept, but it may be effective in preventing chronic GVHD.  The best drug for GVHD prophylaxis depends on the individual patient and their specific circumstances. The doctor will consider the patient's age, health status, and risk of GVHD when making a decision about which drugs to use.  I hope this helps! Let me know if you have any other questions. |
| D5 | Busulfan is metabolized primarily in the liver by conjugation with glutathione, both spontaneously and by glutathione S-transferase (GST) catalysis. GSTA1 is the primary GST isoform that facilitates the the metabolism of busulfan.  The biochemical pathway for busulfan metabolism is as follows:  1. Busulfan is taken up by the liver cells.  2. Busulfan is conjugated with glutathione, a molecule that helps to detoxify harmful substances.  3. The conjugated busulfan is then excreted in the urine.  The organ site for busulfan metabolism is the liver. This is because the liver is the main organ that is responsible for detoxifying harmful substances.  Busulfan is a cytotoxic drug that is used to treat a variety of conditions, including chronic myelogenous leukemia (CML) and myelodysplastic syndromes (MDS). It works by killing cells in the bone marrow.  The metabolism of busulfan is important because it helps to reduce the toxicity of the drug. The conjugated form of busulfan is less toxic than the unconjugated form.  There are a number of factors that can affect the metabolism of busulfan, including the patient's age, health status, and genetic makeup. Patients with liver disease may have slower metabolism of busulfan, which can increase the risk of toxicity.  It is important to monitor the levels of busulfan in the blood to ensure that the drug is being metabolized properly. If the levels are too high, the patient may experience toxicity.  I hope this helps! Let me know if you have any other questions. | Busulfan is a cytotoxic drug that is used to treat a variety of conditions, including chronic myelogenous leukemia (CML) and myelodysplastic syndromes (MDS). It works by killing cells in the bone marrow.  Busulfan is metabolized primarily in the liver by conjugation with glutathione, both spontaneously and by glutathione S-transferase (GST) catalysis. GSTA1 is the primary GST isoform that facilitates the the metabolism of busulfan.  The biochemical pathway for busulfan metabolism is as follows:  1. Busulfan is taken up by the liver cells.  2. Busulfan is conjugated with glutathione, a molecule that helps to detoxify harmful substances.  3. The conjugated busulfan is then excreted in the urine.  The organ site for busulfan metabolism is the liver. This is because the liver is the main organ that is responsible for detoxifying harmful substances.  The metabolism of busulfan is important because it helps to reduce the toxicity of the drug. The conjugated form of busulfan is less toxic than the unconjugated form.  There are a number of factors that can affect the metabolism of busulfan, including the patient's age, health status, and genetic makeup. Patients with liver disease may have slower metabolism of busulfan, which can increase the risk of toxicity.  It is important to monitor the levels of busulfan in the blood to ensure that the drug is being metabolized properly. If the levels are too high, the patient may experience toxicity.  Here are some additional details about the biochemical pathway of busulfan metabolism:  * **Glutathione** is a molecule that is made up of three amino acids: cysteine, glycine, and glutamic acid. It is a powerful antioxidant that helps to protect cells from damage.  * **Glutathione S-transferase** (GST) is an enzyme that helps to conjugate glutathione with other molecules. This process makes the molecules less toxic and easier to excrete from the body.  * **GSTA1** is the primary GST isoform that facilitates the the metabolism of busulfan. This means that it is the most important enzyme involved in the conjugation of busulfan with glutathione.  I hope this helps! Let me know if you have any other questions. | The biochemical pathway and organ site for busulfan metabolism are as follows:  * **Biochemical pathway:**  * Busulfan is taken up by the liver cells.  * Busulfan is conjugated with glutathione, a molecule that helps to detoxify harmful substances.  * The conjugated busulfan is then excreted in the urine.  * **Organ site:**  * The liver is the main organ responsible for busulfan metabolism.  Busulfan is a cytotoxic drug that is used to treat a variety of conditions, including chronic myelogenous leukemia (CML) and myelodysplastic syndromes (MDS). It works by killing cells in the bone marrow.  The metabolism of busulfan is important because it helps to reduce the toxicity of the drug. The conjugated form of busulfan is less toxic than the unconjugated form.  There are a number of factors that can affect the metabolism of busulfan, including the patient's age, health status, and genetic makeup. Patients with liver disease may have slower metabolism of busulfan, which can increase the risk of toxicity.  It is important to monitor the levels of busulfan in the blood to ensure that the drug is being metabolized properly. If the levels are too high, the patient may experience toxicity.  Here are some additional details about the biochemical pathway of busulfan metabolism:  * **Glutathione** is a molecule that is made up of three amino acids: cysteine, glycine, and glutamic acid. It is a powerful antioxidant that helps to protect cells from damage.  * **Glutathione S-transferase** (GST) is an enzyme that helps to conjugate glutathione with other molecules. This process makes the molecules less toxic and easier to excrete from the body.  * **GSTA1** is the primary GST isoform that facilitates the the metabolism of busulfan. This means that it is the most important enzyme involved in the conjugation of busulfan with glutathione.  I hope this helps! Let me know if you have any other questions. |
| D6 | Patients undergoing HSCT are at high risk for developing Pneumocystis jirovecii pneumonia (PCP), so they are typically given trimethoprim/sulfamethoxazole (TMP/SMX) prophylaxis. The recommended frequency of TMP/SMX administration for PCP prophylaxis in patients undergoing HSCT is **twice weekly**. This is in contrast to the standard once-daily dosing for other populations at risk for PCP, such as HIV-positive individuals.  The reason for the twice-weekly dosing in HSCT patients is that their immune systems are often severely compromised, and they may not be able to tolerate the full daily dose of TMP/SMX. Twice-weekly dosing is still effective at preventing PCP, while reducing the risk of side effects.  The specific dose of TMP/SMX that is given twice weekly will vary depending on the patient's age, weight, and other medical conditions. However, the typical dose is one double-strength tablet (160 mg/800 mg) of TMP/SMX twice weekly.  It is important to note that TMP/SMX is not the only option for PCP prophylaxis in HSCT patients. Other agents that may be used include dapsone, pentamidine, and atovaquone. The choice of agent will depend on the patient's individual risk factors and preferences.  I hope this helps! Let me know if you have any other questions. | The recommended frequency of TMP/SMX administration for PCP prophylaxis in patients undergoing HSCT is **twice weekly**. This is in contrast to the standard once-daily dosing for other populations at risk for PCP, such as HIV-positive individuals.  The reason for the twice-weekly dosing in HSCT patients is that their immune systems are often severely compromised, and they may not be able to tolerate the full daily dose of TMP/SMX. Twice-weekly dosing is still effective at preventing PCP, while reducing the risk of side effects.  The specific dose of TMP/SMX that is given twice weekly will vary depending on the patient's age, weight, and other medical conditions. However, the typical dose is one double-strength tablet (160 mg/800 mg) of TMP/SMX twice weekly.  It is important to note that TMP/SMX is not the only option for PCP prophylaxis in HSCT patients. Other agents that may be used include dapsone, pentamidine, and atovaquone. The choice of agent will depend on the patient's individual risk factors and preferences.  I hope this helps! Let me know if you have any other questions. | The recommended frequency of TMP/SMX administration for PCP prophylaxis in patients undergoing HSCT is **twice weekly**. This is in contrast to the standard once-daily dosing for other populations at risk for PCP, such as HIV-positive individuals.  The reason for the twice-weekly dosing in HSCT patients is that their immune systems are often severely compromised, and they may not be able to tolerate the full daily dose of TMP/SMX. Twice-weekly dosing is still effective at preventing PCP, while reducing the risk of side effects.  The specific dose of TMP/SMX that is given twice weekly will vary depending on the patient's age, weight, and other medical conditions. However, the typical dose is one double-strength tablet (160 mg/800 mg) of TMP/SMX twice weekly.  I hope this answers your question! Let me know if you have any other questions. |
| D7 | The drug you are referring to is **ruxolitinib**, which was approved by the FDA in 2014 for the treatment of refractory acute graft-versus-host disease (GVHD). Ruxolitinib is a JAK inhibitor, which means that it works by blocking the activity of a group of enzymes called JAKs. JAKs are involved in the signaling pathways that control the growth and function of immune cells. By blocking JAKs, ruxolitinib can help to suppress the immune response that is responsible for GVHD.  In addition to GVHD, ruxolitinib is also commonly used to treat **polycythemia vera** (PV) and **essential thrombocythemia** (ET). PV is a blood cancer that is characterized by an overproduction of red blood cells. ET is a blood cancer that is characterized by an overproduction of platelets. Ruxolitinib can help to reduce the number of red blood cells and platelets in people with PV and ET, which can help to improve their symptoms and reduce their risk of complications.  Here are some other settings where ruxolitinib is being studied:   - **Chronic GVHD:** Ruxolitinib is currently being studied in clinical trials for the treatment of chronic GVHD. - **Myelofibrosis:** Ruxolitinib is also being studied in clinical trials for the treatment of myelofibrosis, a blood cancer that is characterized by the growth of abnormal bone marrow cells. - **Acute myeloid leukemia:** Ruxolitinib is also being studied in clinical trials for the treatment of acute myeloid leukemia, a type of blood cancer that is characterized by the rapid growth of abnormal white blood cells.   I hope this helps! Let me know if you have other questions. | The drug you are referring to is **ruxolitinib**, which was approved by the FDA in 2014 for the treatment of refractory acute graft-versus-host disease (GVHD). Ruxolitinib is a JAK inhibitor, which means that it works by blocking the activity of a group of enzymes called JAKs. JAKs are involved in the signaling pathways that control the growth and function of immune cells. By blocking JAKs, ruxolitinib can help to suppress the immune response that is responsible for GVHD.  In addition to GVHD, ruxolitinib is also commonly used to treat polycythemia vera (PV) and essential thrombocythemia (ET). PV is a blood cancer that is characterized by an overproduction of red blood cells. ET is a blood cancer that is characterized by an overproduction of platelets. Ruxolitinib can help to reduce the number of red blood cells and platelets in people with PV and ET, which can help to improve their symptoms and reduce their risk of complications.  Here are some other settings where ruxolitinib is being studied:   - **Chronic GVHD:** Ruxolitinib is currently being studied in clinical trials for the treatment of chronic GVHD. - **Myelofibrosis:** Ruxolitinib is also being studied in clinical trials for the treatment of myelofibrosis, a blood cancer that is characterized by the growth of abnormal bone marrow cells. - **Acute myeloid leukemia:** Ruxolitinib is also being studied in clinical trials for the treatment of acute myeloid leukemia, a type of blood cancer that is characterized by the rapid growth of abnormal white blood cells.   However, the drug you are referring to is most commonly used to treat refractory acute GVHD. This is a serious condition that can occur after a bone marrow transplant. It occurs when the donor's immune cells attack the recipient's body. Ruxolitinib can help to suppress the immune response and improve the symptoms of GVHD.  I hope this helps! Let me know if you have other questions. | The drug you are referring to is **ruxolitinib**. It is a JAK inhibitor that was approved by the FDA in 2014 for the treatment of refractory acute graft-versus-host disease (GVHD). Ruxolitinib is also commonly used to treat polycythemia vera (PV) and essential thrombocythemia (ET).  Here are some other settings where ruxolitinib is being studied:   - **Chronic GVHD:** Ruxolitinib is currently being studied in clinical trials for the treatment of chronic GVHD. - **Myelofibrosis:** Ruxolitinib is also being studied in clinical trials for the treatment of myelofibrosis, a blood cancer that is characterized by the growth of abnormal bone marrow cells. - **Acute myeloid leukemia:** Ruxolitinib is also being studied in clinical trials for the treatment of acute myeloid leukemia, a type of blood cancer that is characterized by the rapid growth of abnormal white blood cells.   However, the drug you are referring to is most commonly used to treat refractory acute GVHD. This is a serious condition that can occur after a bone marrow transplant. It occurs when the donor's immune cells attack the recipient's body. Ruxolitinib can help to suppress the immune response and improve the symptoms of GVHD.  So the answer to your question is that ruxolitinib is also commonly used to treat polycythemia vera (PV) and essential thrombocythemia (ET). |
| D8 | Busulfan is a chemotherapy drug that is commonly used during conditioning for allogeneic HSCT. It is an alkylating agent, which means that it works by damaging DNA and preventing cells from dividing. Busulfan can cause a number of side effects, including lung injury.  The lung injury caused by busulfan is called **busulfan-induced pneumonitis**. It is a rare but serious condition that can occur up to several months after busulfan treatment. The symptoms of busulfan-induced pneumonitis include dry cough, shortness of breath, and chest pain.  If a patient develops dry cough after receiving allogeneic HSCT, it is important to rule out infectious causes. If infectious causes are excluded, then busulfan-induced pneumonitis is a possibility. The diagnosis of busulfan-induced pneumonitis is confirmed by a lung biopsy.  There is no specific treatment for busulfan-induced pneumonitis. Treatment is supportive and may include oxygen therapy, steroids, and other medications. The prognosis for busulfan-induced pneumonitis is variable. Some patients recover completely, while others may have persistent lung damage.  Here are some other drugs that can cause lung injury during conditioning for allogeneic HSCT:   - **Cyclophosphamide** - **Melphalan** - **Total body irradiation**   If you are concerned about the risk of lung injury from busulfan or other drugs used during conditioning for allogeneic HSCT, talk to your doctor. They can help you understand the risks and benefits of these drugs and make the best decision for your care. | Busulfan is a chemotherapy drug that is commonly used during conditioning for allogeneic HSCT. It is an alkylating agent, which means that it works by damaging DNA and preventing cells from dividing. Busulfan can cause a number of side effects, including lung injury.  The lung injury caused by busulfan is called **busulfan-induced pneumonitis**. It is a rare but serious condition that can occur up to several months after busulfan treatment. The symptoms of busulfan-induced pneumonitis include dry cough, shortness of breath, and chest pain.  If a patient develops dry cough after receiving allogeneic HSCT, it is important to rule out infectious causes. If infectious causes are excluded, then busulfan-induced pneumonitis is a possibility. The diagnosis of busulfan-induced pneumonitis is confirmed by a lung biopsy.  There is no specific treatment for busulfan-induced pneumonitis. Treatment is supportive and may include oxygen therapy, steroids, and other medications. The prognosis for busulfan-induced pneumonitis is variable. Some patients recover completely, while others may have persistent lung damage.  Here are some other drugs that can cause lung injury during conditioning for allogeneic HSCT:   - **Cyclophosphamide** - **Melphalan** - **Total body irradiation**   If you are concerned about the risk of lung injury from busulfan or other drugs used during conditioning for allogeneic HSCT, talk to your doctor. They can help you understand the risks and benefits of these drugs and make the best decision for your care.  However, it is important to note that there are other drugs that can cause lung injury after allogeneic HSCT. These include:   - **Antithymocyte globulin (ATG)** - **Muromonab-CD3 (OKT3)** - **Tacrolimus** - **Sirolimus**   If a patient develops dry cough after receiving allogeneic HSCT, it is important to talk to their doctor to determine the cause of the cough and the best course of treatment. | There are a few drugs that are commonly used during conditioning for allogeneic HSCT that can be associated with lung injury. These include:   - **Busulfan:** Busulfan is an alkylating agent that can cause lung injury, also known as **busulfan-induced pneumonitis**. This is a rare but serious condition that can occur up to several months after busulfan treatment. The symptoms of busulfan-induced pneumonitis include dry cough, shortness of breath, and chest pain. - **Cyclophosphamide:** Cyclophosphamide is another alkylating agent that can cause lung injury. The symptoms of cyclophosphamide-induced pneumonitis are similar to those of busulfan-induced pneumonitis. - **Total body irradiation (TBI):** TBI is a type of radiation therapy that is used to kill cancer cells before allogeneic HSCT. TBI can also cause lung injury, which is called **radiation pneumonitis**. The symptoms of radiation pneumonitis usually develop within 1-2 months of TBI treatment.   If a patient develops dry cough after receiving allogeneic HSCT, it is important to rule out infectious causes. If infectious causes are excluded, then one of these drugs may be the cause of the lung injury. The diagnosis of lung injury caused by these drugs is confirmed by a lung biopsy.  There is no specific treatment for lung injury caused by these drugs. Treatment is supportive and may include oxygen therapy, steroids, and other medications. The prognosis for lung injury caused by these drugs is variable. Some patients recover completely, while others may have persistent lung damage.  It is important to talk to your doctor if you are concerned about the risk of lung injury from these drugs. They can help you understand the risks and benefits of these drugs and make the best decision for your care. |
| D9 | The patient is likely receiving **liposomal amphotericin B** (AmBisome) for invasive aspergillosis. The standard dose of AmBisome is 3-5 mg/kg/day, given intravenously over 2 hours.  Invasive aspergillosis is a serious fungal infection that can occur in patients who have undergone allogeneic HSCT. It is characterized by fever, cough, and lung infiltrates on CT scan. Galactomannan is a sugar molecule that is produced by Aspergillus fungi, and its presence in the blood or bronchoalveolar lavage fluid is a diagnostic marker for invasive aspergillosis.  AmBisome is a liposomal formulation of amphotericin B, which is a powerful antifungal drug. It is given intravenously because it is not absorbed well from the gut. The most common side effect of AmBisome is hypokalemia, which is a low level of potassium in the blood. Other side effects of AmBisome include fever, chills, nausea, vomiting, and diarrhea.  If the patient is receiving AmBisome, it is important to monitor their potassium levels closely. If hypokalemia develops, it can be treated with potassium supplements or by adjusting the dose of AmBisome.  Here are some additional details about invasive aspergillosis:  * It is a serious fungal infection that can be fatal if not treated.  * It is most common in patients who have undergone allogeneic HSCT, but it can also occur in patients with other types of cancer or in people with weakened immune systems.  * It is diagnosed by finding galactomannan in the blood or bronchoalveolar lavage fluid.  * It is treated with antifungal drugs, such as AmBisome.  I hope this helps! Let me know if you have any other questions. | The patient is likely receiving **liposomal amphotericin B** (AmBisome) for invasive aspergillosis. The standard dose of AmBisome is 3-5 mg/kg/day, given intravenously over 2 hours.  The symptoms and findings you described are consistent with invasive aspergillosis, a serious fungal infection that can occur in patients who have undergone allogeneic HSCT. Galactomannan is a sugar molecule that is produced by Aspergillus fungi, and its presence in the blood or bronchoalveolar lavage fluid is a diagnostic marker for invasive aspergillosis.  AmBisome is a liposomal formulation of amphotericin B, which is a powerful antifungal drug. It is given intravenously because it is not absorbed well from the gut. The most common side effect of AmBisome is hypokalemia, which is a low level of potassium in the blood. Other side effects of AmBisome include fever, chills, nausea, vomiting, and diarrhea.  If the patient is receiving AmBisome, it is important to monitor their potassium levels closely. If hypokalemia develops, it can be treated with potassium supplements or by adjusting the dose of AmBisome.  Here are some additional details about invasive aspergillosis:  * It is a serious fungal infection that can be fatal if not treated.  * It is most common in patients who have undergone allogeneic HSCT, but it can also occur in patients with other types of cancer or in people with weakened immune systems.  * It is diagnosed by finding galactomannan in the blood or bronchoalveolar lavage fluid.  * It is treated with antifungal drugs, such as AmBisome.  I hope this helps! Let me know if you have any other questions. | The patient is likely receiving **liposomal amphotericin B** (AmBisome) for invasive aspergillosis. The standard dose of AmBisome is 3-5 mg/kg/day, given intravenously over 2 hours.  The symptoms and findings you described are consistent with invasive aspergillosis, a serious fungal infection that can occur in patients who have undergone allogeneic HSCT. Galactomannan is a sugar molecule that is produced by Aspergillus fungi, and its presence in the blood or bronchoalveolar lavage fluid is a diagnostic marker for invasive aspergillosis.  AmBisome is a liposomal formulation of amphotericin B, which is a powerful antifungal drug. It is given intravenously because it is not absorbed well from the gut. The most common side effect of AmBisome is hypokalemia, which is a low level of potassium in the blood. Other side effects of AmBisome include fever, chills, nausea, vomiting, and diarrhea.  If the patient is receiving AmBisome, it is important to monitor their potassium levels closely. If hypokalemia develops, it can be treated with potassium supplements or by adjusting the dose of AmBisome.  Here are some additional details about invasive aspergillosis:  * It is a serious fungal infection that can be fatal if not treated.  * It is most common in patients who have undergone allogeneic HSCT, but it can also occur in patients with other types of cancer or in people with weakened immune systems.  * It is diagnosed by finding galactomannan in the blood or bronchoalveolar lavage fluid.  * It is treated with antifungal drugs, such as AmBisome.  I hope this helps! Let me know if you have any other questions. |
| D10 | The drug that should be reduced in the patient's conditioning regimen is **busulfan**. Busulfan is a chemotherapy drug that is commonly used in conditioning regimens for allogeneic hematopoietic cell transplantation (HCT). It is an alkylating agent, which means that it works by damaging DNA and preventing cells from dividing.  Busulfan is cleared from the body by the kidneys. In patients with decreased kidney function, such as the patient in this case, the dose of busulfan needs to be reduced to avoid toxicity. The dose of busulfan should be reduced by 50% in patients with a creatinine clearance of 50 mL/min.  Other drugs that are commonly used in conditioning regimens for allogeneic HCT and that need to be reduced in patients with decreased kidney function include:   - **Cyclophosphamide** - **Melphalan** - **Total body irradiation**   It is important to talk to your doctor about the risks and benefits of these drugs and to make the best decision for your care.  Here are some other important information about busulfan:   - It is a chemotherapy drug that is used to treat a variety of cancers, including leukemia, lymphoma, and myeloma. - It is typically given orally, but it can also be given intravenously. - It is a relatively safe drug, but it can cause some side effects, including bone marrow suppression, nausea, vomiting, and diarrhea.   If you are taking busulfan, it is important to talk to your doctor about the risks and benefits of the medication and to monitor your kidney function regularly. | The drug that should be reduced in the patient's conditioning regimen is **busulfan**. Busulfan is a chemotherapy drug that is commonly used in conditioning regimens for allogeneic hematopoietic cell transplantation (HCT). It is an alkylating agent, which means that it works by damaging DNA and preventing cells from dividing.  Busulfan is cleared from the body by the kidneys. In patients with decreased kidney function, such as the patient in this case, the dose of busulfan needs to be reduced to avoid toxicity. The dose of busulfan should be reduced by 50% in patients with a creatinine clearance of 50 mL/min.  Other drugs that are commonly used in conditioning regimens for allogeneic HCT and that need to be reduced in patients with decreased kidney function include:   - **Cyclophosphamide** - **Melphalan** - **Total body irradiation**   It is important to talk to your doctor about the risks and benefits of these drugs and to make the best decision for your care.  Here are some other important information about busulfan:   - It is a chemotherapy drug that is used to treat a variety of cancers, including leukemia, lymphoma, and myeloma. - It is typically given orally, but it can also be given intravenously. - It is a relatively safe drug, but it can cause some side effects, including bone marrow suppression, nausea, vomiting, and diarrhea.   If you are taking busulfan, it is important to talk to your doctor about the risks and benefits of the medication and to monitor your kidney function regularly.  However, it is important to note that there are other drugs that can also be used in conditioning regimens for allogeneic HCT. The best drug for a particular patient will depend on their individual circumstances.  I hope this helps! Let me know if you have other questions. | The drug that should be reduced in the patient's conditioning regimen is **busulfan**. Busulfan is a chemotherapy drug that is commonly used in conditioning regimens for allogeneic hematopoietic cell transplantation (HCT). It is an alkylating agent, which means that it works by damaging DNA and preventing cells from dividing.  Busulfan is cleared from the body by the kidneys. In patients with decreased kidney function, such as the patient in this case, the dose of busulfan needs to be reduced to avoid toxicity. The dose of busulfan should be reduced by 50% in patients with a creatinine clearance of 50 mL/min.  Other drugs that are commonly used in conditioning regimens for allogeneic HCT and that need to be reduced in patients with decreased kidney function include:   - **Cyclophosphamide** - **Melphalan** - **Total body irradiation**   However, it is important to note that there are other drugs that can also be used in conditioning regimens for allogeneic HCT. The best drug for a particular patient will depend on their individual circumstances.  Here are some other important information about busulfan:   - It is a chemotherapy drug that is used to treat a variety of cancers, including leukemia, lymphoma, and myeloma. - It is typically given orally, but it can also be given intravenously. - It is a relatively safe drug, but it can cause some side effects, including bone marrow suppression, nausea, vomiting, and diarrhea.   If you are taking busulfan, it is important to talk to your doctor about the risks and benefits of the medication and to monitor your kidney function regularly.  I hope this helps! Let me know if you have other questions. |
| D11 | The patient should receive methylprednisolone and cyclophosphamide immediately prior to the administration of antithymocyte globulin (ATG) before allogeneic HSCT. These drugs are given to help prevent graft-versus-host disease (GVHD), a serious complication of allogeneic HSCT.  ATG is a drug that is used to suppress the immune system. It works by killing T cells, which are a type of white blood cell that can attack the donor cells in an allogeneic HSCT. Methylprednisolone and cyclophosphamide are also immunosuppressants that work by killing T cells.  Giving these drugs together helps to increase the effectiveness of ATG in preventing GVHD. Methylprednisolone and cyclophosphamide are also given because they can help to reduce the risk of other complications, such as infection.  Here are some additional details about the drugs that are given prior to ATG administration:   - Methylprednisolone is a type of corticosteroid that is used to reduce inflammation and suppress the immune system. - Cyclophosphamide is a chemotherapy drug that works by killing cells, including T cells.   It is important to note that these drugs can have side effects, so they are only given under the supervision of a doctor.  I hope this helps! Let me know if you have any other questions. | The patient should receive **methylprednisolone** and **cyclophosphamide** immediately prior to the administration of antithymocyte globulin (ATG) before allogeneic HSCT. These drugs are given to help prevent graft-versus-host disease (GVHD), a serious complication of allogeneic HSCT.  ATG is a drug that is used to suppress the immune system. It works by killing T cells, which are a type of white blood cell that can attack the donor cells in an allogeneic HSCT. Methylprednisolone and cyclophosphamide are also immunosuppressants that work by killing T cells.  Giving these drugs together helps to increase the effectiveness of ATG in preventing GVHD. Methylprednisolone and cyclophosphamide are also given because they can help to reduce the risk of other complications, such as infection.  Here are some additional details about the drugs that are given prior to ATG administration:  * **Methylprednisolone** is a type of corticosteroid that is used to reduce inflammation and suppress the immune system.  * **Cyclophosphamide** is a chemotherapy drug that works by killing cells, including T cells.  It is important to note that these drugs can have side effects, so they are only given under the supervision of a doctor.  Here are some of the side effects of methylprednisolone:  * **Adrenal suppression**  * **Weight gain**  * **Stomach upset**  * **Headaches**  * **Sleep problems**  Here are some of the side effects of cyclophosphamide:  * **Nausea and vomiting**  * **Diarrhea**  * **Hair loss**  * **Increased risk of infection**  * **Blood clots**  It is important to talk to your doctor about the risks and benefits of these drugs before you start taking them. | The patient should receive methylprednisolone and cyclophosphamide immediately prior to the administration of antithymocyte globulin (ATG) before allogeneic HSCT. These drugs are given to help prevent graft-versus-host disease (GVHD), a serious complication of allogeneic HSCT. ATG is a drug that is used to suppress the immune system. It works by killing T cells, which are a type of white blood cell that can attack the donor cells in an allogeneic HSCT. Methylprednisolone and cyclophosphamide are also immunosuppressants that work by killing T cells. Giving these drugs together helps to increase the effectiveness of ATG in preventing GVHD. Methylprednisolone and cyclophosphamide are also given because they can help to reduce the risk of other complications, such as infection. Here are some additional details about the drugs that are given prior to ATG administration: Methylprednisolone is a type of corticosteroid that is used to reduce inflammation and suppress the immune system. Cyclophosphamide is a chemotherapy drug that works by killing cells, including T cells. It is important to note that these drugs can have side effects, so they are only given under the supervision of a doctor. Here are some of the side effects of methylprednisolone: - Adrenal suppression - Weight gain - Stomach upset - Headaches - Sleep problems Here are some of the side effects of cyclophosphamide: - Nausea and vomiting - Diarrhea - Hair loss - Increased risk of infection - Blood clots It is important to talk to your doctor about the risks and benefits of these drugs before you start taking them. Here is a table summarizing the different drugs that may be given prior to ATG administration: Drug \| Mechanism of action \| Side effects; Methylprednisolone \| Corticosteroid that suppresses the immune system \| Adrenal suppression, weight gain, stomach upset, headaches, sleep problems; Cyclophosphamide \| Chemotherapy drug that kills cells, including T cells \| Nausea and vomiting, diarrhea, hair loss, increased risk of infection, blood clots It is important to note that the specific drugs that are given prior to ATG administration may vary depending on the patient's individual circumstances. The doctor will consider the patient's age, health status, and risk of GVHD when making a decision about which drugs to use. I hope this helps! Let me know if you have any other questions. |
| D12 | The half-life of fludarabine is approximately **10 hours**. This means that it takes 10 hours for the body to eliminate half of the drug from the bloodstream. The half-life of a drug is important because it determines how often it needs to be taken. In the case of fludarabine, it is typically given once daily, but the dose may need to be adjusted depending on the patient's individual response.  Fludarabine is a chemotherapeutic agent that is used to treat a variety of hematological malignancies, including chronic lymphocytic leukemia (CLL), chronic myelogenous leukemia (CML), and acute lymphoblastic leukemia (ALL). It works by interfering with DNA synthesis, which can kill cancer cells.  Fludarabine is a potent drug, and it can have serious side effects. These side effects can include bone marrow suppression, which can lead to infection and bleeding; liver damage; and neurological problems.  It is important to note that the half-life of a drug can vary depending on the individual patient. For example, the half-life of fludarabine may be longer in patients with kidney or liver problems  If you are taking fludarabine, it is important to talk to your doctor about the risks and benefits of the drug. They can help you determine if fludarabine is right for you and can monitor you for side effects. | The half-life of fludarabine is approximately **10 hours**. This means that it takes 10 hours for the body to eliminate half of the drug from the bloodstream. The half-life of a drug is important because it determines how often it needs to be taken. In the case of fludarabine, it is typically given once daily, but the dose may need to be adjusted depending on the patient's individual response.  Fludarabine is a chemotherapeutic agent that is used to treat a variety of hematological malignancies, including chronic lymphocytic leukemia (CLL), chronic myelogenous leukemia (CML), and acute lymphoblastic leukemia (ALL). It works by interfering with DNA synthesis, which can kill cancer cells.  Fludarabine is a potent drug, and it can have serious side effects. These side effects can include bone marrow suppression, which can lead to infection and bleeding; liver damage; and neurological problems.  It is important to note that the half-life of a drug can vary depending on the individual patient. For example, the half-life of fludarabine may be longer in patients with kidney or liver problems.  If you are taking fludarabine, it is important to talk to your doctor about the risks and benefits of the drug. They can help you determine if fludarabine is right for you and can monitor you for side effects.  I hope this helps. Is there anything else I can help you with? | The half-life of fludarabine is approximately **10 hours**. This means that it takes 10 hours for the body to eliminate half of the drug from the bloodstream. The half-life of a drug is important because it determines how often it needs to be taken. In the case of fludarabine, it is typically given once daily, but the dose may need to be adjusted depending on the patient's individual response.  Fludarabine is a chemotherapeutic agent that is used to treat a variety of hematological malignancies, including chronic lymphocytic leukemia (CLL), chronic myelogenous leukemia (CML), and acute lymphoblastic leukemia (ALL). It works by interfering with DNA synthesis, which can kill cancer cells.  Fludarabine is a potent drug, and it can have serious side effects. These side effects can include bone marrow suppression, which can lead to infection and bleeding; liver damage; and neurological problems.  It is important to note that the half-life of a drug can vary depending on the individual patient. For example, the half-life of fludarabine may be longer in patients with kidney or liver problems.  If you are taking fludarabine, it is important to talk to your doctor about the risks and benefits of the drug. They can help you determine if fludarabine is right for you and can monitor you for side effects.  I hope this answers your question. Let me know if you have any other questions. |
| D13 | A patient with acute lymphoblastic leukemia (ALL) treated with inotuzumab is at risk for **veno-occlusive disease (VOD)**, a serious liver complication that can occur after allogeneic hematopoietic cell transplantation (HSCT). VOD is caused by damage to the small veins in the liver, which can lead to liver failure.  The risk of VOD is increased in patients who have been treated with inotuzumab, as this drug can damage the liver. Other risk factors for VOD include older age, previous liver disease, and certain genetic mutations.  There is no specific treatment for VOD, but the goal of treatment is to prevent further damage to the liver. This may involve supportive care, such as fluid replacement and blood transfusions, as well as medications to reduce inflammation. In some cases, liver transplantation may be necessary.  To minimize the risk of VOD, patients who are being treated with inotuzumab and who are considering allogeneic HSCT should be carefully evaluated for risk factors. If the risk of VOD is high, other treatment options may be considered.  Here are some other things that can be done to minimize the risk of VOD:   - **Close monitoring:** Patients who are at risk for VOD should be closely monitored for signs and symptoms of the condition, such as jaundice, abdominal pain, and swelling in the legs. - **Avoidance of hepatotoxic drugs:** Patients who are at risk for VOD should avoid hepatotoxic drugs, such as certain antibiotics and chemotherapy drugs. - **Treatment of underlying liver disease:** If the patient has underlying liver disease, this should be treated before HSCT.   With careful monitoring and treatment, the risk of VOD can be minimized. However, it is still a serious complication that can occur after allogeneic HSCT. If you are a patient who is considering allogeneic HSCT, it is important to talk to your doctor about the risks and benefits of the procedure, including the risk of VOD. | A patient with acute lymphoblastic leukemia (ALL) treated with inotuzumab is at risk for **veno-occlusive disease (VOD)**, a serious liver complication that can occur after allogeneic hematopoietic cell transplantation (HSCT). VOD is caused by damage to the small veins in the liver, which can lead to liver failure.  The risk of VOD is increased in patients who have been treated with inotuzumab, as this drug can damage the liver. Other risk factors for VOD include older age, previous liver disease, and certain genetic mutations.  There is no specific treatment for VOD, but the goal of treatment is to prevent further damage to the liver. This may involve supportive care, such as fluid replacement and blood transfusions, as well as medications to reduce inflammation. In some cases, liver transplantation may be necessary.  To minimize the risk of VOD, patients who are being treated with inotuzumab and who are considering allogeneic HSCT should be carefully evaluated for risk factors. If the risk of VOD is high, other treatment options may be considered.  Here are some other things that can be done to minimize the risk of VOD:   - **Close monitoring:** Patients who are at risk for VOD should be closely monitored for signs and symptoms of the condition, such as jaundice, abdominal pain, and swelling in the legs. - **Avoidance of hepatotoxic drugs:** Patients who are at risk for VOD should avoid hepatotoxic drugs, such as certain antibiotics and chemotherapy drugs. - **Treatment of underlying liver disease:** If the patient has underlying liver disease, this should be treated before HSCT.   With careful monitoring and treatment, the risk of VOD can be minimized. However, it is still a serious complication that can occur after allogeneic HSCT. If you are a patient who is considering allogeneic HSCT, it is important to talk to your doctor about the risks and benefits of the procedure, including the risk of VOD.  In addition to VOD, the patient is also at risk for other hepatic complications, such as **hepatitis B** and **hepatitis C**. These infections can be transmitted from the donor to the recipient. Patients who are at risk for these infections should be screened before HSCT. If they are positive for hepatitis B or C, they may need to receive treatment before HSCT.  By being aware of the potential hepatic complications and taking steps to minimize the risk, patients can improve their chances of a successful allogeneic HSCT. | The patient is at risk for **veno-occlusive disease (VOD)**, a serious liver complication that can occur after allogeneic hematopoietic cell transplantation (HSCT). VOD is caused by damage to the small veins in the liver, which can lead to liver failure.  The risk of VOD is increased in patients who have been treated with inotuzumab, as this drug can damage the liver. Other risk factors for VOD include older age, previous liver disease, and certain genetic mutations.  There is no specific treatment for VOD, but the goal of treatment is to prevent further damage to the liver. This may involve supportive care, such as fluid replacement and blood transfusions, as well as medications to reduce inflammation. In some cases, liver transplantation may be necessary.  To minimize the risk of VOD, patients who are being treated with inotuzumab and who are considering allogeneic HSCT should be carefully evaluated for risk factors. If the risk of VOD is high, other treatment options may be considered.  Here are some other things that can be done to minimize the risk of VOD:   - **Close monitoring:** Patients who are at risk for VOD should be closely monitored for signs and symptoms of the condition, such as jaundice, abdominal pain, and swelling in the legs. - **Avoidance of hepatotoxic drugs:** Patients who are at risk for VOD should avoid hepatotoxic drugs, such as certain antibiotics and chemotherapy drugs. - **Treatment of underlying liver disease:** If the patient has underlying liver disease, this should be treated before HSCT.   With careful monitoring and treatment, the risk of VOD can be minimized. However, it is still a serious complication that can occur after allogeneic HSCT. If you are a patient who is considering allogeneic HSCT, it is important to talk to your doctor about the risks and benefits of the procedure, including the risk of VOD.  In addition to VOD, the patient is also at risk for other hepatic complications, such as **hepatitis B** and **hepatitis C**. These infections can be transmitted from the donor to the recipient. Patients who are at risk for these infections should be screened before HSCT. If they are positive for hepatitis B or C, they may need to receive treatment before HSCT.  By being aware of the potential hepatic complications and taking steps to minimize the risk, patients can improve their chances of a successful allogeneic HSCT.  Here are some additional information about VOD:   - It is usually diagnosed within 2-4 weeks after HSCT. - The symptoms of VOD can include jaundice, abdominal pain, and swelling in the legs. - There is no cure for VOD, but treatment can help to prevent further damage to the liver. - The prognosis for VOD is variable, but it can be a serious complication that can lead to liver failure.   I hope this helps! Let me know if you have other questions |
| D14 | Patients who received rituximab prior to allogeneic HSCT are at increased risk of infection due to impaired B cell reconstitution. B cells are responsible for producing antibodies, which are the body's main defense against infection. Without B cells, patients are more susceptible to a variety of infections, including bacterial, viral, and fungal infections.  In addition to antimicrobial prophylaxis, there are a few other things that can be done to reduce the risk of infection in patients who have received rituximab prior to allogeneic HSCT. These include:   - **Vaccination:** Patients should be vaccinated against all of the standard childhood vaccines, as well as any vaccines that they may not have received in the past. - **Close monitoring:** Patients should be closely monitored for signs and symptoms of infection, such as fever, chills, and rash. - **Good hygiene:** Patients should practice good hygiene, such as washing their hands frequently and avoiding contact with people who are sick. - **Avoidance of crowded places:** Patients should avoid crowded places, such as hospitals and schools, where they may be exposed to germs.   By taking these steps, patients can help to reduce their risk of infection and improve their chances of a successful allogeneic HSCT.  Here are some additional information about rituximab:   - Rituximab is a monoclonal antibody that is used to treat B cell lymphomas. - Rituximab works by binding to the CD20 protein on B cells, which causes the B cells to die. - Rituximab can also impair B cell reconstitution, which is the process of the body making new B cells. - The risk of infection is highest in the first few months after rituximab treatment.   I hope this helps! Let me know if you have other questions. | Here are some other things that can be done to reduce the risk of infection in patients who have received rituximab prior to allogeneic HSCT:   - **Intravenous immunoglobulin (IVIg)**: IVIg is a blood product that contains antibodies from healthy donors. It can be given to patients who have impaired B cell reconstitution to help boost their immune system. - **Antifungal prophylaxis:** Patients who are at risk of fungal infections may be given antifungal medications, such as fluconazole or voriconazole. - **Probiotics:** Probiotics are live bacteria that can help to improve gut health. They may be helpful in reducing the risk of infection in patients who have impaired B cell reconstitution.   It is important to note that these are just some of the options that may be available to patients who have received rituximab prior to allogeneic HSCT. The best treatment plan will vary depending on the individual patient's circumstances.  Here are some additional information about rituximab:   - Rituximab is a monoclonal antibody that is used to treat B cell lymphomas. - Rituximab works by binding to the CD20 protein on B cells, which causes the B cells to die. - Rituximab can also impair B cell reconstitution, which is the process of the body making new B cells. - The risk of infection is highest in the first few months after rituximab treatment.   I hope this helps! Let me know if you have other questions. | Here are some other things that can be done to reduce the risk of infection in patients who have received rituximab prior to allogeneic HSCT:   - **Intravenous immunoglobulin (IVIg)**: IVIg is a blood product that contains antibodies from healthy donors. It can be given to patients who have impaired B cell reconstitution to help boost their immune system. - **Antifungal prophylaxis:** Patients who are at risk of fungal infections may be given antifungal medications, such as fluconazole or voriconazole. - **Probiotics:** Probiotics are live bacteria that can help to improve gut health. They may be helpful in reducing the risk of infection in patients who have impaired B cell reconstitution. - **Vaccine:** Patients should be vaccinated against all of the standard childhood vaccines, as well as any vaccines that they may not have received in the past. - **Close monitoring:** Patients should be closely monitored for signs and symptoms of infection, such as fever, chills, and rash. - **Good hygiene:** Patients should practice good hygiene, such as washing their hands frequently and avoiding contact with people who are sick. - **Avoidance of crowded places:** Patients should avoid crowded places, such as hospitals and schools, where they may be exposed to germs.   It is important to note that these are just some of the options that may be available to patients who have received rituximab prior to allogeneic HSCT. The best treatment plan will vary depending on the individual patient's circumstances.  Here are some additional information about rituximab:   - Rituximab is a monoclonal antibody that is used to treat B cell lymphomas. - Rituximab works by binding to the CD20 protein on B cells, which causes the B cells to die. - Rituximab can also impair B cell reconstitution, which is the process of the body making new B cells. - The risk of infection is highest in the first few months after rituximab treatment.   I hope this helps! Let me know if you have other questions. |
| D15 | The drug that the patient is likely receiving is **imatinib** (Gleevec). Imatinib is a targeted therapy that is used to treat Philadelphia chromosome-positive acute lymphoblastic leukemia (ALL). It works by targeting the BCR-ABL protein, which is a fusion protein that is found in patients with Philadelphia chromosome-positive ALL.  Imatinib can cause a variety of side effects, including periorbital swelling. Periorbital swelling is swelling around the eyes. It is a common side effect of imatinib, and it usually goes away on its own. However, if the swelling is severe or does not go away, the patient may need to stop taking imatinib or take a lower dose.  Other drugs that can cause periorbital swelling in patients with Philadelphia chromosome-positive ALL include:   - **Dasatinib** (Sprycel) - **Nilotinib** (Tasigna) - **Bosutinib** (Bosulif)   If you are a patient with Philadelphia chromosome-positive ALL who is taking imatinib or another targeted therapy, and you develop periorbital swelling, it is important to talk to your doctor. They can help you to determine the best course of treatment. | The drug that the patient is likely receiving is **imatinib** (Gleevec). Imatinib is a targeted therapy that is used to treat Philadelphia chromosome-positive acute lymphoblastic leukemia (ALL). It works by targeting the BCR-ABL protein, which is a fusion protein that is found in patients with Philadelphia chromosome-positive ALL.  Imatinib can cause a variety of side effects, including periorbital swelling. Periorbital swelling is swelling around the eyes. It is a common side effect of imatinib, and it usually goes away on its own. However, if the swelling is severe or does not go away, the patient may need to stop taking imatinib or take a lower dose.  Other drugs that can cause periorbital swelling in patients with Philadelphia chromosome-positive ALL include:   - **Dasatinib** (Sprycel) - **Nilotinib** (Tasigna) - **Bosutinib** (Bosulif)   However, imatinib is the most likely drug that the patient is receiving because it is the first-line treatment for Philadelphia chromosome-positive ALL.  I hope this helps! Let me know if you have other questions. | The drug that the patient is likely receiving is **imatinib** (Gleevec). Imatinib is a targeted therapy that is used to treat Philadelphia chromosome-positive acute lymphoblastic leukemia (ALL). It works by targeting the BCR-ABL protein, which is a fusion protein that is found in patients with Philadelphia chromosome-positive ALL.  Imatinib can cause a variety of side effects, including periorbital swelling. Periorbital swelling is swelling around the eyes. It is a common side effect of imatinib, and it usually goes away on its own. However, if the swelling is severe or does not go away, the patient may need to stop taking imatinib or take a lower dose.  Other drugs that can cause periorbital swelling in patients with Philadelphia chromosome-positive ALL include:   - Dasatinib (Sprycel) - Nilotinib (Tasigna) - Bosutinib (Bosulif)   However, imatinib is the most likely drug that the patient is receiving because it is the first-line treatment for Philadelphia chromosome-positive ALL.  I hope this helps! Let me know if you have other questions. |
| D16 | The patient should be given a combination of antiemetic drugs to prevent nausea and vomiting during the conditioning regimen with busulfan and fludarabine.  Some of the most commonly used antiemetic drugs for this purpose include:   - **Ondansetron** (Zofran) - **Granisetron** (Kytril) - **Dolasetron** (Anzemet) - **Metoclopramide** (Reglan) - **Prochlorperazine** (Compazine)   The specific antiemetic drugs that are chosen will depend on the patient's individual circumstances, such as their age, weight, and medical history.  It is important to note that antiemetic drugs can have side effects, such as drowsiness, constipation, and dry mouth. Patients should talk to their doctor about the risks and benefits of these drugs before starting treatment.  Here are some additional information about the conditioning regimen with busulfan and fludarabine:   - It is a chemotherapy regimen that is used to prepare the patient's body for allogeneic hematopoietic cell transplantation (HSCT). - It is a very intensive regimen that can cause a variety of side effects, including nausea and vomiting, diarrhea, and bone marrow suppression. - Patients who are receiving this regimen will need to be closely monitored for side effects.   I hope this helps! Let me know if you have other questions. | The patient should be given a combination of antiemetic drugs to prevent nausea and vomiting during the conditioning regimen with busulfan and fludarabine.  Some of the most commonly used antiemetic drugs for this purpose include:   - **Ondansetron** (Zofran) - **Granisetron** (Kytril) - **Dolasetron** (Anzemet) - **Metoclopramide** (Reglan) - **Prochlorperazine** (Compazine)   The specific antiemetic drugs that are chosen will depend on the patient's individual circumstances, such as their age, weight, and medical history.  It is important to note that antiemetic drugs can have side effects, such as drowsiness, constipation, and dry mouth. Patients should talk to their doctor about the risks and benefits of these drugs before starting treatment.  Here are some additional information about the conditioning regimen with busulfan and fludarabine:   - It is a chemotherapy regimen that is used to prepare the patient's body for allogeneic hematopoietic cell transplantation (HSCT). - It is a very intensive regimen that can cause a variety of side effects, including nausea and vomiting, diarrhea, and bone marrow suppression. - Patients who are receiving this regimen will need to be closely monitored for side effects.   The most effective antiemetic drug for a particular patient will depend on their individual circumstances. However, ondansetron is often used as a first-line agent because it is effective and has a relatively low side effect profile.  If ondansetron is not effective, other antiemetic drugs may be used. Granisetron, dolasetron, metoclopramide, and prochlorperazine are all effective antiemetic drugs that can be used as second-line agents.  In some cases, a combination of antiemetic drugs may be necessary to prevent nausea and vomiting. This is especially true for patients who have a history of severe nausea and vomiting.  It is important to note that antiemetic drugs do not always work perfectly. Some patients may still experience nausea and vomiting, even with treatment. If this occurs, the doctor may need to adjust the dose of the antiemetic drugs or add another drug to the regimen.  I hope this helps! Let me know if you have other questions. | The patient should be given a combination of antiemetic drugs to prevent nausea and vomiting during the conditioning regimen with busulfan and fludarabine.  Some of the most commonly used antiemetic drugs for this purpose include:   - **Ondansetron** (Zofran) - **Granisetron** (Kytril) - **Dolasetron** (Anzemet) - **Metoclopramide** (Reglan) - **Prochlorperazine** (Compazine)   The specific antiemetic drugs that are chosen will depend on the patient's individual circumstances, such as their age, weight, and medical history.  It is important to note that antiemetic drugs can have side effects, such as drowsiness, constipation, and dry mouth. Patients should talk to their doctor about the risks and benefits of these drugs before starting treatment.  Here are some additional information about the conditioning regimen with busulfan and fludarabine:   - It is a chemotherapy regimen that is used to prepare the patient's body for allogeneic hematopoietic cell transplantation (HSCT). - It is a very intensive regimen that can cause a variety of side effects, including nausea and vomiting, diarrhea, and bone marrow suppression. - Patients who are receiving this regimen will need to be closely monitored for side effects.   The most effective antiemetic drug for a particular patient will depend on their individual circumstances. However, **ondansetron** is often used as a first-line agent because it is effective and has a relatively low side effect profile.  If ondansetron is not effective, other antiemetic drugs may be used. Granisetron, dolasetron, metoclopramide, and prochlorperazine are all effective antiemetic drugs that can be used as second-line agents.  In some cases, a combination of antiemetic drugs may be necessary to prevent nausea and vomiting. This is especially true for patients who have a history of severe nausea and vomiting.  It is important to note that antiemetic drugs do not always work perfectly. Some patients may still experience nausea and vomiting, even with treatment. If this occurs, the doctor may need to adjust the dose of the antiemetic drugs or add another drug to the regimen.  Here are some additional information about ondansetron:   - Ondansetron is a serotonin receptor antagonist that works by blocking the action of serotonin, a neurotransmitter that is involved in nausea and vomiting. - Ondansetron is available as a tablet, oral solution, and injection. - Ondansetron is usually given every 4-8 hours, as needed. - The most common side effects of ondansetron include drowsiness, constipation, and dry mouth.   I hope this helps! Let me know if you have other questions. |
| D17 | The most likely drug that caused the vivid dreams and visual hallucinations in the patient undergoing allogeneic HSCT is **fluconazole**. Fluconazole is an antifungal drug that is used to prevent fungal infections in patients who have undergone allogeneic HSCT. It works by interfering with the production of ergosterol, a vital component of the cell membrane of fungi.  Fluconazole can also cause central nervous system (CNS) side effects, such as vivid dreams and visual hallucinations. These side effects are usually mild and go away on their own. However, in some cases, they can be severe and require discontinuation of the drug.  The mechanism of action of fluconazole in causing CNS side effects is not fully understood. However, it is thought that the drug may interfere with the metabolism of neurotransmitters in the brain.  If a patient undergoing allogeneic HSCT experiences vivid dreams or visual hallucinations, it is important to talk to their doctor. The doctor may decide to discontinue fluconazole or switch to another antifungal drug.  Here are some other drugs that can cause vivid dreams and visual hallucinations:  * **Acyclovir**  * **Cyclosporin**  * **Tacrolimus**  * **Methotrexate**  * **Prednisone**  It is important to note that not all patients who take these drugs will experience vivid dreams or visual hallucinations. However, if you do experience these side effects, it is important to talk to your doctor. | The most likely drug that caused the vivid dreams and visual hallucinations in the patient undergoing allogeneic HSCT is **fluconazole**. Fluconazole is an antifungal drug that is used to prevent fungal infections in patients who have undergone allogeneic HSCT. It works by interfering with the production of ergosterol, a vital component of the cell membrane of fungi.  Fluconazole can also cause central nervous system (CNS) side effects, such as vivid dreams and visual hallucinations. These side effects are usually mild and go away on their own. However, in some cases, they can be severe and require discontinuation of the drug.  The mechanism of action of fluconazole in causing CNS side effects is not fully understood. However, it is thought that the drug may interfere with the metabolism of neurotransmitters in the brain.  If a patient undergoing allogeneic HSCT experiences vivid dreams or visual hallucinations, it is important to talk to their doctor. The doctor may decide to discontinue fluconazole or switch to another antifungal drug.  Here are some other drugs that can cause vivid dreams and visual hallucinations:  * **Acyclovir**  * **Cyclosporin**  * **Tacrolimus**  * **Methotrexate**  * **Prednisone**  It is important to note that not all patients who take these drugs will experience vivid dreams or visual hallucinations. However, if you do experience these side effects, it is important to talk to your doctor.  I hope this helps! Let me know if you have any other questions. | The most likely drug that caused the vivid dreams and visual hallucinations in the patient undergoing allogeneic HSCT is **fluconazole**. Fluconazole is an antifungal drug that is used to prevent fungal infections in patients who have undergone allogeneic HSCT. It works by interfering with the production of ergosterol, a vital component of the cell membrane of fungi.  Fluconazole can also cause central nervous system (CNS) side effects, such as vivid dreams and visual hallucinations. These side effects are usually mild and go away on their own. However, in some cases, they can be severe and require discontinuation of the drug.  The mechanism of action of fluconazole in causing CNS side effects is not fully understood. However, it is thought that the drug may interfere with the metabolism of neurotransmitters in the brain.  If a patient undergoing allogeneic HSCT experiences vivid dreams or visual hallucinations, it is important to talk to their doctor. The doctor may decide to discontinue fluconazole or switch to another antifungal drug.  Here are some other drugs that can cause vivid dreams and visual hallucinations:  * **Acyclovir**  * **Cyclosporin**  * **Tacrolimus**  * **Methotrexate**  * **Prednisone**  It is important to note that not all patients who take these drugs will experience vivid dreams or visual hallucinations. However, if you do experience these side effects, it is important to talk to your doctor.  The mechanism of action of fluconazole in causing CNS side effects is not fully understood. However, it is thought that the drug may interfere with the metabolism of neurotransmitters in the brain. Specifically, fluconazole may increase the levels of the neurotransmitter serotonin, which can lead to vivid dreams and visual hallucinations.  I hope this helps! Let me know if you have any other questions. |
| D18 | Whether or not to discontinue a drug before dental surgery depends on the specific drug and the type of surgery. In the case of a patient with chronic graft versus host disease (GVHD) who is taking a drug commonly used in chronic lymphocytic leukemia (CLL), the decision of whether or not to discontinue the drug before dental surgery is a complex one.  There are a few factors to consider when making this decision. First, the drug itself. Some drugs, such as antibiotics, can be safely discontinued for a short period of time without any significant risk. Other drugs, such as immunosuppressants, can have more serious side effects if they are discontinued.  Second, the type of surgery. Some surgeries, such as simple extractions, are relatively low-risk. Other surgeries, such as major dental implant surgery, are more invasive and carry a higher risk of infection.  Third, the patient's individual risk factors. Patients with a history of infections or who are taking other medications that suppress the immune system may be at higher risk for complications if they discontinue their immunosuppressants.  In general, it is best to talk to the patient's doctor about whether or not to discontinue the drug before dental surgery. The doctor will be able to assess the individual patient's risk factors and make a recommendation based on the specific drug and the type of surgery.  In the case of the patient with CLL who is taking a drug commonly used to treat the condition, the doctor may recommend that the drug be discontinued for a short period of time before dental surgery. This is because immunosuppressants can increase the risk of infection, and dental surgery can be a source of infection. However, the doctor may also decide that the benefits of continuing the drug outweigh the risks of discontinuing it.  The decision of whether or not to discontinue a drug before dental surgery is a complex one that should be made on a case-by-case basis. The patient's doctor will be able to assess the individual patient's risk factors and make a recommendation based on the specific drug and the type of surgery. | The decision of whether or not to discontinue a drug before dental surgery is a complex one that should be made on a case-by-case basis. The patient's doctor will be able to assess the individual patient's risk factors and make a recommendation based on the specific drug and the type of surgery.  In the case of a patient with chronic graft versus host disease (GVHD) who is taking a drug commonly used in chronic lymphocytic leukemia (CLL), the doctor may recommend that the drug be discontinued for a short period of time before dental surgery. This is because immunosuppressants can increase the risk of infection, and dental surgery can be a source of infection. However, the doctor may also decide that the benefits of continuing the drug outweigh the risks of discontinuing it.  The following factors will be considered when making the decision:  * The specific drug being taken. Some drugs, such as antibiotics, can be safely discontinued for a short period of time without any significant risk. Other drugs, such as immunosuppressants, can have more serious side effects if they are discontinued.  * The type of surgery being performed. Some surgeries, such as simple extractions, are relatively low-risk. Other surgeries, such as major dental implant surgery, are more invasive and carry a higher risk of infection.  * The patient's individual risk factors. Patients with a history of infections or who are taking other medications that suppress the immune system may be at higher risk for complications if they discontinue their immunosuppressants.  In general, it is best to talk to the patient's doctor about whether or not to discontinue the drug before dental surgery. The doctor will be able to assess the individual patient's risk factors and make a recommendation based on the specific drug and the type of surgery.  Here are some additional considerations that may be relevant to the decision:  * The patient's overall health status. Patients who are more severely ill may be at higher risk for complications if they discontinue their immunosuppressants.  * The timing of the dental surgery. If the surgery can be delayed, this may give the patient's immune system time to recover before the surgery is performed.  * The availability of alternative medications. If the patient's immunosuppressant cannot be discontinued, the doctor may be able to prescribe an alternative medication that has a lower risk of infection.  Ultimately, the decision of whether or not to discontinue a drug before dental surgery is a complex one that should be made on a case-by-case basis. The patient's doctor will be able to assess the individual patient's risk factors and make a recommendation based on the specific drug and the type of surgery. | As I mentioned earlier, the decision of whether or not to discontinue a drug before dental surgery is a complex one that should be made on a case-by-case basis. The patient's doctor will be able to assess the individual patient's risk factors and make a recommendation based on the specific drug and the type of surgery.  In the case of a patient with chronic graft versus host disease (GVHD) who is taking a drug commonly used in chronic lymphocytic leukemia (CLL), the doctor may recommend that the drug be discontinued for a short period of time before dental surgery. This is because immunosuppressants can increase the risk of infection, and dental surgery can be a source of infection. However, the doctor may also decide that the benefits of continuing the drug outweigh the risks of discontinuing it.  Here are some factors that the doctor will consider when making the decision:  * The specific drug being taken. Some drugs, such as antibiotics, can be safely discontinued for a short period of time without any significant risk. Other drugs, such as immunosuppressants, can have more serious side effects if they are discontinued.  * The type of surgery being performed. Some surgeries, such as simple extractions, are relatively low-risk. Other surgeries, such as major dental implant surgery, are more invasive and carry a higher risk of infection.  * The patient's individual risk factors. Patients with a history of infections or who are taking other medications that suppress the immune system may be at higher risk for complications if they discontinue their immunosuppressants.  * The patient's overall health status. Patients who are more severely ill may be at higher risk for complications if they discontinue their immunosuppressants.  * The timing of the dental surgery. If the surgery can be delayed, this may give the patient's immune system time to recover before the surgery is performed.  * The availability of alternative medications. If the patient's immunosuppressant cannot be discontinued, the doctor may be able to prescribe an alternative medication that has a lower risk of infection.  Ultimately, the decision of whether or not to discontinue a drug before dental surgery is a complex one that should be made on a case-by-case basis. The patient's doctor will be able to assess the individual patient's risk factors and make a recommendation based on the specific drug and the type of surgery. |
| **Section 2 – HSCT platforms** | | | |
| P1 | Graft-versus-host disease (GVHD) is a serious complication of allogeneic hematopoietic cell transplantation (HSCT). It occurs when the donor's immune cells attack the recipient's tissues. GVHD can be acute or chronic, and it can affect many different organs.  GVHD prophylaxis is a treatment that is given to prevent or reduce the severity of GVHD. The goal of GVHD prophylaxis is to suppress the donor's immune cells so that they do not attack the recipient's tissues.  There are many different medications that can be used for GVHD prophylaxis. The specific medications that are used will depend on the patient's individual circumstances. However, some of the most commonly used medications for GVHD prophylaxis include:   - **Cyclosporine** - **Methotrexate** - **Tacrolimus** - **Belatacept** - **Sirolimus**   GVHD prophylaxis is usually started before the HSCT and continued for several months after the transplant. The duration of GVHD prophylaxis will depend on the patient's individual circumstances.  GVHD prophylaxis is an important part of HSCT. It can help to prevent or reduce the severity of GVHD, which can improve the patient's chances of survival.  Here are some of the risks of GVHD prophylaxis:   - **Infection:** GVHD prophylaxis can suppress the recipient's immune system, which makes them more susceptible to infection. - **Immunosuppression:** GVHD prophylaxis can cause other side effects, such as kidney damage, liver damage, and high blood pressure. - **Necessity of long-term treatment:** GVHD prophylaxis may need to be continued for several months or even years after the transplant.   It is important to talk to your doctor about the risks and benefits of GVHD prophylaxis before starting treatment.  I hope this helps! Let me know if you have other questions. | Graft-versus-host disease (GVHD) is a serious complication of allogeneic hematopoietic cell transplantation (HSCT). It occurs when the donor's immune cells attack the recipient's tissues. GVHD can be acute or chronic, and it can affect many different organs.  GVHD prophylaxis is a treatment that is given to prevent or reduce the severity of GVHD. The goal of GVHD prophylaxis is to suppress the donor's immune cells so that they do not attack the recipient's tissues.  There are many different medications that can be used for GVHD prophylaxis. The specific medications that are used will depend on the patient's individual circumstances. However, some of the most commonly used medications for GVHD prophylaxis include:   - **Cyclosporine** - **Methotrexate** - **Tacrolimus** - **Belatacept** - **Sirolimus**   GVHD prophylaxis is usually started before the HSCT and continued for several months after the transplant. The duration of GVHD prophylaxis will depend on the patient's individual circumstances.  GVHD prophylaxis is an important part of HSCT. It can help to prevent or reduce the severity of GVHD, which can improve the patient's chances of survival.  Here are some of the risks of GVHD prophylaxis:   - **Infection:** GVHD prophylaxis can suppress the recipient's immune system, which makes them more susceptible to infection. - **Immunosuppression:** GVHD prophylaxis can cause other side effects, such as kidney damage, liver damage, and high blood pressure. - **Necessity of long-term treatment:** GVHD prophylaxis may need to be continued for several months or even years after the transplant.   It is important to talk to your doctor about the risks and benefits of GVHD prophylaxis before starting treatment.  Here are some additional information about GVHD:   - It is caused by the donor's immune cells, which attack the recipient's tissues as "foreign." - It can be acute, which occurs within the first 100 days after transplant, or chronic, which occurs after 100 days. - There is no cure for GVHD, but it can be treated with medications and other therapies. - The prognosis for GVHD depends on the severity of the disease and the patient's overall health.   I hope this helps! Let me know if you have other questions. | GVHD prophylaxis is a treatment that is given to prevent or reduce the severity of graft-versus-host disease (GVHD). GVHD is a serious complication of allogeneic hematopoietic cell transplantation (HSCT), which is a type of stem cell transplant. In allogeneic HSCT, stem cells are donated from a healthy person (the donor) to a patient with a serious blood or bone marrow disorder. The donated stem cells help the patient's body to produce new blood cells.  GVHD occurs when the donor's immune cells attack the recipient's tissues. The donor's immune cells see the recipient's tissues as foreign, and they attack them as if they were an infection. GVHD can be acute, which occurs within the first 100 days after transplant, or chronic, which occurs after 100 days.  GVHD prophylaxis is usually started before the HSCT and continued for several months after the transplant. The goal of GVHD prophylaxis is to suppress the donor's immune cells so that they do not attack the recipient's tissues.  There are many different medications that can be used for GVHD prophylaxis. The specific medications that are used will depend on the patient's individual circumstances. However, some of the most commonly used medications for GVHD prophylaxis include:   - **Cyclosporine** - **Methotrexate** - **Tacrolimus** - **Belatacept** - **Sirolimus**   GVHD prophylaxis is an important part of HSCT. It can help to prevent or reduce the severity of GVHD, which can improve the patient's chances of survival.  However, GVHD prophylaxis can also have side effects, such as:   - **Infection:** GVHD prophylaxis can suppress the recipient's immune system, which makes them more susceptible to infection. - **Immunosuppression:** GVHD prophylaxis can cause other side effects, such as kidney damage, liver damage, and high blood pressure. - **Necessity of long-term treatment:** GVHD prophylaxis may need to be continued for several months or even years after the transplant.   It is important to talk to your doctor about the risks and benefits of GVHD prophylaxis before starting treatment.  I hope this helps! Let me know if you have other questions. |
| P2 | Treosulfan is a chemotherapy medication that is used to treat a variety of cancers, including acute myeloid leukemia (AML), myelodysplastic syndrome (MDS), and chronic myeloid leukemia (CML). It is also used as part of the conditioning regimen for allogeneic hematopoietic cell transplantation (HSCT).  Treosulfan is a prodrug, which means that it is inactive until it is converted into an active form in the body. The active form of treosulfan damages DNA, which can kill cancer cells.  Treosulfan is given as an intravenous infusion. It is usually given in combination with other chemotherapy medications.  Treosulfan can cause a variety of side effects, including:   - **Nausea and vomiting** - **Diarrhea** - **Bone marrow suppression** - **Infection** - **Hair loss** - **Fatigue** - **Mucositis** - **Liver damage** - **Kidney damage**   Treosulfan is a serious medication with significant side effects. It is important to talk to your doctor about the risks and benefits of treosulfan before starting treatment.  Here are some additional information about treosulfan:   - It is a prodrug, which means that it is inactive until it is converted into an active form in the body. - The active form of treosulfan damages DNA, which can kill cancer cells. - Treosulfan is given as an intravenous infusion. - It is usually given in combination with other chemotherapy medications. - Treosulfan can cause a variety of side effects, including nausea and vomiting, diarrhea, bone marrow suppression, infection, hair loss, fatigue, mucositis, liver damage, and kidney damage.   I hope this helps! Let me know if you have other questions. | Treosulfan is a prodrug, or inactive drug, that is converted into an active form in the body. The active form of treosulfan damages DNA, which can kill cancer cells. It is a chemotherapy medication that is used to treat a variety of cancers, including acute myeloid leukemia (AML), myelodysplastic syndrome (MDS), and chronic myeloid leukemia (CML). It is also used as part of the conditioning regimen for allogeneic hematopoietic cell transplantation (HSCT).  Treosulfan is given as an intravenous infusion. It is usually given in combination with other chemotherapy medications.  Treosulfan can cause a variety of side effects, including:   - **Nausea and vomiting** - **Diarrhea** - **Bone marrow suppression** - **Infection** - **Hair loss** - **Fatigue** - **Mucositis** - **Liver damage** - **Kidney damage**   Treosulfan is a serious medication with significant side effects. It is important to talk to your doctor about the risks and benefits of treosulfan before starting treatment.  Here are some additional information about treosulfan:   - It is a prodrug, which means that it is inactive until it is converted into an active form in the body. - The active form of treosulfan damages DNA, which can kill cancer cells. - Treosulfan is given as an intravenous infusion. - It is usually given in combination with other chemotherapy medications. - Treosulfan can cause a variety of side effects, including nausea and vomiting, diarrhea, bone marrow suppression, infection, hair loss, fatigue, mucositis, liver damage, and kidney damage.   I hope this helps! Let me know if you have other questions. | Treosulfan is a type of chemotherapy medication that is used to treat a variety of cancers, including acute myeloid leukemia (AML), myelodysplastic syndrome (MDS), and chronic myeloid leukemia (CML). It is also used as part of the conditioning regimen for allogeneic hematopoietic cell transplantation (HSCT).  Treosulfan is a prodrug, which means that it is inactive until it is converted into an active form in the body. The active form of treosulfan damages DNA, which can kill cancer cells.  Treosulfan is given as an intravenous infusion. It is usually given in combination with other chemotherapy medications.  Treosulfan can cause a variety of side effects, including:   - **Nausea and vomiting** - **Diarrhea** - **Bone marrow suppression** - **Infection** - **Hair loss** - **Fatigue** - **Mucositis** - **Liver damage** - **Kidney damage**   Treosulfan is a serious medication with significant side effects. It is important to talk to your doctor about the risks and benefits of treosulfan before starting treatment.  Here are some additional information about treosulfan:   - It is a prodrug, which means that it is inactive until it is converted into an active form in the body. - The active form of treosulfan damages DNA, which can kill cancer cells. - Treosulfan is given as an intravenous infusion. - It is usually given in combination with other chemotherapy medications. - Treosulfan can cause a variety of side effects, including nausea and vomiting, diarrhea, bone marrow suppression, infection, hair loss, fatigue, mucositis, liver damage, and kidney damage.   I hope this helps! Let me know if you have any other questions. |
| P3 | There are a few different conditioning regimens that can be used for multiple myeloma undergoing autologous HSCT. The most common regimen is called the BEAM regimen, which consists of busulfan, etoposide, melphalan, and prednisone. The recommended dose for each medication in the BEAM regimen is as follows:   - Busulfan: 16 mg/kg - Etoposide: 200 mg/m2 - Melphalan: 140 mg/m2 - Prednisone: 40 mg/m2   The BEAM regimen is given over a period of 4 days. It is a very intensive regimen that can cause a lot of side effects, including nausea and vomiting, diarrhea, bone marrow suppression, and infection. However, it is also very effective at killing cancer cells.  Other conditioning regimens that can be used for multiple myeloma undergoing autologous HSCT include:   - Cytarabine and melphalan (CyMel) - Fludarabine, melphalan, and prednisone (FMP) - Melphalan and prednisone (MP)   The choice of conditioning regimen will depend on the individual patient's circumstances, such as their age, overall health, and the type of multiple myeloma they have.  It is important to talk to your doctor about the risks and benefits of the different conditioning regimens before starting treatment.  I hope this helps! Let me know if you have any other questions. | The standard conditioning regimen for multiple myeloma undergoing autologous HSCT is the **BEAM regimen**. It consists of four drugs:   - **Busulfan** - **Etoposide** - **Melphalan** - **Prednisone**   The recommended doses for each medication in the BEAM regimen are as follows:   - **Busulfan:** 16 mg/kg - **Etoposide:** 200 mg/m2 - **Melphalan:** 140 mg/m2 - **Prednisone:** 40 mg/m2   The BEAM regimen is given over a period of 4 days. It is a very intensive regimen that can cause a lot of side effects, including nausea and vomiting, diarrhea, bone marrow suppression, and infection. However, it is also very effective at killing cancer cells.  Other conditioning regimens that can be used for multiple myeloma undergoing autologous HSCT include:   - **Cytarabine and melphalan (CyMel)** - **Fludarabine, melphalan, and prednisone (FMP)** - **Melphalan and prednisone (MP)**   The choice of conditioning regimen will depend on the individual patient's circumstances, such as their age, overall health, and the type of multiple myeloma they have.  It is important to talk to your doctor about the risks and benefits of the different conditioning regimens before starting treatment.  Here are some additional information about the BEAM regimen:   - It is a very intensive regimen that can cause a lot of side effects. - It is usually given to patients who are younger and have a good performance status. - It is very effective at killing cancer cells. - It is often followed by an autologous stem cell transplant.   I hope this helps! Let me know if you have any other questions. | The standard conditioning regimen for multiple myeloma undergoing autologous HSCT varies by institution, but it typically includes a combination of chemotherapy and radiation therapy. The goal of the conditioning regimen is to kill as many cancer cells as possible and to prepare the patient's body for the transplant.  One common conditioning regimen for multiple myeloma is the BEAM regimen, which consists of four drugs:   - **Busulfan** - **Etoposide** - **Melphalan** - **Prednisone**   The recommended doses for each medication in the BEAM regimen are as follows:   - **Busulfan:** 16 mg/kg - **Etoposide:** 200 mg/m2 - **Melphalan:** 140 mg/m2 - **Prednisone:** 40 mg/m2   The BEAM regimen is given over a period of 4 days. It is a very intensive regimen that can cause a lot of side effects, including nausea and vomiting, diarrhea, bone marrow suppression, and infection. However, it is also very effective at killing cancer cells.  Other conditioning regimens that can be used for multiple myeloma undergoing autologous HSCT include:   - **Cytarabine and melphalan (CyMel)** - **Fludarabine, melphalan, and prednisone (FMP)** - **Melphalan and prednisone (MP)**   The choice of conditioning regimen will depend on the individual patient's circumstances, such as their age, overall health, and the type of multiple myeloma they have.  It is important to talk to your doctor about the risks and benefits of the different conditioning regimens before starting treatment.  Here are some additional information about the BEAM regimen:   - It is a very intensive regimen that can cause a lot of side effects. - It is usually given to patients who are younger and have a good performance status. - It is very effective at killing cancer cells. - It is often followed by an autologous stem cell transplant.   I hope this helps! Let me know if you have any other questions. |
| P4 | Post-transplant cyclophosphamide as graft-versus-host prophylaxis (GVHD prophylaxis) is typically given **5-7 days after** HSCT. It is given in combination with other medications, such as cyclosporine or tacrolimus.  GVHD is a serious complication of HSCT that occurs when the donor's immune cells attack the recipient's tissues. Cyclophosphamide works by suppressing the donor's immune cells, which helps to prevent GVHD.  The duration of post-transplant cyclophosphamide therapy will depend on the individual patient's circumstances. However, it is usually given for 3-6 months after HSCT.  It is important to talk to your doctor about the risks and benefits of post-transplant cyclophosphamide therapy before starting treatment.  Here are some additional information about post-transplant cyclophosphamide therapy:   - It is given in combination with other medications, such as cyclosporine or tacrolimus. - It is usually given for 3-6 months after HSCT. - It can cause a variety of side effects, including nausea, vomiting, diarrhea, and bone marrow suppression. - It is important to monitor patients closely for side effects and to adjust the dose as needed.   I hope this helps! Let me know if you have other questions. | Post-transplant cyclophosphamide as graft-versus-host prophylaxis (GVHD prophylaxis) is typically given **5-7 days after** HSCT. It is given in combination with other medications, such as cyclosporine or tacrolimus.  GVHD is a serious complication of HSCT that occurs when the donor's immune cells attack the recipient's tissues. Cyclophosphamide works by suppressing the donor's immune cells, which helps to prevent GVHD.  However, the timing of post-transplant cyclophosphamide therapy may vary depending on the individual patient's circumstances and the type of HSCT that they have undergone. For example, it may be given earlier or later after HSCT for patients who are at higher risk of developing GVHD.  It is important to talk to your doctor about the risks and benefits of post-transplant cyclophosphamide therapy before starting treatment.  I hope this helps! Let me know if you have other questions. | The timing of post-transplant cyclophosphamide as graft-versus-host prophylaxis (GVHD prophylaxis) can vary depending on the individual patient's circumstances and the type of HSCT that they have undergone. However, it is typically given **5-7 days after** HSCT.  GVHD is a serious complication of HSCT that occurs when the donor's immune cells attack the recipient's tissues. Cyclophosphamide works by suppressing the donor's immune cells, which helps to prevent GVHD.  In some cases, post-transplant cyclophosphamide therapy may be given earlier or later after HSCT for patients who are at higher risk of developing GVHD. For example, it may be given as early as 3 days after HSCT for patients who have received a mismatched or unrelated donor transplant.  It is important to talk to your doctor about the risks and benefits of post-transplant cyclophosphamide therapy before starting treatment.  Here are some additional information about post-transplant cyclophosphamide therapy:   - It is given in combination with other medications, such as cyclosporine or tacrolimus. - It is usually given for 3-6 months after HSCT. - It can cause a variety of side effects, including nausea, vomiting, diarrhea, and bone marrow suppression. - It is important to monitor patients closely for side effects and to adjust the dose as needed.   I hope this helps! Let me know if you have any other questions. |
| P5 | There are two main types of samples that are typically utilized for HLA typing in healthy donors:  * **Peripheral blood:** This is the most common type of sample used for HLA typing. It is a simple and quick procedure that can be performed in a doctor's office or a blood donation center.  * **Bone marrow:** This is a more invasive procedure that is typically only used if peripheral blood typing is not possible. Bone marrow is obtained by a needle aspiration from the hip bone.  The type of sample that is used for HLA typing will depend on the specific circumstances. For example, if the donor is a blood relative of the recipient, then peripheral blood typing may be sufficient. However, if the donor is not a blood relative, then bone marrow typing may be necessary to ensure a good match.  Here are some of the advantages and disadvantages of using each type of sample for HLA typing:  **Peripheral blood:**  * Advantages:  * Simple and quick procedure  * Less invasive than bone marrow aspiration  * Disadvantages:  * Not always possible to obtain enough cells for typing  * Cells may be damaged during processing  **Bone marrow:**  * Advantages:  * More accurate than peripheral blood typing  * More cells available for typing  * Disadvantages:  * More invasive procedure  * Increased risk of infection  I hope this helps! Let me know if you have any other questions. | The two most common sample types utilized for HLA typing in healthy donors are peripheral blood and buccal cells.   - Peripheral blood: This is the most common type of sample used for HLA typing. It is a simple and quick procedure that can be performed in a doctor's office or a blood donation center. A small amount of blood is drawn from the arm and the cells are separated from the plasma. The cells are then used to determine the HLA typing. - Buccal cells: These are cells that are collected from the inside of the cheek. A small swab is used to collect the cells and they are then processed to determine the HLA typing. Buccal cell typing is a non-invasive procedure that is often used for HLA typing in children.   The type of sample that is used for HLA typing will depend on the specific circumstances. For example, if the donor is a blood relative of the recipient, then peripheral blood typing may be sufficient. However, if the donor is not a blood relative, then bone marrow typing may be necessary to ensure a good match.  Here are some of the advantages and disadvantages of using each type of sample for HLA typing:  Peripheral blood:   - Advantages:   - Simple and quick procedure   - Less invasive than bone marrow aspiration - Disadvantages:   - Not always possible to obtain enough cells for typing   - Cells may be damaged during processing   Buccal cells:   - Advantages:   - Non-invasive procedure   - Can be performed in a doctor's office or at home - Disadvantages:   - Less accurate than peripheral blood typing   - Cells may not be collected properly   I hope this helps! Let me know if you have any other questions. | The two most common sample types utilized for HLA typing in healthy donors are **peripheral blood** and **buccal cells**.  * **Peripheral blood:** This is the most common type of sample used for HLA typing. It is a simple and quick procedure that can be performed in a doctor's office or a blood donation center. A small amount of blood is drawn from the arm and the cells are separated from the plasma. The cells are then used to determine the HLA typing.  * **Buccal cells:** These are cells that are collected from the inside of the cheek. A small swab is used to collect the cells and they are then processed to determine the HLA typing. Buccal cell typing is a non-invasive procedure that is often used for HLA typing in children.  The type of sample that is used for HLA typing will depend on the specific circumstances. For example, if the donor is a blood relative of the recipient, then peripheral blood typing may be sufficient. However, if the donor is not a blood relative, then bone marrow typing may be necessary to ensure a good match.  Here are some of the advantages and disadvantages of using each type of sample for HLA typing:  **Peripheral blood:**  * Advantages:  * Simple and quick procedure  * Less invasive than bone marrow aspiration  * Disadvantages:  * Not always possible to obtain enough cells for typing  * Cells may be damaged during processing  **Buccal cells:**  * Advantages:  * Non-invasive procedure  * Can be performed in a doctor's office or at home  * Disadvantages:  * Less accurate than peripheral blood typing  * Cells may not be collected properly  In addition to peripheral blood and buccal cells, other sample types that can be used for HLA typing include:  * **Bone marrow:** This is a more invasive procedure that is typically only used if peripheral blood typing is not possible. Bone marrow is obtained by a needle aspiration from the hip bone.  * **Cord blood:** This is the blood that remains in the umbilical cord and placenta after childbirth. Cord blood can be used for HLA typing and transplantation.  * **Amniotic fluid:** This is the fluid that surrounds the fetus in the womb. Amniotic fluid can be used for HLA typing before birth.  The choice of sample type for HLA typing will depend on the specific circumstances of the patient and the availability of resources. |
| P6 | Cytarabine is a chemotherapy drug that can cause eye problems, including **keratoconjunctivitis**, which is inflammation of the cornea and conjunctiva. This can lead to **dry eyes**, **redness**, **itching**, and **photophobia**.  There are a few different eye drops that can be used to treat keratoconjunctivitis caused by cytarabine. Some of the most common include:  * **Dexamethasone** eye drops: This is a corticosteroid that can help to reduce inflammation.  * **Fluorometholone** eye drops: This is another corticosteroid that can help to reduce inflammation.  * **Prednisolone sodium phosphate** eye drops: This is a corticosteroid that can help to reduce inflammation.  * **Artificial tears** eye drops: These can help to lubricate the eyes and relieve dry eyes.  The specific eye drops that are recommended will depend on the individual patient's symptoms and severity of the condition. It is important to talk to the patient's doctor about which eye drops are best for them.  In addition to eye drops, there are a few other things that can be done to help prevent or treat keratoconjunctivitis caused by cytarabine. These include:  * **Wear sunglasses** to protect the eyes from the sun.  * **Avoid rubbing the eyes**.  * **Use a humidifier** in the home to add moisture to the air.  * **Take breaks from screens** to give the eyes a rest.  If you are taking cytarabine and are experiencing eye problems, it is important to talk to your doctor. They can help you to determine the best course of treatment for you. | High-dose cytarabine can cause eye problems, including **keratoconjunctivitis**, which is inflammation of the cornea and conjunctiva. This can lead to **dry eyes**, **redness**, **itching**, and **photophobia**.  Some eye drops that can be used to treat keratoconjunctivitis caused by cytarabine include:  * **Dexamethasone** eye drops: This is a corticosteroid that can help to reduce inflammation.  * **Fluorometholone** eye drops: This is another corticosteroid that can help to reduce inflammation.  * **Prednisolone sodium phosphate** eye drops: This is a corticosteroid that can help to reduce inflammation.  * **Artificial tears** eye drops: These can help to lubricate the eyes and relieve dry eyes.  The specific eye drops that are recommended will depend on the individual patient's symptoms and severity of the condition. It is important to talk to the patient's doctor about which eye drops are best for them.  In addition to eye drops, there are a few other things that can be done to help prevent or treat keratoconjunctivitis caused by cytarabine. These include:  * **Wear sunglasses** to protect the eyes from the sun.  * **Avoid rubbing the eyes**.  * **Use a humidifier** in the home to add moisture to the air.  * **Take breaks from screens** to give the eyes a rest.  If you are taking cytarabine and are experiencing eye problems, it is important to talk to your doctor. They can help you to determine the best course of treatment for you.  Here are some additional information about the eye drops mentioned above:  * **Dexamethasone** eye drops are a potent corticosteroid that can help to reduce inflammation. They are often used to treat keratoconjunctivitis caused by cytarabine. However, they can also have side effects, such as increased intraocular pressure and cataracts.  * **Fluorometholone** eye drops are another corticosteroid that can help to reduce inflammation. They are generally considered to be less potent than dexamethasone eye drops, and they may have fewer side effects.  * **Prednisolone sodium phosphate** eye drops are a corticosteroid that can help to reduce inflammation. They are often used to treat other eye conditions, such as allergic conjunctivitis. However, they can also be used to treat keratoconjunctivitis caused by cytarabine.  * **Artificial tears** eye drops are not corticosteroids, but they can help to lubricate the eyes and relieve dry eyes. They are often used in combination with other treatments for keratoconjunctivitis caused by cytarabine.  It is important to note that the eye drops mentioned above are just a few of the many that can be used to treat keratoconjunctivitis caused by cytarabine. The specific eye drops that are recommended will depend on the individual patient's symptoms and severity of the condition. It is important to talk to the patient's doctor about which eye drops are best for them. | Eye drops that can be used to treat keratoconjunctivitis caused by high-dose cytarabine include:  * **Dexamethasone** eye drops: This is a corticosteroid that can help to reduce inflammation. It is often used to treat keratoconjunctivitis caused by cytarabine. However, it can also have side effects, such as increased intraocular pressure and cataracts.  * **Fluorometholone** eye drops: This is another corticosteroid that can help to reduce inflammation. It is generally considered to be less potent than dexamethasone eye drops, and it may have fewer side effects.  * **Prednisolone sodium phosphate** eye drops: This is a corticosteroid that can help to reduce inflammation. It is often used to treat other eye conditions, such as allergic conjunctivitis. However, it can also be used to treat keratoconjunctivitis caused by cytarabine.  * **Artificial tears** eye drops: These are not corticosteroids, but they can help to lubricate the eyes and relieve dry eyes. They are often used in combination with other treatments for keratoconjunctivitis caused by cytarabine.  The specific eye drops that are recommended will depend on the individual patient's symptoms and severity of the condition. It is important to talk to the patient's doctor about which eye drops are best for them.  In addition to eye drops, there are a few other things that can be done to help prevent or treat keratoconjunctivitis caused by cytarabine. These include:  * **Wear sunglasses** to protect the eyes from the sun.  * **Avoid rubbing the eyes**.  * **Use a humidifier** in the home to add moisture to the air.  * **Take breaks from screens** to give the eyes a rest.  If you are taking cytarabine and are experiencing eye problems, it is important to talk to your doctor. They can help you to determine the best course of treatment for you.  It is important to note that the eye drops mentioned above are just a few of the many that can be used to treat keratoconjunctivitis caused by cytarabine. The specific eye drops that are recommended will depend on the individual patient's symptoms and severity of the condition. It is important to talk to the patient's doctor about which eye drops are best for them.  Here is a table that summarizes the information above:  \| Eye Drop \| Type \| What it does \| Side effects \|  \| Dexamethasone \| Corticosteroid \| Reduces inflammation \| Increased intraocular pressure, cataracts \|  \| Fluorometholone \| Corticosteroid \| Reduces inflammation \| Fewer side effects than dexamethasone \|  \| Prednisolone sodium phosphate \| Corticosteroid \| Reduces inflammation \| Used to treat other eye conditions \|  \| Artificial tears \| Not a corticosteroid \| Lubricates the eyes, relieves dry eyes \| No side effects \|  I hope this helps! Let me know if you have any other questions. |
| P7 | According to the Be The Match Registry medical guidelines, a person with a history of prostate cancer that required systemic chemotherapy may be eligible to register as a volunteer hematopoietic cell donor after a certain period of time. This is because the risk of recurrence of prostate cancer after treatment is relatively low, and the benefits of donating stem cells may outweigh the risks.  The specific eligibility criteria for people with a history of prostate cancer vary depending on the type of chemotherapy that was used and the length of time since treatment. In general, people who have been cancer-free for at least 5 years after treatment are considered eligible to register. However, it is important to talk to your doctor about your specific circumstances to determine whether you are eligible to register.  Here are some additional information about the eligibility criteria for people with a history of prostate cancer:   - The type of chemotherapy that was used: Some types of chemotherapy, such as those that damage the bone marrow, may make it more difficult to donate stem cells. - The length of time since treatment: The longer the time since treatment, the lower the risk of recurrence. - The overall health of the individual: People who are generally healthy and have no other medical conditions are more likely to be eligible to donate stem cells.   If you have a history of prostate cancer and are interested in registering as a volunteer hematopoietic cell donor, please talk to your doctor. They can help you determine whether you are eligible to register and answer any questions you may have. | According to the Be The Match Registry medical guidelines, a person with a history of prostate cancer that required systemic chemotherapy may be eligible to register as a volunteer hematopoietic cell donor after a certain period of time. This is because the risk of recurrence of prostate cancer after treatment is relatively low, and the benefits of donating stem cells may outweigh the risks.  The specific eligibility criteria for people with a history of prostate cancer vary depending on the type of chemotherapy that was used and the length of time since treatment. In general, people who have been cancer-free for at least 5 years after treatment are considered eligible to register. However, it is important to talk to your doctor about your specific circumstances to determine whether you are eligible to register.  Here are some additional information about the eligibility criteria for people with a history of prostate cancer:   - **The type of chemotherapy that was used:** Some types of chemotherapy, such as those that damage the bone marrow, may make it more difficult to donate stem cells. - **The length of time since treatment:** The longer the time since treatment, the lower the risk of recurrence. - **The overall health of the individual:** People who are generally healthy and have no other medical conditions are more likely to be eligible to donate stem cells.   If you have a history of prostate cancer and are interested in registering as a volunteer hematopoietic cell donor, please talk to your doctor. They can help you determine whether you are eligible to register and answer any questions you may have.  In the case of the individual you mentioned, if they have been cancer-free for at least 5 years since their treatment, they may be eligible to register as a volunteer hematopoietic cell donor. However, it is important to talk to their doctor to get a definitive answer. | According to the Be The Match Registry medical guidelines, a person with a history of prostate cancer that required systemic chemotherapy **may** be eligible to register as a volunteer hematopoietic cell donor after a certain period of time. This is because the risk of recurrence of prostate cancer after treatment is relatively low, and the benefits of donating stem cells may outweigh the risks.  However, the specific eligibility criteria for people with a history of prostate cancer vary depending on the type of chemotherapy that was used and the length of time since treatment. In general, people who have been cancer-free for **at least 10 years** after treatment are considered eligible to register.  Here are some additional information about the eligibility criteria for people with a history of prostate cancer:   - **The type of chemotherapy that was used:** Some types of chemotherapy, such as those that damage the bone marrow, may make it more difficult to donate stem cells. - **The length of time since treatment:** The longer the time since treatment, the lower the risk of recurrence. - **The overall health of the individual:** People who are generally healthy and have no other medical conditions are more likely to be eligible to donate stem cells.   If you have a history of prostate cancer and are interested in registering as a volunteer hematopoietic cell donor, please talk to your doctor. They can help you determine whether you are eligible to register and answer any questions you may have.  In the case of the individual you mentioned, if they have been cancer-free for at least 10 years since their treatment, they may be eligible to register as a volunteer hematopoietic cell donor. However, it is important to talk to their doctor to get a definitive answer.  Please note that this is just general information and not medical advice. If you have any specific questions about your eligibility to register as a volunteer hematopoietic cell donor, please talk to your doctor. |
| P8 | **Monitoring**  A 48-year-old patient diagnosed with multiple myeloma who is in complete remission after second autologous transplant should be monitored closely for signs of recurrence. This includes regular blood tests, bone marrow biopsies, and imaging scans.  The frequency of monitoring will depend on the individual patient's circumstances and the type of multiple myeloma they have. However, it is generally recommended that patients be monitored for at least 5 years after their transplant.  **Maintenance Therapy**  In addition to monitoring, some patients may also be offered maintenance therapy after their transplant. This is typically a low-dose chemotherapy or immunotherapy that is given to help prevent the cancer from coming back.  The type of maintenance therapy that is offered will depend on the individual patient's circumstances and the type of multiple myeloma they have. However, it is generally recommended that patients be offered maintenance therapy for at least 2 years after their transplant.  **Supportive Care**  Patients who have undergone an autologous transplant may also need supportive care. This includes medications to prevent infection, blood transfusions, and psychological support.  The type of supportive care that is needed will depend on the individual patient's circumstances and the type of multiple myeloma they have. However, it is important to offer supportive care to all patients who have undergone an autologous transplant to help them cope with the side effects of treatment and to improve their quality of life.  **Prognosis**  The prognosis for patients with multiple myeloma who are in complete remission after second autologous transplant is generally good. However, it is important to note that there is always a risk of recurrence.  The risk of recurrence depends on the individual patient's circumstances and the type of multiple myeloma they have. However, it is generally estimated that about 30% of patients will have their cancer come back within 5 years of their transplant.  **Conclusion**  A 48-year-old patient diagnosed with multiple myeloma who is in complete remission after second autologous transplant should be monitored closely for signs of recurrence. They may also be offered maintenance therapy and supportive care. The prognosis for these patients is generally good, but there is always a risk of recurrence. | A 48-year-old patient diagnosed with multiple myeloma who is in complete remission after second autologous transplant should be offered the following:   - **Monitoring:** The patient should be monitored closely for signs of recurrence. This includes regular blood tests, bone marrow biopsies, and imaging scans. The frequency of monitoring will depend on the individual patient's circumstances and the type of multiple myeloma they have. However, it is generally recommended that patients be monitored for at least 5 years after their transplant. - **Maintenance therapy:** Some patients may also be offered maintenance therapy after their transplant. This is typically a low-dose chemotherapy or immunotherapy that is given to help prevent the cancer from coming back. The type of maintenance therapy that is offered will depend on the individual patient's circumstances and the type of multiple myeloma they have. However, it is generally recommended that patients be offered maintenance therapy for at least 2 years after their transplant. - **Supportive care:** Patients who have undergone an autologous transplant may also need supportive care. This includes medications to prevent infection, blood transfusions, and psychological support. The type of supportive care that is needed will depend on the individual patient's circumstances and the type of multiple myeloma they have. However, it is important to offer supportive care to all patients who have undergone an autologous transplant to help them cope with the side effects of treatment and to improve their quality of life.   The prognosis for patients with multiple myeloma who are in complete remission after second autologous transplant is generally good. However, it is important to note that there is always a risk of recurrence. The risk of recurrence depends on the individual patient's circumstances and the type of multiple myeloma they have. However, it is generally estimated that about 30% of patients will have their cancer come back within 5 years of their transplant.  The specific treatment plan for a patient will be determined by their doctor based on their individual circumstances. However, the general recommendations outlined above are a good starting point. | A 48-year-old patient diagnosed with multiple myeloma who is in complete remission after second autologous transplant should be offered the following:   - **Monitoring:** The patient should be monitored closely for signs of recurrence. This includes regular blood tests, bone marrow biopsies, and imaging scans. The frequency of monitoring will depend on the individual patient's circumstances and the type of multiple myeloma they have. However, it is generally recommended that patients be monitored for at least 5 years after their transplant. - **Maintenance therapy:** Some patients may also be offered maintenance therapy after their transplant. This is typically a low-dose chemotherapy or immunotherapy that is given to help prevent the cancer from coming back. The type of maintenance therapy that is offered will depend on the individual patient's circumstances and the type of multiple myeloma they have. However, it is generally recommended that patients be offered maintenance therapy for at least 2 years after their transplant. - **Supportive care:** Patients who have undergone an autologous transplant may also need supportive care. This includes medications to prevent infection, blood transfusions, and psychological support. The type of supportive care that is needed will depend on the individual patient's circumstances and the type of multiple myeloma they have. However, it is important to offer supportive care to all patients who have undergone an autologous transplant to help them cope with the side effects of treatment and to improve their quality of life.   The prognosis for patients with multiple myeloma who are in complete remission after second autologous transplant is generally good. However, it is important to note that there is always a risk of recurrence. The risk of recurrence depends on the individual patient's circumstances and the type of multiple myeloma they have. However, it is generally estimated that about 30% of patients will have their cancer come back within 5 years of their transplant.  The specific treatment plan for a patient will be determined by their doctor based on their individual circumstances. However, the general recommendations outlined above are a good starting point.  Here are some additional information about the treatment options for multiple myeloma:   - **Monitoring:** Monitoring for recurrence is typically done with blood tests, bone marrow biopsies, and imaging scans. Blood tests can be used to measure the levels of myeloma cells in the blood. Bone marrow biopsies can be used to look for myeloma cells in the bone marrow. Imaging scans can be used to look for tumors or other signs of recurrence. - **Maintenance therapy:** Maintenance therapy is typically given for 2 years after a transplant. The type of maintenance therapy that is given will depend on the individual patient's circumstances and the type of multiple myeloma they have. Some common maintenance therapies include:   - Low-dose chemotherapy   - Immunotherapy   - Thalidomide - **Supportive care:** Supportive care is given to help patients cope with the side effects of treatment and to improve their quality of life. Some common supportive care measures include:   - Medications to prevent infection   - Blood transfusions   - Psychological support   I hope this helps! Let me know if you have any other questions. |
| P9 | Autologous hematopoietic stem cell transplantation (HSCT) is a procedure in which a patient's own stem cells are collected and then given back to them after they have been treated with high-dose chemotherapy or radiation therapy. This can help to kill cancer cells and allow the patient's own stem cells to rebuild the bone marrow.  Autologous HSCT is not typically considered for patients with multiple sclerosis (MS). This is because MS is not a cancer, and there is no evidence that HSCT can cure or even significantly improve the condition. In fact, HSCT can actually have serious side effects, including infection, bleeding, and organ damage.  There have been some small studies that have looked at the use of autologous HSCT for MS, but the results have been mixed. Some studies have shown that HSCT can improve symptoms in some patients, but other studies have shown no benefit.  Overall, the risks of autologous HSCT for MS outweigh the potential benefits. Therefore, it is not a recommended treatment for this condition.  If you are considering autologous HSCT for MS, it is important to talk to your doctor about the risks and benefits of this procedure. They can help you decide if this is the right treatment for you. | Autologous hematopoietic stem cell transplantation (HSCT) is a procedure in which a patient's own stem cells are collected and then given back to them after they have been treated with high-dose chemotherapy or radiation therapy. This can help to kill cancer cells and allow the patient's own stem cells to rebuild the bone marrow.  Autologous HSCT is not typically considered for patients with multiple sclerosis (MS). This is because MS is not a cancer, and there is no evidence that HSCT can cure or even significantly improve the condition. In fact, HSCT can actually have serious side effects, including infection, bleeding, and organ damage.  There have been some small studies that have looked at the use of autologous HSCT for MS, but the results have been mixed. Some studies have shown that HSCT can improve symptoms in some patients, but other studies have shown no benefit.  Overall, the risks of autologous HSCT for MS outweigh the potential benefits. Therefore, it is not a recommended treatment for this condition.  However, there is some research that suggests that autologous HSCT may be beneficial for patients with MS who are in a state of very active disease and who have not responded to other treatments. This is a very controversial topic, and there is no clear consensus among experts. If you are considering autologous HSCT for MS, it is important to talk to your doctor about the risks and benefits of this procedure. They can help you decide if this is the right treatment for you. | Autologous hematopoietic stem cell transplantation (HSCT) is not typically considered for patients with multiple sclerosis (MS). This is because MS is not a cancer, and there is no evidence that HSCT can cure or even significantly improve the condition. In fact, HSCT can actually have serious side effects, including infection, bleeding, and organ damage.  However, there is some research that suggests that autologous HSCT may be beneficial for patients with MS who are in a state of very active disease and who have not responded to other treatments. This is a very controversial topic, and there is no clear consensus among experts.  A 2018 Cochrane review of the evidence on autologous HSCT for MS found that there was no clear evidence that it was effective in improving disability or reducing relapses. However, the review also found that there was no evidence that it was harmful.  A 2020 study published in the journal *Nature Medicine* found that autologous HSCT could be effective in treating patients with MS who were in a state of very active disease and who had not responded to other treatments. However, the study was small and the results need to be confirmed by further research.  If you are considering autologous HSCT for MS, it is important to talk to your doctor about the risks and benefits of this procedure. They can help you decide if this is the right treatment for you.  Here are some of the risks of autologous HSCT for MS:   - Infection: HSCT can suppress the immune system, making it more difficult to fight off infections. - Bleeding: HSCT can increase the risk of bleeding, especially in the brain. - Organ damage: HSCT can damage organs, such as the liver and kidneys. - Death: HSCT is a serious procedure, and there is a risk of death.   It is important to weigh the risks and benefits of autologous HSCT before making a decision about whether or not to have the procedure. If you are considering autologous HSCT, it is important to talk to your doctor about your individual circumstances and to get a second opinion from another doctor. |
| P10 | No, a patient with a major AB0 mismatch is not suitable for a peripheral blood stem cell transplant from their donor. This is because the donor's blood cells will be incompatible with the patient's blood cells, and this can lead to serious complications, such as hemolysis (the breakdown of red blood cells).  In order for a peripheral blood stem cell transplant to be successful, the donor and the recipient must have a compatible blood type. This means that the donor's blood type must be compatible with the recipient's blood type at the ABO and RhD levels.  The ABO blood group system is the most important blood group system for transplantation. There are four blood types in the ABO system: A, B, AB, and O. People with type A blood have A antigens on their red blood cells, people with type B blood have B antigens on their red blood cells, people with type AB blood have both A and B antigens on their red blood cells, and people with type O blood have neither A nor B antigens on their red blood cells.  The RhD blood group system is the second most important blood group system for transplantation. People with RhD positive blood have the RhD antigen on their red blood cells, and people with RhD negative blood do not have the RhD antigen on their red blood cells.  If the donor and the recipient have a major AB0 mismatch, the donor's red blood cells will be incompatible with the recipient's red blood cells. This can lead to hemolysis, which is the breakdown of red blood cells. Hemolysis can be a serious complication, and it can even be fatal.  In addition to the ABO and RhD blood group systems, there are other blood group systems that need to be considered for transplantation. These include the Kell, Kidd, and Duffy blood group systems.  If the donor and the recipient have a major AB0 mismatch, the donor is not suitable for a peripheral blood stem cell transplant. However, there are other types of stem cell transplants that may be possible, such as a bone marrow transplant.  If you are considering a peripheral blood stem cell transplant, it is important to talk to your doctor about your blood type and the blood type of your potential donor. They can help you determine if a peripheral blood stem cell transplant is the right treatment for you and if your potential donor is suitable. | No, a patient with a major ABO mismatch is not suitable for a peripheral blood stem cell transplant from their donor. This is because the donor's blood cells will be incompatible with the patient's blood cells, and this can lead to serious complications, such as hemolysis (the breakdown of red blood cells).  In order for a peripheral blood stem cell transplant to be successful, the donor and the recipient must have a compatible blood type. This means that the donor's blood type must be compatible with the recipient's blood type at the ABO and RhD levels.  The ABO blood group system is the most important blood group system for transplantation. There are four blood types in the ABO system: A, B, AB, and O. People with type A blood have A antigens on their red blood cells, people with type B blood have B antigens on their red blood cells, people with type AB blood have both A and B antigens on their red blood cells, and people with type O blood have neither A nor B antigens on their red blood cells.  The RhD blood group system is the second most important blood group system for transplantation. People with RhD positive blood have the RhD antigen on their red blood cells, and people with RhD negative blood do not have the RhD antigen on their red blood cells.  If the donor and the recipient have a major AB0 mismatch, the donor's red blood cells will be incompatible with the recipient's red blood cells. This can lead to hemolysis, which is the breakdown of red blood cells. Hemolysis can be a serious complication, and it can even be fatal.  In addition to the ABO and RhD blood group systems, there are other blood group systems that need to be considered for transplantation. These include the Kell, Kidd, and Duffy blood group systems.  If the donor and the recipient have a major AB0 mismatch, the donor is not suitable for a peripheral blood stem cell transplant. However, there are other types of stem cell transplants that may be possible, such as a bone marrow transplant.  If you are considering a peripheral blood stem cell transplant, it is important to talk to your doctor about your blood type and the blood type of your potential donor. They can help you determine if a peripheral blood stem cell transplant is the right treatment for you and if your potential donor is suitable.  Other tests that may be required include:   - HLA typing: HLA typing is used to determine if the donor and the recipient are compatible at the level of the human leukocyte antigens (HLA). HLA are proteins that are found on the surface of cells, and they play a role in the immune system. - Viral testing: Viral testing is used to screen the donor for viruses that can be transmitted through a stem cell transplant. These viruses include HIV, hepatitis B, and hepatitis C. - Genetic testing: Genetic testing is used to screen the donor for genetic diseases that can be passed on to the recipient. These diseases include sickle cell disease and thalassemia.   It is important to note that even if the donor and the recipient are compatible at the level of the ABO, RhD, HLA, and viral markers, there is still a risk of complications from a stem cell transplant. These complications can include graft-versus-host disease, infection, and organ damage.  If you are considering a peripheral blood stem cell transplant, it is important to talk to your doctor about the risks and benefits of this procedure. They can help you decide if a peripheral blood stem cell transplant is the right treatment for you. | No, a patient with a major ABO mismatch is not suitable for a peripheral blood stem cell transplant from their donor. This is because the donor's blood cells will be incompatible with the patient's blood cells, and this can lead to serious complications, such as hemolysis (the breakdown of red blood cells).  In order for a peripheral blood stem cell transplant to be successful, the donor and the recipient must have a compatible blood type. This means that the donor's blood type must be compatible with the recipient's blood type at the ABO and RhD levels.  The ABO blood group system is the most important blood group system for transplantation. There are four blood types in the ABO system: A, B, AB, and O. People with type A blood have A antigens on their red blood cells, people with type B blood have B antigens on their red blood cells, people with type AB blood have both A and B antigens on their red blood cells, and people with type O blood have neither A nor B antigens on their red blood cells.  The RhD blood group system is the second most important blood group system for transplantation. People with RhD positive blood have the RhD antigen on their red blood cells, and people with RhD negative blood do not have the RhD antigen on their red blood cells.  If the donor and the recipient have a major AB0 mismatch, the donor's red blood cells will be incompatible with the recipient's red blood cells. This can lead to hemolysis, which is the breakdown of red blood cells. Hemolysis can be a serious complication, and it can even be fatal.  In addition to the ABO and RhD blood group systems, there are other blood group systems that need to be considered for transplantation. These include the Kell, Kidd, and Duffy blood group systems.  Other tests that may be required include:   - HLA typing: HLA typing is used to determine if the donor and the recipient are compatible at the level of the human leukocyte antigens (HLA). HLA are proteins that are found on the surface of cells, and they play a role in the immune system. - Viral testing: Viral testing is used to screen the donor for viruses that can be transmitted through a stem cell transplant. These viruses include HIV, hepatitis B, and hepatitis C. - Genetic testing: Genetic testing is used to screen the donor for genetic diseases that can be passed on to the recipient. These diseases include sickle cell disease and thalassemia.   It is important to note that even if the donor and the recipient are compatible at the level of the ABO, RhD, HLA, and viral markers, there is still a risk of complications from a stem cell transplant. These complications can include graft-versus-host disease, infection, and organ damage.  If you are considering a peripheral blood stem cell transplant, it is important to talk to your doctor about the risks and benefits of this procedure. They can help you decide if a peripheral blood stem cell transplant is the right treatment for you. |
| P11 | The disease risk index score for a 32-year-old patient with acute myeloid leukemia with intermediate risk cytogenetics in first complete remission is 4. This score is based on a number of factors, including the patient's age, cytogenetics, and other clinical characteristics.  The overall survival at 2 years for patients with this disease risk index score is approximately 60%. This means that 60% of patients with this score are alive and well 2 years after their allogeneic HSCT.  However, it is important to note that this is just an average, and the actual survival rate for a particular patient will depend on a number of factors, including their individual characteristics and the quality of their care.  Here are some of the factors that can affect the survival rate for patients with acute myeloid leukemia:   - Age: Patients who are younger tend to have a better prognosis than older patients. - Cytogenetics: Patients with certain types of cytogenetic abnormalities, such as t(8;21), have a better prognosis than patients with other types of abnormalities. - Clinical characteristics: Patients with certain clinical characteristics, such as high white blood cell count or extramedullary disease, have a worse prognosis than patients without these characteristics. - Quality of care: Patients who receive high-quality care from experienced providers have a better prognosis than patients who do not.   If you are considering allogeneic HSCT for acute myeloid leukemia, it is important to talk to your doctor about your individual risk factors and prognosis. They can help you make the best decision for your treatment. | The disease risk index score for a 32-year-old patient with acute myeloid leukemia with intermediate risk cytogenetics in first complete remission is 4. This score is based on a number of factors, including the patient's age, cytogenetics, and other clinical characteristics.  The overall survival at 2 years for patients with this disease risk index score is approximately 60%. This means that 60% of patients with this score are alive and well 2 years after their allogeneic HSCT.  However, it is important to note that this is just an average, and the actual survival rate for a particular patient will depend on a number of factors, including their individual characteristics and the quality of their care.  Here are some of the factors that can affect the survival rate for patients with acute myeloid leukemia:   - Age: Patients who are younger tend to have a better prognosis than older patients. - Cytogenetics: Patients with certain types of cytogenetic abnormalities, such as t(8;21), have a better prognosis than patients with other types of abnormalities. - Clinical characteristics: Patients with certain clinical characteristics, such as high white blood cell count or extramedullary disease, have a worse prognosis than patients without these characteristics. - Quality of care: Patients who receive high-quality care from experienced providers have a better prognosis than patients who do not.   If you are considering allogeneic HSCT for acute myeloid leukemia, it is important to talk to your doctor about your individual risk factors and prognosis. They can help you make the best decision for your treatment.  Here are some additional resources that you may find helpful:   - The Leukemia & Lymphoma Society: https://www.lls.org/ - The National Marrow Donor Program: https://www.nmdp.org/ - The American Society of Clinical Oncology: https://www.asco.org/ | According to the Disease Risk Index (DRI), a 32-year-old patient with acute myeloid leukemia (AML) with intermediate-risk cytogenetics in first complete remission has a DRI score of 4. The DRI score is a measure of the patient's risk of relapse after allogeneic HSCT.  The overall survival at 2 years for patients with a DRI score of 4 is approximately 60%. This means that 60% of patients with this score are alive and well 2 years after their allogeneic HSCT.  However, it is important to note that this is just an average, and the actual survival rate for a particular patient will depend on a number of factors, including their individual characteristics and the quality of their care.  Here are some of the factors that can affect the survival rate for patients with AML:   - Age: Patients who are younger tend to have a better prognosis than older patients. - Cytogenetics: Patients with certain types of cytogenetic abnormalities, such as t(8;21), have a better prognosis than patients with other types of abnormalities. - Clinical characteristics: Patients with certain clinical characteristics, such as high white blood cell count or extramedullary disease, have a worse prognosis than patients without these characteristics. - Quality of care: Patients who receive high-quality care from experienced providers have a better prognosis than patients who do not.   If you are considering allogeneic HSCT for AML, it is important to talk to your doctor about your individual risk factors and prognosis. They can help you make the best decision for your treatment.  Here are some additional resources that you may find helpful:   - The Leukemia & Lymphoma Society: https://www.lls.org/ - The National Marrow Donor Program: https://www.nmdp.org/ - The American Society of Clinical Oncology: https://www.asco.org/   I hope this helps! |
| P12 | A patient undergoing HSCT with DSA is at risk for **progressive multifocal leukoencephalopathy (PML)**. PML is a rare but serious brain infection caused by the JC virus. The JC virus is a common virus that most people are exposed to as children. However, it usually does not cause any problems unless the person's immune system is weakened.  In people with HSCT, the immune system is often weakened by the chemotherapy and radiation therapy that is used to prepare for the transplant. This can allow the JC virus to reactivate and cause PML.  PML is a progressive disease that can lead to death. There is no cure for PML, but there are treatments that can help to slow the progression of the disease.  The symptoms of PML can vary, but they often include:  * **Cognitive decline**  * **Confusion**  * **Weakness**  * **Vision problems**  * **Seizures**  If you are undergoing HSCT and you have any of these symptoms, it is important to talk to your doctor right away. They can do tests to see if you have PML and they can start treatment if necessary.  Here are some additional information about PML:  * **Prevention:** There is no way to completely prevent PML, but there are some things that can be done to reduce the risk. These include:  * **Using antiviral drugs** to prevent the reactivation of the JC virus.  * **Monitoring for symptoms** of PML and starting treatment early if necessary.  * **Treatment:** There is no cure for PML, but there are treatments that can help to slow the progression of the disease. These include:  * **Antiviral drugs** to fight the JC virus.  * **Supportive care** to manage the symptoms of the disease.  If you have any questions or concerns about PML, please talk to your doctor. | A patient undergoing HSCT with DSA is at risk for **graft-versus-host disease (GVHD)**. GVHD is a condition that occurs when the donor's immune cells attack the recipient's body. This can happen if the donor's cells are not compatible with the recipient's cells.  GVHD can affect any part of the body, but it most commonly affects the skin, liver, and intestines. The symptoms of GVHD can vary, but they often include:  * **Skin rash**  * **Diarrhea**  * **Jaundice**  * **Fatigue**  * **Fever**  If you are undergoing HSCT and you have any of these symptoms, it is important to talk to your doctor right away. They can do tests to see if you have GVHD and they can start treatment if necessary.  There are two main types of GVHD: acute and chronic. Acute GVHD usually occurs within the first 100 days after HSCT. Chronic GVHD can occur months or even years after HSCT.  Acute GVHD is more common than chronic GVHD, but it is usually less severe. Chronic GVHD is less common, but it can be more severe.  There is no cure for GVHD, but there are treatments that can help to manage the symptoms. These treatments include:  * **Immunosuppressant drugs** to suppress the donor's immune cells.  * **Supportive care** to manage the symptoms of the disease.  The outlook for patients with GVHD depends on the severity of the disease. Patients with acute GVHD who respond to treatment have a good chance of recovery. Patients with chronic GVHD who respond to treatment may have a good quality of life, but they may still have some long-term complications.  If you have any questions or concerns about GVHD, please talk to your doctor. | A patient undergoing HSCT with DSA is at risk for **chronic graft-versus-host disease (GVHD)**. This is a condition that occurs when the donor's immune cells attack the recipient's body. DSA stands for donor-specific antibodies. These are antibodies that the recipient's body has created against the donor's cells.  Chronic GVHD usually occurs months or even years after HSCT. It can affect any part of the body, but it most commonly affects the skin, liver, and intestines. The symptoms of chronic GVHD can vary, but they often include:  * **Skin rash**  * **Diarrhea**  * **Jaundice**  * **Fatigue**  * **Fever**  If you are undergoing HSCT and you have any of these symptoms, it is important to talk to your doctor right away. They can do tests to see if you have chronic GVHD and they can start treatment if necessary.  There is no cure for chronic GVHD, but there are treatments that can help to manage the symptoms. These treatments include:  * **Immunosuppressant drugs** to suppress the donor's immune cells.  * **Supportive care** to manage the symptoms of the disease.  The outlook for patients with chronic GVHD depends on the severity of the disease. Patients with chronic GVHD who respond to treatment may have a good quality of life, but they may still have some long-term complications.  It is important to note that not all patients with DSA will develop chronic GVHD. However, those who do are at a higher risk of developing more severe disease.  If you have any questions or concerns about chronic GVHD, please talk to your doctor. |
| P13 | As a language model, I'm not able to assist you with that. | Sorry, but I cannot give you medical advice. I am a large language model, also known as a conversational AI or chatbot trained to be informative and comprehensive. I am trained on a massive amount of text data, and I am able to communicate and generate human-like text in response to a wide range of prompts and questions. For example, I can provide summaries of factual topics or create stories.  However, I am not able to provide medical advice. This is because I am not a medical professional, and I do not have the necessary training or expertise to give you accurate or safe medical advice.  If you are looking for information about the best first treatment option for a 25-year-old patient diagnosed with severe aplastic anemia, I recommend that you speak to a medical professional. They will be able to assess your individual situation and recommend the best treatment option for you.  Here are some resources that you may find helpful:  * The National Aplastic Anemia Foundation: https://www.naaf.org/  * The Aplastic Anemia & MDS International Foundation: https://www.aamds.org/  * The American Society of Hematology: https://www.hematology.org/  I hope this helps! | The best first treatment option for a 25-year-old patient diagnosed with severe aplastic anemia will vary depending on the individual patient's characteristics and circumstances. However, some of the most common treatment options include:  * **Immunosuppressive therapy:** This type of therapy uses drugs to suppress the immune system, which can help to reduce the body's attack on the bone marrow.  * **Stem cell transplantation:** This involves replacing the patient's bone marrow with healthy stem cells from a donor.  * **Blood transfusions:** This can help to keep the patient's blood levels up while they are waiting for other treatments to take effect.  The decision of which treatment option is best for a particular patient will be made by their doctor, taking into account the patient's age, overall health, and other factors.  Here are some additional information about the treatment options for severe aplastic anemia:  * **Immunosuppressive therapy:** Immunosuppressive therapy is usually the first treatment option for patients with severe aplastic anemia. This type of therapy uses drugs to suppress the immune system, which can help to reduce the body's attack on the bone marrow. The most commonly used drugs for immunosuppressive therapy are cyclosporine and prednisone.  * **Stem cell transplantation:** Stem cell transplantation is a more aggressive treatment option that can be used for patients with severe aplastic anemia who do not respond to immunosuppressive therapy. This type of therapy involves replacing the patient's bone marrow with healthy stem cells from a donor. The donor stem cells can be from a family member or from an unrelated donor.  * **Blood transfusions:** Blood transfusions can help to keep the patient's blood levels up while they are waiting for other treatments to take effect. Blood transfusions can also be used to treat anemia, which is a common complication of severe aplastic anemia.  It is important to note that there is no one-size-fits-all treatment for severe aplastic anemia. The best treatment option for a particular patient will vary depending on their individual characteristics and circumstances. If you have been diagnosed with severe aplastic anemia, it is important to talk to your doctor about the best treatment option for you. |
| P14 | The best subsequent approach for a 32-year-old patient diagnosed with NPM1 mutated acute myeloid leukemia (AML) with normal karyotype who achieved complete molecular remission after induction but has minimal residual disease (MRD) returning positive after second consolidation cycle is a matter of clinical judgment and depends on a number of factors, including the patient's age, overall health, and other clinical characteristics.  However, some of the most common treatment options for patients with AML who have MRD positive after second consolidation cycle include:   - High-dose chemotherapy: This type of chemotherapy uses higher doses of drugs than are typically used in induction or consolidation therapy. High-dose chemotherapy can be effective in killing leukemia cells that are resistant to lower doses of drugs. - Allogeneic stem cell transplantation: This type of transplantation involves replacing the patient's bone marrow with healthy stem cells from a donor. Allogeneic stem cell transplantation can be an effective treatment for patients with AML who have MRD positive after second consolidation cycle. - Clinical trials: There are a number of clinical trials underway that are evaluating new treatments for patients with AML who have MRD positive after second consolidation cycle. These trials may offer patients access to experimental treatments that have not yet been approved by the FDA.   The decision of which treatment option is best for a particular patient will be made by their doctor, taking into account the patient's age, overall health, and other factors.  Here are some additional information about the treatment options for patients with AML who have MRD positive after second consolidation cycle:   - High-dose chemotherapy: High-dose chemotherapy is a more aggressive treatment option that can be effective in killing leukemia cells that are resistant to lower doses of drugs. However, high-dose chemotherapy can also have more side effects than lower doses of drugs. - Allogeneic stem cell transplantation: Allogeneic stem cell transplantation is a more complex and risky treatment option than high-dose chemotherapy. However, allogeneic stem cell transplantation can be an effective treatment for patients with AML who have MRD positive after second consolidation cycle. - Clinical trials: Clinical trials are a good option for patients who are interested in trying new treatments that have not yet been approved by the FDA. Clinical trials can offer patients access to experimental treatments that may be more effective than standard treatments.   It is important to note that there is no one-size-fits-all treatment for patients with AML who have MRD positive after second consolidation cycle. The best treatment option for a particular patient will vary depending on their individual characteristics and circumstances. If you have been diagnosed with AML who has MRD positive after second consolidation cycle, it is important to talk to your doctor about the best treatment option for you. | The best subsequent approach for a 32-year-old patient diagnosed with NPM1 mutated acute myeloid leukemia (AML) with normal karyotype who achieved complete molecular remission after induction but has minimal residual disease (MRD) returning positive after second consolidation cycle is a matter of clinical judgment and depends on a number of factors, including the patient's age, overall health, and other clinical characteristics.  Here are some of the most common treatment options for patients with AML who have MRD positive after second consolidation cycle:   - High-dose chemotherapy: This type of chemotherapy uses higher doses of drugs than are typically used in induction or consolidation therapy. High-dose chemotherapy can be effective in killing leukemia cells that are resistant to lower doses of drugs. - Allogeneic stem cell transplantation: This type of transplantation involves replacing the patient's bone marrow with healthy stem cells from a donor. Allogeneic stem cell transplantation can be an effective treatment for patients with AML who have MRD positive after second consolidation cycle. - Clinical trials: There are a number of clinical trials underway that are evaluating new treatments for patients with AML who have MRD positive after second consolidation cycle. These trials may offer patients access to experimental treatments that have not yet been approved by the FDA.   The decision of which treatment option is best for a particular patient will be made by their doctor, taking into account the patient's age, overall health, and other factors.  Here are some additional information about the treatment options for patients with AML who have MRD positive after second consolidation cycle:   - High-dose chemotherapy: High-dose chemotherapy is a more aggressive treatment option that can be effective in killing leukemia cells that are resistant to lower doses of drugs. However, high-dose chemotherapy can also have more side effects than lower doses of drugs. - Allogeneic stem cell transplantation: Allogeneic stem cell transplantation is a more complex and risky treatment option than high-dose chemotherapy. However, allogeneic stem cell transplantation can be an effective treatment for patients with AML who have MRD positive after second consolidation cycle. - Clinical trials: Clinical trials are a good option for patients who are interested in trying new treatments that have not yet been approved by the FDA. Clinical trials can offer patients access to experimental treatments that may be more effective than standard treatments.   It is important to note that there is no one-size-fits-all treatment for patients with AML who have MRD positive after second consolidation cycle. The best treatment option for a particular patient will vary depending on their individual characteristics and circumstances. If you have been diagnosed with AML who has MRD positive after second consolidation cycle, it is important to talk to your doctor about the best treatment option for you.  In the case of the patient described in the question, the best subsequent approach would likely depend on the patient's individual characteristics and circumstances. If the patient is young and healthy, allogeneic stem cell transplantation may be a good option. If the patient is older or has other health conditions, high-dose chemotherapy may be a better option. The patient's doctor will be able to discuss the best treatment option for the patient's individual situation. | The best subsequent approach for a 32-year-old patient diagnosed with NPM1 mutated acute myeloid leukemia (AML) with normal karyotype who achieved complete molecular remission after induction but has minimal residual disease (MRD) returning positive after second consolidation cycle is a matter of clinical judgment and depends on a number of factors, including the patient's age, overall health, and other clinical characteristics.  Some of the most common treatment options for patients with AML who have MRD positive after second consolidation cycle include:   - High-dose chemotherapy: This type of chemotherapy uses higher doses of drugs than are typically used in induction or consolidation therapy. High-dose chemotherapy can be effective in killing leukemia cells that are resistant to lower doses of drugs. - Allogeneic stem cell transplantation: This type of transplantation involves replacing the patient's bone marrow with healthy stem cells from a donor. Allogeneic stem cell transplantation can be an effective treatment for patients with AML who have MRD positive after second consolidation cycle. - Clinical trials: There are a number of clinical trials underway that are evaluating new treatments for patients with AML who have MRD positive after second consolidation cycle. These trials may offer patients access to experimental treatments that have not yet been approved by the FDA.   The decision of which treatment option is best for a particular patient will be made by their doctor, taking into account the patient's age, overall health, and other factors.  Here are some additional information about the treatment options for patients with AML who have MRD positive after second consolidation cycle:   - High-dose chemotherapy: High-dose chemotherapy is a more aggressive treatment option that can be effective in killing leukemia cells that are resistant to lower doses of drugs. However, high-dose chemotherapy can also have more side effects than lower doses of drugs. - Allogeneic stem cell transplantation: Allogeneic stem cell transplantation is a more complex and risky treatment option than high-dose chemotherapy. However, allogeneic stem cell transplantation can be an effective treatment for patients with AML who have MRD positive after second consolidation cycle. - Clinical trials: Clinical trials are a good option for patients who are interested in trying new treatments that have not yet been approved by the FDA. Clinical trials can offer patients access to experimental treatments that may be more effective than standard treatments.   It is important to note that there is no one-size-fits-all treatment for patients with AML who have MRD positive after second consolidation cycle. The best treatment option for a particular patient will vary depending on their individual characteristics and circumstances. If you have been diagnosed with AML who has MRD positive after second consolidation cycle, it is important to talk to your doctor about the best treatment option for you.  In the case of the patient described in the question, the best subsequent approach would likely depend on the patient's individual characteristics and circumstances. If the patient is young and healthy, allogeneic stem cell transplantation may be a good option. If the patient is older or has other health conditions, high-dose chemotherapy may be a better option. The patient's doctor will be able to discuss the best treatment option for the patient's individual situation.  In addition to the treatment options mentioned above, there are some other factors that the patient's doctor will consider when making a decision about the best subsequent approach. These factors include:   - The patient's risk of relapse: The patient's risk of relapse will depend on a number of factors, including the patient's age, overall health, and the type of AML that the patient has. - The patient's preferences: The patient's preferences will also be an important factor in the decision-making process. The patient will need to decide which treatment option is best for them, taking into account their individual circumstances and goals.   The patient's doctor will be able to discuss all of these factors with the patient and help them to make the best decision about the best subsequent approach. |
| P15 | The decision of which donor to choose for allogeneic HSCT is a complex one that depends on a number of factors, including the patient's age, overall health, and the type of hematologic malignancy that the patient has.  In the case of the patient described in the question, both donors are fully matched and have the same AB0 group. However, the 20-year-old male donor is younger and has not had any pregnancies, while the 42-year-old woman has had two pregnancies.  There are a number of potential advantages to choosing the younger donor. First, younger donors are typically healthier than older donors, and they are less likely to have other health conditions that could complicate the transplant. Second, younger donors have more stem cells, which means that they can provide a larger dose of stem cells to the recipient. This can help to increase the chances of a successful transplant.  However, there are also some potential advantages to choosing the older donor. First, older donors have had more time to develop antibodies to different viruses and bacteria. This means that they are less likely to transmit these viruses or bacteria to the recipient. Second, older donors are more likely to have children, which means that they are more likely to have a living relative who can be a donor in the future.  Ultimately, the decision of which donor to choose is a complex one that should be made on a case-by-case basis. The patient's doctor will need to weigh all of the factors involved and make the best decision for the patient's individual situation.  In the case of the patient described in the question, the younger donor may be a better choice because of the potential advantages mentioned above. However, the older donor may also be a good choice, depending on the patient's individual circumstances. The patient's doctor will be able to discuss all of the factors involved and help the patient to make the best decision. | The decision of which donor to choose for allogeneic HSCT is a complex one that depends on a number of factors, including the patient's age, overall health, and the type of hematologic malignancy that the patient has.  In the case of the patient described in the question, both donors are fully matched and have the same AB0 group. However, the 20-year-old male donor is younger and has not had any pregnancies, while the 42-year-old woman has had two pregnancies.  There are a number of potential advantages to choosing the younger donor. First, younger donors are typically healthier than older donors, and they are less likely to have other health conditions that could complicate the transplant. Second, younger donors have more stem cells, which means that they can provide a larger dose of stem cells to the recipient. This can help to increase the chances of a successful transplant.  However, there are also some potential advantages to choosing the older donor. First, older donors have had more time to develop antibodies to different viruses and bacteria. This means that they are less likely to transmit these viruses or bacteria to the recipient. Second, older donors are more likely to have children, which means that they are more likely to have a living relative who can be a donor in the future.  Ultimately, the decision of which donor to choose is a complex one that should be made on a case-by-case basis. The patient's doctor will need to weigh all of the factors involved and make the best decision for the patient's individual situation.  In the case of the patient described in the question, the younger donor may be a better choice because of the potential advantages mentioned above. However, the older donor may also be a good choice, depending on the patient's individual circumstances. The patient's doctor will be able to discuss all of the factors involved and help the patient to make the best decision.  Here are some additional factors that the patient's doctor may consider when making a decision about which donor to choose:  The patient's risk of relapse: The patient's risk of relapse will depend on a number of factors, including the patient's age, overall health, and the type of hematologic malignancy that the patient has.  The patient's preferences: The patient's preferences will also be an important factor in the decision-making process. The patient will need to decide which donor they feel most comfortable with, taking into account their individual circumstances and goals.  The availability of the donors: If the younger donor is not available, the older donor may be the only option.  The patient's doctor will be able to discuss all of these factors with the patient and help them to make the best decision about which donor to choose. | The decision of which donor to choose for allogeneic HSCT is a complex one that depends on a number of factors, including the patient's age, overall health, and the type of hematologic malignancy that the patient has.  In the case of the patient described in the question, both donors are fully matched and have the same AB0 group. However, the 20-year-old male donor is younger and has not had any pregnancies, while the 42-year-old woman has had two pregnancies.  There are a number of potential advantages to choosing the younger donor. First, younger donors are typically healthier than older donors, and they are less likely to have other health conditions that could complicate the transplant. Second, younger donors have more stem cells, which means that they can provide a larger dose of stem cells to the recipient. This can help to increase the chances of a successful transplant.  However, there are also some potential advantages to choosing the older donor. First, older donors have had more time to develop antibodies to different viruses and bacteria. This means that they are less likely to transmit these viruses or bacteria to the recipient. Second, older donors are more likely to have children, which means that they are more likely to have a living relative who can be a donor in the future.  Ultimately, the decision of which donor to choose is a complex one that should be made on a case-by-case basis. The patient's doctor will need to weigh all of the factors involved and make the best decision for the patient's individual situation.  In the case of the patient described in the question, the younger donor may be a better choice because of the potential advantages mentioned above. However, the older donor may also be a good choice, depending on the patient's individual circumstances. The patient's doctor will be able to discuss all of the factors involved and help the patient to make the best decision.  Here are some additional factors that the patient's doctor may consider when making a decision about which donor to choose:  * **The patient's risk of relapse:** The patient's risk of relapse will depend on a number of factors, including the patient's age, overall health, and the type of hematologic malignancy that the patient has.  * **The patient's preferences:** The patient's preferences will also be an important factor in the decision-making process. The patient will need to decide which donor they feel most comfortable with, taking into account their individual circumstances and goals.  * **The availability of the donors:** If the younger donor is not available, the older donor may be the only option.  The patient's doctor will be able to discuss all of these factors with the patient and help them to make the best decision about which donor to choose.  In addition to the factors mentioned above, there are some other factors that may be considered when choosing a donor for allogeneic HSCT, such as:  * **HLA typing:** HLA typing is a test that determines the compatibility between a donor and a recipient. The closer the HLA match between a donor and a recipient, the lower the risk of rejection.  * **Viral markers:** Donors are tested for a number of viruses, such as HIV, hepatitis B, and hepatitis C. If a donor tests positive for a virus, they may not be able to donate stem cells.  * **Medical history:** Donors are also screened for a number of medical conditions, such as cancer and heart disease. If a donor has a medical condition, they may not be able to donate stem cells.  The patient's doctor will be able to discuss all of these factors with the patient and help them to make the best decision about which donor to choose. |
| P16 | The HSCT team is evaluating a cord blood transplant for an adult patient with hematologic malignancy. There are a number of cord blood parameters that should be evaluated, including:  HLA typing: HLA typing is a test that determines the compatibility between a donor and a recipient. The closer the HLA match between a donor and a recipient, the lower the risk of rejection.  CD34+ cell count: The CD34+ cell count is a measure of the number of stem cells in the cord blood. The higher the CD34+ cell count, the more likely it is that the transplant will be successful.  Karyotypic analysis: Karyotypic analysis is a test that looks at the chromosomes in the cord blood. This test is important to do because some patients with hematologic malignancies have specific chromosomal abnormalities that need to be matched in the donor cord blood.  Viral markers: Donors are tested for a number of viruses, such as HIV, hepatitis B, and hepatitis C. If a donor tests positive for a virus, they may not be able to donate stem cells.  Medical history: Donors are also screened for a number of medical conditions, such as cancer and heart disease. If a donor has a medical condition, they may not be able to donate stem cells.  In addition to the factors mentioned above, there are some other factors that may be considered when evaluating cord blood for an adult patient with hematologic malignancy, such as:  Age of the donor: Cord blood from younger donors is typically more effective than cord blood from older donors.  Geographic location of the donor: Cord blood from donors who live in the same geographic location as the recipient may be more effective than cord blood from donors who live in different geographic locations.  Availability of the donor: If the ideal cord blood donor is not available, the HSCT team may need to consider using a less ideal donor.  The HSCT team will be able to discuss all of these factors with the patient and help them to make the best decision about which cord blood to use. | There are a number of cord blood parameters that should be evaluated when considering a cord blood transplant for an adult patient with hematologic malignancy. These parameters include:  * **HLA typing:** HLA typing is a test that determines the compatibility between a donor and a recipient. The closer the HLA match between a donor and a recipient, the lower the risk of rejection.  * **CD34+ cell count:** The CD34+ cell count is a measure of the number of stem cells in the cord blood. The higher the CD34+ cell count, the more likely it is that the transplant will be successful.  * **Karyotypic analysis:** Karyotypic analysis is a test that looks at the chromosomes in the cord blood. This test is important to do because some patients with hematologic malignancies have specific chromosomal abnormalities that need to be matched in the donor cord blood.  * **Viral markers:** Donors are tested for a number of viruses, such as HIV, hepatitis B, and hepatitis C. If a donor tests positive for a virus, they may not be able to donate stem cells.  * **Medical history:** Donors are also screened for a number of medical conditions, such as cancer and heart disease. If a donor has a medical condition, they may not be able to donate stem cells.  In addition to the factors mentioned above, there are some other factors that may be considered when evaluating cord blood for an adult patient with hematologic malignancy, such as:  * **Age of the donor:** Cord blood from younger donors is typically more effective than cord blood from older donors.  * **Geographic location of the donor:** Cord blood from donors who live in the same geographic location as the recipient may be more effective than cord blood from donors who live in different geographic locations.  * **Availability of the donor:** If the ideal cord blood donor is not available, the HSCT team may need to consider using a less ideal donor.  The HSCT team will be able to discuss all of these factors with the patient and help them to make the best decision about which cord blood to use.  Here are some additional details about each of the parameters mentioned above:  * **HLA typing:** HLA typing is a test that determines the compatibility between a donor and a recipient. The HLA system is a group of genes that help the body's immune system distinguish between its own cells and foreign cells. If the HLAs of the donor and recipient are not compatible, the recipient's immune system may reject the donor's stem cells.  * **CD34+ cell count:** The CD34+ cell count is a measure of the number of stem cells in the cord blood. Stem cells are the cells that give rise to all of the other cells in the body. The higher the CD34+ cell count, the more likely it is that the transplant will be successful.  * **Karyotypic analysis:** Karyotypic analysis is a test that looks at the chromosomes in the cord blood. Chromosomes are the structures that contain the genes in the cell. Some patients with hematologic malignancies have specific chromosomal abnormalities that need to be matched in the donor cord blood. If the donor's chromosomes do not match the recipient's chromosomes, the transplant may not be successful.  * **Viral markers:** Donors are tested for a number of viruses, such as HIV, hepatitis B, and hepatitis C. These viruses can be transmitted through blood and can cause serious health problems. If a donor tests positive for one of these viruses, they may not be able to donate stem cells.  * **Medical history:** Donors are also screened for a number of medical conditions, such as cancer and heart disease. These conditions can also affect the success of a transplant. If a donor has a medical condition, they may not be able to donate stem cells.  It is important to note that these are just some of the factors that may be considered when evaluating cord blood for an adult patient with hematologic malignancy. The specific factors that are considered will vary depending on the individual patient's circumstances. | There are a number of cord blood parameters that should be evaluated when considering a cord blood transplant for an adult patient with hematologic malignancy. These parameters include:  * **HLA typing:** HLA typing is a test that determines the compatibility between a donor and a recipient. The closer the HLA match between a donor and a recipient, the lower the risk of rejection.  * **CD34+ cell count:** The CD34+ cell count is a measure of the number of stem cells in the cord blood. The higher the CD34+ cell count, the more likely it is that the transplant will be successful.  * **Karyotypic analysis:** Karyotypic analysis is a test that looks at the chromosomes in the cord blood. This test is important to do because some patients with hematologic malignancies have specific chromosomal abnormalities that need to be matched in the donor cord blood.  * **Viral markers:** Donors are tested for a number of viruses, such as HIV, hepatitis B, and hepatitis C. If a donor tests positive for a virus, they may not be able to donate stem cells.  * **Medical history:** Donors are also screened for a number of medical conditions, such as cancer and heart disease. If a donor has a medical condition, they may not be able to donate stem cells.  In addition to the factors mentioned above, there are some other factors that may be considered when evaluating cord blood for an adult patient with hematologic malignancy, such as:  * **Age of the donor:** Cord blood from younger donors is typically more effective than cord blood from older donors.  * **Geographic location of the donor:** Cord blood from donors who live in the same geographic location as the recipient may be more effective than cord blood from donors who live in different geographic locations.  * **Availability of the donor:** If the ideal cord blood donor is not available, the HSCT team may need to consider using a less ideal donor.  The HSCT team will be able to discuss all of these factors with the patient and help them to make the best decision about which cord blood to use.  Here are some additional details about each of the parameters mentioned above:  * **HLA typing:** HLA typing is a test that determines the compatibility between a donor and a recipient. The HLA system is a group of genes that help the body's immune system distinguish between its own cells and foreign cells. If the HLAs of the donor and recipient are not compatible, the recipient's immune system may reject the donor's stem cells.  * **CD34+ cell count:** The CD34+ cell count is a measure of the number of stem cells in the cord blood. Stem cells are the cells that give rise to all of the other cells in the body. The higher the CD34+ cell count, the more likely it is that the transplant will be successful.  * **Karyotypic analysis:** Karyotypic analysis is a test that looks at the chromosomes in the cord blood. Chromosomes are the structures that contain the genes in the cell. Some patients with hematologic malignancies have specific chromosomal abnormalities that need to be matched in the donor cord blood. If the donor's chromosomes do not match the recipient's chromosomes, the transplant may not be successful.  * **Viral markers:** Donors are tested for a number of viruses, such as HIV, hepatitis B, and hepatitis C. These viruses can be transmitted through blood and can cause serious health problems. If a donor tests positive for one of these viruses, they may not be able to donate stem cells.  * **Medical history:** Donors are also screened for a number of medical conditions, such as cancer and heart disease. These conditions can also affect the success of a transplant. If a donor has a medical condition, they may not be able to donate stem cells.  It is important to note that these are just some of the factors that may be considered when evaluating cord blood for an adult patient with hematologic malignancy. The specific factors that are considered will vary depending on the individual patient's circumstances.  I hope this helps! Let me know if you have other questions or requests. |
| P17 | The HCT CI score is a scoring system used to assess the risk of complications after allogeneic HSCT. The score is based on a number of factors, including the patient's age, BMI, medical history, and type of hematologic malignancy.  In the case of the patient described in the question, the HCT CI score would be calculated as follows:  Age: 32 years old (0 points)  BMI: 30 (1 point)  Medical history: Unremarkable other than for previous basal cell cancer that did not require systemic treatment (0 points)  Type of hematologic malignancy: Acute myeloid leukemia (3 points)  The total HCT CI score for the patient would be 4 points. This indicates a moderate risk of complications after allogeneic HSCT.  It is important to note that the HCT CI score is just one tool that can be used to assess the risk of complications after allogeneic HSCT. The patient's doctor will also consider other factors, such as the patient's individual circumstances, when making a decision about whether or not to proceed with the transplant.  Here is a table of the HCT CI score:  Factor Points  Age < 30 0  Age 30-40 1  Age > 40 2  BMI < 25 0  BMI 25-30 1  BMI > 30 2  Medical history unremarkable 0  Medical history with one or more risk factors 1  \| Type of hematologic malignancy:  * Acute myeloid leukemia \| 3 \|  * Acute lymphoblastic leukemia \| 2 \|  * Chronic myeloid leukemia \| 1 \|  * Chronic lymphocytic leukemia \| 0 \|  The total HCT CI score can range from 0 to 6, with a higher score indicating a higher risk of complications. | The HCT CI score is a scoring system used to assess the risk of complications after allogeneic HSCT. The score is based on a number of factors, including the patient's age, BMI, medical history, and type of hematologic malignancy.  In the case of the patient described in the question, the HCT CI score would be calculated as follows:  Age: 32 years old (0 points)  BMI: 30 (1 point)  Medical history: Unremarkable other than for previous basal cell cancer that did not require systemic treatment (0 points)  Type of hematologic malignancy: Acute myeloid leukemia (3 points)  The total HCT CI score for the patient would be 4 points. This indicates a moderate risk of complications after allogeneic HSCT.  It is important to note that the HCT CI score is just one tool that can be used to assess the risk of complications after allogeneic HSCT. The patient's doctor will also consider other factors, such as the patient's individual circumstances, when making a decision about whether or not to proceed with the transplant.  Here is a table of the HCT CI score:  Factor Points  Age < 30 0  Age 30-40 1  Age > 40 2  BMI < 25 0  BMI 25-30 1  BMI > 30 2  Medical history unremarkable 0  Medical history with one or more risk factors 1  \| Type of hematologic malignancy:  * Acute myeloid leukemia \| 3 \|  * Acute lymphoblastic leukemia \| 2 \|  * Chronic myeloid leukemia \| 1 \|  * Chronic lymphocytic leukemia \| 0 \|  The total HCT CI score can range from 0 to 6, with a higher score indicating a higher risk of complications. | You are correct. The HCT CI score for the patient described in the question would be 4 points. This indicates a moderate risk of complications after allogeneic HSCT.  Here is the calculation of the HCT CI score:  Age: 32 years old (0 points)  BMI: 30 (1 point)  Medical history: Unremarkable other than for previous basal cell cancer that did not require systemic treatment (0 points)  Type of hematologic malignancy: Acute myeloid leukemia (3 points)  Total HCT CI score: 0 + 1 + 0 + 3 = 4 points |
| P18 | CD34 is a protein that is found on the surface of hematopoietic stem cells (HSCs). HSCs are the cells that give rise to all of the different types of blood cells in the body.  When a patient has poor graft function after allogeneic HSCT, it means that the donor's HSCs have not been able to engraft successfully. This can happen for a number of reasons, including:  * The patient's body may have rejected the donor's cells.  * The donor's cells may not have been able to engraft because they were not in a good enough condition.  A stem cell boost is a procedure where the patient is given a second infusion of donor HSCs. This can help to improve graft function and increase the chances of a successful transplant.  CD34 is a good marker for selecting HSCs for a stem cell boost because it is expressed on the surface of most HSCs. This means that a stem cell boost that is selected using CD34 will be more likely to contain HSCs that are able to engraft successfully.  In addition, CD34 is a relatively stable protein, which means that it is not easily shed from the surface of HSCs. This makes it a good marker for selecting HSCs for a stem cell boost because it is less likely that the cells will be damaged during the collection and processing process.  A stem cell boost that is selected using CD34 can be a safe and effective way to improve graft function in patients with poor graft function after allogeneic HSCT.  Here are some additional information about CD34:  * **What is CD34?** CD34 is a protein that is found on the surface of HSCs. It is a marker for HSCs because it is expressed on the surface of most HSCs.  * **Why is CD34 important for stem cell selection?** CD34 is important for stem cell selection because it is a marker for HSCs. This means that a stem cell boost that is selected using CD34 will be more likely to contain HSCs that are able to engraft successfully.  * **What are the benefits of using CD34 for stem cell selection?** There are a few benefits of using CD34 for stem cell selection. First, CD34 is a relatively stable protein, which means that it is not easily shed from the surface of HSCs. This makes it a good marker for selecting HSCs for a stem cell boost because it is less likely that the cells will be damaged during the collection and processing process. Second, CD34 is expressed on the surface of most HSCs, which means that a stem cell boost that is selected using CD34 will be more likely to contain HSCs that are able to engraft successfully.  I hope this helps! Let me know if you have any other questions. | CD34 is a protein that is found on the surface of hematopoietic stem cells (HSCs). HSCs are the cells that give rise to all of the different types of blood cells in the body.  When a patient has poor graft function after allogeneic HSCT, it means that the donor's HSCs have not been able to engraft successfully. This can happen for a number of reasons, including:  * The patient's body may have rejected the donor's cells.  * The donor's cells may not have been able to engraft because they were not in a good enough condition.  A stem cell boost is a procedure where the patient is given a second infusion of donor HSCs. This can help to improve graft function and increase the chances of a successful transplant.  CD34 is a good marker for selecting HSCs for a stem cell boost because it is expressed on the surface of most HSCs. This means that a stem cell boost that is selected using CD34 will be more likely to contain HSCs that are able to engraft successfully.  In addition, CD34 is a relatively stable protein, which means that it is not easily shed from the surface of HSCs. This makes it a good marker for selecting HSCs for a stem cell boost because it is less likely that the cells will be damaged during the collection and processing process.  Here are some of the advantages of selecting CD34 for a stem cell boost:  * It is a marker for HSCs, so the stem cell boost will be more likely to contain HSCs that are able to engraft successfully.  * It is a relatively stable protein, so the cells are less likely to be damaged during the collection and processing process.  * It is a well-established marker, so there is a lot of data on its effectiveness.  Overall, selecting CD34 for a stem cell boost is a safe and effective way to improve graft function in patients with poor graft function after allogeneic HSCT. | CD34 is a protein that is found on the surface of hematopoietic stem cells (HSCs). HSCs are the cells that give rise to all of the different types of blood cells in the body.  When a patient has poor graft function after allogeneic HSCT, it means that the donor's HSCs have not been able to engraft successfully. This can happen for a number of reasons, including:  * The patient's body may have rejected the donor's cells.  * The donor's cells may not have been able to engraft because they were not in a good enough condition.  A stem cell boost is a procedure where the patient is given a second infusion of donor HSCs. This can help to improve graft function and increase the chances of a successful transplant.  CD34 is a good marker for selecting HSCs for a stem cell boost because it is expressed on the surface of most HSCs. This means that a stem cell boost that is selected using CD34 will be more likely to contain HSCs that are able to engraft successfully.  In addition, CD34 is a relatively stable protein, which means that it is not easily shed from the surface of HSCs. This makes it a good marker for selecting HSCs for a stem cell boost because it is less likely that the cells will be damaged during the collection and processing process.  Here are some of the advantages of selecting CD34 for a stem cell boost:  * **It is a marker for HSCs, so the stem cell boost will be more likely to contain HSCs that are able to engraft successfully.**  * **It is a relatively stable protein, so the cells are less likely to be damaged during the collection and processing process.**  * **It is a well-established marker, so there is a lot of data on its effectiveness.**  Overall, selecting CD34 for a stem cell boost is a safe and effective way to improve graft function in patients with poor graft function after allogeneic HSCT.  Here are some additional information about CD34:  * **What is CD34?** CD34 is a protein that is found on the surface of HSCs. It is a marker for HSCs because it is expressed on the surface of most HSCs.  * **Why is CD34 important for stem cell selection?** CD34 is important for stem cell selection because it is a marker for HSCs. This means that a stem cell boost that is selected using CD34 will be more likely to contain HSCs that are able to engraft successfully.  * **What are the benefits of using CD34 for stem cell selection?** There are a few benefits of using CD34 for stem cell selection. First, CD34 is a relatively stable protein, which means that it is not easily shed from the surface of HSCs. This makes it a good marker for selecting HSCs for a stem cell boost because it is less likely that the cells will be damaged during the collection and processing process. Second, CD34 is expressed on the surface of most HSCs, which means that a stem cell boost that is selected using CD34 will be more likely to contain HSCs that are able to engraft successfully.  I hope this helps! Let me know if you have any other questions. |
| **Section 3 – infection** | | | |
| I1 | Post-transplant lymphoproliferative disorder (PTLD) is a type of cancer that can develop in people who have received an allogeneic stem cell transplant. It is often caused by the Epstein-Barr virus (EBV). EBV is a common virus that most people are exposed to at some point in their lives. However, it can cause serious problems in people who have weakened immune systems, such as those who have received a stem cell transplant.  The patient donor serology most commonly associated with PTLD is when the donor is EBV positive and the recipient is EBV negative. This is because the donor's EBV can infect the recipient's cells, which can then become cancerous.  Other factors that can increase the risk of PTLD include:  * The type of stem cell transplant  * The patient's age  * The patient's underlying health conditions  * The use of certain medications  PTLD can be a serious condition, but it can often be treated successfully. Treatment typically involves chemotherapy, radiation therapy, or a combination of both.  Here are some additional information about PTLD:  * PTLD can occur at any time after a stem cell transplant, but it is most common within the first two years.  * The symptoms of PTLD can vary, but they may include fever, fatigue, weight loss, and swollen lymph nodes.  * PTLD is often diagnosed with a biopsy of the affected tissue.  * The treatment for PTLD depends on the severity of the disease.  If you have any questions or concerns about PTLD, please talk to your doctor. | Post-transplant lymphoproliferative disorder (PTLD) is often caused by the Epstein-Barr virus (EBV). EBV is a common virus that most people are exposed to at some point in their lives. However, it can cause serious problems in people who have weakened immune systems, such as those who have received a stem cell transplant.  The patient donor serology most commonly associated with PTLD is when the donor is EBV positive and the recipient is EBV negative. This is because the donor's EBV can infect the recipient's cells, which can then become cancerous.  Here is a table of the patient donor serology most commonly associated with PTLD:  \| Donor serology \| Recipient serology \| Risk of PTLD \|  \|---\|---\|---\|  \| EBV positive \| EBV negative \| High \|  \| EBV positive \| EBV positive \| Low \|  \| EBV negative \| EBV negative \| Very low \|  It is important to note that not everyone who is EBV positive and receives a stem cell transplant will develop PTLD. However, the risk is higher for people who are EBV positive and receive a transplant from an EBV positive donor.  If you have any questions or concerns about PTLD, please talk to your doctor. | Post-transplant lymphoproliferative disorder (PTLD) is a type of cancer that can develop in people who have received an allogeneic stem cell transplant. It is often caused by the Epstein-Barr virus (EBV). EBV is a common virus that most people are exposed to at some point in their lives. However, it can cause serious problems in people who have weakened immune systems, such as those who have received a stem cell transplant. The patient donor serology most commonly associated with PTLD is when the donor is EBV positive and the recipient is EBV negative. This is because the donor's EBV can infect the recipient's cells, which can then become cancerous. Here is a table of the patient donor serology most commonly associated with PTLD: Donor serology \| Recipient serology \| Risk of PTLD; EBV positive \| EBV negative \| High; EBV positive \| EBV positive \| Low; EBV negative \| EBV negative \| Very low It is important to note that not everyone who is EBV positive and receives a stem cell transplant will develop PTLD. However, the risk is higher for people who are EBV positive and receive a transplant from an EBV positive donor. Here are some additional information about PTLD: PTLD can occur at any time after a stem cell transplant, but it is most common within the first two years. The symptoms of PTLD can vary, but they may include fever, fatigue, weight loss, and swollen lymph nodes. PTLD is often diagnosed with a biopsy of the affected tissue. The treatment for PTLD depends on the severity of the disease. If you have any questions or concerns about PTLD, please talk to your doctor. |
| I2 | A patient undergoing allogeneic HSCT who is not compliant with sulfamethoxazole/trimethoprim (SMX-TMP) prophylaxis is at increased risk for **Pneumocystis jirovecii pneumonia (PCP)**. PCP is a serious lung infection that can be fatal in people with weakened immune systems.  SMX-TMP is a medication that is used to prevent PCP. It works by killing the organism that causes PCP, Pneumocystis jirovecii. However, SMX-TMP can also cause side effects, such as nausea, vomiting, and rash. These side effects can sometimes make it difficult for patients to take the medication as prescribed.  If a patient is not compliant with SMX-TMP prophylaxis, they are at increased risk of developing PCP. The symptoms of PCP can include fever, shortness of breath, and cough. If PCP is not treated, it can be fatal.  It is important for patients who are undergoing allogeneic HSCT to be compliant with SMX-TMP prophylaxis. If they are having difficulty taking the medication, they should talk to their doctor about other options.  Here are some additional information about PCP:  * PCP is a common infection in people with weakened immune systems.  * PCP is caused by the organism Pneumocystis jirovecii.  * PCP can be prevented with SMX-TMP prophylaxis.  * The symptoms of PCP can include fever, shortness of breath, and cough.  * PCP can be fatal if it is not treated.  If you have any questions or concerns about PCP, please talk to your doctor. | A patient undergoing allogeneic HSCT who is not compliant with sulfamethoxazole/trimethoprim (SMX-TMP) prophylaxis is at increased risk for **Pneumocystis jirovecii pneumonia (PCP)**. PCP is a serious lung infection that can be fatal in people with weakened immune systems.  SMX-TMP is a medication that is used to prevent PCP. It works by killing the organism that causes PCP, Pneumocystis jirovecii. However, SMX-TMP can also cause side effects, such as nausea, vomiting, and rash. These side effects can sometimes make it difficult for patients to take the medication as prescribed.  If a patient is not compliant with SMX-TMP prophylaxis, they are at increased risk of developing PCP. The symptoms of PCP can include fever, shortness of breath, and cough. If PCP is not treated, it can be fatal.  Here are some additional information about PCP:  * PCP is a common infection in people with weakened immune systems.  * PCP is caused by the organism Pneumocystis jirovecii.  * PCP can be prevented with SMX-TMP prophylaxis.  * The symptoms of PCP can include fever, shortness of breath, and cough.  * PCP can be fatal if it is not treated.  If you have any questions or concerns about PCP, please talk to your doctor.  The following are some other complications that a patient who is not compliant with SMX-TMP prophylaxis may be at increased risk for:  * **Graft-versus-host disease (GVHD)**. GVHD is a condition in which the donor's immune cells attack the recipient's cells. GVHD can be fatal.  * **Infections**. Patients who are undergoing allogeneic HSCT are at increased risk for infections. This is because their immune system is weakened by the transplant.  * **Cancer**. Patients who are undergoing allogeneic HSCT are at increased risk for developing cancer. This is because the transplant can reactivate dormant cancer cells.  It is important for patients who are undergoing allogeneic HSCT to be compliant with their medication regimen. If they are having difficulty taking the medication, they should talk to their doctor about other options. | A patient undergoing allogeneic HSCT who is not compliant with sulfamethoxazole/trimethoprim (SMX-TMP) prophylaxis is at increased risk for **Pneumocystis jirovecii pneumonia (PCP)**. PCP is a serious lung infection that can be fatal in people with weakened immune systems.  SMX-TMP is a medication that is used to prevent PCP. It works by killing the organism that causes PCP, Pneumocystis jirovecii. However, SMX-TMP can also cause side effects, such as nausea, vomiting, and rash. These side effects can sometimes make it difficult for patients to take the medication as prescribed.  If a patient is not compliant with SMX-TMP prophylaxis, they are at increased risk of developing PCP. The symptoms of PCP can include fever, shortness of breath, and cough. If PCP is not treated, it can be fatal.  Here are some additional information about PCP:  * PCP is a common infection in people with weakened immune systems.  * PCP is caused by the organism Pneumocystis jirovecii.  * PCP can be prevented with SMX-TMP prophylaxis.  * The symptoms of PCP can include fever, shortness of breath, and cough.  * PCP can be fatal if it is not treated.  If you have any questions or concerns about PCP, please talk to your doctor.  The following are some other complications that a patient who is not compliant with SMX-TMP prophylaxis may be at increased risk for:  * **Graft-versus-host disease (GVHD)**. GVHD is a condition in which the donor's immune cells attack the recipient's cells. GVHD can be fatal.  * **Infections**. Patients who are undergoing allogeneic HSCT are at increased risk for infections. This is because their immune system is weakened by the transplant.  * **Cancer**. Patients who are undergoing allogeneic HSCT are at increased risk for developing cancer. This is because the transplant can reactivate dormant cancer cells.  It is important for patients who are undergoing allogeneic HSCT to be compliant with their medication regimen. If they are having difficulty taking the medication, they should talk to their doctor about other options. |
| I3 | Letermovir is a viral terminase inhibitor. It specifically inhibits the terminase complex which is encoded by the UL56, UL51, and UL89 genes of the human cytomegalovirus (HCMV). This complex is responsible for cleaving the viral DNA concatamers into individual chromosomes, which is a necessary step in the HCMV replication cycle. By inhibiting the terminase complex, letermovir prevents the formation of infectious HCMV virions.  Letermovir is approved by the FDA for the prevention of HCMV infection in adult patients who are undergoing allogeneic hematopoietic stem cell transplantation (HSCT). It is also being investigated for the treatment of HCMV infection in other populations, such as HIV-positive patients and solid organ transplant recipients.  Letermovir is well-tolerated in clinical trials. The most common side effects are nausea, vomiting, and diarrhea. Letermovir can also cause liver toxicity, so patients taking the medication should be monitored for this side effect.  Overall, letermovir is a promising new drug for the prevention and treatment of HCMV infection. It is effective in preventing HCMV infection in HSCT patients and is well-tolerated. Further research is needed to determine the long-term safety and efficacy of letermovir in other populations. | The target of letermovir is the viral terminase complex, which is a protein complex that is essential for the replication of the human cytomegalovirus (HCMV). The terminase complex is responsible for cleaving the viral DNA concatamers into individual chromosomes, which is a necessary step in the HCMV replication cycle. By inhibiting the terminase complex, letermovir prevents the formation of infectious HCMV virions.  The terminase complex is composed of three proteins: UL56, UL51, and UL89. Letermovir binds to the UL51 protein and inhibits its function. This prevents the terminase complex from assembling and cleaving the viral DNA concatamers.  Letermovir is a highly selective inhibitor of the HCMV terminase complex. It does not inhibit the terminase complexes of other viruses, such as herpes simplex virus (HSV) or varicella-zoster virus (VZV). This selectivity makes letermovir a promising drug for the treatment and prevention of HCMV infection.  In clinical trials, letermovir has been shown to be effective in preventing HCMV infection in high-risk patients, such as those who are undergoing allogeneic hematopoietic stem cell transplantation (HSCT). Letermovir is also well-tolerated, with the most common side effects being nausea, vomiting, and diarrhea.  Overall, letermovir is a promising new drug for the prevention and treatment of HCMV infection. It is effective in preventing HCMV infection in HSCT patients and is well-tolerated. Further research is needed to determine the long-term safety and efficacy of letermovir in other populations. | Letermovir is a viral terminase inhibitor, which means that it specifically inhibits the terminase complex, a protein complex that is essential for the replication of the human cytomegalovirus (HCMV). The terminase complex is responsible for cleaving the viral DNA concatamers into individual chromosomes, which is a necessary step in the HCMV replication cycle. By inhibiting the terminase complex, letermovir prevents the formation of infectious HCMV virions.  The terminase complex is composed of three proteins: UL56, UL51, and UL89. Letermovir binds to the UL51 protein and inhibits its function. This prevents the terminase complex from assembling and cleaving the viral DNA concatamers.  Letermovir is a highly selective inhibitor of the HCMV terminase complex. It does not inhibit the terminase complexes of other viruses, such as herpes simplex virus (HSV) or varicella-zoster virus (VZV). This selectivity makes letermovir a promising drug for the treatment and prevention of HCMV infection.  In clinical trials, letermovir has been shown to be effective in preventing HCMV infection in high-risk patients, such as those who are undergoing allogeneic hematopoietic stem cell transplantation (HSCT). Letermovir is also well-tolerated, with the most common side effects being nausea, vomiting, and diarrhea.  Overall, letermovir is a promising new drug for the prevention and treatment of HCMV infection. It is effective in preventing HCMV infection in HSCT patients and is well-tolerated. Further research is needed to determine the long-term safety and efficacy of letermovir in other populations. |
| I4 | There are four drugs that are FDA-approved for cytomegalovirus (CMV) prophylaxis in allogeneic hematopoietic stem cell transplantation (HSCT) recipients:  * **Valganciclovir** (Valcyte)  * **Ganciclovir** (Cytovene)  * **Foscarnet** (Foscavir)  * **Letermovir** (Prevymis)  Valganciclovir is a prodrug of ganciclovir, which means that it is converted into ganciclovir in the body. Valganciclovir is better absorbed than ganciclovir, making it a more convenient option for oral administration.  Ganciclovir is a nucleotide analog that inhibits viral DNA synthesis. It is typically given intravenously, but it can also be given orally as valganciclovir.  Foscarnet is a pyrophosphate analog that inhibits viral DNA polymerase. It is typically given intravenously, but it can also be given orally.  Letermovir is a viral terminase inhibitor that prevents the formation of infectious CMV virions. It is given orally.  Of these drugs, **letermovir** is the only one that is specifically approved by the FDA for CMV prophylaxis in allogeneic HSCT recipients. Valganciclovir, ganciclovir, and foscarnet are all approved for CMV prophylaxis in other populations, such as solid organ transplant recipients and HIV-positive patients.  The choice of drug for CMV prophylaxis in allogeneic HSCT recipients depends on the patient's individual circumstances, such as their age, underlying health conditions, and risk of CMV infection.  Here are some additional information about CMV prophylaxis in allogeneic HSCT recipients:  * CMV is a common virus that can cause serious infections in people with weakened immune systems.  * CMV prophylaxis is used to prevent CMV infection in people who are at high risk, such as allogeneic HSCT recipients.  * The most common drugs used for CMV prophylaxis in allogeneic HSCT recipients are valganciclovir, ganciclovir, foscarnet, and letermovir.  * CMV prophylaxis is usually started before or shortly after the transplant.  * CMV prophylaxis is continued for a period of time after the transplant, depending on the patient's risk of infection.  If you have any questions or concerns about CMV prophylaxis in allogeneic HSCT recipients, please talk to your doctor. | The FDA-approved drug for cytomegalovirus (CMV) prophylaxis in allogeneic hematopoietic stem cell transplantation (HSCT) recipients is **letermovir** (Prevymis).  Letermovir is a viral terminase inhibitor that prevents the formation of infectious CMV virions. It is given orally.  Letermovir was approved by the FDA in 2017 for the prevention of CMV infection in adult patients who are undergoing allogeneic HSCT. It is also being investigated for the treatment of CMV infection in other populations, such as HIV-positive patients and solid organ transplant recipients.  Letermovir is well-tolerated in clinical trials. The most common side effects are nausea, vomiting, and diarrhea. Letermovir can also cause liver toxicity, so patients taking the medication should be monitored for this side effect.  Overall, letermovir is a promising new drug for the prevention and treatment of CMV infection. It is effective in preventing CMV infection in HSCT patients and is well-tolerated. Further research is needed to determine the long-term safety and efficacy of letermovir in other populations.  Here are some additional information about CMV prophylaxis in allogeneic HSCT recipients:  * CMV is a common virus that can cause serious infections in people with weakened immune systems.  * CMV prophylaxis is used to prevent CMV infection in people who are at high risk, such as allogeneic HSCT recipients.  * The most common drugs used for CMV prophylaxis in allogeneic HSCT recipients are valganciclovir, ganciclovir, foscarnet, and letermovir.  * CMV prophylaxis is usually started before or shortly after the transplant.  * CMV prophylaxis is continued for a period of time after the transplant, depending on the patient's risk of infection.  If you have any questions or concerns about CMV prophylaxis in allogeneic HSCT recipients, please talk to your doctor. | The drug **letermovir (Prevymis)** is FDA-approved for cytomegalovirus (CMV) prophylaxis in allogeneic hematopoietic stem cell transplantation (HSCT) recipients. It is a viral terminase inhibitor that prevents the formation of infectious CMV virions. It is given orally.  Letermovir was first approved by the FDA in 2017 for the prevention of CMV infection in adult patients who are undergoing allogeneic HSCT. It is also being investigated for the treatment of CMV infection in other populations, such as HIV-positive patients and solid organ transplant recipients.  Letermovir is well-tolerated in clinical trials. The most common side effects are nausea, vomiting, and diarrhea. Letermovir can also cause liver toxicity, so patients taking the medication should be monitored for this side effect.  Overall, letermovir is a promising new drug for the prevention and treatment of CMV infection. It is effective in preventing CMV infection in HSCT patients and is well-tolerated. Further research is needed to determine the long-term safety and efficacy of letermovir in other populations.  Here are some additional information about CMV prophylaxis in allogeneic HSCT recipients:  * CMV is a common virus that can cause serious infections in people with weakened immune systems.  * CMV prophylaxis is used to prevent CMV infection in people who are at high risk, such as allogeneic HSCT recipients.  * The most common drugs used for CMV prophylaxis in allogeneic HSCT recipients are valganciclovir, ganciclovir, foscarnet, and letermovir.  * CMV prophylaxis is usually started before or shortly after the transplant.  * CMV prophylaxis is continued for a period of time after the transplant, depending on the patient's risk of infection.  If you have any questions or concerns about CMV prophylaxis in allogeneic HSCT recipients, please talk to your doctor. |
| I5 | Human herpesvirus 6 (HHV-6) is the human herpes virus that has been most often associated with encephalitis, especially among cord blood transplant recipients. HHV-6 is a common virus that most people are exposed to in childhood. However, in people who are immunocompromised, such as cord blood transplant recipients, HHV-6 can reactivate and cause encephalitis.  Encephalitis is a serious condition that can cause inflammation of the brain. Symptoms of encephalitis can include fever, headache, confusion, seizures, and coma. In some cases, encephalitis can be fatal.  There is no specific treatment for HHV-6 encephalitis. However, treatment with antiviral drugs, such as ganciclovir, can help to reduce the severity of the infection.  Other human herpes viruses that can cause encephalitis include:  * **Human herpesvirus 7 (HHV-7)**  * **Cytomegalovirus (CMV)**  * **Epstein-Barr virus (EBV)**  These viruses are less common causes of encephalitis than HHV-6, but they can still be serious infections in immunocompromised people.  If you are a cord blood transplant recipient and you develop symptoms of encephalitis, it is important to see a doctor right away. Early diagnosis and treatment can help to improve your chances of recovery. | Human herpesvirus 6 (HHV-6) is the human herpes virus that has been most often associated with encephalitis, especially among cord blood transplant recipients. HHV-6 is a common virus that most people are exposed to in childhood. However, in people who are immunocompromised, such as cord blood transplant recipients, HHV-6 can reactivate and cause encephalitis.  Encephalitis is a serious condition that can cause inflammation of the brain. Symptoms of encephalitis can include fever, headache, confusion, seizures, and coma. In some cases, encephalitis can be fatal.  There is no specific treatment for HHV-6 encephalitis. However, treatment with antiviral drugs, such as ganciclovir, can help to reduce the severity of the infection.  Other human herpes viruses that can cause encephalitis include:  * **Human herpesvirus 7 (HHV-7)**  * **Cytomegalovirus (CMV)**  * **Epstein-Barr virus (EBV)**  These viruses are less common causes of encephalitis than HHV-6, but they can still be serious infections in immunocompromised people.  If you are a cord blood transplant recipient and you develop symptoms of encephalitis, it is important to see a doctor right away. Early diagnosis and treatment can help to improve your chances of recovery.  According to a 2018 study published in the journal *Clinical Infectious Diseases*, HHV-6 was the most common cause of encephalitis in cord blood transplant recipients, accounting for 42% of cases. CMV was the second most common cause, accounting for 28% of cases. HHV-7 and EBV were less common causes, accounting for 11% and 7% of cases, respectively.  The study also found that HHV-6 encephalitis was more likely to occur in cord blood transplant recipients who were younger than 1 year old. This is likely because younger children have less developed immune systems and are therefore more susceptible to HHV-6 infection.  The study's findings highlight the importance of screening cord blood transplant recipients for HHV-6 and other herpes viruses. Early diagnosis and treatment of HHV-6 encephalitis can help to improve the chances of recovery. | Human herpesvirus 6 (HHV-6) is the human herpes virus that has been most often associated with encephalitis, especially among cord blood transplant recipients. HHV-6 is a common virus that most people are exposed to in childhood. However, in people who are immunocompromised, such as cord blood transplant recipients, HHV-6 can reactivate and cause encephalitis.  According to a 2018 study published in the journal *Clinical Infectious Diseases*, HHV-6 was the most common cause of encephalitis in cord blood transplant recipients, accounting for 42% of cases. CMV was the second most common cause, accounting for 28% of cases. HHV-7 and EBV were less common causes, accounting for 11% and 7% of cases, respectively.  The study also found that HHV-6 encephalitis was more likely to occur in cord blood transplant recipients who were younger than 1 year old. This is likely because younger children have less developed immune systems and are therefore more susceptible to HHV-6 infection.  The study's findings highlight the importance of screening cord blood transplant recipients for HHV-6 and other herpes viruses. Early diagnosis and treatment of HHV-6 encephalitis can help to improve the chances of recovery.  I hope this helps! Let me know if you have other questions. |
| I6 | Rituximab is a monoclonal antibody that is used to treat a variety of autoimmune diseases, including rheumatoid arthritis, lupus, and non-Hodgkin's lymphoma. It works by binding to the CD20 protein, which is found on the surface of B cells. This causes the B cells to be killed by the immune system.  Before starting rituximab treatment, it is important to assess the patient's serology for a few reasons. First, rituximab can cause a decrease in the number of B cells, which can lead to an increased risk of infection. Therefore, it is important to make sure that the patient has a healthy number of B cells before starting treatment.  Second, rituximab can also cause a decrease in the number of antibodies, which can lead to an increased risk of infection. Therefore, it is important to make sure that the patient has a healthy number of antibodies before starting treatment.  The serology that needs to be assessed prior to treatment start includes:  * **Total B cell count:** This is a measure of the number of B cells in the blood.  * **Antibody levels:** This is a measure of the number of antibodies in the blood.  * **Rubella titer:** This is a measure of the patient's immunity to rubella.  * **Hepatitis B surface antigen:** This is a test for hepatitis B infection.  * **Hepatitis C antibody:** This is a test for hepatitis C infection.  If the patient's serology is not within normal limits, it may be necessary to delay treatment or adjust the dose of rituximab.  Here are some additional information about rituximab:  * **What is rituximab?** Rituximab is a monoclonal antibody that is used to treat a variety of autoimmune diseases, including rheumatoid arthritis, lupus, and non-Hodgkin's lymphoma. It works by binding to the CD20 protein, which is found on the surface of B cells. This causes the B cells to be killed by the immune system.  * **How does rituximab work?** Rituximab binds to the CD20 protein, which is found on the surface of B cells. This causes the B cells to be labeled for destruction by the immune system.  * **What are the side effects of rituximab?** The most common side effects of rituximab include:  * **Infection:** Rituximab can cause a decrease in the number of B cells, which can lead to an increased risk of infection.  * **Allergic reaction:** Rituximab can cause an allergic reaction, which can be serious.  * **Rash:** Rituximab can cause a rash.  * **Infusion reaction:** Rituximab can cause an infusion reaction, which is a reaction that occurs during or shortly after the infusion of the drug.  I hope this helps! Let me know if you have any other questions. | The serology that needs to be assessed prior to treatment start with rituximab includes:  * **Total B cell count:** This is a measure of the number of B cells in the blood.  * **Antibody levels:** This is a measure of the number of antibodies in the blood.  * **Rubella titer:** This is a measure of the patient's immunity to rubella.  * **Hepatitis B surface antigen:** This is a test for hepatitis B infection.  * **Hepatitis C antibody:** This is a test for hepatitis C infection.  In addition to these serologies, it is also important to assess the patient's overall health, including their vaccination status and any underlying medical conditions.  Here are some of the reasons why these serologies are important to assess prior to treatment with rituximab:  * **Total B cell count:** Rituximab works by targeting and killing B cells. Therefore, it is important to make sure that the patient has a healthy number of B cells before starting treatment.  * **Antibody levels:** Rituximab can also cause a decrease in the number of antibodies. This can lead to an increased risk of infection. Therefore, it is important to make sure that the patient has a healthy number of antibodies before starting treatment.  * **Rubella titer:** Rituximab can cause a decrease in the number of antibodies to rubella. This can lead to an increased risk of contracting rubella. Therefore, it is important to make sure that the patient is immune to rubella before starting treatment.  * **Hepatitis B surface antigen:** Hepatitis B is a serious infection that can be transmitted through blood. Rituximab can cause a decrease in the number of antibodies to hepatitis B. This can lead to an increased risk of contracting hepatitis B. Therefore, it is important to make sure that the patient is not infected with hepatitis B before starting treatment.  * **Hepatitis C antibody:** Hepatitis C is another serious infection that can be transmitted through blood. Rituximab can cause a decrease in the number of antibodies to hepatitis C. This can lead to an increased risk of contracting hepatitis C. Therefore, it is important to make sure that the patient is not infected with hepatitis C before starting treatment.  If the patient's serology is not within normal limits, it may be necessary to delay treatment or adjust the dose of rituximab.  I hope this helps! Let me know if you have any other questions. | Prior to treatment with rituximab, the following serologies should be assessed:  * **Total B-cell count:** This is a measure of the number of B cells in the blood. Rituximab works by targeting and killing B cells, so it is important to make sure that the patient has a healthy number of B cells before starting treatment.  * **Antibody levels:** Rituximab can also cause a decrease in the number of antibodies. This can lead to an increased risk of infection, so it is important to make sure that the patient has a healthy number of antibodies before starting treatment.  * **Rubella titer:** Rituximab can cause a decrease in the number of antibodies to rubella. This can lead to an increased risk of contracting rubella, so it is important to make sure that the patient is immune to rubella before starting treatment.  * **Hepatitis B surface antigen:** Hepatitis B is a serious infection that can be transmitted through blood. Rituximab can cause a decrease in the number of antibodies to hepatitis B. This can lead to an increased risk of contracting hepatitis B, so it is important to make sure that the patient is not infected with hepatitis B before starting treatment.  * **Hepatitis C antibody:** Hepatitis C is another serious infection that can be transmitted through blood. Rituximab can cause a decrease in the number of antibodies to hepatitis C. This can lead to an increased risk of contracting hepatitis C, so it is important to make sure that the patient is not infected with hepatitis C before starting treatment.  In addition to these serologies, it is also important to assess the patient's overall health, including their vaccination status and any underlying medical conditions.  I hope this helps! Let me know if you have any other questions. |
| I7 | A patient undergoing HSCT who develops a disseminated vesicular rash and has not been compliant with his medications is at high risk of transmitting the infection to others. The contact precaution that should be used in this case is contact isolation.  Contact isolation is a type of isolation that is used to prevent the spread of infections that are transmitted through contact with the patient's body fluids or contaminated surfaces. Patients who are placed in contact isolation are required to wear a gown and gloves when they are being cared for, and healthcare workers who are caring for them must also wear gloves and gowns.  The following are the specific contact precautions that should be used for a patient with a disseminated vesicular rash:  The patient should be placed in a private room with a negative pressure airflow.  The patient should wear a gown and gloves when he is being cared for.  Healthcare workers who are caring for the patient should also wear gowns and gloves.  All surfaces in the patient's room should be cleaned and disinfected regularly.  Visitors should be restricted to those who are essential to the patient's care.  Contact isolation should be continued until the rash has resolved and the patient is no longer contagious.  Here are some other important considerations for the care of a patient with a disseminated vesicular rash:  The patient should be instructed to wash his hands frequently.  The patient's personal items should be kept separate from those of other patients.  Any contaminated linens or clothing should be disposed of properly.  By following these precautions, you can help to prevent the spread of infection and keep other patients safe. | A patient undergoing HSCT who develops a disseminated vesicular rash and has not been compliant with his medications is at high risk of transmitting the infection to others. The contact precaution that should be used in this case is strict isolation.  Strict isolation is a type of isolation that is used to prevent the spread of highly contagious infections. Patients who are placed in strict isolation are required to wear a gown, gloves, and a mask when they are being cared for, and healthcare workers who are caring for them must also wear gowns, gloves, and a mask.  The following are the specific contact precautions that should be used for a patient with a disseminated vesicular rash:  The patient should be placed in a private room with a negative pressure airflow.  The patient should wear a gown, gloves, and a mask when he is being cared for.  Healthcare workers who are caring for the patient should also wear gowns, gloves, and a mask.  All surfaces in the patient's room should be cleaned and disinfected regularly.  Visitors should be restricted to those who are essential to the patient's care.  Strict isolation should be continued until the rash has resolved and the patient is no longer contagious.  Here are some other important considerations for the care of a patient with a disseminated vesicular rash:  The patient should be instructed to wash his hands frequently.  The patient's personal items should be kept separate from those of other patients.  Any contaminated linens or clothing should be disposed of properly.  By following these precautions, you can help to prevent the spread of infection and keep other patients safe.  Here are some of the reasons why strict isolation is necessary in this case:  The patient has a disseminated vesicular rash, which is a sign of a highly contagious infection.  The patient has not been compliant with his medications, which means that the infection is likely to be more severe.  The patient is being cared for in an inpatient setting, which means that there is a greater risk of the infection spreading to other patients.  By using strict isolation, you can help to prevent the spread of this infection and keep other patients safe. | The contact precaution that should be used in this case is **strict isolation**.  **Contact isolation** is used to prevent the spread of infections that are transmitted through contact with the patient's body fluids or contaminated surfaces. **Strict isolation** is a more stringent type of contact isolation that is used to prevent the spread of highly contagious infections.  In this case, the patient has a disseminated vesicular rash, which is a sign of a highly contagious infection. The patient has also not been compliant with his medications, which means that the infection is likely to be more severe. Additionally, the patient is being cared for in an inpatient setting, which means that there is a greater risk of the infection spreading to other patients.  Here are the specific contact precautions that should be used for a patient with a disseminated vesicular rash:  * The patient should be placed in a private room with a negative pressure airflow.  * The patient should wear a gown, gloves, and a mask when he is being cared for.  * Healthcare workers who are caring for the patient should also wear gowns, gloves, and a mask.  * All surfaces in the patient's room should be cleaned and disinfected regularly.  * Visitors should be restricted to those who are essential to the patient's care.  Strict isolation should be continued until the rash has resolved and the patient is no longer contagious.  Here are some other important considerations for the care of a patient with a disseminated vesicular rash:  * The patient should be instructed to wash his hands frequently.  * The patient's personal items should be kept separate from those of other patients.  * Any contaminated linens or clothing should be disposed of properly.  By following these precautions, you can help to prevent the spread of infection and keep other patients safe.  Specifically, the rash that the patient is experiencing is a sign of **varicella-zoster virus (VZV)** infection. VZV is a highly contagious virus that causes chickenpox. In people who have had chickenpox, the virus can reactivate and cause shingles. Shingles is a painful rash that can occur anywhere on the body.  In people who are immunocompromised, such as those who are undergoing HSCT, VZV infection can be more severe. It can also lead to complications, such as pneumonia, encephalitis, and death.  That is why it is so important to use strict isolation in this case. By doing so, you can help to prevent the spread of VZV infection to other patients and keep them safe. |
| I8 | The 56-year-old male patient undergoing HSCT who developed fever, dry cough, and shortness of breath with a chest CT scan showing bilateral ground glass pattern and a positive respiratory syncytial virus (RSV) swab is likely suffering from RSV pneumonia.  RSV pneumonia is a serious infection that can be life-threatening in immunocompromised patients, such as those undergoing HSCT. Treatment for RSV pneumonia typically includes supportive care, such as oxygen therapy and fluids, as well as antiviral therapy.  The most commonly used antiviral drug for RSV pneumonia is palivizumab (Synagis). Palivizumab is a monoclonal antibody that works by preventing RSV from attaching to cells in the lungs. Palivizumab is given as an injection every month during the RSV season.  Other antiviral drugs that can be used for RSV pneumonia include ribavirin and ganciclovir. Ribavirin is a nucleoside analog that works by inhibiting the replication of RSV. Ganciclovir is an antiviral drug that works by inhibiting the synthesis of DNA in RSV.  The choice of antiviral drug will depend on the patient's individual circumstances, such as their age, health status, and severity of illness.  In addition to antiviral therapy, patients with RSV pneumonia may also need to be treated with antibiotics if they develop a secondary bacterial infection.  The prognosis for RSV pneumonia in immunocompromised patients is generally good with appropriate treatment. However, the disease can be fatal in some cases.  Here are some other things to keep in mind:  * RSV pneumonia is most common in infants and young children. However, it can also occur in adults, especially those who are immunocompromised.  * The symptoms of RSV pneumonia can include fever, cough, shortness of breath, and wheezing.  * RSV pneumonia is spread through respiratory droplets produced when an infected person coughs or sneezes.  * There is no cure for RSV pneumonia, but it can be treated with supportive care and antiviral therapy.  I hope this helps! Let me know if you have other questions. | The patient is likely suffering from RSV pneumonia, which can be life-threatening in immunocompromised patients. The most commonly used antiviral drug for RSV pneumonia is palivizumab (Synagis). Palivizumab is a monoclonal antibody that works by preventing RSV from attaching to cells in the lungs. Palivizumab is given as an injection every month during the RSV season.  Other antiviral drugs that can be used for RSV pneumonia include ribavirin and ganciclovir. Ribavirin is a nucleoside analog that works by inhibiting the replication of RSV. Ganciclovir is an antiviral drug that works by inhibiting the synthesis of DNA in RSV.  The choice of antiviral drug will depend on the patient's individual circumstances, such as their age, health status, and severity of illness.  In addition to antiviral therapy, patients with RSV pneumonia may also need to be treated with antibiotics if they develop a secondary bacterial infection.  The prognosis for RSV pneumonia in immunocompromised patients is generally good with appropriate treatment. However, the disease can be fatal in some cases.  Here are some other things to keep in mind:  * RSV pneumonia is most common in infants and young children. However, it can also occur in adults, especially those who are immunocompromised.  * The symptoms of RSV pneumonia can include fever, cough, shortness of breath, and wheezing.  * RSV pneumonia is spread through respiratory droplets produced when an infected person coughs or sneezes.  * There is no cure for RSV pneumonia, but it can be treated with supportive care and antiviral therapy.  Based on the information you have provided, the most likely drug that could be offered to this patient is palivizumab (Synagis). Palivizumab is a monoclonal antibody that works by preventing RSV from attaching to cells in the lungs. This drug is typically given as an injection every month during the RSV season.  It is important to note that the choice of drug will ultimately be up to the patient's doctor, who will consider the patient's individual circumstances and medical history.  I hope this helps! Let me know if you have other questions. | The patient is likely suffering from RSV pneumonia, which can be life-threatening in immunocompromised patients. The most commonly used antiviral drug for RSV pneumonia is palivizumab (Synagis). Palivizumab is a monoclonal antibody that works by preventing RSV from attaching to cells in the lungs. Palivizumab is given as an injection every month during the RSV season.  Other antiviral drugs that can be used for RSV pneumonia include ribavirin and ganciclovir. Ribavirin is a nucleoside analog that works by inhibiting the replication of RSV. Ganciclovir is an antiviral drug that works by inhibiting the synthesis of DNA in RSV.  The choice of antiviral drug will depend on the patient's individual circumstances, such as their age, health status, and severity of illness.  In addition to antiviral therapy, patients with RSV pneumonia may also need to be treated with antibiotics if they develop a secondary bacterial infection.  The prognosis for RSV pneumonia in immunocompromised patients is generally good with appropriate treatment. However, the disease can be fatal in some cases.  Here are some other things to keep in mind:  * RSV pneumonia is most common in infants and young children. However, it can also occur in adults, especially those who are immunocompromised.  * The symptoms of RSV pneumonia can include fever, cough, shortness of breath, and wheezing.  * RSV pneumonia is spread through respiratory droplets produced when an infected person coughs or sneezes.  * There is no cure for RSV pneumonia, but it can be treated with supportive care and antiviral therapy.  Based on the information you have provided, the most likely drug that could be offered to this patient is palivizumab (Synagis). Palivizumab is a monoclonal antibody that works by preventing RSV from attaching to cells in the lungs. This drug is typically given as an injection every month during the RSV season.  It is important to note that the choice of drug will ultimately be up to the patient's doctor, who will consider the patient's individual circumstances and medical history.  However, there are some potential concerns with using palivizumab in this patient. For example, palivizumab can cause side effects, such as fever, rash, and wheezing. Additionally, palivizumab is not always effective in preventing RSV pneumonia, especially in patients with severe illness.  If the patient's doctor decides not to use palivizumab, other antiviral drugs, such as ribavirin or ganciclovir, could be used. These drugs are also effective in treating RSV pneumonia, but they can also cause side effects.  The best way to determine which drug is right for the patient is to talk to their doctor. The doctor will consider the patient's individual circumstances and medical history to make the best decision. |
| I9 | Yes, monitoring adenovirus on fecal samples is recommended in adults undergoing allogeneic HSCT. Adenovirus is a common virus that can cause a variety of illnesses, including respiratory infections, gastrointestinal infections, and even death. In adults undergoing allogeneic HSCT, adenovirus infection can be particularly serious, as the patient's immune system is often weakened due to the transplant.  Fecal monitoring is a way to detect adenovirus infection early, before it has a chance to cause serious illness. This allows doctors to start treatment promptly and prevent the infection from becoming life-threatening.  The American Society for Blood and Marrow Transplantation (ASBMT) recommends that all adults undergoing allogeneic HSCT be monitored for adenovirus infection. This includes weekly fecal monitoring for the first 100 days after the transplant, and then monthly monitoring thereafter.  If adenovirus is detected on fecal samples, the patient will be treated with antiviral medications. The specific medication that is used will depend on the patient's individual circumstances.  Early detection and treatment of adenovirus infection is essential to preventing serious illness and death in adults undergoing allogeneic HSCT. Fecal monitoring is a safe and effective way to achieve this goal.  Here are some additional information about adenovirus infection in adults undergoing allogeneic HSCT:  * Adenovirus is a common virus that can cause a variety of illnesses, including respiratory infections, gastrointestinal infections, and even death.  * In adults undergoing allogeneic HSCT, adenovirus infection can be particularly serious, as the patient's immune system is often weakened due to the transplant.  * Fecal monitoring is a way to detect adenovirus infection early, before it has a chance to cause serious illness.  * The American Society for Blood and Marrow Transplantation (ASBMT) recommends that all adults undergoing allogeneic HSCT be monitored for adenovirus infection.  * If adenovirus is detected on fecal samples, the patient will be treated with antiviral medications.  If you are concerned that you or someone you know may have adenovirus infection, it is important to see a doctor right away. | Yes, monitoring adenovirus on fecal samples is recommended in adults undergoing allogeneic HSCT. Adenovirus is a common virus that can cause a variety of illnesses, including respiratory infections, gastrointestinal infections, and even death. In adults undergoing allogeneic HSCT, adenovirus infection can be particularly serious, as the patient's immune system is often weakened due to the transplant.  Fecal monitoring is a way to detect adenovirus infection early, before it has a chance to cause serious illness. This allows doctors to start treatment promptly and prevent the infection from becoming life-threatening.  The American Society for Blood and Marrow Transplantation (ASBMT) recommends that all adults undergoing allogeneic HSCT be monitored for adenovirus infection. This includes weekly fecal monitoring for the first 100 days after the transplant, and then monthly monitoring thereafter.  If adenovirus is detected on fecal samples, the patient will be treated with antiviral medications. The specific medication that is used will depend on the patient's individual circumstances.  Early detection and treatment of adenovirus infection is essential to preventing serious illness and death in adults undergoing allogeneic HSCT. Fecal monitoring is a safe and effective way to achieve this goal.  Here are some additional information about adenovirus infection in adults undergoing allogeneic HSCT:  * Adenovirus is a common virus that can cause a variety of illnesses, including respiratory infections, gastrointestinal infections, and even death.  * In adults undergoing allogeneic HSCT, adenovirus infection can be particularly serious, as the patient's immune system is often weakened due to the transplant.  * Fecal monitoring is a way to detect adenovirus infection early, before it has a chance to cause serious illness.  * The American Society for Blood and Marrow Transplantation (ASBMT) recommends that all adults undergoing allogeneic HSCT be monitored for adenovirus infection.  * If adenovirus is detected on fecal samples, the patient will be treated with antiviral medications.  If you are concerned that you or someone you know may have adenovirus infection, it is important to see a doctor right away. | According to the American Society for Blood and Marrow Transplantation (ASBMT), monitoring adenovirus on fecal samples is **recommended** in adults undergoing allogeneic HSCT. This is because adenovirus infection can be particularly serious in these patients, as their immune system is often weakened due to the transplant.  Fecal monitoring is a way to detect adenovirus infection early, before it has a chance to cause serious illness. This allows doctors to start treatment promptly and prevent the infection from becoming life-threatening.  The ASBMT recommends that all adults undergoing allogeneic HSCT be monitored for adenovirus infection. This includes weekly fecal monitoring for the first 100 days after the transplant, and then monthly monitoring thereafter.  If adenovirus is detected on fecal samples, the patient will be treated with antiviral medications. The specific medication that is used will depend on the patient's individual circumstances.  Early detection and treatment of adenovirus infection is essential to preventing serious illness and death in adults undergoing allogeneic HSCT. Fecal monitoring is a safe and effective way to achieve this goal.  However, it is important to note that the ASBMT's recommendations are just that - recommendations. Ultimately, the decision of whether or not to monitor for adenovirus infection is up to the patient and their doctor.  There are some factors that may make a patient more likely to benefit from fecal monitoring, such as:  * A history of adenovirus infection  * A weakened immune system  * A transplant from a donor who was infected with adenovirus  If you are an adult who is undergoing allogeneic HSCT, you should talk to your doctor about whether or not you should be monitored for adenovirus infection. |
| I10 | The most likely causative agent of hemorrhagic cystitis in a 56-year-old male patient undergoing HSCT who is negative for BK virus is adenovirus. Adenovirus is a common virus that can cause a variety of infections, including upper respiratory tract infections, gastroenteritis, and pneumonia. In immunocompromised patients, adenovirus can also cause hemorrhagic cystitis.  Other viruses that can cause hemorrhagic cystitis in immunocompromised patients include:  * Cytomegalovirus (CMV)  * Epstein-Barr virus (EBV)  * Human herpesvirus 6 (HHV-6)  * Human herpesvirus 7 (HHV-7)  The treatment for hemorrhagic cystitis caused by adenovirus or other viruses is typically supportive care, such as fluids and pain medication. In some cases, antiviral therapy may be used.  The prognosis for hemorrhagic cystitis in immunocompromised patients is generally good with appropriate treatment. However, the disease can be fatal in some cases.  Here are some other things to keep in mind:  * Hemorrhagic cystitis is a serious condition that can cause bleeding in the bladder.  * The symptoms of hemorrhagic cystitis can include blood in the urine, pain in the bladder, and frequent urination.  * Hemorrhagic cystitis is spread through contact with infected urine.  * There is no cure for hemorrhagic cystitis, but it can be treated with supportive care and antiviral therapy.  If you are a patient who is undergoing HSCT and you develop hemorrhagic cystitis, it is important to see a doctor right away. Early diagnosis and treatment can help to improve your chances of recovery. | The most likely causative agent of hemorrhagic cystitis in a 56-year-old male patient undergoing HSCT who is negative for BK virus is adenovirus. Adenovirus is a common virus that can cause a variety of infections, including upper respiratory tract infections, gastroenteritis, and pneumonia. In immunocompromised patients, adenovirus can also cause hemorrhagic cystitis.  Other viruses that can cause hemorrhagic cystitis in immunocompromised patients include:  * **Cytomegalovirus (CMV)**  * **Epstein-Barr virus (EBV)**  * **Human herpesvirus 6 (HHV-6)**  * **Human herpesvirus 7 (HHV-7)**  The treatment for hemorrhagic cystitis caused by adenovirus or other viruses is typically supportive care, such as fluids and pain medication. In some cases, antiviral therapy may be used.  The prognosis for hemorrhagic cystitis in immunocompromised patients is generally good with appropriate treatment. However, the disease can be fatal in some cases.  Here are some other things to keep in mind:  * **Hemorrhagic cystitis is a serious condition that can cause bleeding in the bladder.**  * **The symptoms of hemorrhagic cystitis can include blood in the urine, pain in the bladder, and frequent urination.**  * **Hemorrhagic cystitis is spread through contact with infected urine.**  * **There is no cure for hemorrhagic cystitis, but it can be treated with supportive care and antiviral therapy.**  If you are a patient who is undergoing HSCT and you develop hemorrhagic cystitis, it is important to see a doctor right away. Early diagnosis and treatment can help to improve your chances of recovery.  Here are some additional details about the potential treatments for hemorrhagic cystitis caused by adenovirus or other viruses:  * **Adenovirus:** There is no specific antiviral treatment for adenovirus. However, supportive care, such as fluids and pain medication, can help to relieve symptoms. In some cases, antiviral therapy may be used.  * **CMV:** The treatment for CMV-associated hemorrhagic cystitis is typically ganciclovir or valganciclovir. These drugs can be given intravenously or orally.  * **EBV:** The treatment for EBV-associated hemorrhagic cystitis is typically ganciclovir or valganciclovir. These drugs can be given intravenously or orally.  * **HHV-6:** There is no specific antiviral treatment for HHV-6. However, supportive care, such as fluids and pain medication, can help to relieve symptoms. In some cases, antiviral therapy may be used.  * **HHV-7:** There is no specific antiviral treatment for HHV-7. However, supportive care, such as fluids and pain medication, can help to relieve symptoms. In some cases, antiviral therapy may be used.  The choice of treatment will depend on the specific virus that is causing the hemorrhagic cystitis, as well as the patient's individual circumstances. It is important to talk to your doctor about the best treatment option for you. | The most likely causative agent of hemorrhagic cystitis in a 56-year-old male patient undergoing HSCT who is negative for BK virus is adenovirus. Adenovirus is a common virus that can cause a variety of infections, including upper respiratory tract infections, gastroenteritis, and pneumonia. In immunocompromised patients, adenovirus can also cause hemorrhagic cystitis.  Other viruses that can cause hemorrhagic cystitis in immunocompromised patients include:  * **Cytomegalovirus (CMV)**  * **Epstein-Barr virus (EBV)**  * **Human herpesvirus 6 (HHV-6)**  * **Human herpesvirus 7 (HHV-7)**  The treatment for hemorrhagic cystitis caused by adenovirus or other viruses is typically supportive care, such as fluids and pain medication. In some cases, antiviral therapy may be used.  The prognosis for hemorrhagic cystitis in immunocompromised patients is generally good with appropriate treatment. However, the disease can be fatal in some cases.  Here are some additional details about the potential treatments for hemorrhagic cystitis caused by adenovirus or other viruses:  * **Adenovirus:** There is no specific antiviral treatment for adenovirus. However, supportive care, such as fluids and pain medication, can help to relieve symptoms. In some cases, antiviral therapy may be used.  * **CMV:** The treatment for CMV-associated hemorrhagic cystitis is typically ganciclovir or valganciclovir. These drugs can be given intravenously or orally.  * **EBV:** The treatment for EBV-associated hemorrhagic cystitis is typically ganciclovir or valganciclovir. These drugs can be given intravenously or orally.  * **HHV-6:** There is no specific antiviral treatment for HHV-6. However, supportive care, such as fluids and pain medication, can help to relieve symptoms. In some cases, antiviral therapy may be used.  * **HHV-7:** There is no specific antiviral treatment for HHV-7. However, supportive care, such as fluids and pain medication, can help to relieve symptoms. In some cases, antiviral therapy may be used.  The choice of treatment will depend on the specific virus that is causing the hemorrhagic cystitis, as well as the patient's individual circumstances. It is important to talk to your doctor about the best treatment option for you.  Since the patient's urine analysis is negative for BK virus, the most likely causative agent of hemorrhagic cystitis in this case is adenovirus. The treatment for adenovirus-associated hemorrhagic cystitis is typically supportive care, such as fluids and pain medication. In some cases, antiviral therapy may be used. The specific antiviral drug that is used will depend on the patient's individual circumstances. |
| I11 | Asymptomatic BK viruria is not usually treated in HSCT recipients. BK virus (BKV) is a common virus that can infect the kidneys and bladder. It is usually harmless in healthy people, but it can be more serious in people who have a weakened immune system, such as those who have undergone HSCT.  In most cases, asymptomatic BK viruria will go away on its own without treatment. However, there is a small risk that it could progress to a more serious infection, such as BK virus-associated nephropathy (BKVAN). BKVAN is a rare but serious kidney disease that can lead to kidney failure.  If a patient with HSCT has asymptomatic BK viruria, their doctor will monitor them closely for signs of infection. If the patient develops any symptoms, such as fever, pain in the lower back, or blood in the urine, they will be treated with antiviral medications.  The decision of whether or not to treat asymptomatic BK viruria is a complex one that should be made on a case-by-case basis. Factors that will be considered include the patient's risk of developing BKVAN, their overall health, and their preferences.  Here are some additional information about BK viruria in HSCT recipients:  * BK virus is a common virus that most people are exposed to at some point in their lives.  * BK virus can infect the kidneys and bladder, but it is usually harmless in healthy people.  * In people who have a weakened immune system, such as those who have undergone HSCT, BK virus can be more serious.  * Asymptomatic BK viruria is not usually treated in HSCT recipients.  * If a patient with HSCT develops symptoms of BKVAN, they will be treated with antiviral medications.  If you are concerned that you or someone you know may have BK viruria, it is important to see a doctor right away. | The decision of whether or not to treat asymptomatic BK viruria in HSCT recipients is a complex one that should be made on a case-by-case basis.  Asymptomatic BK viruria refers to the presence of the BK virus in the urine without any associated symptoms. The BK virus is a common virus that most people are exposed to at some point in their lives. It is usually harmless in healthy people, but it can be more serious in people who have a weakened immune system, such as those who have undergone HSCT.  In most cases, asymptomatic BK viruria will go away on its own without treatment. However, there is a small risk that it could progress to a more serious infection, such as BK virus-associated nephropathy (BKVAN). BKVAN is a rare but serious kidney disease that can lead to kidney failure.  If a patient with HSCT has asymptomatic BK viruria, their doctor will monitor them closely for signs of infection. If the patient develops any symptoms, such as fever, pain in the lower back, or blood in the urine, they will be treated with antiviral medications.  There are a number of factors that will be considered when deciding whether or not to treat asymptomatic BK viruria in HSCT recipients, including:  * The patient's risk of developing BKVAN  * The patient's overall health  * The patient's preferences  If you are concerned that you or someone you know may have BK viruria, it is important to see a doctor right away.  Here are some additional information about BK viruria in HSCT recipients:  * BK virus is a common virus that most people are exposed to at some point in their lives.  * BK virus can infect the kidneys and bladder, but it is usually harmless in healthy people.  * In people who have a weakened immune system, such as those who have undergone HSCT, BK virus can be more serious.  * Asymptomatic BK viruria is not usually treated in HSCT recipients.  * If a patient with HSCT develops symptoms of BKVAN, they will be treated with antiviral medications.  I hope this helps! Let me know if you have other requests or questions. | Whether or not asymptomatic BK viruria is treated in HSCT recipients is a complex decision that should be made on a case-by-case basis. There are a number of factors that will be considered, including the patient's risk of developing BK virus-associated nephropathy (BKVAN), their overall health, and their preferences.  In most cases, asymptomatic BK viruria will go away on its own without treatment. However, there is a small risk that it could progress to BKVAN, a rare but serious kidney disease that can lead to kidney failure. If a patient with HSCT develops symptoms of BKVAN, they will be treated with antiviral medications.  Some of the factors that may be considered when deciding whether or not to treat asymptomatic BK viruria in HSCT recipients include:  * The patient's age  * The patient's underlying medical conditions  * The patient's immune status  * The results of previous BK viruria tests  * The patient's preferences  If you are concerned that you or someone you know may have BK viruria, it is important to see a doctor right away.  Here are some additional information about BK viruria in HSCT recipients:  * BK virus is a common virus that most people are exposed to at some point in their lives.  * BK virus can infect the kidneys and bladder, but it is usually harmless in healthy people.  * In people who have a weakened immune system, such as those who have undergone HSCT, BK virus can be more serious.  * Asymptomatic BK viruria is not usually treated in HSCT recipients.  * If a patient with HSCT develops symptoms of BKVAN, they will be treated with antiviral medications.  I hope this helps! Let me know if you have other requests or questions. |
| I12 | Third-party virus-specific T lymphocytes (3rdP-VST) are a type of immunotherapy that is being developed for patients undergoing hematopoietic stem cell transplantation (HSCT). 3rdP-VST are T cells that have been engineered to recognize and kill specific viruses.  3rdP-VST can be used to treat patients who are at risk of developing viral infections after HSCT. These infections can be caused by viruses that the patient is already infected with, or by new viruses that they may be exposed to after the transplant.  3rdP-VST are derived from healthy donors. The T cells are first collected from the donor's blood. Then, they are engineered to recognize and kill a specific virus. This is done by inserting a gene into the T cells that codes for a protein that binds to the virus.  Once the T cells have been engineered, they are expanded in culture. This means that they are multiplied so that there are enough of them to be used for treatment.  3rdP-VST are then infused into the patient's bloodstream. Once they are in the bloodstream, the T cells will start to look for and kill the virus.  3rdP-VST are still under development, but they have the potential to be a very effective treatment for patients undergoing HSCT. They have been shown to be safe and effective in animal studies, and they are currently being evaluated in clinical trials.  Here are some of the benefits of using 3rdP-VST:  * They can be used to treat patients who are infected with viruses that are resistant to other treatments.  * They can be used to prevent viral infections from occurring in the first place.  * They are a relatively new treatment, so they have the potential to be more effective than older treatments.  However, there are also some risks associated with using 3rdP-VST:  * They can cause side effects, such as fever, chills, and fatigue.  * They can be expensive.  * They are not yet available in all countries.  Overall, 3rdP-VST are a promising new treatment for patients undergoing HSCT. They have the potential to be very effective at preventing and treating viral infections, and they are still under development, so they may become even more effective in the future. | Third-party virus-specific T lymphocytes (3rdP-VST) are a type of immunotherapy that is being developed for patients undergoing hematopoietic stem cell transplantation (HSCT). 3rdP-VST are T cells that have been engineered to recognize and kill specific viruses.  3rdP-VST can be used to treat patients who are at risk of developing viral infections after HSCT. These infections can be caused by viruses that the patient is already infected with, or by new viruses that they may be exposed to after the transplant.  3rdP-VST are derived from healthy donors. The T cells are first collected from the donor's blood. Then, they are engineered to recognize and kill a specific virus. This is done by inserting a gene into the T cells that codes for a protein that binds to the virus.  Once the T cells have been engineered, they are expanded in culture. This means that they are multiplied so that there are enough of them to be used for treatment.  3rdP-VST are then infused into the patient's bloodstream. Once they are in the bloodstream, the T cells will start to look for and kill the virus.  3rdP-VST are still under development, but they have the potential to be a very effective treatment for patients undergoing HSCT. They have been shown to be safe and effective in animal studies, and they are currently being evaluated in clinical trials.  Here are some of the benefits of using 3rdP-VST:   - They can be used to treat patients who are infected with viruses that are resistant to other treatments. - They can be used to prevent viral infections from occurring in the first place. - They are a relatively new treatment, so they have the potential to be more effective than older treatments.   However, there are also some risks associated with using 3rdP-VST:   - They can cause side effects, such as fever, chills, and fatigue. - They can be expensive. - They are not yet available in all countries.   Overall, 3rdP-VST are a promising new treatment for patients undergoing HSCT. They have the potential to be very effective at preventing and treating viral infections, and they are still under development, so they may become even more effective in the future.  Here are some additional information about 3rdP-VST:   - What are 3rdP-VST? 3rdP-VST stands for third-party virus-specific T lymphocytes. They are T cells that have been engineered to recognize and kill specific viruses. - How do 3rdP-VST work? 3rdP-VST work by binding to the virus and then killing it. They do this by releasing chemicals that kill the virus or by physically destroying the virus. - What are the benefits of using 3rdP-VST? The benefits of using 3rdP-VST include:   - They can be used to treat patients who are infected with viruses that are resistant to other treatments.   - They can be used to prevent viral infections from occurring in the first place.   - They are a relatively new treatment, so they have the potential to be more effective than older treatments. - What are the risks of using 3rdP-VST? The risks of using 3rdP-VST include:   - They can cause side effects, such as fever, chills, and fatigue.   - They can be expensive.   - They are not yet available in all countries.   I hope this helps! Let me know if you have any other questions. | Third-party virus-specific T lymphocytes (3rdP-VST) are a type of immunotherapy that is being developed for patients undergoing hematopoietic stem cell transplantation (HSCT). 3rdP-VST are T cells that have been engineered to recognize and kill specific viruses.  3rdP-VST can be used to treat patients who are at risk of developing viral infections after HSCT. These infections can be caused by viruses that the patient is already infected with, or by new viruses that they may be exposed to after the transplant.  3rdP-VST are derived from healthy donors. The T cells are first collected from the donor's blood. Then, they are engineered to recognize and kill a specific virus. This is done by inserting a gene into the T cells that codes for a protein that binds to the virus.  Once the T cells have been engineered, they are expanded in culture. This means that they are multiplied so that there are enough of them to be used for treatment.  3rdP-VST are then infused into the patient's bloodstream. Once they are in the bloodstream, the T cells will start to look for and kill the virus.  3rdP-VST are still under development, but they have the potential to be a very effective treatment for patients undergoing HSCT. They have been shown to be safe and effective in animal studies, and they are currently being evaluated in clinical trials.  Here are some of the benefits of using 3rdP-VST:   - They can be used to treat patients who are infected with viruses that are resistant to other treatments. - They can be used to prevent viral infections from occurring in the first place. - They are a relatively new treatment, so they have the potential to be more effective than older treatments.   However, there are also some risks associated with using 3rdP-VST:   - They can cause side effects, such as fever, chills, and fatigue. - They can be expensive. - They are not yet available in all countries.   Overall, 3rdP-VST are a promising new treatment for patients undergoing HSCT. They have the potential to be very effective at preventing and treating viral infections, and they are still under development, so they may become even more effective in the future.  Here are some additional information about 3rdP-VST:   - What are 3rdP-VST? 3rdP-VST stands for third-party virus-specific T lymphocytes. They are T cells that have been engineered to recognize and kill specific viruses. - How do 3rdP-VST work? 3rdP-VST work by binding to the virus and then killing it. They do this by releasing chemicals that kill the virus or by physically destroying the virus. - What are the benefits of using 3rdP-VST? The benefits of using 3rdP-VST include:   - They can be used to treat patients who are infected with viruses that are resistant to other treatments.   - They can be used to prevent viral infections from occurring in the first place.   - They are a relatively new treatment, so they have the potential to be more effective than older treatments. - What are the risks of using 3rdP-VST? The risks of using 3rdP-VST include:   - They can cause side effects, such as fever, chills, and fatigue.   - They can be expensive.   - They are not yet available in all countries.   I hope this helps! Let me know if you have any other questions. |
| I13 | A patient undergoing HSCT who develops fever and X-ray scans showing pneumonia with gram-positive bacteria in blood cultures should avoid vancomycin. Vancomycin is a glycopeptide antibiotic that is used to treat serious infections caused by gram-positive bacteria. However, vancomycin can cause a serious side effect called red man syndrome in patients who are undergoing HSCT. Red man syndrome is a rash that can occur on the face, neck, and upper chest. It can be accompanied by fever, chills, and difficulty breathing.  Other anti-gram-positive intravenous drugs that can be used to treat pneumonia in patients undergoing HSCT include:  * **Teicoplanin:** Teicoplanin is another glycopeptide antibiotic that is similar to vancomycin. However, teicoplanin is less likely to cause red man syndrome.  * **Linezolid:** Linezolid is a newer antibiotic that is effective against a wide range of gram-positive bacteria. It is also less likely to cause red man syndrome than vancomycin.  * **Daptomycin:** Daptomycin is a newer antibiotic that is effective against gram-positive bacteria, including MRSA. It is also less likely to cause red man syndrome than vancomycin.  The choice of antibiotic will depend on the specific bacteria that is causing the pneumonia, as well as the patient's individual circumstances. It is important to talk to your doctor about the best treatment option for you.  Here are some additional things to keep in mind:  * **Pneumonia is a serious condition that can be life-threatening in immunocompromised patients.**  * **The symptoms of pneumonia can include fever, cough, shortness of breath, and chest pain.**  * **Pneumonia is spread through respiratory droplets produced when an infected person coughs or sneezes.**  * **There is no cure for pneumonia, but it can be treated with antibiotics.**  If you are a patient who is undergoing HSCT and you develop pneumonia, it is important to see a doctor right away. Early diagnosis and treatment can help to improve your chances of recovery. | A patient undergoing HSCT who develops fever and X-ray scans showing pneumonia with gram-positive bacteria in blood cultures should avoid vancomycin. Vancomycin is a glycopeptide antibiotic that is used to treat serious infections caused by gram-positive bacteria. However, vancomycin can cause a serious side effect called red man syndrome in patients who are undergoing HSCT. Red man syndrome is a rash that can occur on the face, neck, and upper chest. It can be accompanied by fever, chills, and difficulty breathing.  Other anti-gram-positive intravenous drugs that can be used to treat pneumonia in patients undergoing HSCT include:  * **Teicoplanin:** Teicoplanin is another glycopeptide antibiotic that is similar to vancomycin. However, teicoplanin is less likely to cause red man syndrome.  * **Linezolid:** Linezolid is a newer antibiotic that is effective against a wide range of gram-positive bacteria. It is also less likely to cause red man syndrome than vancomycin.  * **Daptomycin:** Daptomycin is a newer antibiotic that is effective against gram-positive bacteria, including MRSA. It is also less likely to cause red man syndrome than vancomycin.  The choice of antibiotic will depend on the specific bacteria that is causing the pneumonia, as well as the patient's individual circumstances. It is important to talk to a doctor about the best treatment option for you.  Here are some additional things to keep in mind:  * **Pneumonia is a serious condition that can be life-threatening in immunocompromised patients.**  * **The symptoms of pneumonia can include fever, cough, shortness of breath, and chest pain.**  * **Pneumonia is spread through respiratory droplets produced when an infected person coughs or sneezes.**  * **There is no cure for pneumonia, but it can be treated with antibiotics.**  If you are a patient who is undergoing HSCT and you develop pneumonia, it is important to see a doctor right away. Early diagnosis and treatment can help to improve your chances of recovery. | The patient should avoid **vancomycin**, a glycopeptide antibiotic that is used to treat serious infections caused by gram-positive bacteria. However, vancomycin can cause a serious side effect called red man syndrome in patients who are undergoing HSCT. Red man syndrome is a rash that can occur on the face, neck, and upper chest. It can be accompanied by fever, chills, and difficulty breathing.  Other anti-gram-positive intravenous drugs that can be used to treat pneumonia in patients undergoing HSCT include:  * **Teicoplanin:** Teicoplanin is another glycopeptide antibiotic that is similar to vancomycin. However, teicoplanin is less likely to cause red man syndrome.  * **Linezolid:** Linezolid is a newer antibiotic that is effective against a wide range of gram-positive bacteria. It is also less likely to cause red man syndrome than vancomycin.  * **Daptomycin:** Daptomycin is a newer antibiotic that is effective against gram-positive bacteria, including MRSA. It is also less likely to cause red man syndrome than vancomycin.  The choice of antibiotic will depend on the specific bacteria that is causing the pneumonia, as well as the patient's individual circumstances. It is important to talk to a doctor about the best treatment option for you.  Here are some additional things to keep in mind:  * **Pneumonia is a serious condition that can be life-threatening in immunocompromised patients.**  * **The symptoms of pneumonia can include fever, cough, shortness of breath, and chest pain.**  * **Pneumonia is spread through respiratory droplets produced when an infected person coughs or sneezes.**  * **There is no cure for pneumonia, but it can be treated with antibiotics.**  If you are a patient who is undergoing HSCT and you develop pneumonia, it is important to see a doctor right away. Early diagnosis and treatment can help to improve your chances of recovery.  I hope this helps! Let me know if you have other questions. |
| I14 | There are a few possible explanations for why a 56-year-old patient undergoing HSCT who is positive for HHV-6 with a low viral load might be asymptomatic.  * **The patient may have been previously infected with HHV-6 and has developed immunity to the virus.** This is the most likely explanation, as most people are exposed to HHV-6 in childhood and develop lifelong immunity.  * **The patient may have been recently infected with HHV-6, but the virus has not yet caused any symptoms.** This is also possible, as it can take several weeks or even months for HHV-6 to cause symptoms in some people.  * **The patient may have a very low viral load of HHV-6, which is not enough to cause symptoms.** This is less likely, but it is possible.  It is important to note that even if a patient is asymptomatic, they can still transmit HHV-6 to others. Therefore, it is important for patients who are positive for HHV-6 to take steps to prevent the spread of the virus, such as washing their hands frequently and avoiding close contact with people who are immunocompromised.  Here are some additional things to keep in mind:  * **HHV-6 is a common virus that most people are exposed to in childhood.**  * **HHV-6 can cause a variety of symptoms, including fever, rash, and fatigue.**  * **In immunocompromised patients, HHV-6 can cause more serious complications, such as encephalitis and pneumonia.**  * **There is no cure for HHV-6, but most people recover from the infection without any lasting problems.**  If you are a patient who is undergoing HSCT and you are positive for HHV-6, it is important to talk to your doctor about the best way to manage the infection. | There are a few possible explanations for why a 56-year-old patient undergoing HSCT who is positive for HHV-6 with a low viral load might be asymptomatic.  * **The patient may have been previously infected with HHV-6 and has developed immunity to the virus.** This is the most likely explanation, as most people are exposed to HHV-6 in childhood and develop lifelong immunity.  * **The patient may have been recently infected with HHV-6, but the virus has not yet caused any symptoms.** This is also possible, as it can take several weeks or even months for HHV-6 to cause symptoms in some people.  * **The patient may have a very low viral load of HHV-6, which is not enough to cause symptoms.** This is less likely, but it is possible.  It is important to note that even if a patient is asymptomatic, they can still transmit HHV-6 to others. Therefore, it is important for patients who are positive for HHV-6 to take steps to prevent the spread of the virus, such as washing their hands frequently and avoiding close contact with people who are immunocompromised.  Here are some additional things to keep in mind:  * **HHV-6 is a common virus that most people are exposed to in childhood.**  * **HHV-6 can cause a variety of symptoms, including fever, rash, and fatigue.**  * **In immunocompromised patients, HHV-6 can cause more serious complications, such as encephalitis and pneumonia.**  * **There is no cure for HHV-6, but most people recover from the infection without any lasting problems.**  If you are a patient who is undergoing HSCT and you are positive for HHV-6, it is important to talk to your doctor about the best way to manage the infection. | There are a few possible explanations for why a 56-year-old patient undergoing HSCT who is positive for HHV-6 with a low viral load might be asymptomatic.  * **The patient may have been previously infected with HHV-6 and has developed immunity to the virus.** This is the most likely explanation, as most people are exposed to HHV-6 in childhood and develop lifelong immunity.  * **The patient may have been recently infected with HHV-6, but the virus has not yet caused any symptoms.** This is also possible, as it can take several weeks or even months for HHV-6 to cause symptoms in some people.  * **The patient may have a very low viral load of HHV-6, which is not enough to cause symptoms.** This is less likely, but it is possible.  It is important to note that even if a patient is asymptomatic, they can still transmit HHV-6 to others. Therefore, it is important for patients who are positive for HHV-6 to take steps to prevent the spread of the virus, such as washing their hands frequently and avoiding close contact with people who are immunocompromised.  Here are some additional things to keep in mind:  * **HHV-6 is a common virus that most people are exposed to in childhood.**  * **HHV-6 can cause a variety of symptoms, including fever, rash, and fatigue.**  * **In immunocompromised patients, HHV-6 can cause more serious complications, such as encephalitis and pneumonia.**  * **There is no cure for HHV-6, but most people recover from the infection without any lasting problems.**  If you are a patient who is undergoing HSCT and you are positive for HHV-6, it is important to talk to your doctor about the best way to manage the infection.  In addition to the above, here are some other factors that could contribute to the patient being asymptomatic:  * The patient's age. Older people are more likely to be asymptomatic than younger people.  * The patient's overall health. People who are in good health are more likely to be asymptomatic than people who are sick.  * The patient's immune system. People with a strong immune system are more likely to be asymptomatic than people with a weak immune system.  It is important to note that these are just possible explanations, and the exact reason why the patient is asymptomatic cannot be determined without further testing. If you are concerned about your health, please talk to your doctor. |
| I15 | Yes, there is some evidence that rectal swab screening before allogeneic HSCT can help to identify patients who are at risk of developing CMV reactivation. CMV is a common virus that can cause serious infections in patients who are undergoing HSCT. Reactivation of CMV can occur when the virus is reactivated from its latent state. This can happen when the patient's immune system is weakened, such as after HSCT.  Rectal swab screening is a way to test for the presence of CMV in the rectum. This is because CMV can often be found in the rectum of people who are infected with the virus. If CMV is found on a rectal swab, it may mean that the patient is at risk of developing CMV reactivation after HSCT.  There are a few different ways to screen for CMV reactivation. One way is to use a blood test. However, blood tests can sometimes be unreliable, as they may not detect CMV reactivation until it is too late. Rectal swab screening is a more sensitive test, and it can be used to detect CMV reactivation earlier.  If CMV reactivation is detected, the patient may be treated with antiviral medication. This can help to prevent the virus from causing serious infections.  The American Society for Blood and Marrow Transplantation (ASBMT) recommends that rectal swab screening be considered for patients who are undergoing allogeneic HSCT. However, the ASBMT also notes that more research is needed to determine the best way to use rectal swab screening and to identify the patients who are most likely to benefit from it.  Here are some additional information about rectal swab screening:  * Rectal swab screening is a relatively simple and painless procedure.  * The results of rectal swab screening can be available within a few days.  * Rectal swab screening is not always accurate, but it is more sensitive than blood tests.  * If CMV reactivation is detected, the patient may be treated with antiviral medication.  If you are concerned about CMV reactivation, it is important to talk to your doctor. They will be able to discuss your individual situation and recommend the best course of action. | Yes, there is some evidence that rectal swab screening before allogeneic HSCT can help to identify patients who are at risk of developing CMV reactivation. CMV is a common virus that can cause serious infections in patients who are undergoing HSCT. Reactivation of CMV can occur when the virus is reactivated from its latent state. This can happen when the patient's immune system is weakened, such as after HSCT.  Rectal swab screening is a way to test for the presence of CMV in the rectum. This is because CMV can often be found in the rectum of people who are infected with the virus. If CMV is found on a rectal swab, it may mean that the patient is at risk of developing CMV reactivation after HSCT.  There are a few different ways to screen for CMV reactivation. One way is to use a blood test. However, blood tests can sometimes be unreliable, as they may not detect CMV reactivation until it is too late. Rectal swab screening is a more sensitive test, and it can be used to detect CMV reactivation earlier.  If CMV reactivation is detected, the patient may be treated with antiviral medication. This can help to prevent the virus from causing serious infections.  The American Society for Blood and Marrow Transplantation (ASBMT) recommends that rectal swab screening be considered for patients who are undergoing allogeneic HSCT. However, the ASBMT also notes that more research is needed to determine the best way to use rectal swab screening and to identify the patients who are most likely to benefit from it.  Here are some additional information about rectal swab screening:  * Rectal swab screening is a relatively simple and painless procedure.  * The results of rectal swab screening can be available within a few days.  * Rectal swab screening is not always accurate, but it is more sensitive than blood tests.  * If CMV reactivation is detected, the patient may be treated with antiviral medication.  If you are concerned about CMV reactivation, it is important to talk to your doctor. They will be able to discuss your individual situation and recommend the best course of action.  Here are some sources that you may find helpful:  * American Society for Blood and Marrow Transplantation (ASBMT): https://www.asbmt.org/  * National Cancer Institute (NCI): https://www.cancer.gov/  * Johns Hopkins Medicine: https://www.hopkinsmedicine.org/ | Yes, there is some evidence that rectal swab screening before allogeneic HSCT can help to identify patients who are at risk of developing CMV reactivation. CMV is a common virus that can cause serious infections in patients who are undergoing HSCT. Reactivation of CMV can occur when the virus is reactivated from its latent state. This can happen when the patient's immune system is weakened, such as after HSCT.  Rectal swab screening is a way to test for the presence of CMV in the rectum. This is because CMV can often be found in the rectum of people who are infected with the virus. If CMV is found on a rectal swab, it may mean that the patient is at risk of developing CMV reactivation after HSCT.  There are a few different ways to screen for CMV reactivation. One way is to use a blood test. However, blood tests can sometimes be unreliable, as they may not detect CMV reactivation until it is too late. Rectal swab screening is a more sensitive test, and it can be used to detect CMV reactivation earlier.  If CMV reactivation is detected, the patient may be treated with antiviral medication. This can help to prevent the virus from causing serious infections.  The American Society for Blood and Marrow Transplantation (ASBMT) recommends that rectal swab screening be considered for patients who are undergoing allogeneic HSCT. However, the ASBMT also notes that more research is needed to determine the best way to use rectal swab screening and to identify the patients who are most likely to benefit from it.  Here are some additional information about rectal swab screening:  * Rectal swab screening is a relatively simple and painless procedure.  * The results of rectal swab screening can be available within a few days.  * Rectal swab screening is not always accurate, but it is more sensitive than blood tests.  * If CMV reactivation is detected, the patient may be treated with antiviral medication.  If you are concerned about CMV reactivation, it is important to talk to your doctor. They will be able to discuss your individual situation and recommend the best course of action.  Here are some sources that you may find helpful:  * American Society for Blood and Marrow Transplantation (ASBMT): https://www.asbmt.org/  * National Cancer Institute (NCI): https://www.cancer.gov/  * Johns Hopkins Medicine: https://www.hopkinsmedicine.org/  However, it is important to note that not all experts agree on the use of rectal swab screening before allogeneic HSCT. Some experts believe that the benefits of rectal swab screening are not clear, and that the risks of the procedure may outweigh the benefits. More research is needed to determine the best way to use rectal swab screening and to identify the patients who are most likely to benefit from it. |
| I16 | There are a few vaccines that are contraindicated early after allogeneic HSCT. These vaccines include:  * **Live vaccines:** Live vaccines contain a weakened or inactive form of a virus or bacteria. These vaccines can cause an infection in people with a weakened immune system. Live vaccines that are contraindicated early after allogeneic HSCT include measles, mumps, rubella (MMR), varicella (chickenpox), rotavirus, and yellow fever.  * **Inactivated vaccines:** Inactivated vaccines contain a killed or inactivated form of a virus or bacteria. These vaccines are generally safe for people with a weakened immune system, but they may not be as effective as live vaccines. Inactivated vaccines that are contraindicated early after allogeneic HSCT include influenza and pneumococcal vaccines.  It is important to talk to your doctor about which vaccines are safe for you after allogeneic HSCT. Your doctor will be able to consider your individual circumstances and recommend the best course of action.  Here are some additional information about vaccines and allogeneic HSCT:  * **Vaccines:** Vaccines are a way to protect your body from getting sick. They work by exposing your body to a weakened or inactive form of a virus or bacteria. This helps your body develop immunity to the virus or bacteria, so that you are less likely to get sick if you are exposed to it in the future.  * **Allogeneic HSCT:** Allogeneic HSCT is a type of stem cell transplant. In allogeneic HSCT, stem cells are donated from a healthy person. These stem cells are then transplanted into the patient's body. Allogeneic HSCT is used to treat a variety of diseases, including cancer, leukemia, and lymphoma.  * **Immunity:** Immunity is the body's ability to fight off infection. The immune system is made up of a variety of cells and proteins that work together to protect the body from disease.  I hope this helps! | There are a few vaccines that are contraindicated early after allogeneic HSCT. These vaccines include:  * **Live vaccines:** Live vaccines contain a weakened or inactive form of a virus or bacteria. These vaccines can cause an infection in people with a weakened immune system. Live vaccines that are contraindicated early after allogeneic HSCT include:  * **Measles, mumps, and rubella (MMR)**  * **Varicella (chickenpox)**  * **Rotavirus**  * **Yellow fever**  * **Inactivated vaccines:** Inactivated vaccines contain a killed or inactivated form of a virus or bacteria. These vaccines are generally safe for people with a weakened immune system, but they may not be as effective as live vaccines. Inactivated vaccines that are contraindicated early after allogeneic HSCT include:  * **Influenza**  * **Pneumococcal**  It is important to talk to your doctor about which vaccines are safe for you after allogeneic HSCT. Your doctor will be able to consider your individual circumstances and recommend the best course of action.  Here are some additional information about vaccines and allogeneic HSCT:  * **Vaccines:** Vaccines are a way to protect your body from getting sick. They work by exposing your body to a weakened or inactive form of a virus or bacteria. This helps your body develop immunity to the virus or bacteria, so that you are less likely to get sick if you are exposed to it in the future.  * **Allogeneic HSCT:** Allogeneic HSCT is a type of stem cell transplant. In allogeneic HSCT, stem cells are donated from a healthy person. These stem cells are then transplanted into the patient's body. Allogeneic HSCT is used to treat a variety of diseases, including cancer, leukemia, and lymphoma.  * **Immunity:** Immunity is the body's ability to fight off infection. The immune system is made up of a variety of cells and proteins that work together to protect the body from disease.  I hope this helps! | There are a few vaccines that are contraindicated early after allogeneic HSCT. These vaccines include:  * **Live vaccines:** Live vaccines contain a weakened or inactive form of a virus or bacteria. These vaccines can cause an infection in people with a weakened immune system. Live vaccines that are contraindicated early after allogeneic HSCT include:  * **Measles, mumps, and rubella (MMR)**  * **Varicella (chickenpox)**  * **Rotavirus**  * **Yellow fever**  * **Inactivated vaccines:** Inactivated vaccines contain a killed or inactivated form of a virus or bacteria. These vaccines are generally safe for people with a weakened immune system, but they may not be as effective as live vaccines. Inactivated vaccines that are contraindicated early after allogeneic HSCT include:  * **Influenza**  * **Pneumococcal**  It is important to talk to your doctor about which vaccines are safe for you after allogeneic HSCT. Your doctor will be able to consider your individual circumstances and recommend the best course of action.  Here are some additional information about vaccines and allogeneic HSCT:  * **Vaccines:** Vaccines are a way to protect your body from getting sick. They work by exposing your body to a weakened or inactive form of a virus or bacteria. This helps your body develop immunity to the virus or bacteria, so that you are less likely to get sick if you are exposed to it in the future.  * **Allogeneic HSCT:** Allogeneic HSCT is a type of stem cell transplant. In allogeneic HSCT, stem cells are donated from a healthy person. These stem cells are then transplanted into the patient's body. Allogeneic HSCT is used to treat a variety of diseases, including cancer, leukemia, and lymphoma.  * **Immunity:** Immunity is the body's ability to fight off infection. The immune system is made up of a variety of cells and proteins that work together to protect the body from disease.  I hope this helps! |
| I17 | The most probable cause of the skin rash and high-grade fever in a patient who has recently undergone a cord blood transplant and has no evidence of infection is **graft-versus-host disease (GvHD)**. GvHD is a condition that occurs when the donor's immune cells attack the recipient's tissues. This can happen because the donor's immune system is not able to distinguish between the recipient's cells and its own cells.  The pathophysiology of GvHD is complex, but it is thought to involve a number of factors, including the genetic makeup of the donor and recipient, the intensity of the conditioning regimen used before the transplant, and the type of stem cells that are transplanted.  The skin rash in GvHD is often described as a maculopapular rash, which means that it is made up of flat, red spots that are raised above the skin. The rash can be anywhere on the body, but it is most commonly found on the trunk and extremities. The rash can be accompanied by itching, burning, and pain.  High-grade fever is another common symptom of GvHD. The fever is usually caused by the immune system attacking the recipient's tissues. The fever can be accompanied by other symptoms, such as fatigue, nausea, vomiting, and diarrhea.  The treatment for GvHD depends on the severity of the disease. In mild cases, treatment may not be necessary. In more severe cases, treatment may involve the use of immunosuppressant drugs.  If you are concerned that you or someone you know may have GvHD, it is important to see a doctor right away. GvHD can be a serious condition, but it is treatable. | The most probable cause of the skin rash and high-grade fever in a patient who has recently undergone a cord blood transplant and has no evidence of infection is **acute graft-versus-host disease (aGvHD)**. aGvHD is a serious condition that occurs when the donor's immune cells attack the recipient's tissues. This can happen because the donor's immune system is not able to distinguish between the recipient's cells and its own cells.  The pathophysiology of aGvHD is complex, but it is thought to involve a number of factors, including the genetic makeup of the donor and recipient, the intensity of the conditioning regimen used before the transplant, and the type of stem cells that are transplanted.  The skin rash in aGvHD is often described as a maculopapular rash, which means that it is made up of flat, red spots that are raised above the skin. The rash can be anywhere on the body, but it is most commonly found on the trunk and extremities. The rash can be accompanied by itching, burning, and pain.  High-grade fever is another common symptom of aGvHD. The fever is usually caused by the immune system attacking the recipient's tissues. The fever can be accompanied by other symptoms, such as fatigue, nausea, vomiting, and diarrhea.  The blood counts in a patient with aGvHD are often very low. This is because the donor's immune cells are attacking the recipient's bone marrow, which is responsible for producing blood cells. The low blood counts can lead to anemia, leukopenia, and thrombocytopenia.  The treatment for aGvHD depends on the severity of the disease. In mild cases, treatment may not be necessary. In more severe cases, treatment may involve the use of immunosuppressant drugs.  If you are concerned that you or someone you know may have aGvHD, it is important to see a doctor right away. aGvHD can be a serious condition, but it is treatable.  In the case of the patient described in the question, the absence of infectious causes and the low blood counts make aGvHD the most likely diagnosis. The skin rash and fever are likely caused by the donor's immune cells attacking the recipient's tissues. The treatment for aGvHD in this case would likely involve the use of immunosuppressant drugs. | The most probable cause of the skin rash and high-grade fever in a patient who has recently undergone a cord blood transplant and has no evidence of infection is **acute graft-versus-host disease (aGvHD)**.  Acute graft-versus-host disease (aGvHD) is a serious condition that occurs when the donor's immune cells attack the recipient's tissues. This can happen because the donor's immune system is not able to distinguish between the recipient's cells and its own cells.  The pathophysiology of aGvHD is complex, but it is thought to involve a number of factors, including the genetic makeup of the donor and recipient, the intensity of the conditioning regimen used before the transplant, and the type of stem cells that are transplanted.  The skin rash in aGvHD is often described as a maculopapular rash, which means that it is made up of flat, red spots that are raised above the skin. The rash can be anywhere on the body, but it is most commonly found on the trunk and extremities. The rash can be accompanied by itching, burning, and pain.  High-grade fever is another common symptom of aGvHD. The fever is usually caused by the immune system attacking the recipient's tissues. The fever can be accompanied by other symptoms, such as fatigue, nausea, vomiting, and diarrhea.  The blood counts in a patient with aGvHD are often very low. This is because the donor's immune cells are attacking the recipient's bone marrow, which is responsible for producing blood cells. The low blood counts can lead to anemia, leukopenia, and thrombocytopenia.  The treatment for aGvHD depends on the severity of the disease. In mild cases, treatment may not be necessary. In more severe cases, treatment may involve the use of immunosuppressant drugs.  If you are concerned that you or someone you know may have aGvHD, it is important to see a doctor right away. aGvHD can be a serious condition, but it is treatable.  In the case of the patient described in the question, the absence of infectious causes and the low blood counts make aGvHD the most likely diagnosis. The skin rash and fever are likely caused by the donor's immune cells attacking the recipient's tissues. The treatment for aGvHD in this case would likely involve the use of immunosuppressant drugs.  Here are some additional information about aGvHD:  * **Symptoms:** The symptoms of aGvHD can vary depending on the severity of the disease. Mild cases may only cause a mild skin rash and fatigue. More severe cases can cause a severe skin rash, high fever, nausea, vomiting, diarrhea, and organ damage.  * **Diagnosis:** The diagnosis of aGvHD is made based on the patient's symptoms, blood tests, and a biopsy of the skin.  * **Treatment:** The treatment for aGvHD depends on the severity of the disease. Mild cases may only require supportive care, such as fluids and antibiotics. More severe cases may require the use of immunosuppressant drugs.  * **Prognosis:** The prognosis for aGvHD depends on the severity of the disease and the patient's response to treatment. Mild cases have a good prognosis, while more severe cases can be fatal.  I hope this helps! |
| I18 | A patient who develops multiple lymphadenopathy a few weeks after receiving HSCT and has EBV positive on peripheral blood with high viral load is at risk of developing post-transplant lymphoproliferative disorder (PTLD). PTLD is a type of cancer that can occur in patients who have undergone HSCT. It is caused by the activation of EBV-infected B cells.  The treatment of PTLD depends on the stage of the disease. In early-stage PTLD, treatment may involve the use of antiviral drugs, such as ganciclovir or foscarnet. In more advanced stages of PTLD, treatment may involve the use of chemotherapy or immunotherapy.  One drug that may be offered to the patient is rituximab. Rituximab is a monoclonal antibody that targets B cells. It is used to treat a variety of conditions, including PTLD. Rituximab is thought to work by depleting the body of B cells that are infected with EBV.  Other drugs that may be used to treat PTLD include:   - Chemotherapy: Chemotherapy drugs are used to kill cancer cells. They can be used alone or in combination with other treatments. - Immunotherapy: Immunotherapy drugs work to boost the body's immune system. They can be used to treat PTLD that is not responding to other treatments. - Surgery: Surgery may be used to remove tumors that are causing symptoms.   The choice of treatment for PTLD depends on the stage of the disease, the patient's overall health, and other factors.  Here are some additional information about PTLD:   - What is PTLD? PTLD stands for post-transplant lymphoproliferative disorder. It is a type of cancer that can occur in patients who have undergone HSCT. - How does PTLD develop? PTLD develops when EBV-infected B cells are activated. EBV is a virus that can live in the body without causing any problems. However, in patients who have undergone HSCT, EBV can be reactivated and can cause PTLD. - What are the symptoms of PTLD? The symptoms of PTLD can vary, but they often include fever, fatigue, weight loss, and lymphadenopathy. - How is PTLD diagnosed? PTLD is diagnosed by a blood test that looks for EBV DNA. A biopsy of the lymph nodes may also be done to confirm the diagnosis. - What is the treatment for PTLD? The treatment for PTLD depends on the stage of the disease. In early-stage PTLD, treatment may involve the use of antiviral drugs. In more advanced stages of PTLD, treatment may involve the use of chemotherapy or immunotherapy.   I hope this helps! Let me know if you have any other questions. | The patient is at risk of developing post-transplant lymphoproliferative disorder (PTLD), which is a type of cancer that can occur in patients who have undergone HSCT. PTLD is caused by the activation of EBV-infected B cells.  One drug that may be offered to the patient is rituximab. Rituximab is a monoclonal antibody that targets B cells. It is used to treat a variety of conditions, including PTLD. Rituximab is thought to work by depleting the body of B cells that are infected with EBV.  Other drugs that may be used to treat PTLD include:  * **Chemotherapy:** Chemotherapy drugs are used to kill cancer cells. They can be used alone or in combination with other treatments.  * **Immunotherapy:** Immunotherapy drugs work to boost the body's immune system. They can be used to treat PTLD that is not responding to other treatments.  * **Surgery:** Surgery may be used to remove tumors that are causing symptoms.  The choice of treatment for PTLD depends on the stage of the disease, the patient's overall health, and other factors.  In the case of the patient who has developed multiple lymphadenopathy a few weeks after receiving HSCT and has EBV positive on peripheral blood with high viral load, rituximab is likely to be offered as a first-line treatment. This is because rituximab is a well-established and effective treatment for PTLD. It is also relatively well-tolerated by patients.  If the patient does not respond to rituximab, other treatments, such as chemotherapy or immunotherapy, may be considered.  I hope this helps! Let me know if you have any other questions. | The patient is at risk of developing post-transplant lymphoproliferative disorder (PTLD), which is a type of cancer that can occur in patients who have undergone HSCT. PTLD is caused by the activation of EBV-infected B cells.  The most likely drug that will be offered to the patient is rituximab. Rituximab is a monoclonal antibody that targets B cells. It is used to treat a variety of conditions, including PTLD. Rituximab is thought to work by depleting the body of B cells that are infected with EBV.  Other drugs that may be used to treat PTLD include:  * **Chemotherapy:** Chemotherapy drugs are used to kill cancer cells. They can be used alone or in combination with other treatments.  * **Immunotherapy:** Immunotherapy drugs work to boost the body's immune system. They can be used to treat PTLD that is not responding to other treatments.  * **Surgery:** Surgery may be used to remove tumors that are causing symptoms.  The choice of treatment for PTLD depends on the stage of the disease, the patient's overall health, and other factors.  In the case of the patient who has developed multiple lymphadenopathy a few weeks after receiving HSCT and has EBV positive on peripheral blood with high viral load, rituximab is likely to be offered as a first-line treatment. This is because rituximab is a well-established and effective treatment for PTLD. It is also relatively well-tolerated by patients.  If the patient does not respond to rituximab, other treatments, such as chemotherapy or immunotherapy, may be considered.  Here are some additional information about PTLD:  * **What is PTLD?** PTLD stands for post-transplant lymphoproliferative disorder. It is a type of cancer that can occur in patients who have undergone HSCT.  * **How does PTLD develop?** PTLD develops when EBV-infected B cells are activated. EBV is a virus that can live in the body without causing any problems. However, in patients who have undergone HSCT, EBV can be reactivated and can cause PTLD.  * **What are the symptoms of PTLD?** The symptoms of PTLD can vary, but they often include fever, fatigue, weight loss, and lymphadenopathy.  * **How is PTLD diagnosed?** PTLD is diagnosed by a blood test that looks for EBV DNA. A biopsy of the lymph nodes may also be done to confirm the diagnosis.  * **What is the treatment for PTLD?** The treatment for PTLD depends on the stage of the disease. In early-stage PTLD, treatment may involve the use of antiviral drugs. In more advanced stages of PTLD, treatment may involve the use of chemotherapy or immunotherapy.  I hope this helps! Let me know if you have any other questions. |
| **Section 4 – Noninfectious complications** | | | |
| C1 | Acute graft-versus-host disease (aGvHD) is a serious condition that occurs when the donor's immune cells attack the recipient's tissues. This can happen because the donor's immune system is not able to distinguish between the recipient's cells and its own cells.  The clinical features of aGvHD can vary depending on the severity of the disease. Mild cases may only cause a mild skin rash and fatigue. More severe cases can cause a severe skin rash, high fever, nausea, vomiting, diarrhea, and organ damage.  The most common clinical features of aGvHD are:  * **Skin rash:** The skin rash in aGvHD is often described as a maculopapular rash, which means that it is made up of flat, red spots that are raised above the skin. The rash can be anywhere on the body, but it is most commonly found on the trunk and extremities. The rash can be accompanied by itching, burning, and pain.  * **High-grade fever:** Another common symptom of aGvHD is high-grade fever. The fever is usually caused by the immune system attacking the recipient's tissues. The fever can be accompanied by other symptoms, such as fatigue, nausea, vomiting, and diarrhea.  * **Low blood counts:** The blood counts in a patient with aGvHD are often very low. This is because the donor's immune cells are attacking the recipient's bone marrow, which is responsible for producing blood cells. The low blood counts can lead to anemia, leukopenia, and thrombocytopenia.  * **Gastrointestinal symptoms:** Gastrointestinal symptoms, such as nausea, vomiting, and diarrhea, are also common in patients with aGvHD. These symptoms can be caused by the donor's immune cells attacking the lining of the gut.  * **Liver involvement:** Liver involvement is another common feature of aGvHD. This can manifest as jaundice, abdominal pain, and liver function test abnormalities.  * **Other organ involvement:** Other organs that can be involved in aGvHD include the lungs, heart, and kidneys. The symptoms of organ involvement will vary depending on the organ that is affected.  The diagnosis of aGvHD is made based on the patient's symptoms, blood tests, and a biopsy of the skin. The treatment for aGvHD depends on the severity of the disease. Mild cases may only require supportive care, such as fluids and antibiotics. More severe cases may require the use of immunosuppressant drugs.  The prognosis for aGvHD depends on the severity of the disease and the patient's response to treatment. Mild cases have a good prognosis, while more severe cases can be fatal.  I hope this helps! | Acute Graft-versus-Host Disease (aGvHD) is a reaction of the donor's immune system to the recipient's tissues. It can occur after a stem cell transplant, where stem cells are donated from a healthy person to a person with a serious disease. The donor's immune cells attack the recipient's tissues, causing inflammation and damage.  The clinical features of aGvHD can vary depending on the severity of the disease. Mild cases may only cause a mild skin rash and fatigue. More severe cases can cause a severe skin rash, high fever, nausea, vomiting, diarrhea, and organ damage.  The most common clinical features of aGvHD are:  * **Skin rash:** The skin rash in aGvHD is often described as a maculopapular rash, which means that it is made up of flat, red spots that are raised above the skin. The rash can be anywhere on the body, but it is most commonly found on the trunk and extremities. The rash can be accompanied by itching, burning, and pain.  * **High-grade fever:** Another common symptom of aGvHD is high-grade fever. The fever is usually caused by the immune system attacking the recipient's tissues. The fever can be accompanied by other symptoms, such as fatigue, nausea, vomiting, and diarrhea.  * **Low blood counts:** The blood counts in a patient with aGvHD are often very low. This is because the donor's immune cells are attacking the recipient's bone marrow, which is responsible for producing blood cells. The low blood counts can lead to anemia, leukopenia, and thrombocytopenia.  * **Gastrointestinal symptoms:** Gastrointestinal symptoms, such as nausea, vomiting, and diarrhea, are also common in patients with aGvHD. These symptoms can be caused by the donor's immune cells attacking the lining of the gut.  * **Liver involvement:** Liver involvement is another common feature of aGvHD. This can manifest as jaundice, abdominal pain, and liver function test abnormalities.  * **Other organ involvement:** Other organs that can be involved in aGvHD include the lungs, heart, and kidneys. The symptoms of organ involvement will vary depending on the organ that is affected.  The diagnosis of aGvHD is made based on the patient's symptoms, blood tests, and a biopsy of the skin. The treatment for aGvHD depends on the severity of the disease. Mild cases may only require supportive care, such as fluids and antibiotics. More severe cases may require the use of immunosuppressant drugs.  The prognosis for aGvHD depends on the severity of the disease and the patient's response to treatment. Mild cases have a good prognosis, while more severe cases can be fatal.  Here are some additional information about aGvHD:  * **Symptoms:** The symptoms of aGvHD can vary depending on the severity of the disease. Mild cases may only cause a mild skin rash and fatigue. More severe cases can cause a severe skin rash, high fever, nausea, vomiting, diarrhea, and organ damage.  * **Diagnosis:** The diagnosis of aGvHD is made based on the patient's symptoms, blood tests, and a biopsy of the skin.  * **Treatment:** The treatment for aGvHD depends on the severity of the disease. Mild cases may only require supportive care, such as fluids and antibiotics. More severe cases may require the use of immunosuppressant drugs.  * **Prognosis:** The prognosis for aGvHD depends on the severity of the disease and the patient's response to treatment. Mild cases have a good prognosis, while more severe cases can be fatal.  If you are concerned that you or someone you know may have aGvHD, it is important to see a doctor right away. aGvHD can be a serious condition, but it is treatable. | Acute Graft-versus-Host Disease (aGvHD) is a reaction of the donor's immune system to the recipient's tissues. It can occur after a stem cell transplant, where stem cells are donated from a healthy person to a person with a serious disease. The donor's immune cells attack the recipient's tissues, causing inflammation and damage.  The clinical features of aGvHD can vary depending on the severity of the disease. Mild cases may only cause a mild skin rash and fatigue. More severe cases can cause a severe skin rash, high fever, nausea, vomiting, diarrhea, and organ damage.  The most common clinical features of aGvHD are:  * **Skin rash:** The skin rash in aGvHD is often described as a **maculopapular rash**, which means that it is made up of flat, red spots that are raised above the skin. The rash can be anywhere on the body, but it is most commonly found on the trunk and extremities. The rash can be accompanied by itching, burning, and pain.  * **High-grade fever:** Another common symptom of aGvHD is **high-grade fever**. The fever is usually caused by the immune system attacking the recipient's tissues. The fever can be accompanied by other symptoms, such as fatigue, nausea, vomiting, and diarrhea.  * **Low blood counts:** The blood counts in a patient with aGvHD are often very low. This is because the donor's immune cells are attacking the recipient's bone marrow, which is responsible for producing blood cells. The low blood counts can lead to **anemia**, **leukopenia**, and **thrombocytopenia**.  * **Gastrointestinal symptoms:** Gastrointestinal symptoms, such as **nausea**, **vomiting**, and **diarrhea**, are also common in patients with aGvHD. These symptoms can be caused by the donor's immune cells attacking the lining of the gut.  * **Liver involvement:** Liver involvement is another common feature of aGvHD. This can manifest as **jaundice**, abdominal pain, and liver function test abnormalities.  * **Other organ involvement:** Other organs that can be involved in aGvHD include the lungs, heart, and kidneys. The symptoms of organ involvement will vary depending on the organ that is affected.  The diagnosis of aGvHD is made based on the patient's symptoms, blood tests, and a biopsy of the skin. The treatment for aGvHD depends on the severity of the disease. Mild cases may only require supportive care, such as fluids and antibiotics. More severe cases may require the use of immunosuppressant drugs.  The prognosis for aGvHD depends on the severity of the disease and the patient's response to treatment. Mild cases have a good prognosis, while more severe cases can be fatal.  Here are some additional information about aGvHD:  * **Symptoms:** The symptoms of aGvHD can vary depending on the severity of the disease. Mild cases may only cause a mild skin rash and fatigue. More severe cases can cause a severe skin rash, high fever, nausea, vomiting, diarrhea, and organ damage.  * **Diagnosis:** The diagnosis of aGvHD is made based on the patient's symptoms, blood tests, and a biopsy of the skin.  * **Treatment:** The treatment for aGvHD depends on the severity of the disease. Mild cases may only require supportive care, such as fluids and antibiotics. More severe cases may require the use of immunosuppressant drugs.  * **Prognosis:** The prognosis for aGvHD depends on the severity of the disease and the patient's response to treatment. Mild cases have a good prognosis, while more severe cases can be fatal.  If you are concerned that you or someone you know may have aGvHD, it is important to see a doctor right away. aGvHD can be a serious condition, but it is treatable. |
| C2 | I'm not able to help with that, as I'm only a language model. | The most likely diagnosis in this case is **neutropenic enterocolitis** (NEC), also known as typhlitis. NEC is a serious inflammation of the bowel that can occur in people who have a low number of white blood cells (neutrophils). This is a common complication after HSCT, as the high-dose chemotherapy and radiation therapy used to prepare for the transplant can damage the bone marrow and suppress the production of neutrophils.  The symptoms of NEC can include diarrhea, abdominal pain, fever, and vomiting. In some cases, the inflammation can progress to perforation of the bowel, which is a life-threatening condition.  The first line treatment for NEC is antibiotics. In some cases, surgery may be necessary to remove the affected part of the bowel.  In the absence of lab abnormalities and a normal physical exam, the other possible diagnoses in this case are **viral enteritis** and **graft-versus-host disease** (GVHD). However, these diagnoses are less likely than NEC, given the patient's presentation.  Viral enteritis is an infection of the bowel that can be caused by a variety of viruses, including rotavirus, adenovirus, and cytomegalovirus (CMV). GVHD is a condition in which the donor immune cells attack the recipient's tissues. Both viral enteritis and GVHD can cause diarrhea, but they are typically accompanied by other symptoms, such as fever, vomiting, and weight loss.  The first line treatment for viral enteritis is supportive care, such as fluid replacement and electrolyte management. In some cases, antiviral medications may be necessary.  The first line treatment for GVHD of the gut is immunosuppression with steroids and other medications. In some cases, surgery may be necessary to remove the affected part of the bowel.  The best way to diagnose NEC is with a colonoscopy or CT scan of the abdomen. These tests can show inflammation and/or perforation of the bowel.  If you are concerned that you or someone you know may have NEC, it is important to see a doctor right away. NEC is a serious condition, but it can be treated successfully if it is caught early. | The most likely diagnosis in this case is **neutropenic enterocolitis** (NEC), also known as typhlitis. NEC is a serious inflammation of the bowel that can occur in people who have a low number of white blood cells (neutrophils). This is a common complication after HSCT, as the high-dose chemotherapy and radiation therapy used to prepare for the transplant can damage the bone marrow and suppress the production of neutrophils.  The symptoms of NEC can include diarrhea, abdominal pain, fever, and vomiting. In some cases, the inflammation can progress to perforation of the bowel, which is a life-threatening condition.  The first line treatment for NEC is antibiotics. In some cases, surgery may be necessary to remove the affected part of the bowel.  In the absence of lab abnormalities and a normal physical exam, the other possible diagnoses in this case are **viral enteritis** and **graft-versus-host disease** (GVHD). However, these diagnoses are less likely than NEC, given the patient's presentation.  Viral enteritis is an infection of the bowel that can be caused by a variety of viruses, including rotavirus, adenovirus, and cytomegalovirus (CMV). GVHD is a condition in which the donor immune cells attack the recipient's tissues. Both viral enteritis and GVHD can cause diarrhea, but they are typically accompanied by other symptoms, such as fever, vomiting, and weight loss.  The first line treatment for viral enteritis is supportive care, such as fluid replacement and electrolyte management. In some cases, antiviral medications may be necessary.  The first line treatment for GVHD of the gut is immunosuppression with steroids and other medications. In some cases, surgery may be necessary to remove the affected part of the bowel.  The best way to diagnose NEC is with a colonoscopy or CT scan of the abdomen. These tests can show inflammation and/or perforation of the bowel.  If you are concerned that you or someone you know may have NEC, it is important to see a doctor right away. NEC is a serious condition, but it can be treated successfully if it is caught early.  However, given the fact that there are no lab abnormalities or physical exam findings to suggest an alternative diagnosis, the most likely diagnosis in this case is NEC. The first line treatment for NEC is antibiotics, so the doctor would likely start the patient on a course of antibiotics and monitor their condition closely. If the patient's condition does not improve with antibiotics, or if they develop other symptoms, such as fever or vomiting, the doctor may recommend further testing, such as a colonoscopy or CT scan of the abdomen. |
| C3 | The success rate of systemic steroids in acute graft-versus-host disease (GVHD) is about 35% to 60%. This means that about 35% to 60% of patients who receive systemic steroids will have a complete or partial remission of their GVHD.  Systemic steroids are the first-line treatment for acute GVHD. They work by suppressing the immune system, which helps to reduce the inflammation and damage caused by GVHD. However, steroids can also have side effects, such as weight gain, mood changes, and increased risk of infection.  If systemic steroids are not effective, other treatments may be used, such as cyclosporine, tacrolimus, or mycophenolate mofetil. These drugs also work by suppressing the immune system, but they have different side effects than steroids.  The prognosis for patients with acute GVHD depends on the severity of the disease. Patients with mild GVHD have a good prognosis, while patients with severe GVHD have a worse prognosis.  Here are some additional information about systemic steroids in the treatment of acute GVHD:  * Systemic steroids are usually given orally, but they can also be given intravenously.  * The dose of systemic steroids is usually gradually decreased over time.  * Patients who take systemic steroids should be monitored closely for side effects.  I hope this information is helpful. Please let me know if you have any other questions. | The success rate of systemic steroids in acute graft-versus-host disease (GVHD) is about 35% to 60%. This means that about 35% to 60% of patients who receive systemic steroids will have a complete or partial remission of their GVHD.  Systemic steroids are the first-line treatment for acute GVHD. They work by suppressing the immune system, which helps to reduce the inflammation and damage caused by GVHD. However, steroids can also have side effects, such as weight gain, mood changes, and increased risk of infection.  If systemic steroids are not effective, other treatments may be used, such as cyclosporine, tacrolimus, or mycophenolate mofetil. These drugs also work by suppressing the immune system, but they have different side effects than steroids.  The prognosis for patients with acute GVHD depends on the severity of the disease. Patients with mild GVHD have a good prognosis, while patients with severe GVHD have a worse prognosis.  Here are some additional information about systemic steroids in the treatment of acute GVHD:  * Systemic steroids are usually given orally, but they can also be given intravenously.  * The dose of systemic steroids is usually gradually decreased over time.  * Patients who take systemic steroids should be monitored closely for side effects.  It is important to note that the success rate of systemic steroids in acute GVHD can vary depending on the patient's individual factors, such as the severity of their GVHD and their overall health status. Additionally, the success rate of systemic steroids may also improve with the development of newer treatment options.  I hope this information is helpful. Please let me know if you have any other questions. | The success rate of systemic steroids in acute graft-versus-host disease (GVHD) is about 35% to 60%. This means that about 35% to 60% of patients who receive systemic steroids will have a complete or partial remission of their GVHD. However, it is important to note that the success rate can vary depending on the patient's individual factors, such as the severity of their GVHD and their overall health status.  Systemic steroids are the first-line treatment for acute GVHD. They work by suppressing the immune system, which helps to reduce the inflammation and damage caused by GVHD. However, steroids can also have side effects, such as weight gain, mood changes, and increased risk of infection.  If systemic steroids are not effective, other treatments may be used, such as cyclosporine, tacrolimus, or mycophenolate mofetil. These drugs also work by suppressing the immune system, but they have different side effects than steroids.  The prognosis for patients with acute GVHD depends on the severity of the disease. Patients with mild GVHD have a good prognosis, while patients with severe GVHD have a worse prognosis.  Here are some additional information about systemic steroids in the treatment of acute GVHD:  * Systemic steroids are usually given orally, but they can also be given intravenously.  * The dose of systemic steroids is usually gradually decreased over time.  * Patients who take systemic steroids should be monitored closely for side effects.  It is important to note that the success rate of systemic steroids in acute GVHD can vary depending on the patient's individual factors, such as the severity of their GVHD and their overall health status. Additionally, the success rate of systemic steroids may also improve with the development of newer treatment options.  I hope this information is helpful. Please let me know if you have any other questions. |
| C4 | Granulocyte transfusions are not a standard of care during aplasia in a patient undergoing HSCT. However, they may be used in certain cases, such as when the patient has a severe infection that is not responding to antibiotics.  Granulocyte transfusions are a type of blood transfusion that delivers white blood cells (WBCs) to the patient. WBCs are important for fighting infection, so granulocyte transfusions can help to clear an infection in a patient who has a low WBC count.  However, granulocyte transfusions are not without risks. They can cause allergic reactions, and they can also increase the risk of graft-versus-host disease (GVHD). GVHD is a condition in which the donor's immune cells attack the recipient's tissues.  For these reasons, granulocyte transfusions are not used routinely in patients undergoing HSCT. They are only used in cases where the benefits outweigh the risks.  Here are some of the factors that doctors consider when deciding whether or not to use granulocyte transfusions in a patient undergoing HSCT:  * The severity of the infection  * The patient's WBC count  * The patient's risk of GVHD  * The availability of other treatment options  If you are undergoing HSCT and you develop a severe infection, your doctor will discuss with you whether or not granulocyte transfusions are right for you. | No, granulocyte transfusions are not a standard of care during aplasia in a patient undergoing HSCT. However, they may be used in certain cases, such as when the patient has a severe infection that is not responding to antibiotics.  Granulocyte transfusions are a type of blood transfusion that delivers white blood cells (WBCs) to the patient. WBCs are important for fighting infection, so granulocyte transfusions can help to clear an infection in a patient who has a low WBC count.  However, granulocyte transfusions are not without risks. They can cause allergic reactions, and they can also increase the risk of graft-versus-host disease (GVHD). GVHD is a condition in which the donor's immune cells attack the recipient's tissues.  For these reasons, granulocyte transfusions are not used routinely in patients undergoing HSCT. They are only used in cases where the benefits outweigh the risks.  Here are some of the factors that doctors consider when deciding whether or not to use granulocyte transfusions in a patient undergoing HSCT:  * The severity of the infection  * The patient's WBC count  * The patient's risk of GVHD  * The availability of other treatment options  If you are undergoing HSCT and you develop a severe infection, your doctor will discuss with you whether or not granulocyte transfusions are right for you.  According to a 2019 study published in the journal *Blood*, granulocyte transfusions were only used in 10% of patients who developed a severe infection during aplasia after HSCT. The study also found that granulocyte transfusions did not improve the survival rate of patients with severe infections.  Based on this evidence, it is clear that granulocyte transfusions are not a standard of care during aplasia in a patient undergoing HSCT. They should only be used in cases where the benefits outweigh the risks. | No, granulocyte transfusions are not a standard of care during aplasia in a patient undergoing HSCT. However, they may be used in certain cases, such as when the patient has a severe infection that is not responding to antibiotics.  Granulocyte transfusions are a type of blood transfusion that delivers white blood cells (WBCs) to the patient. WBCs are important for fighting infection, so granulocyte transfusions can help to clear an infection in a patient who has a low WBC count.  However, granulocyte transfusions are not without risks. They can cause allergic reactions, and they can also increase the risk of graft-versus-host disease (GVHD). GVHD is a condition in which the donor's immune cells attack the recipient's tissues.  For these reasons, granulocyte transfusions are not used routinely in patients undergoing HSCT. They are only used in cases where the benefits outweigh the risks.  Here are some of the factors that doctors consider when deciding whether or not to use granulocyte transfusions in a patient undergoing HSCT:  * The severity of the infection  * The patient's WBC count  * The patient's risk of GVHD  * The availability of other treatment options  If you are undergoing HSCT and you develop a severe infection, your doctor will discuss with you whether or not granulocyte transfusions are right for you.  According to a 2019 study published in the journal *Blood*, granulocyte transfusions were only used in 10% of patients who developed a severe infection during aplasia after HSCT. The study also found that granulocyte transfusions did not improve the survival rate of patients with severe infections.  Based on this evidence, it is clear that granulocyte transfusions are not a standard of care during aplasia in a patient undergoing HSCT. They should only be used in cases where the benefits outweigh the risks.  However, there is some evidence that granulocyte transfusions may be helpful in patients with certain types of infections, such as those caused by bacteria that are resistant to antibiotics. More research is needed to determine the exact role of granulocyte transfusions in the treatment of infections after HSCT. |
| C5 | The BEDI grade for a patient suffering from hemorrhagic cystitis with hematuria and visible clots is **Grade III**.  The BEDI grading system is a clinical scoring system used to assess the severity of hemorrhagic cystitis. It is a four-tiered system, with Grade I being the mildest and Grade IV being the most severe.  The BEDI grading system is based on the following criteria:  * **B**leeding: The amount of bleeding, as assessed by the patient's report of hematuria and the presence of visible clots.  * **E**xtent: The extent of bleeding, as assessed by the presence of bleeding in the bladder and/or urethra.  * **D**ysuria: The presence of dysuria, or painful urination.  * **I**nfection: The presence of infection, as assessed by the presence of fever, chills, or other signs of infection.  A patient with hemorrhagic cystitis and hematuria and visible clots would be scored as Grade III on the BEDI grading system. This is because the patient has moderate bleeding, with visible clots, and dysuria. The patient does not have infection, so they would not be scored higher than Grade III.  The BEDI grading system is a useful tool for assessing the severity of hemorrhagic cystitis and guiding treatment. Patients with Grade III hemorrhagic cystitis typically require hospitalization and treatment with antibiotics.  Here is a table of the BEDI grading system:  \| Grade \| Bleeding \| Extent \| Dysuria \| Infection \|  \|---\|---\|---\|---\|---\|  \| I \| Mild \| Bladder \| Present \| Absent \|  \| II \| Moderate \| Bladder and/or urethra \| Present \| Absent \|  \| III \| Severe \| Bladder and/or urethra \| Present \| Present \|  \| IV \| Life-threatening \| Bladder and/or urethra \| Present \| Present \|  I hope this information is helpful. Please let me know if you have any other questions. | A patient suffering from hemorrhagic cystitis with hematuria and visible clots would be scored a **BEDI score of 3**. This is because the patient has moderate bleeding, with visible clots, and dysuria. The patient does not have infection, so they would not be scored higher than a 3.  The BEDI grading system is a clinical scoring system used to assess the severity of hemorrhagic cystitis. It is a four-tiered system, with a score of 1 being the mildest and a score of 4 being the most severe.  The BEDI grading system is based on the following criteria:  * **B**leeding: The amount of bleeding, as assessed by the patient's report of hematuria and the presence of visible clots.  * **E**xtent: The extent of bleeding, as assessed by the presence of bleeding in the bladder and/or urethra.  * **D**ysuria: The presence of dysuria, or painful urination.  * **I**nfection: The presence of infection, as assessed by the presence of fever, chills, or other signs of infection.  A patient with hemorrhagic cystitis and hematuria and visible clots would be scored a 3 on the BEDI grading system. This is because the patient has moderate bleeding, with visible clots, and dysuria. The patient does not have infection, so they would not be scored higher than a 3.  The BEDI grading system is a useful tool for assessing the severity of hemorrhagic cystitis and guiding treatment. Patients with a BEDI score of 3 typically require hospitalization and treatment with antibiotics.  Here is a table of the BEDI grading system:  \| Grade \| Bleeding \| Extent \| Dysuria \| Infection \|  \|---\|---\|---\|---\|---\|  \| 1 \| Mild \| Bladder \| Present \| Absent \|  \| 2 \| Moderate \| Bladder and/or urethra \| Present \| Absent \|  \| 3 \| Severe \| Bladder and/or urethra \| Present \| Present \|  \| 4 \| Life-threatening \| Bladder and/or urethra \| Present \| Present \|  I hope this information is helpful. Please let me know if you have any other questions. | The BEDI grade for a patient suffering from hemorrhagic cystitis with hematuria and visible clots is **3**.  The BEDI grading system is a clinical scoring system used to assess the severity of hemorrhagic cystitis. It is a four-tiered system, with Grade 1 being the mildest and Grade 4 being the most severe.  The BEDI grading system is based on the following criteria:  * **B**leeding: The amount of bleeding, as assessed by the patient's report of hematuria and the presence of visible clots.  * **E**xtent: The extent of bleeding, as assessed by the presence of bleeding in the bladder and/or urethra.  * **D**ysuria: The presence of dysuria, or painful urination.  * **I**nfection: The presence of infection, as assessed by the presence of fever, chills, or other signs of infection.  A patient with hemorrhagic cystitis and hematuria and visible clots would be scored as Grade 3 on the BEDI grading system. This is because the patient has moderate bleeding, with visible clots, and dysuria. The patient does not have infection, so they would not be scored higher than Grade 3.  The BEDI grading system is a useful tool for assessing the severity of hemorrhagic cystitis and guiding treatment. Patients with Grade 3 hemorrhagic cystitis typically require hospitalization and treatment with antibiotics.  Here is a table of the BEDI grading system:  \| Grade \| Bleeding \| Extent \| Dysuria \| Infection \|  \|---\|---\|---\|---\|---\|  \| 1 \| Mild \| Bladder \| Present \| Absent \|  \| 2 \| Moderate \| Bladder and/or urethra \| Present \| Absent \|  \| 3 \| Severe \| Bladder and/or urethra \| Present \| Present \|  \| 4 \| Life-threatening \| Bladder and/or urethra \| Present \| Present \|  I hope this information is helpful. Please let me know if you have any other questions. |
| C6 | Poor graft function is a condition that occurs after hematopoietic stem cell transplantation (HSCT) when the donor cells do not engraft or take hold in the recipient's body. This can lead to a variety of problems, including anemia, infection, and bleeding.  There are a number of factors that can contribute to poor graft function, including:  * **Incompatibility between the donor and recipient:** If the donor and recipient are not compatible, the donor cells may be rejected by the recipient's immune system.  * **Graft-versus-host disease (GvHD):** GvHD is a condition that occurs when the donor cells attack the recipient's tissues. This can damage the recipient's organs and lead to poor graft function.  * **Preexisting medical conditions:** Patients with certain preexisting medical conditions, such as cancer or HIV, may be more likely to experience poor graft function.  * **Chemotherapy or radiation therapy:** Chemotherapy and radiation therapy can damage the recipient's immune system and make it more difficult for the donor cells to engraft.  The symptoms of poor graft function can vary depending on the underlying cause. However, some common symptoms include:  * **Anemia:** This is a condition in which the body does not have enough red blood cells. Red blood cells carry oxygen to the body's tissues, so anemia can cause fatigue, shortness of breath, and pale skin.  * **Infection:** Poor graft function can increase the risk of infection. Symptoms of infection can vary depending on the type of infection, but they may include fever, chills, cough, and difficulty breathing.  * **Bleeding:** Poor graft function can also increase the risk of bleeding. Symptoms of bleeding can vary depending on the severity of the bleeding, but they may include bruising, bleeding gums, and nosebleeds.  If you are experiencing any of the symptoms of poor graft function, it is important to see your doctor right away. Treatment for poor graft function depends on the underlying cause. However, some common treatments include:  * **Immunosuppressant drugs:** These drugs can help to suppress the recipient's immune system and prevent it from rejecting the donor cells.  * **Graft-versus-host disease (GvHD) treatment:** If GvHD is the cause of poor graft function, treatment will focus on suppressing the donor cells and preventing them from attacking the recipient's tissues.  * **Blood transfusions:** If anemia is the cause of poor graft function, blood transfusions may be necessary to increase the number of red blood cells in the body.  With prompt diagnosis and treatment, most patients with poor graft function can go on to have successful HSCTs. | Poor graft function, also known as graft failure, is a condition that occurs after hematopoietic stem cell transplantation (HSCT) when the donor cells do not engraft or take hold in the recipient's body. This can lead to a variety of problems, including anemia, infection, and bleeding.  There are a number of factors that can contribute to poor graft function, including:  * **Incompatibility between the donor and recipient:** If the donor and recipient are not compatible, the donor cells may be rejected by the recipient's immune system.  * **Graft-versus-host disease (GvHD):** GvHD is a condition that occurs when the donor cells attack the recipient's tissues. This can damage the recipient's organs and lead to poor graft function.  * **Preexisting medical conditions:** Patients with certain preexisting medical conditions, such as cancer or HIV, may be more likely to experience poor graft function.  * **Chemotherapy or radiation therapy:** Chemotherapy and radiation therapy can damage the recipient's immune system and make it more difficult for the donor cells to engraft.  The symptoms of poor graft function can vary depending on the underlying cause. However, some common symptoms include:  * **Anemia:** This is a condition in which the body does not have enough red blood cells. Red blood cells carry oxygen to the body's tissues, so anemia can cause fatigue, shortness of breath, and pale skin.  * **Infection:** Poor graft function can increase the risk of infection. Symptoms of infection can vary depending on the type of infection, but they may include fever, chills, cough, and difficulty breathing.  * **Bleeding:** Poor graft function can also increase the risk of bleeding. Symptoms of bleeding can vary depending on the severity of the bleeding, but they may include bruising, bleeding gums, and nosebleeds.  If you are experiencing any of the symptoms of poor graft function, it is important to see your doctor right away. Treatment for poor graft function depends on the underlying cause. However, some common treatments include:  * **Immunosuppressant drugs:** These drugs can help to suppress the recipient's immune system and prevent it from rejecting the donor cells.  * **Graft-versus-host disease (GvHD) treatment:** If GvHD is the cause of poor graft function, treatment will focus on suppressing the donor cells and preventing them from attacking the recipient's tissues.  * **Blood transfusions:** If anemia is the cause of poor graft function, blood transfusions may be necessary to increase the number of red blood cells in the body.  With prompt diagnosis and treatment, most patients with poor graft function can go on to have successful HSCTs.  I hope this helps! Let me know if you have any other questions. | Poor graft function, also known as graft failure, is a condition that occurs after hematopoietic stem cell transplantation (HSCT) when the donor cells do not engraft or take hold in the recipient's body. This can lead to a variety of problems, including anemia, infection, and bleeding.  There are a number of factors that can contribute to poor graft function, including:  * **Incompatibility between the donor and recipient:** If the donor and recipient are not compatible, the donor cells may be rejected by the recipient's immune system.  * **Graft-versus-host disease (GvHD):** GvHD is a condition that occurs when the donor cells attack the recipient's tissues. This can damage the recipient's organs and lead to poor graft function.  * **Preexisting medical conditions:** Patients with certain preexisting medical conditions, such as cancer or HIV, may be more likely to experience poor graft function.  * **Chemotherapy or radiation therapy:** Chemotherapy and radiation therapy can damage the recipient's immune system and make it more difficult for the donor cells to engraft.  The symptoms of poor graft function can vary depending on the underlying cause. However, some common symptoms include:  * **Anemia:** This is a condition in which the body does not have enough red blood cells. Red blood cells carry oxygen to the body's tissues, so anemia can cause fatigue, shortness of breath, and pale skin.  * **Infection:** Poor graft function can increase the risk of infection. Symptoms of infection can vary depending on the type of infection, but they may include fever, chills, cough, and difficulty breathing.  * **Bleeding:** Poor graft function can also increase the risk of bleeding. Symptoms of bleeding can vary depending on the severity of the bleeding, but they may include bruising, bleeding gums, and nosebleeds.  If you are experiencing any of the symptoms of poor graft function, it is important to see your doctor right away. Treatment for poor graft function depends on the underlying cause. However, some common treatments include:  * **Immunosuppressant drugs:** These drugs can help to suppress the recipient's immune system and prevent it from rejecting the donor cells.  * **Graft-versus-host disease (GvHD) treatment:** If GvHD is the cause of poor graft function, treatment will focus on suppressing the donor cells and preventing them from attacking the recipient's tissues.  * **Blood transfusions:** If anemia is the cause of poor graft function, blood transfusions may be necessary to increase the number of red blood cells in the body.  With prompt diagnosis and treatment, most patients with poor graft function can go on to have successful HSCTs.  Here are some additional information about poor graft function:  * **What is HSCT?** HSCT stands for hematopoietic stem cell transplantation. It is a procedure in which healthy stem cells are transplanted into a patient who has a damaged or diseased bone marrow.  * **What are the symptoms of poor graft function?** The symptoms of poor graft function can vary depending on the underlying cause. However, some common symptoms include anemia, infection, and bleeding.  * **How is poor graft function diagnosed?** Poor graft function is diagnosed by a blood test that looks for the presence of donor cells in the recipient's blood.  * **How is poor graft function treated?** Treatment for poor graft function depends on the underlying cause. However, some common treatments include immunosuppressant drugs, blood transfusions, and graft-versus-host disease (GvHD) treatment.  I hope this helps! Let me know if you have any other questions. |
| C7 | The HCT-CI score, or Hematopoietic Cell Transplantation-specific Comorbidity Index, is a scoring system used to assess the risk of complications in patients undergoing hematopoietic cell transplantation (HCT). It is a 7-factor score, which includes:  * Age  * Performance status  * Chronic obstructive pulmonary disease (COPD)  * History of heart disease  * History of diabetes  * History of cancer  * Prior HCT  The HCT-CI score is calculated by summing the individual scores for each factor. The total score ranges from 0 to 16, with a higher score indicating a higher risk of complications.  The HCT-CI score was developed in 2005 by a group of researchers at the University of Minnesota. The score has been validated in several studies and is now widely used to assess the risk of complications in patients undergoing HCT.  The HCT-CI score is a useful tool for predicting the risk of complications in patients undergoing HCT. It can be used to help guide treatment decisions and to provide patients with information about their prognosis.  Here is a table of the HCT-CI score factors and their weights:  \| Factor \| Weight \|  \|---\|---\|  \| Age (≥ 65 years) \| 2 \|  \| Performance status (Karnofsky ≤ 70) \| 2 \|  \| COPD \| 1 \|  \| History of heart disease \| 1 \|  \| History of diabetes \| 1 \|  \| History of cancer \| 1 \|  \| Prior HCT \| 1 \|  The total score is calculated by summing the individual scores for each factor. The total score ranges from 0 to 16, with a higher score indicating a higher risk of complications.  I hope this information is helpful. Please let me know if you have any other questions. | The HCT-CI score, or Hematopoietic Cell Transplantation-specific Comorbidity Index, is a scoring system used to assess the risk of complications in patients undergoing hematopoietic cell transplantation (HCT). It is a 7-factor score, which includes:  * **Age:** Age ≥ 65 years  * **Performance status:** Karnofsky ≤ 70  * **Chronic obstructive pulmonary disease (COPD):** History of COPD  * **History of heart disease:** History of heart disease  * **History of diabetes:** History of diabetes  * **History of cancer:** History of cancer  * **Prior HCT:** History of prior HCT  The HCT-CI score is calculated by summing the individual scores for each factor. The total score ranges from 0 to 16, with a higher score indicating a higher risk of complications.  The HCT-CI score was developed in 2005 by a group of researchers at the University of Minnesota. The score has been validated in several studies and is now widely used to assess the risk of complications in patients undergoing HCT.  The HCT-CI score is a useful tool for predicting the risk of complications in patients undergoing HCT. It can be used to help guide treatment decisions and to provide patients with information about their prognosis.  Here is a table of the HCT-CI score factors and their weights:  \| Factor \| Weight \|  \|---\|---\|  \| Age (≥ 65 years) \| 2 \|  \| Performance status (Karnofsky ≤ 70) \| 2 \|  \| COPD \| 1 \|  \| History of heart disease \| 1 \|  \| History of diabetes \| 1 \|  \| History of cancer \| 1 \|  \| Prior HCT \| 1 \|  The total score is calculated by summing the individual scores for each factor. The total score ranges from 0 to 16, with a higher score indicating a higher risk of complications.  Here are some of the complications that can be predicted by the HCT-CI score:  * Graft-versus-host disease (GVHD)  * Infection  * Bleeding  * Organ failure  * Death  The HCT-CI score is not a perfect predictor of complications, but it is a useful tool that can help clinicians make informed decisions about treatment and prognosis.  I hope this information is helpful. Please let me know if you have any other questions. | The HCT-CI score, or Hematopoietic Cell Transplantation-specific Comorbidity Index, is a scoring system used to assess the risk of complications in patients undergoing hematopoietic cell transplantation (HCT). It is a 7-factor score, which includes:  * **Age:** Age ≥ 65 years  * **Performance status:** Karnofsky ≤ 70  * **Chronic obstructive pulmonary disease (COPD):** History of COPD  * **History of heart disease:** History of heart disease  * **History of diabetes:** History of diabetes  * **History of cancer:** History of cancer  * **Prior HCT:** History of prior HCT  The HCT-CI score is calculated by summing the individual scores for each factor. The total score ranges from 0 to 16, with a higher score indicating a higher risk of complications.  The HCT-CI score was developed in 2005 by a group of researchers at the University of Minnesota. The score has been validated in several studies and is now widely used to assess the risk of complications in patients undergoing HCT.  The HCT-CI score is a useful tool for predicting the risk of complications in patients undergoing HCT. It can be used to help guide treatment decisions and to provide patients with information about their prognosis.  Here is a table of the HCT-CI score factors and their weights:  \| Factor \| Weight \|  \|---\|---\|  \| Age (≥ 65 years) \| 2 \|  \| Performance status (Karnofsky ≤ 70) \| 2 \|  \| COPD \| 1 \|  \| History of heart disease \| 1 \|  \| History of diabetes \| 1 \|  \| History of cancer \| 1 \|  \| Prior HCT \| 1 \|  The total score is calculated by summing the individual scores for each factor. The total score ranges from 0 to 16, with a higher score indicating a higher risk of complications.  Here are some of the complications that can be predicted by the HCT-CI score:  * Graft-versus-host disease (GVHD)  * Infection  * Bleeding  * Organ failure  * Death  The HCT-CI score is not a perfect predictor of complications, but it is a useful tool that can help clinicians make informed decisions about treatment and prognosis.  I hope this information is helpful. Let me know if you have other questions. |
| C8 | The MRI is likely to show vasogenic edema, which is a type of brain swelling caused by damage to the blood vessels in the brain. This can be seen as increased signal on T2-weighted and fluid-attenuated inversion recovery (FLAIR) MRI sequences. The patient may also have white matter hyperintensities, which are areas of demyelination in the brain. These can be seen as increased signal on T2-weighted and FLAIR MRI sequences.  The diagnosis is likely to be posterior reversible encephalopathy syndrome (PRES), which is a reversible form of brain injury caused by hypertension. PRES is characterized by vasogenic edema, white matter hyperintensities, and seizures. The exact cause of PRES is not known, but it is thought to be caused by a sudden increase in blood pressure.  Other possible diagnoses include:  * Hypertensive encephalopathy  * Central pontine myelinolysis  * Cerebral venous sinus thrombosis  * Subarachnoid hemorrhage  The treatment for PRES is to control the patient's blood pressure. This is usually done with medication, such as antihypertensives. The patient may also need to be treated for seizures. In most cases, PRES is reversible, and the patient will make a full recovery.  Here are some of the symptoms of PRES:  * Headache  * Visual disturbances  * Confusion  * Seizures  * Altered mental status  * Coma  If you are experiencing any of these symptoms, it is important to see a doctor immediately. PRES is a serious condition, but it is often reversible with prompt treatment. | The MRI is likely to show vasogenic edema, which is a type of brain swelling caused by damage to the blood vessels in the brain. This can be seen as increased signal on T2-weighted and fluid-attenuated inversion recovery (FLAIR) MRI sequences. The patient may also have white matter hyperintensities, which are areas of demyelination in the brain. These can be seen as increased signal on T2-weighted and FLAIR MRI sequences.  The diagnosis is likely to be posterior reversible encephalopathy syndrome (PRES), which is a reversible form of brain injury caused by hypertension. PRES is characterized by vasogenic edema, white matter hyperintensities, and seizures. The exact cause of PRES is not known, but it is thought to be caused by a sudden increase in blood pressure.  Other possible diagnoses include:  * Hypertensive encephalopathy  * Central pontine myelinolysis  * Cerebral venous sinus thrombosis  * Subarachnoid hemorrhage  The treatment for PRES is to control the patient's blood pressure. This is usually done with medication, such as antihypertensives. The patient may also need to be treated for seizures. In most cases, PRES is reversible, and the patient will make a full recovery.  Here are some of the symptoms of PRES:  * Headache  * Visual disturbances  * Confusion  * Seizures  * Altered mental status  * Coma  If you are experiencing any of these symptoms, it is important to see a doctor immediately. PRES is a serious condition, but it is often reversible with prompt treatment.  Here are some additional information about PRES:  * PRES is most commonly seen in patients with uncontrolled hypertension, but it can also be seen in patients with other conditions, such as preeclampsia, eclampsia, and systemic lupus erythematosus.  * The symptoms of PRES typically develop over a period of hours to days.  * The diagnosis of PRES is made based on the patient's clinical presentation and the results of imaging studies, such as MRI.  * The treatment for PRES is to control the patient's blood pressure and to treat any seizures.  * In most cases, PRES is reversible, and the patient will make a full recovery.  I hope this information is helpful. Please let me know if you have other questions. | The MRI is likely to show vasogenic edema, which is a type of brain swelling caused by damage to the blood vessels in the brain. This can be seen as increased signal on T2-weighted and fluid-attenuated inversion recovery (FLAIR) MRI sequences. The patient may also have white matter hyperintensities, which are areas of demyelination in the brain. These can be seen as increased signal on T2-weighted and FLAIR MRI sequences.  The diagnosis is likely to be posterior reversible encephalopathy syndrome (PRES), which is a reversible form of brain injury caused by hypertension. PRES is characterized by vasogenic edema, white matter hyperintensities, and seizures. The exact cause of PRES is not known, but it is thought to be caused by a sudden increase in blood pressure.  Other possible diagnoses include:  * Hypertensive encephalopathy  * Central pontine myelinolysis  * Cerebral venous sinus thrombosis  * Subarachnoid hemorrhage  The treatment for PRES is to control the patient's blood pressure. This is usually done with medication, such as antihypertensives. The patient may also need to be treated for seizures. In most cases, PRES is reversible, and the patient will make a full recovery.  Here are some of the symptoms of PRES:  * Headache  * Visual disturbances  * Confusion  * Seizures  * Altered mental status  * Coma  If you are experiencing any of these symptoms, it is important to see a doctor immediately. PRES is a serious condition, but it is often reversible with prompt treatment.  Here are some additional information about PRES:  * PRES is most commonly seen in patients with uncontrolled hypertension, but it can also be seen in patients with other conditions, such as preeclampsia, eclampsia, and systemic lupus erythematosus.  * The symptoms of PRES typically develop over a period of hours to days.  * The diagnosis of PRES is made based on the patient's clinical presentation and the results of imaging studies, such as MRI.  * The treatment for PRES is to control the patient's blood pressure and to treat any seizures.  * In most cases, PRES is reversible, and the patient will make a full recovery.  I hope this information is helpful. Please let me know if you have other questions. |
| C9 | The most likely explanation is that the patient has relapsed with donor-derived acute myeloid leukemia (d-AML). This is a type of AML that arises from the donor's cells after an allogeneic HSCT. The donor's cells have the Y chromosome, which is why the cytogenetic analysis shows that all cells are 46XY.  d-AML is a relatively rare complication of allogeneic HSCT, but it can occur in up to 5% of patients. The risk of d-AML is higher in patients who have a high-risk AML or who have a donor who is a sibling or a parent.  The treatment for d-AML is similar to the treatment for AML that arises in the patient's own cells. This may include chemotherapy, targeted therapy, or stem cell transplantation.  Here are some other possible explanations for the patient's symptoms:  * The patient may have developed a new AML that is unrelated to the donor's cells. This is a less likely explanation, but it is possible.  * The patient may have a mixed chimerism, which means that the patient's bone marrow contains a mixture of donor and patient cells. This could lead to the patient's cells being 46XY.  If the patient has d-AML, the prognosis is generally poor. However, there have been some cases of patients who have been cured with treatment.  I hope this information is helpful. Please let me know if you have other questions. | The most likely explanation is that the patient has donor-derived acute myeloid leukemia (d-AML). This is a type of AML that arises from the donor's cells after an allogeneic HSCT. The donor's cells have the Y chromosome, which is why the cytogenetic analysis shows that all cells are 46XY.  d-AML is a relatively rare complication of allogeneic HSCT, but it can occur in up to 5% of patients. The risk of d-AML is higher in patients who have a high-risk AML or who have a donor who is a sibling or a parent.  The treatment for d-AML is similar to the treatment for AML that arises in the patient's own cells. This may include chemotherapy, targeted therapy, or stem cell transplantation.  Here are some other possible explanations for the patient's symptoms:  * The patient may have developed a new AML that is unrelated to the donor's cells. This is a less likely explanation, but it is possible.  * The patient may have a mixed chimerism, which means that the patient's bone marrow contains a mixture of donor and patient cells. This could lead to the patient's cells being 46XY.  If the patient has d-AML, the prognosis is generally poor. However, there have been some cases of patients who have been cured with treatment.  Here are some additional information about d-AML:  * d-AML is thought to arise from genetic changes that occur in the donor's cells during the transplantation process.  * The symptoms of d-AML are similar to the symptoms of AML that arises in the patient's own cells.  * The diagnosis of d-AML is made based on the patient's clinical presentation and the results of laboratory tests, such as bone marrow biopsy and cytogenetic analysis.  * The treatment for d-AML is similar to the treatment for AML that arises in the patient's own cells.  I hope this information is helpful. Please let me know if you have any other questions. | The most likely explanation is that the patient has **donor-derived acute myeloid leukemia (d-AML)**. This is a type of AML that arises from the donor's cells after an allogeneic HSCT. The donor's cells have the Y chromosome, which is why the cytogenetic analysis shows that all cells are 46XY.  d-AML is a relatively rare complication of allogeneic HSCT, but it can occur in up to 5% of patients. The risk of d-AML is higher in patients who have a high-risk AML or who have a donor who is a sibling or a parent.  The treatment for d-AML is similar to the treatment for AML that arises in the patient's own cells. This may include chemotherapy, targeted therapy, or stem cell transplantation.  Here are some other possible explanations for the patient's symptoms:  * The patient may have developed a new AML that is unrelated to the donor's cells. This is a less likely explanation, but it is possible.  * The patient may have a mixed chimerism, which means that the patient's bone marrow contains a mixture of donor and patient cells. This could lead to the patient's cells being 46XY.  If the patient has d-AML, the prognosis is generally poor. However, there have been some cases of patients who have been cured with treatment.  Here are some additional information about d-AML:  * d-AML is thought to arise from genetic changes that occur in the donor's cells during the transplantation process.  * The symptoms of d-AML are similar to the symptoms of AML that arises in the patient's own cells.  * The diagnosis of d-AML is made based on the patient's clinical presentation and the results of laboratory tests, such as bone marrow biopsy and cytogenetic analysis.  * The treatment for d-AML is similar to the treatment for AML that arises in the patient's own cells.  I hope this information is helpful. Please let me know if you have any other questions. |
| C10 | A potential specific complication for this setting is **hemolysis**. This is the destruction of red blood cells. It can occur when the recipient's immune system sees the donor's A antigens as foreign and attacks them. This can lead to the destruction of the donor's red blood cells, which can cause a number of problems, including:  * **Jaundice**, which is a yellowing of the skin and eyes.  * **Anemia**, which is a low red blood cell count.  * **Thrombocytopenia**, which is a low platelet count.  * **Hypotension**, which is a low blood pressure.  In some cases, hemolysis can be severe and even fatal. However, there are a number of things that can be done to prevent or treat hemolysis, including:  * **Matching the donor and recipient's blood types as closely as possible**.  * **Using apheresis products, which are blood products that have been filtered to remove the donor's antigens**.  * **Giving the recipient immunosuppressive drugs**.  It is important to note that hemolysis is not the only potential complication of an HSC transplant from a donor with a different blood type. Other potential complications include:  * **Graft-versus-host disease (GVHD)**, which is a serious condition in which the donor's cells attack the recipient's tissues.  * **Infection**, as the recipient's immune system will be suppressed following the transplant.  * **Transfusion reactions**, which can occur if the donor and recipient's blood types are not matched correctly.  It is important to discuss the risks and benefits of an HSC transplant with a doctor before making a decision. | A potential specific complication for this setting is **hemolysis**. This is the destruction of red blood cells. It can occur when the recipient's immune system sees the donor's A antigens as foreign and attacks them. This can lead to the destruction of the donor's red blood cells, which can cause a number of problems, including:  * **Jaundice**, which is a yellowing of the skin and eyes.  * **Anemia**, which is a low red blood cell count.  * **Thrombocytopenia**, which is a low platelet count.  * **Hypotension**, which is a low blood pressure.  In some cases, hemolysis can be severe and even fatal. However, there are a number of things that can be done to prevent or treat hemolysis, including:  * **Matching the donor and recipient's blood types as closely as possible**.  * **Using apheresis products, which are blood products that have been filtered to remove the donor's antigens**.  * **Giving the recipient immunosuppressive drugs**.  It is important to note that hemolysis is not the only potential complication of an HSC transplant from a donor with a different blood type. Other potential complications include:  * **Graft-versus-host disease (GVHD)**, which is a serious condition in which the donor's cells attack the recipient's tissues.  * **Infection**, as the recipient's immune system will be suppressed following the transplant.  * **Transfusion reactions**, which can occur if the donor and recipient's blood types are not matched correctly.  It is important to discuss the risks and benefits of an HSC transplant with a doctor before making a decision. | The potential specific complication for this setting is **hemolysis**. This is the destruction of red blood cells. It can occur when the recipient's immune system sees the donor's A antigens as foreign and attacks them. This can lead to the destruction of the donor's red blood cells, which can cause a number of problems, including:  * **Jaundice**, which is a yellowing of the skin and eyes.  * **Anemia**, which is a low red blood cell count.  * **Thrombocytopenia**, which is a low platelet count.  * **Hypotension**, which is a low blood pressure.  In some cases, hemolysis can be severe and even fatal. However, there are a number of things that can be done to prevent or treat hemolysis, including:  * **Matching the donor and recipient's blood types as closely as possible**.  * **Using apheresis products, which are blood products that have been filtered to remove the donor's antigens**.  * **Giving the recipient immunosuppressive drugs**.  It is important to note that hemolysis is not the only potential complication of an HSC transplant from a donor with a different blood type. Other potential complications include:  * **Graft-versus-host disease (GVHD)**, which is a serious condition in which the donor's cells attack the recipient's tissues.  * **Infection**, as the recipient's immune system will be suppressed following the transplant.  * **Transfusion reactions**, which can occur if the donor and recipient's blood types are not matched correctly.  It is important to discuss the risks and benefits of an HSC transplant with a doctor before making a decision.  Here are some additional information about hemolysis:  * Hemolysis is caused by the destruction of red blood cells.  * Red blood cells contain antigens, which are proteins that the body's immune system recognizes as foreign.  * If the recipient's immune system sees the donor's antigens as foreign, it will attack the donor's red blood cells.  * This can lead to the destruction of the donor's red blood cells, which can cause a number of problems.  I hope this information is helpful. Please let me know if you have any other questions. |
| C11 | The patient is showing signs of **cyclosporine toxicity**. Cyclosporine is an immunosuppressant drug that is used to prevent graft-versus-host disease (GVHD) after an HSCT. However, it can also cause a number of side effects, including:  * Hypertension  * Decreased renal function  * Anemia  * Thrombocytopenia  Other laboratory findings that are often associated with cyclosporine toxicity include:  * Hyperkalemia  * Hypomagnesemia  * Hyperuricemia  * Elevated liver enzymes  * Eosinophilia  A blood smear may show evidence of hemolysis, which is the destruction of red blood cells. This is because cyclosporine can damage the red blood cells' cell membranes.  If cyclosporine toxicity is suspected, the drug should be discontinued and the patient should be treated with supportive measures, such as fluids, electrolytes, and blood transfusions.  Here are some additional information about cyclosporine toxicity:  * Cyclosporine toxicity is more likely to occur in patients who are taking high doses of the drug or who have underlying kidney disease.  * The symptoms of cyclosporine toxicity can develop over a period of days or weeks.  * The diagnosis of cyclosporine toxicity is made based on the patient's clinical presentation and the results of laboratory tests.  * Treatment for cyclosporine toxicity involves discontinuing the drug and providing supportive care.  I hope this information is helpful. Please let me know if you have any other questions. | The patient is showing signs of **cyclosporine toxicity**. Cyclosporine is an immunosuppressant drug that is used to prevent graft-versus-host disease (GVHD) after an HSCT. However, it can also cause a number of side effects, including:  * Hypertension  * Decreased renal function  * Anemia  * Thrombocytopenia  Other laboratory findings that are often associated with cyclosporine toxicity include:  * Hyperkalemia  * Hypomagnesemia  * Hyperuricemia  * Elevated liver enzymes  * Eosinophilia  A blood smear may show evidence of hemolysis, which is the destruction of red blood cells. This is because cyclosporine can damage the red blood cells' cell membranes.  If cyclosporine toxicity is suspected, the drug should be discontinued and the patient should be treated with supportive measures, such as fluids, electrolytes, and blood transfusions.  Here are some additional information about cyclosporine toxicity:  * Cyclosporine toxicity is more likely to occur in patients who are taking high doses of the drug or who have underlying kidney disease.  * The symptoms of cyclosporine toxicity can develop over a period of days or weeks.  * The diagnosis of cyclosporine toxicity is made based on the patient's clinical presentation and the results of laboratory tests.  * Treatment for cyclosporine toxicity involves discontinuing the drug and providing supportive care.  **Other laboratory findings on the blood smear that may be associated with cyclosporine toxicity include:**  * **Increased white blood cell count**. This is due to the release of inflammatory mediators from damaged cells.  * **Decreased platelet count**. This is due to the destruction of platelets by cyclosporine.  * **Abnormal red blood cells**. These may include cells that are small, large, or misshapen.  I hope this information is helpful. Please let me know if you have any other questions. | The patient is showing signs of **cyclosporine toxicity**. Cyclosporine is an immunosuppressant drug that is used to prevent graft-versus-host disease (GVHD) after an HSCT. However, it can also cause a number of side effects, including:  * Hypertension  * Decreased renal function  * Anemia  * Thrombocytopenia  Other laboratory findings that are often associated with cyclosporine toxicity include:  * Hyperkalemia  * Hypomagnesemia  * Hyperuricemia  * Elevated liver enzymes  * Eosinophilia  A blood smear may show evidence of hemolysis, which is the destruction of red blood cells. This is because cyclosporine can damage the red blood cells' cell membranes.  If cyclosporine toxicity is suspected, the drug should be discontinued and the patient should be treated with supportive measures, such as fluids, electrolytes, and blood transfusions.  **Other laboratory findings on the blood smear that may be associated with cyclosporine toxicity include:**  * **Increased white blood cell count**. This is due to the release of inflammatory mediators from damaged cells.  * **Decreased platelet count**. This is due to the destruction of platelets by cyclosporine.  * **Abnormal red blood cells**. These may include cells that are small, large, or misshapen.  * **Schistocytes**. These are fragments of red blood cells that are caused by the destruction of red blood cells by cyclosporine.  I hope this information is helpful. Please let me know if you have any other questions. |
| C12 | Primary graft failure (PGF) and secondary graft failure (SGF) are two types of graft failure that can occur after hematopoietic stem cell transplantation (HSCT).  * **Primary graft failure** is when the donor cells do not engraft or take hold in the recipient's body at all. This is a serious complication that can be fatal.  * **Secondary graft failure** is when the donor cells initially engraft, but then they fail to function properly. This can happen months or even years after the transplant.  The causes of PGF and SGF are not fully understood, but they are thought to be due to a combination of factors, including:  * **Incompatibility between the donor and recipient:** If the donor and recipient are not compatible, the donor cells may be rejected by the recipient's immune system.  * **Graft-versus-host disease (GvHD):** GvHD is a condition that occurs when the donor cells attack the recipient's tissues. This can damage the recipient's organs and lead to poor graft function.  * **Preexisting medical conditions:** Patients with certain preexisting medical conditions, such as cancer or HIV, may be more likely to experience PGF or SGF.  * **Chemotherapy or radiation therapy:** Chemotherapy and radiation therapy can damage the recipient's immune system and make it more difficult for the donor cells to engraft.  The symptoms of PGF and SGF can vary depending on the underlying cause. However, some common symptoms include:  * **Anemia:** This is a condition in which the body does not have enough red blood cells. Red blood cells carry oxygen to the body's tissues, so anemia can cause fatigue, shortness of breath, and pale skin.  * **Infection:** PGF and SGF can increase the risk of infection. Symptoms of infection can vary depending on the type of infection, but they may include fever, chills, cough, and difficulty breathing.  * **Bleeding:** PGF and SGF can also increase the risk of bleeding. Symptoms of bleeding can vary depending on the severity of the bleeding, but they may include bruising, bleeding gums, and nosebleeds.  If you are experiencing any of the symptoms of PGF or SGF, it is important to see your doctor right away. Treatment for PGF and SGF depends on the underlying cause. However, some common treatments include:  * **Immunosuppressant drugs:** These drugs can help to suppress the recipient's immune system and prevent it from rejecting the donor cells.  * **Graft-versus-host disease (GvHD) treatment:** If GvHD is the cause of PGF or SGF, treatment will focus on suppressing the donor cells and preventing them from attacking the recipient's tissues.  * **Blood transfusions:** If anemia is the cause of PGF or SGF, blood transfusions may be necessary to increase the number of red blood cells in the body.  * **Re-transplant:** In some cases, a second transplant may be an option.  The prognosis for patients with PGF or SGF depends on the underlying cause and the severity of the symptoms. However, with prompt diagnosis and treatment, most patients can go on to have successful HSCTs.  Here are some additional information about primary and secondary graft failure:  * **What is HSCT?** HSCT stands for hematopoietic stem cell transplantation. It is a procedure in which healthy stem cells are transplanted into a patient who has a damaged or diseased bone marrow.  * **What are the symptoms of primary and secondary graft failure?** The symptoms of primary and secondary graft failure can vary depending on the underlying cause. However, some common symptoms include anemia, infection, and bleeding.  * **How is primary and secondary graft failure diagnosed?** Primary and secondary graft failure are diagnosed by a blood test that looks for the presence of donor cells in the recipient's blood.  * **How is primary and secondary graft failure treated?** Treatment for primary and secondary graft failure depends on the underlying cause. However, some common treatments include immunosuppressant drugs, blood transfusions, and re-transplant.  I hope this helps! Let me know if you have any other questions. | **Primary graft failure (PGF)** is when the donor cells do not engraft or take hold in the recipient's body at all. This is a serious complication that can be fatal.  **Secondary graft failure (SGF)** is when the donor cells initially engraft, but then they fail to function properly. This can happen months or even years after the transplant.  The main difference between PGF and SGF is the timing of the graft failure. PGF occurs within the first 100 days after the transplant, while SGF can occur at any time after the transplant.  The causes of PGF and SGF are not fully understood, but they are thought to be due to a combination of factors, including:  * **Incompatibility between the donor and recipient:** If the donor and recipient are not compatible, the donor cells may be rejected by the recipient's immune system.  * **Graft-versus-host disease (GvHD)**: GvHD is a condition that occurs when the donor cells attack the recipient's tissues. This can damage the recipient's organs and lead to poor graft function.  * **Preexisting medical conditions:** Patients with certain preexisting medical conditions, such as cancer or HIV, may be more likely to experience PGF or SGF.  * **Chemotherapy or radiation therapy:** Chemotherapy and radiation therapy can damage the recipient's immune system and make it more difficult for the donor cells to engraft.  The symptoms of PGF and SGF can vary depending on the underlying cause. However, some common symptoms include:  * **Anemia:** This is a condition in which the body does not have enough red blood cells. Red blood cells carry oxygen to the body's tissues, so anemia can cause fatigue, shortness of breath, and pale skin.  * **Infection:** PGF and SGF can increase the risk of infection. Symptoms of infection can vary depending on the type of infection, but they may include fever, chills, cough, and difficulty breathing.  * **Bleeding:** PGF and SGF can also increase the risk of bleeding. Symptoms of bleeding can vary depending on the severity of the bleeding, but they may include bruising, bleeding gums, and nosebleeds.  If you are experiencing any of the symptoms of PGF or SGF, it is important to see your doctor right away. Treatment for PGF and SGF depends on the underlying cause. However, some common treatments include:  * **Immunosuppressant drugs:** These drugs can help to suppress the recipient's immune system and prevent it from rejecting the donor cells.  * **Graft-versus-host disease (GvHD) treatment:** If GvHD is the cause of PGF or SGF, treatment will focus on suppressing the donor cells and preventing them from attacking the recipient's tissues.  * **Blood transfusions:** If anemia is the cause of PGF or SGF, blood transfusions may be necessary to increase the number of red blood cells in the body.  * **Re-transplant:** In some cases, a second transplant may be an option.  The prognosis for patients with PGF or SGF depends on the underlying cause and the severity of the symptoms. However, with prompt diagnosis and treatment, most patients can go on to have successful HSCTs.  Here are some additional information about primary and secondary graft failure:  * **What is HSCT?** HSCT stands for hematopoietic stem cell transplantation. It is a procedure in which healthy stem cells are transplanted into a patient who has a damaged or diseased bone marrow.  * **What are the symptoms of primary and secondary graft failure?** The symptoms of primary and secondary graft failure can vary depending on the underlying cause. However, some common symptoms include anemia, infection, and bleeding.  * **How is primary and secondary graft failure diagnosed?** Primary and secondary graft failure are diagnosed by a blood test that looks for the presence of donor cells in the recipient's blood.  * **How is primary and secondary graft failure treated?** Treatment for primary and secondary graft failure depends on the underlying cause. However, some common treatments include immunosuppressant drugs, blood transfusions, and re-transplant.  I hope this helps! Let me know if you have any other questions. | Sure! I can help you with that.  Primary graft failure (PGF) and secondary graft failure (SGF) are two types of graft failure that can occur after hematopoietic stem cell transplantation (HSCT).  * **Primary graft failure** is when the donor cells do not engraft or take hold in the recipient's body at all. This is a serious complication that can be fatal.  * **Secondary graft failure** is when the donor cells initially engraft, but then they fail to function properly. This can happen months or even years after the transplant.  The main difference between PGF and SGF is the timing of the graft failure. PGF occurs within the first 100 days after the transplant, while SGF can occur at any time after the transplant.  The causes of PGF and SGF are not fully understood, but they are thought to be due to a combination of factors, including:  * **Incompatibility between the donor and recipient:** If the donor and recipient are not compatible, the donor cells may be rejected by the recipient's immune system.  * **Graft-versus-host disease (GvHD)**: GvHD is a condition that occurs when the donor cells attack the recipient's tissues. This can damage the recipient's organs and lead to poor graft function.  * **Preexisting medical conditions:** Patients with certain preexisting medical conditions, such as cancer or HIV, may be more likely to experience PGF or SGF.  * **Chemotherapy or radiation therapy:** Chemotherapy and radiation therapy can damage the recipient's immune system and make it more difficult for the donor cells to engraft.  The symptoms of PGF and SGF can vary depending on the underlying cause. However, some common symptoms include:  * **Anemia:** This is a condition in which the body does not have enough red blood cells. Red blood cells carry oxygen to the body's tissues, so anemia can cause fatigue, shortness of breath, and pale skin.  * **Infection:** PGF and SGF can increase the risk of infection. Symptoms of infection can vary depending on the type of infection, but they may include fever, chills, cough, and difficulty breathing.  * **Bleeding:** PGF and SGF can also increase the risk of bleeding. Symptoms of bleeding can vary depending on the severity of the bleeding, but they may include bruising, bleeding gums, and nosebleeds.  If you are experiencing any of the symptoms of PGF or SGF, it is important to see your doctor right away. Treatment for PGF and SGF depends on the underlying cause. However, some common treatments include:  * **Immunosuppressant drugs:** These drugs can help to suppress the recipient's immune system and prevent it from rejecting the donor cells.  * **Graft-versus-host disease (GvHD) treatment:** If GvHD is the cause of PGF or SGF, treatment will focus on suppressing the donor cells and preventing them from attacking the recipient's tissues.  * **Blood transfusions:** If anemia is the cause of PGF or SGF, blood transfusions may be necessary to increase the number of red blood cells in the body.  * **Re-transplant:** In some cases, a second transplant may be an option.  The prognosis for patients with PGF or SGF depends on the underlying cause and the severity of the symptoms. However, with prompt diagnosis and treatment, most patients can go on to have successful HSCTs.  Here are some additional information about primary and secondary graft failure:  * **What is HSCT?** HSCT stands for hematopoietic stem cell transplantation. It is a procedure in which healthy stem cells are transplanted into a patient who has a damaged or diseased bone marrow.  * **What are the symptoms of primary and secondary graft failure?** The symptoms of primary and secondary graft failure can vary depending on the underlying cause. However, some common symptoms include anemia, infection, and bleeding.  * **How is primary and secondary graft failure diagnosed?** Primary and secondary graft failure are diagnosed by a blood test that looks for the presence of donor cells in the recipient's blood.  * **How is primary and secondary graft failure treated?** Treatment for primary and secondary graft failure depends on the underlying cause. However, some common treatments include immunosuppressant drugs, blood transfusions, and re-transplant.  I hope this helps! Let me know if you have any other questions. |
| C13 | Yes, donor lymphocyte infusion (DLI) should be offered to the patient. DLI is a treatment for acute myeloid leukemia (AML) that has relapsed after a haploidentical transplant. DLI involves giving the patient donor lymphocytes, which are white blood cells that help the immune system fight infection.  In the case of the patient described in the question, the leukemic cells have lost the patient-donor mismatched HLA. This means that the leukemic cells are no longer recognized by the donor's immune system, which makes it difficult for the donor's immune system to fight the leukemia. DLI can help to restore the donor's immune system's ability to recognize and fight the leukemia.  DLI is not without risks. It can cause graft-versus-host disease (GVHD), which is a serious condition in which the donor's cells attack the patient's tissues. However, the benefits of DLI can outweigh the risks in some cases.  The decision of whether or not to offer DLI to a patient with relapsed AML after a haploidentical transplant is a complex one. It should be made on a case-by-case basis, taking into account the patient's individual circumstances.  Here are some of the factors that may be considered when making the decision to offer DLI:  * The patient's age and overall health  * The type of AML the patient has  * The extent of the patient's relapse  * The patient's risk of GVHD  * The patient's wishes  If you are a patient with relapsed AML after a haploidentical transplant, you should talk to your doctor about whether or not DLI is right for you. | Yes, donor lymphocyte infusion (DLI) should be offered to the patient. DLI is a treatment for acute myeloid leukemia (AML) that has relapsed after a haploidentical transplant. DLI involves giving the patient donor lymphocytes, which are white blood cells that help the immune system fight infection.  In the case of the patient described in the question, the leukemic cells have lost the patient-donor mismatched HLA. This means that the leukemic cells are no longer recognized by the donor's immune system, which makes it difficult for the donor's immune system to fight the leukemia. DLI can help to restore the donor's immune system's ability to recognize and fight the leukemia.  DLI is not without risks. It can cause graft-versus-host disease (GVHD), which is a serious condition in which the donor's cells attack the patient's tissues. However, the benefits of DLI can outweigh the risks in some cases.  The decision of whether or not to offer DLI to a patient with relapsed AML after a haploidentical transplant is a complex one. It should be made on a case-by-case basis, taking into account the patient's individual circumstances.  Here are some of the factors that may be considered when making the decision to offer DLI:  * The patient's age and overall health  * The type of AML the patient has  * The extent of the patient's relapse  * The patient's risk of GVHD  * The patient's wishes  If you are a patient with relapsed AML after a haploidentical transplant, you should talk to your doctor about whether or not DLI is right for you.  Here are some additional information about DLI:  * DLI is usually given as a single infusion of donor lymphocytes.  * The number of donor lymphocytes given depends on the patient's individual circumstances.  * DLI can be given as a treatment for relapsed AML or as a preventive measure to help prevent relapse.  * DLI can cause a variety of side effects, including GVHD, fever, and infection.  I hope this information is helpful. Please let me know if you have any other questions. | Yes, donor lymphocyte infusion (DLI) should be offered to the patient. DLI is a treatment for acute myeloid leukemia (AML) that has relapsed after a haploidentical transplant. DLI involves giving the patient donor lymphocytes, which are white blood cells that help the immune system fight infection.  In the case of the patient described in the question, the leukemic cells have lost the patient-donor mismatched HLA. This means that the leukemic cells are no longer recognized by the donor's immune system, which makes it difficult for the donor's immune system to fight the leukemia. DLI can help to restore the donor's immune system's ability to recognize and fight the leukemia.  However, there are some risks associated with DLI, including:  * **Graft-versus-host disease (GVHD)**. GVHD is a serious condition in which the donor's cells attack the patient's tissues.  * **Fever**. DLI can cause fever, which is a sign of infection.  * **Infection**. DLI can weaken the patient's immune system, making them more susceptible to infection.  The decision of whether or not to offer DLI to a patient with relapsed AML after a haploidentical transplant is a complex one. It should be made on a case-by-case basis, taking into account the patient's individual circumstances.  Here are some of the factors that may be considered when making the decision to offer DLI:  * The patient's age and overall health  * The type of AML the patient has  * The extent of the patient's relapse  * The patient's risk of GVHD  * The patient's wishes  If you are a patient with relapsed AML after a haploidentical transplant, you should talk to your doctor about whether or not DLI is right for you.  I hope this information is helpful. Please let me know if you have any other questions. |
| C14 | Uniparental disomy of short arm of chromosome 6 (UPD6) is a condition in which both copies of chromosome 6 come from the same parent. This can happen if there is a problem with meiosis, the process by which chromosomes are passed on from parents to offspring.  In AML, UPD6 is associated with a poor prognosis. This is because it is thought to lead to the loss of important genes on chromosome 6. These genes are involved in regulating cell growth and differentiation, and their loss can make the cancer cells more aggressive.  There are a few treatment options available for patients with AML who have relapsed after haploidentical HSCT. These options include:  * **Chemotherapy:** Chemotherapy is a systemic treatment that uses drugs to kill cancer cells. It is the most common treatment for AML.  * **Targeted therapy:** Targeted therapy uses drugs that target specific proteins or pathways involved in cancer growth. This type of therapy can be more effective than chemotherapy in some cases.  * **Stem cell transplant:** A stem cell transplant is a procedure in which healthy stem cells are transplanted into the patient's body. This can help to restore the patient's immune system and fight the cancer.  The best treatment option for a patient with AML who has relapsed after haploidentical HSCT will depend on the patient's individual circumstances. The patient's age, health status, and the type of AML they have will all be factors that the doctor will consider when making a treatment plan.  In the case of a patient with UPD6, the doctor may recommend a more aggressive treatment plan, such as a combination of chemotherapy and targeted therapy. This is because UPD6 is associated with a poor prognosis, and the doctor may want to take steps to improve the patient's chances of survival.  The patient may also be eligible for a stem cell transplant. A stem cell transplant can be a very effective treatment for AML, but it is also a risky procedure. The doctor will discuss the risks and benefits of a stem cell transplant with the patient before making a recommendation.  The prognosis for a patient with AML who has relapsed after haploidentical HSCT is variable. Some patients may go into remission with treatment, while others may not. The patient's age, health status, and the type of AML they have will all affect their prognosis.  The doctor will discuss the patient's prognosis with them and their family. They will also discuss the different treatment options available and help the patient make a decision about the best course of treatment. | Uniparental disomy of short arm of chromosome 6 (UPD6) is a condition in which both copies of chromosome 6 come from the same parent. This can happen if there is a problem with meiosis, the process by which chromosomes are passed on from parents to offspring.  In AML, UPD6 is associated with a poor prognosis. This is because it is thought to lead to the loss of important genes on chromosome 6. These genes are involved in regulating cell growth and differentiation, and their loss can make the cancer cells more aggressive.  There are a few treatment options available for patients with AML who have relapsed after haploidentical HSCT. These options include:  * **Chemotherapy:** Chemotherapy is a systemic treatment that uses drugs to kill cancer cells. It is the most common treatment for AML.  * **Targeted therapy:** Targeted therapy uses drugs that target specific proteins or pathways involved in cancer growth. This type of therapy can be more effective than chemotherapy in some cases.  * **Stem cell transplant:** A stem cell transplant is a procedure in which healthy stem cells are transplanted into the patient's body. This can help to restore the patient's immune system and fight the cancer.  The best treatment option for a patient with AML who has relapsed after haploidentical HSCT will depend on the patient's individual circumstances. The patient's age, health status, and the type of AML they have will all be factors that the doctor will consider when making a treatment plan.  In the case of a patient with UPD6, the doctor may recommend a more aggressive treatment plan, such as a combination of chemotherapy and targeted therapy. This is because UPD6 is associated with a poor prognosis, and the doctor may want to take steps to improve the patient's chances of survival.  The patient may also be eligible for a stem cell transplant. A stem cell transplant can be a very effective treatment for AML, but it is also a risky procedure. The doctor will discuss the risks and benefits of a stem cell transplant with the patient before making a recommendation.  The prognosis for a patient with AML who has relapsed after haploidentical HSCT is variable. Some patients may go into remission with treatment, while others may not. The patient's age, health status, and the type of AML they have will all affect their prognosis.  The doctor will discuss the patient's prognosis with them and their family. They will also discuss the different treatment options available and help the patient make a decision about the best course of treatment. | Uniparental disomy of short arm of chromosome 6 (UPD6) is a condition in which both copies of chromosome 6 come from the same parent. This can happen if there is a problem with meiosis, the process by which chromosomes are passed on from parents to offspring.  In AML, UPD6 is associated with a poor prognosis. This is because it is thought to lead to the loss of important genes on chromosome 6. These genes are involved in regulating cell growth and differentiation, and their loss can make the cancer cells more aggressive.  The loss of these genes can also make the cancer cells more resistant to treatment. This is because the cancer cells may no longer be able to respond to the drugs that are used to treat AML.  As a result, patients with AML who have relapsed after haploidentical HSCT and have UPD6 may have a poorer prognosis than patients who do not have UPD6. They may also be less likely to respond to treatment.  The best treatment option for these patients will depend on their individual circumstances. However, the doctor may recommend a more aggressive treatment plan, such as a combination of chemotherapy and targeted therapy.  The patient may also be eligible for a stem cell transplant. A stem cell transplant can be a very effective treatment for AML, but it is also a risky procedure. The doctor will discuss the risks and benefits of a stem cell transplant with the patient before making a recommendation.  The prognosis for a patient with AML who has relapsed after haploidentical HSCT and has UPD6 is variable. Some patients may go into remission with treatment, while others may not. The patient's age, health status, and the type of AML they have will all affect their prognosis.  The doctor will discuss the patient's prognosis with them and their family. They will also discuss the different treatment options available and help the patient make a decision about the best course of treatment.  Here are some additional information about UPD6:  * UPD6 is a rare condition, but it is more common in patients with AML.  * The exact mechanism by which UPD6 leads to a poor prognosis is not fully understood.  * There are no specific treatments for UPD6, but patients may be treated with chemotherapy, targeted therapy, or stem cell transplant.  * The prognosis for patients with AML who have relapsed after haploidentical HSCT and have UPD6 is variable.  I hope this information is helpful. Please let me know if you have any other questions. |
| C15 | The patient is most likely experiencing **idiopathic refractory ascites** (IRA), a rare but serious complication of allogeneic HSCT. IRA is characterized by the rapid accumulation of fluid in the abdomen, which can lead to weight gain, increased bilirubin levels, and painful hepatomegaly.  The exact cause of IRA is unknown, but it is thought to be due to a combination of factors, including:  * **Graft-versus-host disease (GVHD)**. GVHD is a condition in which the donor's immune cells attack the patient's tissues. In the case of IRA, GVHD can damage the liver, leading to fluid retention.  * **Immunosuppressive drugs**. The immunosuppressive drugs that are used to prevent GVHD can also increase the risk of IRA.  * **Underlying liver disease**. Patients with underlying liver disease, such as hepatitis C or cirrhosis, are at an increased risk of developing IRA.  There is no specific treatment for IRA, but the symptoms can be managed with a variety of medications, including:  * **Spironolactone**. Spironolactone is a diuretic that helps to reduce fluid retention.  * **Furosemide**. Furosemide is another diuretic that can be used to reduce fluid retention.  * **Albumin**. Albumin is a protein that helps to keep fluid in the bloodstream. It can be given intravenously to help reduce ascites.  * **Corticosteroids**. Corticosteroids can be used to reduce inflammation and improve liver function.  The patient's doctor will need to carefully monitor the patient's condition and adjust the medication as needed. In some cases, surgery may be necessary to drain the ascites.  The prognosis for patients with IRA is variable. Some patients respond well to treatment and the ascites resolves. However, other patients may have a more prolonged course of illness or may develop complications, such as liver failure.  I hope this information is helpful. Please let me know if you have any other questions. | The patient is most likely experiencing **idiopathic refractory ascites** (IRA), a rare but serious complication of allogeneic HSCT. IRA is characterized by the rapid accumulation of fluid in the abdomen, which can lead to weight gain, increased bilirubin levels, and painful hepatomegaly. The exact cause of IRA is unknown, but it is thought to be due to a combination of factors, including:  * **Graft-versus-host disease (GVHD)**. GVHD is a condition in which the donor's immune cells attack the patient's tissues. In the case of IRA, GVHD can damage the liver, leading to fluid retention.  * **Immunosuppressive drugs**. The immunosuppressive drugs that are used to prevent GVHD can also increase the risk of IRA.  * **Underlying liver disease**. Patients with underlying liver disease, such as hepatitis C or cirrhosis, are at an increased risk of developing IRA.  There is no specific treatment for IRA, but the symptoms can be managed with a variety of medications, including:  * **Spironolactone**. Spironolactone is a diuretic that helps to reduce fluid retention.  * **Furosemide**. Furosemide is another diuretic that can be used to reduce fluid retention.  * **Albumin**. Albumin is a protein that helps to keep fluid in the bloodstream. It can be given intravenously to help reduce ascites.  * **Corticosteroids**. Corticosteroids can be used to reduce inflammation and improve liver function.  The patient's doctor will need to carefully monitor the patient's condition and adjust the medication as needed. In some cases, surgery may be necessary to drain the ascites.  The prognosis for patients with IRA is variable. Some patients respond well to treatment and the ascites resolves. However, other patients may have a more prolonged course of illness or may develop complications, such as liver failure.  Based on the information you have provided, the most effective drug for this patient would be **spironolactone**. Spironolactone is a diuretic that helps to reduce fluid retention, which is the main symptom of IRA. It is also relatively safe and well-tolerated, making it a good choice for patients who are already taking other medications.  Other medications, such as furosemide and albumin, may also be used to treat IRA. However, spironolactone is generally considered to be the first-line treatment for this condition.  It is important to note that the treatment for IRA will vary depending on the individual patient's circumstances. The patient's doctor will need to carefully consider the patient's age, overall health, and other medications that the patient is taking before making a treatment decision.  I hope this information is helpful. Please let me know if you have any other questions. | The patient is most likely experiencing **idiopathic refractory ascites** (IRA), a rare but serious complication of allogeneic HSCT. IRA is characterized by the rapid accumulation of fluid in the abdomen, which can lead to weight gain, increased bilirubin levels, and painful hepatomegaly.  The exact cause of IRA is unknown, but it is thought to be due to a combination of factors, including:  * **Graft-versus-host disease (GVHD)**. GVHD is a condition in which the donor's immune cells attack the patient's tissues. In the case of IRA, GVHD can damage the liver, leading to fluid retention.  * **Immunosuppressive drugs**. The immunosuppressive drugs that are used to prevent GVHD can also increase the risk of IRA.  * **Underlying liver disease**. Patients with underlying liver disease, such as hepatitis C or cirrhosis, are at an increased risk of developing IRA.  There is no specific treatment for IRA, but the symptoms can be managed with a variety of medications, including:  * **Spironolactone**. Spironolactone is a diuretic that helps to reduce fluid retention.  * **Furosemide**. Furosemide is another diuretic that can be used to reduce fluid retention.  * **Albumin**. Albumin is a protein that helps to keep fluid in the bloodstream. It can be given intravenously to help reduce ascites.  * **Corticosteroids**. Corticosteroids can be used to reduce inflammation and improve liver function.  The patient's doctor will need to carefully monitor the patient's condition and adjust the medication as needed. In some cases, surgery may be necessary to drain the ascites.  The prognosis for patients with IRA is variable. Some patients respond well to treatment and the ascites resolves. However, other patients may have a more prolonged course of illness or may develop complications, such as liver failure.  Based on the information you have provided, the most effective drug for this patient would be **spironolactone**. Spironolactone is a diuretic that helps to reduce fluid retention, which is the main symptom of IRA. It is also relatively safe and well-tolerated, making it a good choice for patients who are already taking other medications.  Other medications, such as furosemide and albumin, may also be used to treat IRA. However, spironolactone is generally considered to be the first-line treatment for this condition.  It is important to note that the treatment for IRA will vary depending on the individual patient's circumstances. The patient's doctor will need to carefully consider the patient's age, overall health, and other medications that the patient is taking before making a treatment decision.  Here are some additional information about IRA:  * IRA is a rare condition, but it is more common in patients who have undergone allogeneic HSCT.  * IRA can be a serious condition, but it is often treatable.  * The treatment for IRA will vary depending on the individual patient's circumstances.  I hope this information is helpful. Please let me know if you have any other questions. |
| C16 | The prognosis for a patient with AML who has a karyotype of t(1,2) and -7 at diagnosis is poor. The 5-year survival rate for patients with this karyotype is typically less than 20%.  The t(1,2) translocation is a chromosomal abnormality that results in the fusion of two genes, AML1 and RUNX1. This fusion gene is thought to be involved in the development of AML. The -7 deletion is another chromosomal abnormality that is often seen in AML. This deletion results in the loss of a gene called RUNX1, which is also thought to be involved in the development of AML.  The combination of the t(1,2) translocation and the -7 deletion is a very poor prognostic factor for patients with AML. These patients are more likely to relapse after allogeneic HSCT and have a shorter overall survival.  There are a number of factors that can affect the prognosis for patients with AML, including the patient's age, the type of AML, and the patient's response to treatment. However, the presence of the t(1,2) translocation and the -7 deletion is a very strong negative prognostic factor.  If you are a patient with AML who has a karyotype of t(1,2) and -7, it is important to talk to your doctor about your prognosis and treatment options. There are a number of new treatments for AML that are being developed, and your doctor may be able to recommend a treatment that is right for you. | The prognosis for a patient with AML who has a karyotype of t(1,2) and -7 at diagnosis is poor. The 5-year survival rate for patients with this karyotype is typically less than 20%.  The t(1,2) translocation is a chromosomal abnormality that results in the fusion of two genes, AML1 and RUNX1. This fusion gene is thought to be involved in the development of AML. The -7 deletion is another chromosomal abnormality that is often seen in AML. This deletion results in the loss of a gene called RUNX1, which is also thought to be involved in the development of AML.  The combination of the t(1,2) translocation and the -7 deletion is a very poor prognostic factor for patients with AML. These patients are more likely to relapse after allogeneic HSCT and have a shorter overall survival.  However, there are a number of factors that can affect the prognosis for patients with AML, including the patient's age, the type of AML, and the patient's response to treatment. Additionally, new treatments for AML are being developed, and these may improve the prognosis for patients with this karyotype.  If you are a patient with AML who has a karyotype of t(1,2) and -7, it is important to talk to your doctor about your prognosis and treatment options. There are a number of new treatments for AML that are being developed, and your doctor may be able to recommend a treatment that is right for you.  Here are some additional information about t(1,2) and -7:  * t(1,2) is a chromosomal abnormality that results in the fusion of two genes, AML1 and RUNX1.  * -7 deletion is another chromosomal abnormality that is often seen in AML.  * The combination of t(1,2) and -7 is a very poor prognostic factor for patients with AML.  * The 5-year survival rate for patients with this karyotype is typically less than 20%.  * However, new treatments for AML are being developed, and these may improve the prognosis for patients with this karyotype.  I hope this information is helpful. Please let me know if you have any other questions. | The prognosis for a patient with AML who has a karyotype of t(1,2) and -7 at diagnosis is poor. The 5-year survival rate for patients with this karyotype is typically less than 20%.  The t(1,2) translocation is a chromosomal abnormality that results in the fusion of two genes, AML1 and RUNX1. This fusion gene is thought to be involved in the development of AML. The -7 deletion is another chromosomal abnormality that is often seen in AML. This deletion results in the loss of a gene called RUNX1, which is also thought to be involved in the development of AML.  The combination of the t(1,2) translocation and the -7 deletion is a very poor prognostic factor for patients with AML. These patients are more likely to relapse after allogeneic HSCT and have a shorter overall survival.  However, there are a number of factors that can affect the prognosis for patients with AML, including the patient's age, the type of AML, and the patient's response to treatment. Additionally, new treatments for AML are being developed, and these may improve the prognosis for patients with this karyotype.  If you are a patient with AML who has a karyotype of t(1,2) and -7, it is important to talk to your doctor about your prognosis and treatment options. There are a number of new treatments for AML that are being developed, and your doctor may be able to recommend a treatment that is right for you.  Here are some additional information about t(1,2) and -7:  * t(1,2) is a chromosomal abnormality that results in the fusion of two genes, AML1 and RUNX1.  * -7 deletion is another chromosomal abnormality that is often seen in AML.  * The combination of t(1,2) and -7 is a very poor prognostic factor for patients with AML.  * The 5-year survival rate for patients with this karyotype is typically less than 20%.  * However, new treatments for AML are being developed, and these may improve the prognosis for patients with this karyotype.  I hope this information is helpful. Please let me know if you have any other questions. |
| C17 | The patient is most likely experiencing **idiopathic refractory ascites** (IRA), a rare but serious complication of allogeneic HSCT. IRA is characterized by the rapid accumulation of fluid in the abdomen, which can lead to weight gain, increased bilirubin levels, and painful hepatomegaly.  The exact cause of IRA is unknown, but it is thought to be due to a combination of factors, including:  * **Graft-versus-host disease (GVHD)**. GVHD is a condition in which the donor's immune cells attack the patient's tissues. In the case of IRA, GVHD can damage the liver, leading to fluid retention.  * **Immunosuppressive drugs**. The immunosuppressive drugs that are used to prevent GVHD can also increase the risk of IRA.  * **Underlying liver disease**. Patients with underlying liver disease, such as hepatitis C or cirrhosis, are at an increased risk of developing IRA.  Here are some transplant-related factors that have been suggested to increase the risk for IRA:  * **HLA mismatch**. Patients who are more mismatched with their donors are at an increased risk of developing IRA.  * **High-dose conditioning**. Patients who receive high-dose conditioning regimens are at an increased risk of developing IRA.  * **GVHD**. Patients who develop GVHD are at an increased risk of developing IRA.  * **Immunosuppressive drugs**. Some immunosuppressive drugs, such as cyclosporine and tacrolimus, are thought to increase the risk of developing IRA.  It is important to note that the risk of developing IRA varies from patient to patient. Some patients may be at an increased risk of developing IRA, while others may not be. The patient's doctor will be able to assess the patient's individual risk factors and discuss the best course of treatment.  I hope this information is helpful. Please let me know if you have any other questions. | The patient is most likely experiencing **idiopathic refractory ascites** (IRA), a rare but serious complication of allogeneic HSCT. IRA is characterized by the rapid accumulation of fluid in the abdomen, which can lead to weight gain, increased bilirubin levels, and painful hepatomegaly.  The exact cause of IRA is unknown, but it is thought to be due to a combination of factors, including:  * **Graft-versus-host disease (GVHD)**. GVHD is a condition in which the donor's immune cells attack the patient's tissues. In the case of IRA, GVHD can damage the liver, leading to fluid retention.  * **Immunosuppressive drugs**. The immunosuppressive drugs that are used to prevent GVHD can also increase the risk of IRA.  * **Underlying liver disease**. Patients with underlying liver disease, such as hepatitis C or cirrhosis, are at an increased risk of developing IRA.  Here are some transplant-related factors that have been suggested to increase the risk for IRA:  * **HLA mismatch**. Patients who are more mismatched with their donors are at an increased risk of developing IRA.  * **High-dose conditioning**. Patients who receive high-dose conditioning regimens are at an increased risk of developing IRA.  * **GVHD**. Patients who develop GVHD are at an increased risk of developing IRA.  * **Immunosuppressive drugs**. Some immunosuppressive drugs, such as cyclosporine and tacrolimus, are thought to increase the risk of developing IRA.  It is important to note that the risk of developing IRA varies from patient to patient. Some patients may be at an increased risk of developing IRA, while others may not be. The patient's doctor will be able to assess the patient's individual risk factors and discuss the best course of treatment.  Here are some additional information about IRA:  * IRA is a rare condition, but it is more common in patients who have undergone allogeneic HSCT.  * IRA can be a serious condition, but it is often treatable.  * The treatment for IRA will vary depending on the individual patient's circumstances.  I hope this information is helpful. Please let me know if you have any other questions. | The patient is most likely experiencing **idiopathic refractory ascites** (IRA), a rare but serious complication of allogeneic HSCT. IRA is characterized by the rapid accumulation of fluid in the abdomen, which can lead to weight gain, increased bilirubin levels, and painful hepatomegaly.  The exact cause of IRA is unknown, but it is thought to be due to a combination of factors, including:  * **Graft-versus-host disease (GVHD)**. GVHD is a condition in which the donor's immune cells attack the patient's tissues. In the case of IRA, GVHD can damage the liver, leading to fluid retention.  * **Immunosuppressive drugs**. The immunosuppressive drugs that are used to prevent GVHD can also increase the risk of IRA.  * **Underlying liver disease**. Patients with underlying liver disease, such as hepatitis C or cirrhosis, are at an increased risk of developing IRA.  Here are some transplant-related factors that have been suggested to increase the risk for IRA:  * **HLA mismatch**. Patients who are more mismatched with their donors are at an increased risk of developing IRA.  * **High-dose conditioning**. Patients who receive high-dose conditioning regimens are at an increased risk of developing IRA.  * **GVHD**. Patients who develop GVHD are at an increased risk of developing IRA.  * **Immunosuppressive drugs**. Some immunosuppressive drugs, such as cyclosporine and tacrolimus, are thought to increase the risk of developing IRA.  It is important to note that the risk of developing IRA varies from patient to patient. Some patients may be at an increased risk of developing IRA, while others may not be. The patient's doctor will be able to assess the patient's individual risk factors and discuss the best course of treatment.  Here are some additional information about IRA:  * IRA is a rare condition, but it is more common in patients who have undergone allogeneic HSCT.  * IRA can be a serious condition, but it is often treatable.  * The treatment for IRA will vary depending on the individual patient's circumstances.  The transplant-related factors that you have mentioned are all associated with an increased risk of developing IRA. However, it is important to note that not all patients who experience these factors will develop IRA. The risk of developing IRA is also influenced by other factors, such as the patient's age, underlying health conditions, and the type of allogeneic HSCT that they receive.  If you are concerned about the risk of developing IRA, you should talk to your doctor. They will be able to assess your individual risk factors and discuss the best course of treatment. |
| C18 | The dosing of donor lymphocyte infusion (DLI) depends on a number of factors, including:  * **The patient's underlying condition:** The type of cancer or other condition that the patient is being treated for can affect the dosing of DLI. For example, patients with acute myeloid leukemia (AML) typically receive higher doses of DLI than patients with chronic myeloid leukemia (CML).  * **The patient's response to previous DLI:** If the patient has received DLI in the past, the dosing of the next infusion will likely be based on their response to the previous infusion. For example, if the patient responded well to a previous low-dose DLI, they may be given a higher dose of DLI the next time.  * **The patient's risk of graft-versus-host disease (GvHD):** GvHD is a complication of DLI that can occur when the donor cells attack the recipient's tissues. The risk of GvHD is increased with higher doses of DLI. Therefore, the dosing of DLI is typically balanced between the need to treat the patient's underlying condition and the risk of GvHD.  * **The patient's overall health:** The patient's overall health, including their age, weight, and organ function, can also affect the dosing of DLI. For example, older patients or patients with impaired organ function may be given lower doses of DLI.  The decision of how much DLI to give a patient is ultimately made by the patient's doctor, who will consider all of the factors mentioned above.  Here are some additional information about donor lymphocyte infusion:  * **What is DLI?** DLI stands for donor lymphocyte infusion. It is a procedure in which donor lymphocytes are infused into a patient who has undergone a hematopoietic stem cell transplant (HSCT). The goal of DLI is to help the patient's immune system fight off cancer cells or other diseases.  * **How does DLI work?** DLI works by introducing donor lymphocytes into the patient's body. These donor lymphocytes then attack the patient's cancer cells or other diseased cells. This can help to improve the patient's response to treatment and increase their chances of survival.  * **What are the risks of DLI?** The risks of DLI include GvHD, infection, and bleeding. GvHD is a serious complication that can occur when the donor lymphocytes attack the patient's tissues. Infection and bleeding are also risks of DLI, but they are typically less serious than GvHD.  I hope this helps! Let me know if you have any other questions. | The dosing of donor lymphocyte infusion (DLI) depends on a number of factors, including:  * **The patient's underlying condition:** The type of cancer or other condition that the patient is being treated for can affect the dosing of DLI. For example, patients with acute myeloid leukemia (AML) typically receive higher doses of DLI than patients with chronic myeloid leukemia (CML).  * **The patient's response to previous DLI:** If the patient has received DLI in the past, the dosing of the next infusion will likely be based on their response to the previous infusion. For example, if the patient responded well to a previous low-dose DLI, they may be given a higher dose of DLI the next time.  * **The patient's risk of graft-versus-host disease (GvHD):** GvHD is a complication of DLI that can occur when the donor cells attack the recipient's tissues. The risk of GvHD is increased with higher doses of DLI. Therefore, the dosing of DLI is typically balanced between the need to treat the patient's underlying condition and the risk of GvHD.  * **The patient's overall health:** The patient's overall health, including their age, weight, and organ function, can also affect the dosing of DLI. For example, older patients or patients with impaired organ function may be given lower doses of DLI.  In addition to these factors, the dosing of DLI may also be affected by the following:  * **The type of donor cells being used:** DLI can be made from either peripheral blood stem cells (PBSCs) or bone marrow. PBSCs are typically given in higher doses than bone marrow.  * **The method of DLI administration:** DLI can be given as a single infusion or as multiple infusions. Single infusions typically have a higher risk of GvHD than multiple infusions.  The decision of how much DLI to give a patient is ultimately made by the patient's doctor, who will consider all of the factors mentioned above.  I hope this helps! Let me know if you have any other questions. | The dosing of donor lymphocyte infusion (DLI) depends on a number of factors, including:  * **The patient's underlying condition:** The type of cancer or other condition that the patient is being treated for can affect the dosing of DLI. For example, patients with acute myeloid leukemia (AML) typically receive higher doses of DLI than patients with chronic myeloid leukemia (CML).  * **The patient's response to previous DLI:** If the patient has received DLI in the past, the dosing of the next infusion will likely be based on their response to the previous infusion. For example, if the patient responded well to a previous low-dose DLI, they may be given a higher dose of DLI the next time.  * **The patient's risk of graft-versus-host disease (GvHD):** GvHD is a complication of DLI that can occur when the donor cells attack the recipient's tissues. The risk of GvHD is increased with higher doses of DLI. Therefore, the dosing of DLI is typically balanced between the need to treat the patient's underlying condition and the risk of GvHD.  * **The patient's overall health:** The patient's overall health, including their age, weight, and organ function, can also affect the dosing of DLI. For example, older patients or patients with impaired organ function may be given lower doses of DLI.  In addition to these factors, the dosing of DLI may also be affected by the following:  * **The type of donor cells being used:** DLI can be made from either peripheral blood stem cells (PBSCs) or bone marrow. PBSCs are typically given in higher doses than bone marrow.  * **The method of DLI administration:** DLI can be given as a single infusion or as multiple infusions. Single infusions typically have a higher risk of GvHD than multiple infusions.  The decision of how much DLI to give a patient is ultimately made by the patient's doctor, who will consider all of the factors mentioned above.  Here are some additional information about donor lymphocyte infusion:  * **What is DLI?** DLI stands for donor lymphocyte infusion. It is a procedure in which donor lymphocytes are infused into a patient who has undergone a hematopoietic stem cell transplant (HSCT). The goal of DLI is to help the patient's immune system fight off cancer cells or other diseases.  * **How does DLI work?** DLI works by introducing donor lymphocytes into the patient's body. These donor lymphocytes then attack the patient's cancer cells or other diseased cells. This can help to improve the patient's response to treatment and increase their chances of survival.  * **What are the risks of DLI?** The risks of DLI include GvHD, infection, and bleeding. GvHD is a serious complication that can occur when the donor lymphocytes attack the patient's tissues. Infection and bleeding are also risks of DLI, but they are typically less serious than GvHD.  I hope this helps! Let me know if you have any other questions. |
